# Development of a Non-Hydroxamate Dual Matrix Metalloproteinase (MMP)-7/-13 Inhibitor

**DOI:** 10.3390/molecules22091548

**Published:** 2017-09-14

**Authors:** Thomas Fischer, Rainer Riedl

**Affiliations:** Center for Organic and Medicinal Chemistry, Institute of Chemistry and Biotechnology, Zurich University of Applied Sciences ZHAW, Einsiedlerstrasse 31, 8820 Wädenswil, Switzerland; thomas.fischer@zhaw.ch

**Keywords:** matrix metalloproteinase inhibitor, polypharmacology, organic synthesis, drug discovery, structure activity relationship

## Abstract

Matrix metalloproteinase 7 (MMP-7) is a member of the MMP superfamily and is able to degrade extracellular matrix proteins such as casein, gelatin, fibronectin and proteoglycan. MMP-7 is a validated target for the development of small molecule drugs against cancer. MMP-13 is within the enzyme class the most efficient contributor to type II collagen degeneration and is a validated target in arthritis and cancer. We have developed the dual MMP-7/-13 inhibitor ZHAWOC6941 with IC_50_-values of 2.2 μM (MMP-7) and 1.2 μM (MMP-13) that is selective over a broad range of MMP isoforms. It spares MMP-1, -2, -3, -8, -9, -12 and -14, making it a valuable modulator for targeted polypharmacology approaches.

## 1. Introduction

Matrix metalloproteinases (MMPs) are a family of calcium- and zinc-dependent endopeptidases able to metabolize components of the extracellular matrix (ECM) [1]. In healthy organisms, the activity of MMPs is strongly regulated by the tissue inhibitors of metalloproteinases (TIMPs) [2]. An imbalance in this network can lead to a series of serious diseases including, but not exclusively, different forms of cancer or arthritis [3,4,5,6,7,8]. The enzyme class has been in the focus of the pharmaceutical industry for decades since their first description by Gross and Lapiere [9,10]. Many different MMP inhibitors are known to date, demonstrating a range of potency and selectivity [11,12,13,14,15,16,17,18,19,20,21]. Early inhibitors of the target family incorporated a hydroxamic acid moiety as a strong metal chelating group interacting with the catalytic zinc which is conserved in all MMP isoforms [22]. This led to potent inhibitors, which however did not display satisfying selectivity profiles [23]. In clinical trials those inhibitors failed due to painful side effects such as the joint-stiffening musculoskeletal syndrome (MSS) [24,25]. Further development led to more sophisticated inhibitors with superior selectivity profiles compared to hydroxamate derivatives. For some MMPs very selective inhibitors are available nowadays. For example MMP-13, the key player in collagen degradation and a valid target for arthritis and cancer [7,26], can be inhibited selectively and with high affinity with a variety of ligands [27,28,29].

Matrix metalloproteinase 7 (MMP-7) is a MMP-family member that differs from most of the other isoforms because it lacks the haemopexin-like domain, found in all MMPs except for MMP-7, MMP-23 and MMP-26 [4]. It is capable of activating the pro-forms of MMP-2 and MMP-9 [30]. Overall and Kleifeld have reviewed the role of MMPs as drug targets and anti-targets in relation to cancer therapy [31]. Several investigations indicate that MMP-7 is a validated drug target related to cancer. It is associated with prostate cancer [32,33], tumor proliferation [34], invasion of ovarian cancer [30], gastric cancer [35] as well as colorectal cancer [36]. In mouse models the administration of inhibitors addressing MMP-7 reduced the number of intestinal polyps [37]. Those findings suggest that inhibitors of MMP-7 that spare other MMP isoforms could be promising compounds for the treatment of the diseases.

**Figure 1:**
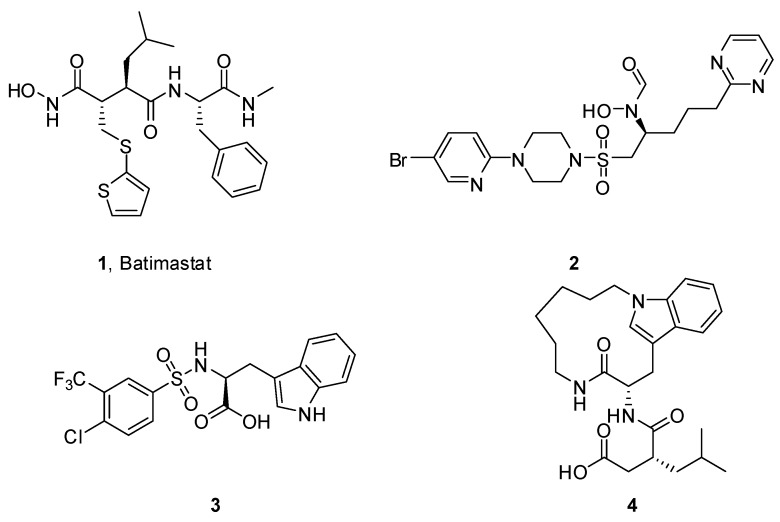
Structures of different MMP-7 inhibitors.

Compounds **1** (batimastat) and **2** (PDB code: TQJ) (Figure 1) are potent MMP inhibitors of the hydroxamate class that inhibit MMP-7 with IC_50_-values of 6 nM (**1**) [38] and 79 nM (**2**) [39] but also inhibit other members of the target class with the same potency leading to a lack in selectivity [38,39,40]. The non-hydroxamate based compounds **3** (PDB code: TQI) and **4** (PDB code: RSS) are weaker inhibitors with IC_50_-values of 10 μM (**3**) [39] and 850 nM (**4**) [41]. Compound **3** shows 10-fold selectivity over MMP-1 and MMP-14 [39].

**Figure 2:**
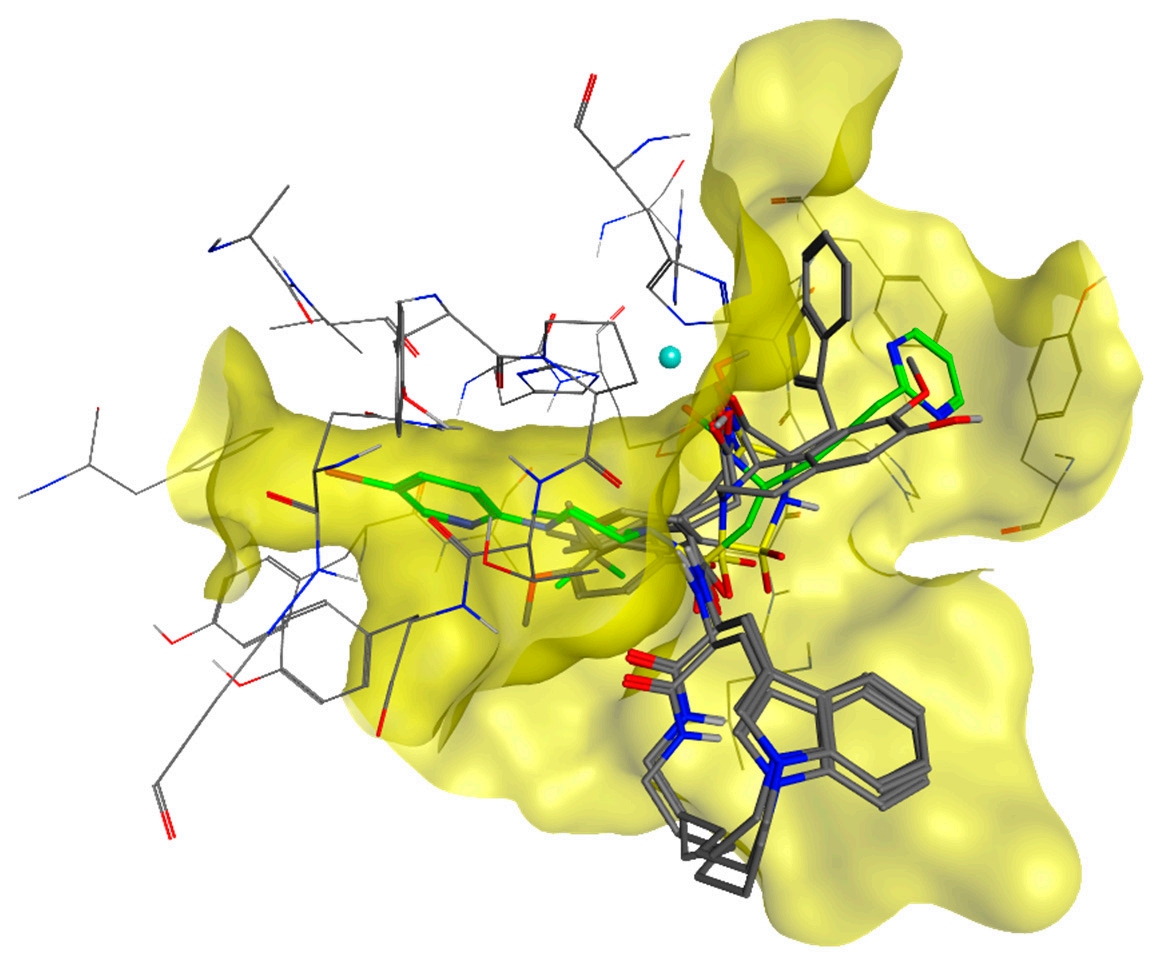
Overlay of all known MMP-7 inhibitors, found in the Protein Data Bank, within the active site. PDB codes: 1MMP, 1MMR, 1MMQ, 2DDY, 2Y6C, 2Y6D (ligand from 2Y6D depicted in green).

As displayed in Figure 2, all co-crystallized MMP-7 inhibitors found in the Protein Data Bank populate the active site, but only **2** (PDB 2Y6D [39], green) penetrates the S_1_’ channel, which is responsible for selective binding among different subtypes in this enzyme class [42].

**Figure 3:**
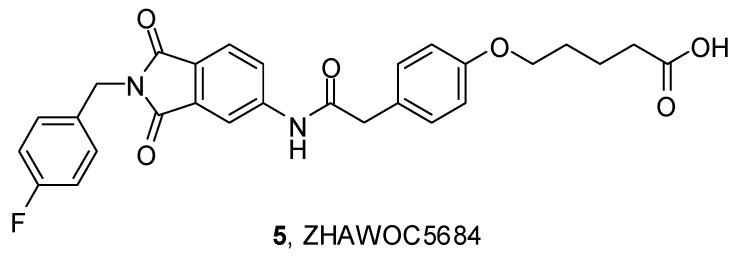
Previously identified potent and selective MMP-13 inhibitor.

Work conducted previously in our lab led to the potent (IC_50_ = 6 nM) and selective MMP-13 inhibitor **5** (Figure 3) that was selective over all tested subtypes of the target class [43].

Herein, we present an approach that enabled us to modify the characteristics of the MMP-13 inhibitor **5** towards a dual MMP-7/-13 inhibitor, while conserving the selectivity profile over other MMP isoforms.

## 2. Results

### 2.1. Strategy of Inhibitor Development

Compound **5** was originally designed as an MMP-13 inhibitor and displayed very high potency and selectivity against the target enzyme with an IC_50_-value of 6 nM.

The selectivity profile of **5** (Table 1) demonstrated high selectivity against all examined MMPs. Nevertheless, initial inhibitory activity against MMP-7 was detected at an inhibitor concentration of 10 µM (Table 1). Consecutive dose dependent measurements revealed an IC_50_-value of 15.7 µM against MMP-7 resulting in a >2600 fold selectivity for MMP-13 over MMP-7. This finding motivated us to investigate structural modifications of **5** with the intention of improving the affinity against MMP-7 while maintaining the appealing selectivity profile over MMP family members being anti-targets in cancer therapy such as MMP (-3, -8, -9, -12 and -14) [31].

**Figure 4:**
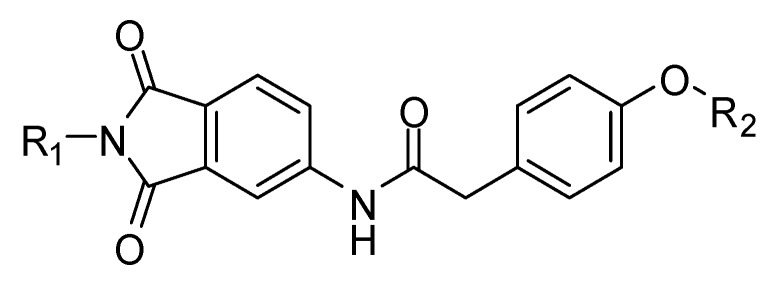
Sites of modification R_1_ and R_2_.

The initial scaffold was modified at the positions indicated by R_1_ and R_2_ in Figure 4 to probe the effects of structural changes on the enzymatic activity of MMP-7 and the selectivity profile over other MMP isoforms. At position R_1_ we examined the initial para fluorinated benzyl residue along with a non-fluorinated benzyl and a methyl substituent with the aim of probing the influence of electronic properties and size of the moiety tolerated for optimal interactions. As the S_1_’ pocket of MMP-7 is smaller than in MMP-13, a smaller residue in this area is hypothesized to rather fit to the limited space in MMP-7. At the R_2_ position, we varied the length of the aliphatic linker between the phenolic oxygen and the carboxylic acid head group from 1–9 CH_2_ entities for the identification of the optimal chain length for the inhibition of MMP-7.

### 2.2. Chemistry

**Scheme 1:**
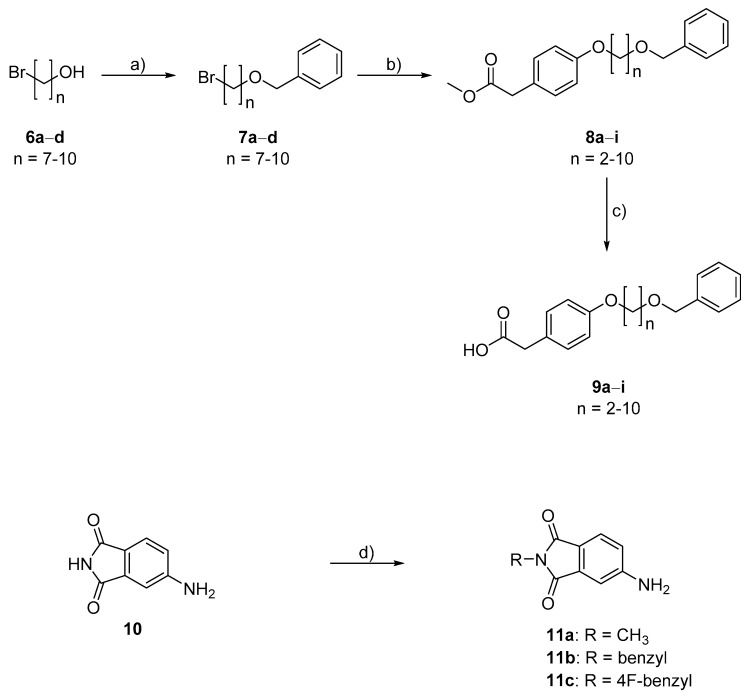
Synthesis of intermediates **9** and **11**. *Reagents, conditions and yields*: (**a**) Bromoalkyl alcohol, NaH, benzyl bromide, THF, RT, 3 d, **7a**: 94%, **7b**: 91%, **7c**: 90%, **7d**: 76%; (**b**) methyl 2-(4-hydroxyphenyl) acetate, Cs_2_CO_3_, DMF, RT, 18 h, **8a**: 84%, **8b**: 77%, **8c**: 68%, **8d**: 90%, **8e**: 74%, **8f**: 78%, **8g**: 69%, **8h**: 55%, **8i**: 49%; (**c**) KOH 10% in H_2_O, methanol, RT, 30 min., **9a**: 95%, **9b**: 98%, **9c**: 90%, **9d**: 91%, **9e**: 73%, **9f**: 94%, **9g**: 96%, **9h**: 81%, **9i**: 99%; (**d**) 4-aminophthalimide **10**, KOH, alkyl halide, DMF, RT, 18 h, **11a**: 92%, **11b**: 59%, **11c**: 68%.

The synthetic routes to the intermediates **9** and **11** are illustrated in Scheme 1. The right hand side fragment could be synthesized from the benzylated bromoalkylalcohol. In case this intermediate could not be purchased, it was synthesized through protection of the according bromoalkylalcohol with benzylbromide. A nucleophilic substitution reaction between the benzylated bromoalkylalcohols and the commercially available methyl 2-(4-hydroxyphenyl)acetate led to the intermediates **8a**–**i** and by a consecutive saponification with potassium hydroxide to the building blocks **9a**–**i**. The left hand side fragments **11a**–**c** were synthesized by alkylation of the commercially available 4-aminophthalimide **10** with the corresponding alkyl halide.

**Scheme 2:**
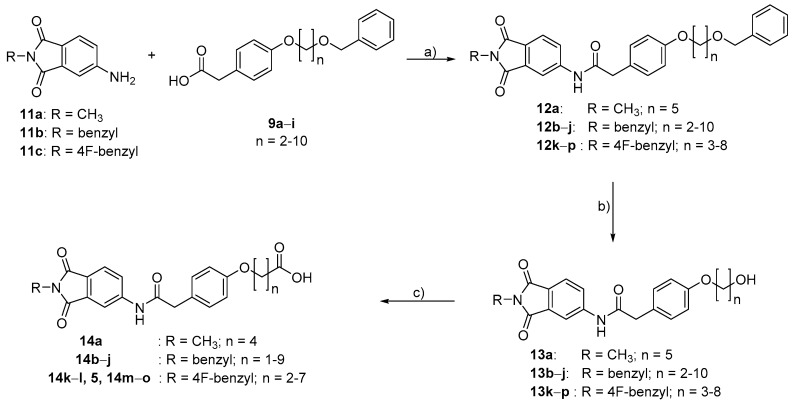
Synthesis of the test compounds **5** and **14a-o**. *Reagents, conditions and yields*: (**a**) SOCl_2_, DIPEA, THF, RT, 2 h, **12a**: 11%, **12b**: 80%, **12c**: 62%, **12d**: 64%, **12e**: 44%, **12f**: 34%, **12g**: 18%, **12h**: 38%, **12i**: 63%, **12j**: 46%, **12k**: 72%, **12l**: 55%, **12m**: 58%, **12n**: 36%, **12o**: 47%, **12p**: 57%; (**b**) TMSI, CH_2_Cl_2_, RT, 2h, **13a**: 22%; Pd/C 10%, ethanol, hydrogen atmosphere, RT, 2h, **13b**: 39%, **13c**: 71%, **13d**: 60%, **13e**: 24%, **13f**: 6%, **13g**: 24%, **13h**: 59%, **13i**: 30%, **13j**: 18%, **13k**: 34%, **13l**: 15%, **13m**: 44%, **13n**: 26%, **13o**: 53%, **13p**: 24%; (**c**) TEMPO, sodium phosphate 0.67 M in H_2_O pH 6.7, NaClO_2_, NaOCl, ACN, 40 °C, 4 h, **14a**: 36%, **14b**: 38%, **14c**: 86%, **14d**: 67%, **14e**: 62%, **14f**: 96%, **14g**: 55%, **14h**: 50%, **14i**: 39%, **14j**: 33%, **14k**: 62%, **14l**: 97%, **5**: 50%, **14m**: 59%, **14n**: 56%, **14o**: 30%.

As displayed in Scheme 2, the aniline derivatives **11a**–**c** and the carboxylic acids **9a**–**i** were coupled to form the intermediates **12a**–**p** via the formation of the acid chloride. By debenzylation of the protected alcohol moieties with TMSI for **13a** and hydrogen for **13b**–**p**, and consecutive oxidation employing TEMPO, the final compounds **5** and **14a**–**o** could be obtained as the free carboxylic acids in moderate to good yields. Complete analytical data of the synthesized compounds and IC_50_-curves are shown in the Supplementary Materials.

### 2.3. Biological Evaluation

The synthesized compounds were examined in in vitro assays to determine their inhibitory potential against MMP-7 and MMP-13. Table 2 depicts the structure activity relationship of the novel inhibitors.

Compound **14i** could be identified as the most potent MMP-7 inhibitor within the series displaying an IC_50_-value of 2.2 µM (Figure S1a). In combination with a remaining affinity towards MMP-13 (IC_50_-value of 1.2 µM, Figure S1b), this qualifies **14i** as a dual MMP-7/MMP-13 inhibitor in the low micromolar range.

Consecutively, we tested inhibitor **14i** in single dose assays against a set of MMP isoforms in order to determine its selectivity profile. Table 3 shows the remaining enzymatic activity at an inhibitor concentration of 10 µM.

The remaining enzymatic activities in the range of >70—100% at an inhibitor concentration of 10 µM indicate IC_50_-values against those enzymes higher than 10 µM.

## 3. Discussion

The initial compound **5** was modified with the aim of improving its inhibitory potential against MMP-7. A first modification led to compound **14a**, here the *para*-fluorobenzyl residue has been replaced by a methyl group resulting in a total loss of inhibition. This indicated that an aromatic moiety is important for protein ligand interactions such as π-stacking to aromatic amino acids in this area of the receptor. Therefore we decided to conserve a benzyl group in this area of the molecule. We crafted two series of compounds one with the original fluorinated benzyl residue and the second with a non-fluorinated benzyl moiety. In both series the length of the aliphatic linker between the phenolic oxygen and the carboxylic acid head group was varied. The series containing a fluorine atom was equipped with different linkers ranging from 2 to 7 CH_2_ entities in length. Here, a tendency could be observed that longer linkers corresponded to better inhibition of MMP-7 with an optimum at six carbons in **14n**, which demonstrated remaining enzymatic activity of 47.5% at 10 µM inhibitor concentration. A similar trend could be observed for the ensemble lacking the fluorine atom. A chain length between one and four carbons was not tolerated and resulted in very low to no inhibition. With longer linkers, better inhibition could be achieved with an optimum at 8 CH_2_ entities. The most potent inhibitor of the series **14i** displayed an IC_50_-value of 2.2 μM against MMP-7 and 1.2 μM against MMP-13. Compared to MMP-7 inhibitors in literature, our compound is with a low single digit micromolar IC_50_ amongst the most potent non-hydroxamate compounds [39,41,44], and to the best of our knowledge unique in displaying selectivity over a wide range of other MMP isoforms [39,41].

**Figure 5:**
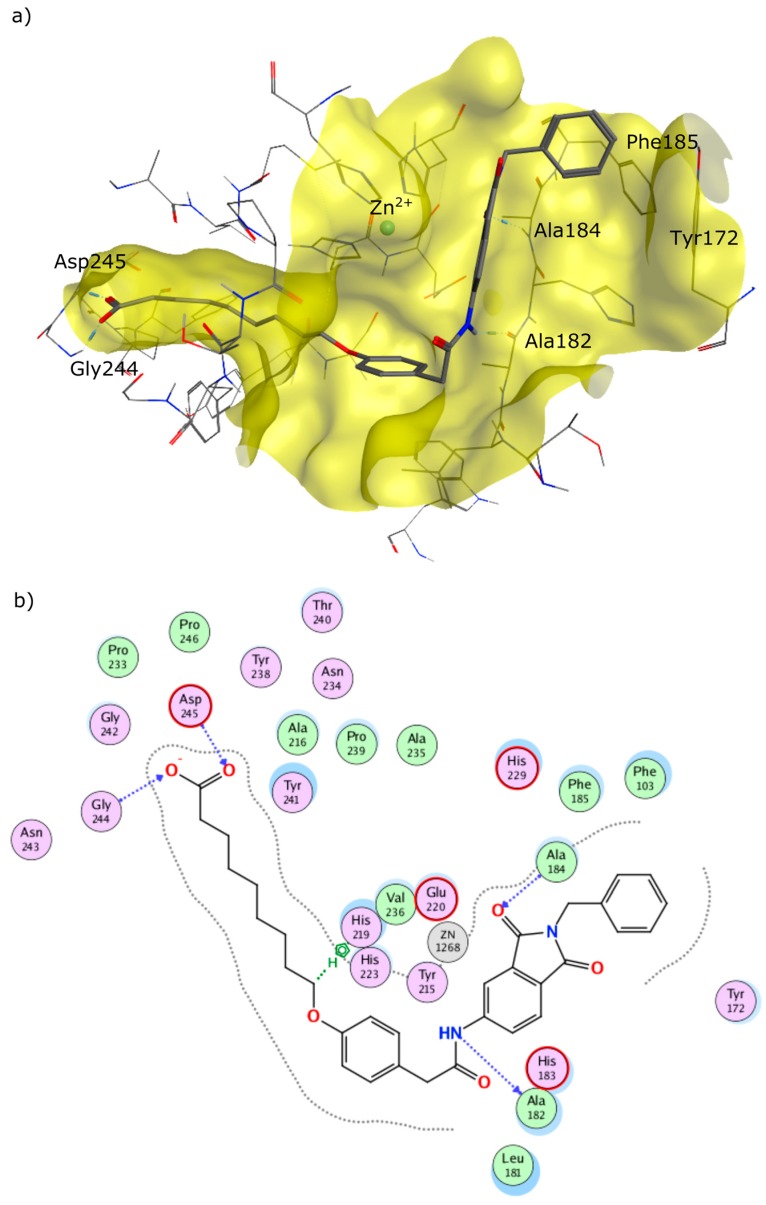

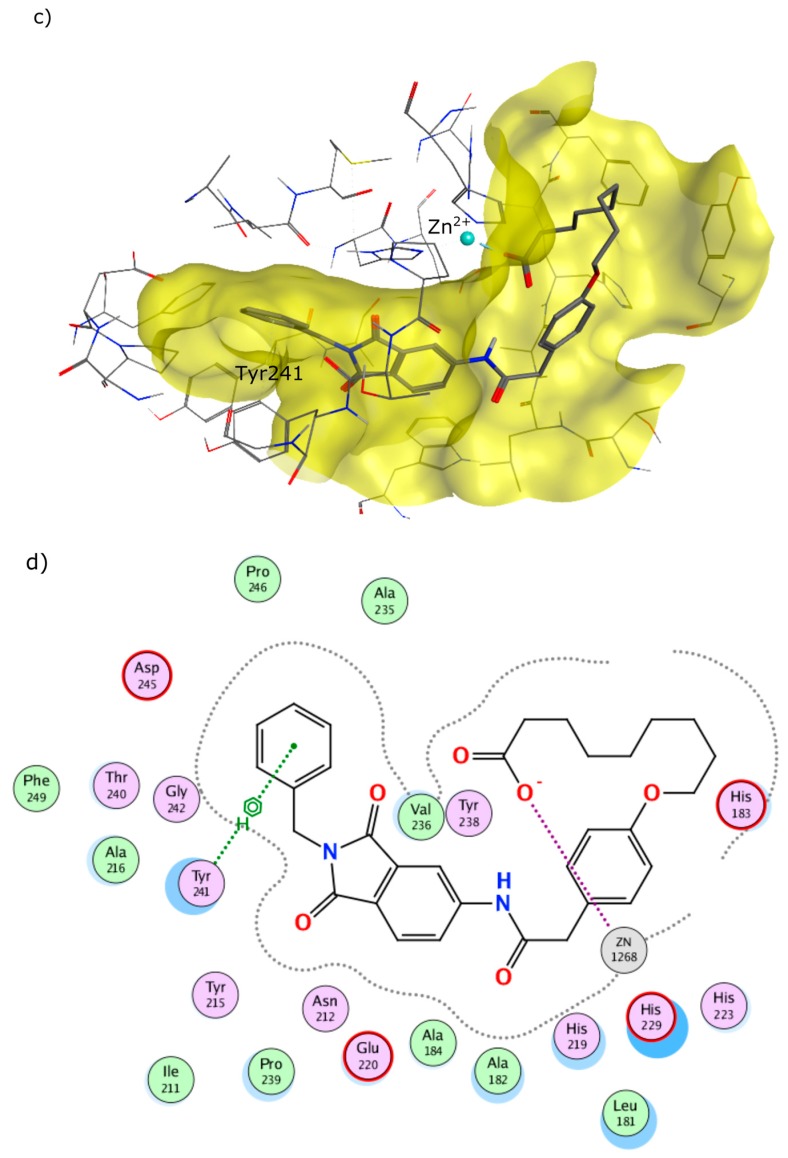

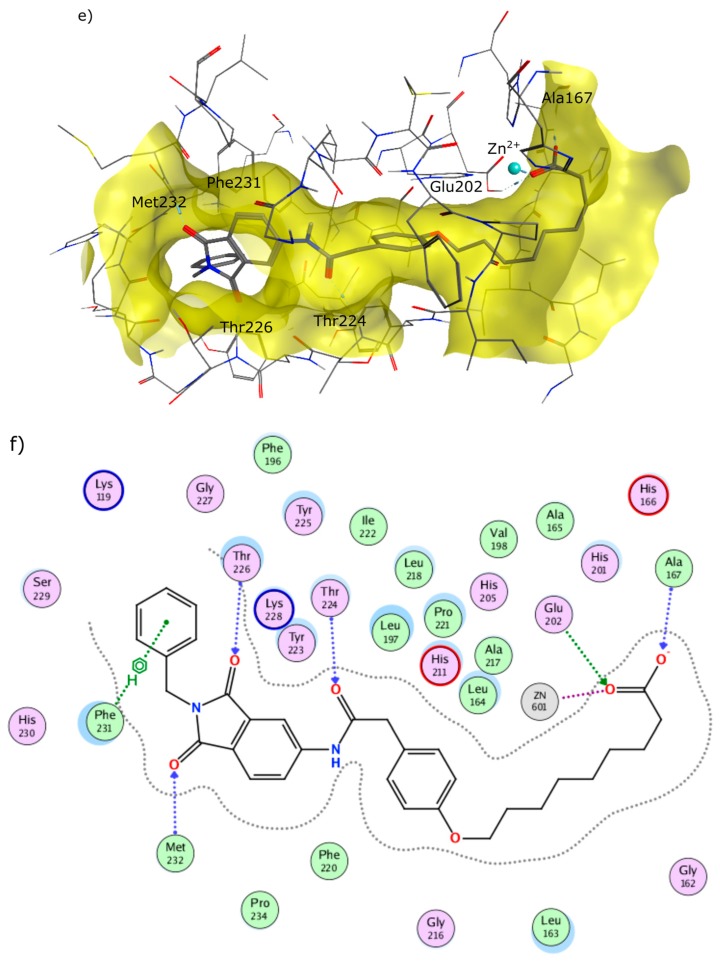
Potential binding modes of **14i** in MMP-7 and MMP-13, (**a**) non zinc-binding mode in MMP-7; (**b**) interaction map of the non zinc-binding pose in MMP-7; (**c**) zinc-binding mode in MMP-7; (**d**) interaction map of the zinc-binding pose in MMP-7; (**e**) zinc-binding docking pose in MMP-13; (**f**) interaction map of the zinc-binding pose in MMP-13.

We performed molecular docking experiments to examine potential binding modes of **14i** with MMP-7 and MMP-13. The water molecules present in the used co-crystal structures (PDB: 2Y6D [39] for MMP-7 and 2OW9 [45] for MMP-13) were set to inactive with respect to the force field prior to the docking experiment to enable the ligand to populate all space available in the active site. The water molecule which is expected to populate the remaining coordination site at the zinc(II) ion (Figure 5a) is not present in the co-crystal structure (2Y6D) used for the docking experiments as this is replaced by the zinc-chelating ligand within the complex.

For MMP-7 two low energy docking poses were found with significantly diverse binding modes. As visible in Figure 5a (the non zinc-binding mode), the ligand interacts with four amino acids of the receptor by the establishment of hydrogen bonds to Ala182, Ala184, Gly244 and Asp245. In this pose the ligand does not coordinate to the catalytic zinc(II) ion. The aliphatic linker between the scaffold and the carboxylic acid populates the hydrophobic S_1_’ channel and the phthalimide blocks the groove at the active site. The benzyl residue can be engaged in π-interactions with Tyr172 and Phe185. In the second pose, depicted in Figure 5c (the zinc-binding mode), the benzyl moiety populates the S_1_’ pocket and interacts with Tyr241 via a face to edge aromatic interaction.

The *para*-substituted phenyl ring and the aliphatic linker chain populate the cleft where the substrate is recognized and block the active site. The carboxylic acid head group ligates the catalytic zinc(II) ion, which is known to be a strong interaction leading to enhanced affinity between the ligand and the target enzyme. Both of the proposed binding modes qualify as reasonable explanations for the appealing affinity towards MMP-7 without the need of hydroxamic acids as zinc-binding fragments and can be used for further structure-based optimization of the inhibitor. Docking of **14i** into MMP-13 (Figure 5e) revealed a binding mode where the phthalimide oxygens and the amide oxygen form hydrogen bonds to the backbone amino acids Thr224, Thr226 and Met232 and the unsubstituted benzyl ring populates the S_1_’* selectivity loop, buried deep in the S_1_’ channel. The carboxylate head group chelates the catalytic zinc(II) ion and interacts with Ala167 and Glu202 by the formation of hydrogen bonds.

## 4. Materials and Methods

### 4.1. General Information

All reagents and solvents were purchased from Sigma Aldrich (Buchs, Switzerland), TCI (Zwijndrecht, Belgium) or Fluorochem (Hadfield, UK) and used as received. Solvents were stored over 4 Å molecular sieves. NMR spectra were recorded at 25 °C on an AVANCE III HD 500 One Bay spectrometer (Bruker, Fällanden, Switzerland) with a magnetic field of 11.75 T. For ^1^H-NMR spectra a frequency of 500 MHz resulted. Chemical shifts are reported in ppm from tetramethylsilane as internal standard. Data are reported as follows: chemical shift, multiplicity (s = singlet, d = doublet, t = triplet, q = quartet, quint. = quintet, br. = broad, m = multiplet), coupling constants (Hz), integration. For ^13^C-NMR spectra a frequency of 125 MHz resulted. Chemical shifts are reported in ppm from tetramethylsilane as internal standard. The multiplicities of the signals were determined by DEPT measurements. Low-resolution mass spectrometry was performed on a MSQ Plus device (Thermo Scientific, Reinach, Switzerland). High-resolution mass spectrometry was performed on an 6530 Q-TOF (Agilent Technologies, Basel, Switzerland). NMR spectra, HRMS spectra and IC_50_ curves can be found in the Supplementary Materials.

### 4.2. Chemistry

*5-Amino-2-methyl-2,3-dihydro-1H-isoindole-1,3-dione* (**11a**; ZHAWOC3444): Potassium hydroxide (0.35 g, 6.17 mmol) was added to a solution of 4-aminophthalimide **10** (1.00 g, 6.17 mmol) in dimethylformamide (30 mL) and the mixture was stirred at ambient temperature for 2 h. Iodomethane (0.88 g, 6.17 mmol) was added and it was stirred for another 18 h at the same temperature. Water (50 mL) and ethyl acetate (50 mL) was added and the resulting phases were separated. The organic phase was washed with brine, dried over sodium sulfate and concentrated in vacuum. Purification by chromatography on silica gel (Gradient: 0–100% ethyl acetate in cyclohexane) afforded the title compound **11a** as a yellow solid (1.00 g, 92% yield): ^1^H-NMR (DMSO-*d*_6_): δ = 7.46 (d, *J* = 8.24 Hz, 1H), 6.90 (d, *J* = 2.00 Hz, 1H), 6.77 (dd, *J* = 8.24 Hz, 2.00 Hz, 1H), 6.42 (br. s, 2H), 2.94 (s, 3H) ppm. ^13^C-NMR (DMSO-*d*_6_): δ = 168.87, 168.57, 155.27, 135.10, 125.17, 117.33, 116.84, 107.42, 23.83 ppm. MS (*m*/*z*): 177 [M + H]^+^.

In analogy to ZHAWOC3444 the following derivatives were synthesized, employing the alkyl bromide instead of the alkyl iodide:

*5-Amino-2-benzyl-2,3-dihydro-1H-isoindole-1,3-dione* (**11b**; ZHAWOC899): The title compound **11b** was obtained as a yellow solid in 59% yield: ^1^H-NMR DMSO-*d*_6_): δ = 7.51 (d, *J* = 8.04 Hz, 1H), 7.21–7.38 (m, 5H), 6.96 (d, *J* = 1.94 Hz, 1H), 6.82 (dd, *J* = 8.04 Hz, 1.94 Hz, 1H), 6.51 (s, 2H), 4.68 (s, 2H) ppm. ^13^C-NMR (DMSO-*d*_6_): δ = 168.06, 167.69, 155.07, 137.20, 134.41, 128.51, 127.25, 125.02, 116.67, 116.44, 107.09, 40.43 ppm. MS (*m*/*z*): 253 [M + H]^+^.

*5-Amino-2-[(4-fluorophenyl)methyl]-2,3-dihydro-1H-isoindole-1,3-dione* (**11c**; ZHAWOC3199): The title compound **11c** was obtained as a yellow solid in 68% yield: ^1^H-NMR DMSO-*d*_6_): δ = 7.49 (d, *J* = 8.14 Hz, 1H), 7.33–7.28 (m, 2H), 7.17–7.11 (m, 2H), 6.93 (d, *J* = 1.94 Hz, 1H), 6.80 (dd, *J* = 8.14 Hz, 1.94 Hz, 1H), 6.50 (s, 2H), 4.65 (s, 2H) ppm. ^13^C-NMR (DMSO-*d*_6_): δ = 168.50, 168.11, 161.82 (d, *J* = 243.45 Hz, 1C), 155.55, 134.88, 133.90 (d, *J* = 3.02 Hz, 1C), 129.95 (d, *J* = 8.25 Hz, 2C), 125.52, 117.14, 116.87, 115.76 (d, *J* = 21.43 Hz, 2C), 107.58, 40.22 ppm. MS (*m*/*z*): 271 [M + H]^+^.

*{[(7-Bromoheptyl)oxy]methyl}benzene* (**7a**; ZHAWOC7096): 1-bromoheptanol (5.00 g, 25.62 mmol) was dissolved in tetrahydrofuran (45 mL) under an argon atmosphere. Benzylbromide (4.5 mL, 38.43 mmol) and sodium hydride (2.05 g, 85.4 mmol) were added and the mixture was stirred for 3 days. The reaction was quenched with saturated sodium hydrogen carbonate and diluted with water (20 mL) and extracted with diethyl ether (3 × 30 mL). The combined organic extracts were dried over sodium sulphate and concentrated in vacuum. Purification by chromatography on silica gel (Gradient: 0–100% ethyl acetate in cyclohexane) afforded the title compound **7a** as a colorless oil (6.86 g, 94% yield): ^1^H-NMR (CDCl_3_): δ = 7.42–7.28 (m, 5H), 4.53 (s, 2H), 3.49 (t, *J* = 6.59 Hz, 2H), 3.43 (t, *J* = 6.82 Hz, 2H), 1.91–1.84 (m, 2H), 1.69–1.61 (m, 2H), 1.50–1.33 (m, 6H) ppm. ^13^C-NMR (CDCl_3_): δ = 138.67, 128.36, 127.63, 127.50, 72.90, 70.34, 33.98, 32.76, 29.67, 28.60, 28.12, 26.04 ppm. MS (*m*/*z*): 285 [M + H]^+^.

In analogy to ZHAWOC7096 the following derivatives were synthesized:

*{[(8-Bromooctyl)oxy]methyl}benzene* (**7b**; ZHAWOC6856): The title compound **7b** was obtained as a colorless oil in 91% yield: ^1^H-NMR (DMSO-*d*_6_): δ = 7.37–7.25 (m, 5H), 4.44 (s, 2H), 3.51 (t, *J* = 6.73 Hz, 2H), 3.41 (t, *J* = 6.52 Hz, 2H), 1.81–1.74 (m, 2H), 1.56-1.49 (m, 2H), 1.39–1.23 (m, 8H) ppm. ^13^C-NMR (DMSO-*d*_6_): δ = 139.20, 128.66, 127.84, 127.75, 72.25, 70.03, 35.67, 32.69, 29.61, 29.13, 28.54, 27.94, 26.08 ppm. MS (*m*/*z*): 299 [M + H]^+^.

*{[(9-Bromononyl)oxy]methyl}benzene* (**7c**; ZHAWOC6852): The title compound **7c** was obtained as a colorless oil in 90% yield: ^1^H-NMR (CDCl_3_): δ = 7.37–7.25 (m, 5H), 4.50 (s, 2H), 3.46 (t, *J* = 6.62 Hz, 2H), 3.40 (t, *J* = 6.89 Hz, 2H), 1.88–1.81 (m, 2H), 1.64–1.57 (m, 2H), 1.45–1.27 (m, 10H) ppm. ^13^C-NMR (CDCl_3_): δ = 138.73, 128.36, 127.64, 127.49, 72.89, 70.49, 34.04, 32.84, 29.77, 29.39, 29.37, 28.72, 28.17, 26.17 ppm. MS (*m*/*z*): 313 [M + H]^+^.

*{[(10-Bromodecyl)oxy]methyl}benzene* (**7d**; ZHAWOC6853): The title compound **7d** was obtained as a colorless oil in 76% yield: ^1^H-NMR (CDCl_3_): δ = 7.37–7.24 (m, 5H), 4.50 (s, 2H), 3.46 (t, *J* = 6.62 Hz, 2H), 3.39 (t, *J* = 6.82 Hz, 2H), 1.87–1.81 (m, 2H), 1.64–1.57 (m, 2H), 1.44–1.26 (m, 12H) ppm. ^13^C-NMR (CDCl_3_): δ = 138.75, 128.36, 127.63, 127.48, 72.89, 70.52, 34.06, 32.86, 29.79, 29.49, 29.45, 29.39, 28.77, 28.19, 26.20 ppm. MS (*m*/*z*): 327 [M + H]^+^.

*Methyl 2-{4-[2-(benzyloxy)ethoxy]phenyl}acetate* (**8a**; ZHAWOC7100): Under an argon atmosphere, methyl 2-(4-hydroxyphenyl)acetate (3.51 g, 21.14 mmol) and caesium carbonate (13.78 g, 42.28 mmol) were suspended in dimethylformamide (130 mL), the mixture was stirred at ambient temperature for 2 h. Benzyl-2-bromoethylether (5.00 g, 23.25 mmol) was added and it was stirred at ambient temperature for further 12 h. Water (250 mL) and ethyl acetate (250 mL) were added and the resulting phases separated. The organic phase was dried over sodium sulfate and concentrated in vacuum. Purification by chromatography on silica gel (gradient: 0–100% ethyl acetate in cyclohexane) afforded the title compound **8a** as a white solid (5.30 g, 84% yield): ^1^H-NMR (DMSO-*d*_6_): δ = 7.38–7.26 (m, 5H), 7.19–7.15 (m, 2H), 6.92–6.88 (m, 2H), 4.55 (s, 2H), 4.13–4.10 (m, 2H), 3.78–3.74 (m, 2H), 3.60 (s, 3H), 3.59 (s, 2H) ppm. ^13^C-NMR (DMSO-*d*_6_): δ = 172.33, 157.88, 138.78, 130.83, 128.70, 127.99, 127.89, 126.83, 114.83, 72.55, 68.71, 67.53, 52.06, 39.72 ppm. MS (*m*/*z*): 301 [M + H]^+^.

In analogy to ZHAWOC7100 the following derivatives were synthesized:

*Methyl 2-{4-[3-(benzyloxy)propoxy]phenyl}acetate* (**8b**; ZHAWOC4496): The title compound **8b** was obtained as a white solid (1.46 g, 77% yield): ^1^H-NMR (CDCl_3_): δ = 7.35–7.20 (m, 5H), 7.19–7.13 (m, 2H), 6.87–6.79 (m, 2H), 4.49 (s, 2H), 4.04 (t, *J* = 6.24 Hz, 2H), 3.65 (s, 3H), 3.63 (t, *J* = 6.15 Hz, 2H), 3.53 (s, 2H), 2.05 (quint., *J* = 6.20 Hz, 2H) ppm. ^13^C-NMR (CDCl_3_): δ = 172.39, 158.21, 138.49, 130.30, 128.43, 127.65, 127.61, 1276.04, 114.69, 73.07, 66.86, 64.89, 51.99, 40.33, 29.80 ppm. MS (*m*/*z*): 337 [M + Na]^+^.

*Methyl 2-{4-[4-(benzyloxy)butoxy]phenyl}acetate* (**8c**; ZHAWOC4534): The title compound **8c** was obtained as a white solid in 68% yield: ^1^H-NMR (CDCl_3_): δ = 7.37–7.22 (m, 5H), 7.20–7.12 (m, 2H), 6.86–6.79 (m, 2H), 4.50 (s, 2H), 3.95 (t, *J* = 6.08 Hz, 2H), 3.66 (s, 3H), 3.54 (s, 2H), 3.53 (t, *J* = 6.08 Hz, 2H), 1.93–1.72 (m, 4H) ppm. ^13^C-NMR (CDCl_3_): δ = 172.40, 158.22, 138.59, 130.26, 128.40, 127.66, 127.57, 125.93, 114.63, 72.94, 69.95, 67.67, 51.99, 40.32, 26.40, 26.18 ppm. MS (*m*/*z*): 351 [M + Na]^+^.

*Methyl 2-(4-{[5-(benzyloxy)pentyl]oxy}phenyl)acetate* (**8d**; ZHAWOC5921): The title compound **8d** was obtained as a white solid in 90% yield: ^1^H-NMR (CDCl_3_): δ = 7.43–7.30 (m, 5H), 7.26–7.21 (m, 2H), 6.93–6.88 (m, 2H), 4.56 (s, 2H), 3.99 (t, *J* = 6.46 Hz, 2H), 3.72 (s, 3H), 3.61 (s, 2H), 3.56 (t, *J* = 6.46 Hz, 2H), 1.89–1.81 (m, 2H), 1.79–1.72 (m, 2H), 1.66–1.58 (m, 2H) ppm. ^13^C-NMR (CDCl_3_): δ = 172.22, 158.18, 138.59, 130.16, 128.28, 127.53, 127.42, 125.82, 114.51, 72.84, 70.15, 67.71, 51.82, 40.20, 29.48, 29.05, 22.77 ppm. MS (*m*/*z*): 343 [M + H]^+^.

*Methyl 2-(4-{[6-(benzyloxy)hexyl]oxy}phenyl)acetate* (**8e**; ZHAWOC5946): The title compound **8e** was obtained as a white solid in 74% yield: ^1^H-NMR (CDCl_3_): δ = 7.39–7.34 (m, 4H), 7.32–7.27 (m, 1H), 7.22–7.18 (m, 2H), 6.88–6.84 (m, 2H), 4.53 (s, 2H), 3.95 (t, *J* = 6.51 Hz, 2H), 3.70 (s, 3H), 3.58 (s, 2H), 3.50 (t, *J* = 6.51 Hz, 2H), 1.84–1.76 (m, 2H), 1.71–1.64 (m, 2H), 1.53–1.43 (m, 4H) ppm. ^13^C-NMR (CDCl_3_): δ = 172.42, 158.31, 138.73, 130.29, 128.41, 127.68, 127.55, 125.91, 114.65, 72.94, 70.37, 67.92, 52.02, 40.37, 29.78, 29.30, 26.07, 25.99 ppm. MS (*m*/*z*): 357 [M + H]^+^.

*Methyl 2-(4-{[7-(benzyloxy)heptyl]oxy}phenyl)acetate* (**8f**; ZHAWOC7097): The title compound **8f** was obtained as a white solid in 78% yield: ^1^H-NMR (CDCl_3_): δ = 7.37–7.29 (m, 4H), 7.29–7.22 (m, 1H), 7.18–7.13 (m, 2H), 6.86–6.79 (m, 2H), 4.49 (s, 2H), 3.91 (t, *J* = 6.63 Hz, 2H), 3.66 (s, 3H), 3.54 (s, 2H), 3.46 (t, *J* = 6.63 Hz, 2H), 1.79–1.71 (m, 2H), 1.66–1.59 (m, 2H), 1.49–1.31 (m, 6H) ppm. ^13^C-NMR (CDCl_3_): δ = 172.38, 158.30, 138.74, 130.7425, 128.37, 127.63, 127.50, 125.86, 114.62, 72.89, 70.44, 67.94, 51.99, 40.34, 29.75, 29.25, 26.19, 26.05 ppm. MS (*m*/*z*): 371 [M + H]^+^.

*Methyl 2-(4-{[8-(benzyloxy)octyl]oxy}phenyl)acetate* (**8g**; ZHAWOC6857): The title compound **8g** was obtained as a white solid in 69% yield: ^1^H-NMR (DMSO-*d*_6_): δ = 7.36–7.24 (m, 5H), 7.18–7.13 (m, 2H), 6.88–6.83 (m, 2H), 4.43 (s, 2H), 3.92 (t, *J* = 6.31 Hz, 2H), 3.59 (s, 3H), 3.58 (s, 2H), 3.41 (t, *J* = 6.31 Hz, 2H), 1.71–1.64 (m, 2H), 1.56–1.50 (m, 2H), 1.42–1.24 (m, 8H) ppm. ^13^C-NMR (DMSO-*d*_6_): δ = 172.36, 158.06, 139.21, 130.79, 128.66, 127.84, 127.75, 126.54, 114.74, 72.25, 70.05, 67.81, 52.07, 39.71, 29.64, 29.25, 29.20, 29.13, 26.12, 25.94 ppm. MS (*m*/*z*): 385 [M + H]^+^.

*Methyl 2-(4-{[9-(benzyloxy)nonyl]oxy}phenyl)acetate* (**8h**; ZHAWOC6854): The title compound **8h** was obtained as a white solid in 55% yield: ^1^H-NMR (CDCl_3_): δ = 7.36–7.31 (m, 4H), 7.29–24 (m, 1H), 7.20–7.14 (m, 2H), 6.86–6.82 (m, 2H), 4.50 (s, 2H), 3.92 (t, *J* = 6.83 Hz, 2H), 3.67 (s, 3H), 3.55 (s, 2H), 3.46 (t, *J* = 6.74 Hz, 2H), 1.79–1.72 (m, 2H), 1.64–1.58 (m, 2H), 1.46–1.27 (m, 10H) ppm. ^13^C-NMR (CDCl_3_): δ = 172.40, 158.30, 138.74, 130.23, 128.35, 127.62, 127.47, 125.82, 114.61, 72.88, 70.51, 68.00, 51.99, 40.33, 29.79, 29.53, 29.43, 29.35, 29.29, 26.21, 26.05 ppm. MS (*m*/*z*): 399 [M + H]^+^.

*Methyl 2-(4-{[10-(benzyloxy)decyl]oxy}phenyl)acetate* (**8i**; ZHAWOC6855): The title compound **8i** was obtained as a white solid in 49% yield: ^1^H-NMR (CDCl_3_): δ = 7.35–7.31 (m, 4H), 7.29–24 (m, 1H), 7.19–7.14 (m, 2H), 6.86–6.82 (m, 2H), 4.49 (s, 2H), 3.92 (t, *J* = 6.65 Hz, 2H), 3.67 (s, 3H), 3.54 (s, 2H), 3.46 (t, *J* = 6.65 Hz, 2H), 1.79–1.72 (m, 2H), 1.64–1.58 (m, 2H), 1.46–1.27 (m, 12H) ppm. ^13^C-NMR (CDCl_3_): δ = 172.40, 158.33, 138.78, 130.25, 128.36, 127.63, 127.48, 125.84, 114.62, 72.89, 70.54, 68.01, 51.98, 40.33, 29.82, 29.56, 29.55, 29.50, 29.41, 29.31, 26.23, 26.08 ppm. MS (*m*/*z*): 413 [M + H]^+^.

*2-{4-[2-(Benzyloxy)ethoxy]phenyl}acetic acid* (**9a**; ZHAWOC7101): The ester (**8a**) (5.20 g, 11.33 mmol) was dissolved in methanol (300 mL) and stirred at ambient temperature. Potassium hydroxide 10% in water (300 mL) was added over 10 min. and the mixture was stirred for another 20 min. Methanol was removed in vacuum and the aqueous phase extracted with diethyl ether (200 mL). The aqueous phase was acidified with concentrated hydrochloric acid and extracted with diethyl ether (300 mL). The second organic phase was dried over sodium sulfate and concentrated in vacuum to obtain the title compound **9a** as a white solid (4.71 g, 95% yield): ^1^H–NMR (DMSO-*d*_6_): δ = 12.25 (s, 1H), 7.38–7.33 (m, 4H), 7.31–7.26 (m, 1H), 7.20–7.14 (m, 2H), 6.92–6.86 (m, 2H), 4.55 (s, 2H), 4.13–4.09 (m, 2H), 3.78–3.74 (m, 2H), 3.49 (s, 2H) ppm. ^13^C-NMR (DMSO-*d*_6_): δ = 173.44, 157.72, 138.78, 130.84, 128.71, 128.00, 127.89, 127.53, 114.73, 72.56, 68.72, 67.53, 40.26 ppm. MS (*m*/*z*): 285 [M − H]^−^.

In analogy to ZHAWOC7101 the following derivatives were synthesized:

*2-{4-[3-(Benzyloxy)propoxy]phenyl}acetic acid* (**9b**; ZHAWOC4497): The title compound **9b** was obtained as a white solid (0.48 g, 98% yield): ^1^H-NMR (DMSO-*d*_6_): δ = 12.24 (br. s, 1H), 7.36–7.23 (m, 5H), 7.20–7.11 (m, 2H), 6.90–6.80 (m, 2H), 4.48 (s, 2H), 4.02 (t, *J* = 6.33 Hz, 2H), 3.59 (t, *J* = 6.33 Hz, 2H), 3.49 (s, 2H), 1.99 (quint., *J* = 6.33 Hz, 4H) ppm. ^13^C-NMR (DMSO-*d*_6_: δ = 173.43, 157.83, 139.00, 130.79, 128.66, 127.83, 127.77, 127.38, 114.67, 72.40, 66.76, 64.99, 40.29, 29.64 ppm. MS (*m*/*z*): 299 [M − H]^−^.

*2-{4-[4-(Benzyloxy)butoxy]phenyl}acetic acid* (**9c**; ZHAWOC4535): The title compound **9c** was obtained as a white solid in 90% yield: ^1^H-NMR (CDCl_3_): δ = 8.90 (br. s, 1H), 7.36–7.23 (m, 5H), 7.19–7.11 (m, 2H), 6.86–6.78 (m, 2H), 4.51 (s, 2H), 3.94 (t, *J* = 6.03 Hz, 2H), 3.55 (s, 2H), 3.53 (t, *J* = 6.03 Hz, 2H), 1.92–1.71 (m, 4H) ppm. ^13^C-NMR (CDCl_3_): δ = 177.93, 158.34, 138.47, 130.41, 128.42, 127.73, 127.62, 125.32, 114.68, 72.93, 69.93, 67.67, 40.20, 26.35, 26.14 ppm. MS (*m*/*z*): 313 [M − H]^−^.

*2-(4-{[5-(Benzyloxy)pentyl]oxy}phenyl)acetic acid* (**9d**; ZHAWOC5922): The title compound **9d** was obtained as a white solid in 91% yield: ^1^H-NMR (CDCl_3_): δ = 7.36–7.26 (m, 5H), 7.20–7.16 (m, 2H), 6.88–6.83 (m, 2H), 4.53 (s, 2H), 3.94 (t, *J* = 6.48 Hz, 2H), 3.55 (s, 2H), 3.57 (t, *J* = 6.48 Hz, 2H), 1.83–1.76 (m, 2H), 1.74–1.67 (m, 2H), 1.59–1.51 (m, 2H) ppm. ^13^C-NMR (CDCl_3_): δ = 177.20, 158.31, 138.48, 130.39, 128.40, 127.71, 127.59, 125.48, 114.64, 72.90, 70.21, 67.83, 40.21, 29.47, 29.09, 22.79 ppm. MS (*m*/*z*): 327 [M − H]^−^.

*2-(4-{[6-(Benzyloxy)hexyl]oxy}phenyl)acetic acid* (**9e**; ZHAWOC5947): The title compound **9e** was obtained as a white solid in 73% yield: ^1^H-NMR (DMSO-*d*_6_): δ = 12.23 (br. s, 1H), 7.36–7.23 (m, 5H), 7.18–7.12 (m, 2H), 6.87–6.81 (m, 2H), 4.44 (s, 2H), 3.91 (t, *J* = 6.50 Hz, 2H), 3.47 (s, 2H), 3.42 (t, *J* = 6.50 Hz, 2H), 1.73–1.65 (m, 2H), 1.59–1.52 (m, 2H), 1.45–1.33 (m, 4H) ppm. ^13^C-NMR (DMSO-*d*_6_): δ = 173.45, 157.90, 139.20, 130.79, 128.66, 127.84, 127.75, 127.25, 114.64, 72.27, 70.01, 67.77, 40.26, 29.63, 29.14, 25.96, 25.85 ppm. MS (*m*/*z*): 341 [M − H]^−^.

*2-(4-{[7-(Benzyloxy)heptyl]oxy}phenyl)acetic acid* (**9f**; ZHAWOC7098): The title compound **9f** was obtained as a white solid in 94% yield: ^1^H-NMR (CDCl_3_): δ = 7.35–7.32 (m, 4H), 7.30–7.25 (m, 1H), 7.20–7.16 (m, 2H), 6.87–6.83 (m, 2H), 4.50 (s, 2H), 3.92 (t, *J* = 6.57 Hz, 2H), 3.58 (s, 2H), 3.47 (t, *J* = 6.66 Hz, 2H), 1.80–1.73 (m, 2H), 1.66–1.59 (m, 2H), 1.48–1.34 (m, 6H) ppm. ^13^C-NMR (CDCl_3_): δ = 158.43, 138.67, 130.34, 128.35, 127.63, 127.48, 125.12, 114.67, 72.87, 70.42, 67.95, 39.85, 29.20, 26.14, 26.00 ppm. MS (*m*/*z*): 355 [M − H]^−^.

*2-(4-{[8-(Benzyloxy)octyl]oxy}phenyl)acetic acid* (**9g**; ZHAWOC6858): The title compound **9g** was obtained as a white solid in 96% yield: ^1^H–NMR (DMSO-*d*_6_): δ = 12.23 (br. s, 1H), 7.36–7.24 (m, 5H), 7.16–7.11 (m, 2H), 6.87–6.82 (m, 2H), 4.44 (s, 2H), 3.91 (t, *J* = 6.50 Hz, 2H), 3.47 (s, 2H), 3.41 (t, *J* = 6.55 Hz, 2H), 1.72–1.64 (m, 2H), 1.57–1.50 (m, 2H), 1.42–1.23 (m, 8H) ppm. ^13^C-NMR (DMSO-*d*_6_,): δ = 173.45, 157.89, 139.21, 130.78, 128.66, 127.84, 127.75, 127.27, 114.63, 72.24, 70.05, 67.81, 40.27, 29.64, 29.25, 29.20, 29.14, 26.12, 25.95 ppm. MS (*m*/*z*): 369 [M − H]^−^.

*2-(4-{[9-(Benzyloxy)nonyl]oxy}phenyl)acetic acid* (**9h**; ZHAWOC6861): The title compound **9h** was obtained as a white solid in 81% yield: ^1^H–NMR (DMSO-*d*_6_): δ = 12.24 (br. s, 1H), 7.36–7.24 (m, 5H), 7.16–7.11 (m, 2H), 6.87–6.82 (m, 2H), 4.43 (s, 2H), 3.91 (t, *J* = 6.56 Hz, 2H), 3.46 (s, 2H), 3.40 (t, *J* = 6.56 Hz, 2H), 1.72–1.64 (m, 2H), 1.57–1.49 (m, 2H), 1.42–1.23 (m, 10H) ppm. ^13^C-NMR (DMSO-*d*_6_): δ = 173.45, 157.89, 139.21, 130.78, 128.66, 127.83, 127.74, 127.27, 114.63, 72.24, 70.05, 67.81, 40.27, 29.65, 29.43, 29.25, 29.20, 29.16, 26.15, 25.98 ppm. MS (*m*/*z*): 383 [M − H]^−^.

*2-(4-{[10-(Benzyloxy)decyl]oxy}phenyl)acetic acid* (**9i**; ZHAWOC6862): The title compound **9i** was obtained as a white solid in 99% yield: ^1^H-NMR (DMSO-*d*_6_): δ = 12.24 (br. s, 1H), 7.36–7.24 (m, 5H), 7.16–7.11 (m, 2H), 6.87–6.82 (m, 2H), 4.43 (s, 2H), 3.91 (t, *J* = 6.38 Hz, 2H), 3.46 (s, 2H), 3.40 (t, *J* = 6.38 Hz, 2H), 1.72–1.64 (m, 2H), 1.57–1.49 (m, 2H), 1.42–1.23 (m, 12H) ppm. ^13^C-NMR (DMSO-*d*_6_): δ = 173.45, 157.89, 139.21, 130.79, 128.66, 127.83, 127.74, 127.27, 114.63, 72.24, 70.06, 67.81, 40.27, 29.65, 29.42, 29.28, 29.22, 29.16, 26.16, 25.99 ppm. MS (*m*/*z*): 397 [M − H]^−^.

*2-(4-{[5-(Benzyloxy)pentyl]oxy}phenyl)-N-(2-methyl-1,3-dioxo-2,3-dihydro-1H-isoindol-5-yl)acetamide* (**12a**; ZHAWOC6647): The acid **9d** (0.92 g, 2.80 mmol) was stirred in an excess of thionyl chloride at 55 °C for 1 h. After removal of excess thionyl chloride under vacuum, the acid chloride was dissolved in tetrahydrofuran (2 mL) and added to a solution of 5-amino-2-methylisoindoline-1,3-dione (**11a**, 0.41 g, 2.33 mmol) in tetrahydrofuran (12 mL) under argon at ambient temperature. Diisopropylethylamine (1.00 mL) was added and the mixture was stirred at ambient temperature for 2 h. After removal of the tetrahydrofuran in vacuum, ethyl acetate (70 mL) and 10% citric acid (70 mL) were added and the resulting phases were separated. The organic phase was washed with 10% sodium bicarbonate (70 mL) and brine (70 mL), dried over sodium sulfate and concentrated in vacuum. Purification by chromatography on silica gel (Gradient: 0–100% ethyl acetate in cyclohexane) afforded the title compound **12a** as a yellow solid (0.12 g, 11% yield): ^1^H-NMR (DMSO-*d*_6_): δ = 10.69 (s, 1H), 8.18 (d, *J* = 1.83 Hz, 1H), 7.85 (dd, *J* = 8.19 Hz, 1.83 Hz, 1H), 7.79 (d, *J* = 8.19 Hz, 1H), 7.35–7.21 (m, 7H), 6.89–6.85 (m, 2H), 4.44 (s. 2H), 3.93 (t, *J* = 6.52 Hz, 2H), 3.63 (s, 2H), 3.43 (t, *J* = 6.47 Hz, 2H), 3.00 (s, 3H), 1.74–1.66 (m, 2H), 1.63–1.56 (m, 2H), 1.50–1.42 (m, 2H) ppm. ^13^C-NMR (DMSO-*d*_6_): δ = 170.90, 168.26, 168.12, 158.03, 145.04, 139.18, 133.82, 130.64, 128.68, 127.86, 127.77, 127.55, 126.09, 124.55, 123.52, 114.82, 113.09, 72.28, 69.98, 67.80, 42.97, 29.39, 28.96, 24.19, 22.85 ppm. MS (*m*/*z*): 487 [M + H]^+^.

In analogy to ZHAWOC6647 the following derivatives were synthesized:

*N-(2-Benzyl-1,3-dioxo-2,3-dihydro-1H-isoindol-5-yl)-2-{4-[2-(benzyloxy)ethoxy]phenyl}acetamide* (**12b**; ZHAWOC5467): The title compound **12b** was obtained as a yellow solid in 80% yield: ^1^H-NMR (DMSO-*d*_6_): δ = 10.74 (s, 1H), 8.21 (d, *J* = 1.85 Hz, 1H), 7.89 (dd, *J* = 8.21 Hz, 1.85 Hz, 1H), 7.84 (d, *J* = 8.21 Hz, 1H), 7.36–7.23 (m, 12H), 6.93–6.89 (m, 2H), 4.73 (s, 2H), 4.55 (s, 2H), 4.13–4.10 (m, 2H), 3.77–3.74 (m, 2H), 3.64 (s, 2H) ppm. ^13^C-NMR (DMSO-*d*_6_): δ = 170.90, 167.94, 167.76, 157.84, 145.30, 138.77, 137.21, 130.67, 129.04, 128.70, 127.99, 127.88, 127.86, 127.83, 127.76, 124.93, 114.89, 113.33, 72.53, 68.70, 67.54, 42.95, 41.31 ppm. MS (*m*/*z*): 521 [M + H]^+^.

*N-(2-Benzyl-1,3-dioxo-2,3-dihydro-1H-isoindol-5-yl)-2-{4-[3-(benzyloxy)propoxy]phenyl}acetamide* (**12c**; ZHAWOC4511): The title compound **12c** was obtained as a yellow solid (2.50g, 62% yield): ^1^H-NMR (DMSO-*d*_6_): δ = 10.73 (s, 1H), 8.21 (d, *J* = 1.80 Hz, 1H), 7.89 (dd, *J* = 8.31 Hz, 1.80 Hz, 1H), 7.83 (d, *J* = 8.31 Hz, 1H), 7.35–7.21 (m, 12H), 6.91–6.85 (m, 2H), 4.73 (s, 2H), 4.47 (s, 2H), 4.02 (t, *J* = 6.35 Hz, 2H), 3.64 (s, 2H), 3.57 (t, *J* = 6.35 Hz, 2H), 1.97 (quint., *J* = 3.65 Hz, 2H) ppm. ^13^C-NMR (DMSO-*d*_6_): δ = 170.92, 167.93, 167.76, 157.93, 145.30, 139.00, 137.21, 133.54, 130.66, 129.04, 128.67, 127.84, 127.83, 127.79, 127.63, 124.92, 123.81, 114.83, 113.33, 72.35, 66.72, 65.02, 42.96, 41.31, 29.61 ppm. MS (*m*/*z*): 535 [M + H]^+^.

*N-(2-Benzyl-1,3-dioxo-2,3-dihydro-1H-isoindol-5-yl)-2-{4-[4-(benzyloxy)butoxy]phenyl}acetamide* (**12d**; ZHAWOC4752): The title compound **12d** was obtained as a yellow solid in 64% yield: ^1^H-NMR (DMSO-*d*_6_): δ = 10.73 (s, 1H), 8.21 (d, *J* = 1.83 Hz, 1H), 7.89 (dd, *J* = 8.23 Hz, 1.83 Hz, 1H), 7.84 (d, *J* = 8.23 Hz, 1H), 7.35–7.21 (m, 12H), 6.89–6.85 (m, 2H), 4.73 (s, 2H), 4.45 (s. 2H), 3.95 (t, *J* = 6.27 Hz, 2H), 3.63 (s, 2H), 3.48 (t, *J* = 6.27 Hz, 2H), 1.79–1.72 (m, 2H), 1.72–1.64 (m, 2H) ppm. ^13^C-NMR (DMSO-*d*_6_): δ = 170.95, 167.96, 167.78, 157.99, 145.32, 139.14, 137.23, 133.55, 130.65, 129.06, 128.69, 127.89, 127.87, 127.84, 127.78, 127.56, 125.73, 124.94, 123.83, 114.84, 113.34, 72.28, 69.75, 67.66, 42.97, 41.32, 26.29, 26.10 ppm. MS (*m*/*z*): 549 [M + H]^+^.

*N-(2-Benzyl-1,3-dioxo-2,3-dihydro-1H-isoindol-5-yl)-2-(4-{[5-(benzyloxy)pentyl]oxy}phenyl)acetamide* (**12e**; ZHAWOC5979): The title compound **12e** was obtained as a white solid in 44% yield: ^1^H-NMR (DMSO-*d*_6_): δ = 10.73 (s, 1H), 8.21 (d, *J* = 1.83 Hz, 1H), 7.89 (dd, *J* = 8.23 Hz, 1.83 Hz, 1H), 7.84 (d, *J* = 8.23 Hz, 1H), 7.35–7.21 (m, 12H), 6.89–6.85 (m, 2H), 4.73 (s, 2H), 4.44 (s. 2H), 3.93 (t, *J* = 6.47 Hz, 2H), 3.63 (s, 2H), 3.43 (t, *J* = 6.39 Hz, 2H), 1.74–1.66 (m, 2H), 1.63–1.56 (m, 2H), 1.50–1.42 (m, 2H) ppm. ^13^C-NMR (DMSO-*d*_6_): δ = 170.93, 167.94, 167.76, 158.01, 145.30, 139.17, 137.21, 133.54, 130.63, 129.05, 128.67, 127.85, 127.83, 127.76, 127.51, 125.72, 124.93, 123.81, 114.82, 113.33, 72.27, 69.97, 67.79, 42.96, 41.31, 29.38, 28.95, 22.84 ppm. MS (*m*/*z*): 563 [M + H]^+^.

*N-(2-Benzyl-1,3-dioxo-2,3-dihydro-1H-isoindol-5-yl)-2-(4-{[6-(benzyloxy)hexyl]oxy}phenyl)acetamide* (**12f**; ZHAWOC5980): The title compound **12f** was obtained as a white solid in 34% yield: ^1^H-NMR (DMSO-*d*_6_): δ = 10.73 (s, 1H), 8.21 (d, *J* = 1.83 Hz, 1H), 7.89 (dd, *J* = 8.23 Hz, 1.83 Hz, 1H), 7.83 (d, *J* = 8.23 Hz, 1H), 7.34–7.21 (m, 12H), 6.89–6.85 (m, 2H), 4.73 (s, 2H), 4.43 (s. 2H), 3.91 (t, *J* = 6.43 Hz, 2H), 3.63 (s, 2H), 3.41 (t, *J* = 6.43 Hz, 2H), 1.72–1.64 (m, 2H), 1.58–1.51 (m, 2H), 1.44–1.32 (m, 4H) ppm. ^13^C-NMR (DMSO-*d*_6_): δ = 170.93, 167.93, 167.75, 158.03, 145.31, 139.18, 137.21, 133.53, 130.63, 129.04, 128.65, 127.85, 127.84, 127.74, 127.50, 125.71, 124.91, 123.80, 114.80, 113.33, 72.25, 69.99, 67.79, 42.97, 41.31, 29.62, 29.11, 25.95, 25.83 ppm. MS (*m*/*z*): 577 [M + H]^+^.

*N-(2-Benzyl-1,3-dioxo-2,3-dihydro-1H-isoindol-5-yl)-2-(4-{[7-(benzyloxy)heptyl]oxy}phenyl)acetamide* (**12g**; ZHAWOC7099): The title compound **12g** was obtained as a white solid in 18% yield: ^1^H-NMR (DMSO-*d*_6_): δ = 10.72 (s, 1H), 8.21 (d, *J* = 1.80 Hz, 1H), 7.89 (dd, *J* = 8.24 Hz, 1.80 Hz, 1H), 7.84 (d, *J* = 8.24 Hz, 1H), 7.36–7.21 (m, 12H), 6.89–6.85 (m, 2H), 4.73 (s, 2H), 4.43 (s. 2H), 3.92 (t, *J* = 6.41 Hz, 2H), 3.63 (s, 2H), 3.41 (t, *J* = 6.41 Hz, 2H), 1.72–1.64 (m, 2H), 1.58–1.50 (m, 2H), 1.43–1.26 (m, 6H) ppm. ^13^C-NMR (DMSO-*d*_6_): δ = 170.93, 167.94, 167.76, 158.03, 145.31, 139.20, 137.21, 133.54, 130.63, 129.04, 128.66, 127.86, 127.83, 127.74, 127.50, 125.72, 124.93, 123.81, 114.81, 113.32, 72.24, 70.02, 67.80, 42.96, 41.31, 29.59, 29.09, 29.01, 26.12, 25.95 ppm. MS (*m*/*z*): 591 [M + H]^+^.

*N-(2-Benzyl-1,3-dioxo-2,3-dihydro-1H-isoindol-5-yl)-2-(4-{[8-(benzyloxy)octyl]oxy}phenyl)acetamide* (**12h**; ZHAWOC7095): The title compound **12h** was obtained as a white solid in 38% yield: ^1^H-NMR (DMSO-*d*_6_): δ = 10.73 (s, 1H), 8.21 (d, *J* = 1.80 Hz, 1H), 7.89 (dd, *J* = 8.24 Hz, 1.80 Hz, 1H), 7.84 (d, *J* = 8.24 Hz, 1H), 7.35–7.21 (m, 12H), 6.89–6.85 (m, 2H), 4.74 (s, 2H), 4.43 (s. 2H), 3.92 (t, *J* = 6.47 Hz, 2H), 3.63 (s, 2H), 3.40 (t, *J* = 6.47 Hz, 2H), 1.72–1.64 (m, 2H), 1.56–1.49 (m, 2H), 1.42–1.24 (m, 8H) ppm. ^13^C-NMR (DMSO-*d*_6_): δ = 170.93, 167.94, 167.76, 158.03, 145.31, 139.20, 137.21, 133.54, 130.63, 129.04, 128.65, 127.86, 127.83, 127.74, 127.49, 125.71, 124.92, 123.81, 114.80, 113.32, 72.24, 70.04, 67.82, 42.96, 41.31, 29.63, 29.24, 29.19, 29.12, 26.11, 25.94 ppm. MS (*m*/*z*): 605 [M + H]^+^.

*N-(2-Benzyl-1,3-dioxo-2,3-dihydro-1H-isoindol-5-yl)-2-(4-{[9-(benzyloxy)nonyl]oxy}phenyl)acetamide* (**12i**; ZHAWOC6931): The title compound **12i** was obtained as a white solid in 63% yield: ^1^H-NMR (DMSO-*d*_6_): δ = 10.73 (s, 1H), 8.21 (d, *J* = 1.80 Hz, 1H), 7.89 (dd, *J* = 8.25 Hz, 1.80 Hz, 1H), 7.83 (d, *J* = 8.25 Hz, 1H), 7.35–7.21 (m, 12H), 6.89–6.85 (m, 2H), 4.74 (s, 2H), 4.42 (s. 2H), 3.92 (t, *J* = 6.64 Hz, 2H), 3.63 (s, 2H), 3.40 (t, *J* = 6.64 Hz, 2H), 1.72–1.64 (m, 2H), 1.56–1.48 (m, 2H), 1.42–1.21 (m, 10H) ppm. ^13^C-NMR (DMSO-*d*_6_): δ = 170.93, 167.93, 167.76, 158.03, 145.31, 139.20, 137.21, 133.54, 130.63, 129.04, 128.65, 127.86, 127.83, 127.74, 127.49, 125.72, 124.92, 123.81, 114.80, 113.33, 72.24, 70.05, 67.83, 42.96, 41.31, 29.64, 29.42, 29.23, 29.17, 29.14, 26.14, 25.96 ppm. MS (*m*/*z*): 619 [M + H]^+^.

*N-(2-Benzyl-1,3-dioxo-2,3-dihydro-1H-isoindol-5-yl)-2-(4-{[10-(benzyloxy)decyl]oxy}phenyl)acetamide* (**12j**; ZHAWOC6932): The title compound **12j** was obtained as a white solid in 46% yield: ^1^H-NMR (DMSO-*d*_6_): δ = 10.74 (s, 1H), 8.21 (d, *J* = 1.85 Hz, 1H), 7.89 (dd, *J* = 8.23 Hz, 1.85 Hz, 1H), 7.84 (d, *J* = 8.23 Hz, 1H), 7.35–7.21 (m, 12H), 6.89–6.85 (m, 2H), 4.73 (s, 2H), 4.42 (s. 2H), 3.92 (t, *J* = 6.65 Hz, 2H), 3.63 (s, 2H), 3.40 (t, *J* = 6.65 Hz, 2H), 1.72–1.64 (m, 2H), 1.55–1.48 (m, 2H), 1.42–1.21 (m, 12H) ppm. ^13^C-NMR (DMSO-*d*_6_): δ = 170.93, 167.94, 167.76, 158.03, 145.31, 139.20, 137.21, 133.54, 130.63, 129.04, 128.65, 127.86, 127.83, 127.74, 127.49, 125.71, 124.92, 123.81, 114.80, 113.32, 72.23, 70.04, 67.83, 42.96, 41.31, 29.64, 29.40, 29.26, 29.19, 29.14, 26.15, 25.97 ppm. MS (*m*/*z*): 633 [M + H]^+^.

*2-{4-[3-(Benzyloxy)propoxy]phenyl}-N-{2-[(4-fluorophenyl)methyl]-1,3-dioxo-2,3-dihydro-1H-isoindol-5-yl}acetamide* (**12k**; ZHAWOC6641): The title compound **12k** was obtained as a yellow solid in 72% yield: ^1^H-NMR (DMSO-*d*_6_): δ = 10.73 (s, 1H), 8.21 (d, *J* = 1.80 Hz, 1H), 7.89 (dd, *J* = 8.27 Hz, 1.80 Hz, 1H), 7.83 (d, *J* = 8.27 Hz, 1H), 7.36–7.28 (m, 6H), 7.26–7.22 (m, 3H), 7.17–7.11 (m, 2H), 6.90–6.85 (m, 2H), 4.71 (s, 2H), 4.47 (s, 2H), 4.02 (t, *J* = 6.50 Hz, 2H), 3.64 (s, 2H), 3.57 (t, *J* = 6.23 Hz, 2H), 2.00–1.94 (m, 2H) ppm. ^13^C-NMR (DMSO-*d*_6_): δ = 170.94, 167.91, 167.73, 161.92 (d, *J* = 243.23 Hz, 1C), 157.95, 145.31, 139.01, 133.55, 133.45 (d, *J* = 3.05 Hz, 1C), 130.67, 130.09 (d, *J* = 7.75 Hz, 2C), 128.69, 127.86, 127.80, 127.64, 125.72, 124.93, 123.82, 115.82 (d, *J* = 21.40 Hz, 2C), 114.85, 113.34, 72.36, 66.73, 65.02, 42.96, 40.63, 29.61 ppm. MS (*m*/*z*): 553 [M + H]^+^.

*2-{4-[4-(Benzyloxy)butoxy]phenyl}-N-{2-[(4-fluorophenyl)methyl]-1,3-dioxo-2,3-dihydro-1H-isoindol-5-yl}acetamide* (**12l**; ZHAWOC7102): The title compound 12l was obtained as a yellow solid in 55% yield: 1H-NMR (DMSO-d6): δ = 10.73 (s, 1H), 8.21 (d, *J* = 1.89 Hz, 1H), 7.89 (dd, *J* = 8.21 Hz, 1.89 Hz, 1H), 7.83 (d, *J* = 8.21 Hz, 1H), 7.36–7.28 (m, 7H), 7.26–7.21 (m, 2H), 7.17–7.11 (m, 2H), 6.90–6.85 (m, 2H), 4.72 (s, 2H), 4.45 (s, 2H), 3.95 (t, *J* = 6.10 Hz, 2H), 3.63 (s, 2H), 3.48 (t, *J* = 6.20 Hz, 2H), 1.80–1.73 (m, 2H), 1.71–1.64 (m, 2H) ppm. ^13^C-NMR (DMSO-*d*_6_): δ = 170.94, 167.92, 167.74, 161.92 (d, *J* = 243.56 Hz, 1C), 157.99, 145.32, 139.14, 133.56, 133.45 (d, *J* = 2.99 Hz, 1C), 130.65, 130.09 (d, *J* = 8.27 Hz, 2C), 128.69, 127.89, 127.78, 127.55, 125.73, 124.94, 123.81, 115.83 (d, *J* = 21.63 Hz, 2C), 114.83, 113.34, 72.28, 69.75, 67.66, 42.96, 40.63, 26.30, 26.10 ppm. MS (*m*/*z*): 567 [M + H]^+^.

*2-(4-{[5-(Benzyloxy)pentyl]oxy}phenyl)-N-{2-[(4-fluorophenyl)methyl]-1,3-dioxo-2,3-dihydro-1H-iso-indol-5-yl}acetamide* (**12m**; ZHAWOC5682): The title compound **12m** was obtained as a yellow solid in 58% yield: ^1^H-NMR (DMSO-*d*_6_): δ = 10.72 (s, 1H), 8.20 (d, *J* = 1.82 Hz, 1H), 7.88 (dd, *J* = 8.27 Hz, 1.82 Hz, 1H), 7.83 (d, *J* = 8.27 Hz, 1H), 7.36–7.21 (m, 9H), 7.17–7.11 (m, 2H), 6.90–6.85 (m, 2H), 4.72 (s, 2H), 4.44 (s, 2H), 3.92 (t, *J* = 6.45 Hz, 2H), 3.63 (s, 2H), 3.43 (t, *J* = 6.40 Hz, 2H), 1.73–1.66 (m, 2H), 1.62–1.55 (m, 2H), 1.50–1.42 (m, 2H) ppm. ^13^C-NMR (DMSO-*d*_6_): δ = 170.92, 167.89, 167.71, 161.90 (d, *J* = 243.23 Hz, 1C), 158.01, 145.30, 139.17, 133.54, 133.45 (d, *J* = 3.06 Hz, 1C), 130.63, 130.08 (d, *J* = 8.28 Hz, 2C), 128.66, 127.84, 127.75, 127.50, 125.71, 124.92, 123.80, 115.81 (d, *J* = 21.43 Hz, 2C), 114.81, 113.33, 72.27, 69.97, 67.79, 42.96, 40.62, 29.38, 28.95, 22.84 ppm. MS (*m*/*z*): 603 [M + Na]^+^.

*2-(4-{[6-(Benzyloxy)hexyl]oxy}phenyl)-N-{2-[(4-fluorophenyl)methyl]-1,3-dioxo-2,3-dihydro-1H-iso-indol-5-yl}acetamide* (**12n**; ZHAWOC6640): The title compound **12n** was obtained as a yellow solid in 36% yield: ^1^H-NMR (DMSO-*d*_6_): δ = 10.73 (s, 1H), 8.21 (d, *J* = 1.83 Hz, 1H), 7.89 (dd, *J* = 8.24 Hz, 1.83 Hz, 1H), 7.83 (d, *J* = 8.24 Hz, 1H), 7.36–7.26 (m, 7H), 7.25–7.21 (m, 2H), 7.17–7.11 (m, 2H), 6.89–6.85 (m, 2H), 4.72 (s, 2H), 4.43 (s, 2H), 3.92 (t, *J* = 6.38 Hz, 2H), 3.63 (s, 2H), 3.41 (t, *J* = 6.45 Hz, 2H), 1.73–1.65 (m, 2H), 1.59–1.51 (m, 2H), 1.44–1.33 (m, 4H) ppm. ^13^C-NMR (DMSO-*d*_6_): δ = 170.93, 167.89, 167.72, 161.90 (d, *J* = 243.56 Hz, 1C), 158.03, 145.31, 139.19, 133.54, 133.44 (d, *J* = 3.08 Hz, 1C), 130.63, 130.08 (d, *J* = 8.34 Hz, 2C), 128.66, 127.84, 127.74, 127.49, 125.71, 124.92, 123.80, 115.81 (d, *J* = 21.43 Hz, 2C), 114.80, 113.32, 72.25, 69.99, 67.79, 42.96, 40.62, 29.62, 29.11, 25.95, 25.83 ppm. MS (*m*/*z*): 595 [M + H]^+^.

*2-(4-{[7-(Benzyloxy)heptyl]oxy}phenyl)-N-{2-[(4-fluorophenyl)methyl]-1,3-dioxo-2,3-dihydro-1H-iso-indol-5-yl}acetamide* (**12o**; ZHAWOC6635): The title compound **12o** was obtained as a yellow solid in 47% yield: ^1^H-NMR (DMSO-*d*_6_): δ = 10.72 (s, 1H), 8.20 (d, *J* = 1.81 Hz, 1H), 7.88 (dd, *J* = 8.26 Hz, 1.81 Hz, 1H), 7.83 (d, *J* = 8.24 Hz, 1H), 7.36–7.26 (m, 7H), 7.25–7.21 (m, 2H), 7.17–7.11 (m, 2H), 6.89–6.85 (m, 2H), 4.71 (s, 2H), 4.42 (s, 2H), 3.91 (t, *J* = 6.35 Hz, 2H), 3.63 (s, 2H), 3.40 (t, *J* = 6.45 Hz, 2H), 1.71–1.64 (m, 2H), 1.57–1.49 (m, 2H), 1.42–1.27 (m, 6H) ppm. ^13^C-NMR (DMSO-*d*_6_): δ = 170.92, 167.89, 167.71, 161.90 (d, *J* = 242.72 Hz, 1C), 158.03, 145.30, 139.20, 133.54, 133.44 (d, *J* = 2.97 Hz, 1C), 130.63, 130.08 (d, *J* = 8.38 Hz, 2C), 128.65, 127.83, 127.73, 127.49, 125.71, 124.92, 123.80, 115.81 (d, *J* = 21.54 Hz, 2C), 114.80, 113.32, 72.24, 70.02, 67.80, 42.96, 40.62, 29.59, 29.09, 29.01, 26.12, 25.95 ppm. MS (*m*/*z*): 609 [M + H]^+^.

*2-(4-{[8-(Benzyloxy)octyl]oxy}phenyl)-N-{2-[(4-fluorophenyl)methyl]-1,3-dioxo-2,3-dihydro-1H-iso-indol-5-yl}acetamide* (**12p**; ZHAWOC6638): The title compound **12p** was obtained as a yellow solid in 57% yield: ^1^H-NMR (DMSO-*d*_6_): δ = 10.72 (s, 1H), 8.20 (d, *J* = 1.81 Hz, 1H), 7.89 (dd, *J* = 8.23 Hz, 1.81 Hz, 1H), 7.83 (d, *J* = 8.23 Hz, 1H), 7.37–7.26 (m, 7H), 7.25–7.21 (m, 2H), 7.17–7.11 (m, 2H), 6.89–6.85 (m, 2H), 4.71 (s, 2H), 4.42 (s, 2H), 3.91 (t, *J* = 6.51 Hz, 2H), 3.63 (s, 2H), 3.40 (t, *J* = 6.51 Hz, 2H), 1.71–1.64 (m, 2H), 1.57–1.49 (m, 2H), 1.42–1.25 (m, 8H) ppm. ^13^C-NMR (DMSO-*d*_6_): δ = 170.93, 167.90, 167.72, 161.90 (d, *J* = 243.01 Hz, 1C), 158.03, 145.30, 139.20, 133.55, 133.44 (d, *J* = 3.21 Hz, 1C), 130.63, 130.08 (d, *J* = 8.28 Hz, 2C), 128.65, 127.83, 127.74, 127.48, 125.71, 124.93, 123.80, 115.81 (d, *J* = 21.37 Hz, 2C), 114.80, 113.32, 72.24, 70.04, 67.82, 42.96, 40.62, 29.63, 29.24, 29.18, 29.12, 26.11, 25.93 ppm. MS (*m*/*z*): 623 [M + H]^+^.

*2-{4-[(5-Hydroxypentyl)oxy]phenyl}-N-(2-methyl-1,3-dioxo-2,3-dihydro-1H-isoindol-5-yl)acetamide* (**13a**; ZHAWOC6648): The benzyl ether **12a** (0.12 g, 0.25 mmol) was dissolved in dichloromethane (3.00 mL) under an argon atmosphere. Trimethylsilyl iodide (0.35 ml) was added and stirred at room temperature for 2 h. The reaction was quenched by an addition of methanol (10 mL). The solvent was removed in vacuum and the residue purified by column chromatography on silica gel (gradient: 0–100% methanol in dichloromethane) to afford the title compound **13a** as a white solid (0.03 g, 28% yield): ^1^H-NMR (DMSO-*d*_6_): δ = 10.73 (s, 1H), 8.18 (d, *J* = 1.86 Hz, 1H), 7.85 (dd, *J* = 8.22 Hz, 1.86 Hz, 1H), 7.80 (d, *J* = 8.22 Hz, 1H), 7.26–7.21 (m, 2H), 6.90–6.86 (m, 2H), 4.36 (t, *J* = 5.10 Hz, 1H), 3.93 (t, *J* = 6.45 Hz, 2H), 3.63 (s, 2H), 3.40 (td, *J* = 5.82 Hz, 5.10 Hz, 2H), 3.00 (s, 3H), 1.74–1.66 (m, 2H), 1.51–1.38 (m, 4H) ppm. ^13^C-NMR (DMSO-*d*_6_): δ = 170.92, 168.27, 168.13, 158.05, 145.06, 133.82, 130.65, 127.55, 126.08, 124.56, 123.5, 114.81, 113.10, 67.89, 61.08, 42.96, 32.69, 29.06, 24.19, 22.61 ppm. MS (*m*/*z*): 397 [M + H]^+^.

*N-(2-Benzyl-1,3-dioxo-2,3-dihydro-1H-isoindol-5-yl)-2-[4-(2-hydroxyethoxy)phenyl]acetamide* (**13b**; ZHAWOC5473): The benzyl ether (**12b**) (0.77 g, 1.48 mmol) was dissolved in ethanol (70 mL). Palladium 10% on activated charcoal (0.15 g, 0.13 mmol) was added and a hydrogen atmosphere was applied at 1 bar. After stirring at ambient temperature for 2 h the mixture was filtered over celite and concentrated in vacuum. Purification by chromatography on silica gel (gradient: 0–100% ethyl acetate in cyclohexane) afforded the title compound **13b** as a white solid (0.25 g, 39% yield): ^1^H-NMR (DMSO-*d*_6_): δ = 10.74 (br. s, 1H), 8.21 (d, *J* = 1.78 Hz, 1H), 7.89 (dd, *J* = 8.20 Hz, 1.78 Hz, 1H), 7.83 (d, *J* = 8.20 Hz, 1H), 7.34–7.22 (m, 7H), 6.92–6.87 (m, 2H), 4.84 (t, *J* = 4.79 Hz, 1H), 4.73 (s, 2H), 3.95 (t, *J* = 5.04 Hz, 2H), 3.70 (q, *J* = 4.45 Hz, 2H), 3.64 (m, 2H) ppm. ^13^C-NMR (DMSO-*d*_6_): δ = 170.93, 167.94, 167.76, 158.04, 145.32, 137.21, 133.54, 130.65, 129.04, 127.86, 127.83, 127.59, 125.71, 124.92, 123.81, 114.84, 113.33, 69.94, 60.04, 42.95, 41.31 ppm. MS (*m*/*z*): 431 [M + H]^+^.

In analogy to ZHAWOC5473 the following derivatives were synthesized:

*N-(2-Benzyl-1,3-dioxo-2,3-dihydro-1H-isoindol-5-yl)-2-[4-(3-hydroxypropoxy)phenyl]acetamide* (**13c**; ZHAWOC4512): The title compound **13c** was obtained as a white solid (0.65 g, 71% yield): ^1^H-NMR (DMSO-*d*_6_): δ = 10.74 (s, 1H), 8.21 (d, *J* = 1.81 Hz, 1H), 7.89 (dd, *J* = 8.25 Hz, 1.81 Hz, 1H), 7.84 (d, *J* = 8.25 Hz, 1H), 7.35–7.21 (m, 7H), 6.91–6.86 (m, 2H), 4.73 (s, 2H), 4.52 (t, *J* = 4.60 Hz, 1H), 4.00 (t, *J* = 6.42 Hz, 2H), 3.64 (s, 2H), 3.54 (m, 2H), 1.84 (quint., *J* = 6.31 Hz, 2H) ppm. ^13^C-NMR (DMSO-*d*_6_): δ = 170.93, 167.94, 167.76, 158.03, 145.30, 137.21, 133.54, 130.64, 129.04, 127.86, 127.83, 127.51, 125.72, 124.92, 123.81, 114.80, 113.32, 64.99, 57.77, 42.95, 41.31, 32.60 ppm. MS (*m*/*z*): 445 [M + H]^+^.

*N-(2-Benzyl-1,3-dioxo-2,3-dihydro-1H-isoindol-5-yl)-2-[4-(4-hydroxybutoxy)phenyl]acetamide* (**13d**; ZHAWOC4753): The title compound **13d** was obtained as a white solid in 60% yield: ^1^H-NMR (DMSO-*d*_6_): δ = 10.74 (s, 1H), 8.21 (d, *J* = 1.83 Hz, 1H), 7.90 (dd, *J* = 8.24 Hz, 1.83 Hz, 1H), 7.84 (d, *J* = 8.24 Hz, 1H), 7.35–7.22 (m, 7H), 6.91–6.86 (m, 2H), 4.74 (s, 2H), 4.44 (t, *J* = 4.54 Hz, 1H), 4.95 (t, *J* = 6.44 Hz, 2H), 3.64 (s, 2H), 3.44 (m, 2H), 1.76–1.69 (m, 2H), 1.59–1.51 (m, 2H) ppm. ^13^C-NMR (DMSO-*d*_6_): δ = 170.94, 167.94, 167.76, 158.02, 145.32, 137.21, 133.54, 130.54, 129.04, 127.86, 127.83, 127.50, 125.71, 124.92, 123.81, 114.81, 113.33, 67.82, 60.86, 42.96, 41.31, 29.47, 25.91 ppm. MS (*m*/*z*): 459 [M + H]^+^.

*N-(2-Benzyl-1,3-dioxo-2,3-dihydro-1H-isoindol-5-yl)-2-{4-[(5-hydroxypentyl)oxy]phenyl}acetamide* (**13e**; ZHAWOC5130): The title compound **13e** was obtained as a white solid in 24% yield: ^1^H-NMR (DMSO-*d*_6_): δ = 10.72 (s, 1H), 8.21 (d, *J* = 1.84 Hz, 1H), 7.89 (dd, *J* = 8.26 Hz, 1.84 Hz, 1H), 7.84 (d, *J* = 8.26 Hz, 1H), 7.35–7.25 (m, 5H), 7.25–7.21 (m, 2H), 6.90–6.85 (m, 2H), 4.73 (s, 2H), 4.36 (t, *J* = 5.16 Hz, 1H), 3.93 (t, *J* = 6.50 Hz, 2H), 3.63 (s, 2H), 3.40 (td, *J* = 6.20 Hz, 5.16 Hz, 2H), 1.73–1.66 (m, 2H), 1.50–1.38 (m, 4H) ppm. ^13^C-NMR (DMSO-*d*_6_): δ = 170.94, 167.94, 167.77, 158.04, 145.30, 137.21, 133.54, 130.64, 129.05, 127.86, 127.83, 127.49, 125.72, 124.93, 123.81, 114.81, 113.33, 67.89, 61.07, 42.96, 41.32, 32.68, 29.06, 22.61 ppm. MS (*m*/*z*): 473 [M + H]^+^.

*N-(2-Benzyl-1,3-dioxo-2,3-dihydro-1H-isoindol-5-yl)-2-{4-[(6-hydroxyhexyl)oxy]phenyl}acetamide* (**13f**; ZHAWOC5132): The title compound **13f** was obtained as a white solid in 6% yield: ^1^H-NMR (DMSO-*d*_6_): δ = 10.73 (s, 1H), 8.21 (d, *J* = 1.82 Hz, 1H), 7.89 (dd, *J* = 8.23 Hz, 1.82 Hz, 1H), 7.84 (d, *J* = 8.23 Hz, 1H), 7.35–7.25 (m, 5H), 7.25–7.21 (m, 2H), 6.90–6.85 (m, 2H), 4.74 (s, 2H), 4.33 (t, *J* = 5.16 Hz, 1H), 3.92 (t, *J* = 6.50 Hz, 2H), 3.63 (s, 2H), 3.38 (td, *J* = 6.32 Hz, 5.16 Hz, 2H), 1.72–1.65 (m, 2H), 1.47–1.29 (m, 6H) ppm. ^13^C-NMR (DMSO-*d*_6_): δ = 170.94, 167.94, 167.77, 158.03, 145.30, 137.21, 133.54, 130.64, 129.05, 127.86, 127.83, 127.49, 125.72, 124.93, 123.81, 114.81, 113.33, 67.82, 61.10, 42.95, 41.32, 32.95, 29.22, 25.90, 25.74 ppm. MS (*m*/*z*): 487 [M + H]^+^.

*N-(2-Benzyl-1,3-dioxo-2,3-dihydro-1H-isoindol-5-yl)-2-{4-[(7-hydroxyheptyl)oxy]phenyl}acetamide* (**13g**; ZHAWOC7103): The title compound **13g** was obtained as a white solid in 24% yield: ^1^H-NMR (DMSO-*d*_6_): δ = 10.74 (s, 1H), 8.21 (d, *J* = 1.83 Hz, 1H), 7.89 (dd, *J* = 8.25 Hz, 1.83 Hz, 1H), 7.84 (d, *J* = 8.25 Hz, 1H), 7.35–7.21 (m, 7H), 6.90–6.86 (m, 2H), 4.74 (s, 2H), 4.32 (t, *J* = 4.98 Hz, 1H), 3.92 (t, *J* = 6.43 Hz, 2H), 3.63 (s, 2H), 3.37 (td, *J* = 6.43 Hz, 4.98 Hz, 2H), 1.72–1.65 (m, 2H), 1.45–1.25 (m, 8H) ppm. ^13^C-NMR (DMSO-*d*_6_): δ = 170.94, 167.94, 167.77, 158.03, 145.30, 137.21, 133.54, 130.64, 129.05, 127.86, 127.82, 127.49, 125.72, 124.93, 123.81, 114.81, 113.32, 67.83, 61.14, 42.96, 41.31, 32.94, 29.14, 29.12, 26.04, 25.93 ppm. MS (*m*/*z*): 501 [M + H]^+^.

*N-(2-Benzyl-1,3-dioxo-2,3-dihydro-1H-isoindol-5-yl)-2-{4-[(8-hydroxyoctyl)oxy]phenyl}acetamide* (**13h**; ZHAWOC7137): The title compound **13h** was obtained as a white solid in 59% yield: ^1^H-NMR (DMSO-*d*_6_): δ = 10.73 (s, 1H), 8.21 (d, *J* = 1.80 Hz, 1H), 7.89 (dd, *J* = 8.20 Hz, 1.80 Hz, 1H), 7.84 (d, *J* = 8.20 Hz, 1H), 7.36–7.18 (m, 7H), 6.92–6.83 (m, 2H), 4.74 (s, 2H), 4.31 (t, *J* = 4.90 Hz, 1H), 3.92 (t, *J* = 6.13 Hz, 2H), 3.63 (s, 2H), 3.37 (td, *J* = 6.43 Hz, 4.90 Hz, 2H), 1.75–1.64 (m, 2H), 1.46–1.19 (m, 10H) ppm. ^13^C-NMR (DMSO-*d*_6_): δ = 170.95, 167.96, 167.78, 158.05, 145.32, 137.23, 133.56, 130.65, 129.06, 127.87, 127.84, 127.50, 125.73, 124.94, 123.82, 114.81, 113.33, 67.85, 61.18, 42.96, 41.32, 33.00, 29.38, 29.30, 29.16, 25.98, 25.93 ppm. MS (*m*/*z*): 515 [M + H]^+^.

*N-(2-Benzyl-1,3-dioxo-2,3-dihydro-1H-isoindol-5-yl)-2-{4-[(9-hydroxynonyl)oxy]phenyl}acetamide* (**13i**; ZHAWOC6936): The title compound **13i** was obtained as a white solid in 30% yield: ^1^H-NMR (DMSO-*d*_6_): δ = 10.74 (s, 1H), 8.21 (d, *J* = 1.83 Hz, 1H), 7.89 (dd, *J* = 8.24 Hz, 1.83 Hz, 1H), 7.83 (d, *J* = 8.24 Hz, 1H), 7.35–7.21 (m, 7H), 6.90–6.85 (m, 2H), 4.73 (s, 2H), 4.31 (t, *J* = 5.12 Hz, 1H), 3.92 (t, *J* = 6.46 Hz, 2H), 3.63 (s, 2H), 3.36 (td, *J* = 6.25 Hz, 5.12 Hz, 2H), 1.71–1.64 (m, 2H), 1.43–1.23 (m, 12H) ppm. ^13^C-NMR (DMSO-*d*_6_): δ = 170.93, 167.93, 167.76, 158.03, 145.31, 137.21, 133.54, 130.63, 129.04, 127.85, 127.83, 127.49, 125.71, 124.92, 123.81, 114.80, 113.33, 67.84, 61.18, 42.96, 41.31, 33.01, 29.53, 29.37, 29.22, 29.16, 26.00, 25.96 ppm. MS (*m*/*z*): 529 [M + H]^+^.

*N-(2-Benzyl-1,3-dioxo-2,3-dihydro-1H-isoindol-5-yl)-2-{4-[(10-hydroxydecyl)oxy]phenyl}acetamide* (**13j**; ZHAWOC6937): The title compound **13j** was obtained as a white solid in 18% yield: ^1^H-NMR (DMSO-*d*_6_): δ = 10.73 (s, 1H), 8.21 (d, *J* = 1.80 Hz, 1H), 7.89 (dd, *J* = 8.16 Hz, 1.80 Hz, 1H), 7.84 (d, *J* = 8.16 Hz, 1H), 7.34–7.21 (m, 7H), 6.89–6.85 (m, 2H), 4.73 (s, 2H), 4.31 (t, *J* = 4.99 Hz, 1H), 3.92 (t, *J* = 6.46 Hz, 2H), 3.63 (s, 2H), 3.36 (td, *J* = 6.25 Hz, 4.99 Hz, 2H), 1.72–1.64 (m, 2H), 1.42–1.22 (m, 14H) ppm. ^13^C-NMR (DMSO-*d*_6_): δ = 170.93, 167.94, 167.76, 158.03, 145.30, 137.21, 133.54, 130.64, 129.04, 127.86, 127.83, 127.49, 125.72, 124.93, 123.80, 114.80, 113.32, 67.83, 61.18, 42.95, 41.31, 33.01, 29.51, 29.45 29.40, 29.24, 29.15, 26.81, 25.97 ppm. MS (*m*/*z*): 543 [M + H]^+^.

*N-{2-[(4-Fluorophenyl)methyl]-1,3-dioxo-2,3-dihydro-1H-isoindol-5-yl}-2-[4-(3-hydroxypropoxy)-phenyl]acetamide* (**13k**; ZHAWOC6642): The title compound **13k** was obtained as a white solid in 34% yield: ^1^H-NMR (DMSO-*d*_6_): δ = 10.73 (s, 1H), 8.20 (d, *J* = 1.99 Hz, 1H), 7.89 (dd, *J* = 8.24 Hz, 1.99 Hz, 1H), 7.83 (d, *J* = 8.24 Hz, 1H), 7.37–7.32 (m, 2H), 7.25–7.21 (m, 2H), 7.17–7.11 (m, 2H), 6.90–6.86 (m, 2H), 4.72 (s, 2H), 4.52 (t, *J* = 5.15 Hz, 1H), 4.00 (t, *J* = 6.53 Hz, 2H), 3.63 (s, 2H), 3.56–3.52 (m, 2H), 1.84 (quint. *J* = 6.25 Hz, 2H) ppm. ^13^C-NMR (DMSO-*d*_6_): δ = 170.95, 167.92, 167.74, 161.92 (d, *J* = 242.66 Hz, 1C), 158.05, 145.32, 133.56, 133.46 (d, *J* = 2.98 Hz, 1C), 130.65, 130.09 (d, *J* = 8.37 Hz, 2C), 127.52, 125.73, 124.94, 123.82, 115.83 (d, *J* = 21.42 Hz, 2C), 114.81, 113.34, 65.00, 57.78, 42.95, 40.63, 32.60 ppm. MS (*m*/*z*): 463 [M + H]^+^.

*N-{2-[(4-Fluorophenyl)methyl]-1,3-dioxo-2,3-dihydro-1H-isoindol-5-yl}-2-[4-(4-hydroxybutoxy)-phenyl]acetamide* (**13l**; ZHAWOC5462): The title compound **13l** was obtained as a white solid in 15% yield: ^1^H-NMR (DMSO-*d*_6_): δ = 10.73 (s, 1H), 8.20 (d, *J* = 1.83 Hz, 1H), 7.89 (dd, *J* = 8.22 Hz, 1.83 Hz, 1H), 7.83 (d, *J* = 8.22 Hz, 1H), 7.37–7.32 (m, 2H), 7.25–7.21 (m, 2H), 7.17–7.11 (m, 2H), 6.90–6.86 (m, 2H), 4.72 (s, 2H), 4.43 (t, *J* = 5.11 Hz, 1H), 3.94 (t, *J* = 6.58 Hz, 2H), 3.63 (s, 2H), 3.44 (td, *J* = 6.38 Hz, 5.11 Hz, 2H), 1.76–1.68 (m, 2H), 1.58–1.51 (m, 2H) ppm. ^13^C-NMR (DMSO-*d*_6_): δ = 170.93, 167.90, 167.72, 161.90 (d, *J* = 243.27 Hz, 1C), 158.02, 145.30, 133.55, 133.44 (d, *J* = 3.06 Hz, 1C), 130.64, 130.08 (d, *J* = 8.27 Hz, 2C), 127.49, 125.71, 124.93, 123.80, 115.82 (d, *J* = 21.38 Hz, 2C), 114.81, 113.32, 67.82, 60.86, 42.95, 40.62, 29.47, 25.91 ppm. MS (*m*/*z*): 477 [M + H]^+^.

*N-{2-[(4-Fluorophenyl)methyl]-1,3-dioxo-2,3-dihydro-1H-isoindol-5-yl}-2-{4-[(5-hydroxypentyl)oxy]-phenyl}acetamide* (**13m**; ZHAWOC5683): The title compound **13m** was obtained as a white solid in 44% yield: ^1^H-NMR (DMSO-*d*_6_): δ = 10.73 (s, 1H), 8.20 (d, *J* = 1.84 Hz, 1H), 7.89 (dd, *J* = 8.20 Hz, 1.84 Hz, 1H), 7.83 (d, *J* = 8.20 Hz, 1H), 7.37–7.32 (m, 2H), 7.25–7.21 (m, 2H), 7.17–7.11 (m, 2H), 6.90–6.86 (m, 2H), 4.72 (s, 2H), 4.36 (t, *J* = 5.10 Hz, 1H), 3.93 (t, *J* = 6.45 Hz, 2H), 3.63 (s, 2H), 3.42–3.38 (m, 2H), 1.73–1.65 (m, 2H), 1.50–1.37 (m, 4H) ppm. ^13^C-NMR (DMSO-*d*_6_): δ = 170.94, 167.90, 167.72, 161.90 (d, *J* = 242.58 Hz, 1C), 158.04, 145.30, 133.55, 133.44 (d, *J* = 3.08 Hz, 1C), 130.64, 130.07 (d, *J* = 8.33 Hz, 2C), 127.49, 125.72, 124.93, 123.81, 115.82 (d, *J* = 21.42 Hz, 2C), 114.80, 113.33, 67.88, 61.07, 42.95, 40.62, 32.68, 29.05, 22.61 ppm. MS (*m*/*z*): 513 [M + Na]^+^.

*N-{2-[(4-Fluorophenyl)methyl]-1,3-dioxo-2,3-dihydro-1H-isoindol-5-yl}-2-{4-[(6-hydroxyhexyl)oxy]-phenyl}acetamide* (**13n**; ZHAWOC6643): The title compound **13n** was obtained as a white solid in 26% yield: ^1^H-NMR (DMSO-*d*_6_): δ = 10.73 (s, 1H), 8.20 (d, *J* = 1.86 Hz, 1H), 7.88 (dd, *J* = 8.19 Hz, 1.86 Hz, 1H), 7.83 (d, *J* = 8.19 Hz, 1H), 7.37–7.32 (m, 2H), 7.25–7.21 (m, 2H), 7.17–7.11 (m, 2H), 6.90–6.86 (m, 2H), 4.72 (s, 2H), 4.33 (t, *J* = 5.12 Hz, 1H), 3.92 (t, *J* = 6.51 Hz, 2H), 3.63 (s, 2H), 3.38 (td, *J* = 6.32 Hz, 5.12 Hz, 2H), 1.73–1.64 (m, 2H), 1.47–1.28 (m, 6H) ppm. ^13^C-NMR (DMSO-*d*_6_): δ = 170.95, 167.92, 167.74, 161.92 (d, *J* = 242.69 Hz, 1C), 158.05, 145.31, 133.56, 133.46 (d, *J* = 3.11 Hz, 1C), 130.65, 130.09 (d, *J* = 8.39 Hz, 2C), 127.50, 125.73, 124.94, 123.82, 115.83 (d, *J* = 21.32 Hz, 2C), 114.82, 113.34, 67.82, 61.11, 42.96, 40.63, 32.96, 29.22, 25.91, 25.75 ppm. MS (*m*/*z*): 505 [M + H]^+^.

*N-{2-[(4-Fluorophenyl)methyl]-1,3-dioxo-2,3-dihydro-1H-isoindol-5-yl}-2-{4-[(7-hydroxyheptyl)oxy]-phenyl}acetamide* (**13o**; ZHAWOC6636): The title compound **13o** was obtained as a white solid in 53% yield: ^1^H-NMR (DMSO-*d*_6_): δ = 10.74 (s, 1H), 8.20 (d, *J* = 1.83 Hz, 1H), 7.89 (dd, *J* = 8.18 Hz, 1.83 Hz, 1H), 7.83 (d, *J* = 8.18 Hz, 1H), 7.37–7.32 (m, 2H), 7.25–7.21 (m, 2H), 7.17–7.11 (m, 2H), 6.90–6.86 (m, 2H), 4.72 (s, 2H), 4.32 (t, *J* = 5.11 Hz, 1H), 3.92 (t, *J* = 6.48 Hz, 2H), 3.63 (s, 2H), 3.37 (td, *J* = 6.35 Hz, 5.11 Hz, 2H), 1.73–1.64 (m, 2H), 1.45–1.23 (m, 8H) ppm. ^13^C-NMR (DMSO-*d*_6_): δ = 170.94, 167.90, 167.72, 161.90 (d, *J* = 243.10 Hz, 1C), 158.03, 145.31, 133.55, 133.45 (d, *J* = 3.10 Hz, 1C), 130.64, 130.07 (d, *J* = 8.23 Hz, 2C), 127.49, 125.71, 124.93, 123.81, 115.82 (d, *J* = 21.48 Hz, 2C), 114.80, 113.33, 67.83, 61.14, 42.96, 40.62, 32.94, 29.14, 29.13, 26.04, 25.93 ppm. MS (*m*/*z*): 519 [M + H]^+^.

*N-{2-[(4-Fluorophenyl)methyl]-1,3-dioxo-2,3-dihydro-1H-isoindol-5-yl}-2-{4-[(8-hydroxyoctyl)oxy]-phenyl}acetamide* (**13p**; ZHAWOC6639): The title compound **13p** was obtained as a white solid in 24% yield: ^1^H-NMR (DMSO-*d*_6_): δ = 10.73 (s, 1H), 8.20 (d, *J* = 1.88 Hz, 1H), 7.89 (dd, *J* = 8.20 Hz, 1.88 Hz, 1H), 7.83 (d, *J* = 8.20 Hz, 1H), 7.37–7.32 (m, 2H), 7.25–7.21 (m, 2H), 7.17–7.11 (m, 2H), 6.90–6.86 (m, 2H), 4.72 (s, 2H), 4.31 (t, *J* = 5.12 Hz, 1H), 3.92 (t, *J* = 6.57 Hz, 2H), 3.63 (s, 2H), 3.37 (td, *J* = 6.48 Hz, 5.12 Hz, 2H), 1.73–1.64 (m, 2H), 1.45–1.21 (m, 10H) ppm. ^13^C-NMR (DMSO-*d*_6_): δ = 170.94, 167.90, 167.72, 161.90 (d, *J* = 243.48 Hz, 1C), 158.03, 145.30, 133.55, 133.44 (d, *J* = 3.05 Hz, 1C), 130.64, 130.07 (d, *J* = 8.38 Hz, 2C), 127.48, 125.72, 124.93, 123.81, 115.82 (d, *J* = 21.51 Hz, 2C), 114.80, 113.33, 67.84, 61.17, 42.95, 40.62, 32.99, 29.38, 29.30, 29.15, 25.97, 25.93 ppm. MS (*m*/*z*): 533 [M + H]^+^.

*5-(4-{[(2-Methyl-1,3-dioxo-2,3-dihydro-1H-isoindol-5-yl)carbamoyl]methyl}phenoxy)pentanoic acid* (**14a**; ZHAWOC6649): The alcohol (**13a**) (27 mg, 0.07 mmol), TEMPO (24 mg, 0.02 mmol) and 0.67 M aqueous sodium phosphate (0.80 ml) in acetonitrile (1.00 mL) were stirred and heated to 40 °C. NaClO_2_ (40 mg, in 0.12 mL water) and 0.25% NaOCl (0.05 mL) were added in parallel dropwise and it was stirred for 4 h. After cooling to ambient temperature, water (1.0 mL) was added and the mixture was poured in an ice cold solution of Na_2_SO_3_ (0.12 g in 2 mL water). The aqueous phase was extracted with diethyl ether (10 mL), acidified with 10% citric acid and again extracted with diethyl ether (2 × 10 mL). The organic phases were dried over sodium sulfate and concentrated in vacuum to obtain the title compound **14a** as a white solid (10 mg, 36% yield, purity 96%): ^1^H-NMR (DMSO-*d*_6_): δ = 12.04 (br. s, 1H), 10.75 (s, 1H), 8.18 (d, *J* = 1.82 Hz, 1H), 7.86 (dd, *J* = 8.15 Hz, 1.82 Hz, 1H), 7.80 (d, *J* = 8.15 Hz, 1H), 7.26–7.22 (m, 2H), 6.90–6.86 (m, 2H), 3.93 (t, *J* = 6.25 Hz, 2H), 3.63 (s, 2H), 3.00 (s, 3H), 2.23 (t, *J* = 7.43 Hz, 2H), 1.74–1.67 (m, 2H), 1.66–1.59 (m, 2H) ppm. ^13^C-NMR (DMSO-*d*_6_): δ = 170.90, 168.26, 168.13, 157.99, 145.06, 133.81, 130.63, 127.58, 126.07, 124.54, 123.53, 114.81, 113.11, 67.61, 42.96, 34.34, 28.70, 24.19, 21.90 ppm. HRMS-TOF (*m*/*z*): [M + H]^+^ calculated for C_22_H_22_N_2_O_6_: 410.1478, found: 410.1473.

In analogy to ZHAWOC6649 the following derivatives were synthesized:

*2-(4-{[(2-Benzyl-1,3-dioxo-2,3-dihydro-1H-isoindol-5-yl)carbamoyl]methyl}phenoxy)acetic acid* (**14b**; ZHAWOC5474): The title compound **14b** was obtained as a white solid in 38% yield and 98% purity: ^1^H-NMR (DMSO-*d*_6_): δ = 11.54 (s, 1H), 8.27 (d, *J* = 1.80 Hz, 1H), 7.99 (dd, *J* = 8.26 Hz, 1.80 Hz, 1H), 7.80 (d, *J* = 8.26 Hz, 1H), 7.34–7.23 (m, 5H), 7.18–7.13 (m, 2H), 6.77–6.72 (m, 2H), 4.73 (s, 2H), 4.13 (s, 2H), 3.62 (s, 2H) ppm. ^13^C-NMR (DMSO-*d*_6_): δ = 171.34, 171.20, 168.02, 167.83, 158.38, 145.76, 137.25, 133.43, 130.17, 129.04, 127.82, 127.79, 126.93, 125.44, 124.75, 123.98, 114.77, 113.46, 68.32, 42.95, 41.27 ppm. HRMS-TOF (*m*/*z*): [M + H]^+^ calculated for C_25_H_20_N_2_O_6_: 444.1321, found: 444.1330.

*3-(4-{[(2-Benzyl-1,3-dioxo-2,3-dihydro-1H-isoindol-5-yl)carbamoyl]methyl}phenoxy)propanoic acid* (**14c**; ZHAWOC4765): The title compound **14c** was obtained as a white solid in 86% yield and 98% purity: ^1^H-NMR (DMSO-*d*_6_): δ = 11.94 (br. s, 1H), 10.77 (s, 1H), 8.21 (d, *J* = 1.75 Hz, 1H), 7.90 (dd, *J* = 8.22 Hz, 1.75 Hz, 1H), 7.83 (d, *J* = 8.22 Hz, 1H), 7.35–7.22 (m, 7H), 6.91–6.87 (m, 2H), 4.73 (s, 2H), 4.14 (t, *J* = 6.02 Hz, 2H), 3.64 (s, 2H), 2.67 (t, *J* = 6.02 Hz, 2H) ppm. ^13^C-NMR (DMSO-*d*_6_): δ = 172.81, 171.71, 170.91, 167.94, 167.77, 157.70, 145.32, 137.21, 133.53, 130.69, 129.04, 127.85, 127.82, 125.71, 124.91, 123.82, 114.80, 113.34, 64.08, 42.93, 41.31, 34.67 ppm. HRMS-TOF (*m*/*z*): [M + H]^+^ calculated for C_26_H_22_N_2_O_6_: 459.1557, found: 459.1538.

*4-(4-{[(2-Benzyl-1,3-dioxo-2,3-dihydro-1H-isoindol-5-yl)carbamoyl]methyl}phenoxy)butanoic acid* (**14d**; ZHAWOC4766): The title compound **14d** was obtained as a white solid in 67% yield and 95% purity: ^1^H-NMR (DMSO-*d*_6_): δ = 12.14 (br. s, 1H), 10.73 (s, 1H), 8.21 (d, *J* = 1.80 Hz, 1H), 7.89 (dd, *J* = 8.21 Hz, 1.80 Hz, 1H), 7.84 (d, *J* = 8.21 Hz, 1H), 7.34–7.21 (m, 7H), 6.90–6.86 (m, 2H), 4.73 (s, 2H), 3.95 (t, *J* = 6.41 Hz, 2H), 3.64 (s, 2H), 2.36 (t, *J* = 6.41 Hz, 2H), 1.92 (quint., *J* = 6.75 Hz, 2H) ppm. ^13^C-NMR (DMSO-*d*_6_): δ = 174.55, 170.92, 167.94, 167.77, 157.86, 145.30, 137.21, 133.54, 130.66, 129.05, 127.86, 127.83, 127.66, 125.72, 124.93, 123.82, 114.84, 113.33, 67.02, 42.94, 41.31, 30.61, 24.72 ppm. HRMS-TOF (*m*/*z*): [M + H]^+^ calculated for C_27_H_24_N_2_O_6_: 473.1713, found: 473.1699.

*5-(4-{[(2-Benzyl-1,3-dioxo-2,3-dihydro-1H-isoindol-5-yl)carbamoyl]methyl}phenoxy)pentanoic acid* (**14e**; ZHAWOC5131): The title compound **14e** was obtained as a white solid in 62% yield and 99% purity: ^1^H-NMR (DMSO-*d*_6_): δ = 10.75 (s, 1H), 8.21 (d, *J* = 1.83 Hz, 1H), 7.89 (dd, *J* = 8.25 Hz, 1.83 Hz, 1H), 7.83 (d, *J* = 8.25 Hz, 1H), 7.34–7.21 (m, 7H), 6.90–6.86 (m, 2H), 4.73 (s, 2H), 3.93 (t, *J* = 6.18 Hz, 2H), 3.63 (s, 2H), 2.27 (t, *J* = 7.35 Hz, 2H), 1.74–1.67 (m, 2H), 1.66–1.59 (m, 2H) ppm. ^13^C-NMR (DMSO-*d*_6_): δ = 174.85, 170.93, 167.94, 167.76, 157.97, 145.31, 137.21, 133.53, 130.64, 129.04, 127.85, 127.82, 127.55, 125.71, 124.91, 123.81, 114.81, 113.33, 67.55, 42.95, 41.31, 33.81, 28.60, 21.71 ppm. HRMS-TOF (*m*/*z*): [M + H]^+^ calculated for C_28_H_26_N_2_O_6_: 487.1870, found: 487.1853.

*6-(4-{[(2-Benzyl-1,3-dioxo-2,3-dihydro-1H-isoindol-5-yl)carbamoyl]methyl}phenoxy)hexanoic acid* (**14f**; ZHAWOC5133): The title compound **14f** was obtained as a white solid in 96% yield and 94% purity: ^1^H-NMR (DMSO-*d*_6_): δ = 12.01 (br. s, 1H), 10.76 (s, 1H), 8.22 (d, *J* = 1.80 Hz, 1H), 7.90 (dd, *J* = 8.27 Hz, 1.80 Hz, 1H), 7.84 (d, *J* = 8.27 Hz, 1H), 7.36–7.21 (m, 7H), 6.90–6.86 (m, 2H), 4.74 (s, 2H), 3.93 (t, *J* = 6.48 Hz, 2H), 3.64 (s, 2H), 2.20 (t, *J* = 7.26 Hz, 2H), 1.73–1.66 (m, 2H), 1.59–1.51 (m, 2H); 1.44–1.37 (m, 2H) ppm. ^13^C-NMR (DMSO-*d*_6_): δ = 177.22, 171.23, 168.02, 167.83, 158.00, 145.78, 137.25, 133.44, 130.63, 129.04, 127.83, 127.79, 127.77, 125.45, 124.77, 123.96, 114.67, 113.47, 67.99, 42.86, 41.27, 38.78, 29.35, 26.76, 26.37 ppm. HRMS-TOF (*m*/*z*): [M + H]^+^ calculated for C_29_H_28_N_2_O_6_: 501.2026, found: 501.2027.

*7-(4-{[(2-Benzyl-1,3-dioxo-2,3-dihydro-1H-isoindol-5-yl)carbamoyl]methyl}phenoxy)heptanoic acid* (**14g**; ZHAWOC6650): The title compound **14g** was obtained as a white solid in 55% yield and 84% purity: ^1^H-NMR (DMSO-*d*_6_): δ = 11.72 (br. s, 1H), 10.73 (s, 1H), 8.21 (d, *J* = 1.81 Hz, 1H), 7.89 (dd, *J* = 8.27 Hz, 1.81 Hz, 1H), 7.84 (d, *J* = 8.27 Hz, 1H), 7.40–7.20 (m, 7H), 6.90–6.85 (m, 2H), 4.74 (s, 2H), 3.92 (t, *J* = 6.41 Hz, 2H), 3.63 (s, 2H), 2.20 (t, *J* = 7.28 Hz, 2H), 1.73–1.64 (m, 2H), 1.55–1.47 (m, 2H); 1.44–1.36 (m, 2H), 1.35–1.28 (m, 2H) ppm. ^13^C-NMR ( DMSO-*d*_6_): δ = 174.95, 170.96, 167.96, 167.79, 158.04, 145.32, 137.23, 133.56, 130.65, 129.06, 127.88, 127.84, 127.65, 125.74, 124.95, 123.83, 114.82, 113.34, 67.78, 42.96, 41.32, 34.06, 29.01, 28.75, 25.72, 24.90 ppm. HRMS-TOF (*m*/*z*): [M + H]^+^ calculated for C_30_H_30_N_2_O_6_: 514.2104, found: 514.2096.

*8-(4-{[(2-Benzyl-1,3-dioxo-2,3-dihydro-1H-isoindol-5-yl)carbamoyl]methyl}phenoxy)octanoic acid* (**14h**; ZHAWOC6651): The title compound **14h** was obtained as a white solid in 50% yield and 92% purity: ^1^H-NMR (DMSO-*d*_6_): δ = 10.79 (s, 1H), 8.21 (d, *J* = 1.81 Hz, 1H), 7.89 (dd, *J* = 8.21 Hz, 1.81 Hz, 1H), 7.84 (d, *J* = 8.21 Hz, 1H), 7.35–7.21 (m, 7H), 6.89–6.85 (m, 2H), 4.73 (s, 2H), 3.92 (t, *J* = 6.48 Hz, 2H), 3.63 (s, 2H), 2.16 (t, *J* = 7.31 Hz, 2H), 1.71–1.64 (m, 2H), 1.52–1.45 (m, 2H); 1.42–1.22 (m, 6H) ppm. ^13^C-NMR (DMSO-*d*_6_): δ = 170.97, 167.96, 167.79, 158.04, 145.35, 137.23, 133.55, 130.65, 129.06, 127.87, 127.84, 127.52, 125.71, 124.93, 123.83, 114.82, 113.35, 67.82, 42.95, 41.31, 34.53, 29.10, 29.04, 28.97, 25.87, 25.08 ppm. HRMS-TOF (*m*/*z*): [M + H]^+^ calculated for C_31_H_32_N_2_O_6_: 528.2260, found: 528.2245.

*9-(4-{[(2-Benzyl-1,3-dioxo-2,3-dihydro-1H-isoindol-5-yl)carbamoyl]methyl}phenoxy)nonanoic acid* (**14i**; ZHAWOC6941): The title compound **14i** was obtained as a white solid in 39% yield and 96% purity: ^1^H-NMR (DMSO-*d*_6_): δ = 11.99 (br. s, 1H), 10.74 (s, 1H), 8.21 (d, *J* = 1.80 Hz, 1H), 7.89 (dd, *J* = 8.21 Hz, 1.80 Hz, 1H), 7.84 (d, *J* = 8.21 Hz, 1H), 7.35–7.21 (m, 7H), 6.89–6.85 (m, 2H), 4.73 (s, 2H), 3.92 (t, *J* = 6.47 Hz, 2H), 3.63 (s, 2H), 2.18 (t, *J* = 7.34 Hz, 2H), 1.72–1.64 (m, 2H), 1.52–1.45 (m, 2H); 1.42–1.35 (m, 2H), 1.33–1.22 (m, 6H) ppm. ^13^C-NMR (DMSO-*d*_6_): δ = 174.99, 170.94, 167.94, 167.77, 158.03, 145.31, 137.21, 133.54, 130.63, 129.04, 127.86, 127.83, 127.49, 125.71, 124.93, 123.81, 114.81, 113.33, 67.84, 42.96, 41.31, 34.20, 29.17, 29.13, 29.11, 28.98, 25.95, 24.98 ppm. HRMS-TOF (*m*/*z*): [M + H]^+^ calculated for C_32_H_34_N_2_O_6_: 542.2417, found: 542.2406.

*10-(4-{[(2-Benzyl-1,3-dioxo-2,3-dihydro-1H-isoindol-5-yl)carbamoyl]methyl}phenoxy)decanoic acid* (**14j**; ZHAWOC6942): The title compound **14j** was obtained as a white solid in 33% yield and 98% purity: ^1^H-NMR (DMSO-*d*_6_): δ = 12.01 (br. s, 1H), 10.76 (s, 1H), 8.21 (d, *J* = 1.86 Hz, 1H), 7.89 (dd, *J* = 8.15 Hz, 1.86 Hz, 1H), 7.84 (d, *J* = 8.15 Hz, 1H), 7.35–7.21 (m, 7H), 6.89–6.85 (m, 2H), 4.73 (s, 2H), 3.92 (t, *J* = 6.51 Hz, 2H), 3.63 (s, 2H), 2.17 (t, *J* = 7.51 Hz, 2H), 1.72–1.64 (m, 2H), 1.52–1.44 (m, 2H); 1.43–1.35 (m, 2H), 1.33–1.20 (m, 8H) ppm. ^13^C-NMR (DMSO-*d*_6_): δ = 170.95, 167.94, 167.77, 158.02, 145.32, 137.21, 133.54, 130.63, 129.04, 127.86, 127.82, 127.50, 125.71, 124.92, 123.81, 114.81, 113.33, 67.83, 42.96, 41.31, 34.29, 29.33, 29.18, 29.16, 29.13, 29.02, 25.96, 25.03 ppm. HRMS-TOF (*m*/*z*): [M + H]^+^ calculated for C_33_H_36_N_2_O_6_: 556.2573, found: 556.2568.

*3-{4-[({2-[(4-Fluorophenyl)methyl]-1,3-dioxo-2,3-dihydro-1H-isoindol-5-yl}carbamoyl)methyl]-phenoxy}propanoic acid* (**14k**; ZHAWOC6644): The title compound **14k** was obtained as a white solid in 62% yield and 97% purity: ^1^H-NMR (DMSO-*d*_6_): δ = 12.35 (br. s, 1H), 10.74 (s, 1H), 8.21 (d, *J* = 1.86 Hz, 1H), 7.89 (dd, *J* = 8.24 Hz, 1.86 Hz, 1H), 7.83 (d, *J* = 8.24 Hz, 1H), 7.37–7.31 (m, 2H), 7.26–7.22 (m, 2H), 7.17–7.12 (m, 2H), 6.91–6.86 (m, 2H), 4.72 (s, 2H), 4.14 (t, *J* = 6.10 Hz, 2H), 3.64 (s, 2H), 2.66 (t, *J* = 6.10 Hz, 2H) ppm. ^13^C-NMR (DMSO-*d*_6_): δ = 172.71, 170.90, 167.91, 167.73, 161.91 (d, *J* = 239.42 Hz, 1C), 157.71, 145.30, 133.55, 133.45 (d, *J* = 3.29 Hz, 1C), 130.69, 130.07 (d, *J* = 8.27 Hz, 2C), 127.81, 125.73, 124.93, 123.82, 115.82 (d, *J* = 21.45 Hz, 2C), 114.81, 113.34, 64.09, 42.93, 40.63, 34.66 ppm. HRMS-TOF (*m*/*z*): [M + H]^+^ calculated for C_26_H_21_FN_2_O_6_: 476.1384, found: 476.1391.

*4-{4-[({2-[(4-Fluorophenyl)methyl]-1,3-dioxo-2,3-dihydro-1H-isoindol-5-yl}carbamoyl)methyl]-phenoxy}butanoic acid* (**14l**; ZHAWOC5463): The title compound **14l** was obtained as a white solid in 97% yield and 97% purity: ^1^H-NMR (DMSO-*d*_6_): δ = 11.99 (br. s, 1H), 10.74 (s, 1H), 8.21 (d, *J* = 1.80 Hz, 1H), 7.89 (dd, *J* = 8.34 Hz, 1.80 Hz, 1H), 7.84 (d, *J* = 8.34 Hz, 1H), 7.37–7.32 (m, 2H), 7.26–7.22 (m, 2H), 7.17–7.12 (m, 2H), 6.91–6.86 (m, 2H), 4.72 (s, 2H), 3.96 (t, *J* = 6.39 Hz, 2H), 3.64 (s, 2H), 2.73 (t, *J* = 7.37 Hz, 2H), 1.92 (quint., *J* = 6.69 Hz, 2H) ppm. ^13^C-NMR (DMSO-*d*_6_): δ = 174.54, 170.91, 167.90, 167.72, 161.91 (d, *J* = 243.34 Hz, 1C), 157.85, 145.30, 133.54, 133.44 (d, *J* = 3.06 Hz, 1C), 130.66, 130.07 (d, *J* = 8.32 Hz, 2C), 127.66, 125.71, 124.92, 123.81, 115.81 (d, *J* = 21.45 Hz, 2C), 114.83, 113.33, 67.00, 42.94, 40.62, 30.57, 24.70 ppm. HRMS-TOF (*m*/*z*): [M + H]^+^ calculated for C_27_H_23_FN_2_O_6_: 490.1540, found: 490.1538.

*5-{4-[({2-[(4-Fluorophenyl)methyl]-1,3-dioxo-2,3-dihydro-1H-isoindol-5-yl}carbamoyl)methyl]-phenoxy}pentanoic acid* (**14m**; ZHAWOC5684): The title compound **14m** was obtained as a white solid in 50% yield and 96% purity: ^1^H-NMR (DMSO-*d*_6_): δ = 11.92 (br. s, 1H), 10.74 (s, 1H), 8.20 (d, *J* = 1.82 Hz, 1H), 7.89 (dd, *J* = 8.25 Hz, 1.82 Hz, 1H), 7.83 (d, *J* = 8.25 Hz, 1H), 7.37–7.31 (m, 2H), 7.26–7.21 (m, 2H), 7.17–7.11 (m, 2H), 6.90–6.86 (m, 2H), 4.72 (s, 2H), 3.94 (t, *J* = 6.16 Hz, 2H), 3.63 (s, 2H), 2.27 (t, *J* = 6.41 Hz, 2H), 1.75–1.67 (m, 2H), 1.66–1.59 (m, 2H) ppm. ^13^C-NMR (DMSO-*d*_6_): δ = 174.83, 170.93, 167.90, 167.72, 161.90 (d, *J* = 243.25 Hz, 1C), 157.97, 145.30, 133.54, 133.44 (d, *J* = 3.03 Hz, 1C), 130.64, 130.07 (d, *J* = 8.24 Hz, 2C), 127.55, 125.71, 124.93, 123.81, 115.81 (d, *J* = 21.45 Hz, 2C), 114.81, 113.33, 67.54, 42.95, 40.63, 33.76, 28.59, 21.69 ppm. HRMS-TOF (*m*/*z*): [M + H]^+^ calculated for C_28_H_25_FN_2_O_6_: 505.1776, found: 505.1758.

*6-{4-[({2-[(4-Fluorophenyl)methyl]-1,3-dioxo-2,3-dihydro-1H-isoindol-5-yl}carbamoyl)methyl]-phenoxy}hexanoic acid* (**14n**; ZHAWOC6645): The title compound **14n** was obtained as a white solid in 59% yield and 97% purity: ^1^H-NMR (DMSO-*d*_6_): δ = 11.98 (br. s, 1H), 10.73 (s, 1H), 8.20 (d, *J* = 1.82 Hz, 1H), 7.89 (dd, *J* = 8.23 Hz, 1.82 Hz, 1H), 7.83 (d, *J* = 8.23 Hz, 1H), 7.37–7.31 (m, 2H), 7.26–7.21 (m, 2H), 7.17–7.11 (m, 2H), 6.90–6.86 (m, 2H), 4.72 (s, 2H), 3.92 (t, *J* = 6.45 Hz, 2H), 3.63 (s, 2H), 2.22 (t, *J* = 7.29 Hz, 2H), 1.73–1.65 (m, 2H), 1.59–1.51 (m, 2H), 1.45–1.37 (m, 2H) ppm. ^13^C-NMR (DMSO-*d*_6_): δ = 174.88, 170.93, 167.90, 167.73, 161.90 (d, *J* = 243.07 Hz, 1C), 158.01, 145.30, 133.55, 133.45 (d, *J* = 3.11 Hz, 1C), 130.64, 130.07 (d, *J* = 8.24 Hz, 2C), 127.52, 125.72, 124.93, 123.81, 115.82 (d, *J* = 21.46 Hz, 2C), 114.81, 113.33, 67.75, 42.95, 40.63, 34.09, 28.91, 25.63, 24.74 ppm. HRMS-TOF (*m*/*z*): [M + H]^+^ calculated for C_29_H_27_FN_2_O_6_: 518.1853, found: 518.1840.

*7-{4-[({2-[(4-Fluorophenyl)methyl]-1,3-dioxo-2,3-dihydro-1H-isoindol-5-yl}carbamoyl)methyl]-phenoxy}heptanoic acid* (**14o**; ZHAWOC6637): The title compound **14o** was obtained as a white solid in 56% yield and 99% purity: ^1^H-NMR (DMSO-*d*_6_): δ = 12.03 (br. s, 1H), 10.74 (s, 1H), 8.20 (d, *J* = 1.85 Hz, 1H), 7.89 (dd, *J* = 8.24 Hz, 1.85 Hz, 1H), 7.83 (d, *J* = 8.24 Hz, 1H), 7.37–7.31 (m, 2H), 7.26–7.21 (m, 2H), 7.17–7.11 (m, 2H), 6.90–6.86 (m, 2H), 4.72 (s, 2H), 3.92 (t, *J* = 6.47 Hz, 2H), 3.63 (s, 2H), 2.19 (t, *J* = 7.51 Hz, 2H), 1.72–1.64 (m, 2H), 1.54–1.47 (m, 2H), 1.43–1.36 (m, 2H), 1.35–1.28 (m, 2H) ppm. ^13^C-NMR (DMSO-*d*_6_): δ = 174.98, 170.94, 167.91, 167.73, 161.90 (d, *J* = 243.56 Hz, 1C), 158.03, 145.31, 133.55, 133.46 (d, *J* = 2.14 Hz, 1C), 130.64, 130.08 (d, *J* = 8.27 Hz, 2C), 127.51, 125.72, 124.93, 123.81, 115.82 (d, *J* = 21.52 Hz, 2C), 114.81, 113.34, 67.78, 42.95, 40.63, 34.20, 29.02, 28.78, 25.73, 24.96 ppm. HRMS-TOF (*m*/*z*): [M + H]^+^ calculated for C_30_H_29_FN_2_O_6_: 532.2010, found: 532.2013.

*8-{4-[({2-[(4-Fluorophenyl)methyl]-1,3-dioxo-2,3-dihydro-1H-isoindol-5-yl}carbamoyl)methyl]-phenoxy}octanoic acid* (**14p**; ZHAWOC6646): The title compound **14p** was obtained as a white solid in 30% yield and 99% purity: ^1^H-NMR (DMSO-*d*_6_): δ = 12.00 (br. s, 1H), 10.74 (s, 1H), 8.20 (d, *J* = 1.87 Hz, 1H), 7.89 (dd, *J* = 8.27 Hz, 1.87 Hz, 1H), 7.83 (d, *J* = 8.27 Hz, 1H), 7.37–7.31 (m, 2H), 7.26–7.21 (m, 2H), 7.17–7.11 (m, 2H), 6.90–6.86 (m, 2H), 4.72 (s, 2H), 3.92 (t, *J* = 6.47 Hz, 2H), 3.63 (s, 2H), 2.19 (t, *J* = 7.39 Hz, 2H), 1.71–1.64 (m, 2H), 1.53–1.45 (m, 2H), 1.42–1.35 (m, 2H), 1.34–1.24 (m, 4H) ppm. ^13^C-NMR (DMSO-*d*_6_): δ = 174.98, 170.94, 167.90, 167.73, 161.90 (d, *J* = 242.92 Hz, 1C), 158.03, 145.31, 133.55, 133.45 (d, *J* = 3.11 Hz, 1C), 130.63, 130.07 (d, *J* = 8.32 Hz, 2C), 127.49, 125.71, 124.93, 123.81, 115.82 (d, *J* = 21.55 Hz, 2C), 114.81, 113.33, 67.82, 42.95, 40.62, 34.20, 29.09, 28.98, 28.94, 25.86, 24.94 ppm. HRMS-TOF (*m*/*z*): [M + H]^+^ calculated for C_31_H_31_FN_2_O_6_: 546.2166, found: 546.2169.

### 4.3. In Vitro Assays

Single dose duplicate assays for the determination of the remaining enzymatic activity at 10 μM inhibitor concentration as well as IC_50_ values, using 10 concentrations starting at 10 μM with 3 fold dilution, for MMP-7 and MMP-13 were determined at Reaction Biology Corporation (Malvern, PA, USA). The substrate used for the determinations was the (5-FAM/QXL^TM^) FRET peptide (sequence = QXL^®^ 520-γ-Abu-Pro-Cha-Ab-Sm-Hi-Al-Dab(5-FAM)-Ala-Lys-NH_2_ (Abu = 2-aminobutyric acid, Cha = β-cyclohexylalanine, Dab = diaminobutyric acid, Smc = S-methyl-L–cysteine, QXL^®^ 520 = quencher, 5-FAM = fluorescence dye); supplier AnaSpec Inc., Fremont, CA, USA, product code: AS-60581-01). The buffer consisted of 50 mM HEPES at pH 7.5 with 10 mM CaCl_2_ and 0.01% Brij-35. 0.1 mg/mL BSA (Sigma Aldrich, St. Louis, MO, USA) was added before use. As a control inhibitor GM6001 (ilomastat, Enzo Life Sciences, Farmingdale, NY, USA) was used. For selectivity examinations single dose duplicate assays were performed in the same manner as described above on the MMP-1, -2, -3, -8, -9, -12 and -14 isoforms.

### 4.4. In Silico Studies

Molecular modeling experiments were performed using the Molecular Operating Environment MOE 2015.10 from Chemical Computing Group (Montreal, QC, Canada). Co-crystal structures of MMP-7 are available from the Protein Data Bank. For the actual work PDB code: 2Y6D was selected for the computational studies. In MOE the pocket was prepared for the dockings via the Protonate 3D method applying the default values for temperature 300 K, pH 7 and salt 0.1. The ligands to be docked to the protein were imported from SD files to receive a MOE compatible molecular database. As the SD files did not contain 3D coordinates, they were generated directly using MOE rebuild3D with an RMSD gradient of 0.1. For docking experiments the Amber10:EHT force field [46,47] was used. The triangle matcher placement was applied with a rigid receptor. The docked poses were subsequently analyzed with respect to their scores and interactions with the target enzyme.

## 5. Conclusions

A highly potent and selective MMP-13 inhibitor was modified to obtain a dual MMP-7/-13 inhibitor with selectivity over a variety of MMP isoforms. We were able to modify the original molecule with a focus on gaining potency against MMP-7 while decreasing its potency against MMP-13. The IC_50_-value against MMP-7 could be improved from 15.7 μM to 2.2 μM by removing the fluorine atom from the benzylic ring and by elongating the aliphatic linker between the phenolic oxygen and the carboxylic acid head group from 4 to 8 CH_2_ entities. Further improvements with respect to the inhibitor’s potency are imaginable by rigidification of the flexible aliphatic linker to yield beneficial entropy terms for the ligand-enzyme complex, for example by the incorporation of non-saturated fragments such as alkenes or alkynes. The improvements towards MMP-7 inhibition decreased the potency on MMP-13 drastically from 6 nM to 1.2 μM resulting in a dual inhibitor in the low micromolar range equally potent against MMP-7 and MMP-13. To our knowledge, this inhibitor is the first of its kind that simultaneously inhibits the two validated drug targets MMP-7 and MMP-13 selectively. This is of utmost interest in polypharmacology, because here two or more targets are addressed at the same time to tackle one disease [48,49].

## Figures and Tables

**Table 1:** Selectivity profile of **5** against a variety of MMPs ^1^ [43].

MMP-1	MMP-2	MMP-3	MMP-7	MMP-8	MMP-9	MMP-12	MMP-14
100%	100%	100%	68%	97%	100%	91%	100%

^1^ Remaining enzymatic activity at 10 µM inhibitor concentration.

**Table 2:** Inhibitory data for MMP-7 and MMP-13.

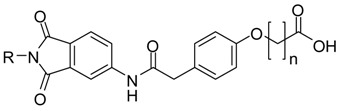						
Inhibitor	R	n	% Activity MMP-7 ^1^	% Activity MMP-13 ^1^	IC_50_ MMP-7 (μM) ^2^	IC_50_ MMP-13 (μM) ^2^
**14a ZHAWOC6649**	CH_3_	4	100.2 ± 4.4	23.7 ± 3.3	n.d. ^3^	n.d.
**14b ZHAWOC5474**	benzyl	1	89.9 ± 10.0	58.7 ± 2.7	n.d.	n.d.
**14c ZHAWOC4765**	benzyl	2	93.1 ± 1.7	35.8 ± 5.1	n.d.	2.244 ± 0.200
**14d ZHAWOC4766**	benzyl	3	91.3 ± 0.4	2.6 ± 1.3	n.d.	0.556 ± 0.125
**14e ZHAWOC5131**	benzyl	4	93.2 ± 3.3	1.0 ± 0.2	n.d.	0.035 ± 0.001
**14f ZHAWOC5133**	benzyl	5	59.4 ± 0.2	2.5 ± 0.1	n.d.	0.151 ± 0.031
**14g ZHAWOC6650**	benzyl	6	53.8 ± 0.8	1.0 ± 0.1	n.d.	0.150 ± 0.031
**14h ZHAWOC6651**	benzyl	7	25.9 ± 0.5	16.0 ± 0.14	3.179 ± 0.249	1.670 ± 0.429
**14i ZHAWOC6941**	benzyl	8	n.d.	n.d.	2.248 ± 0.204	1.249 ± 0.282
**14j ZHAWOC6942**	benzyl	9	n.d.	n.d.	6.204 ± 1.853	7.117 ± 0.940
**14k ZHAWOC6644**	4F-benzyl	2	98.0 ± 7.3	37.0 ± 0.2	n.d.	n.d.
**14l ZHAWOC5463**	4F-benzyl	3	85.1 ± 2.2	3.6 ± 1.1	n.d.	0.361 ± 0.052
**5 ZHAWOC5684**	4F-benzyl	4	67.5 ± 2.6	n.d.	15.650 ± 1.520	0.006 ± 0.003
**14m ZHAWOC6645**	4F-benzyl	5	69.2 ± 0.1	1.2 ± 0.1	n.d.	n.d.
**14n ZHAWOC6637**	4F-benzyl	6	47.5 ± 5.1	1.9 ± 0.5	n.d.	n.d.
**14o ZHAWOC6646**	4F-benzyl	7	64.5 ± 3.7	34.6 ± 2.3	n.d.	n.d.

^1^ Remaining enzyme activity at 10 μM inhibitor concentration, measured in duplicate; ^2^ Measured in duplicate. ^3^ Not determined.

**Table 3:** Selectivity profile of **14i** against a variety of MMPs ^1^.

MMP-1	MMP-2	MMP-3	MMP-8	MMP-9	MMP-12	MMP-14
100%	98%	73%	80%	87%	97%	91%

^1^ Remaining enzymatic activity at 10 µM inhibitor concentration.

## Supplementary Materials

Supplementary materials related to this article, including complete analytical data of the synthesized compounds and IC_50_-curves, are available online.
