# Supplementary material for: Development of a Non-Hydroxamate Dual Matrix Metalloproteinase (MMP)-7/-13 Inhibitor
Source: Molecules. 2017 Sep 14;22(9):1548. doi: 10.3390/molecules22091548 (PMC6151531; doi:10.3390/molecules22091548)

Supplementary Materials:

Development of a Non-Hydroxamate Dual MMP-7/-13 Inhibitor

Thomas Fischer and Rainer Riedl*

Institute of Chemistry and Biotechnology

Center for Organic and Medicinal Chemistry

Zurich University of Applied Sciences ZHAW

Einsiedlerstrasse 31, 8820 Wädenswil, Switzerland

E-mail: rainer.riedl@zhaw.ch

5-amino-2-methyl-2,3-dihydro-1H-isoindole-1,3-dione (**11a**; ZHAWOC3444)

NMR

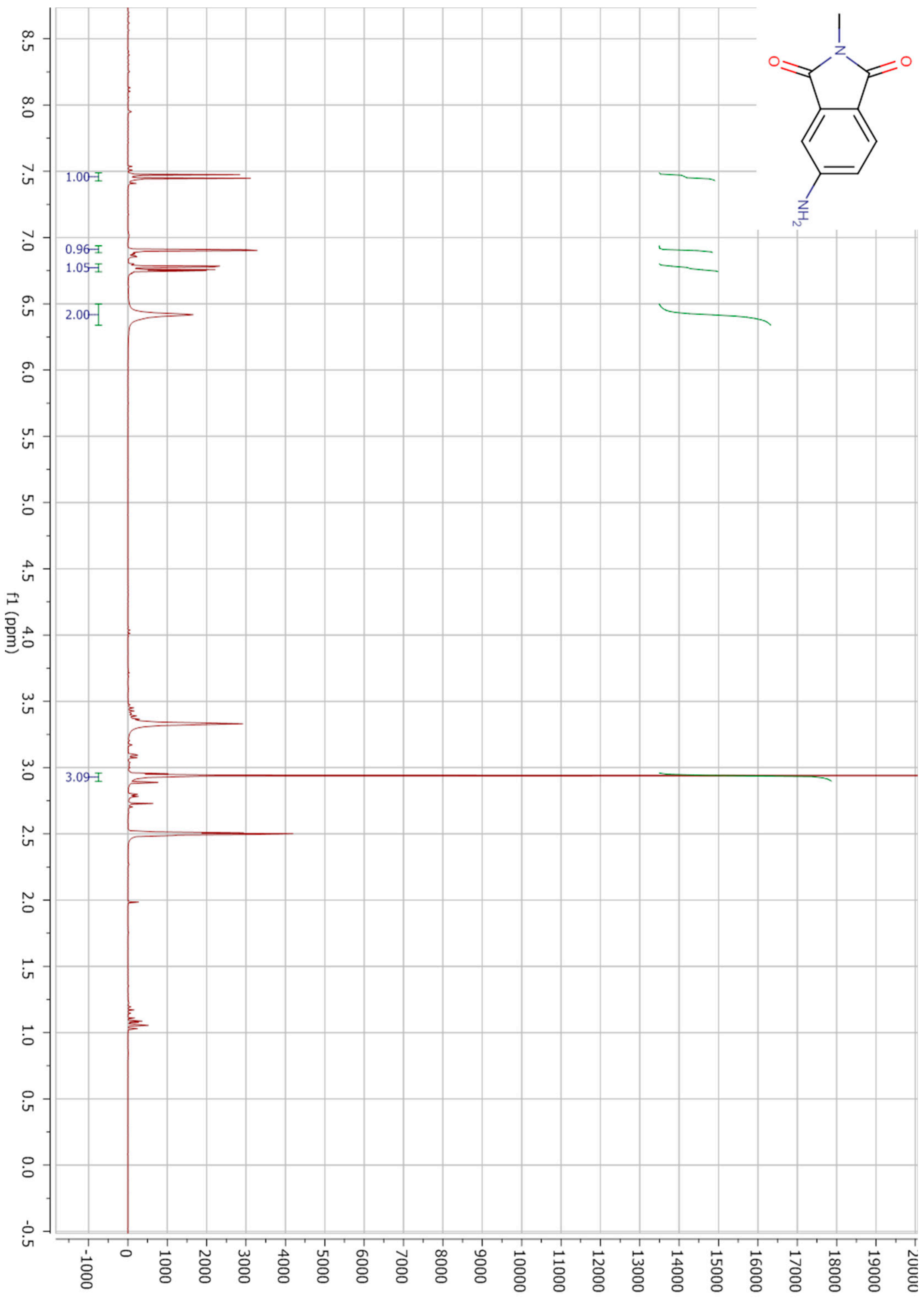

5-amino-2-methyl-2,3-dihydro-1H-isoindole-1,3-dione (**11a**; ZHAWOC3444)

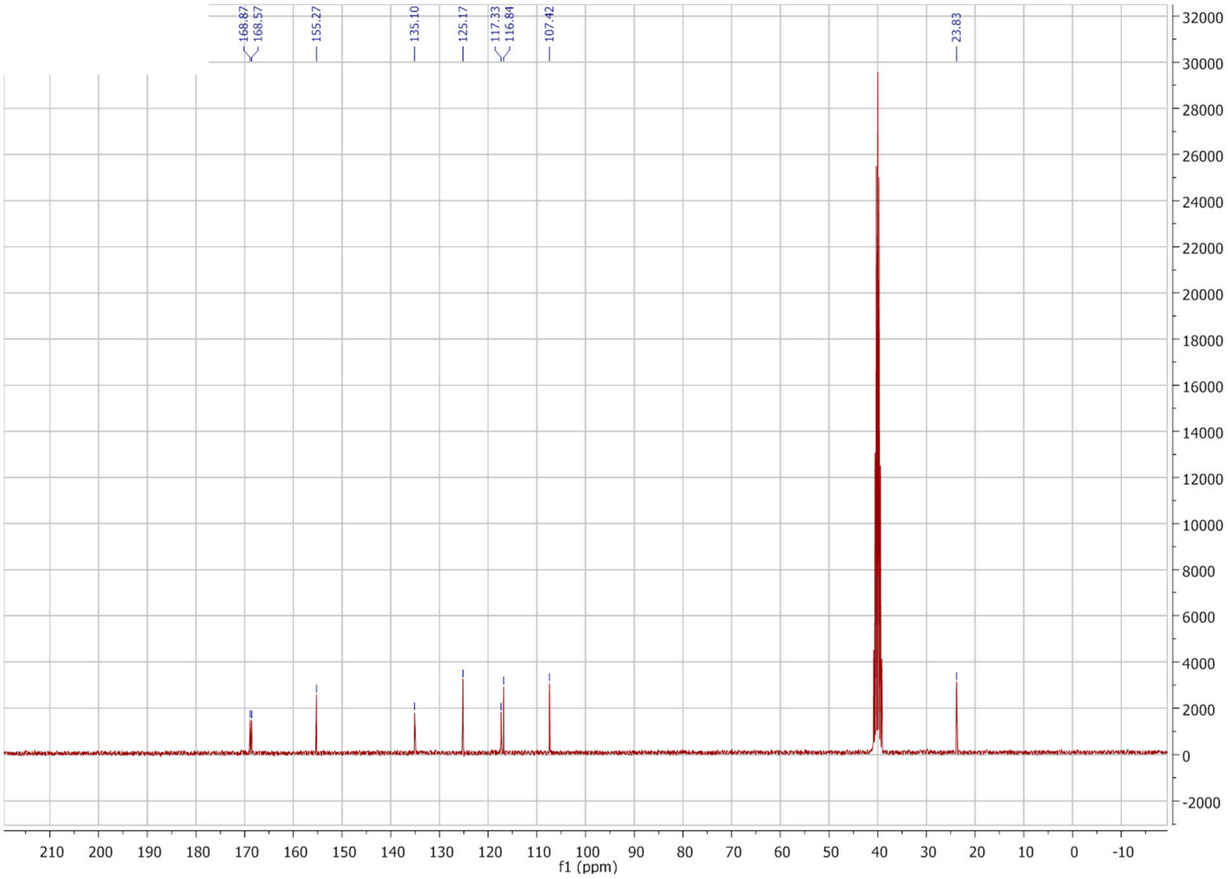

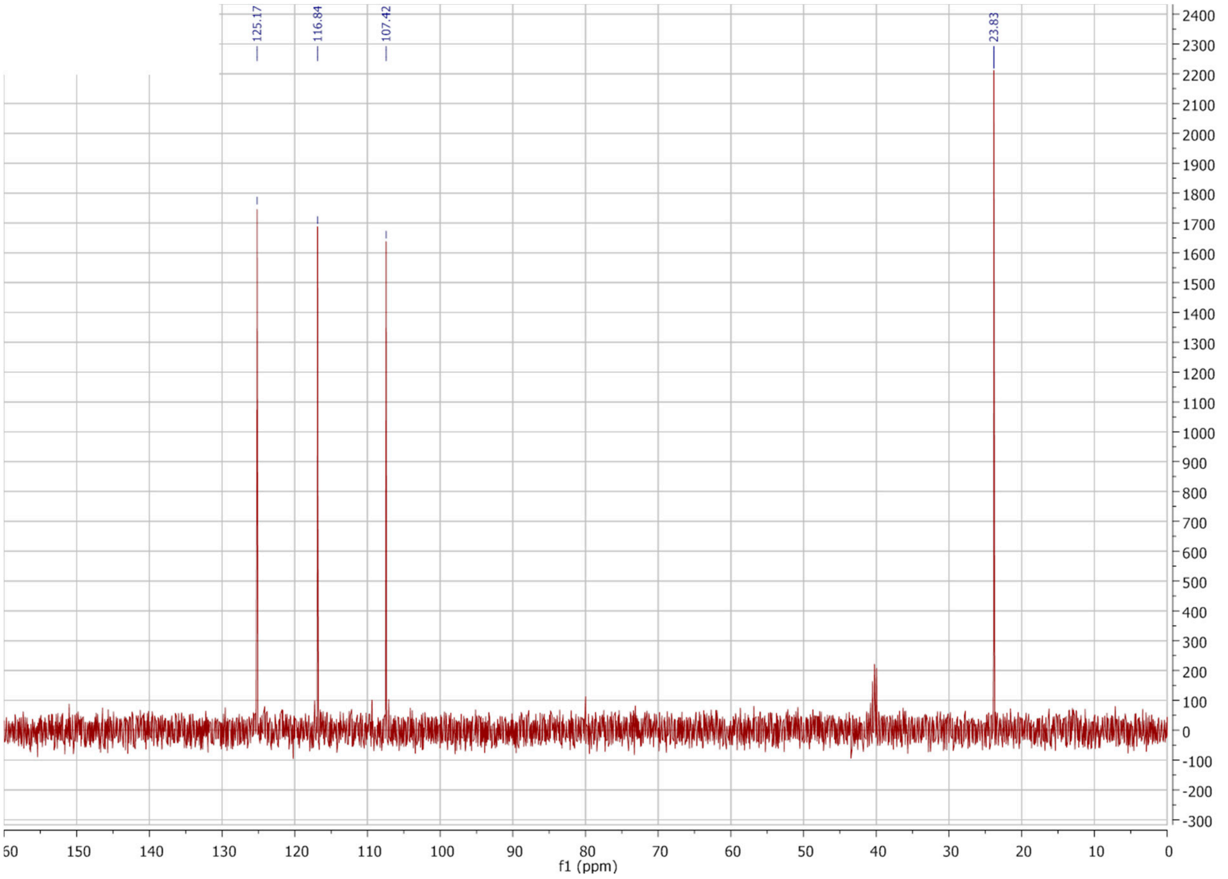

5-amino-2-benzyl-2,3-dihydro-1H-isoindole-1,3-dione (**11b;** ZHAWOC899)

<u>NMR</u>

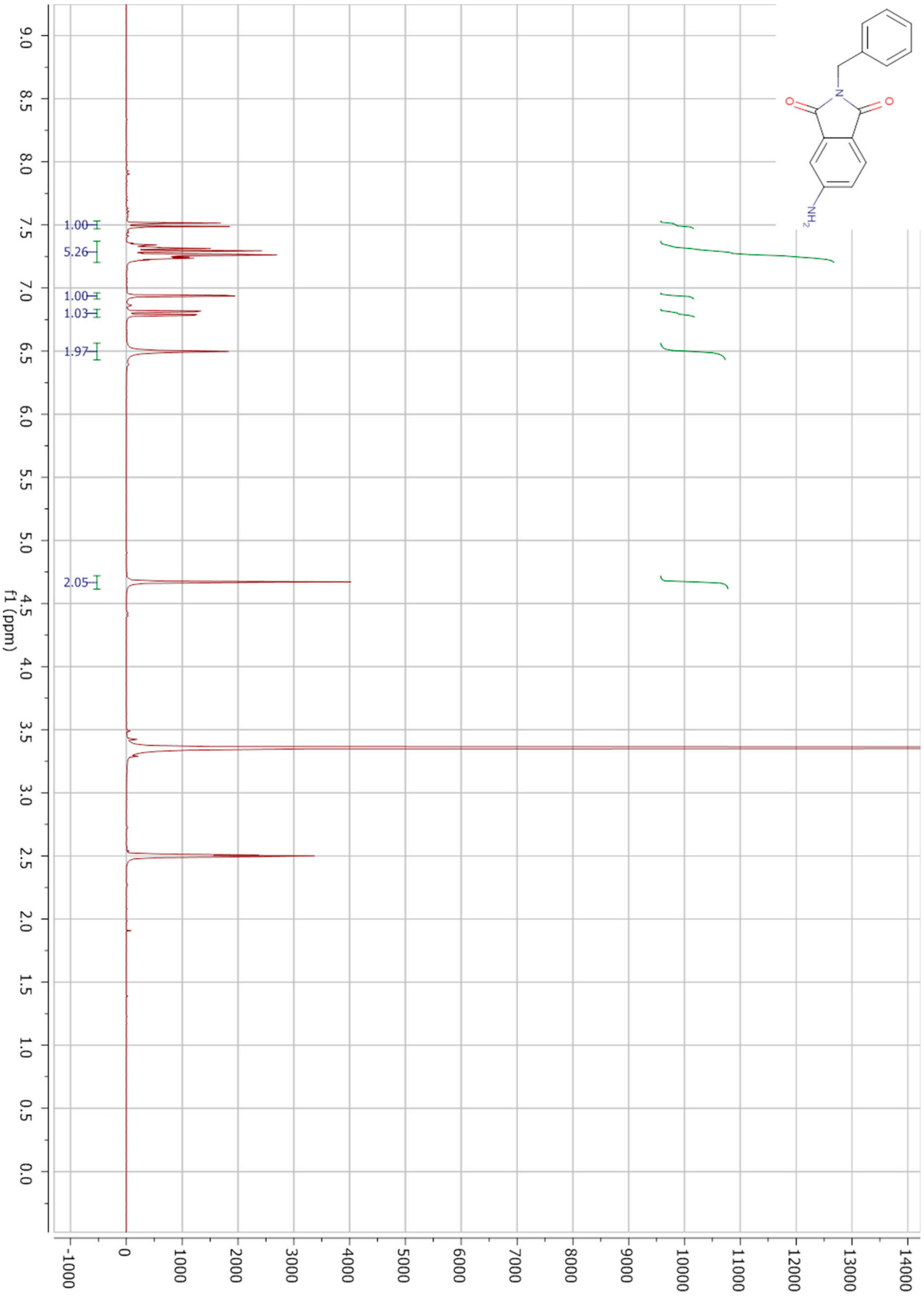

5-amino-2-benzyl-2,3-dihydro-1H-isoindole-1,3-dione (**11b;** ZHAWOC899)

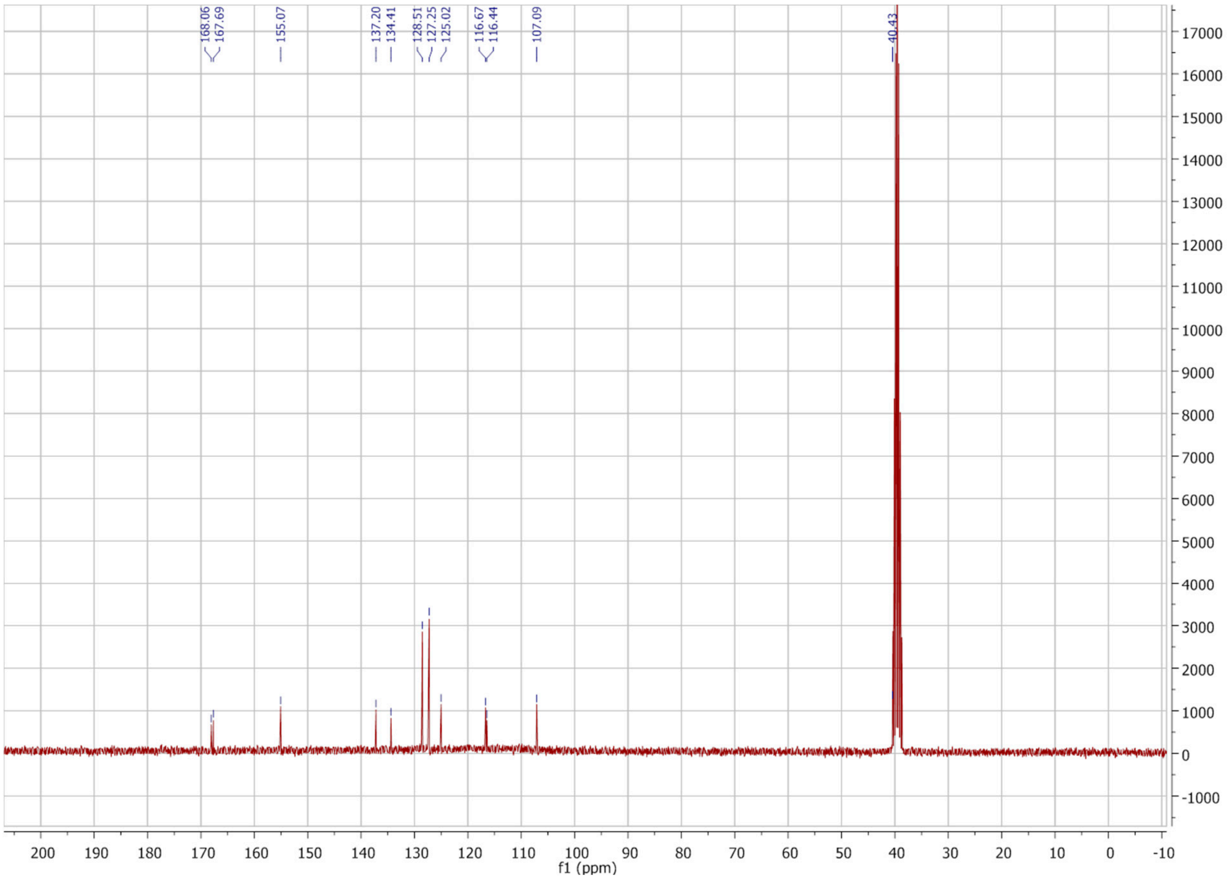

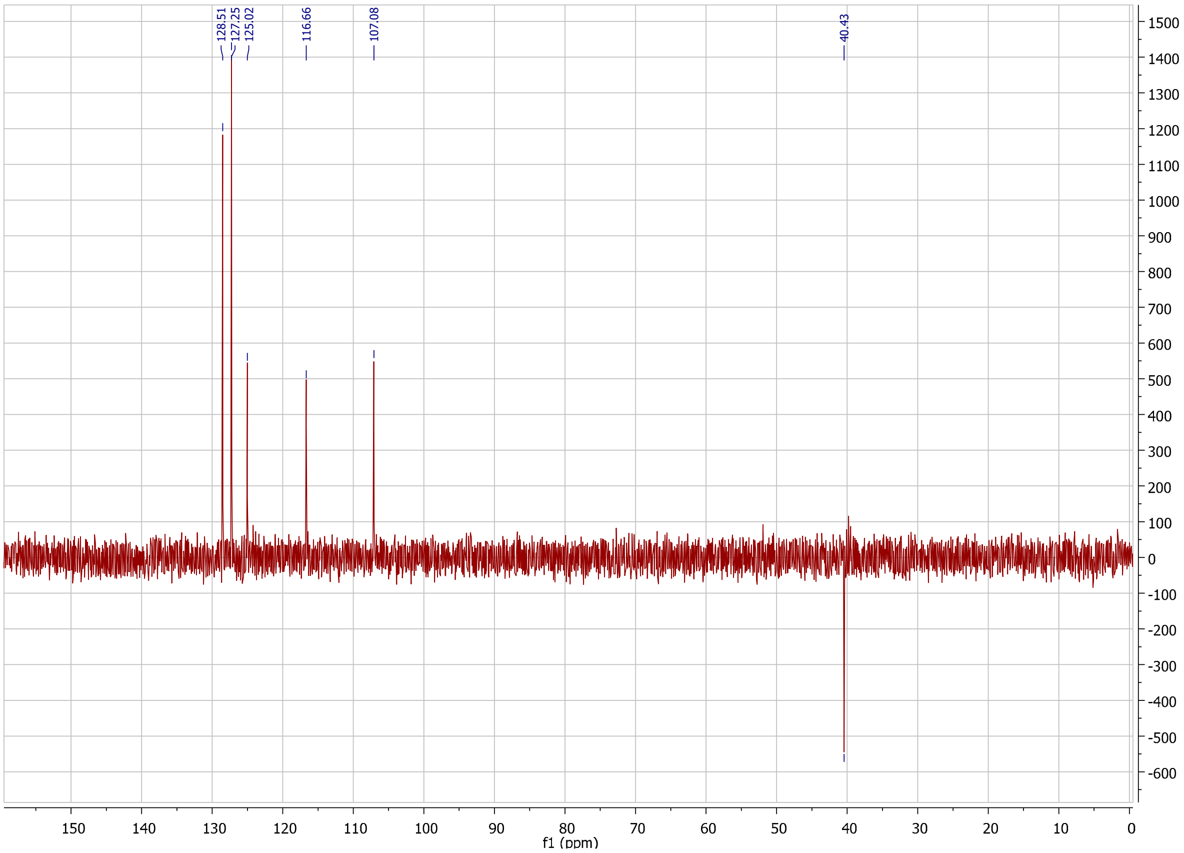

5-amino-2-[(4-fluorophenyl)methyl]-2,3-dihydro-1H-isoindole-1,3-dione (**11c**; ZHAWOC3199)

<u>NMR</u>

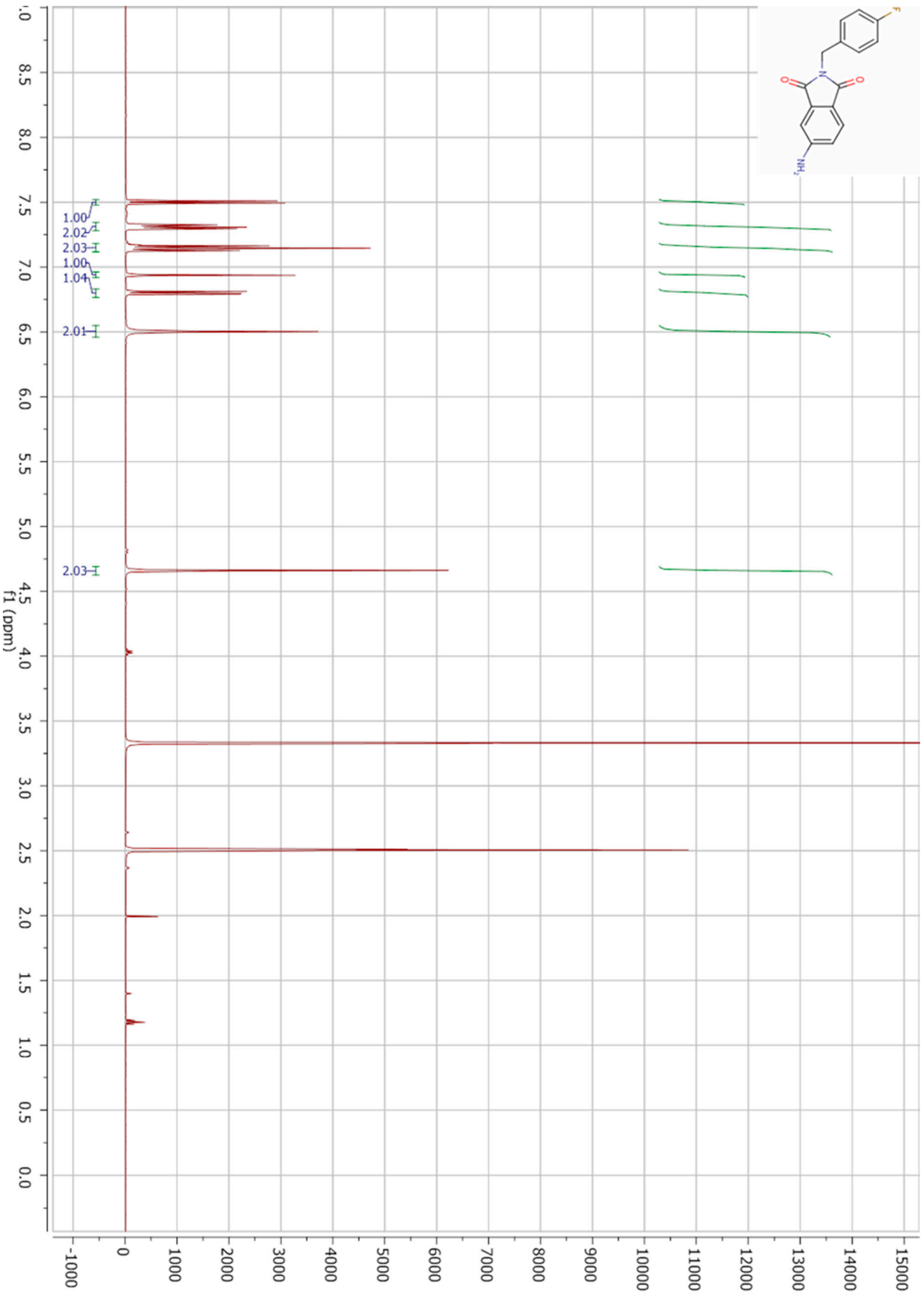

5-amino-2-[(4-fluorophenyl)methyl]-2,3-dihydro-1H-isoindole-1,3-dione (**11c**; ZHAWOC3199)

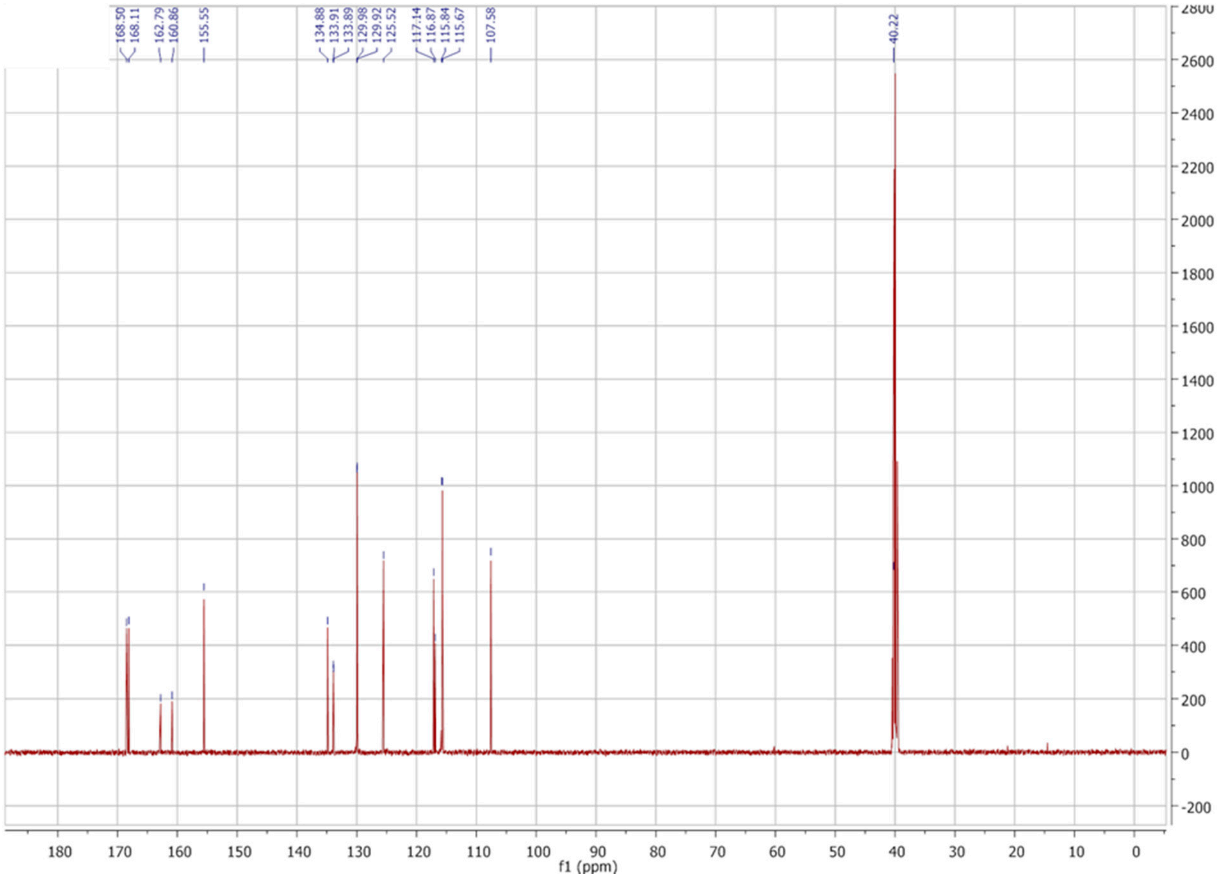

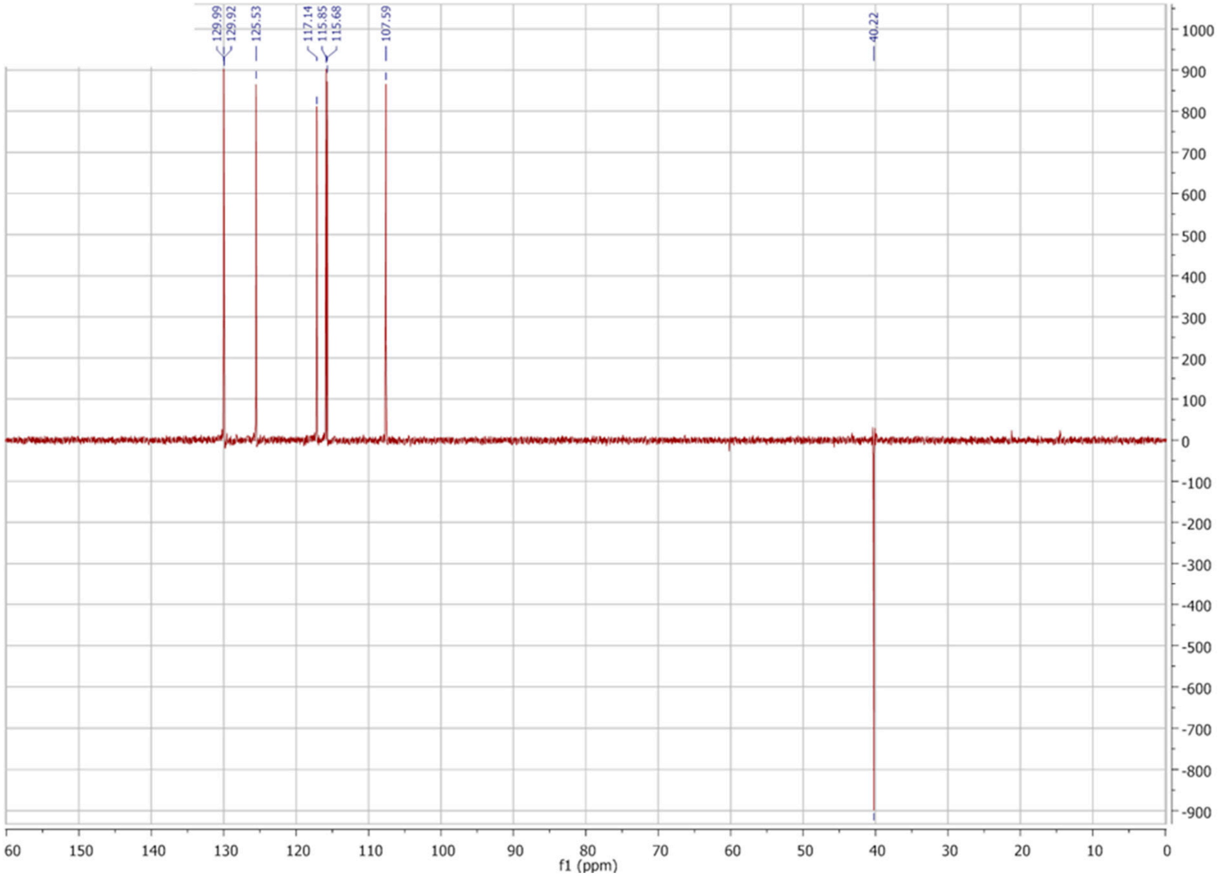

{[(7-bromoheptyl)oxy]methyl}benzene (**7a**; ZHAWOC7096)

<u>NMR</u>

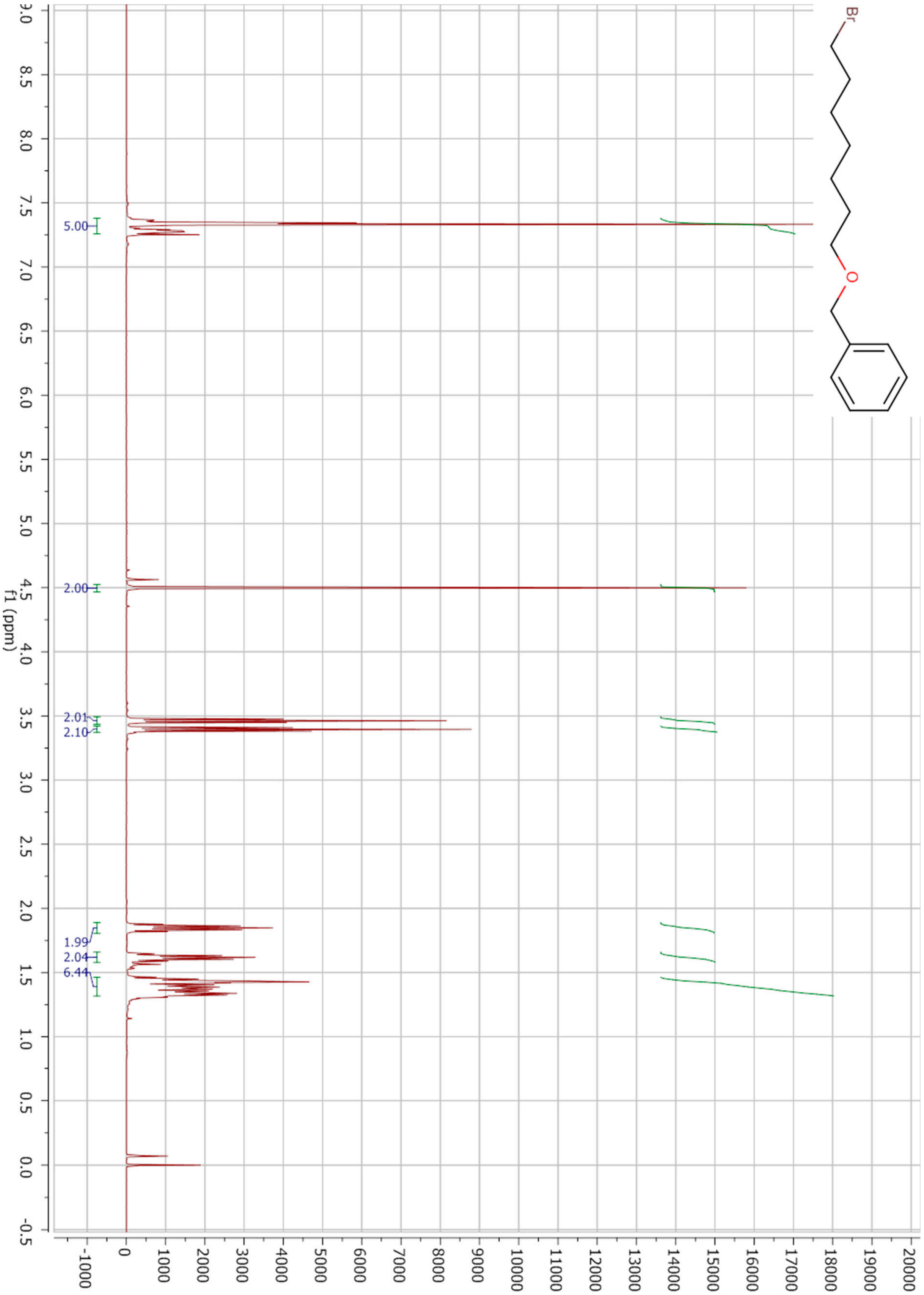

{[(7-bromoheptyl)oxy]methyl}benzene (**7a**; ZHAWOC7096)

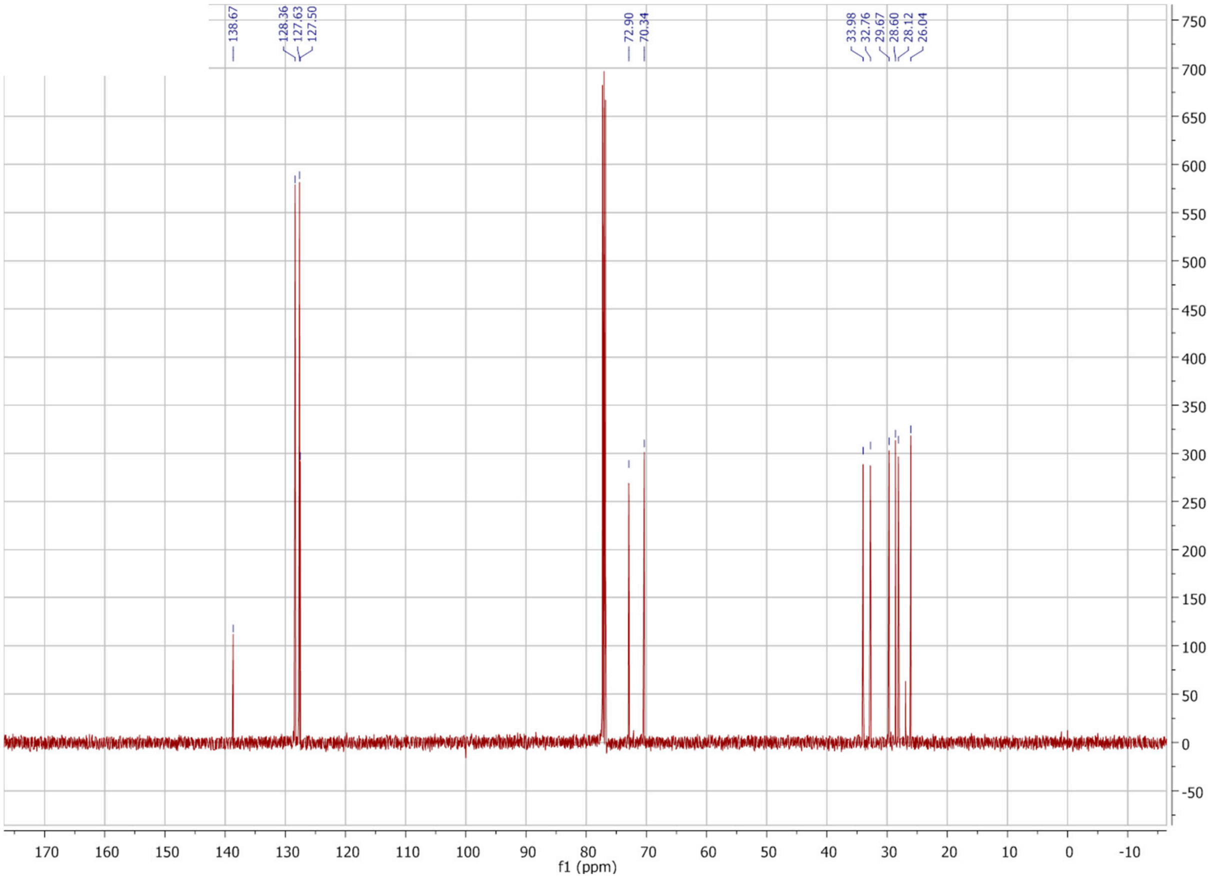

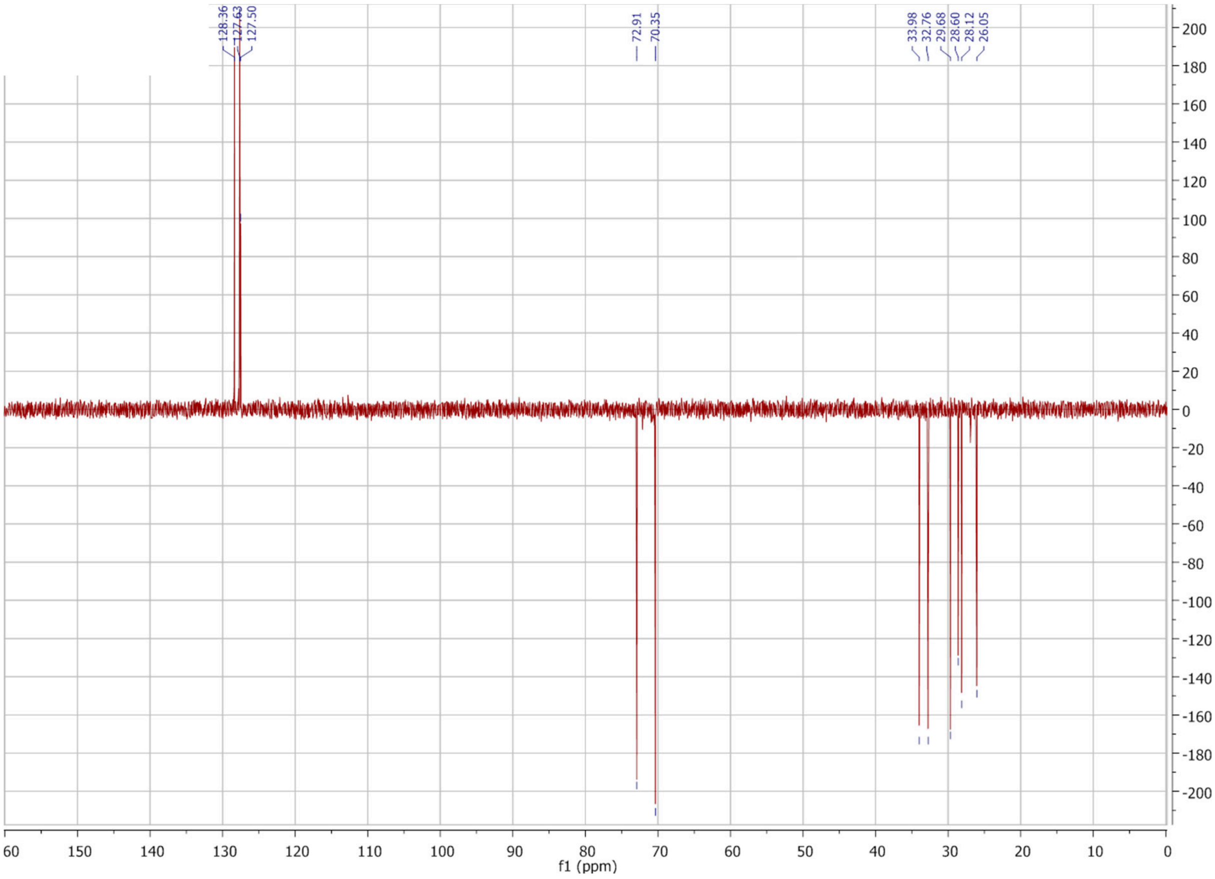

{[(8-bromooctyl)oxy]methyl}benzene (**7b**; ZHAWOC6856)

<u>NMR</u>

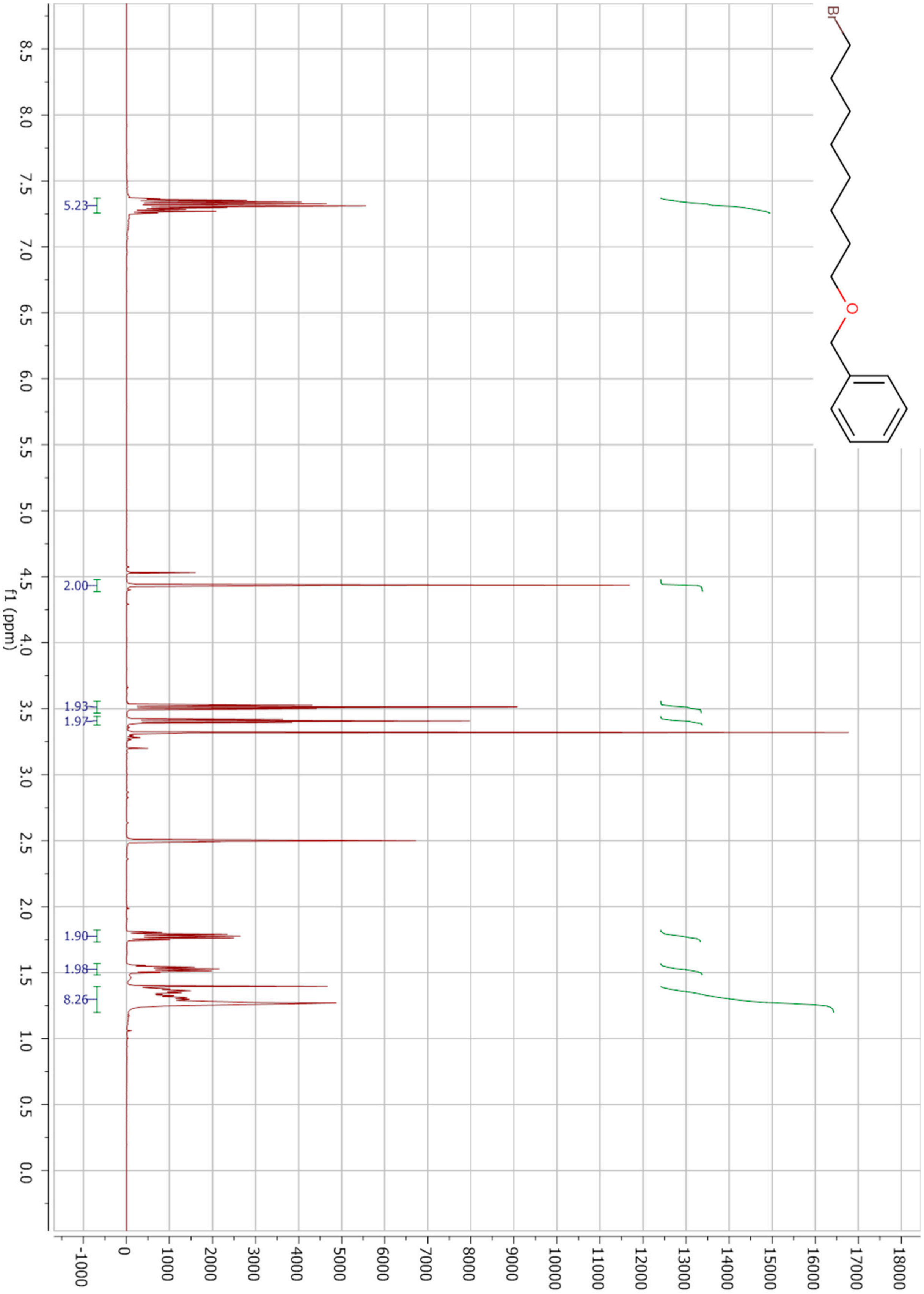

{[(8-bromooctyl)oxy]methyl}benzene (**7b**; ZHAWOC6856)

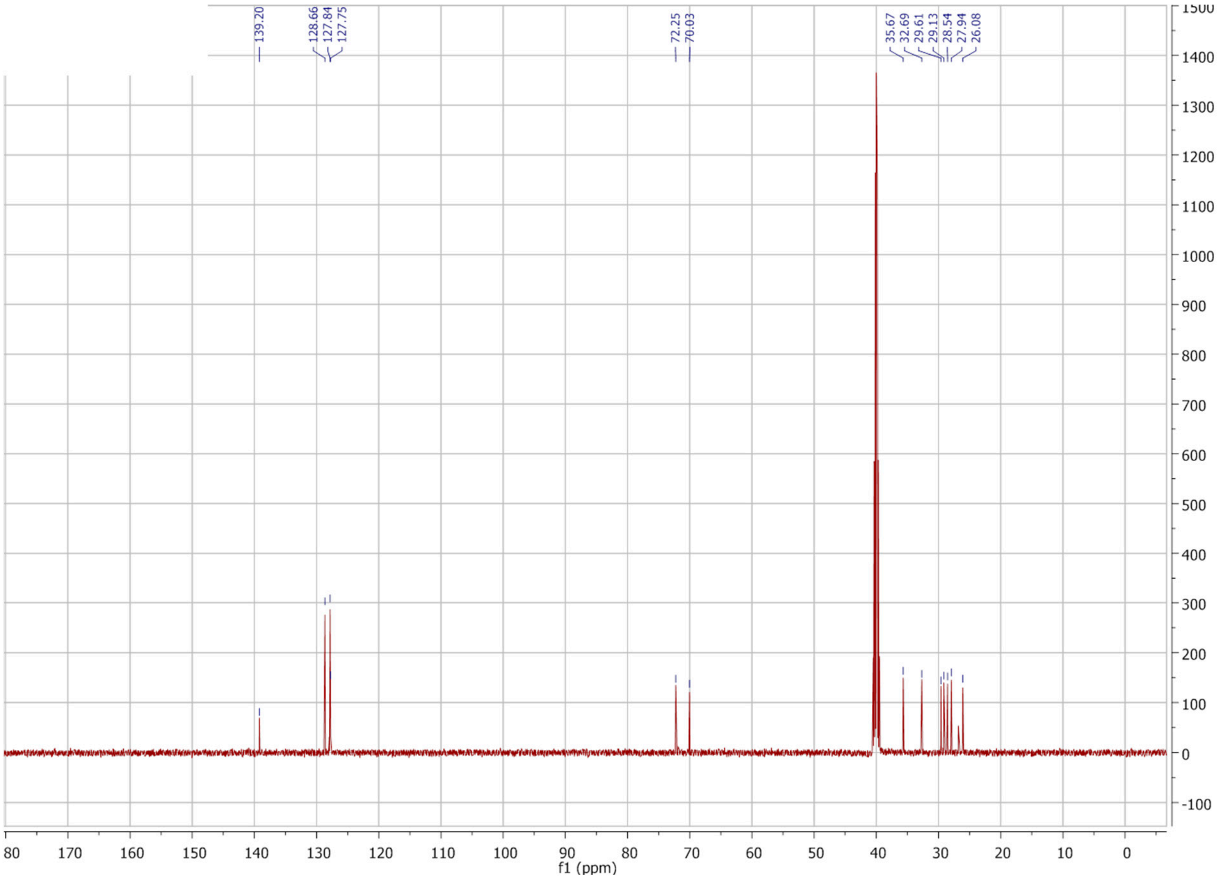

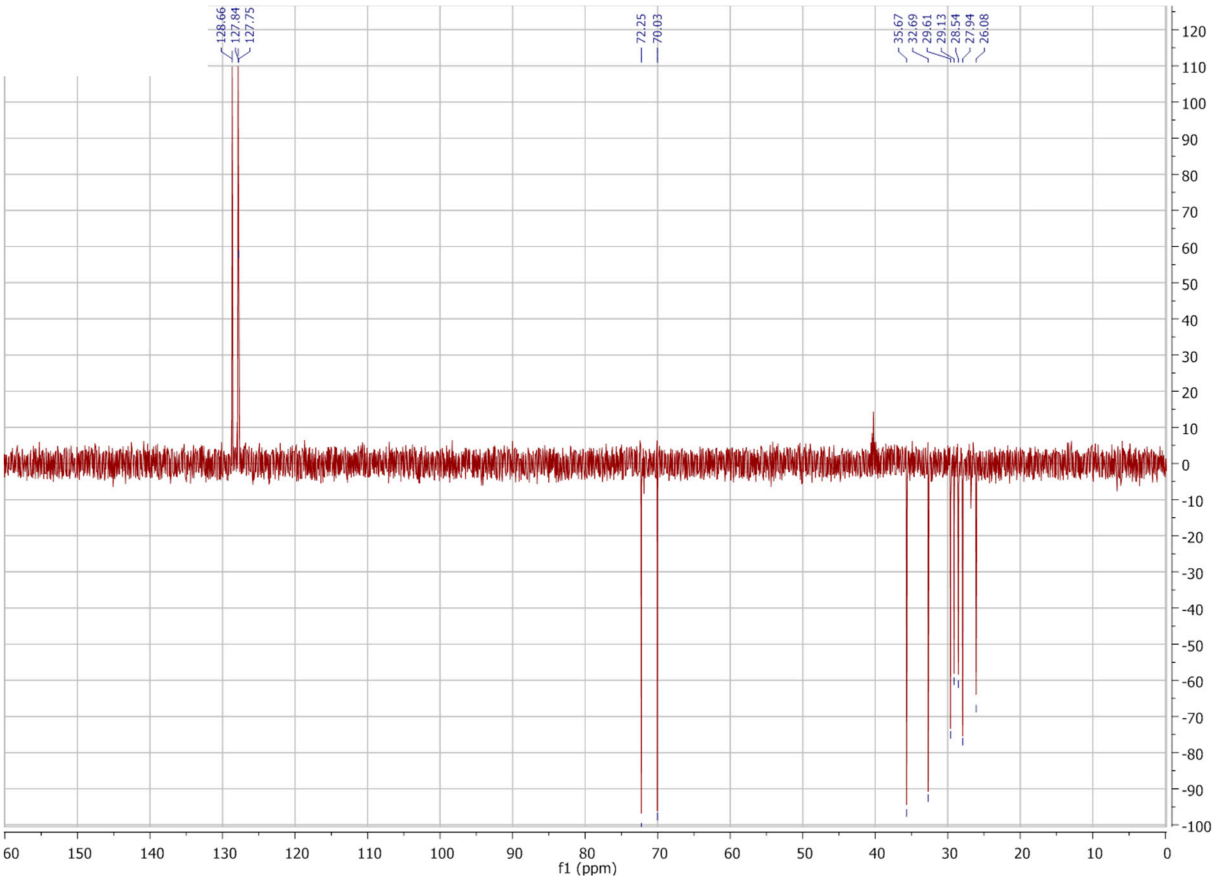

{[(9-bromononyl)oxy]methyl}benzene (**7c**; ZHAWOC6852)

<u>NMR</u>

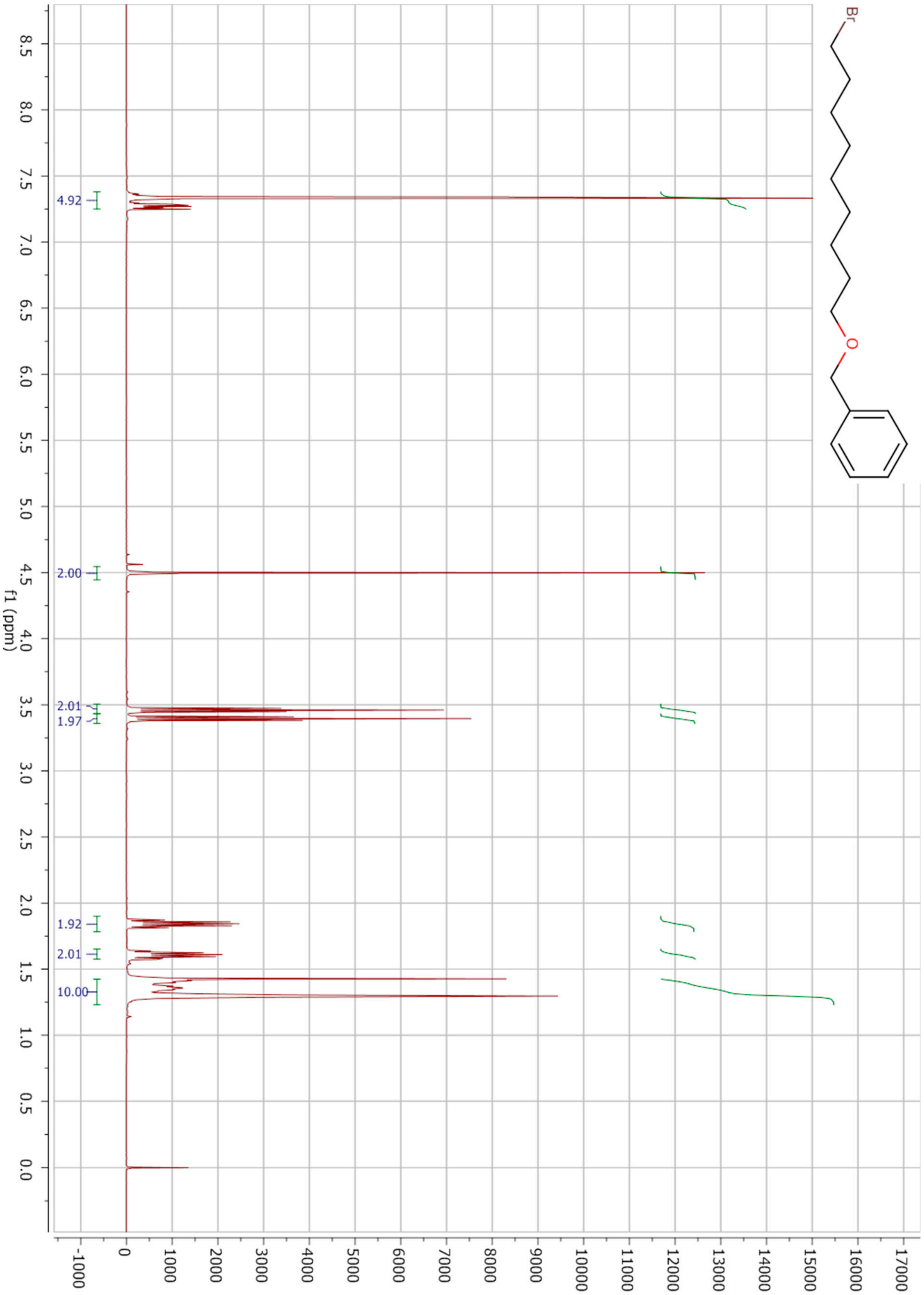

{[(9-bromononyl)oxy]methyl}benzene (**7c**; ZHAWOC6852)

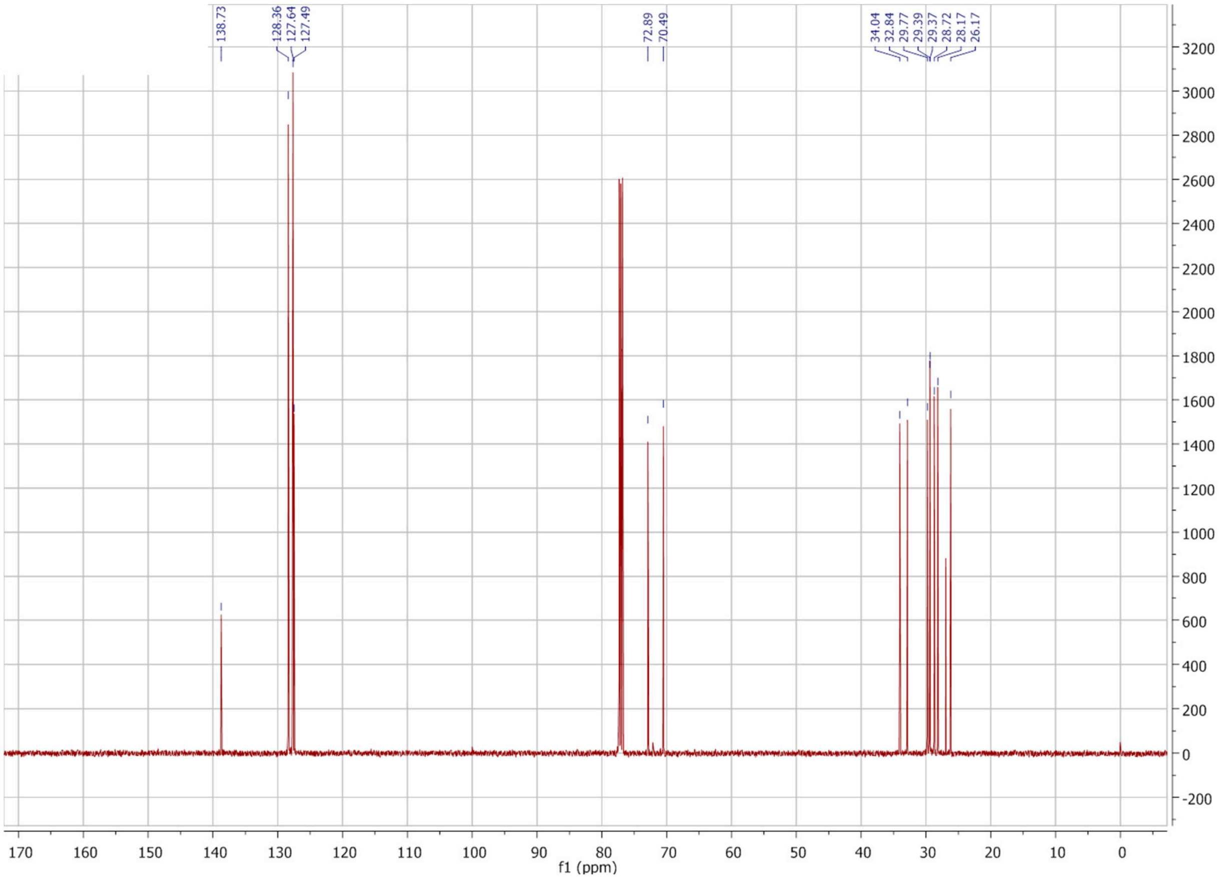

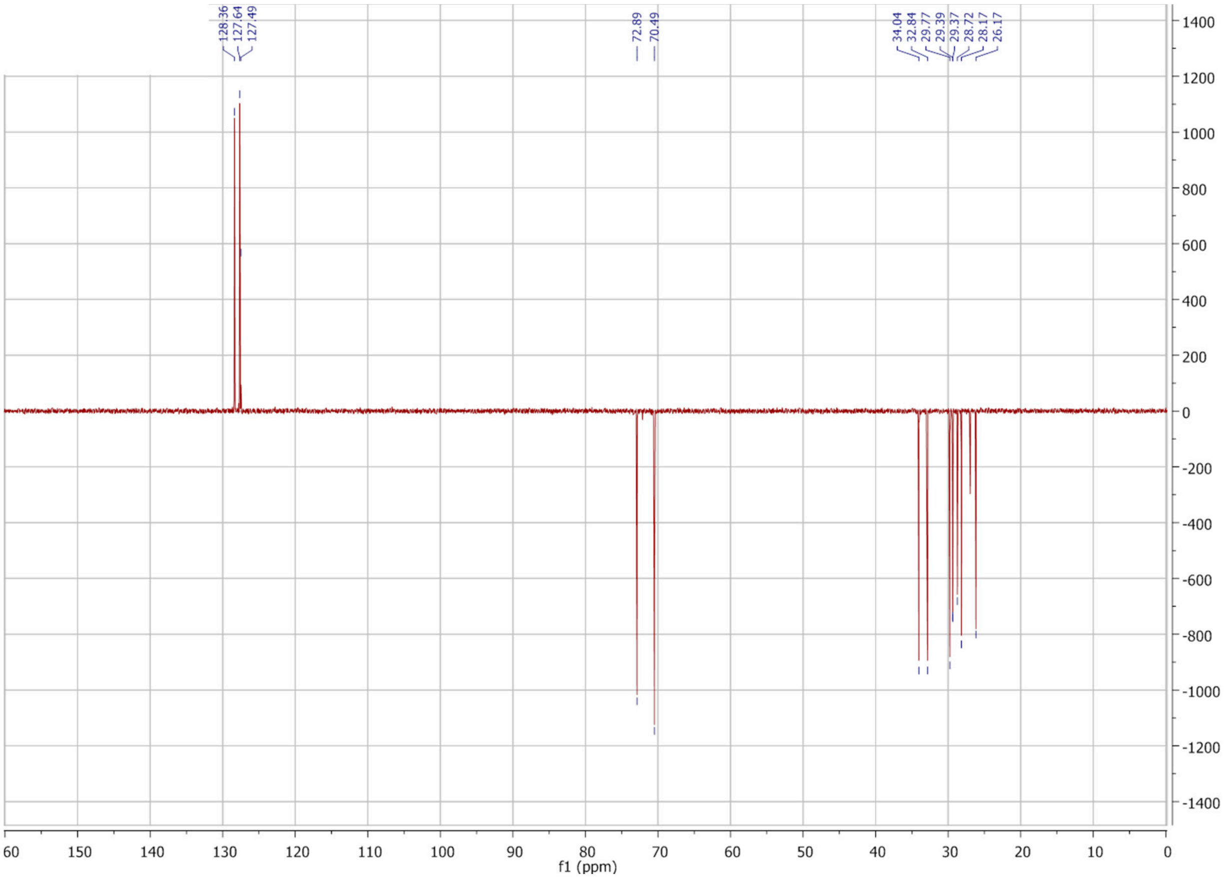

{[(10-bromodecyl)oxy]methyl}benzene (**7d**; ZHAWOC6853)

<u>NMR</u>

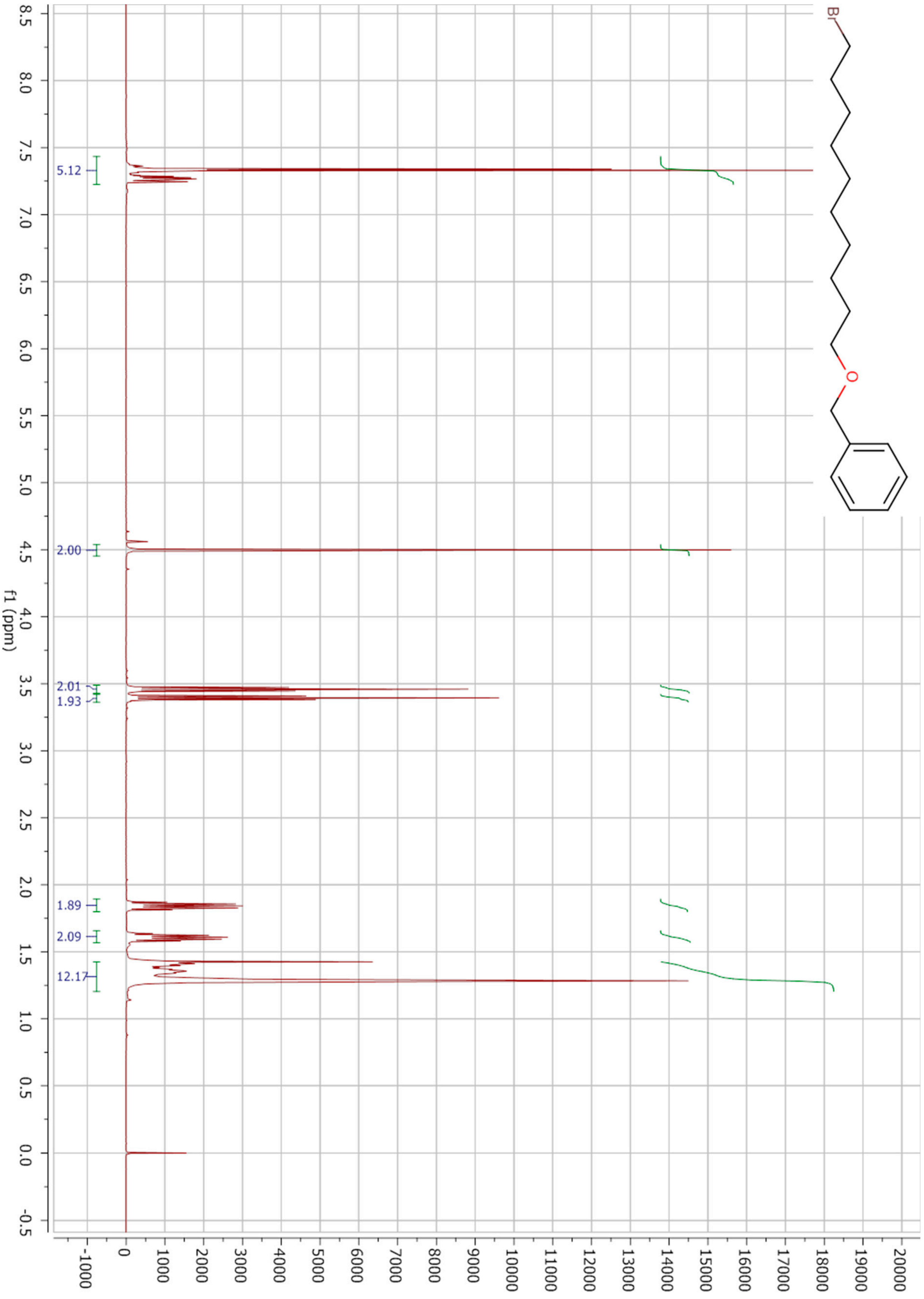

{[(10-bromodecyl)oxy]methyl}benzene (**7d**; ZHAWOC6853)

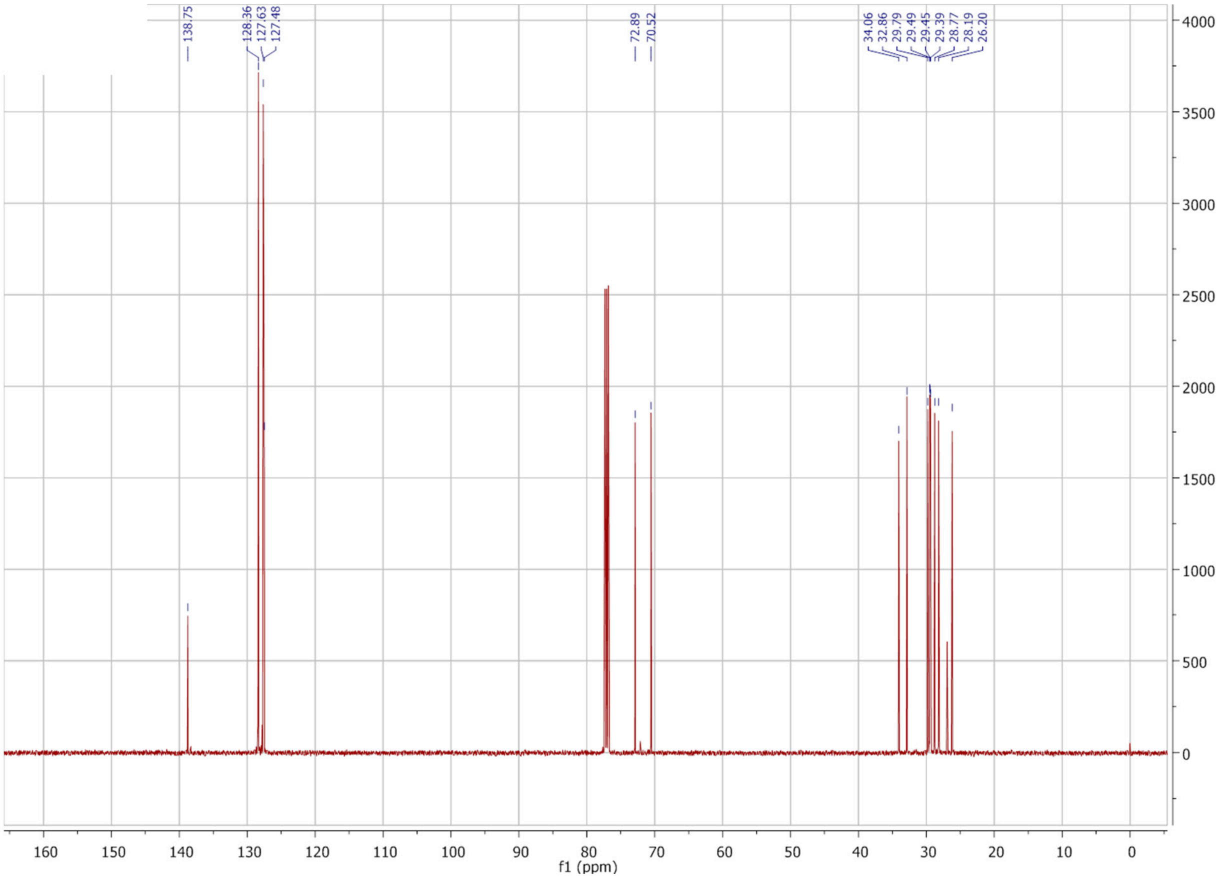

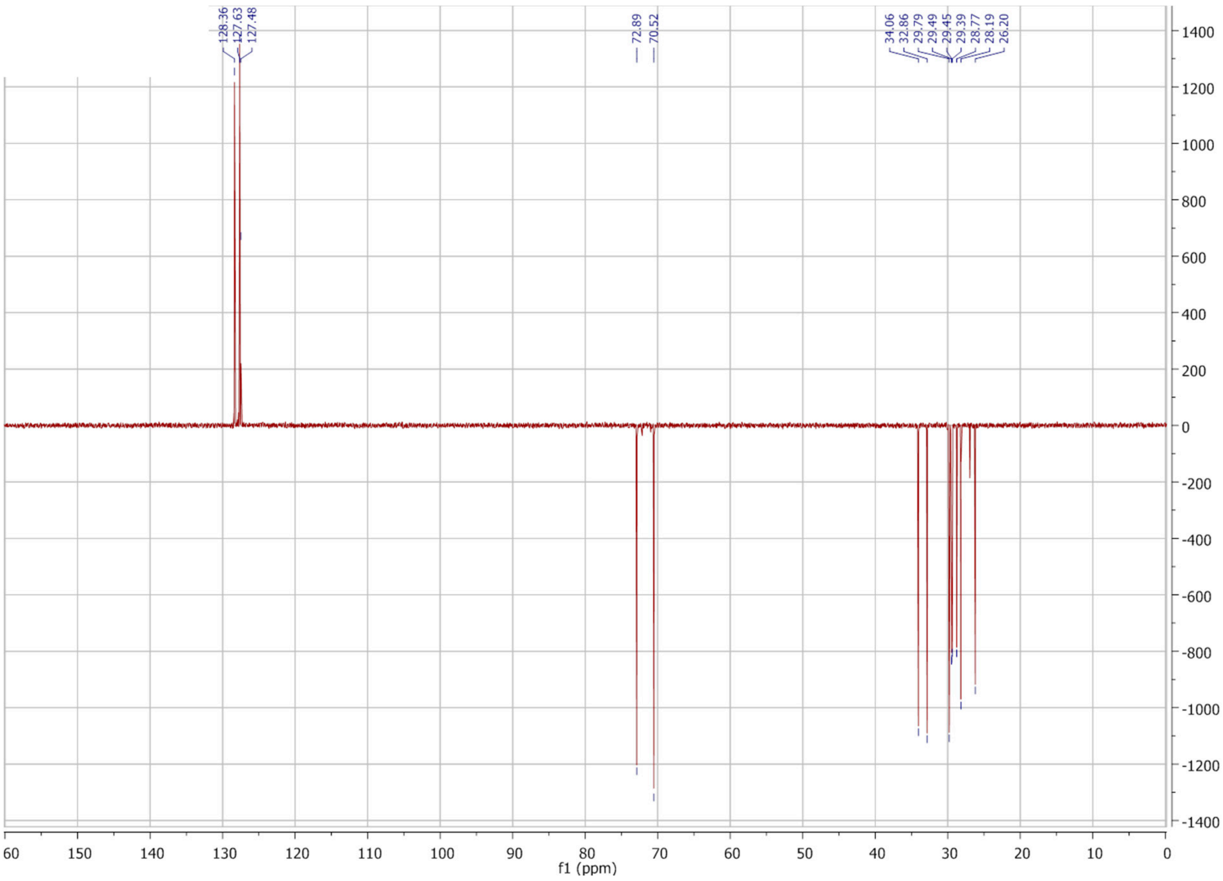

Methyl 2-{4-[2-(benzyloxy)ethoxy]phenyl}acetate (**8a**; ZHAWOC7100)

<u>NMR</u>

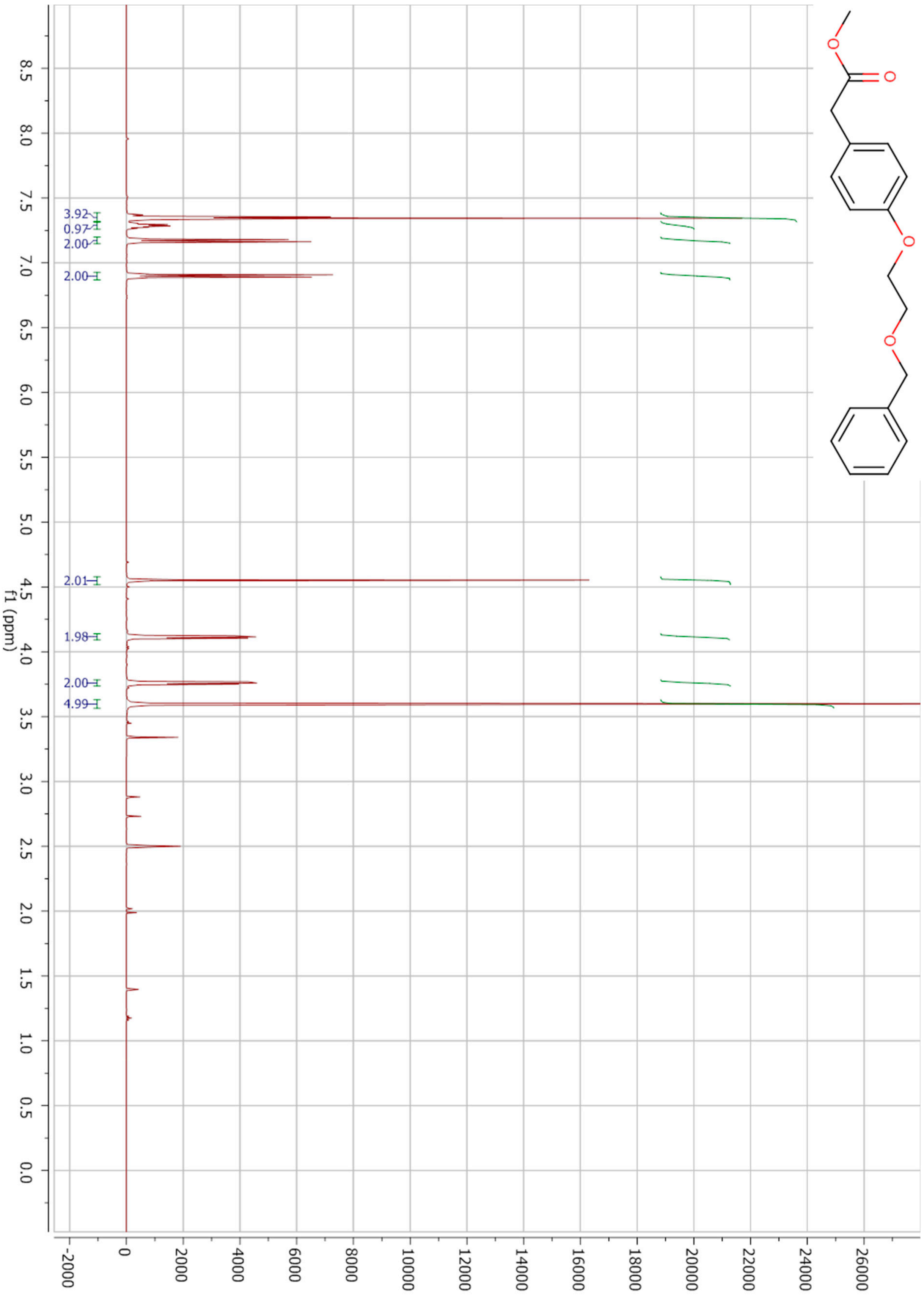

Methyl 2-{4-[2-(benzyloxy)ethoxy]phenyl}acetate (**8a**; ZHAWOC7100)

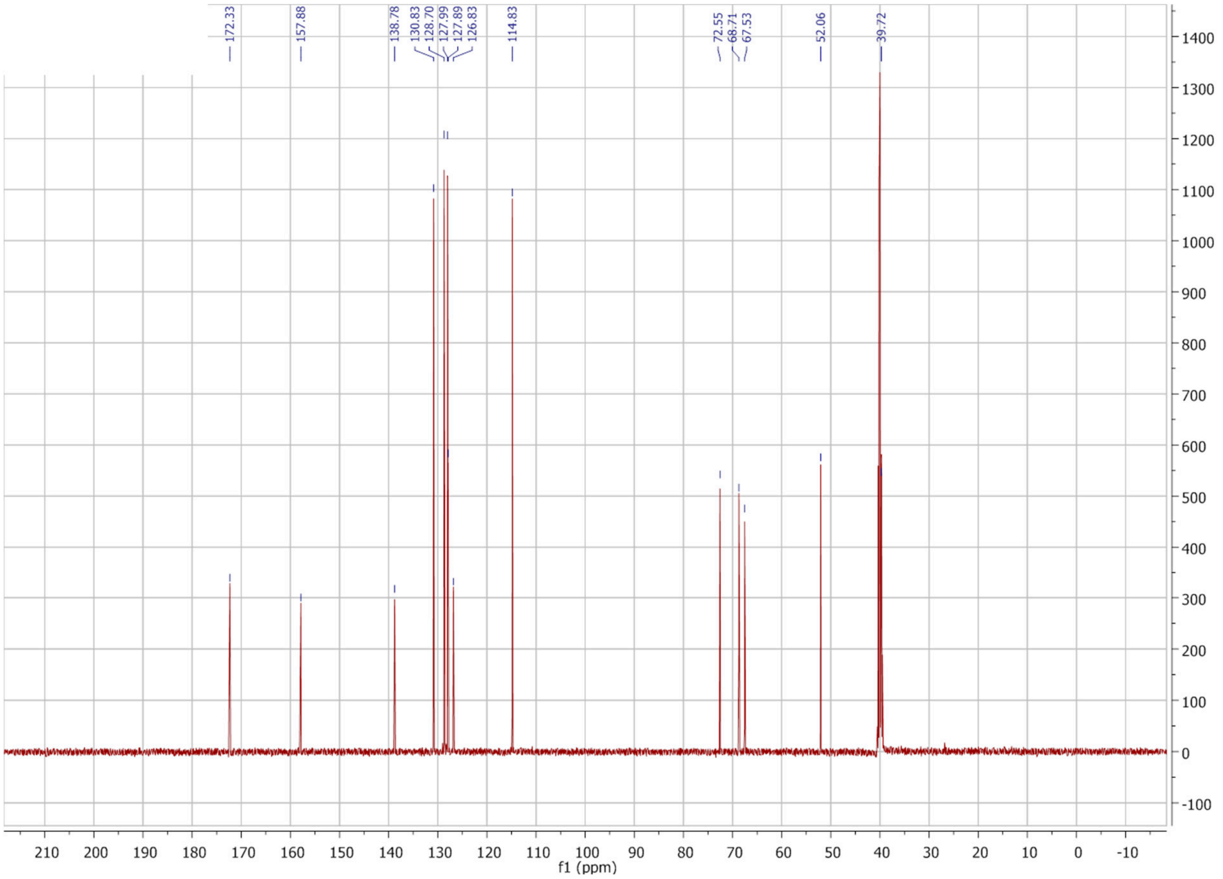

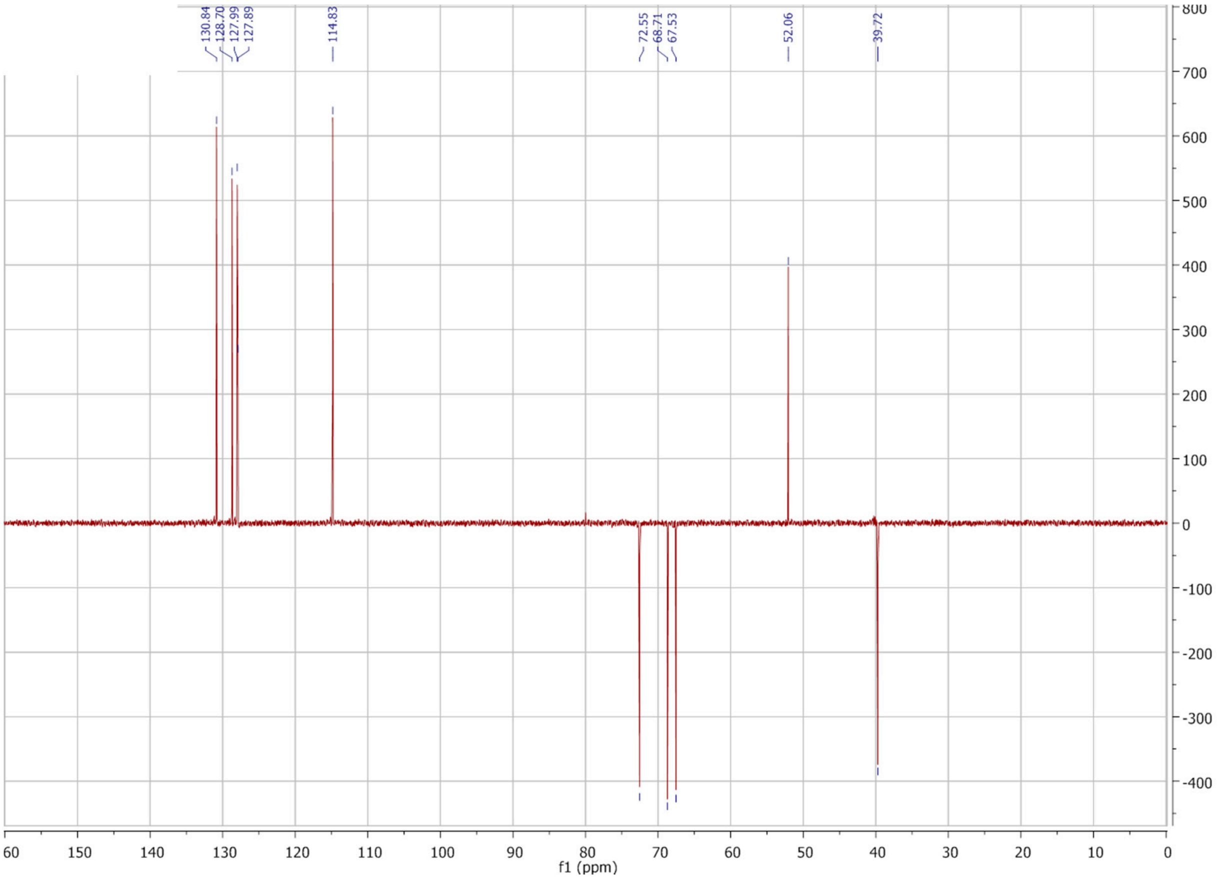

Methyl 2-{4-[3-(benzyloxy)propoxy]phenyl}acetate (**8b;** ZHAWOC4496)

<u>NMR</u>

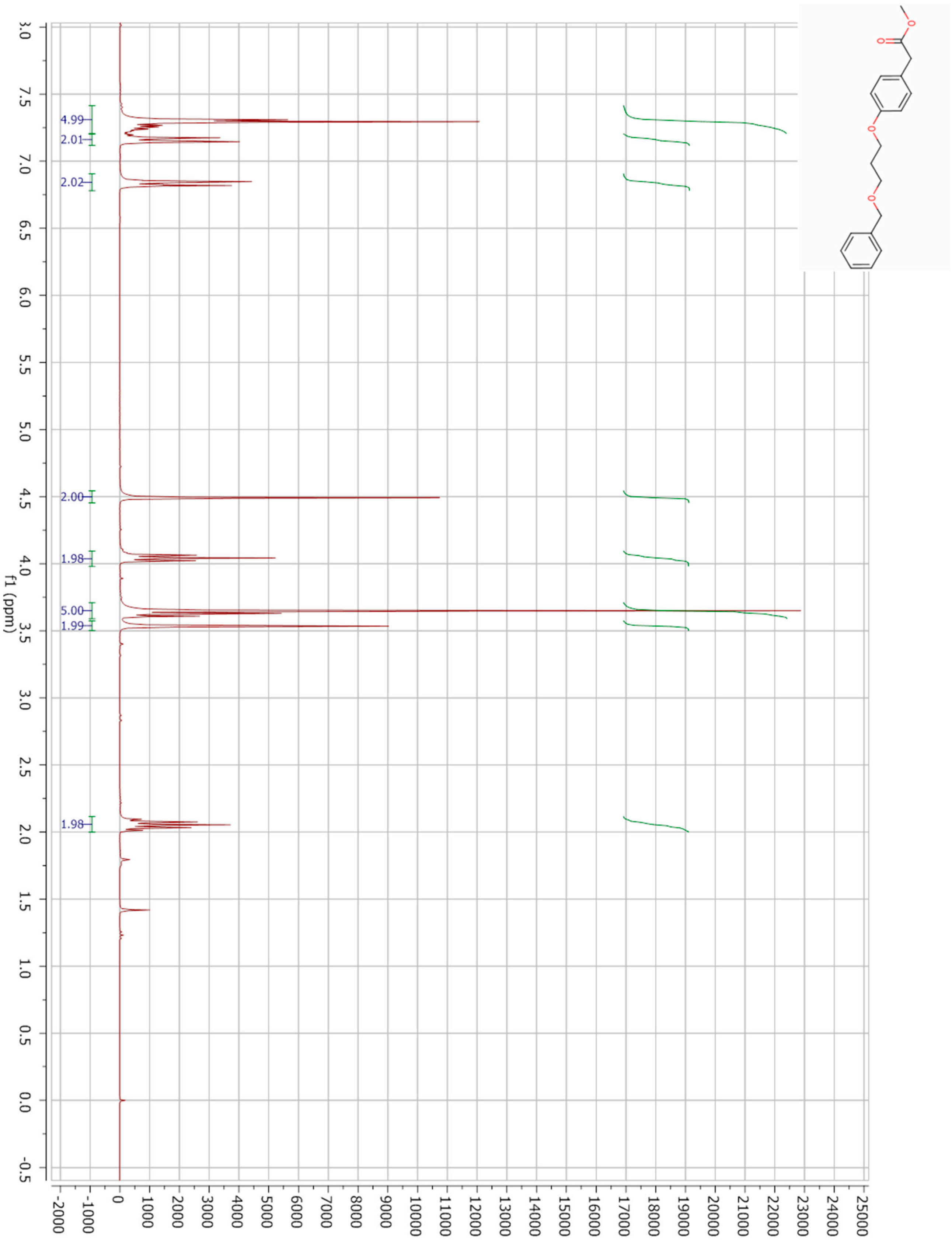

Methyl 2-{4-[3-(benzyloxy)propoxy]phenyl}acetate (**8b;** ZHAWOC4496)

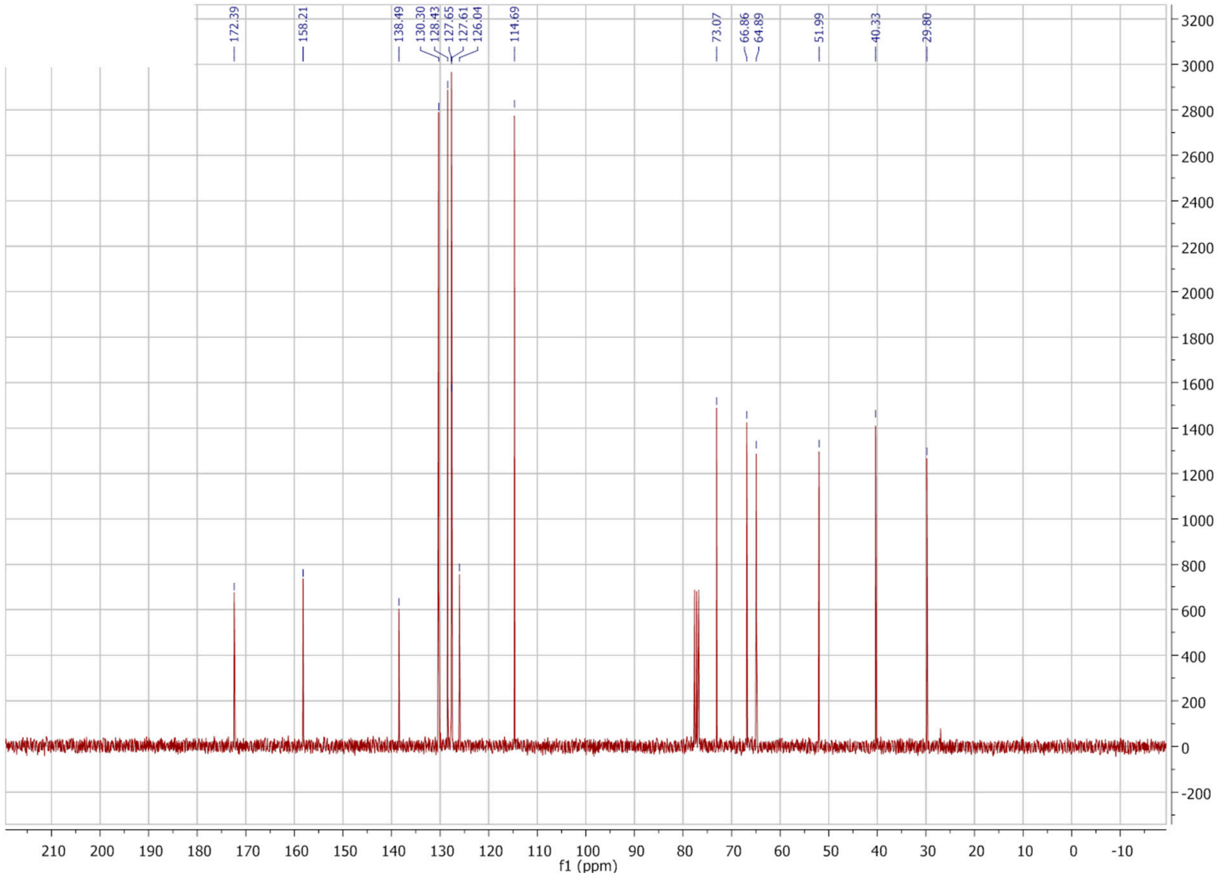

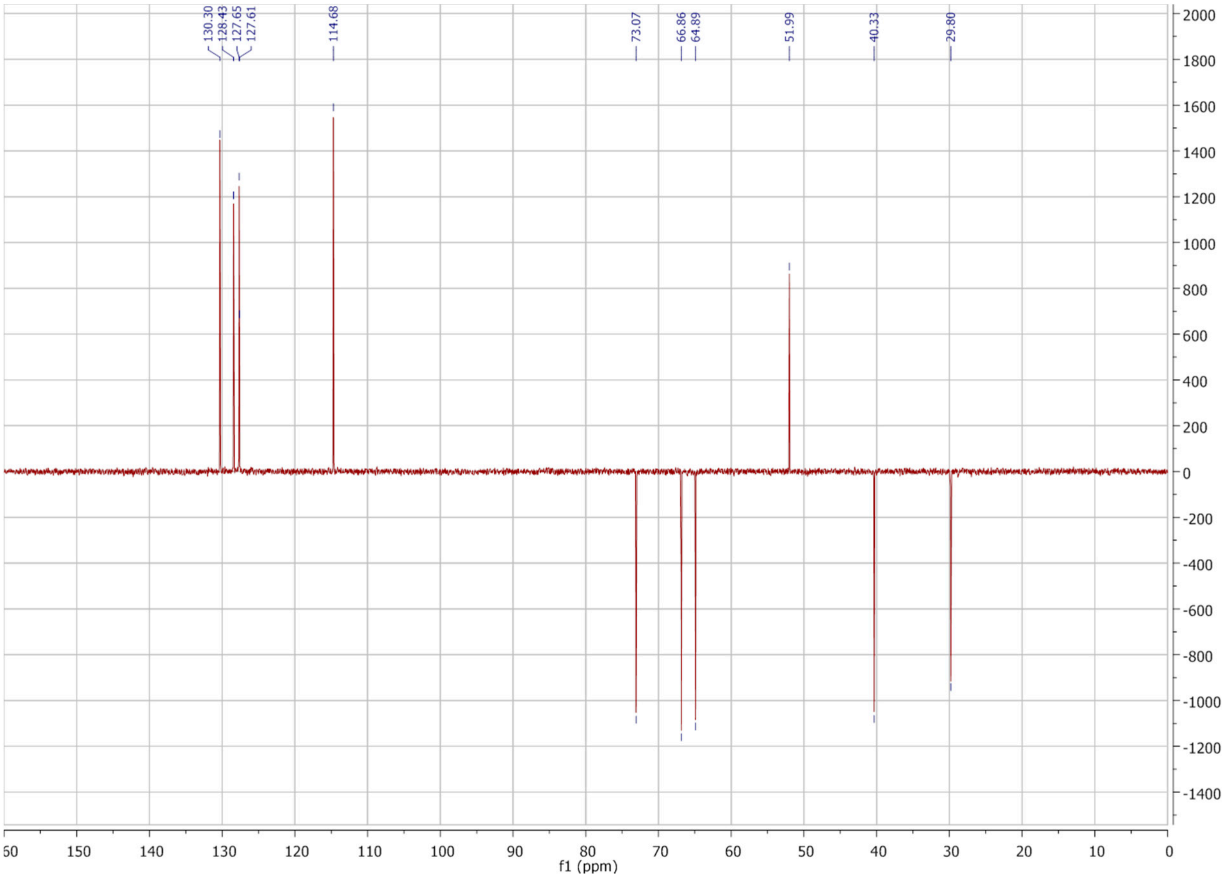

Methyl 2-{4-[4-(benzyloxy)butoxy]phenyl}acetate (**8c;** ZHAWOC4534)

<u>NMR</u>

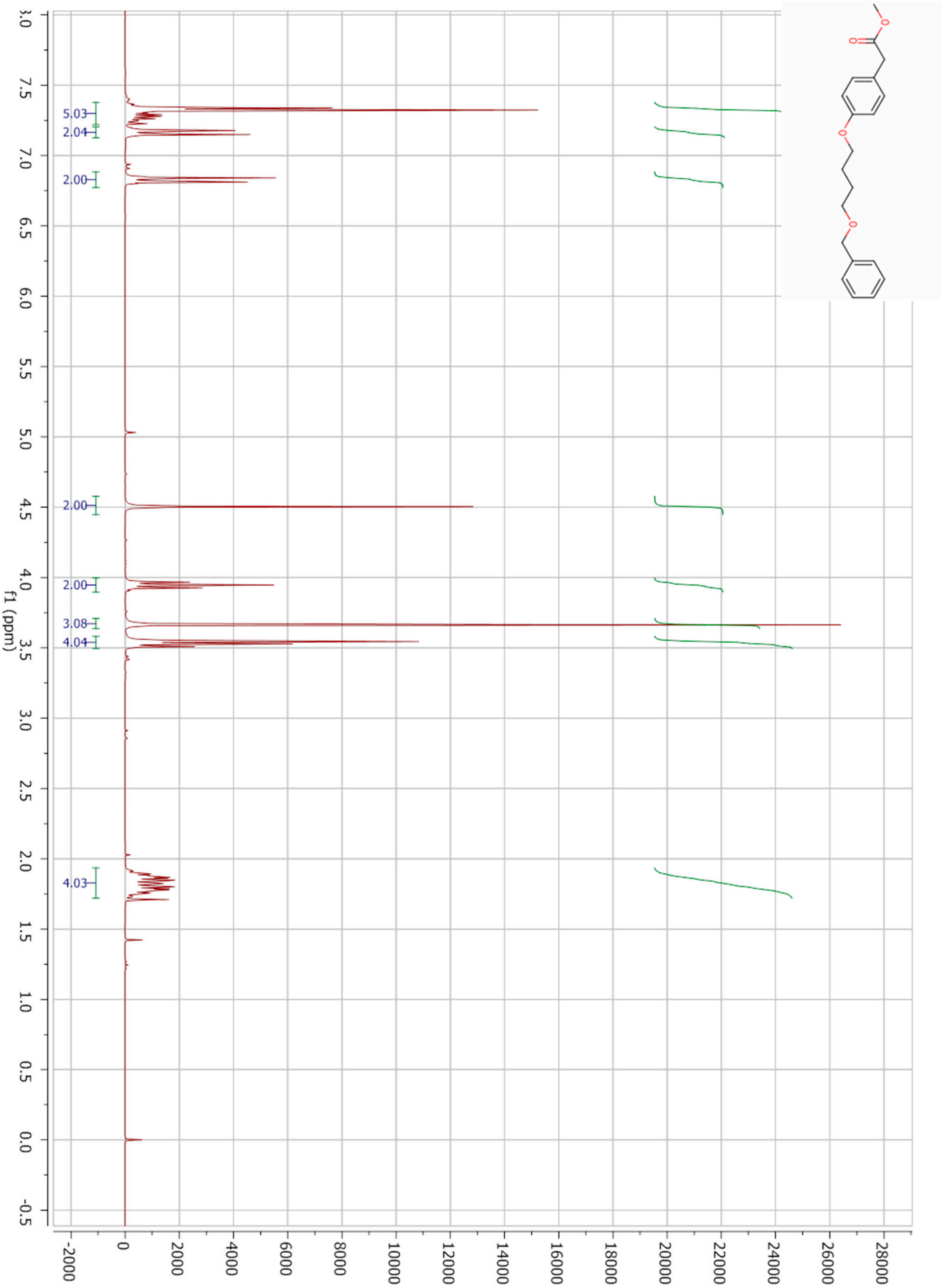

Methyl 2-{4-[4-(benzyloxy)butoxy]phenyl}acetate (**8c;** ZHAWOC4534)

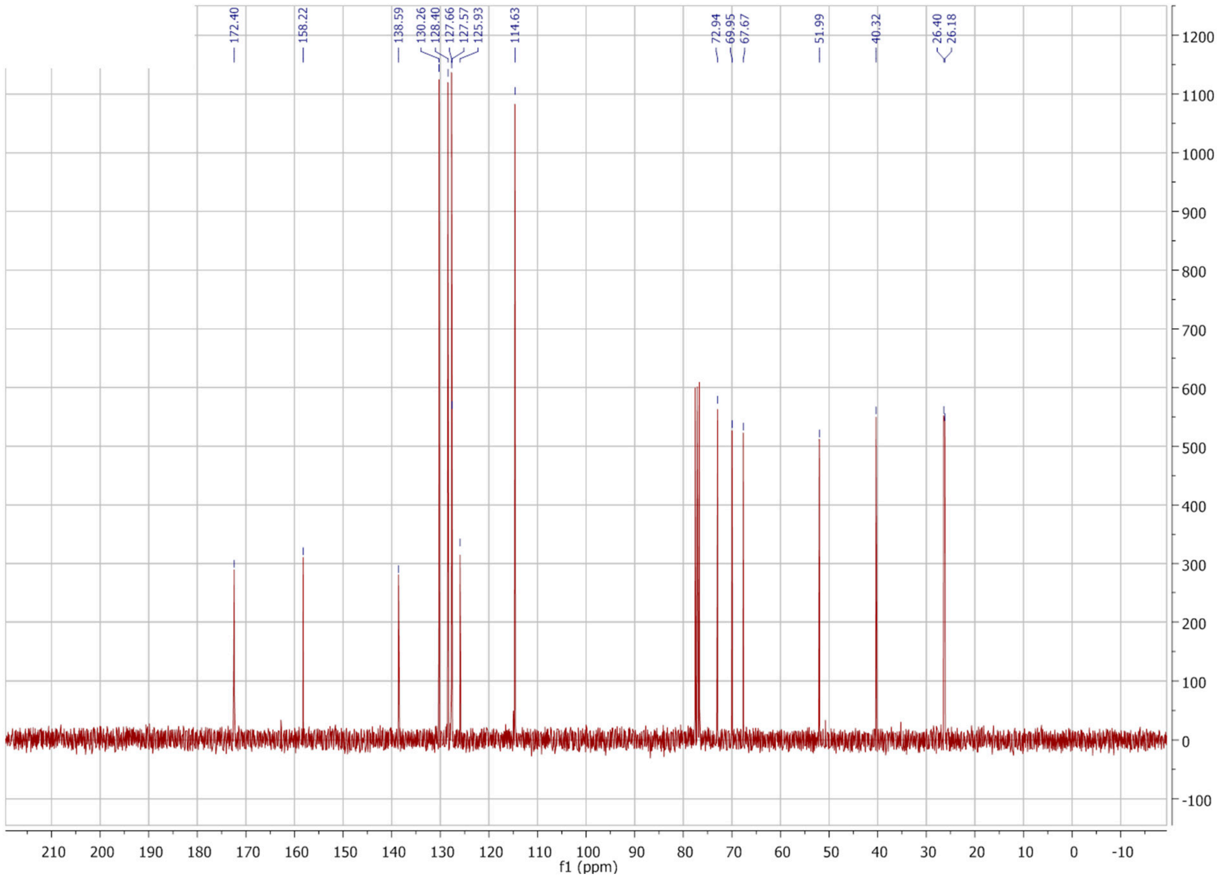

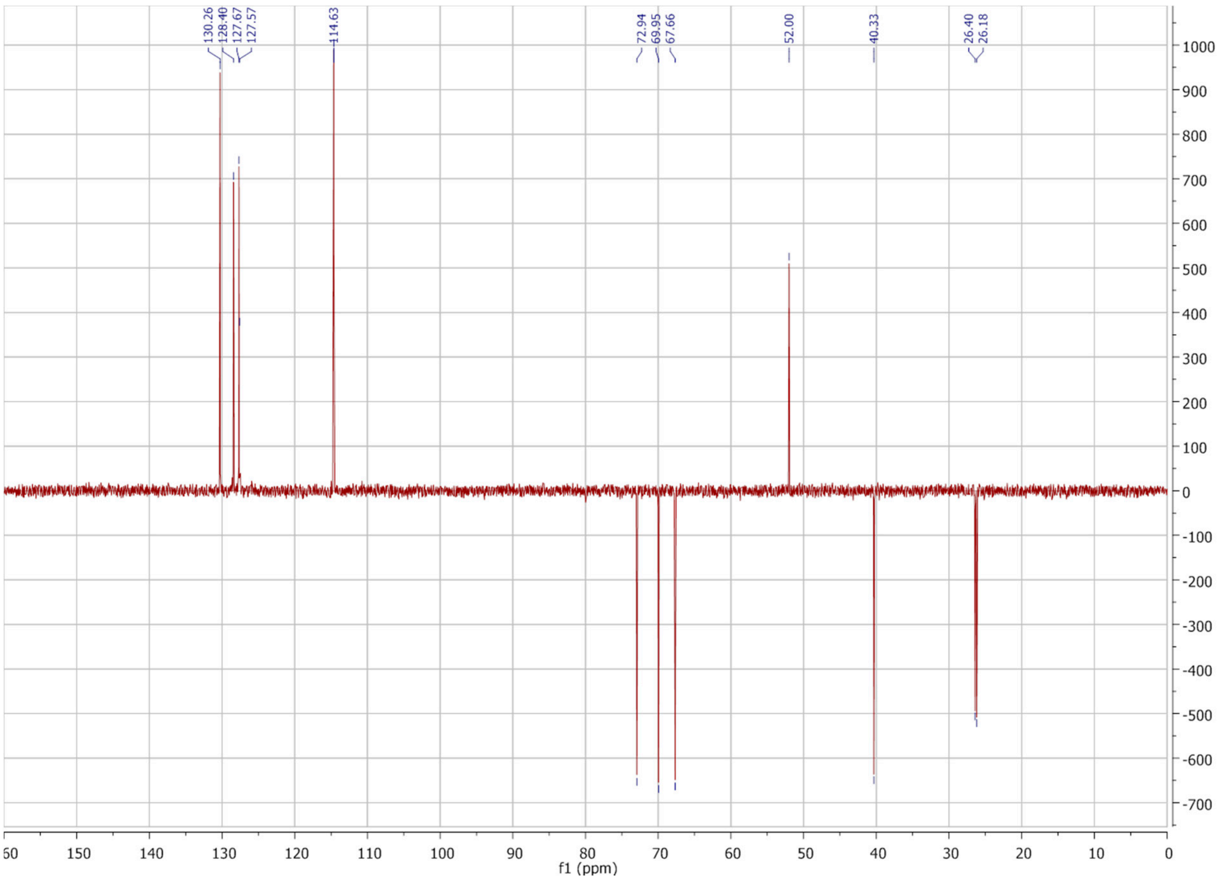

Methyl 2-(4-{[5-(benzyloxy)pentyl]oxy}phenyl)acetate (**8d;** ZHAWOC5921)

<u>NMR</u>

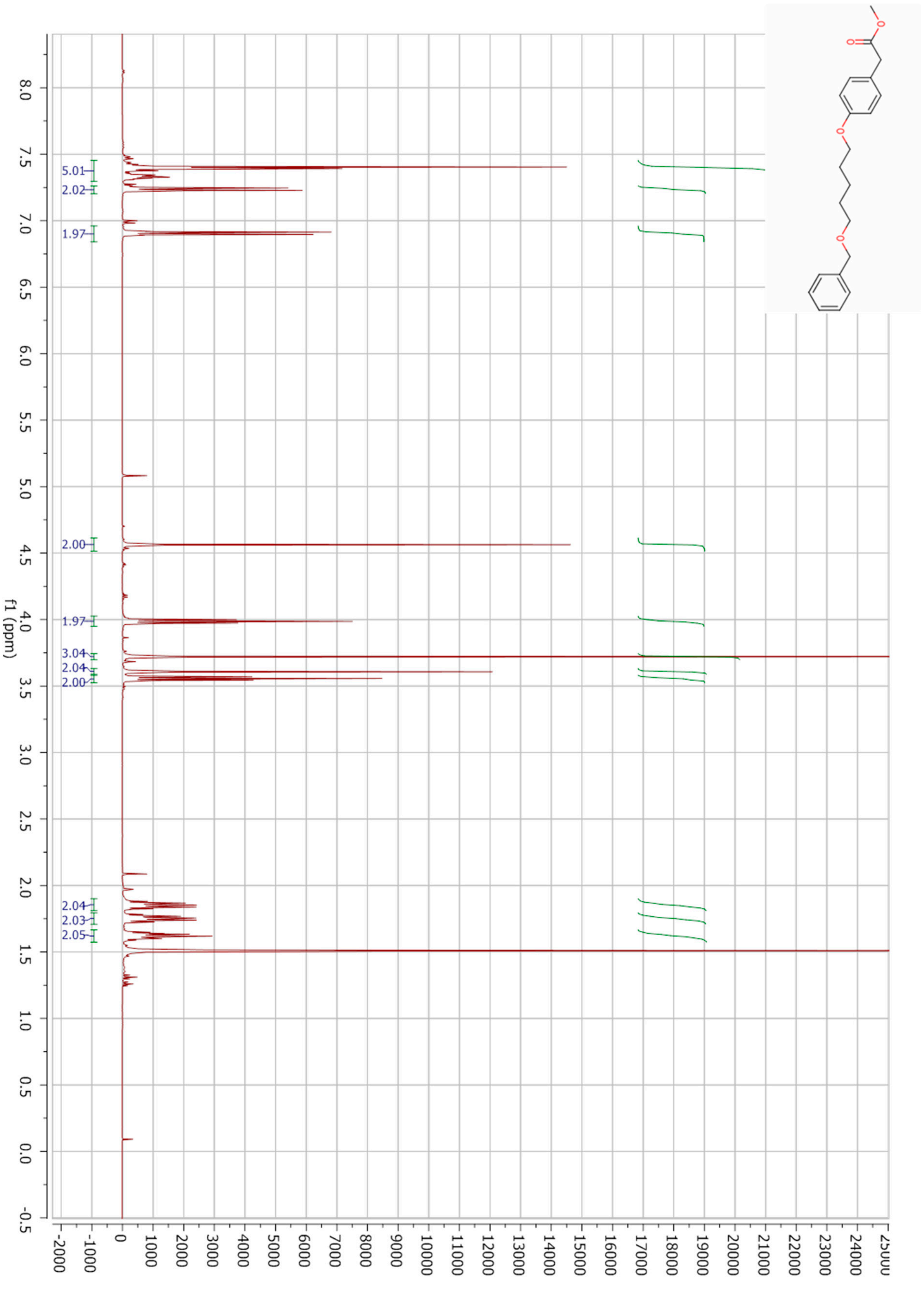

Methyl 2-(4-{[5-(benzyloxy)pentyl]oxy}phenyl)acetate (**8d;** ZHAWOC5921)

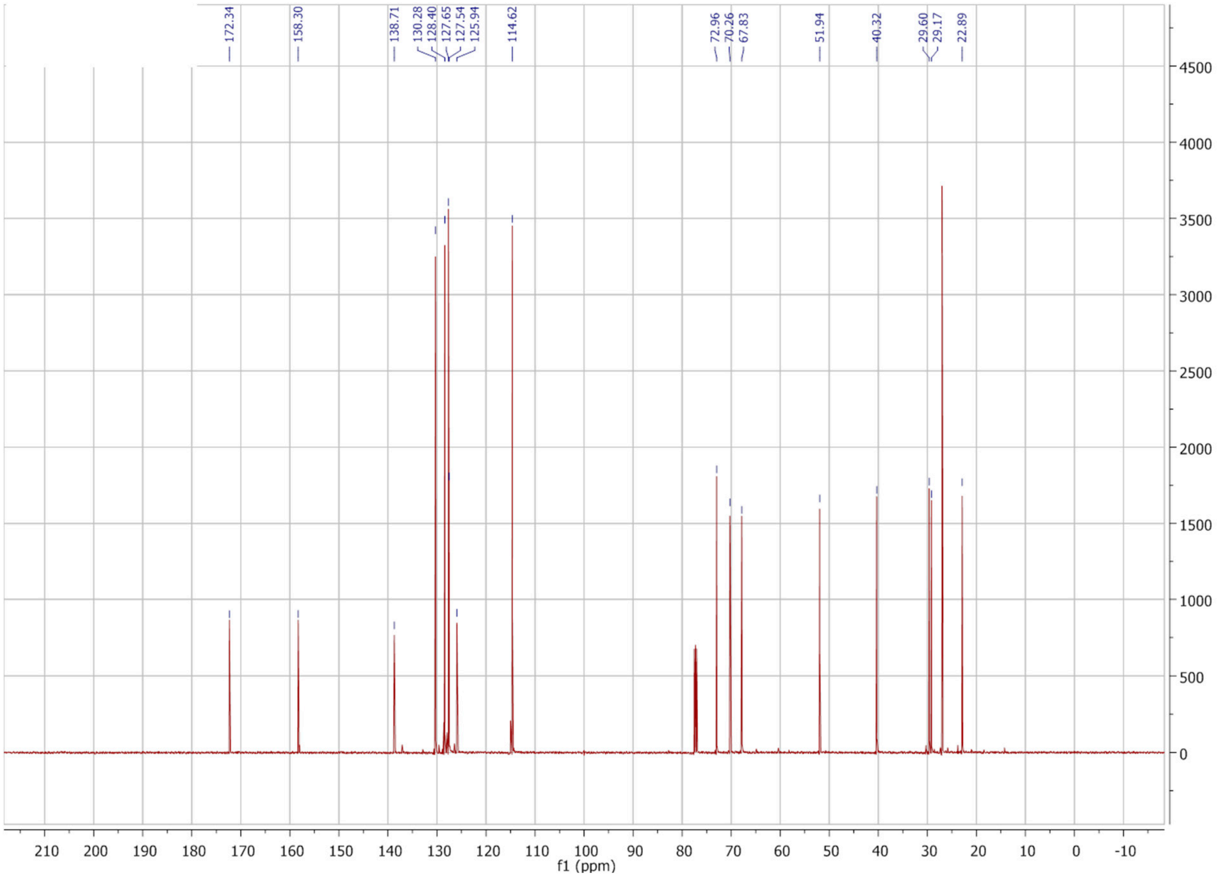

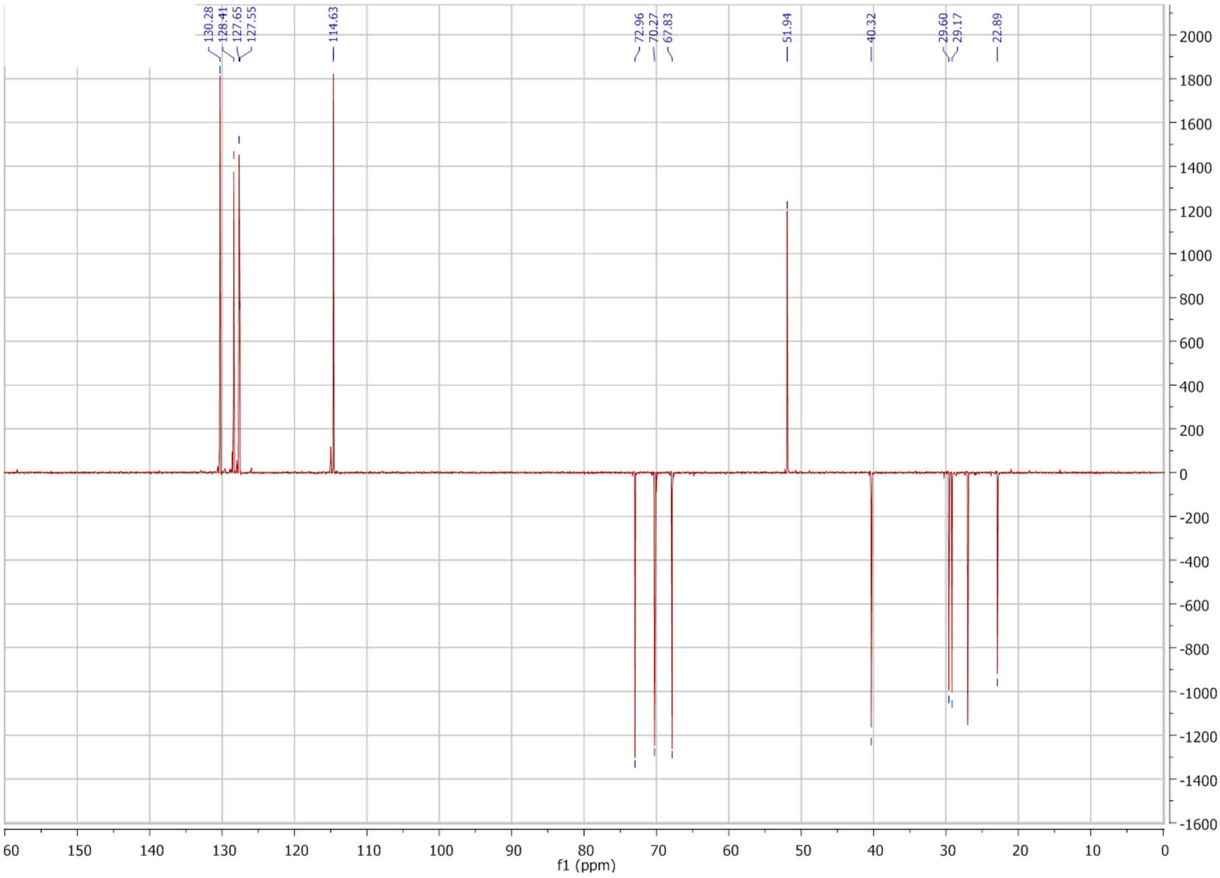

Methyl 2-(4-{[6-(benzyloxy)hexyl]oxy}phenyl)acetate (**8e;** ZHAWOC5946)

<u>NMR</u>

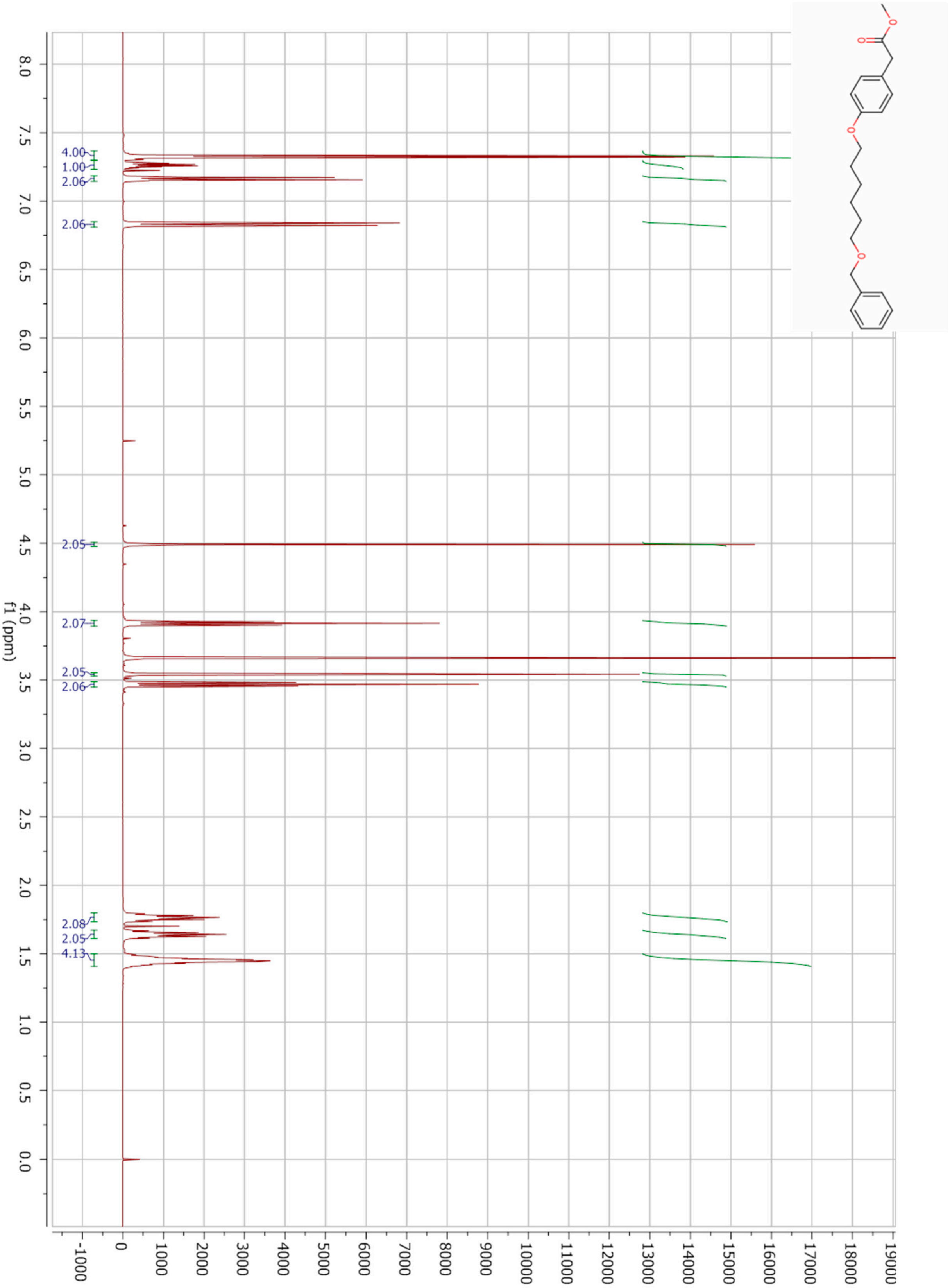

Methyl 2-(4-{[6-(benzyloxy)hexyl]oxy}phenyl)acetate (**8e;** ZHAWOC5946)

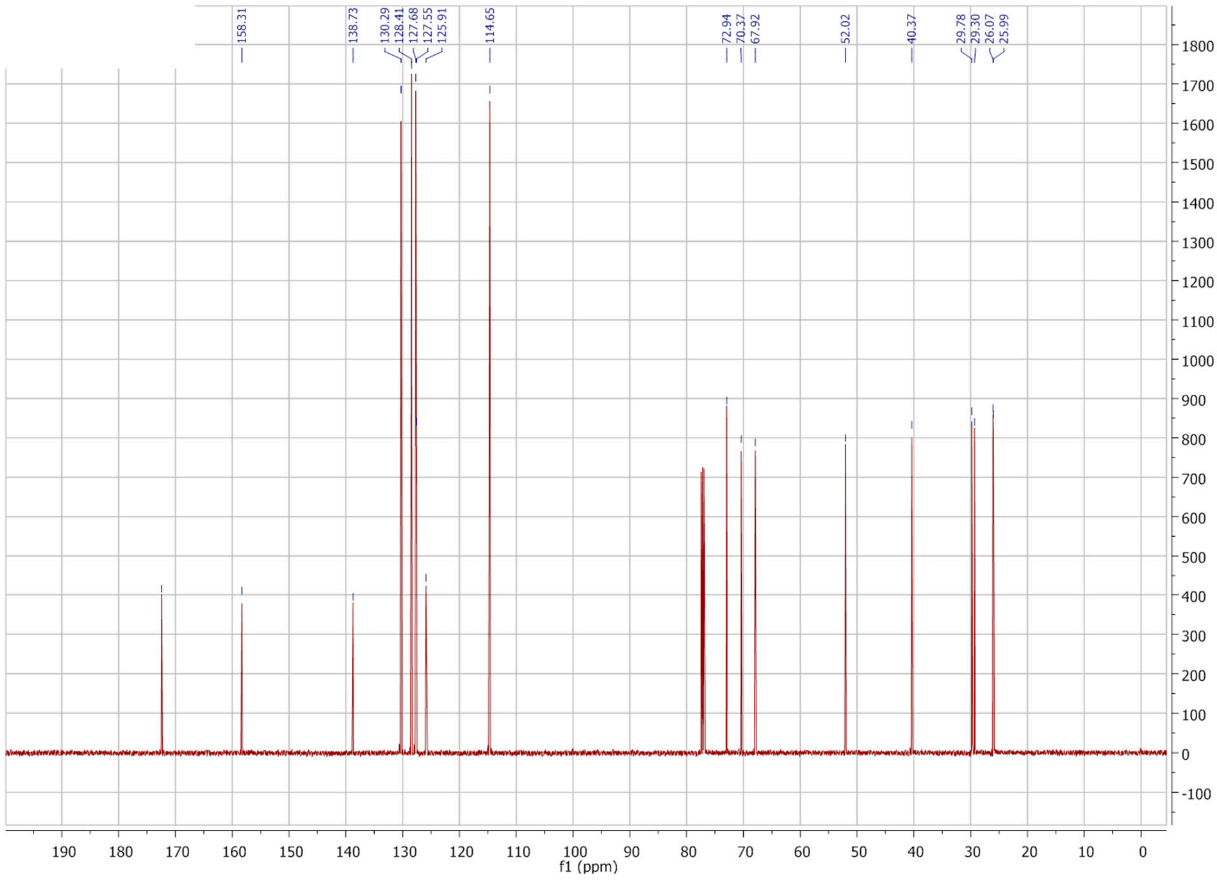

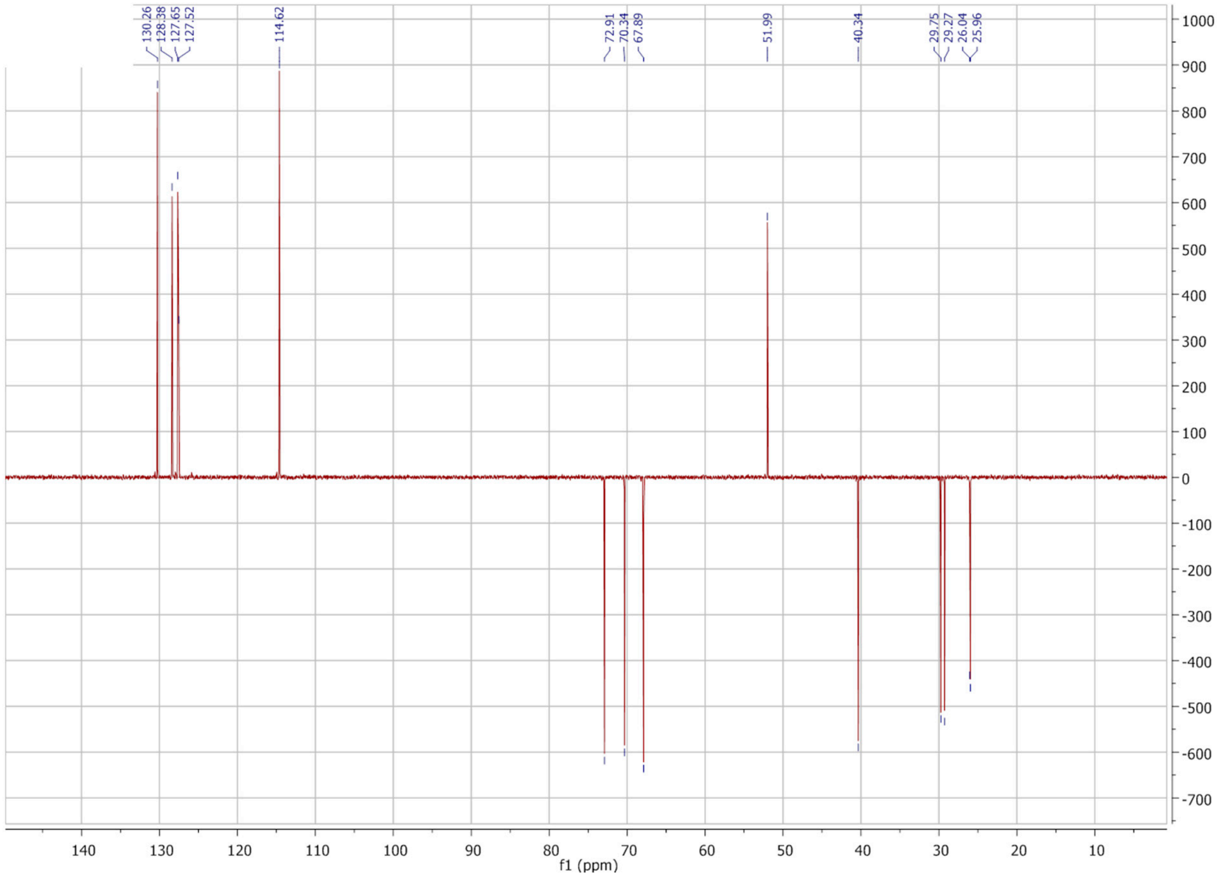

Methyl 2-(4-{[7-(benzyloxy)heptyl]oxy}phenyl)acetate (**8f**; ZHAWOC7097)

<u>NMR</u>

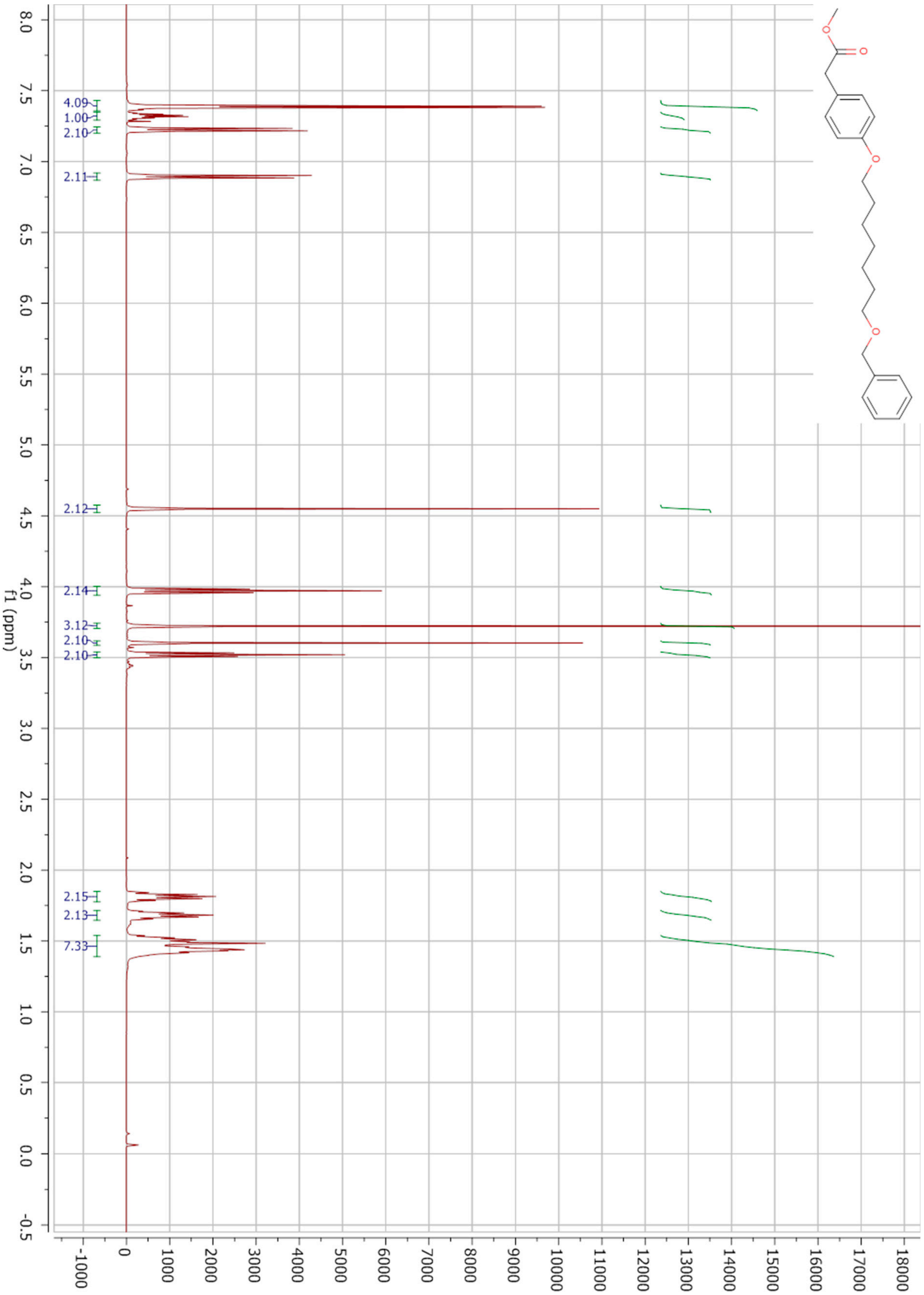

Methyl 2-(4-{[7-(benzyloxy)heptyl]oxy}phenyl)acetate (**8f**; ZHAWOC7097)

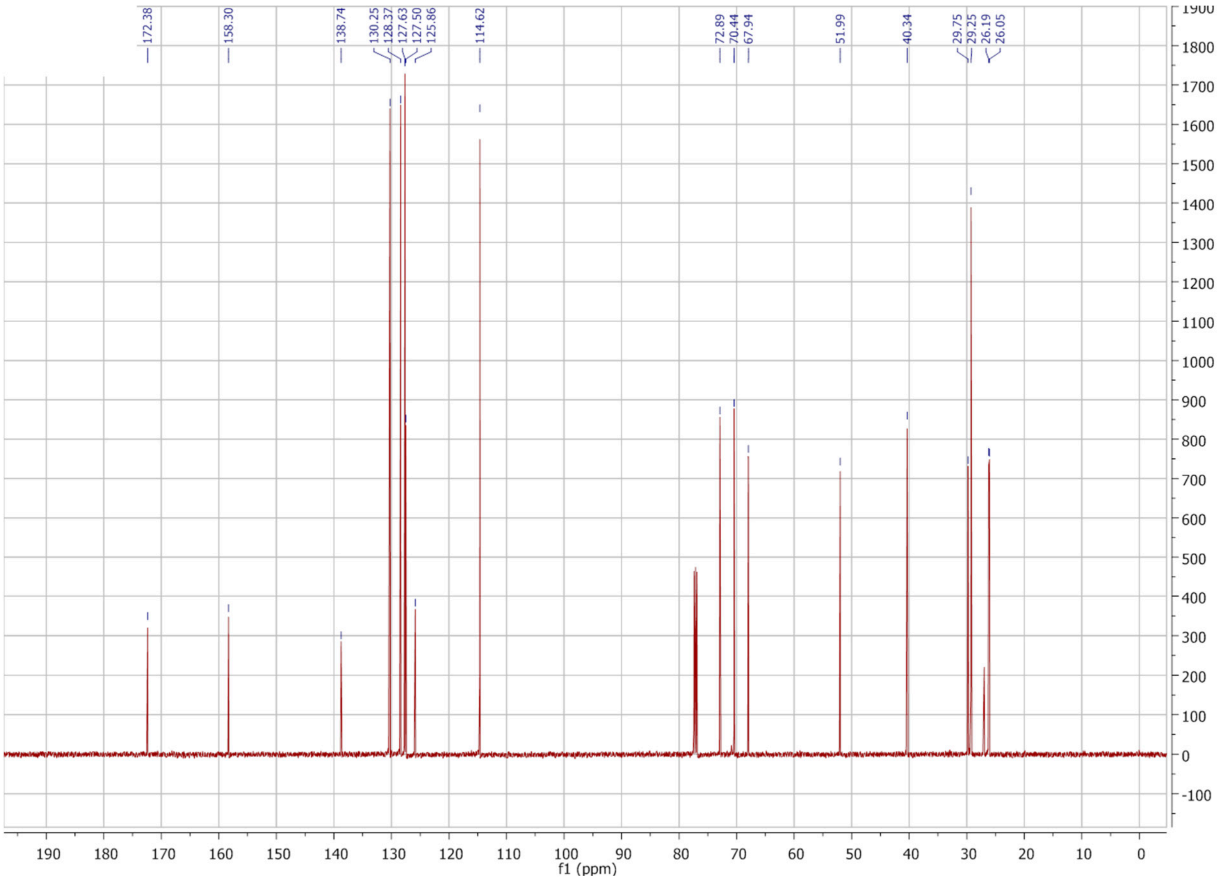

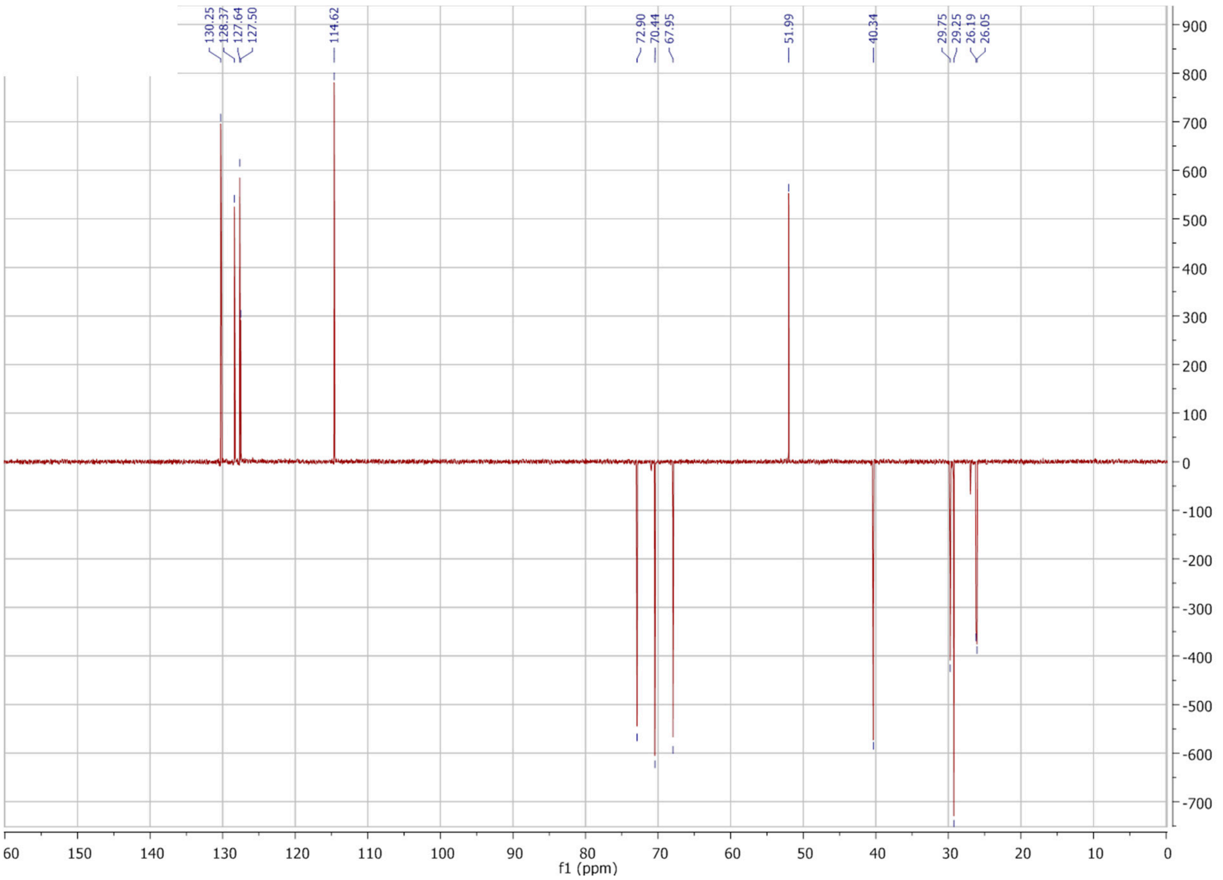

Methyl 2-(4-{[8-(benzyloxy)octyl]oxy}phenyl)acetate (**8g**; ZHAWOC6857)

<u>NMR</u>

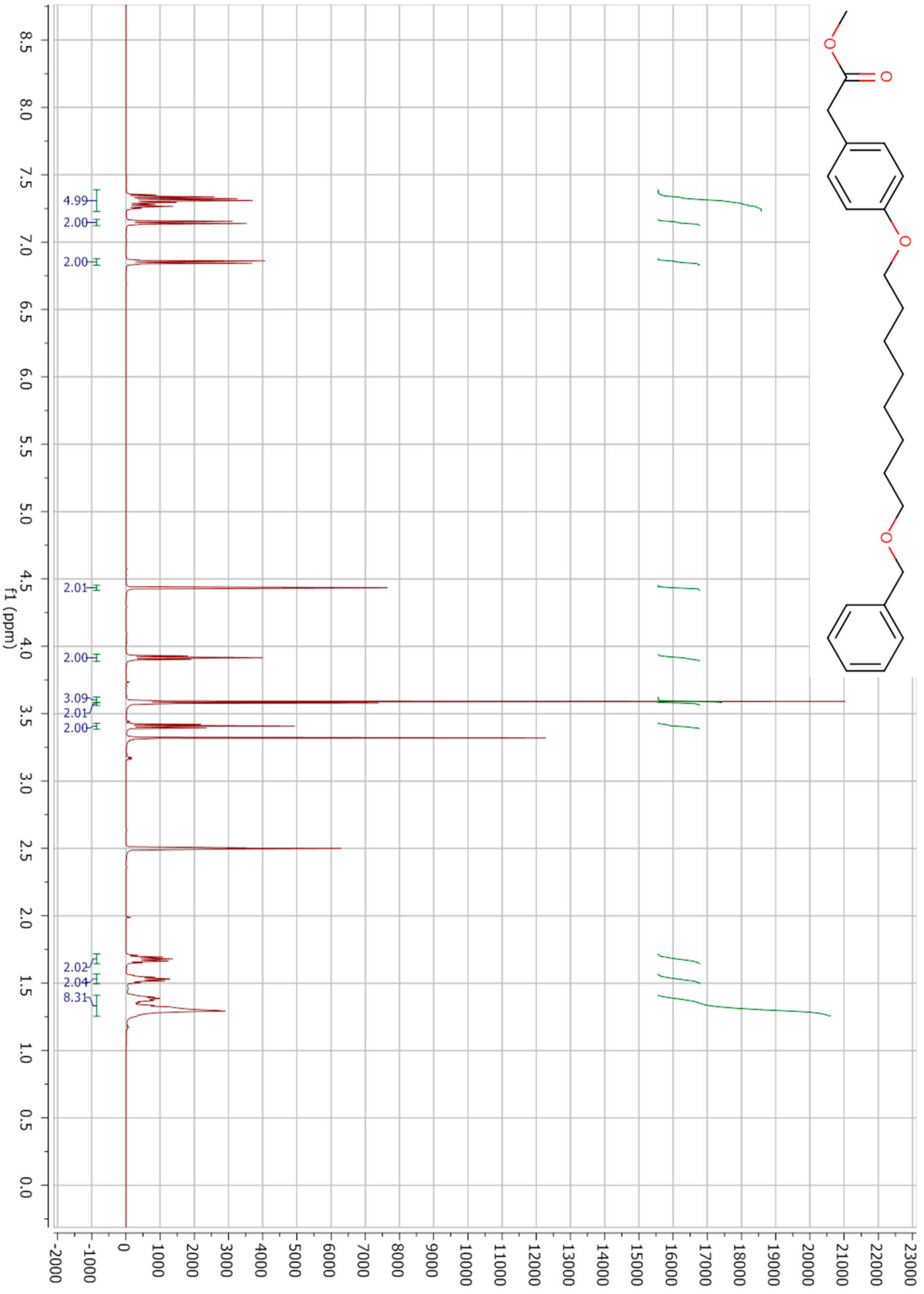

Methyl 2-(4-{[8-(benzyloxy)octyl]oxy}phenyl)acetate (**8g**; ZHAWOC6857)

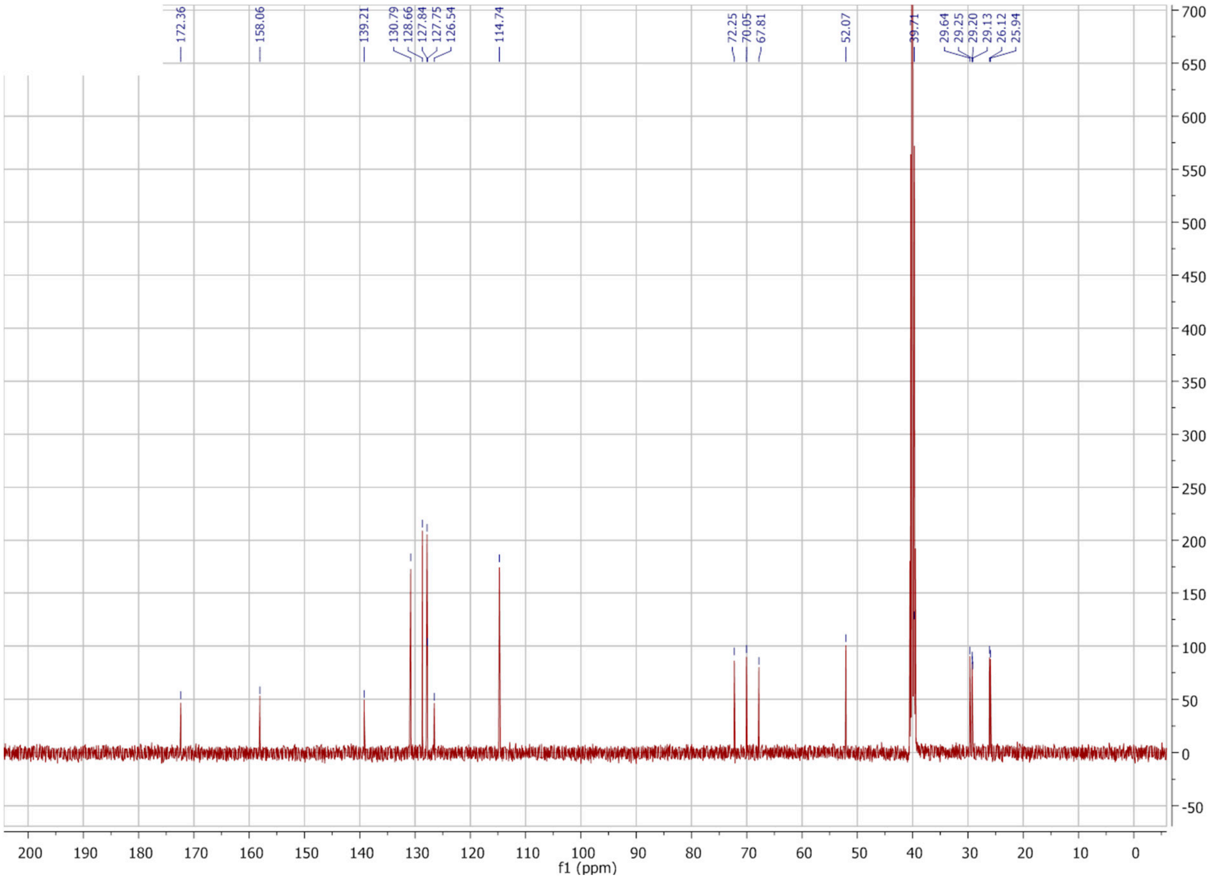

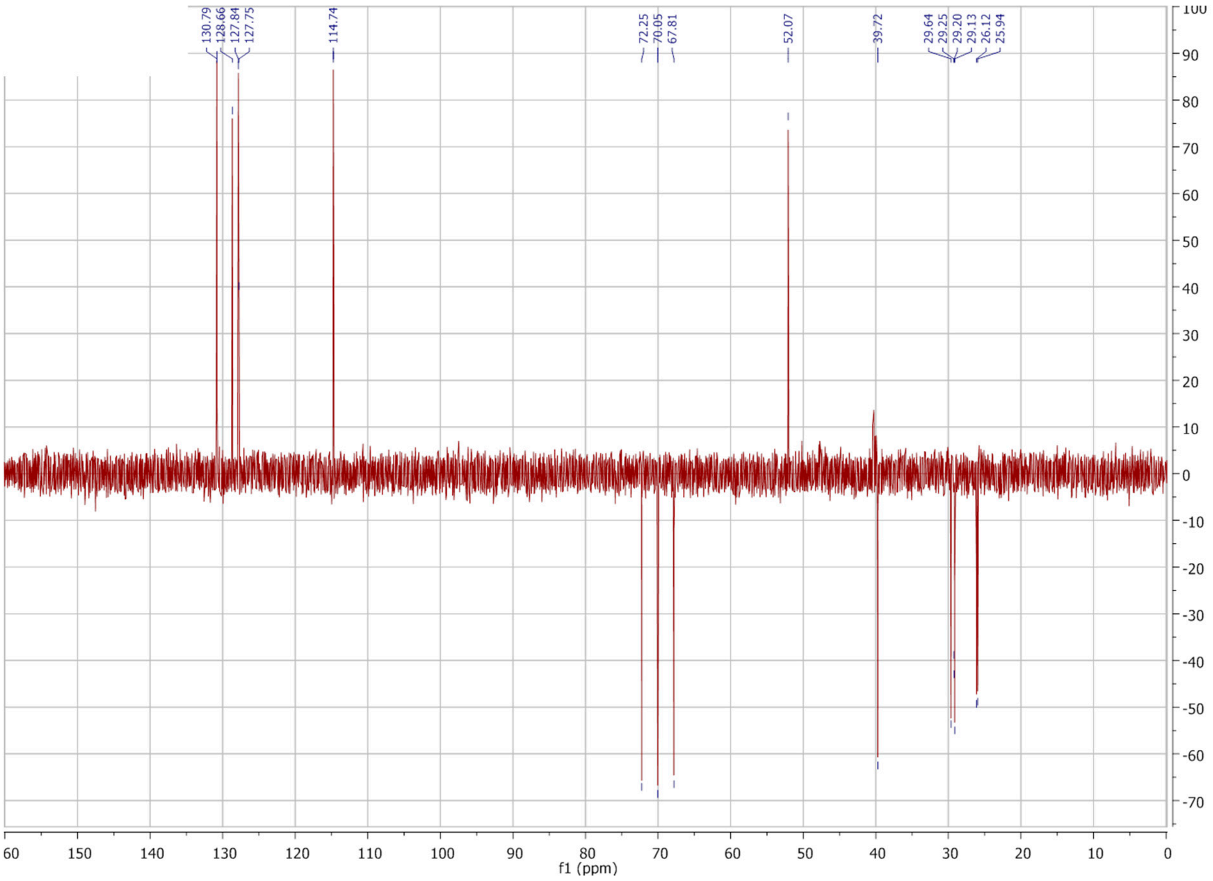

Methyl 2-(4-{[9-(benzyloxy)nonyl]oxy}phenyl)acetate (**8h**; ZHAWOC6854)

<u>NMR</u>

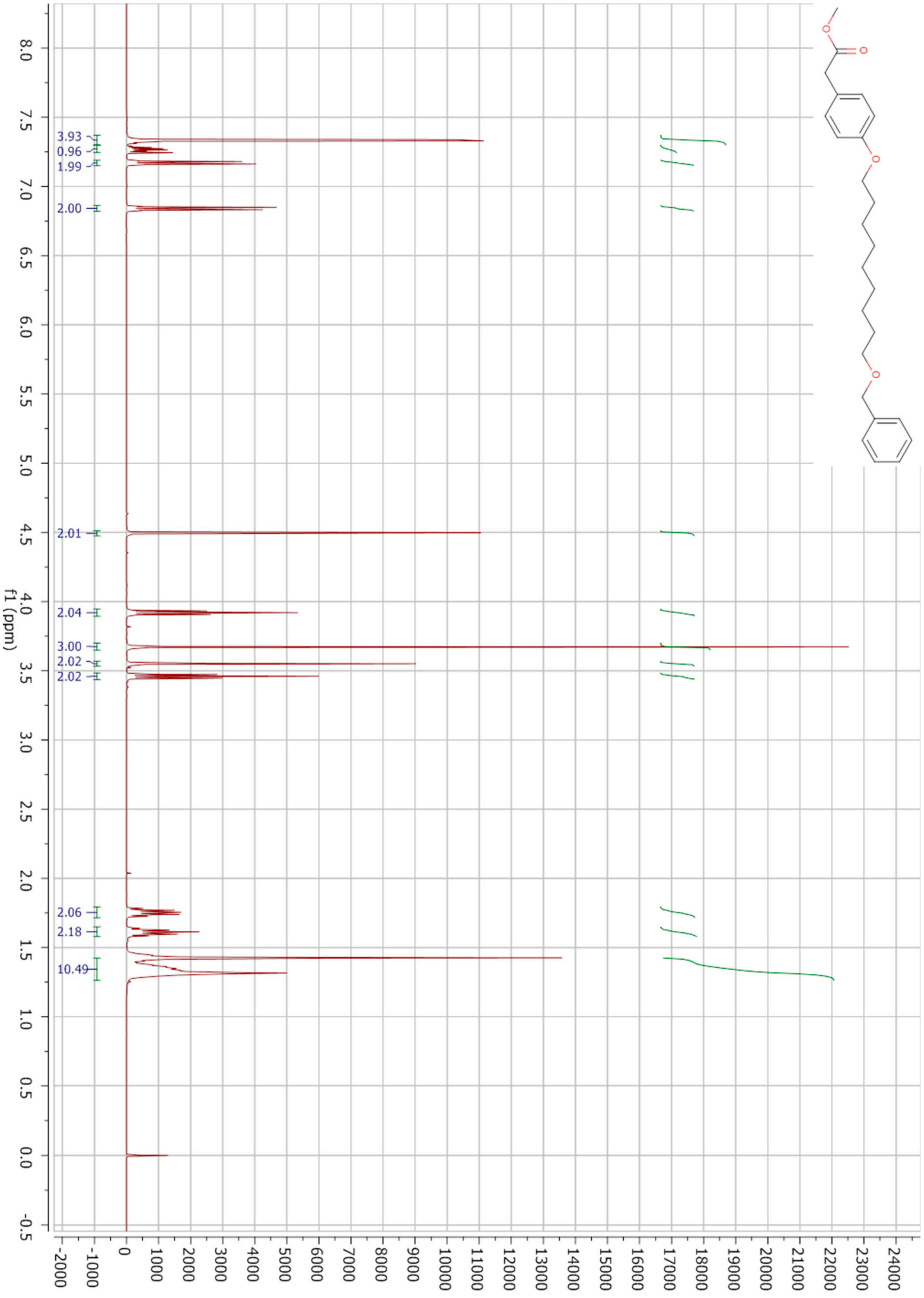

Methyl 2-(4-{[9-(benzyloxy)nonyl]oxy}phenyl)acetate (**8h**; ZHAWOC6854)

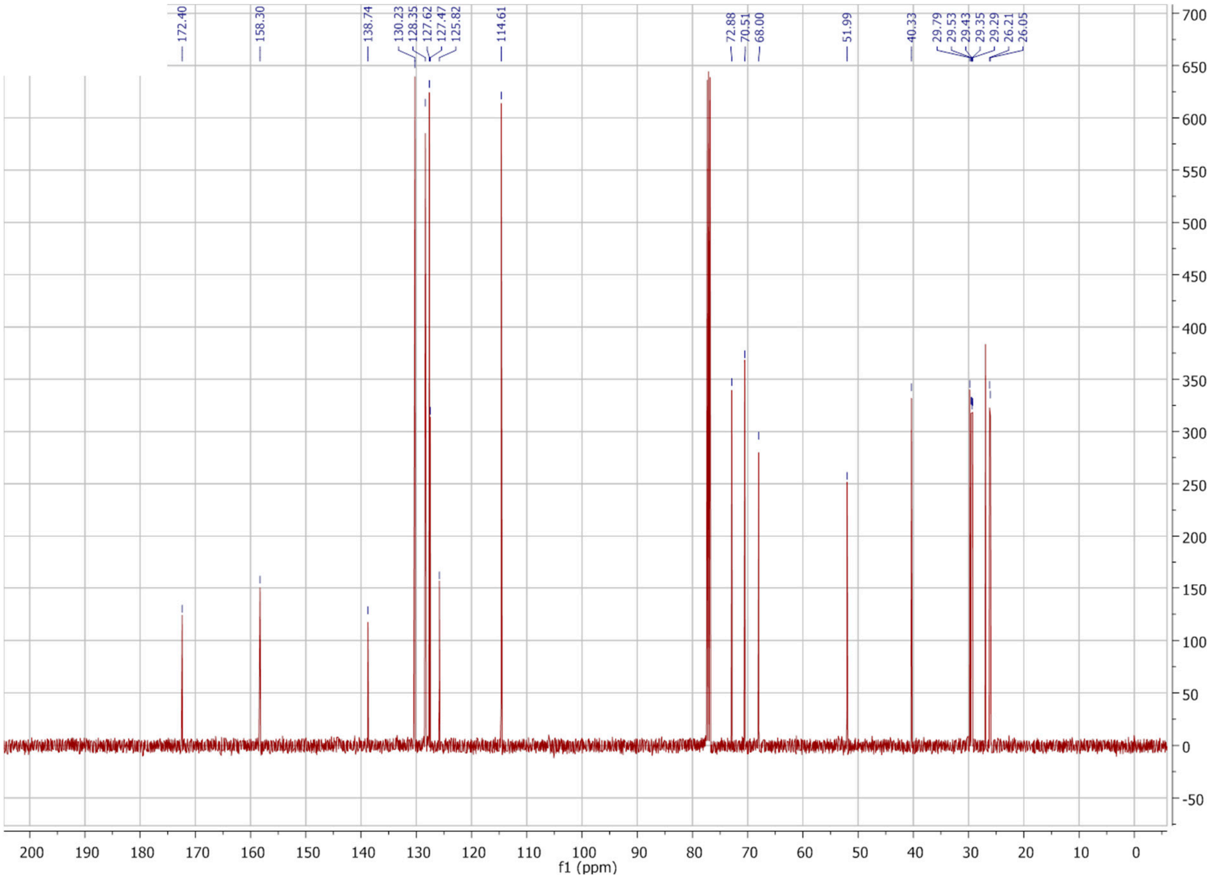

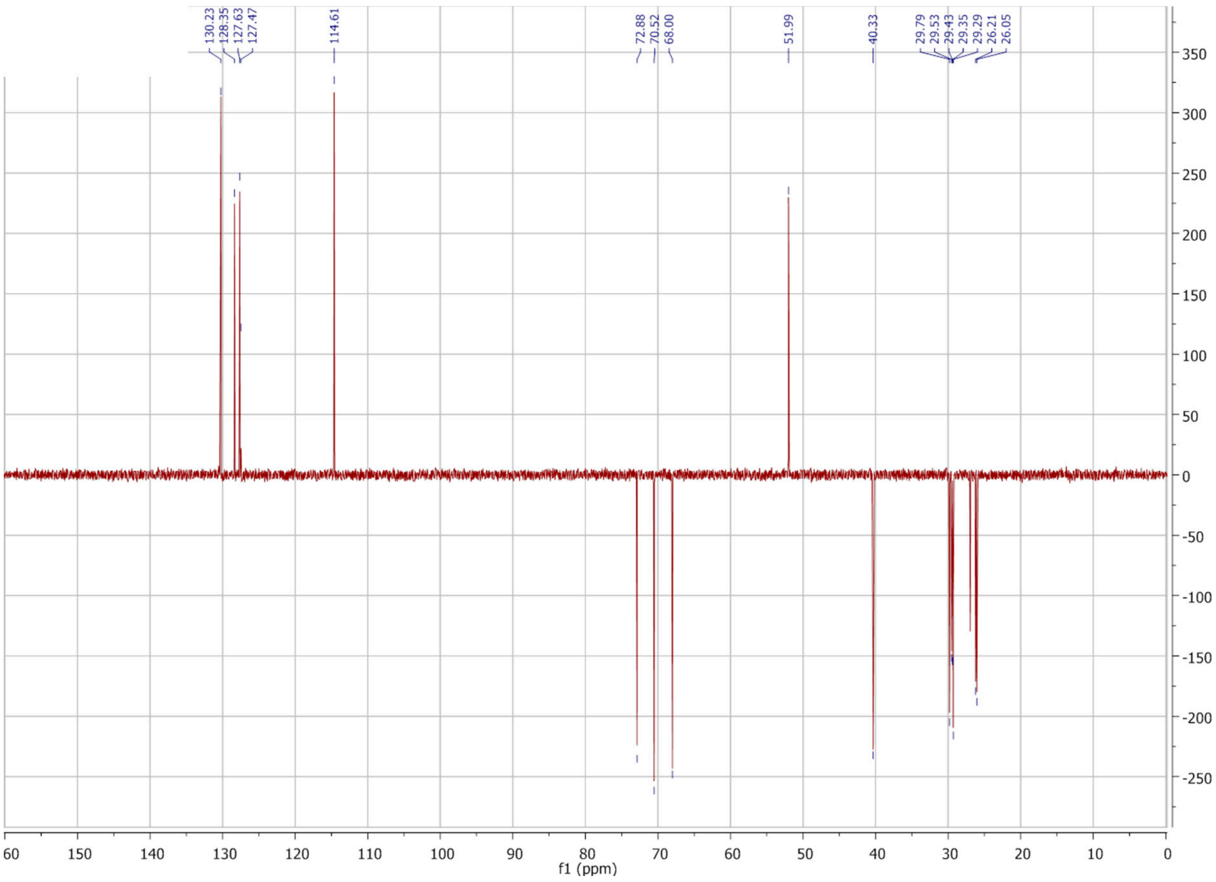

Methyl 2-(4-{[10-(benzyloxy)decyl]oxy}phenyl)acetate (**8i**; ZHAWOC6855)

<u>NMR</u>

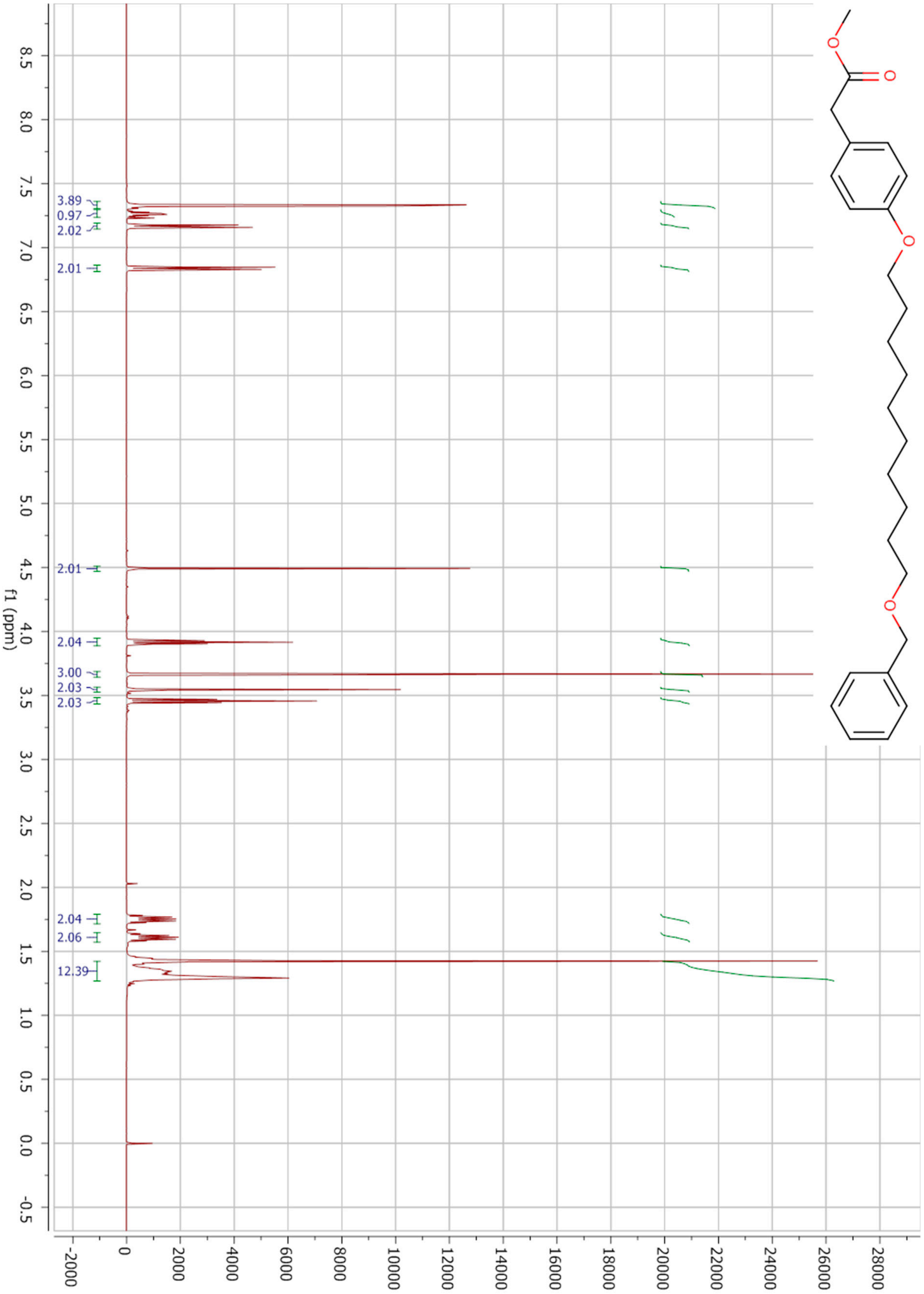

Methyl 2-(4-{[10-(benzyloxy)decyl]oxy}phenyl)acetate (**8i**; ZHAWOC6855)

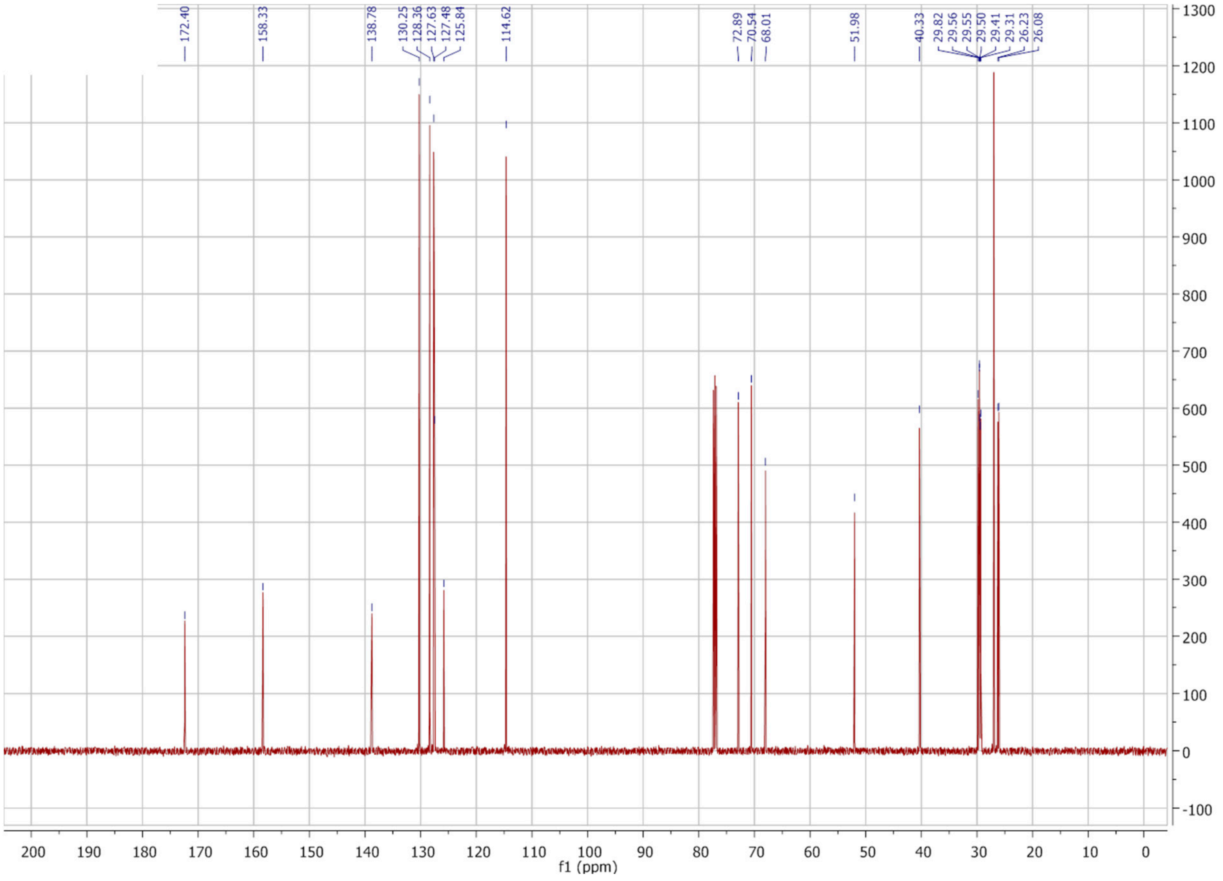

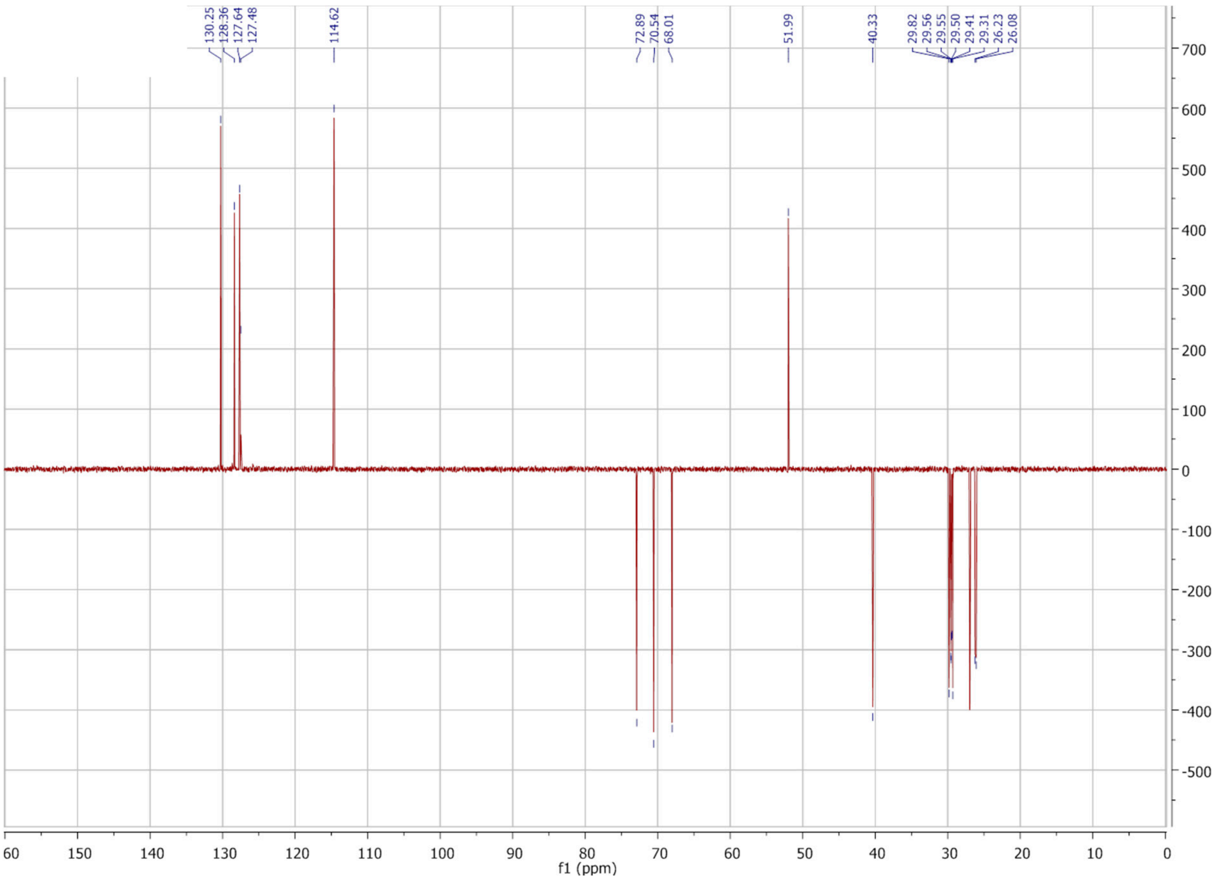

2-{4-[2-(benzyloxy)ethoxy]phenyl}acetic acid (**9a**; ZHAWOC7101)

<u>NMR</u>

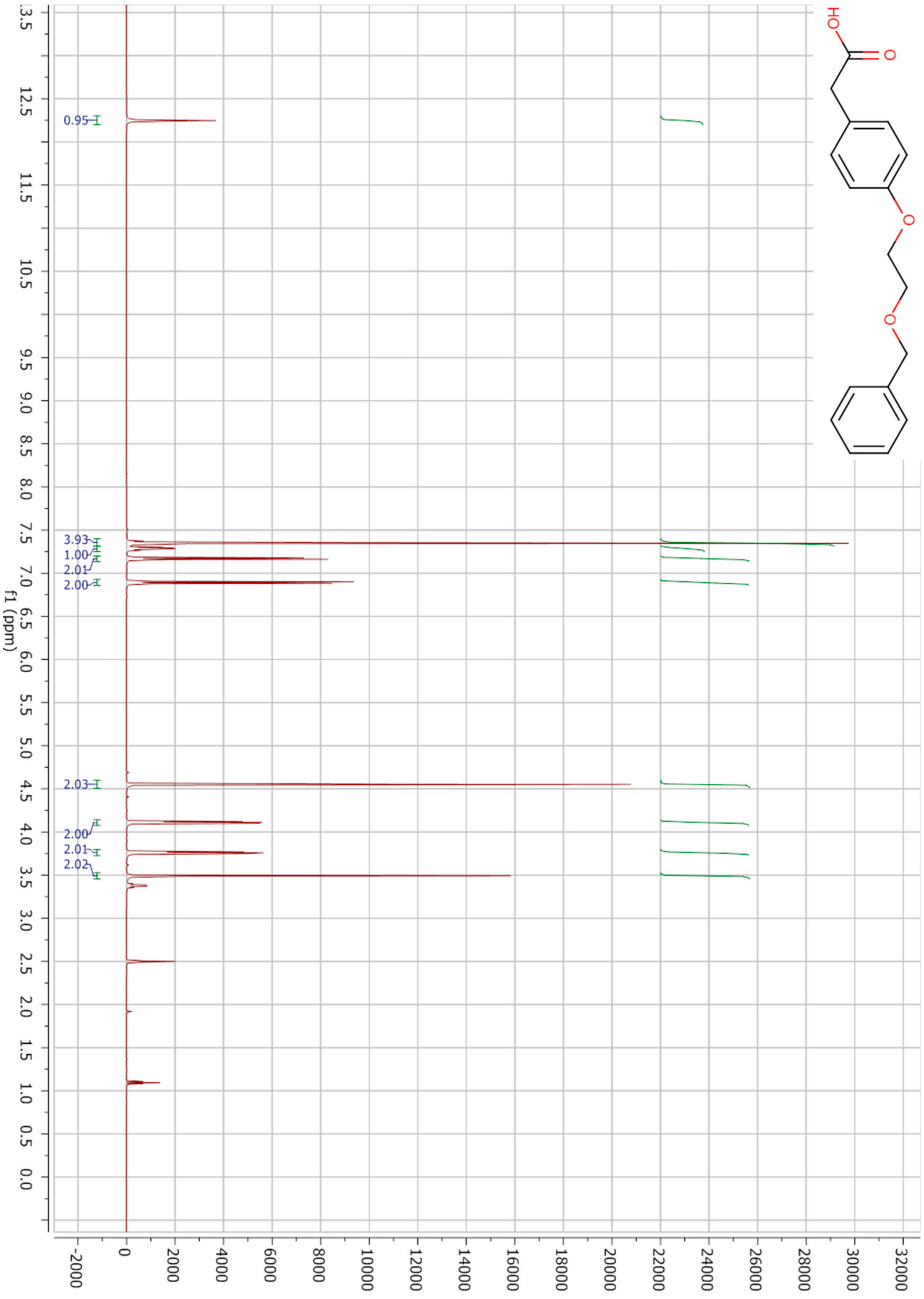

2-{4-[2-(benzyloxy)ethoxy]phenyl}acetic acid (**9a**; ZHAWOC7101)

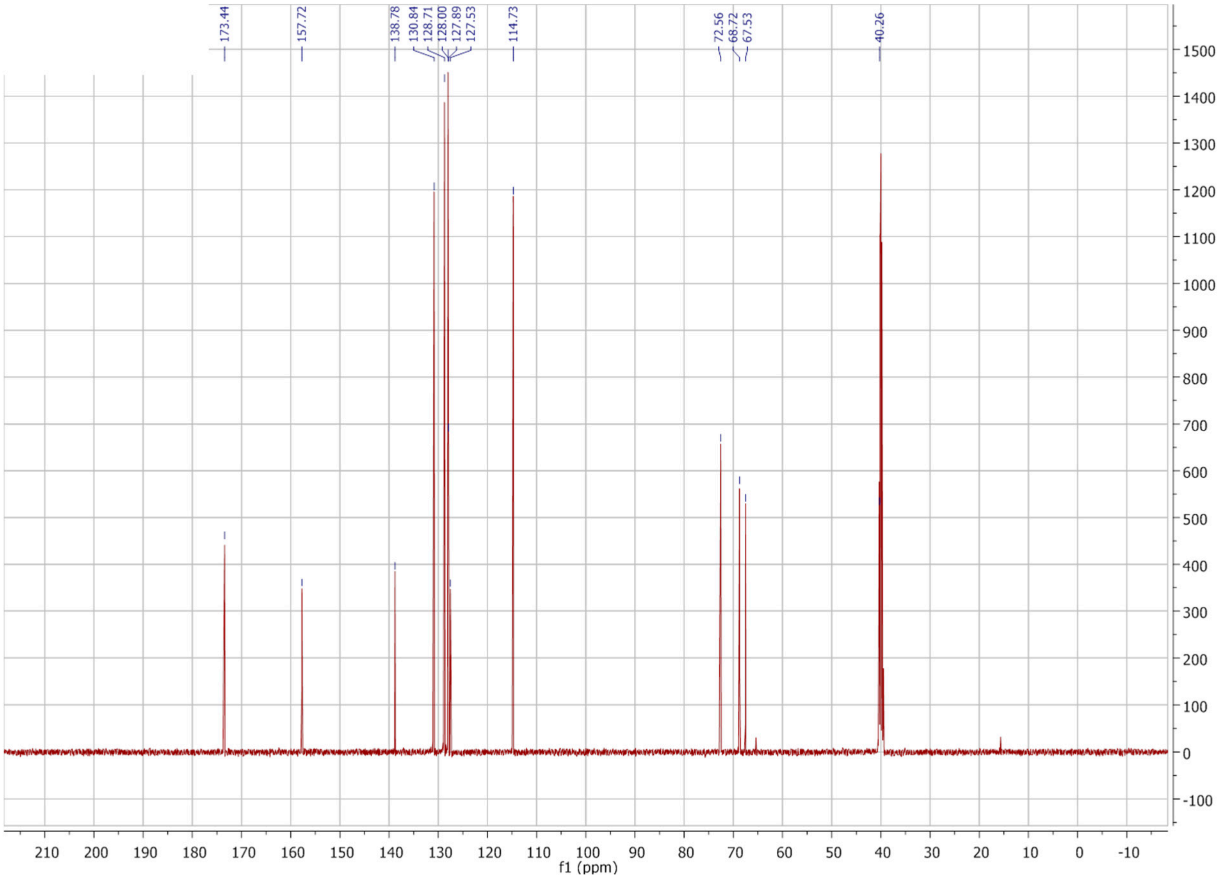

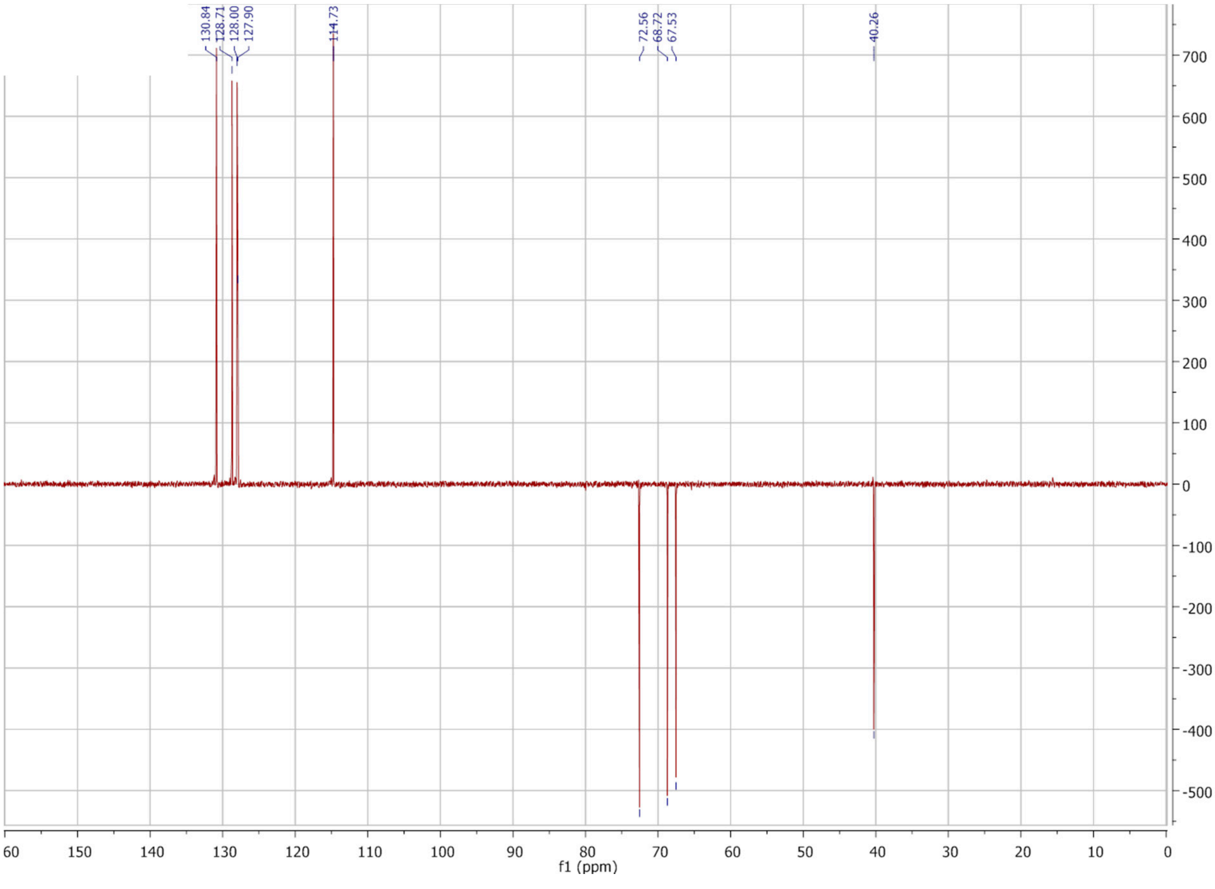

2-{4-[3-(benzyloxy)propoxy]phenyl}acetic acid (**9b;** ZHAWOC4497)

NMR

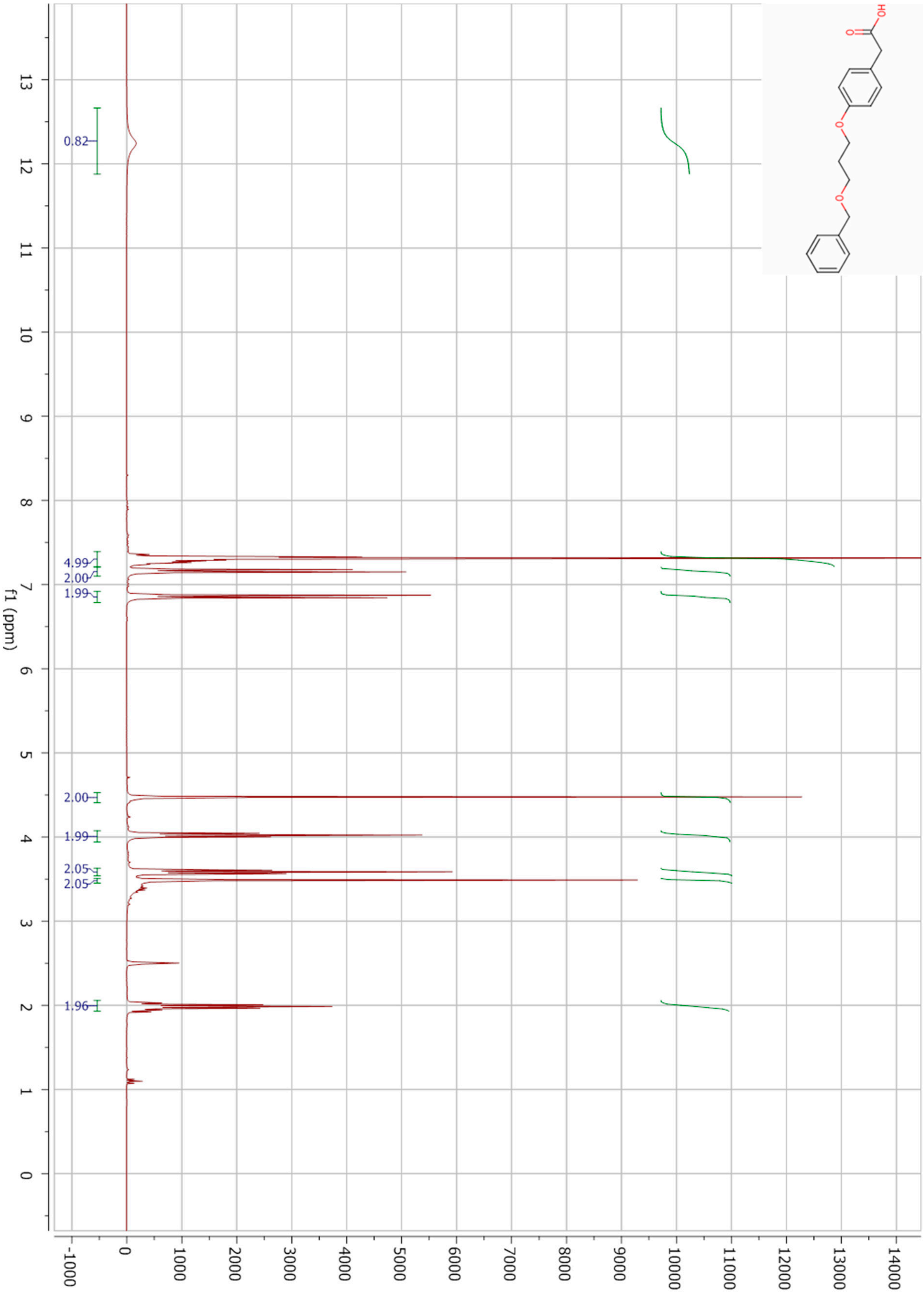

2-{4-[3-(benzyloxy)propoxy]phenyl}acetic acid (**9b;** ZHAWOC4497)

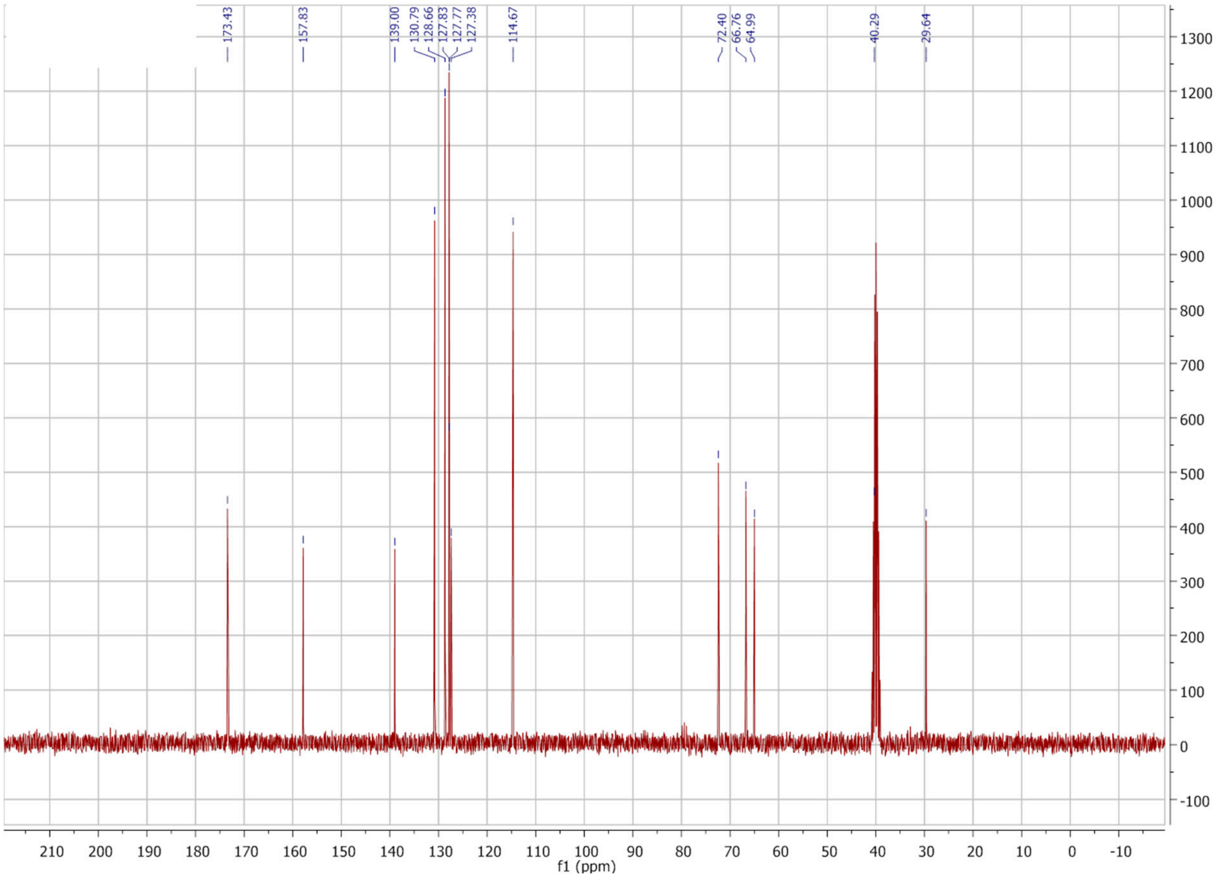

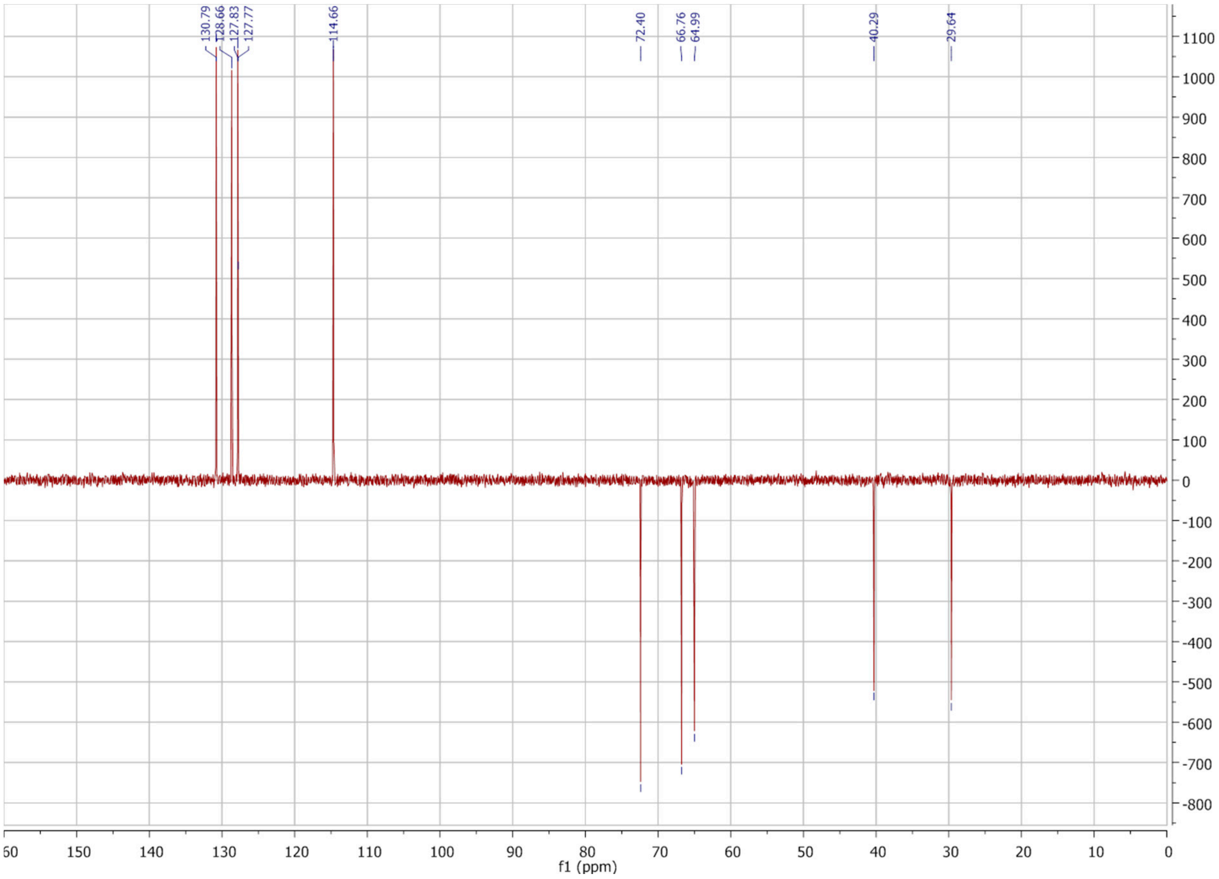

2-{4-[4-(benzyloxy)butoxy]phenyl}acetic acid (**9c;** ZHAWOC4535)

<u>NMR</u>

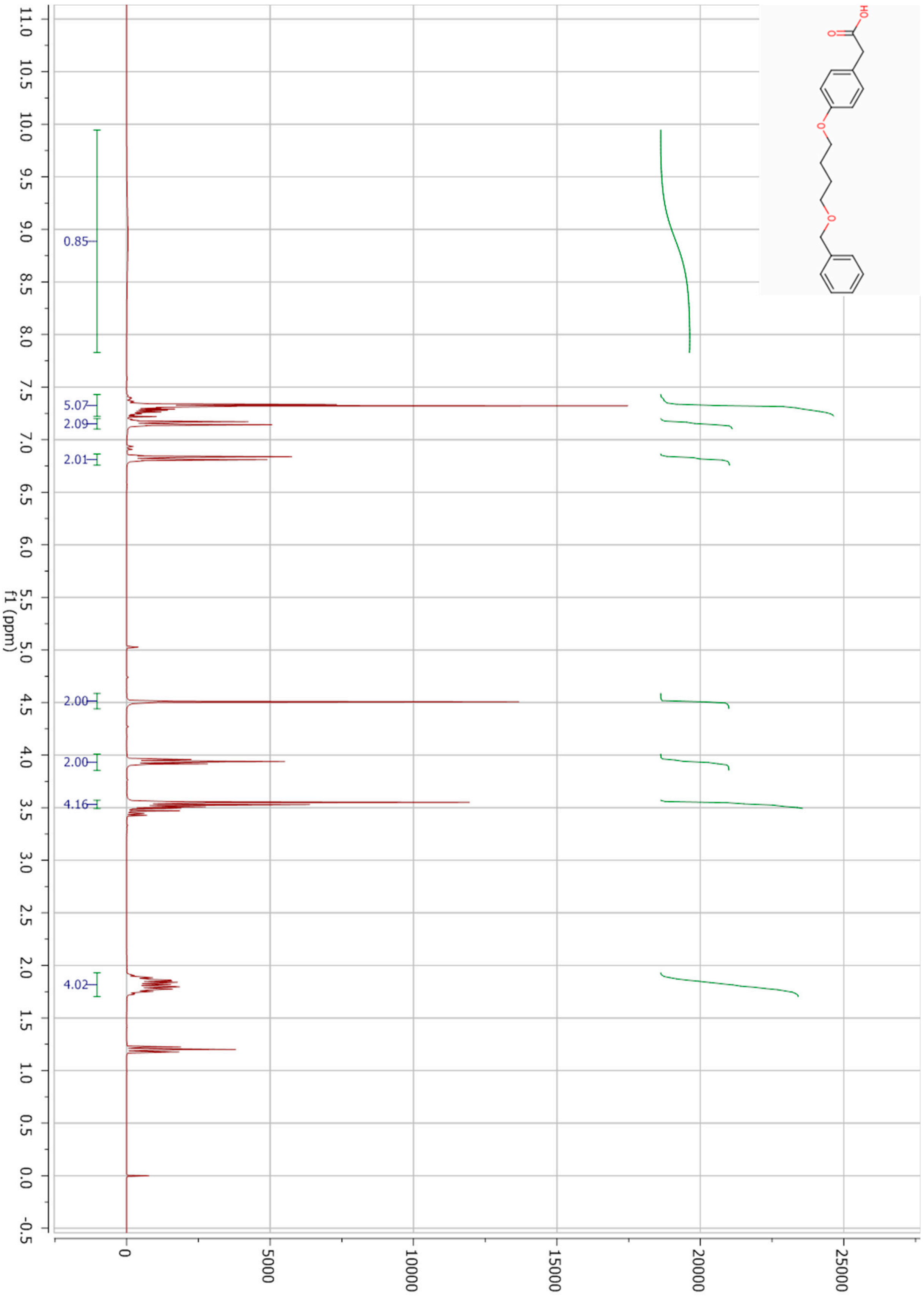

2-{4-[4-(benzyloxy)butoxy]phenyl}acetic acid (**9c;** ZHAWOC4535)

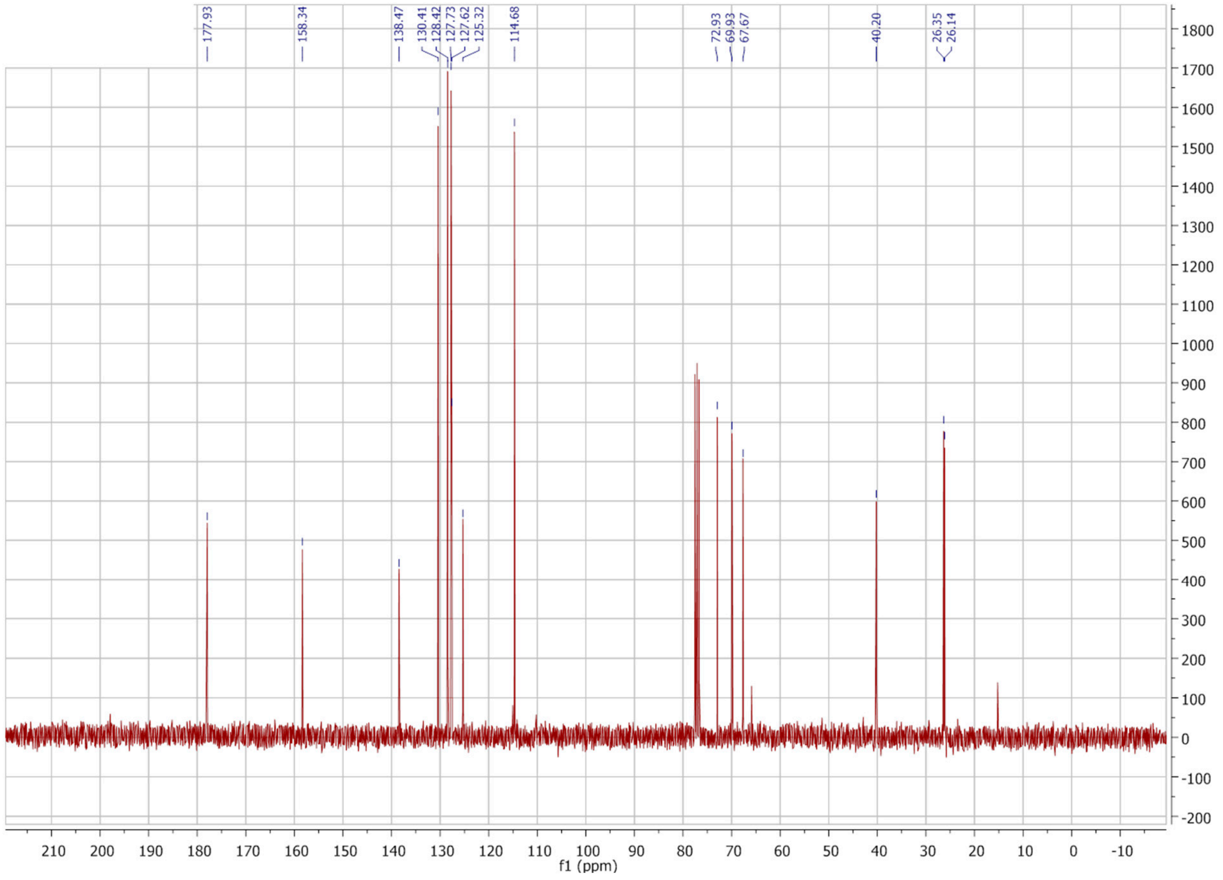

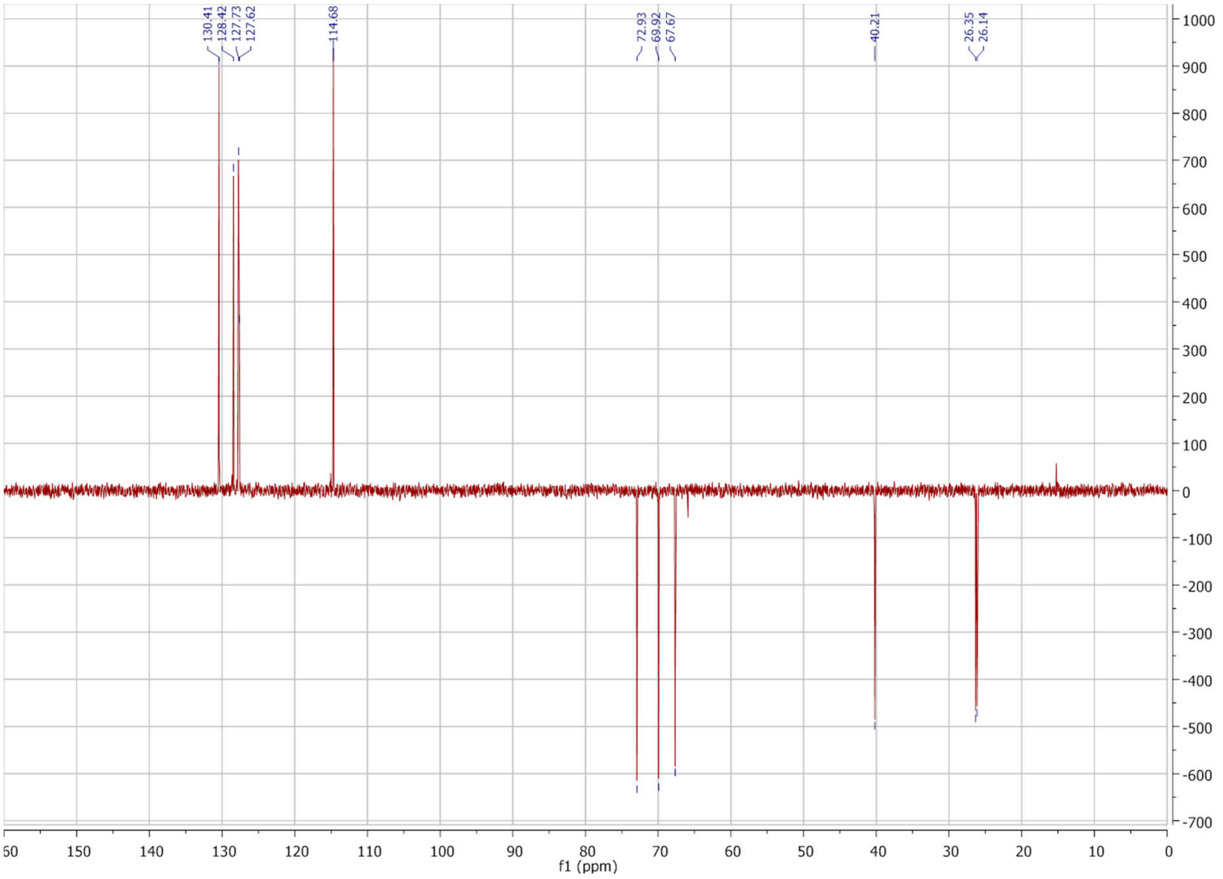

2-(4-{[5-(benzyloxy)pentyl]oxy}phenyl)acetic acid (**9d;** ZHAWOC5922)

<u>NMR</u>

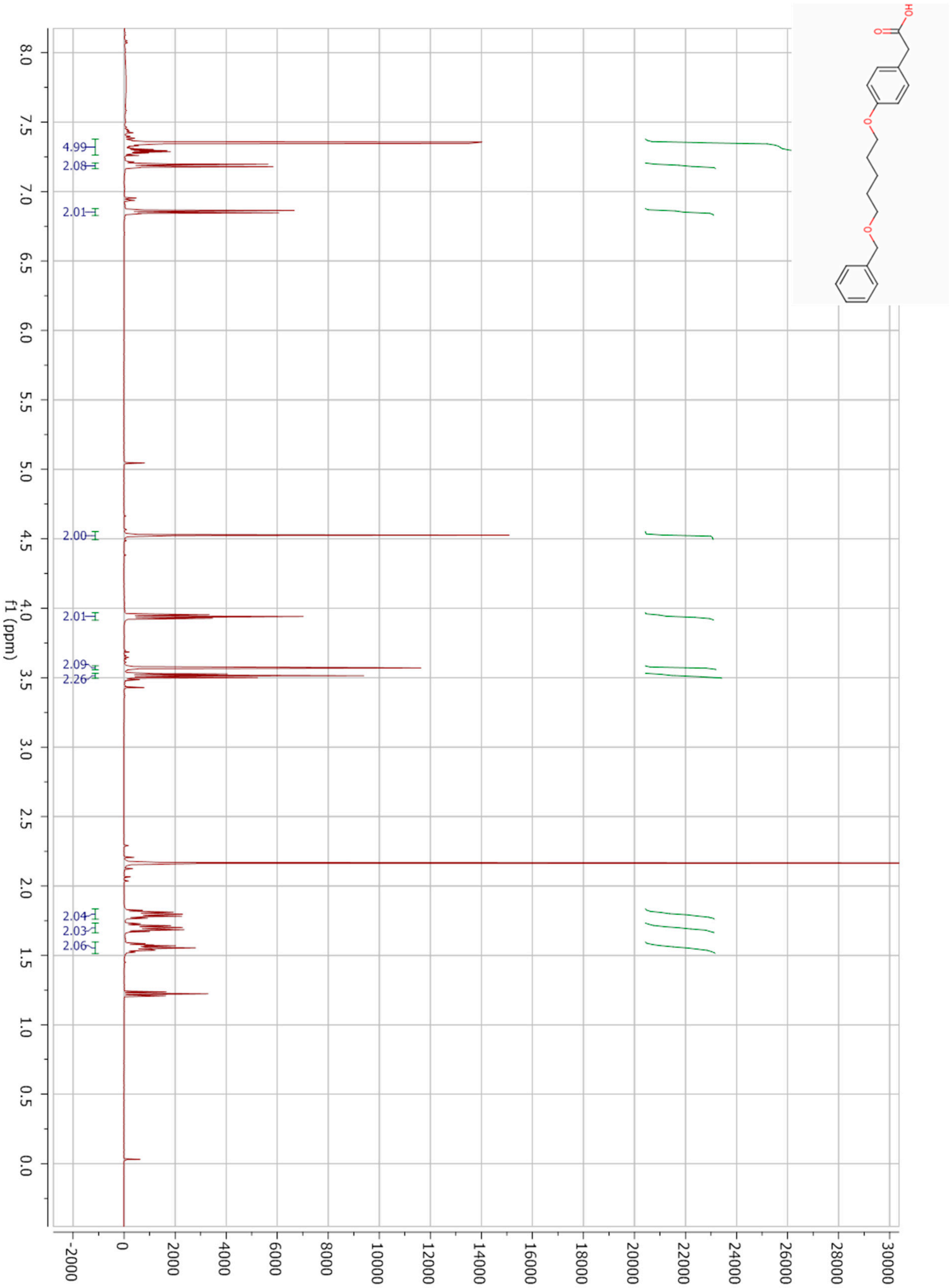

2-(4-{[5-(benzyloxy)pentyl]oxy}phenyl)acetic acid (**9d;** ZHAWOC5922)

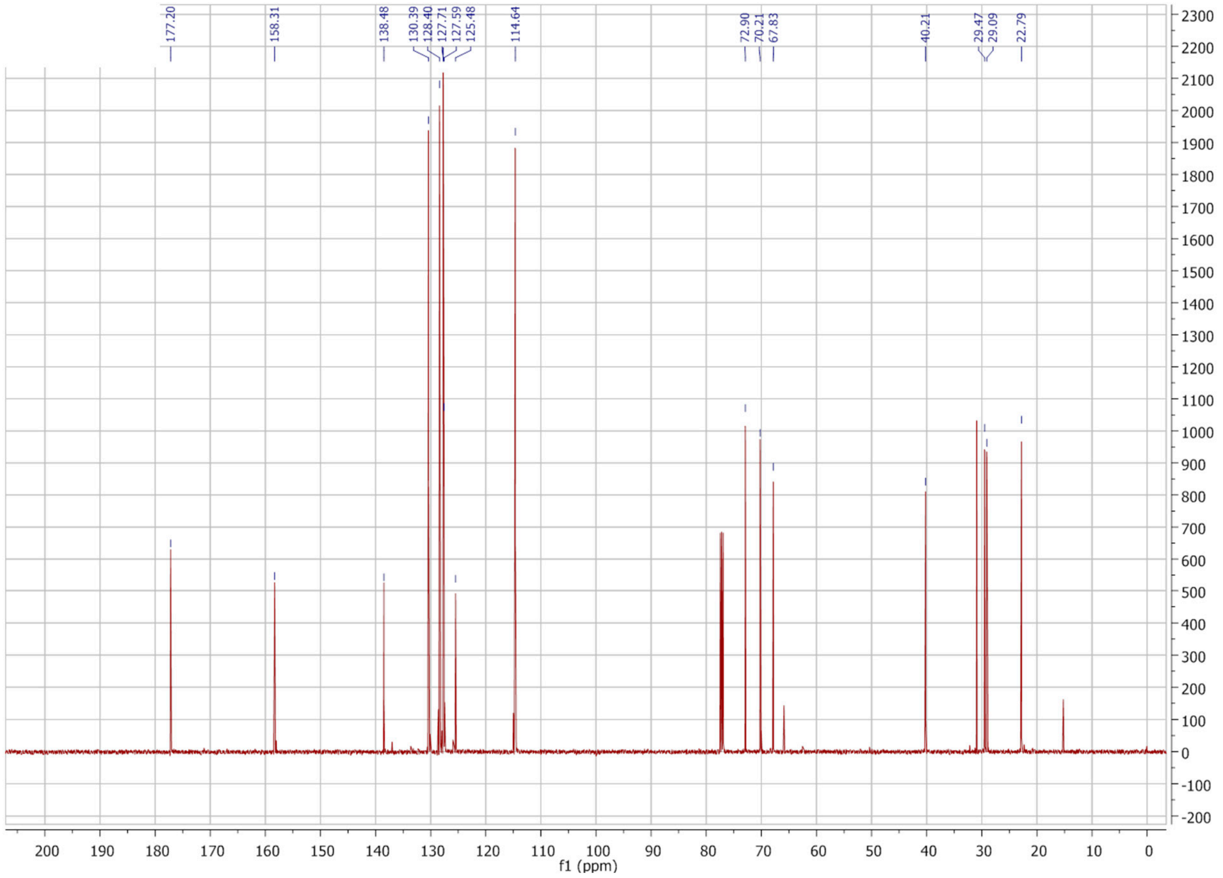

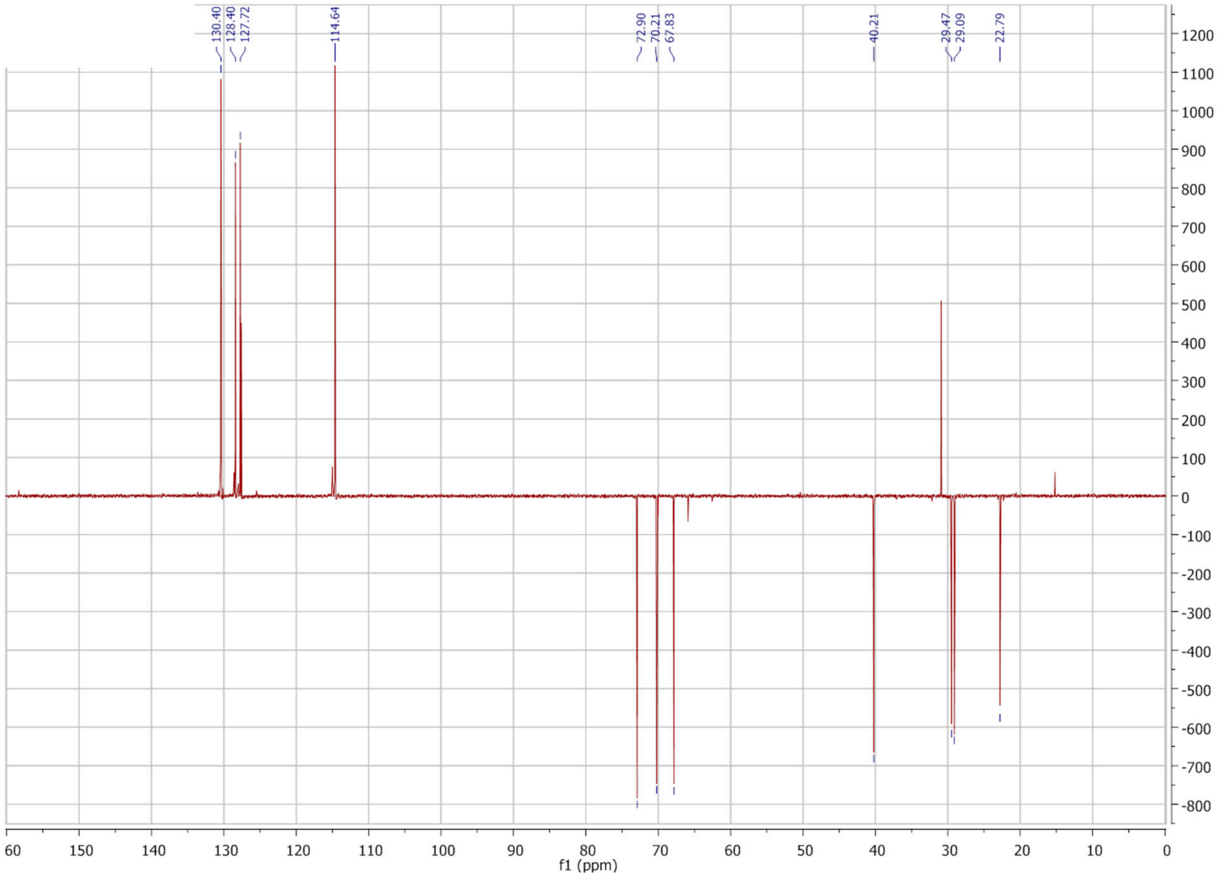

2-(4-{[6-(benzyloxy)hexyl]oxy}phenyl)acetic acid (**9e;** ZHAWOC5947)

NMR

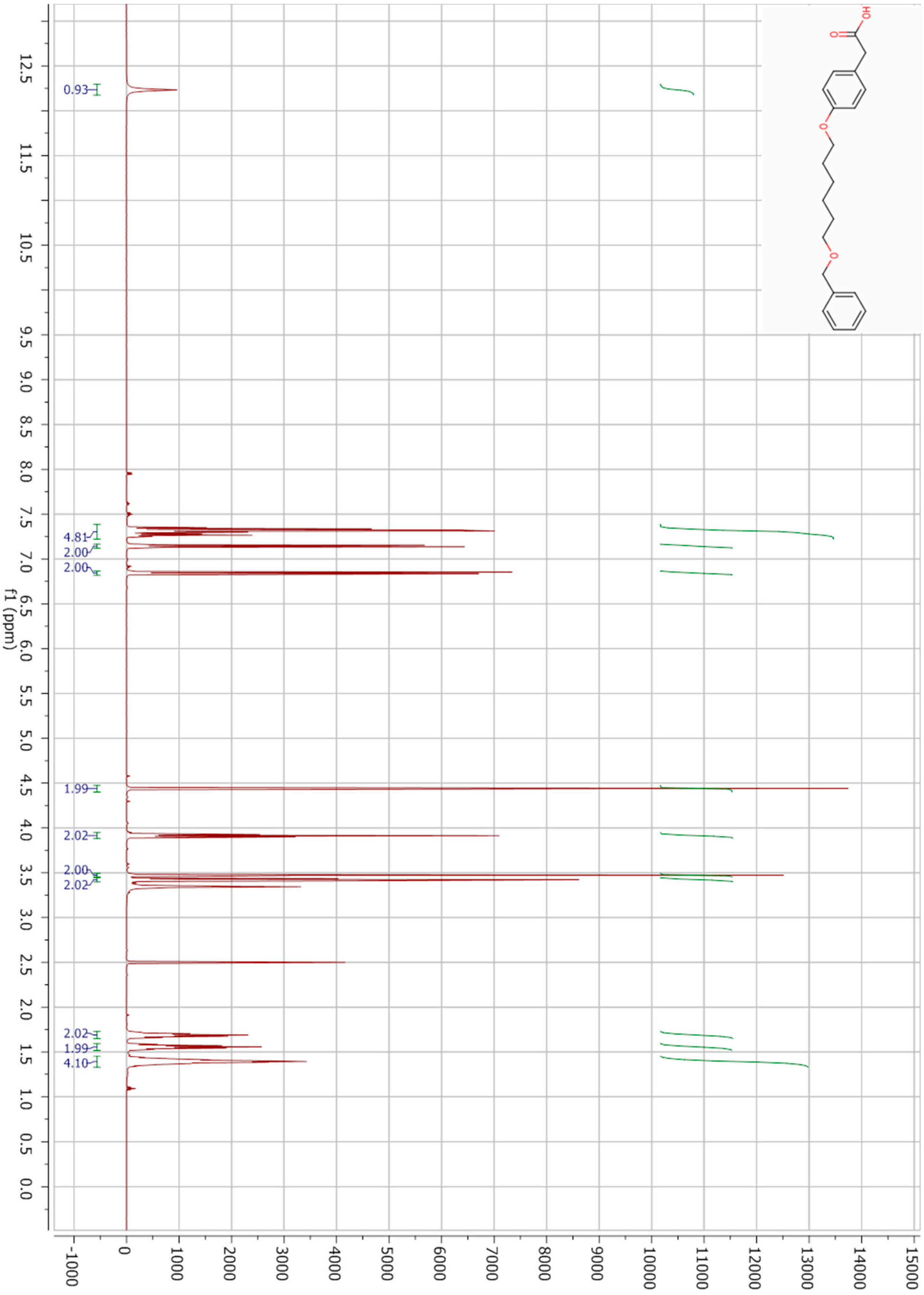

2-(4-{[6-(benzyloxy)hexyl]oxy}phenyl)acetic acid (**9e;** ZHAWOC5947)

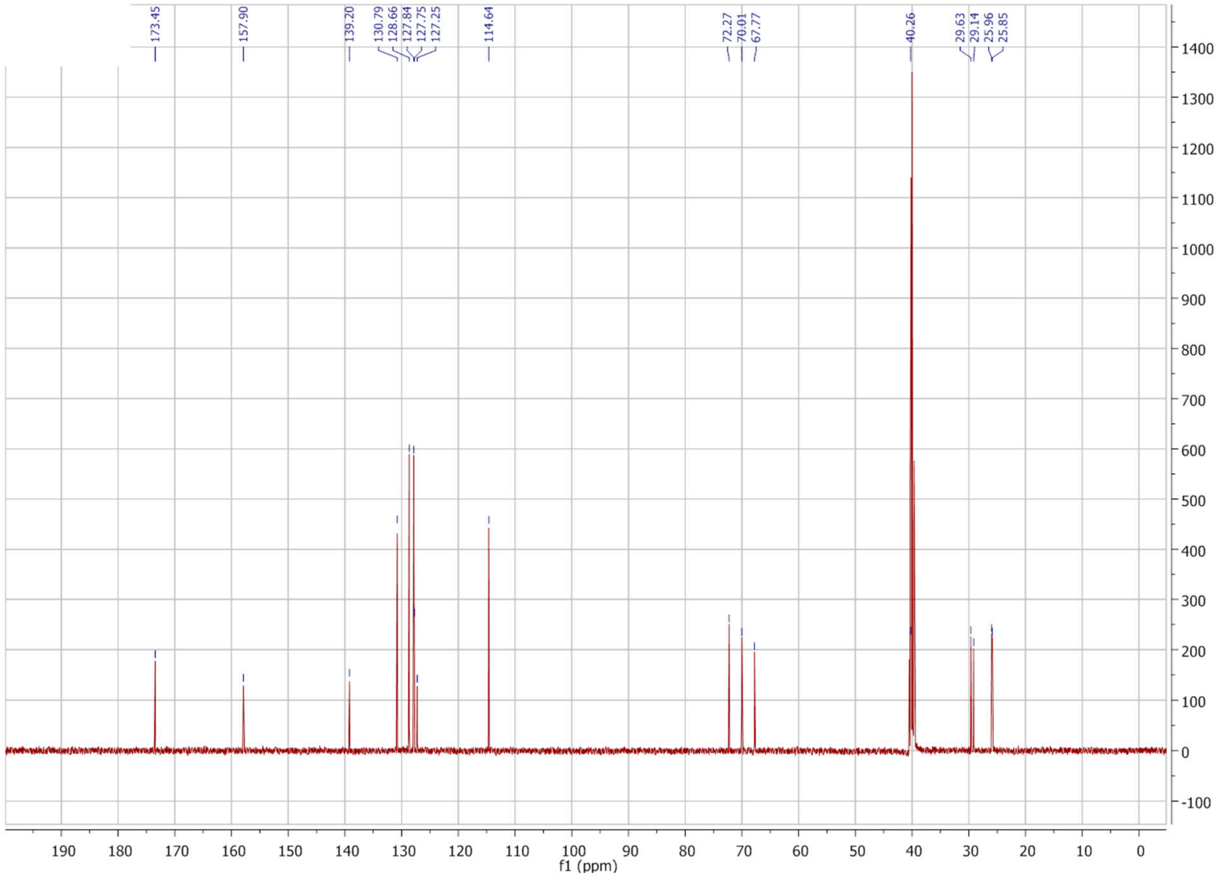

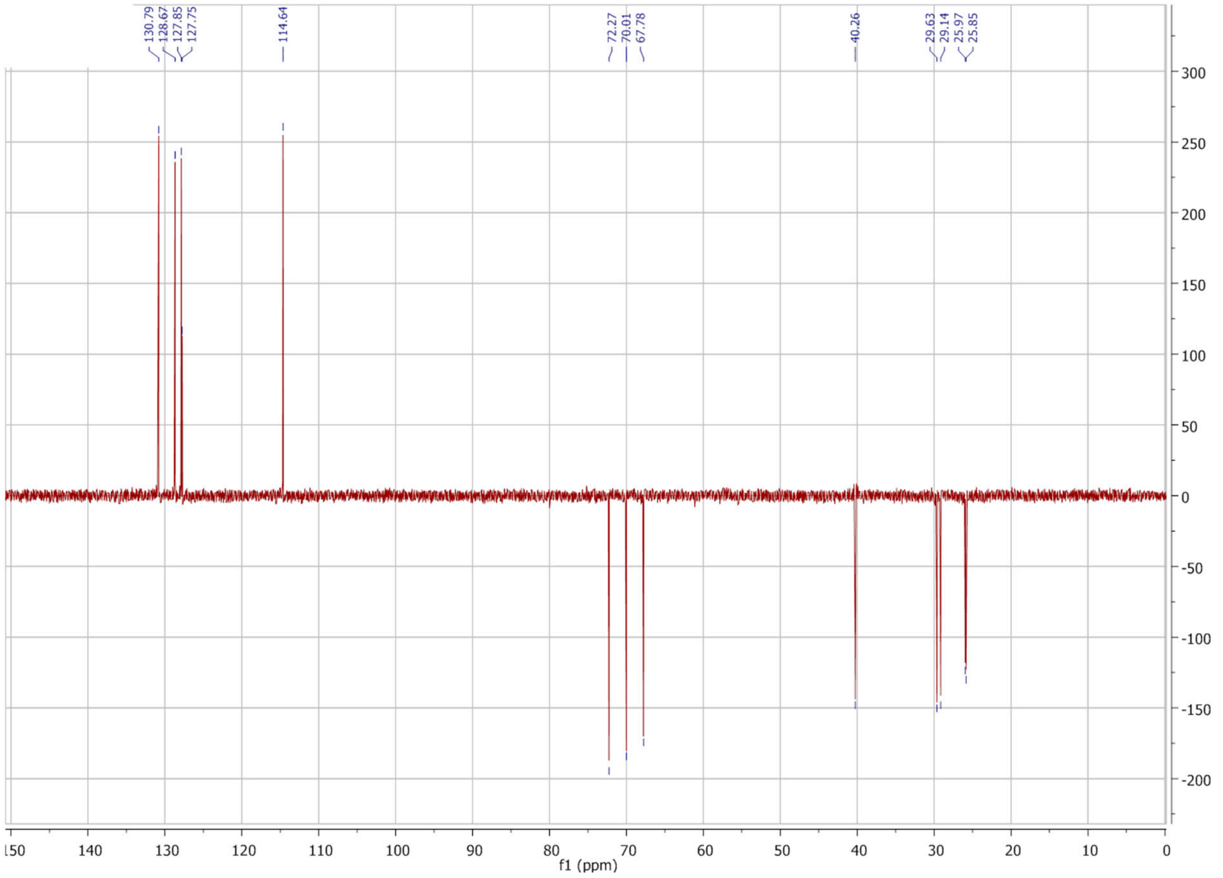

2-(4-{[7-(benzyloxy)heptyl]oxy}phenyl)acetic acid (**9f**; ZHAWOC7098)

NMR

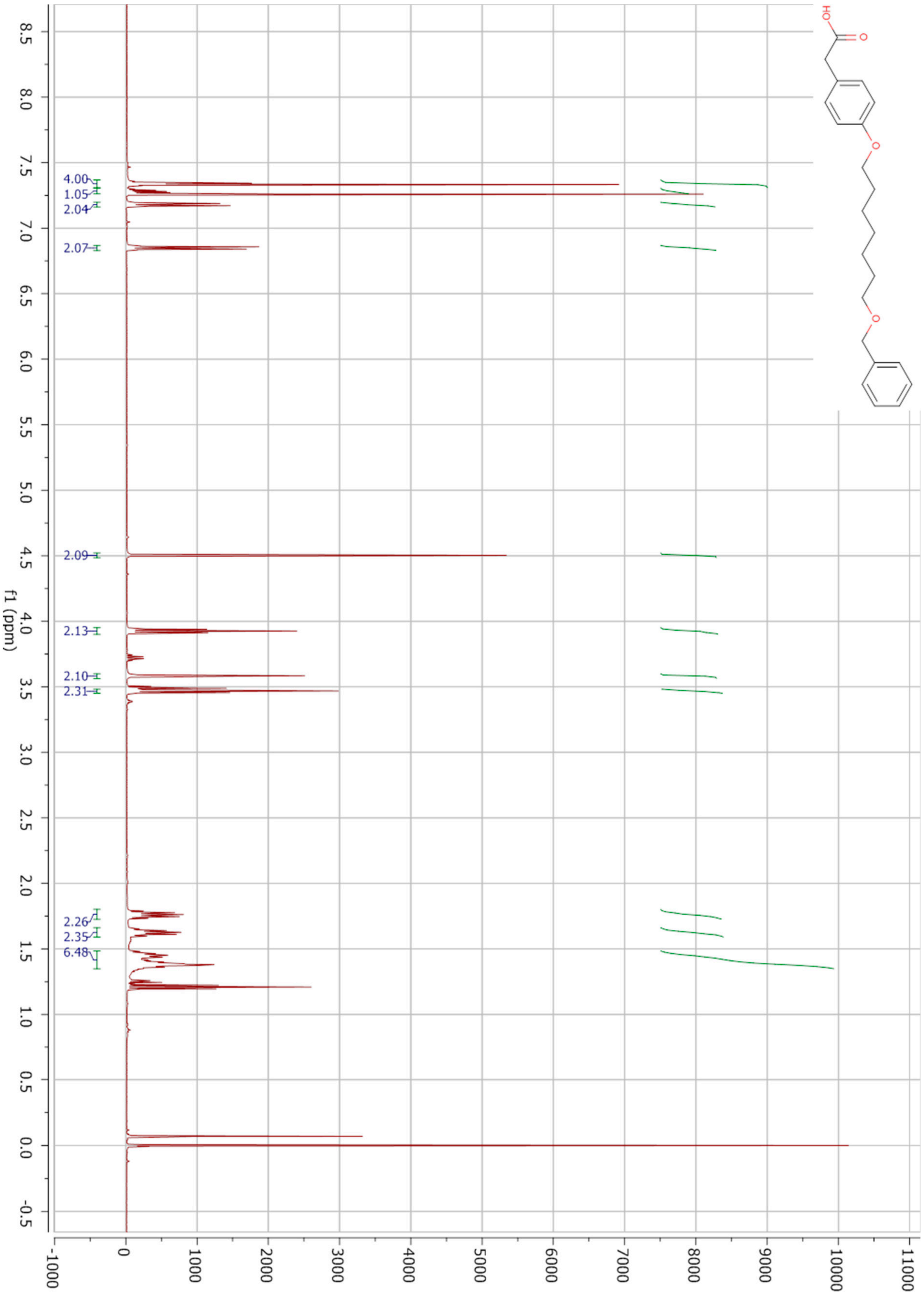

2-(4-{[7-(benzyloxy)heptyl]oxy}phenyl)acetic acid (**9f**; ZHAWOC7098)

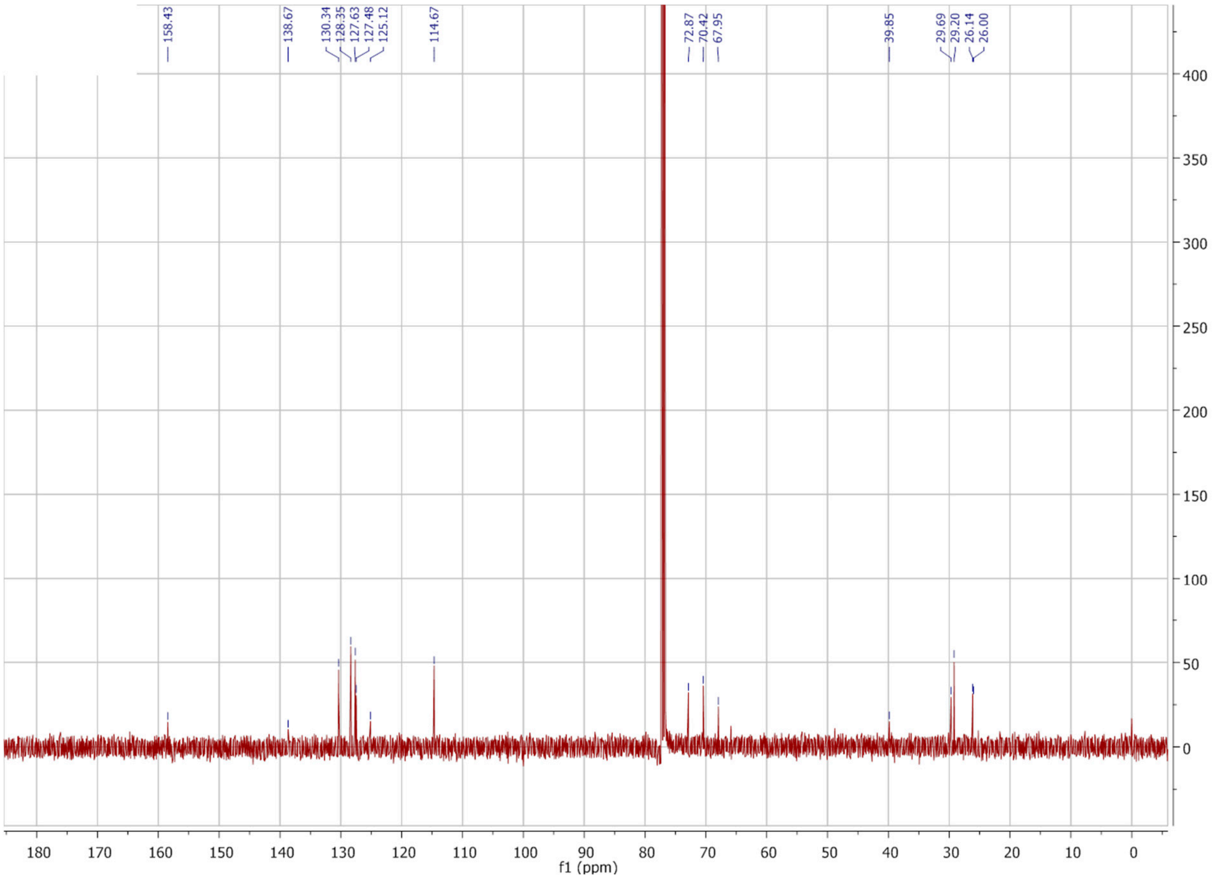

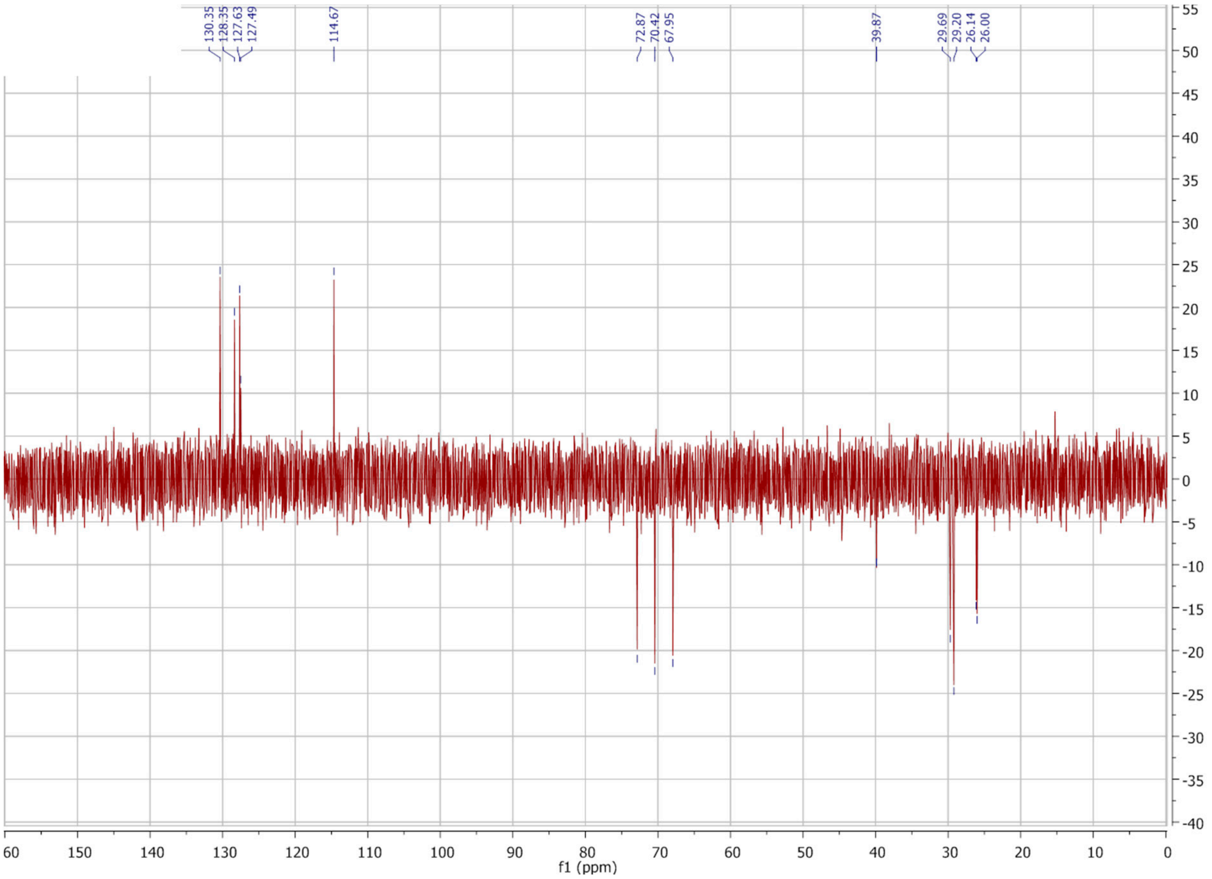

2-(4-{[8-(benzyloxy)octyl]oxy}phenyl)acetic acid (**9g**; ZHAWOC6858)

<u>NMR</u>

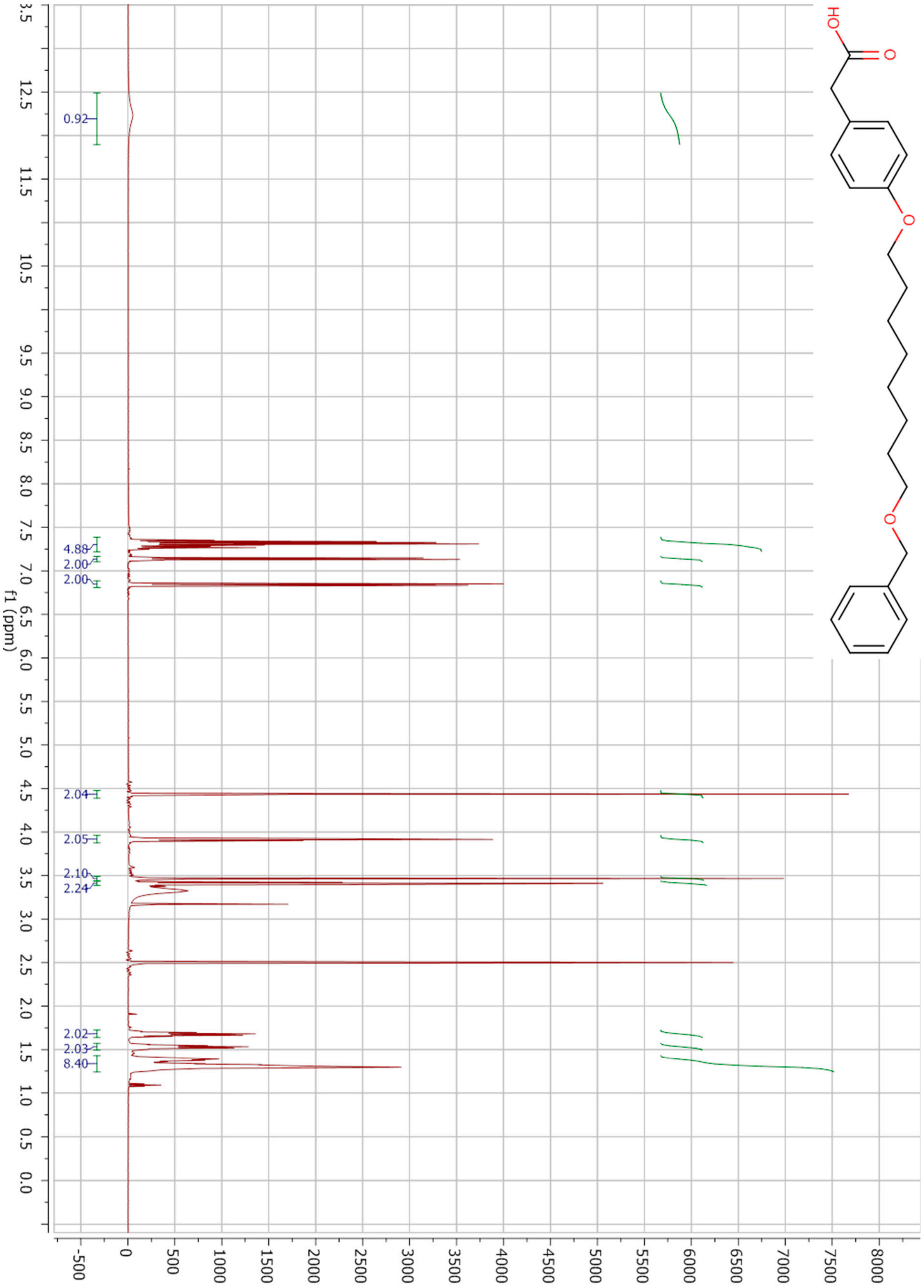

2-(4-{[8-(benzyloxy)octyl]oxy}phenyl)acetic acid (**9g**; ZHAWOC6858)

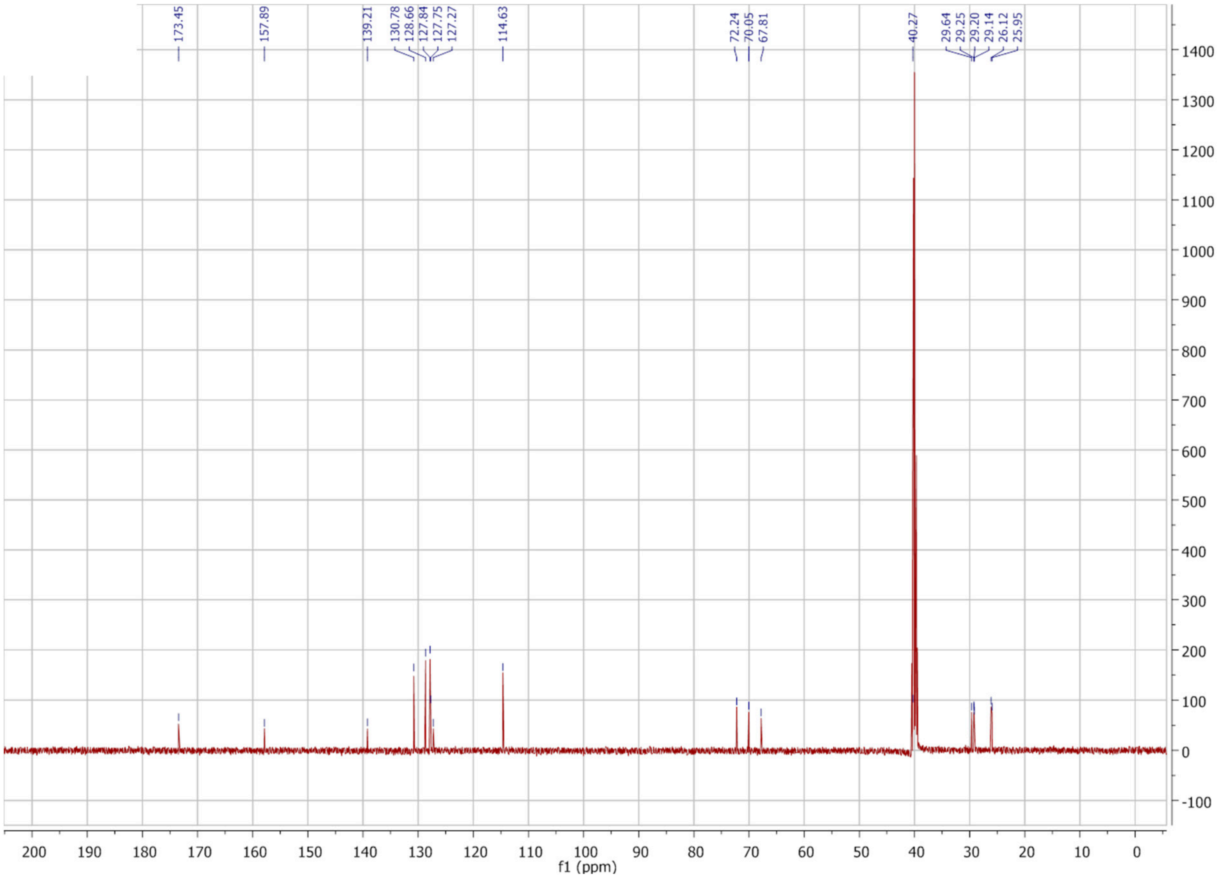

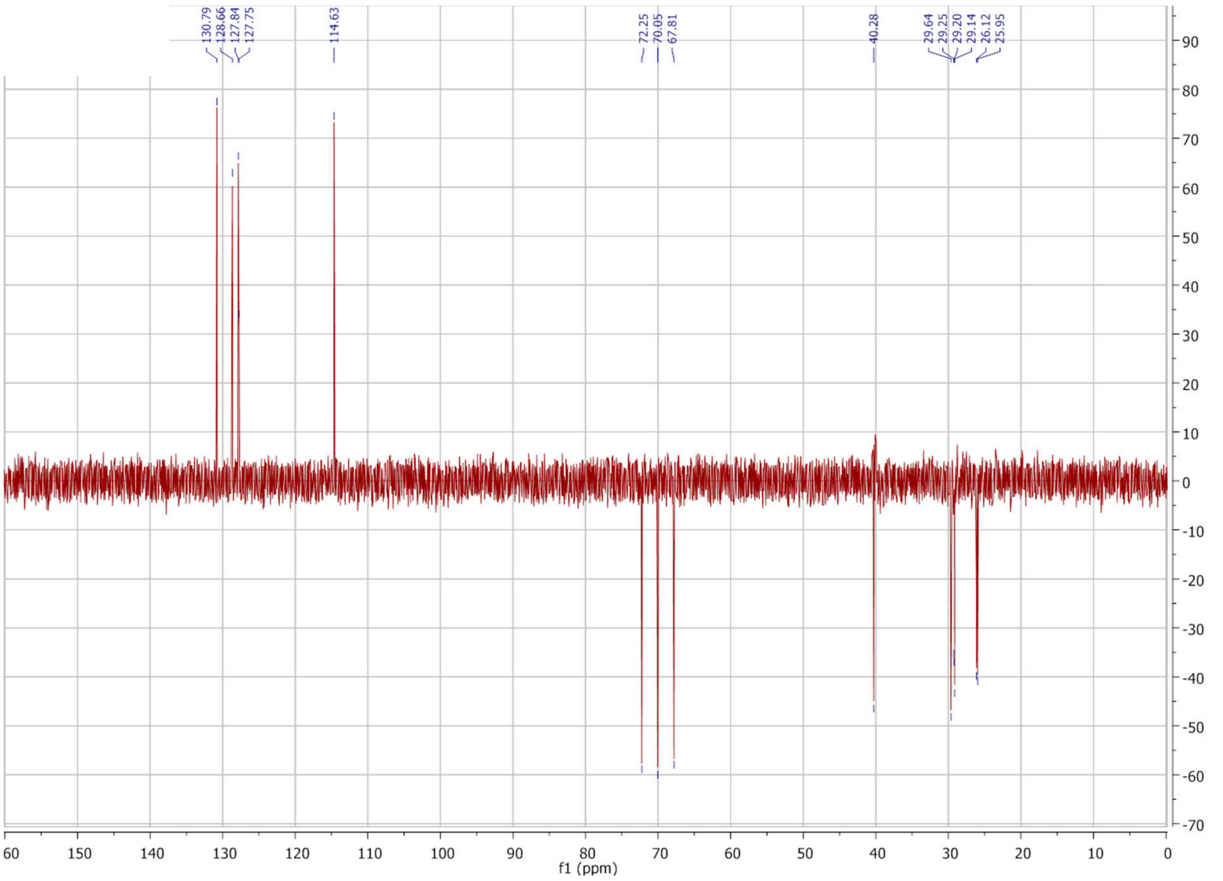

2-(4-{[9-(benzyloxy)nonyl]oxy}phenyl)acetic acid (**9h**; ZHAWOC6861)

<u>NMR</u>

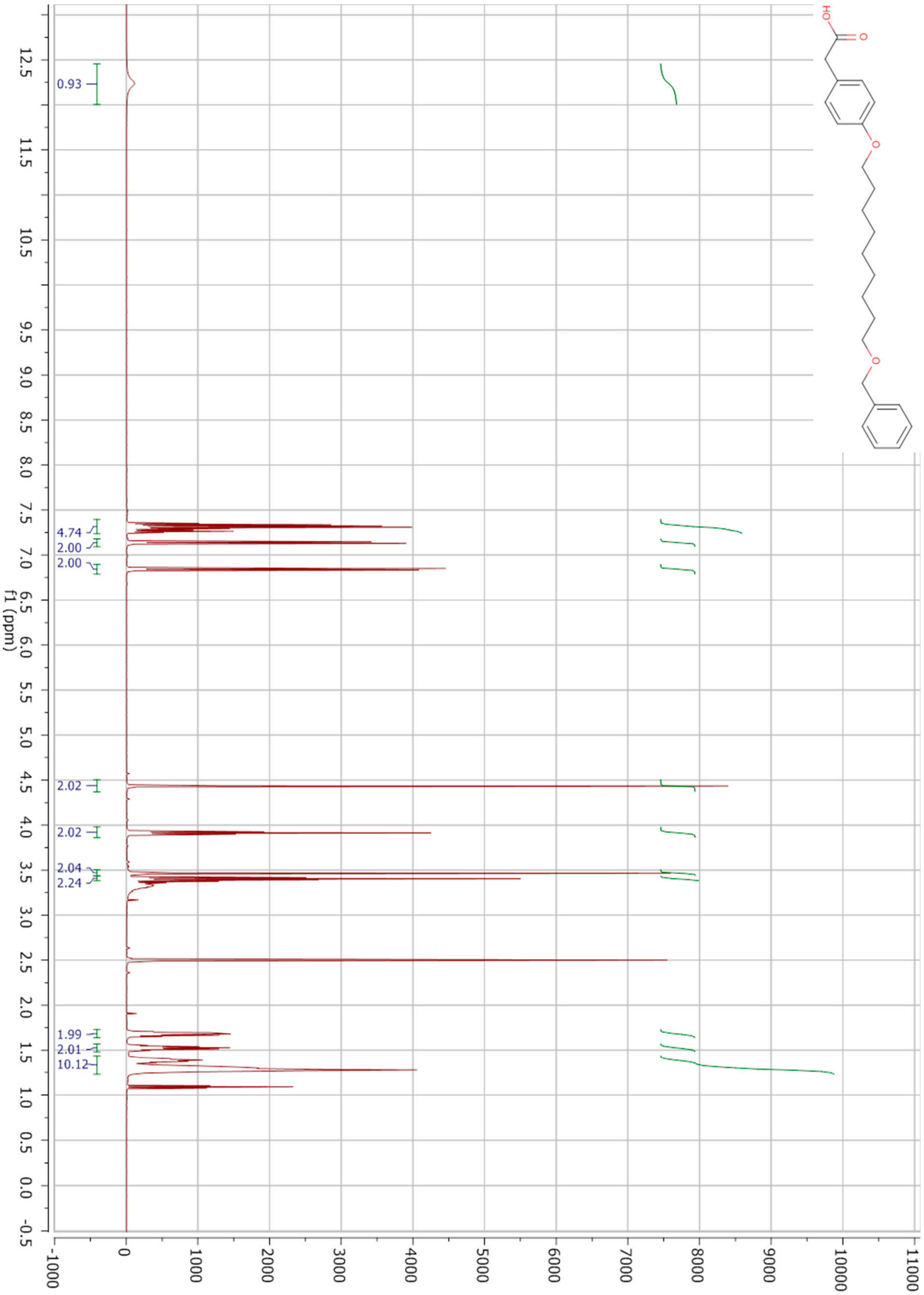

2-(4-{[9-(benzyloxy)nonyl]oxy}phenyl)acetic acid (**9h**; ZHAWOC6861)

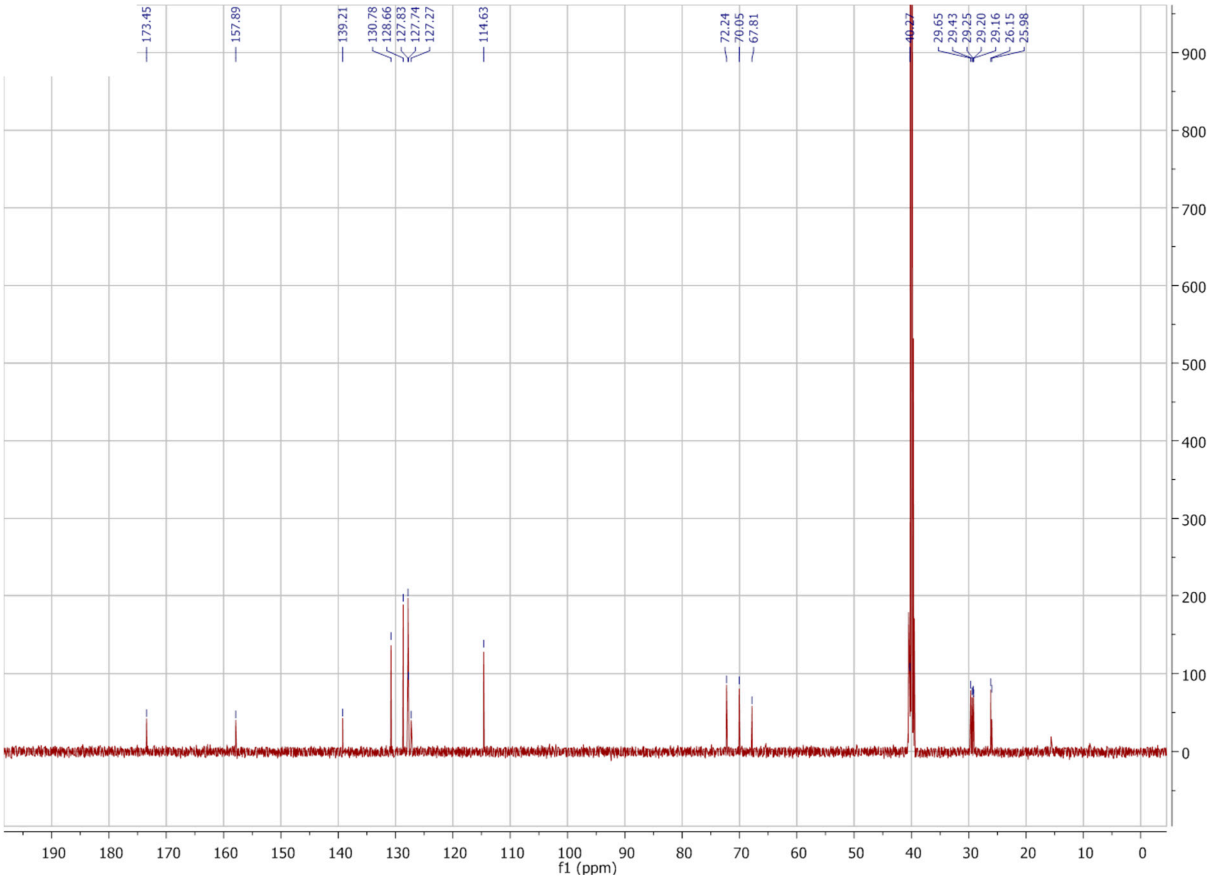

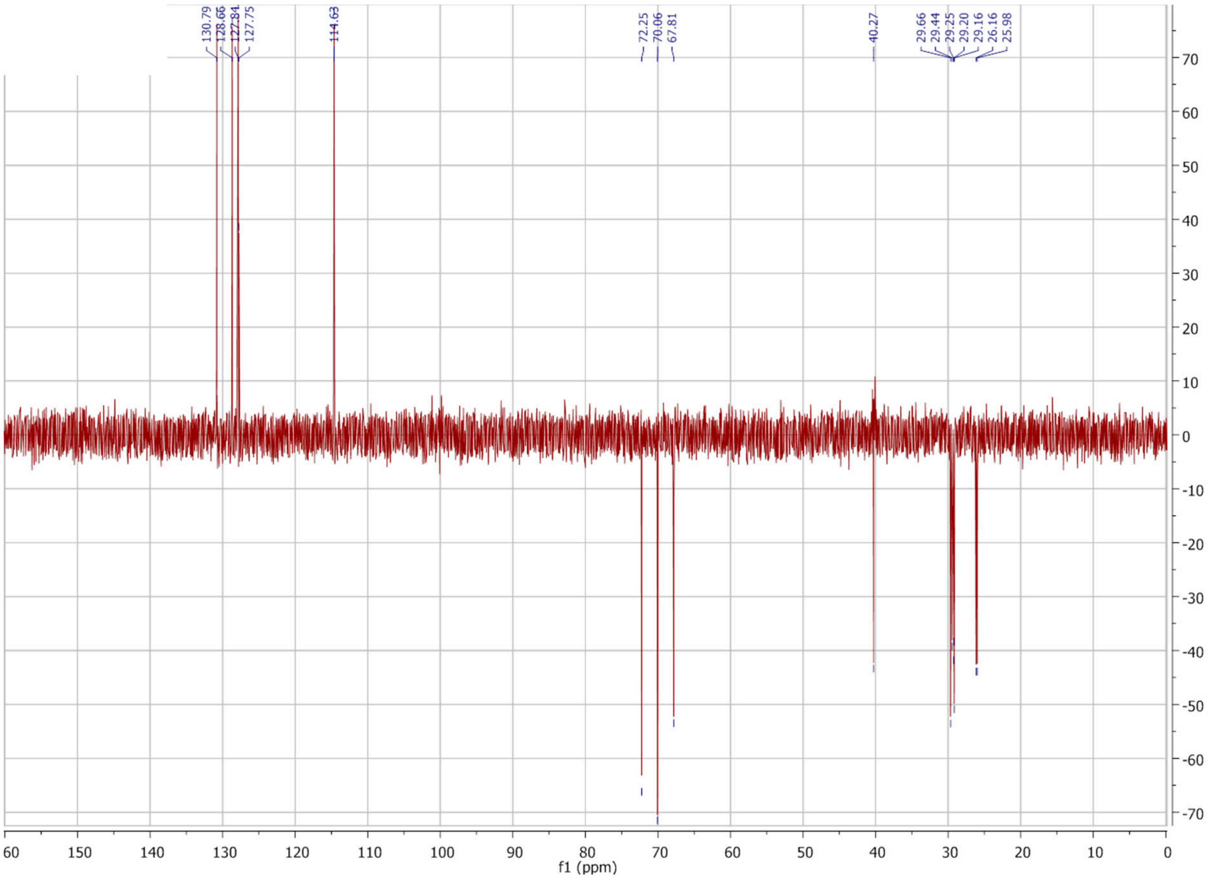

2-(4-{[10-(benzyloxy)decyl]oxy}phenyl)acetic acid (**9i**; ZHAWOC6862)

<u>NMR</u>

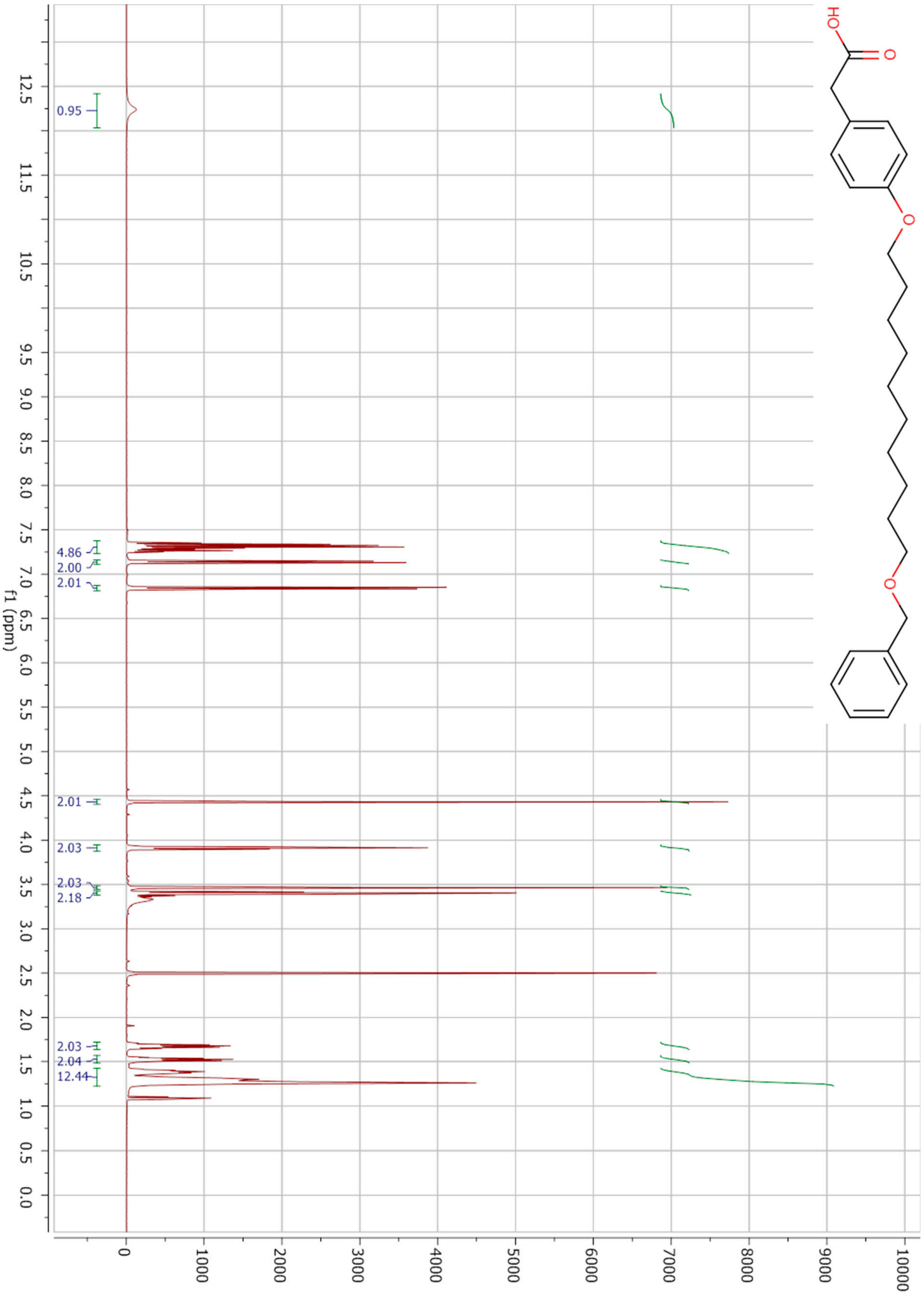

2-(4-{[10-(benzyloxy)decyl]oxy}phenyl)acetic acid (**9i**; ZHAWOC6862)

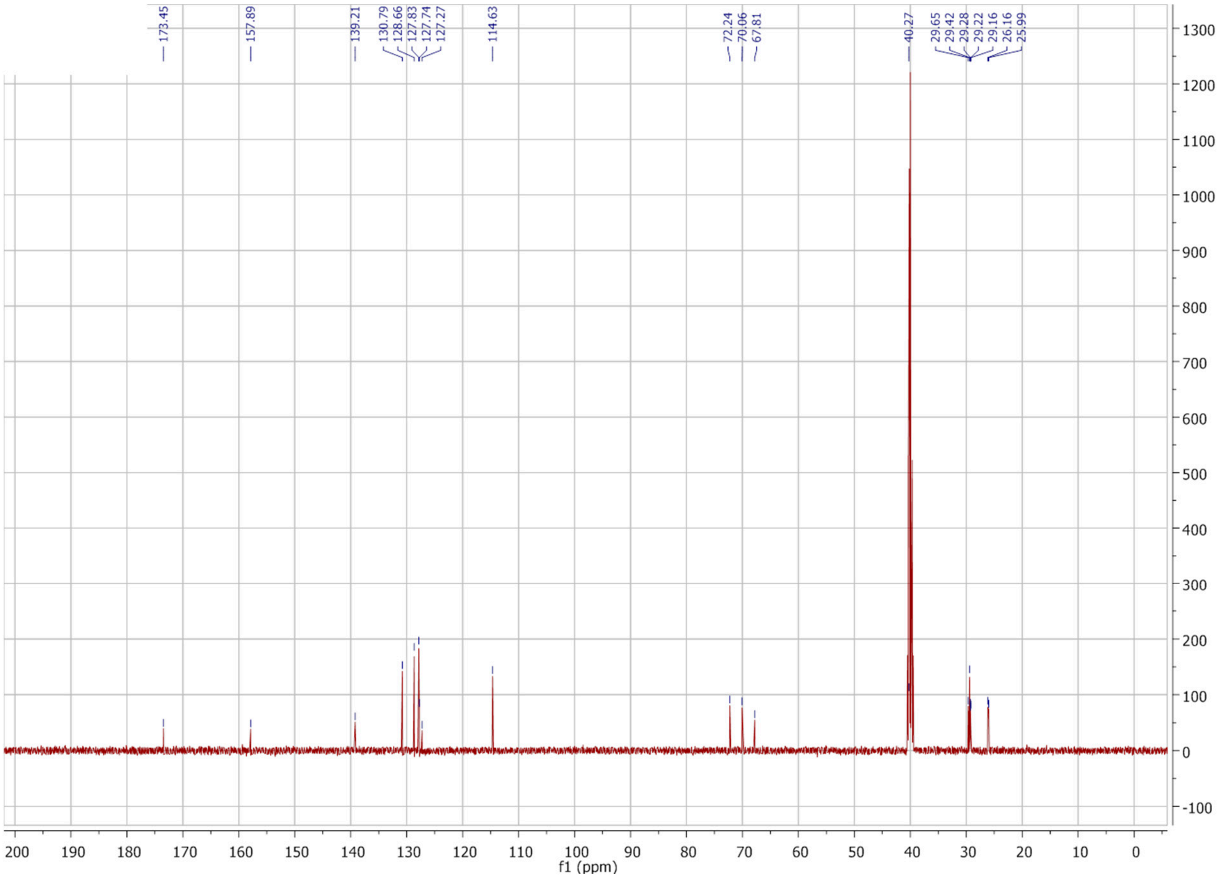

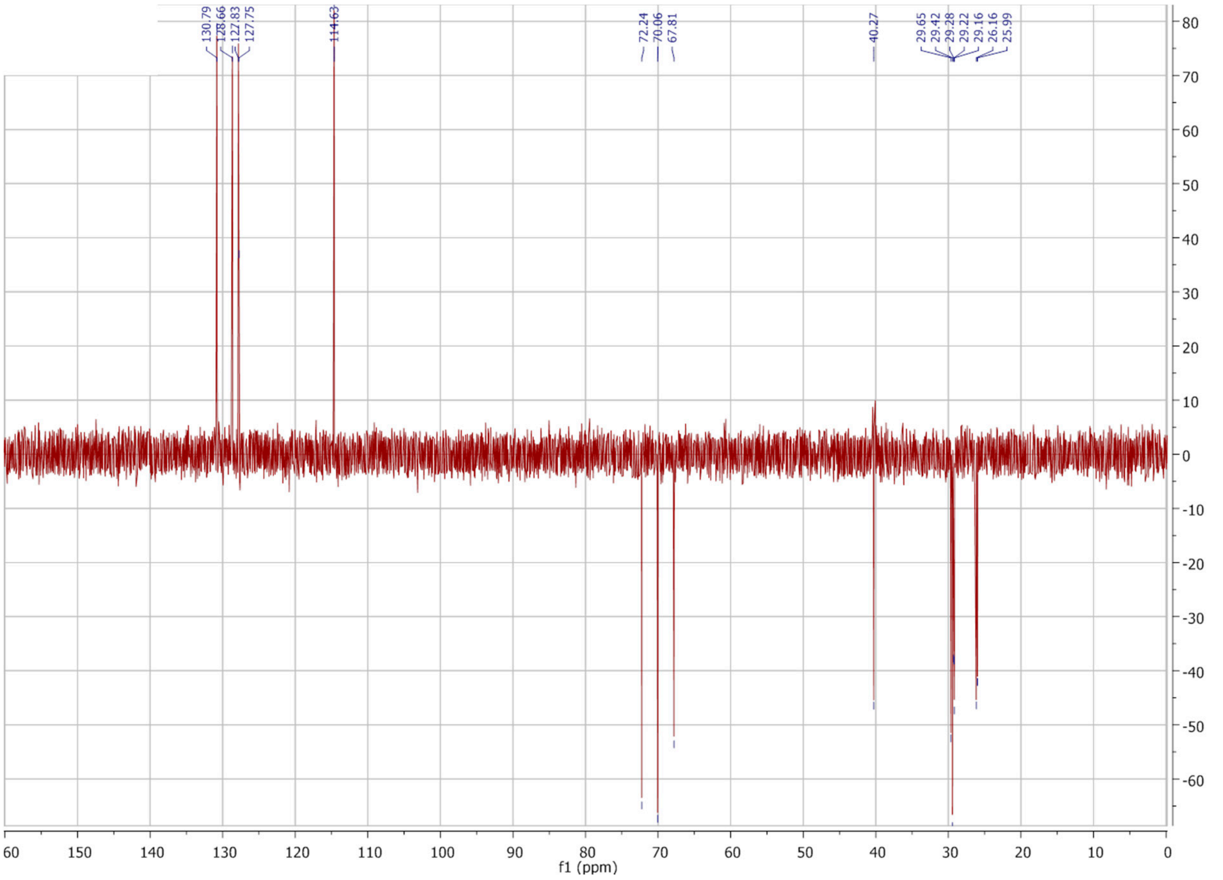

2-(4-{[5-(benzyloxy)pentyl]oxy}phenyl)-N-(2-methyl-1,3-dioxo-2,3-dihydro-1H-isoindol-5-yl)acetamide (**12a**; ZHAWOC6647)

NMR

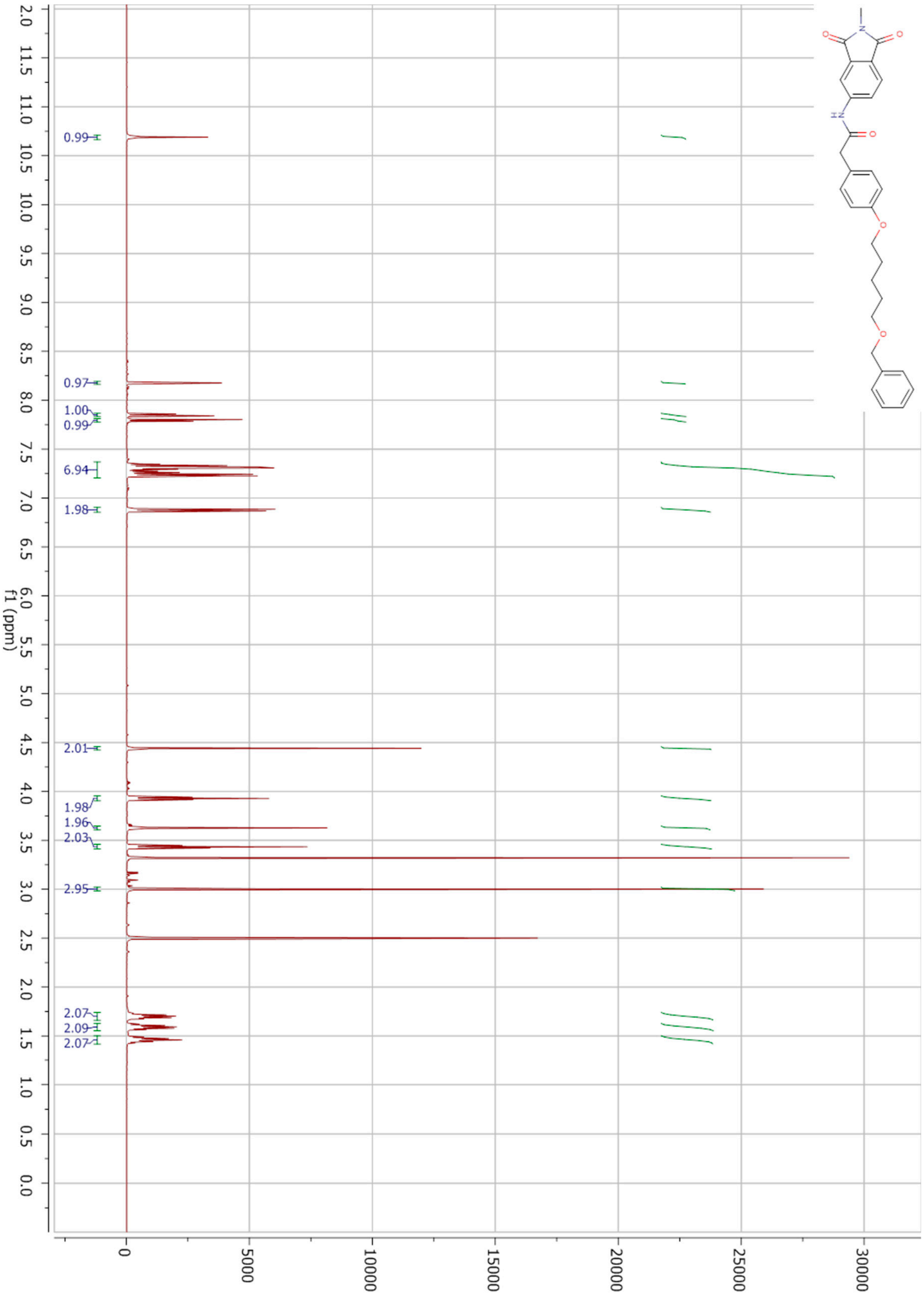

2-(4-{[5-(benzyloxy)pentyl]oxy}phenyl)-N-(2-methyl-1,3-dioxo-2,3-dihydro-1H-isoindol-5-yl)acetamide (**12a**; ZHAWOC6647)

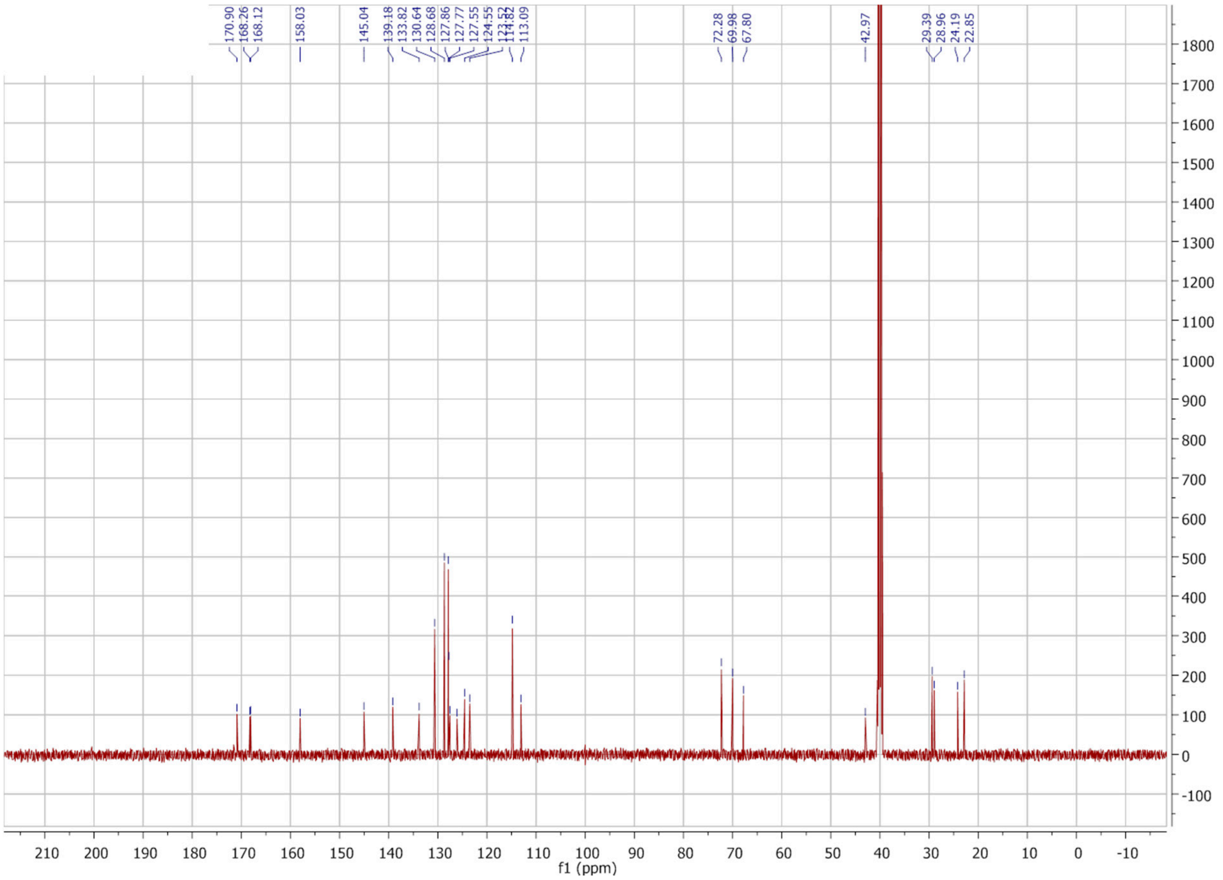

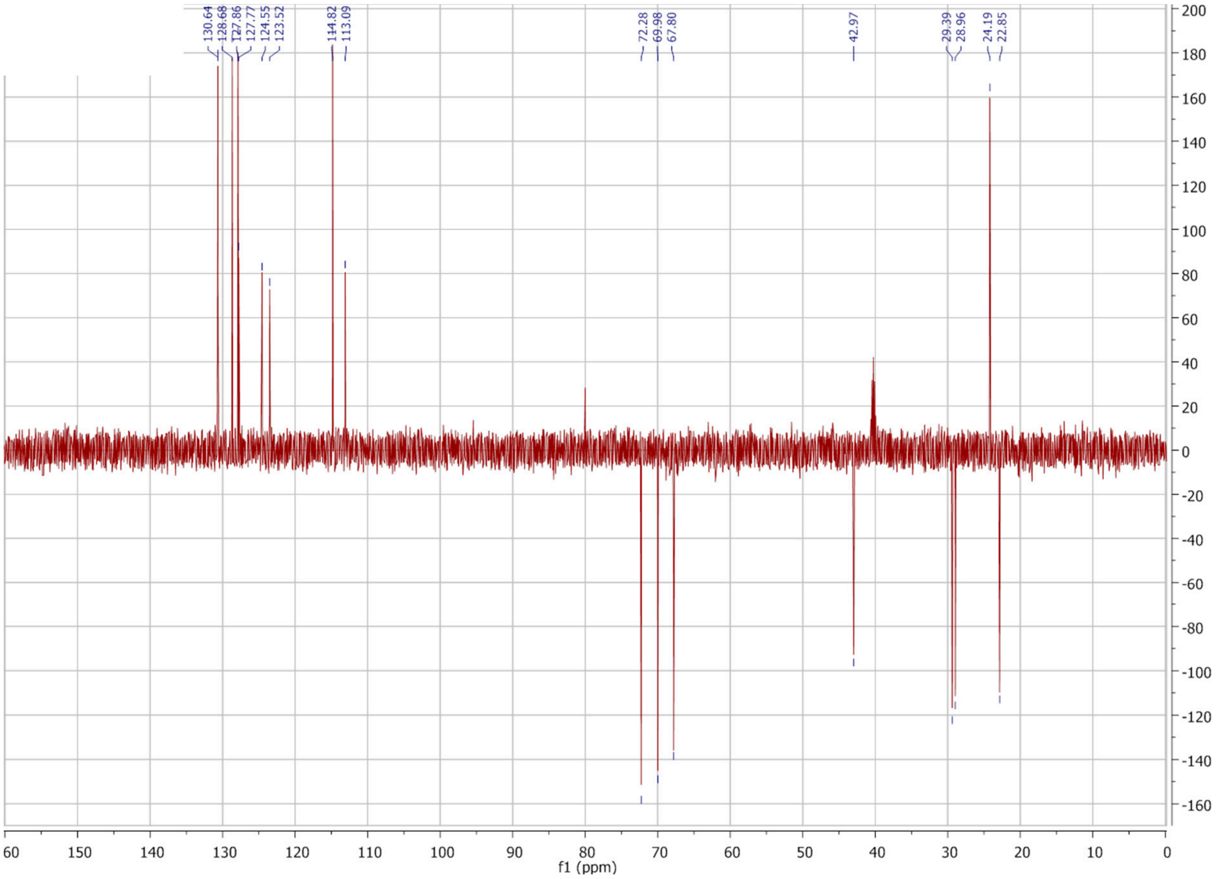

N-(2-benzyl-1,3-dioxo-2,3-dihydro-1H-isoindol-5-yl)-2-{4-[2-(benzyloxy)ethoxy]phenyl}acetamide (**12b**; ZHAWOC5467)

<u>NMR</u>

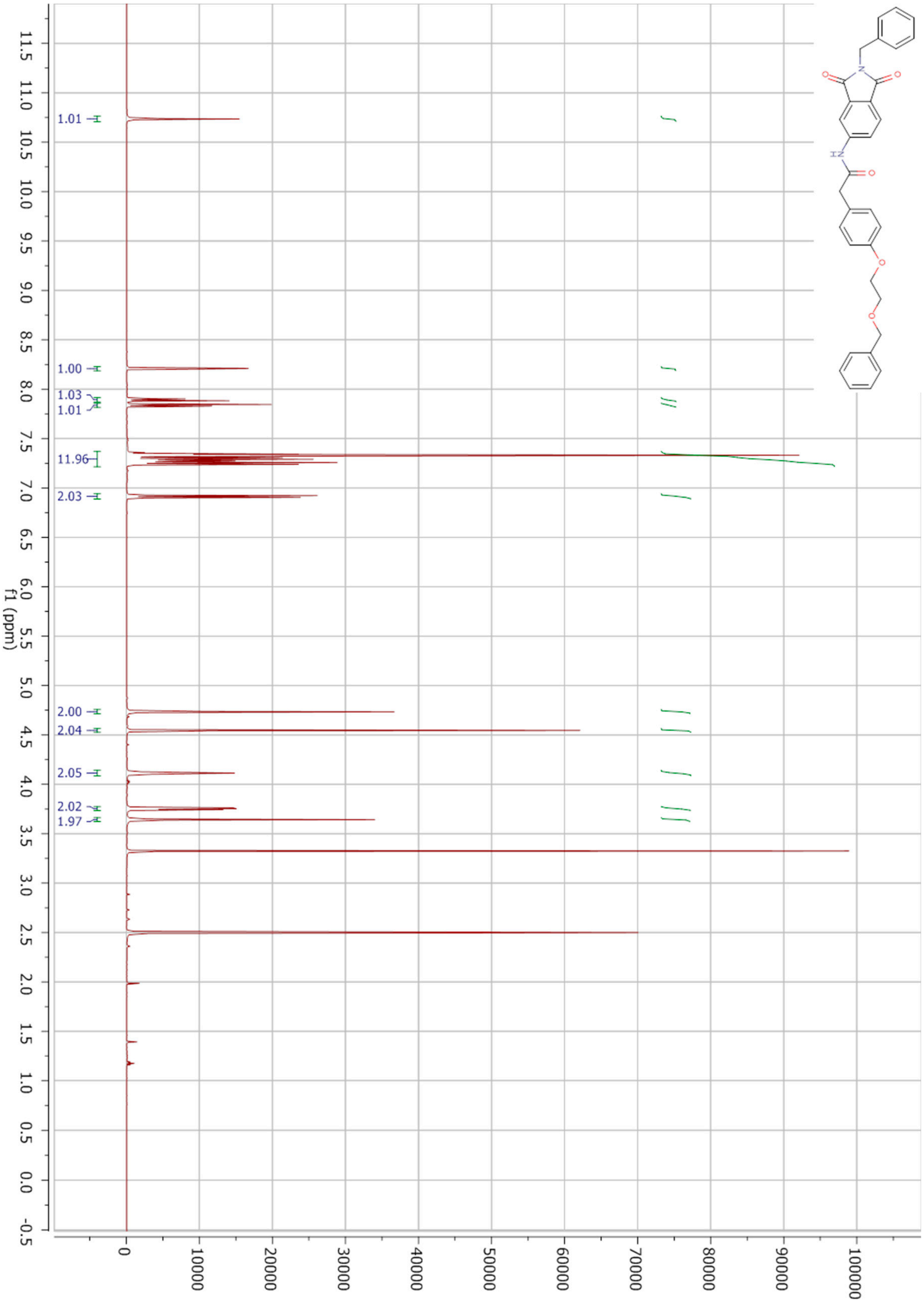

N-(2-benzyl-1,3-dioxo-2,3-dihydro-1H-isoindol-5-yl)-2-{4-[2-(benzyloxy)ethoxy]phenyl}acetamide (**12b**; ZHAWOC5467)

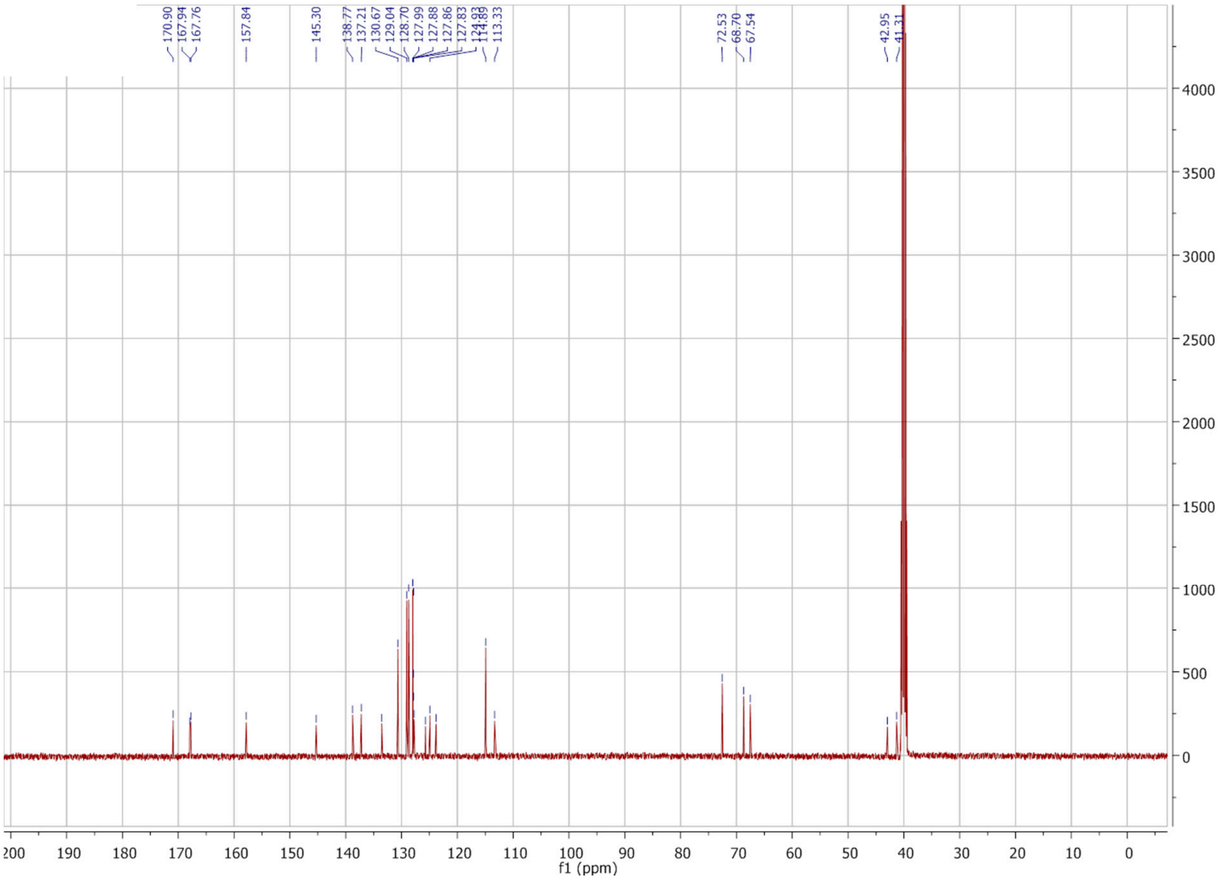

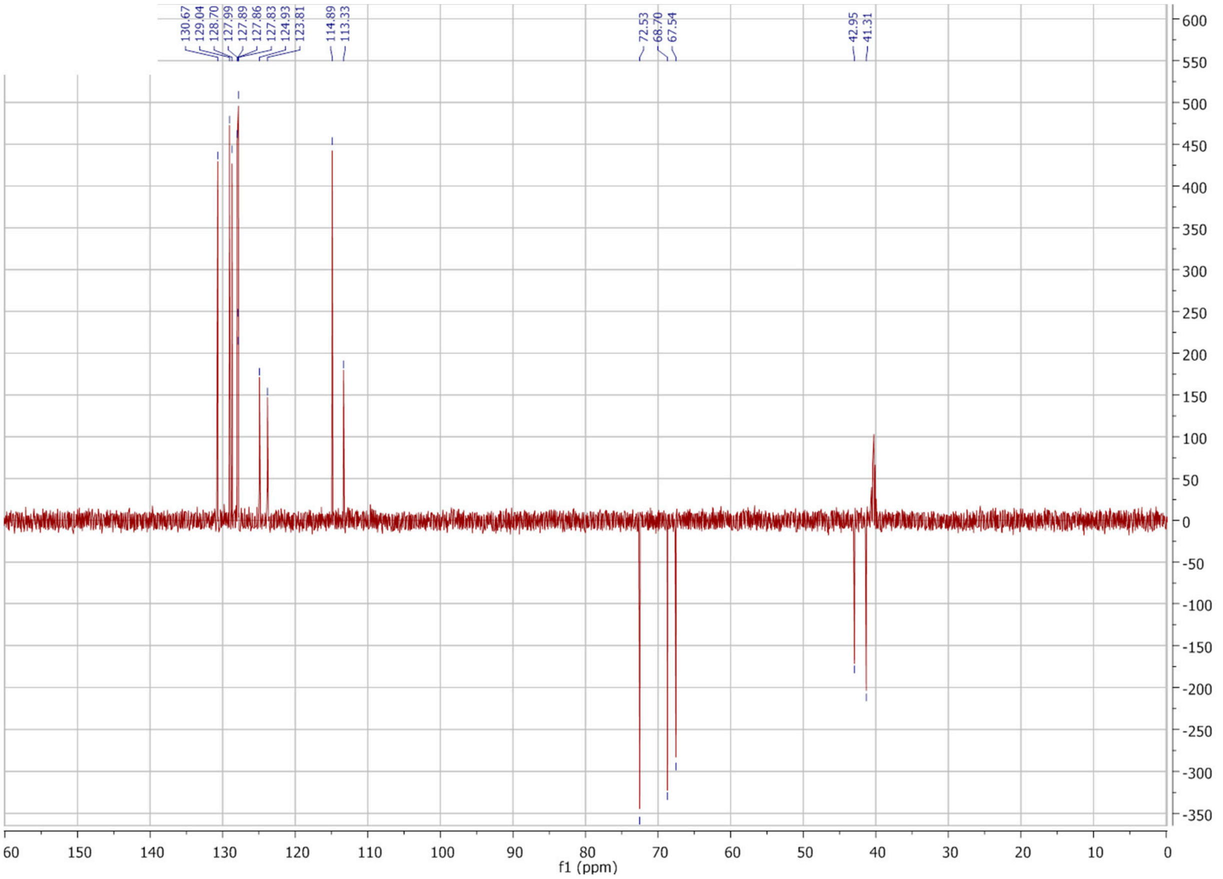

N-(2-benzyl-1,3-dioxo-2,3-dihydro-1H-isoindol-5-yl)-2-{4-[3-(benzyloxy)propoxy]phenyl}acetamide (**12c;** ZHAWOC4511)

<u>NMR</u>

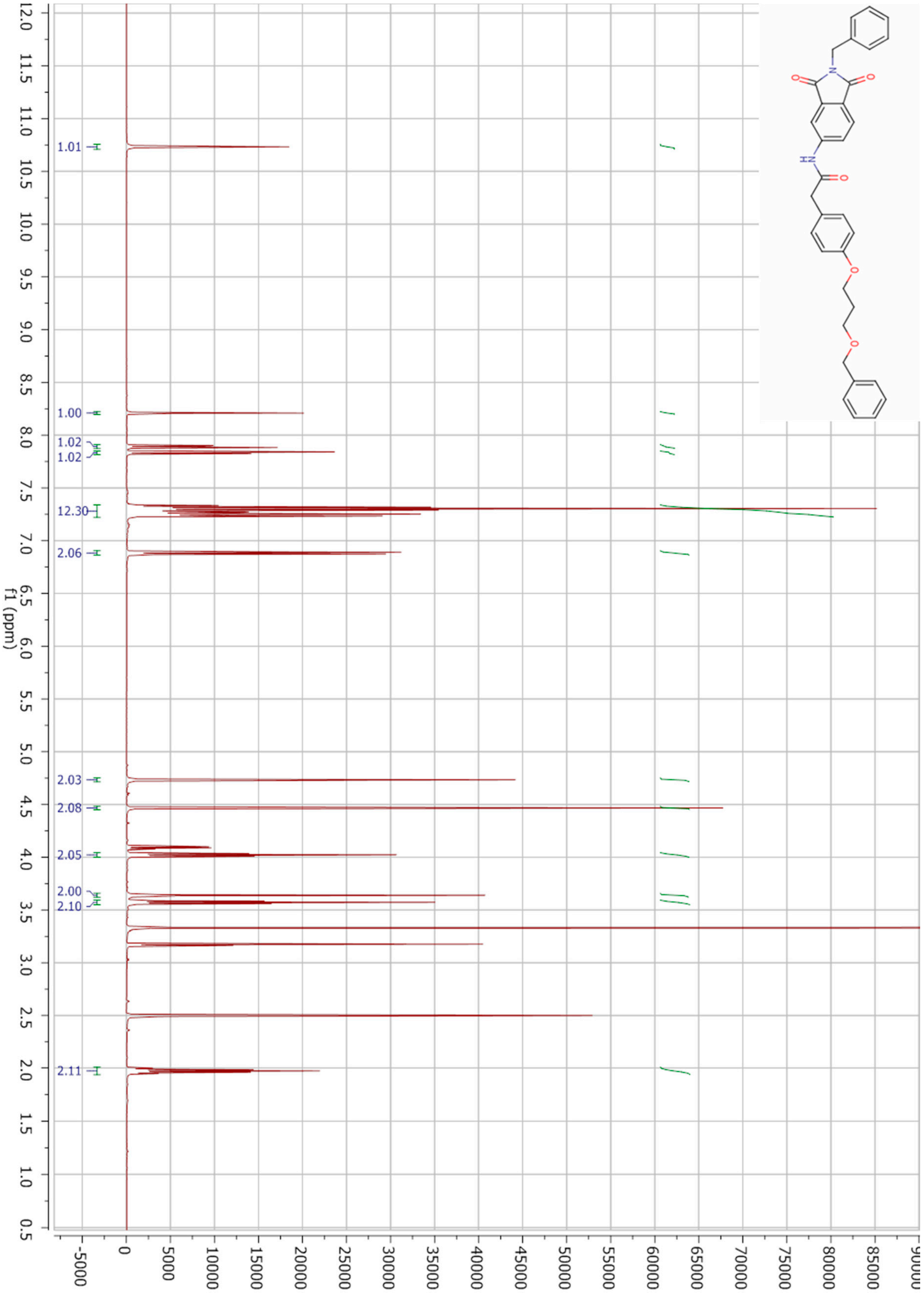

N-(2-benzyl-1,3-dioxo-2,3-dihydro-1H-isoindol-5-yl)-2-{4-[3-(benzyloxy)propoxy]phenyl}acetamide (**12c;** ZHAWOC4511)

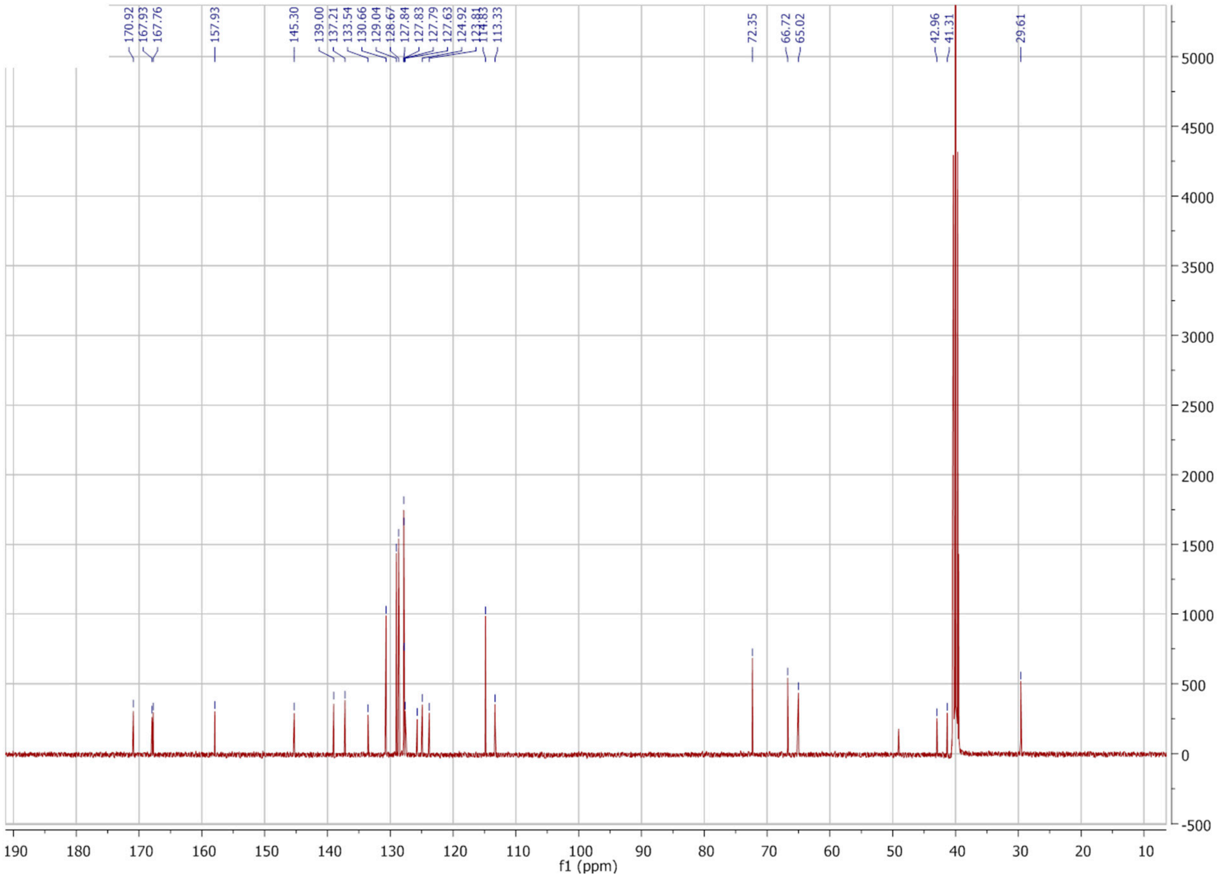

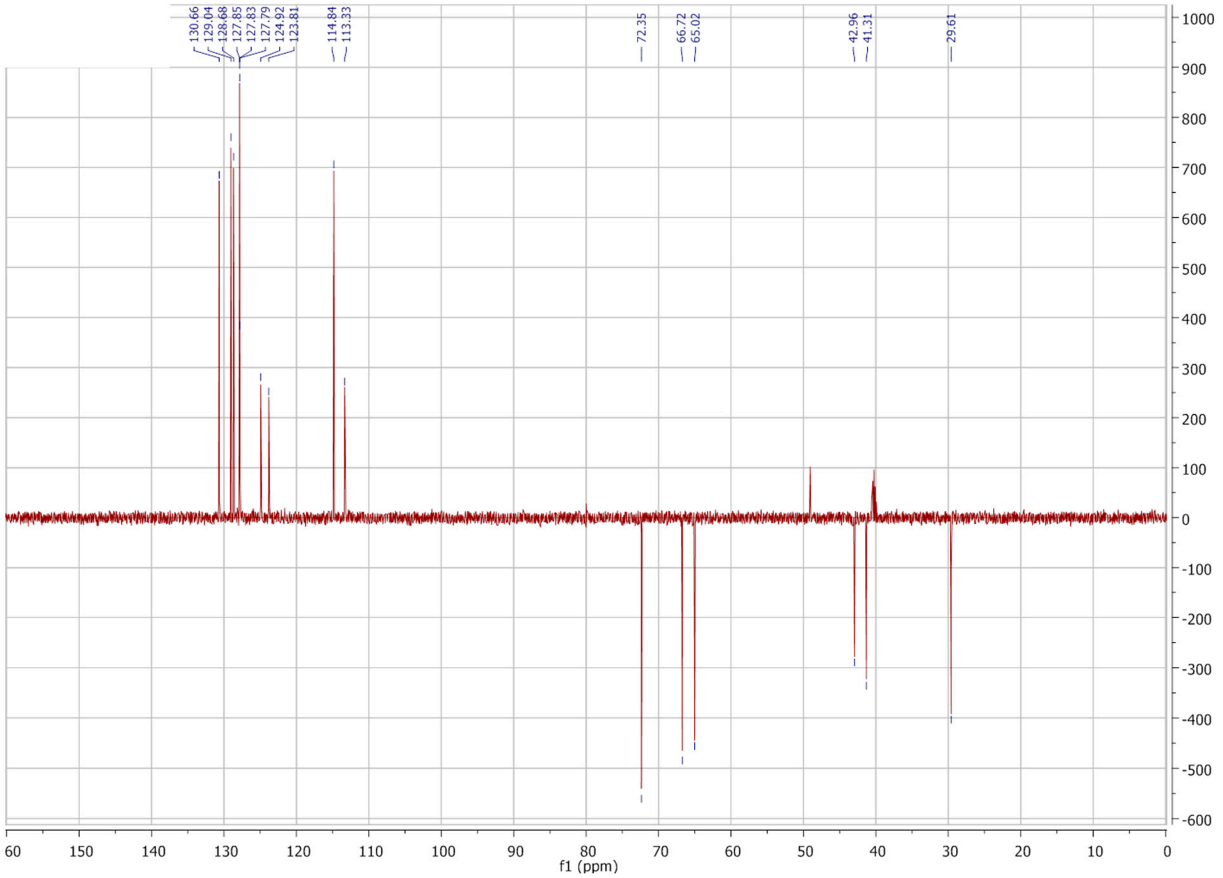

N-(2-benzyl-1,3-dioxo-2,3-dihydro-1H-isoindol-5-yl)-2-{4-[4-(benzyloxy)butoxy]phenyl}acetamide (**12d;** ZHAWOC4752)

<u>NMR</u>

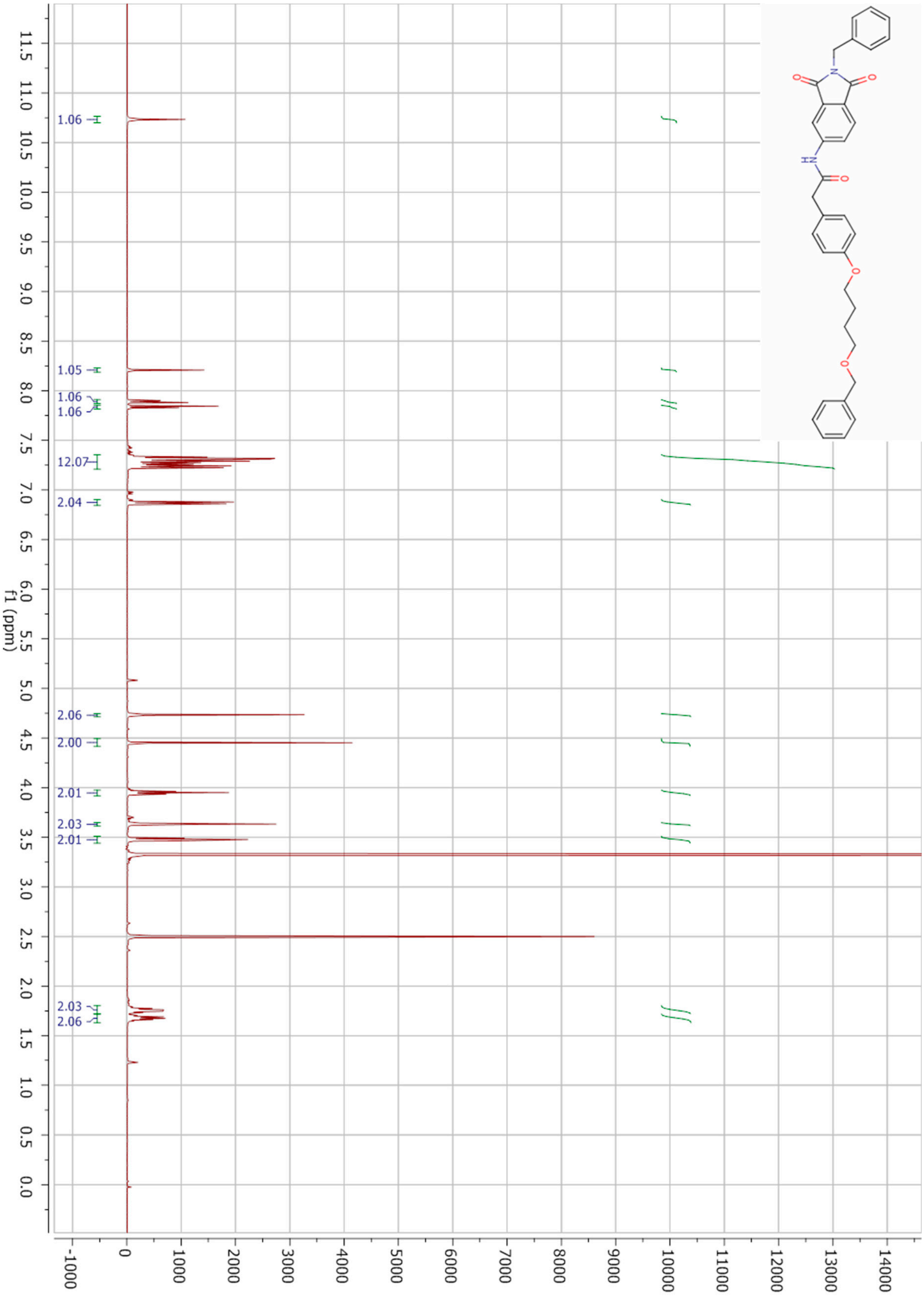

N-(2-benzyl-1,3-dioxo-2,3-dihydro-1H-isoindol-5-yl)-2-{4-[4-(benzyloxy)butoxy]phenyl}acetamide (**12d;** ZHAWOC4752)

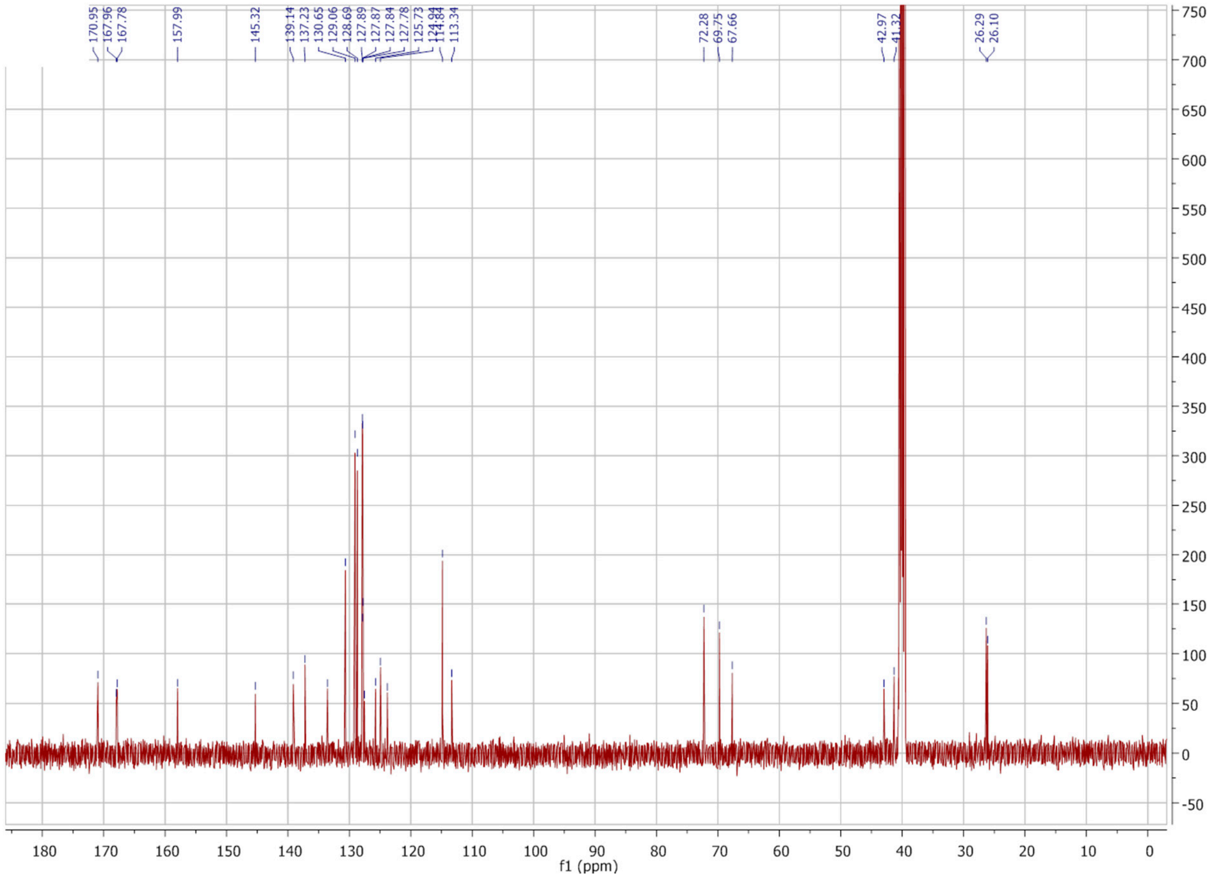

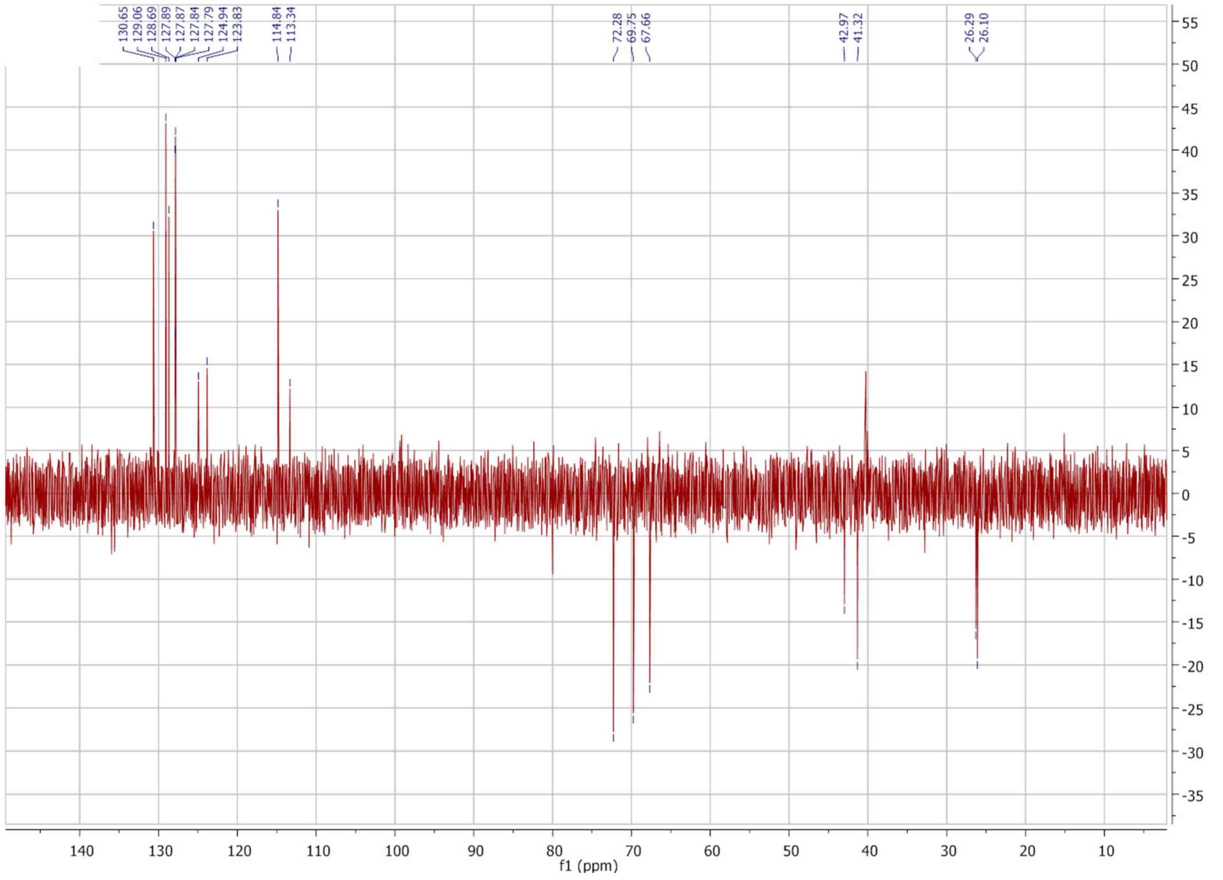

N-(2-benzyl-1,3-dioxo-2,3-dihydro-1H-isoindol-5-yl)-2-(4-{[5-(benzyloxy)pentyl]oxy}phenyl)acetamide (**12e;** ZHAWOC5979)

<u>NMR</u>

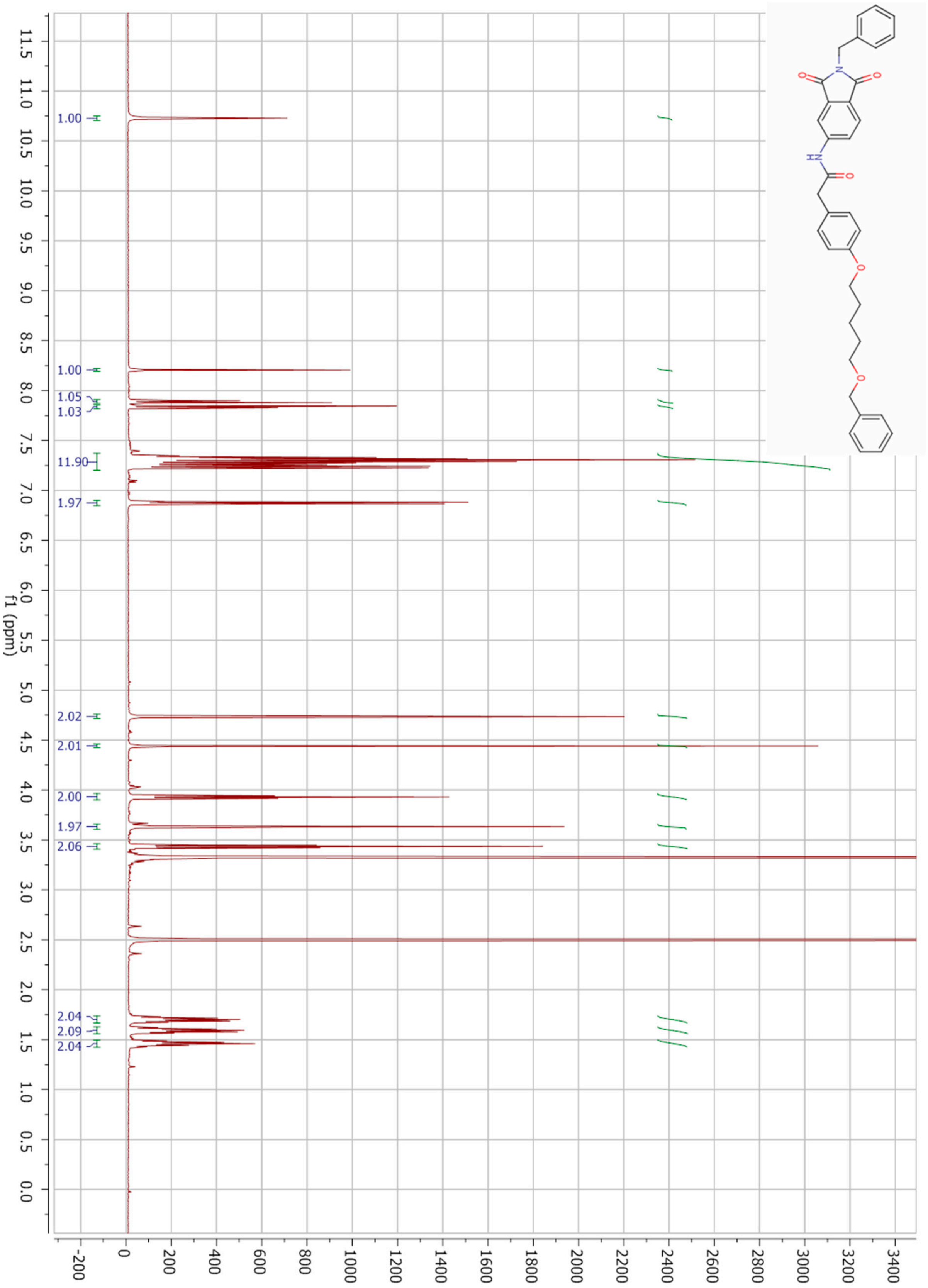

N-(2-benzyl-1,3-dioxo-2,3-dihydro-1H-isoindol-5-yl)-2-(4-{[5-(benzyloxy)pentyl]oxy}phenyl)acetamide (**12e;** ZHAWOC5979)

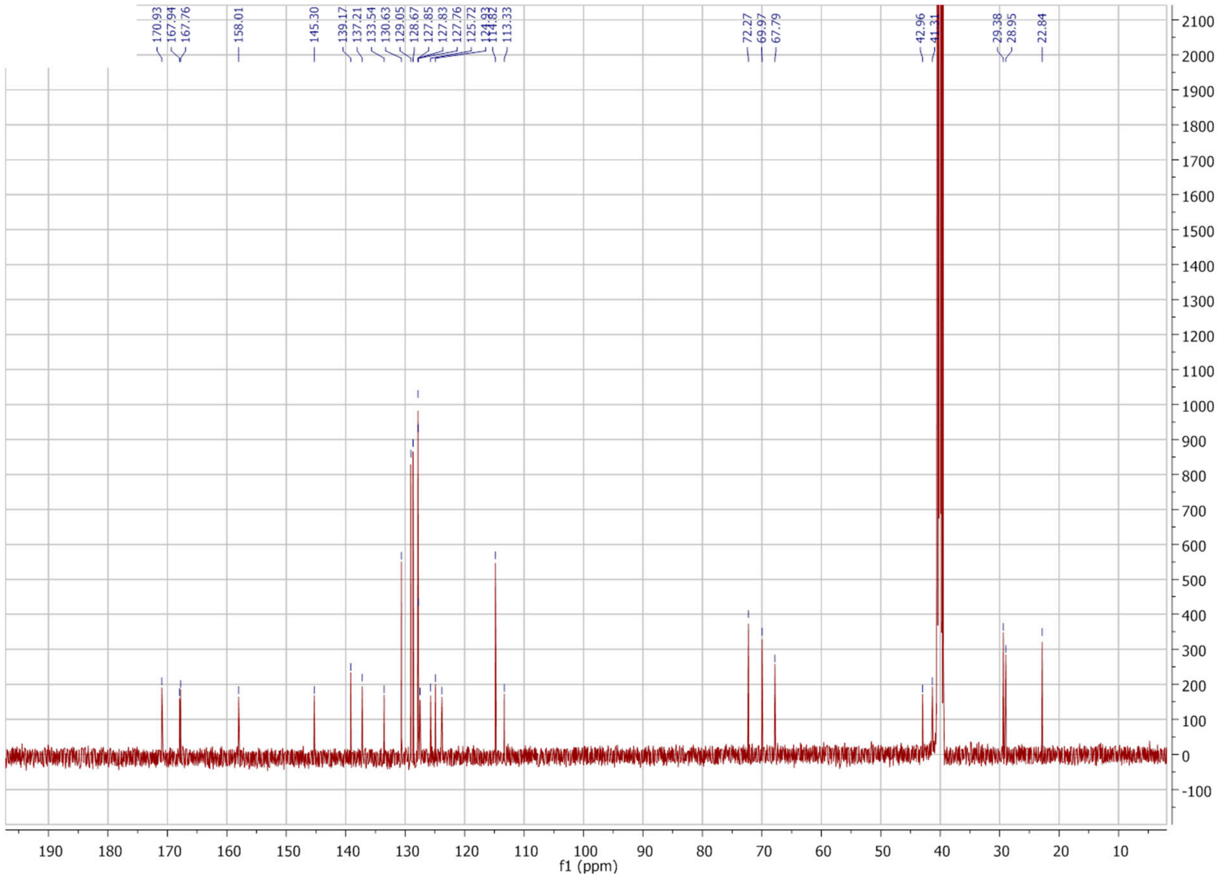

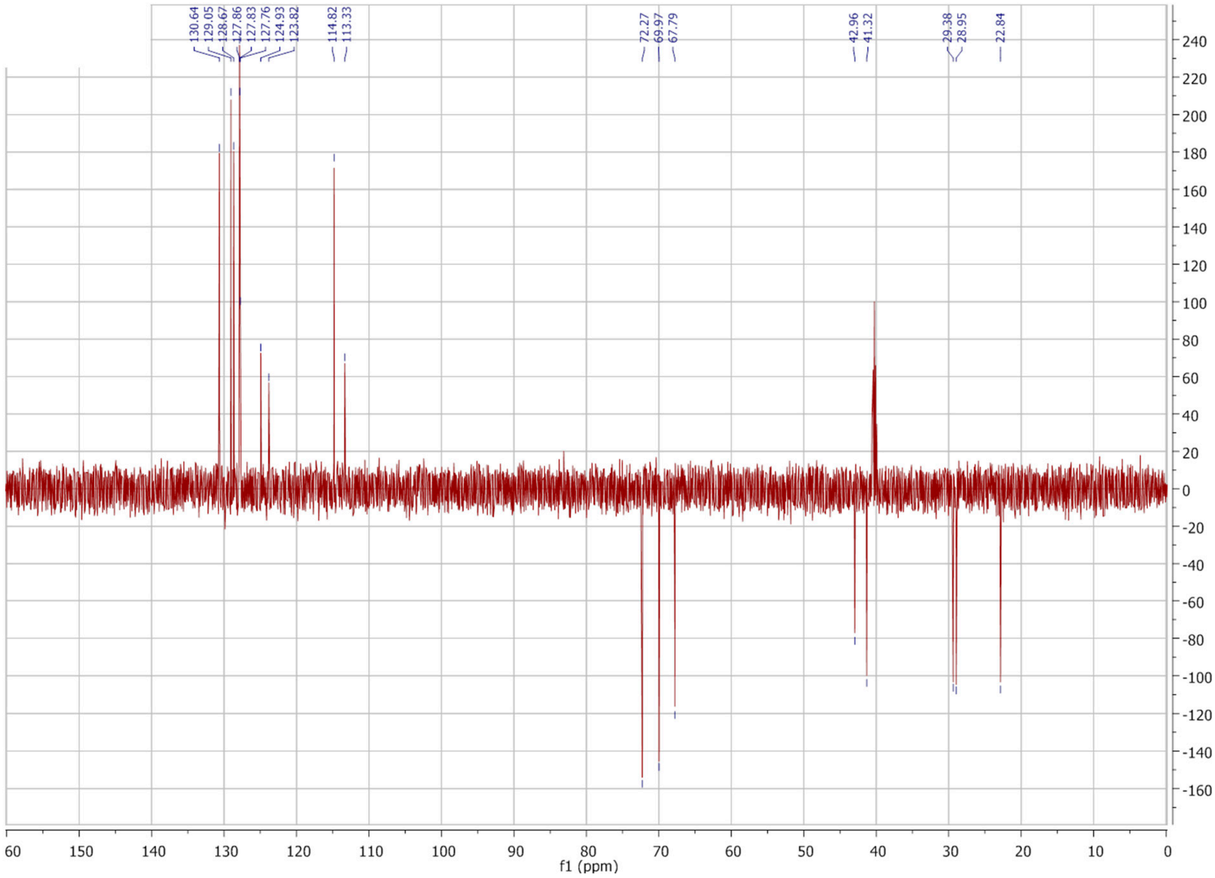

N-(2-benzyl-1,3-dioxo-2,3-dihydro-1H-isoindol-5-yl)-2-(4-{[6-(benzyloxy)hexyl]oxy}phenyl)acetamide (**12f;** ZHAWOC5980)

<u>NMR</u>

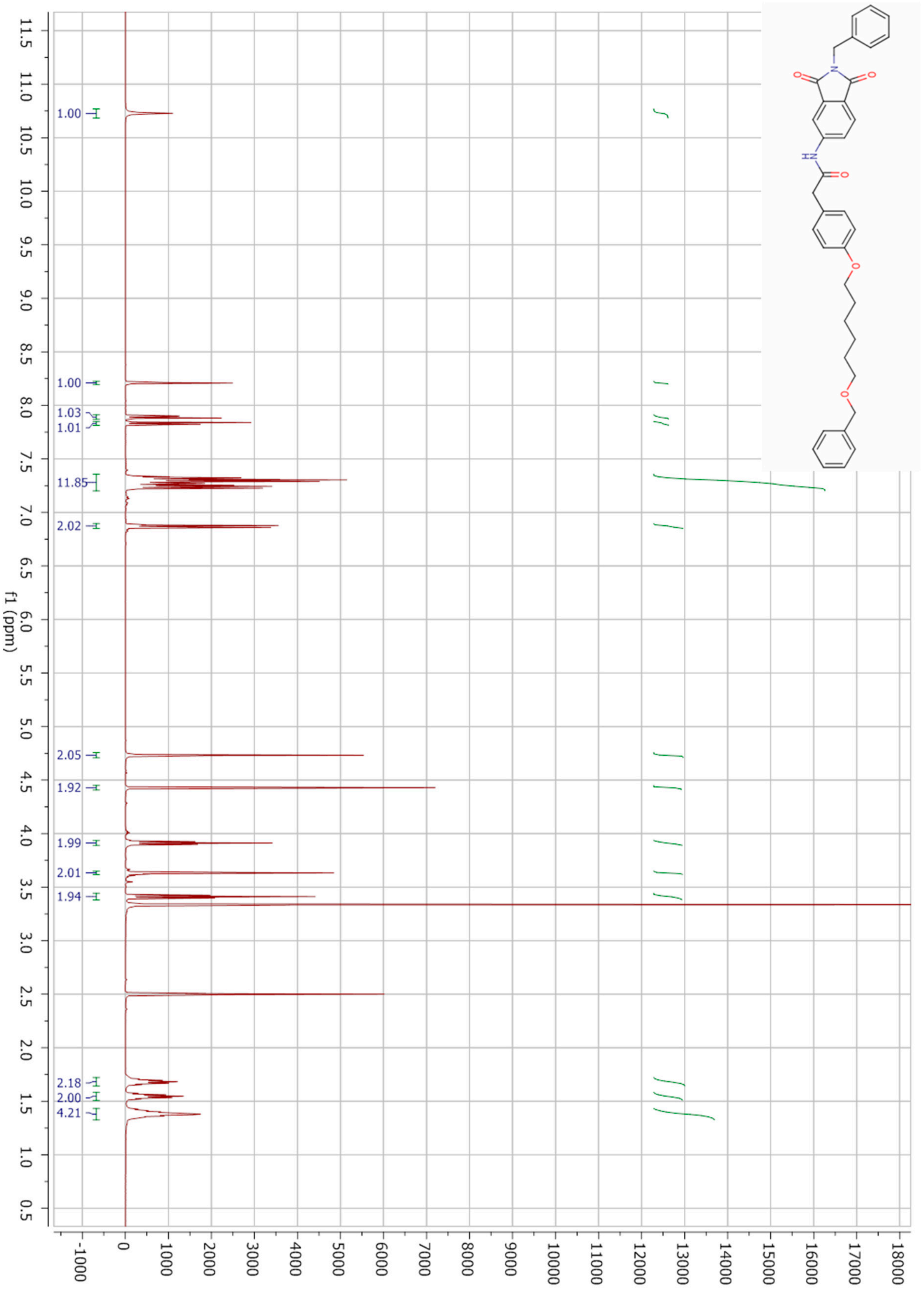

N-(2-benzyl-1,3-dioxo-2,3-dihydro-1H-isoindol-5-yl)-2-(4-{[6-(benzyloxy)hexyl]oxy}phenyl)acetamide (**12f;** ZHAWOC5980)

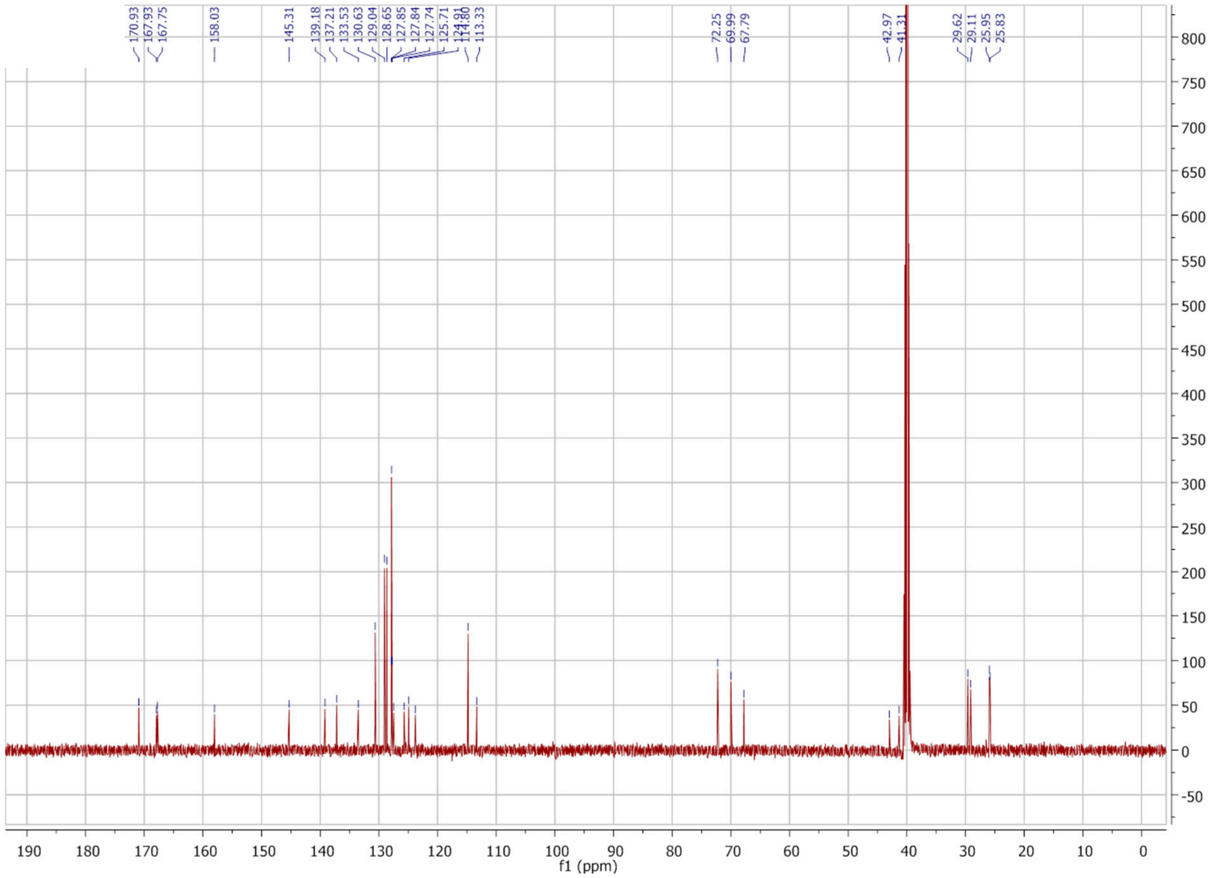

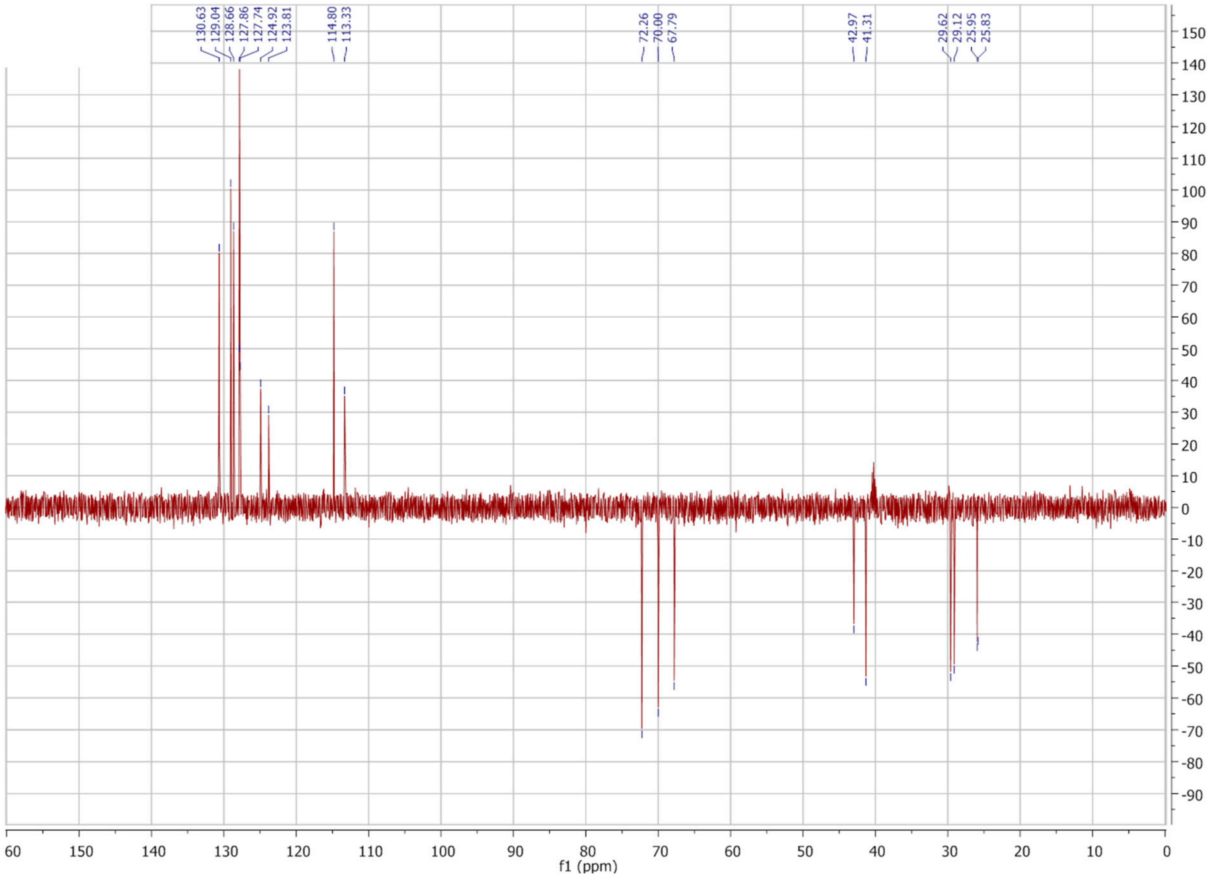

N-(2-benzyl-1,3-dioxo-2,3-dihydro-1H-isoindol-5-yl)-2-(4-{[7-(benzyloxy)heptyl]oxy}phenyl)acetamide (**12g**; ZHAWOC7099)

<u>NMR</u>

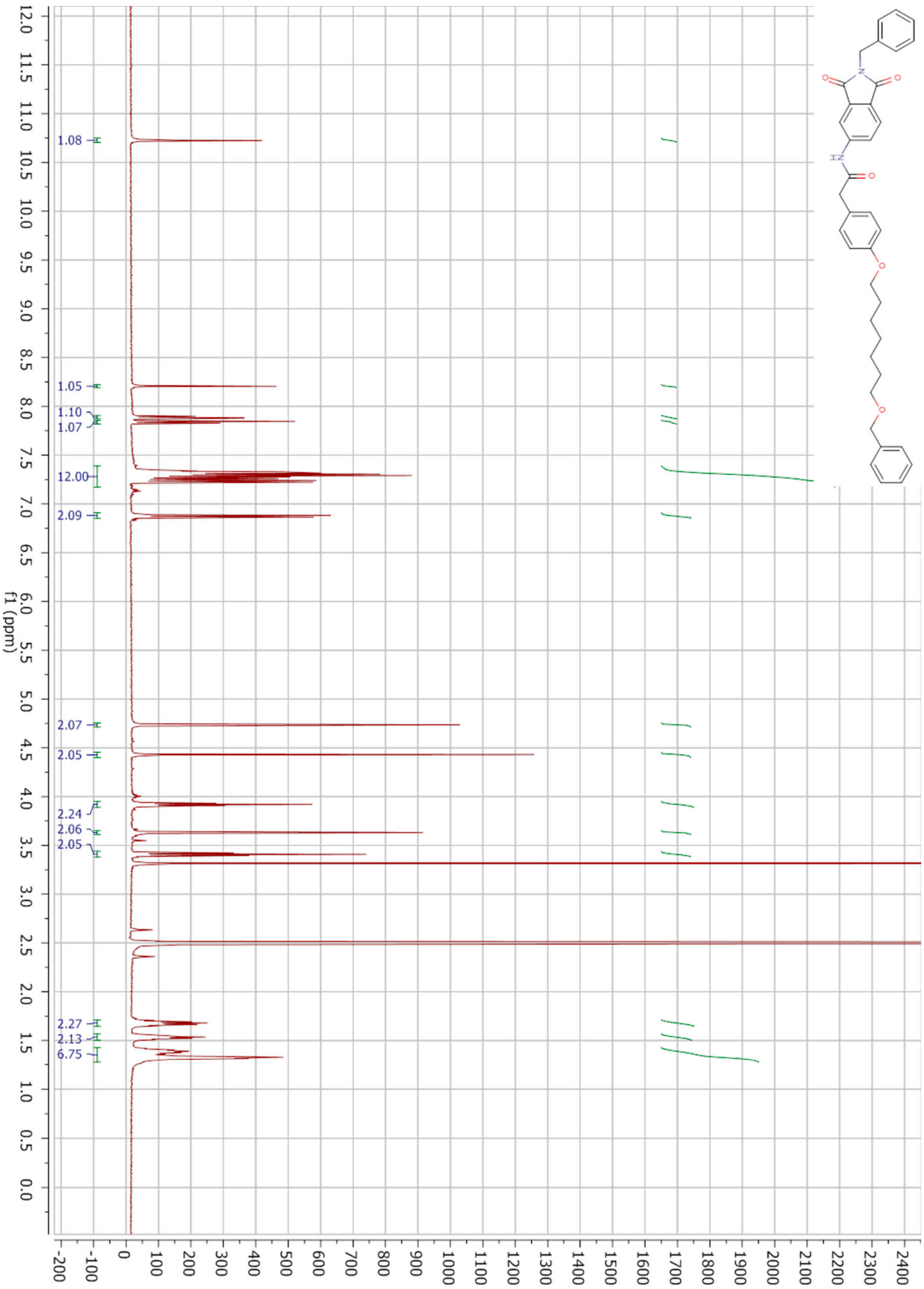

N-(2-benzyl-1,3-dioxo-2,3-dihydro-1H-isoindol-5-yl)-2-(4-{[7-(benzyloxy)heptyl]oxy}phenyl)acetamide (**12g**; ZHAWOC7099)

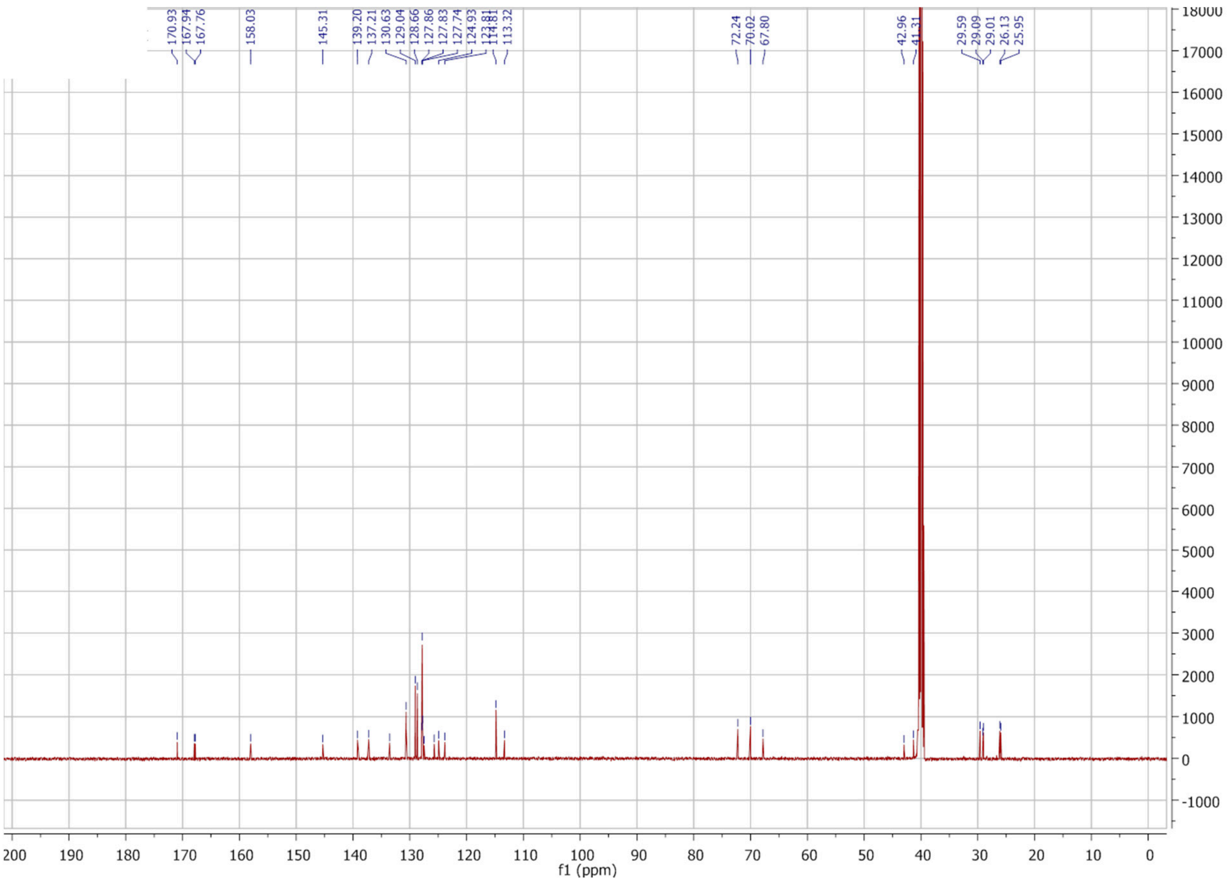

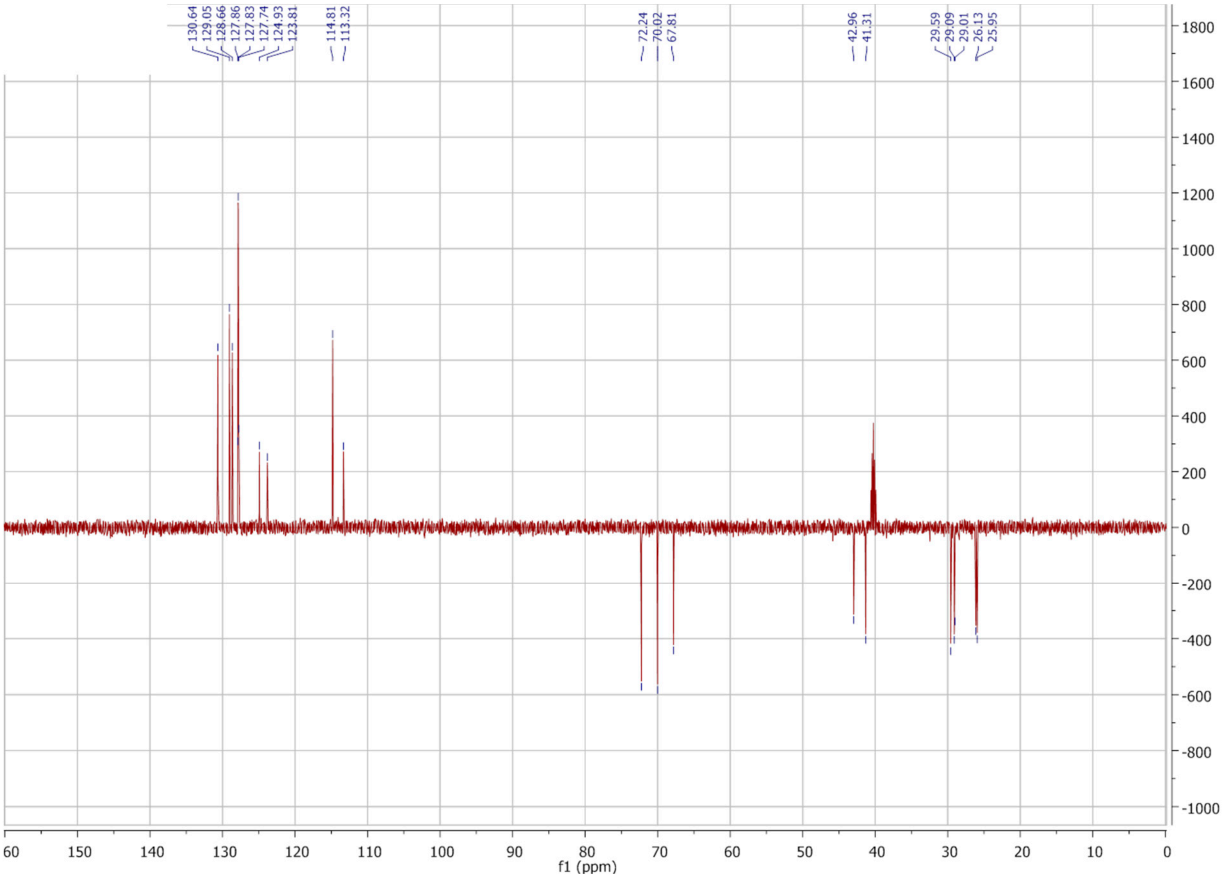

N-(2-benzyl-1,3-dioxo-2,3-dihydro-1H-isoindol-5-yl)-2-(4-{[8-(benzyloxy)octyl]oxy}phenyl)acetamide (**12h**; ZHAWOC7095)

NMR

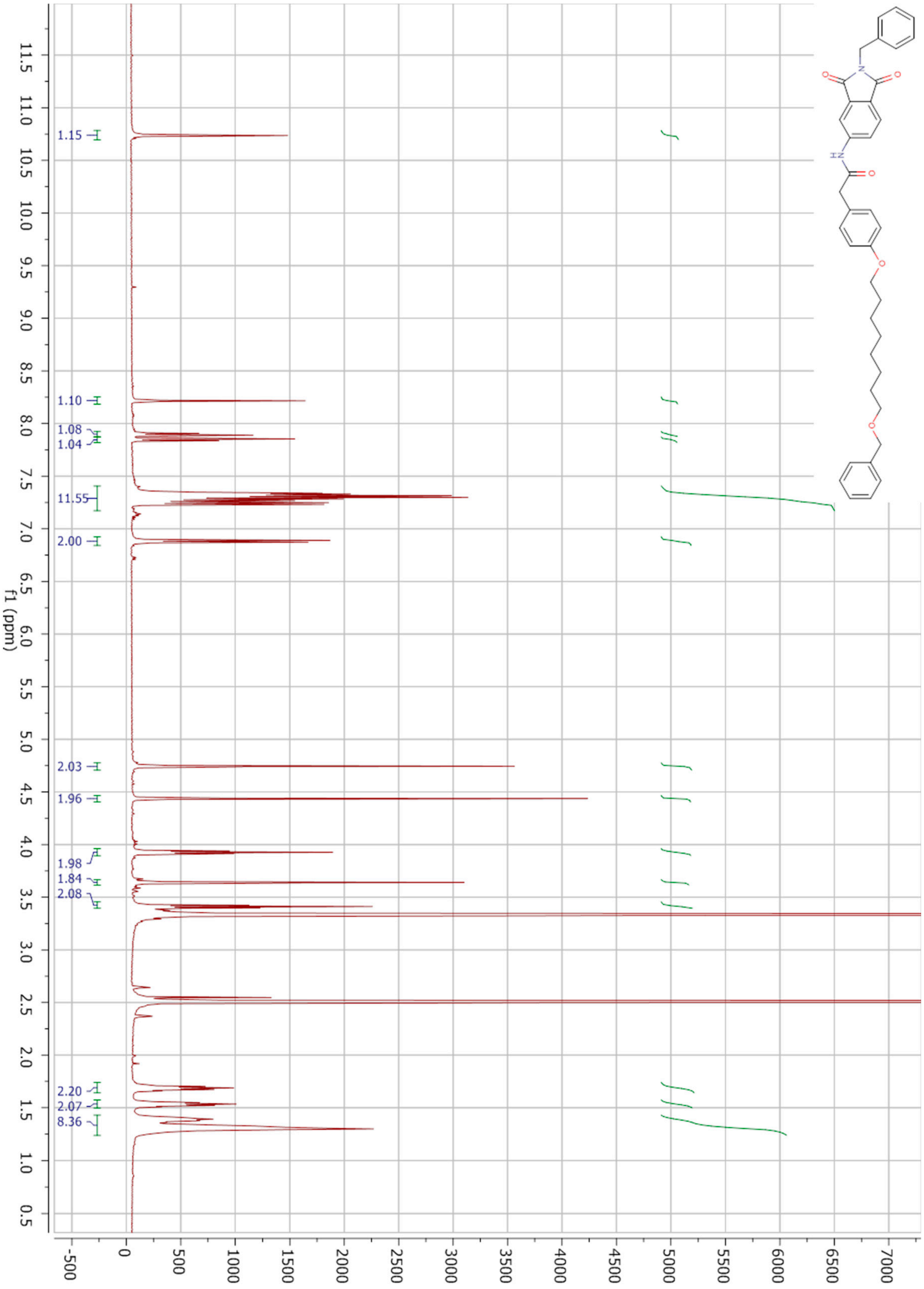

N-(2-benzyl-1,3-dioxo-2,3-dihydro-1H-isoindol-5-yl)-2-(4-{[8-(benzyloxy)octyl]oxy}phenyl)acetamide (**12h**; ZHAWOC7095)

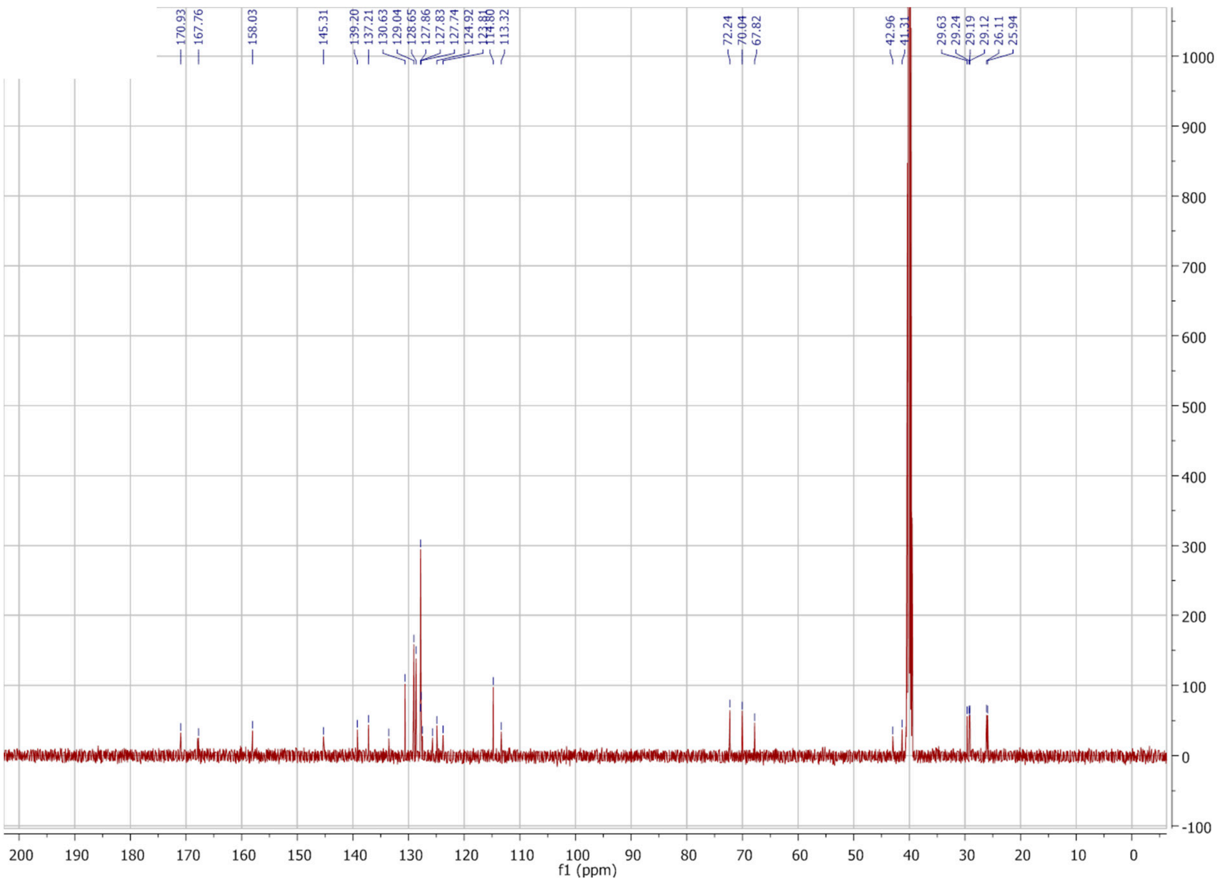

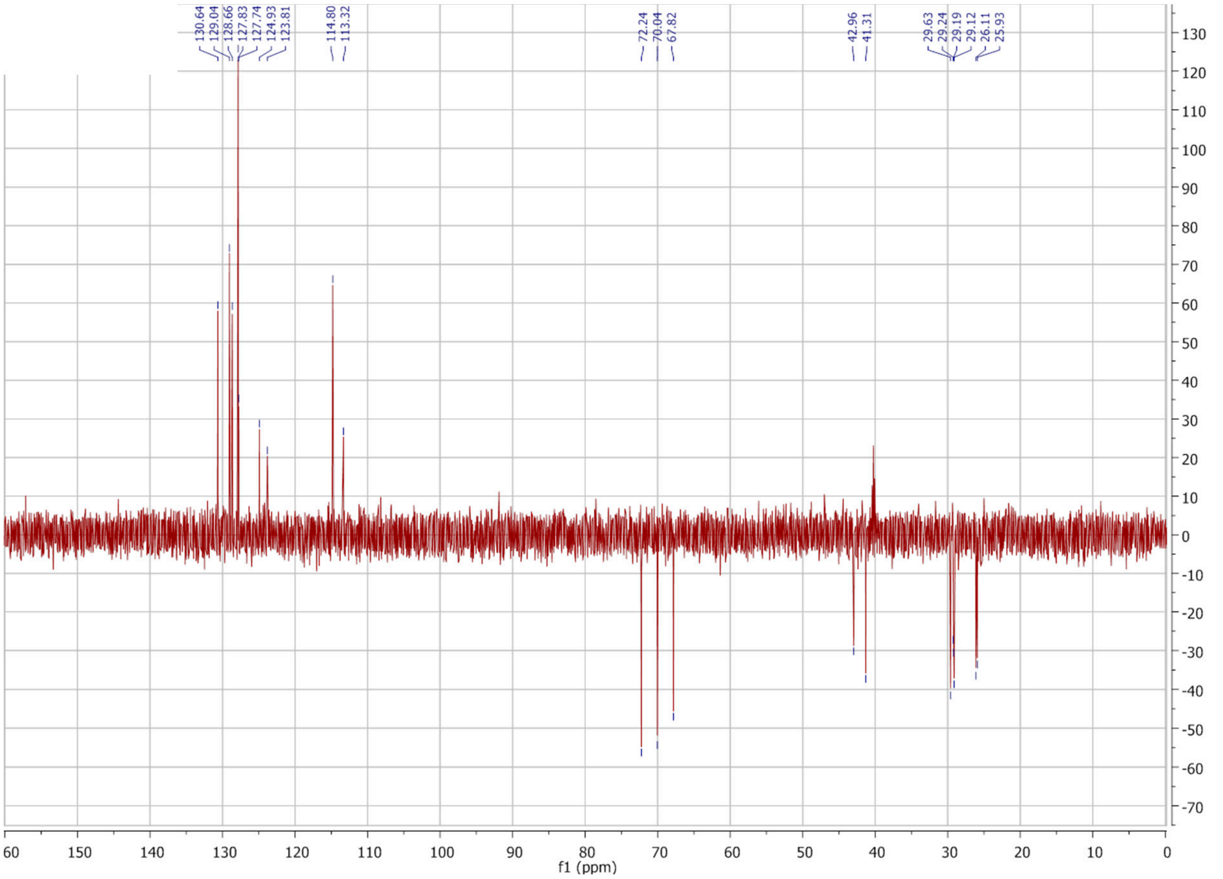

N-(2-benzyl-1,3-dioxo-2,3-dihydro-1H-isoindol-5-yl)-2-(4-{[9-(benzyloxy)nonyl]oxy}phenyl)acetamide (**12i**; ZHAWOC6931)

<u>NMR</u>

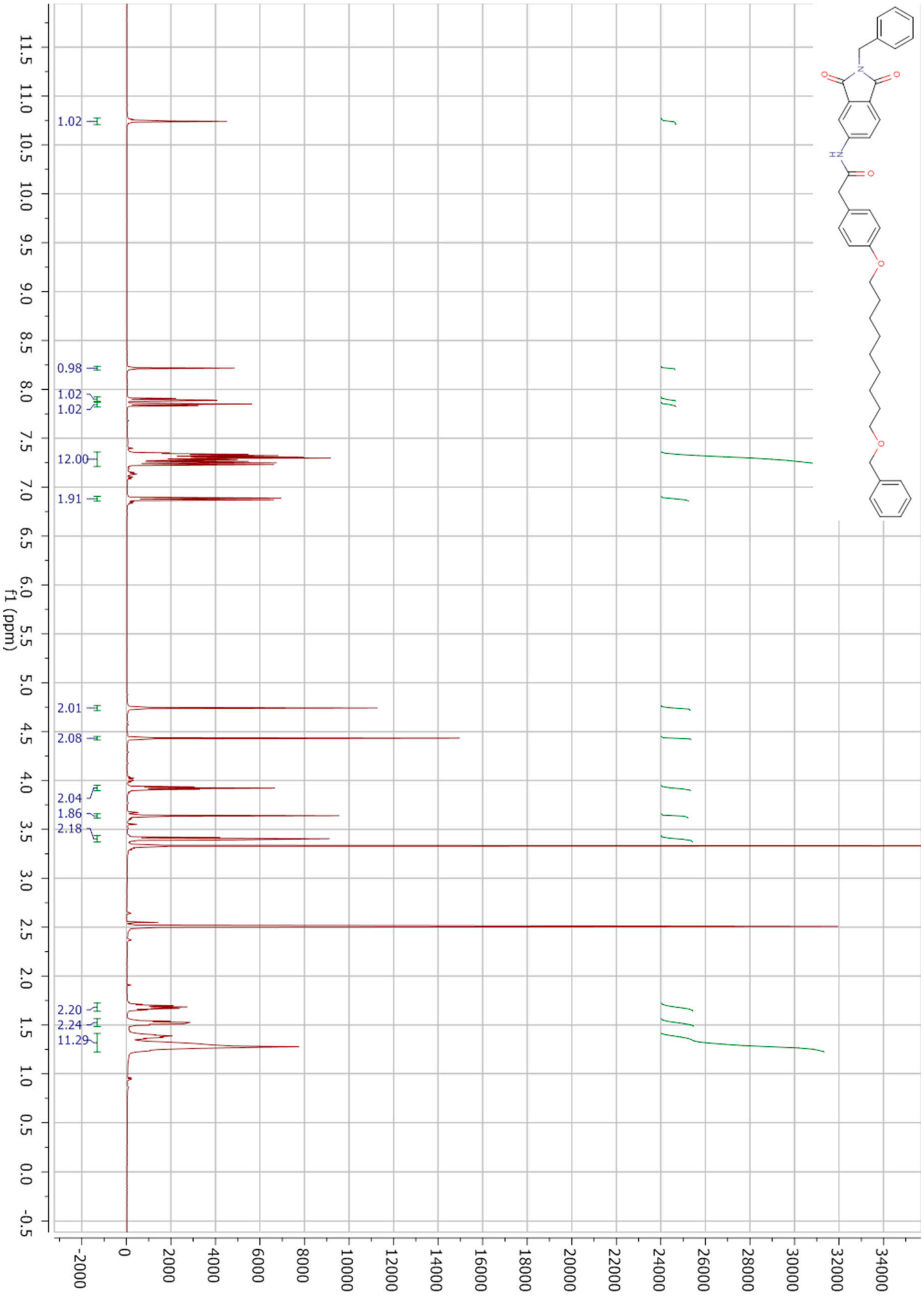

N-(2-benzyl-1,3-dioxo-2,3-dihydro-1H-isoindol-5-yl)-2-(4-{[9-(benzyloxy)nonyl]oxy}phenyl)acetamide (**12i**; ZHAWOC6931)

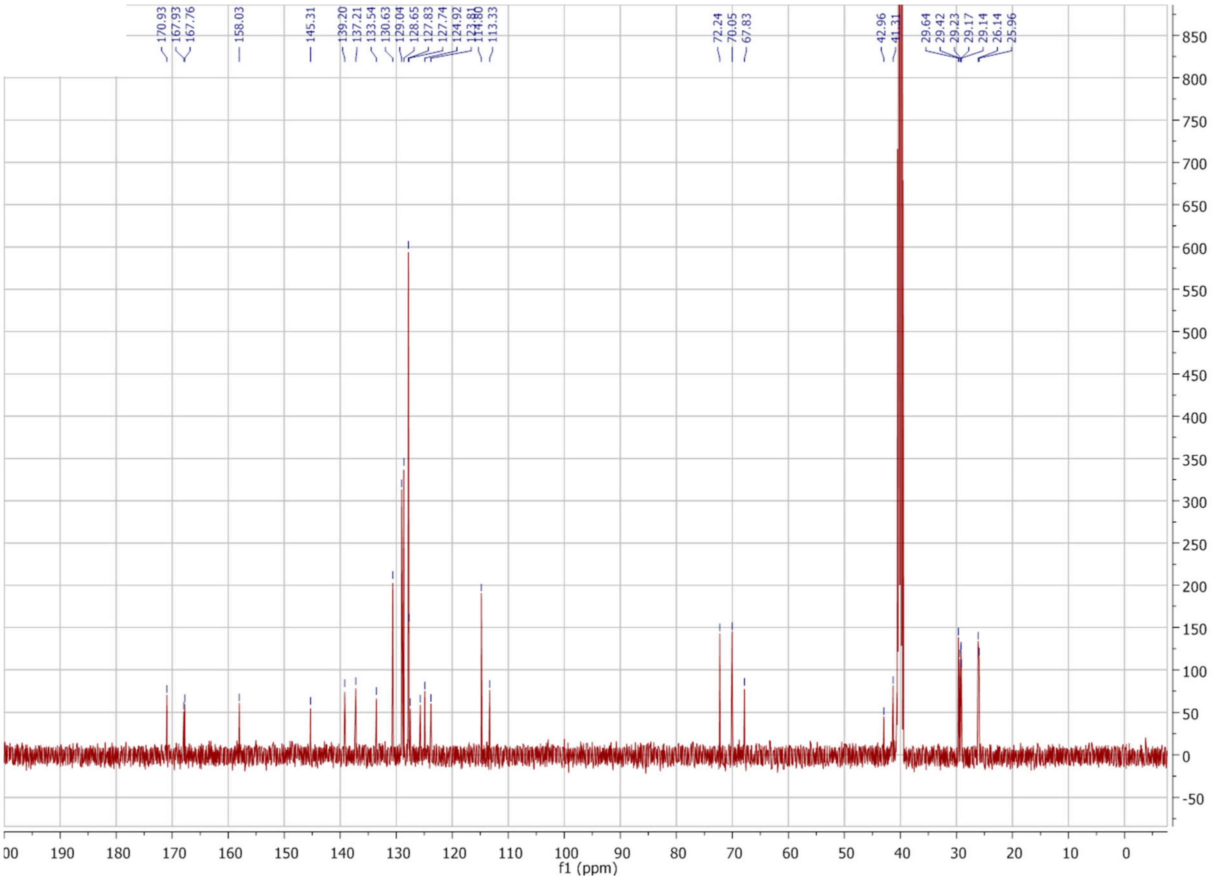

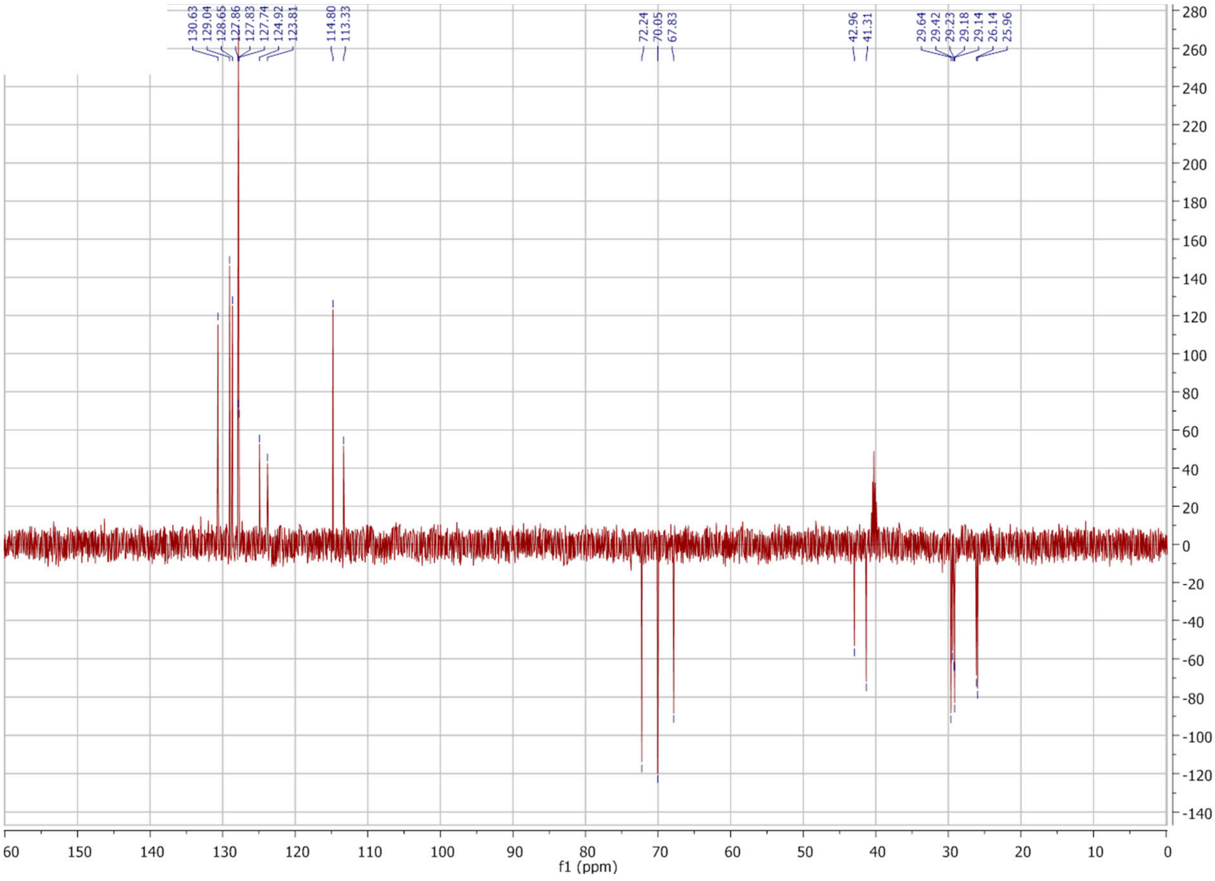

N-(2-benzyl-1,3-dioxo-2,3-dihydro-1H-isoindol-5-yl)-2-(4-{[10-(benzyloxy)decyl]oxy}phenyl)acetamide (**12j**; ZHAWOC6932)

<u>NMR</u>

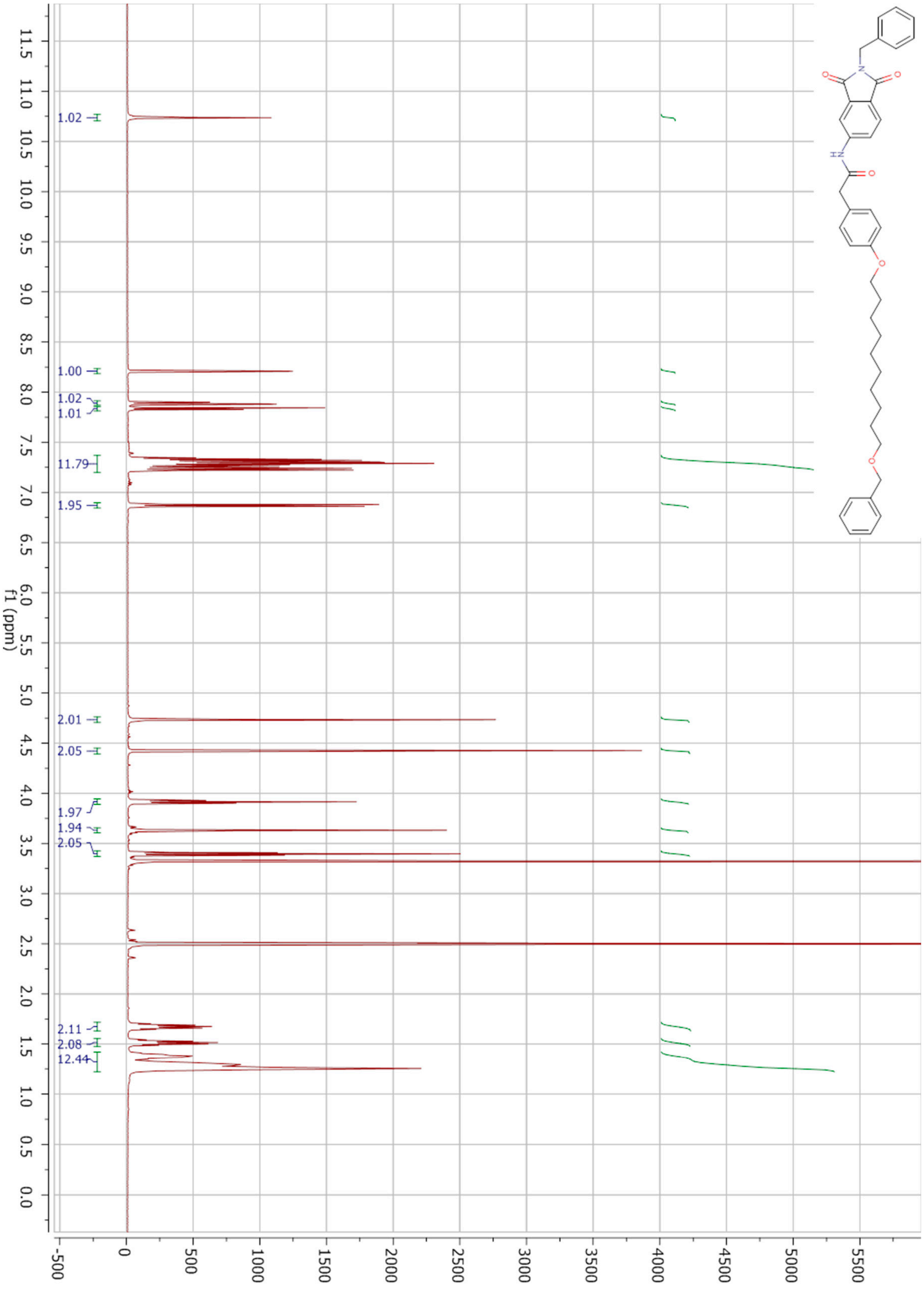

N-(2-benzyl-1,3-dioxo-2,3-dihydro-1H-isoindol-5-yl)-2-(4-{[10-(benzyloxy)decyl]oxy}phenyl)acetamide (**12j**; ZHAWOC6932)

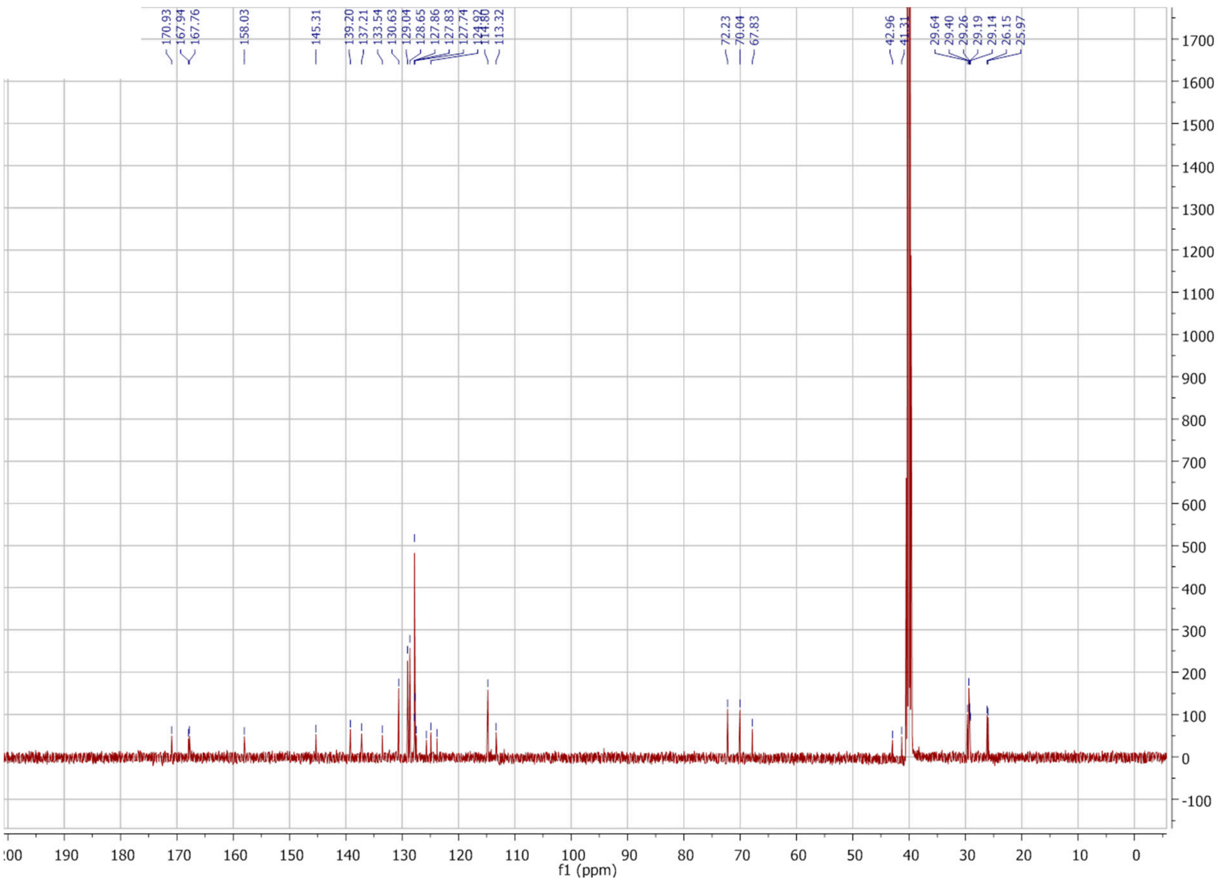

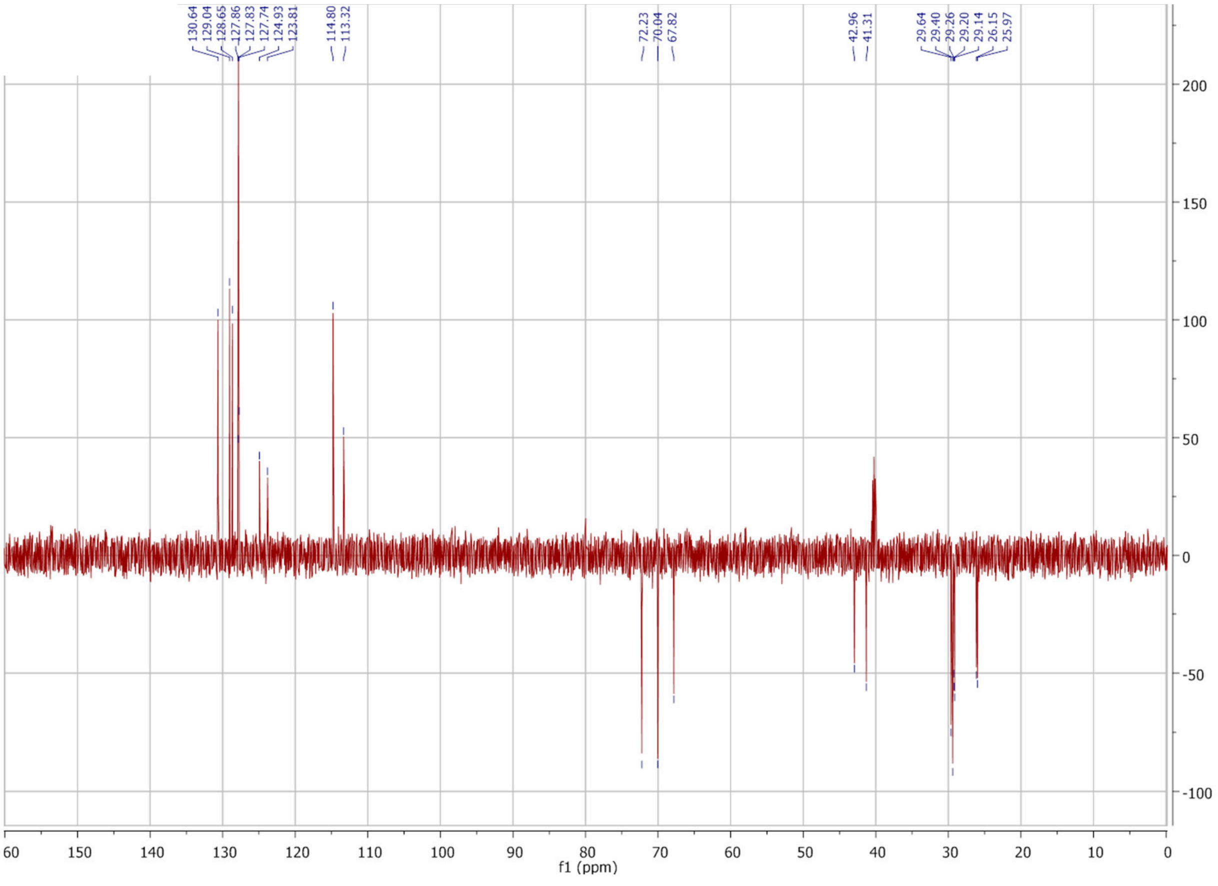

2-{4-[3-(benzyloxy)propoxy]phenyl}-N-{2-[(4-fluorophenyl)methyl]-1,3-dioxo-2,3-dihydro-1H-isoindol-5-yl}acetamide (**12k**; ZHAWOC6641)

<u>NMR</u>

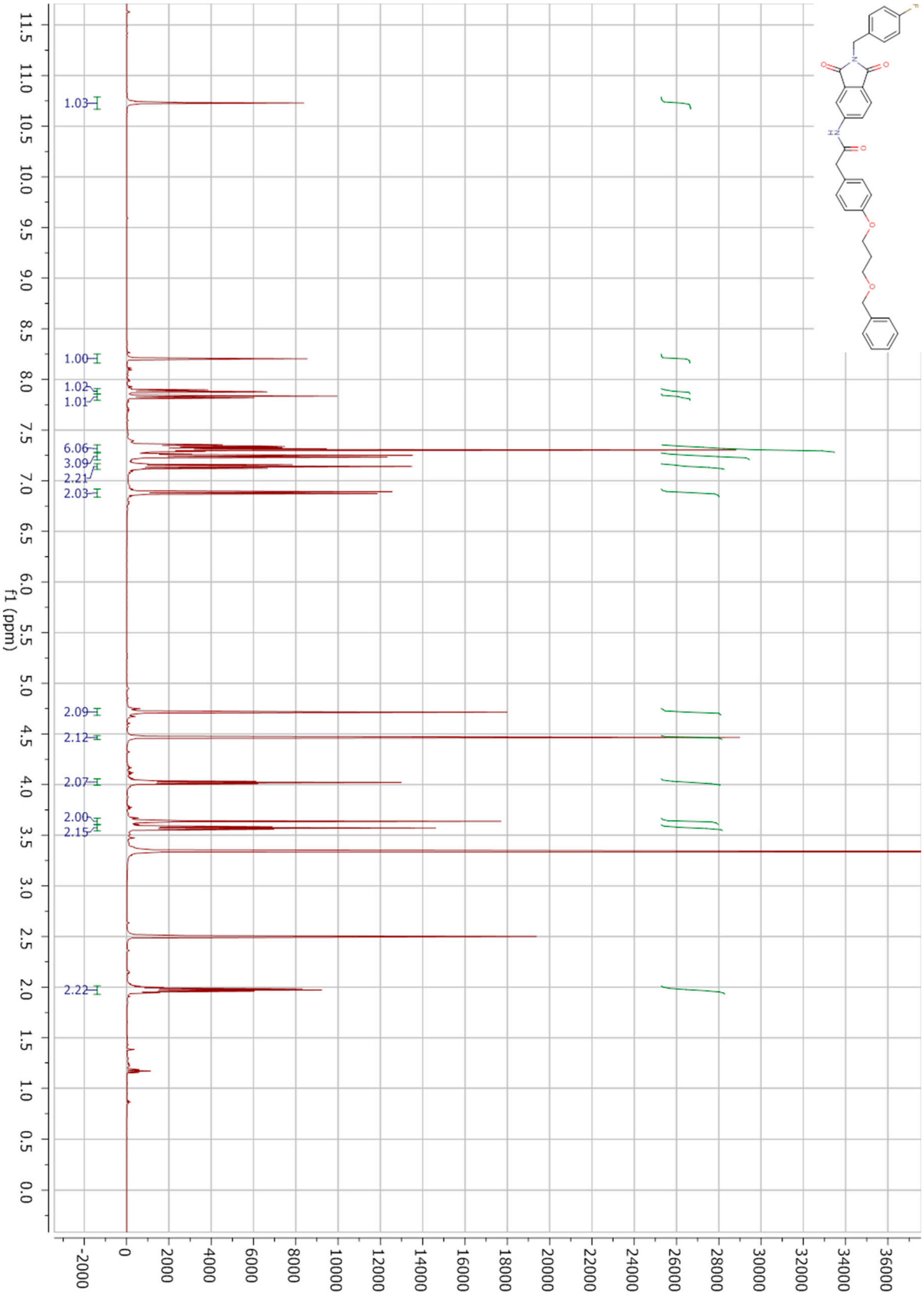

2-{4-[3-(benzyloxy)propoxy]phenyl}-N-{2-[(4-fluorophenyl)methyl]-1,3-dioxo-2,3-dihydro-1H-isoindol-5-yl}acetamide (**12k**; ZHAWOC6641)

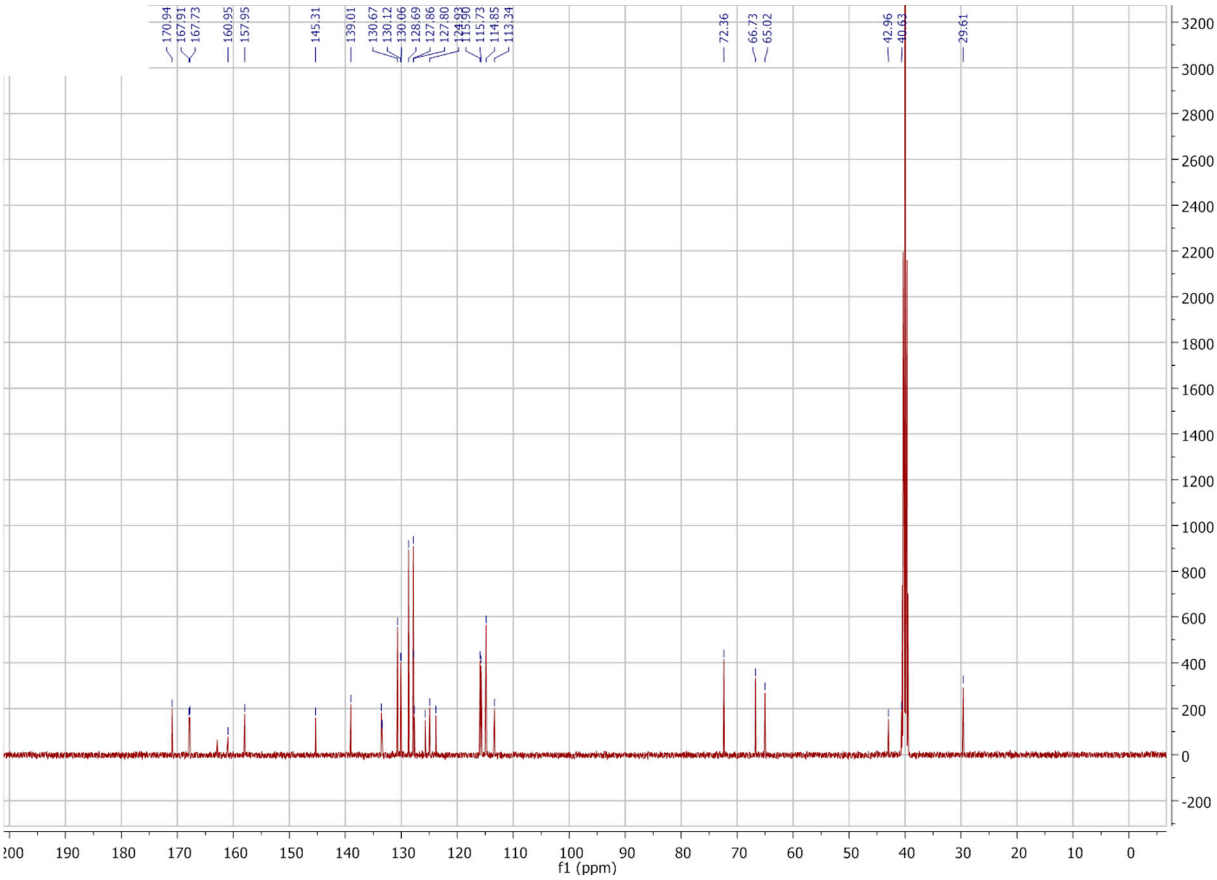

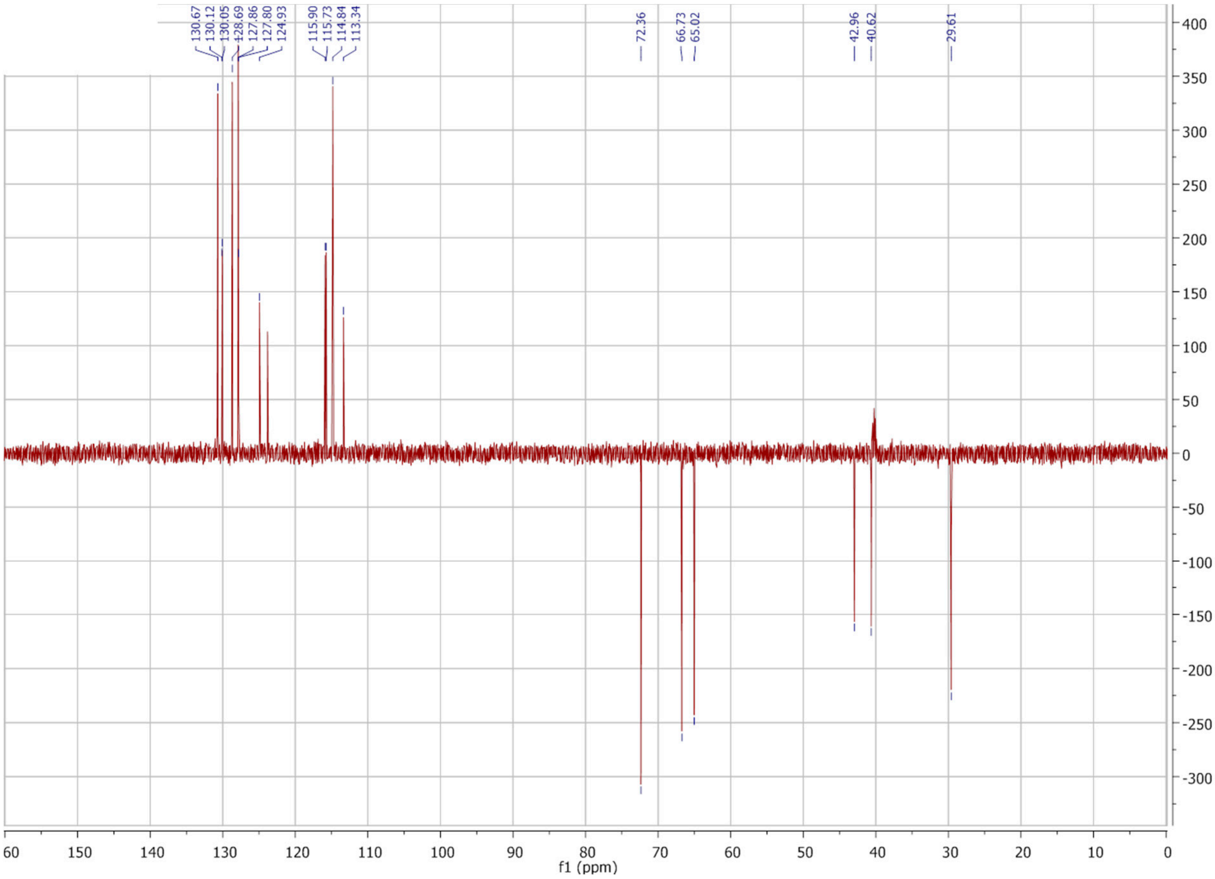

2-{4-[4-(benzyloxy)butoxy]phenyl}-N-{2-[(4-fluorophenyl)methyl]-1,3-dioxo-2,3-dihydro-1H-isoindol-5-yl}acetamide (**12l**; ZHAWOC7102)

<u>NMR</u>

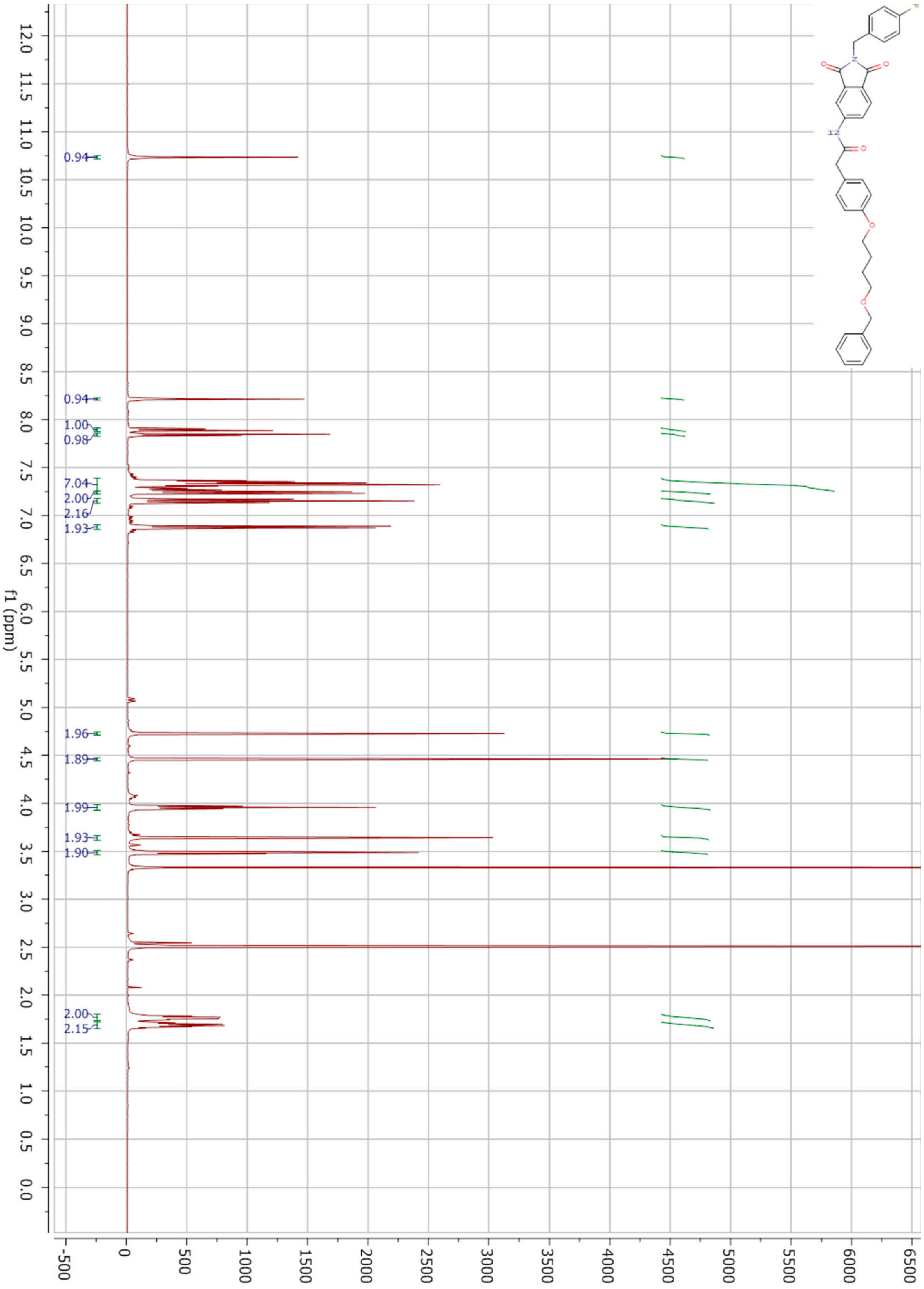

2-{4-[4-(benzyloxy)butoxy]phenyl}-N-{2-[(4-fluorophenyl)methyl]-1,3-dioxo-2,3-dihydro-1H-isoindol-5-yl}acetamide (**12l**; ZHAWOC7102)

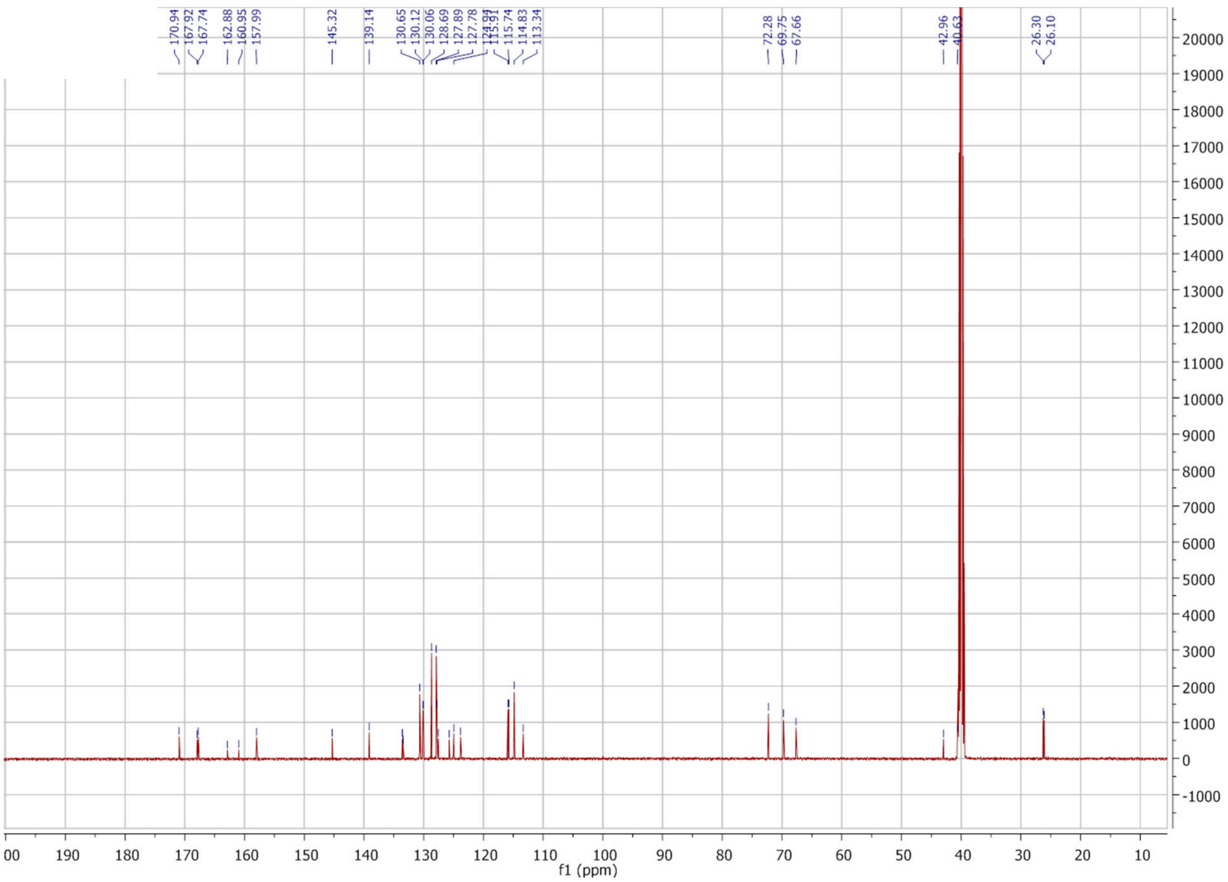

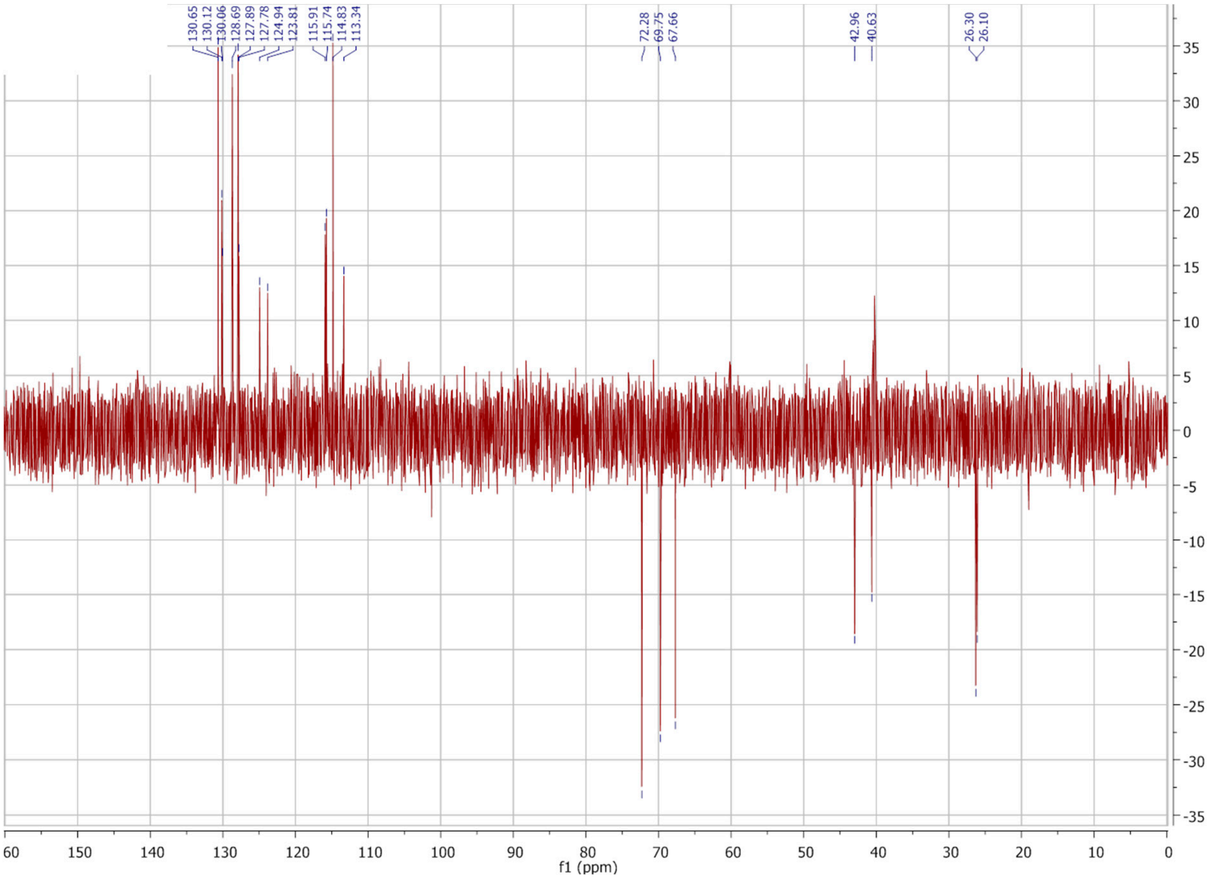

2-(4-{[5-(benzyloxy)pentyl]oxy}phenyl)-N-{2-[(4-fluorophenyl)methyl]-1,3-dioxo-2,3-dihydro-1H-isoindol-5-yl}acetamide (**12m;** ZHAWOC5682)

<u>NMR</u>

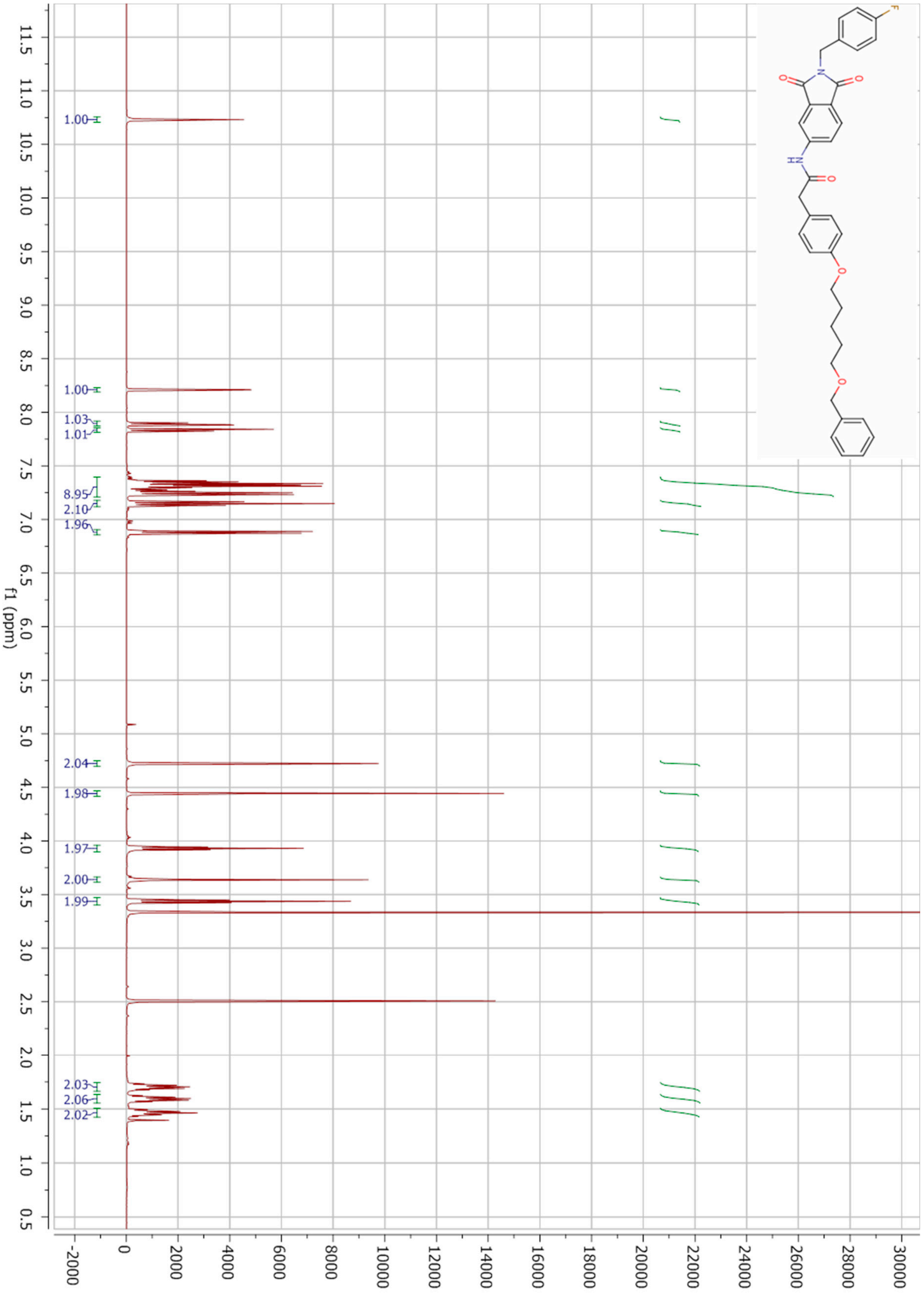

2-(4-{[5-(benzyloxy)pentyl]oxy}phenyl)-N-{2-[(4-fluorophenyl)methyl]-1,3-dioxo-2,3-dihydro-1H-isoindol-5-yl}acetamide (**12m;** ZHAWOC5682)

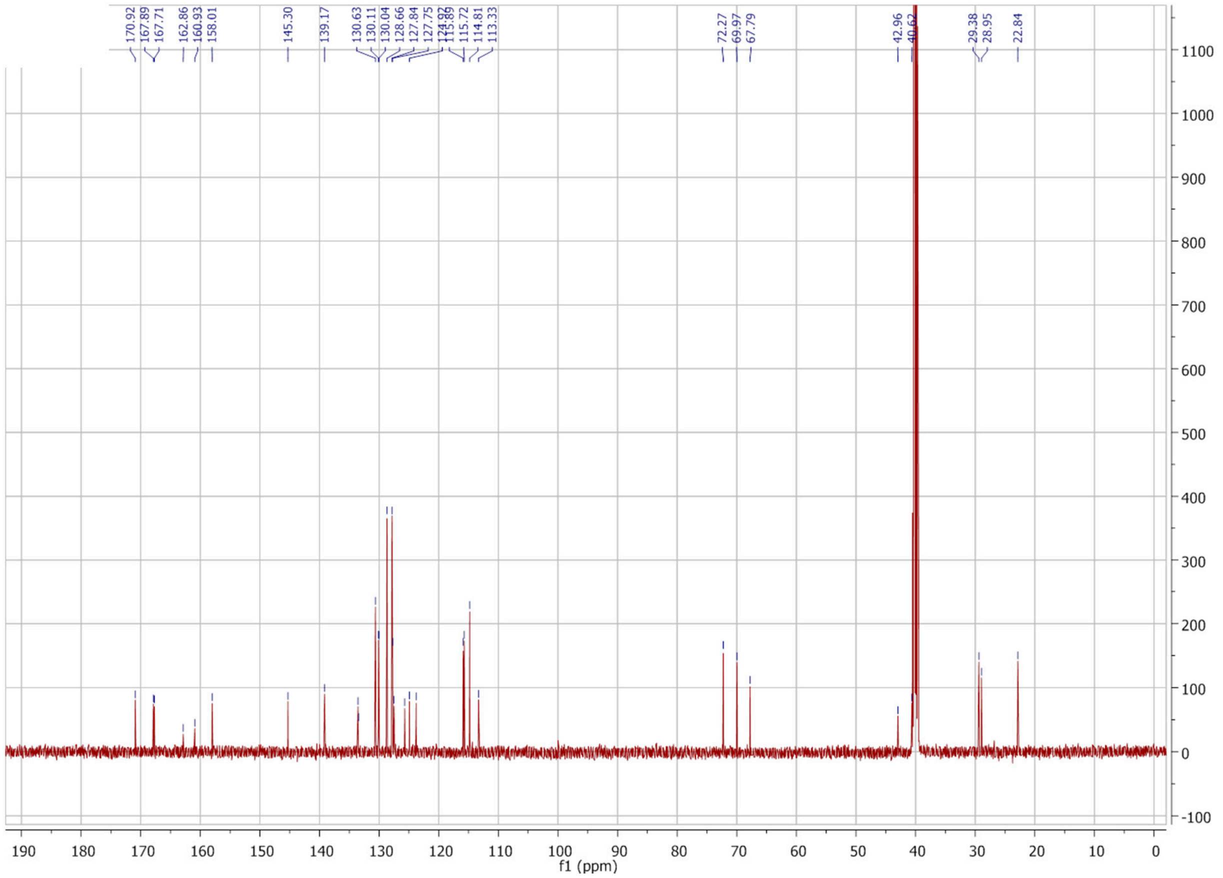

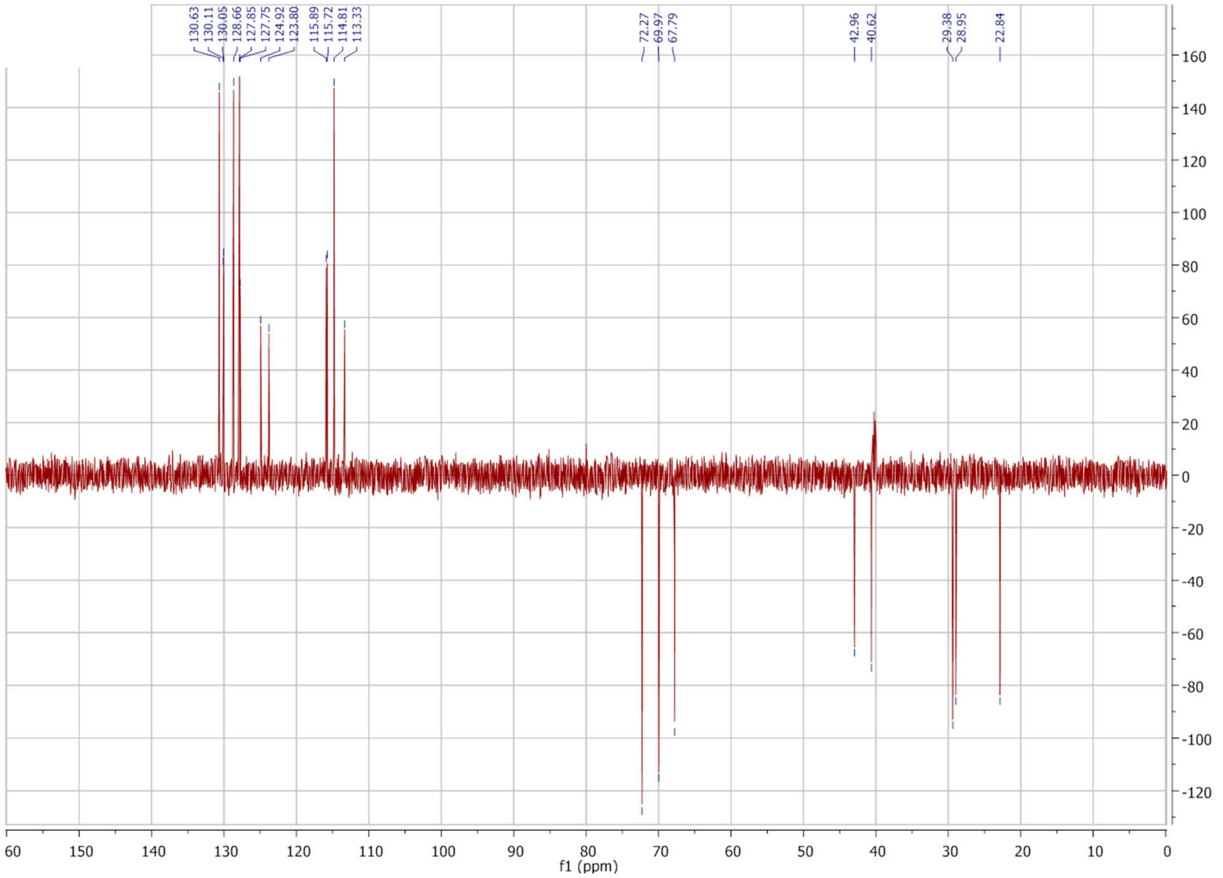

2-(4-{[6-(benzyloxy)hexyl]oxy}phenyl)-N-{2-[(4-fluorophenyl)methyl]-1,3-dioxo-2,3-dihydro-1H-isoindol-5-yl}acetamide (**12n**; ZHAWOC6640)

<u>NMR</u>

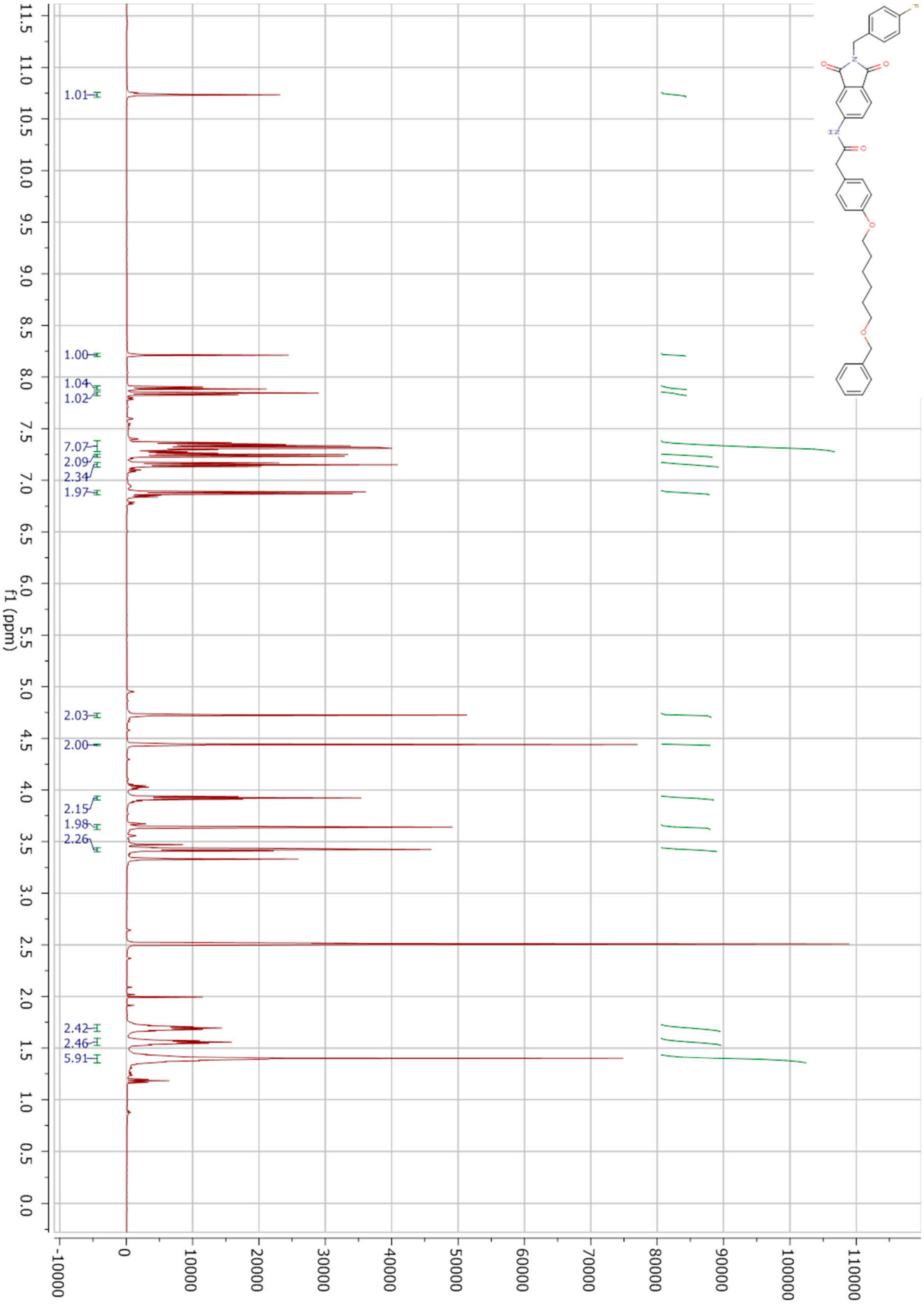

2-(4-{[6-(benzyloxy)hexyl]oxy}phenyl)-N-{2-[(4-fluorophenyl)methyl]-1,3-dioxo-2,3-dihydro-1H-isoindol-5-yl}acetamide (**12n**; ZHAWOC6640)

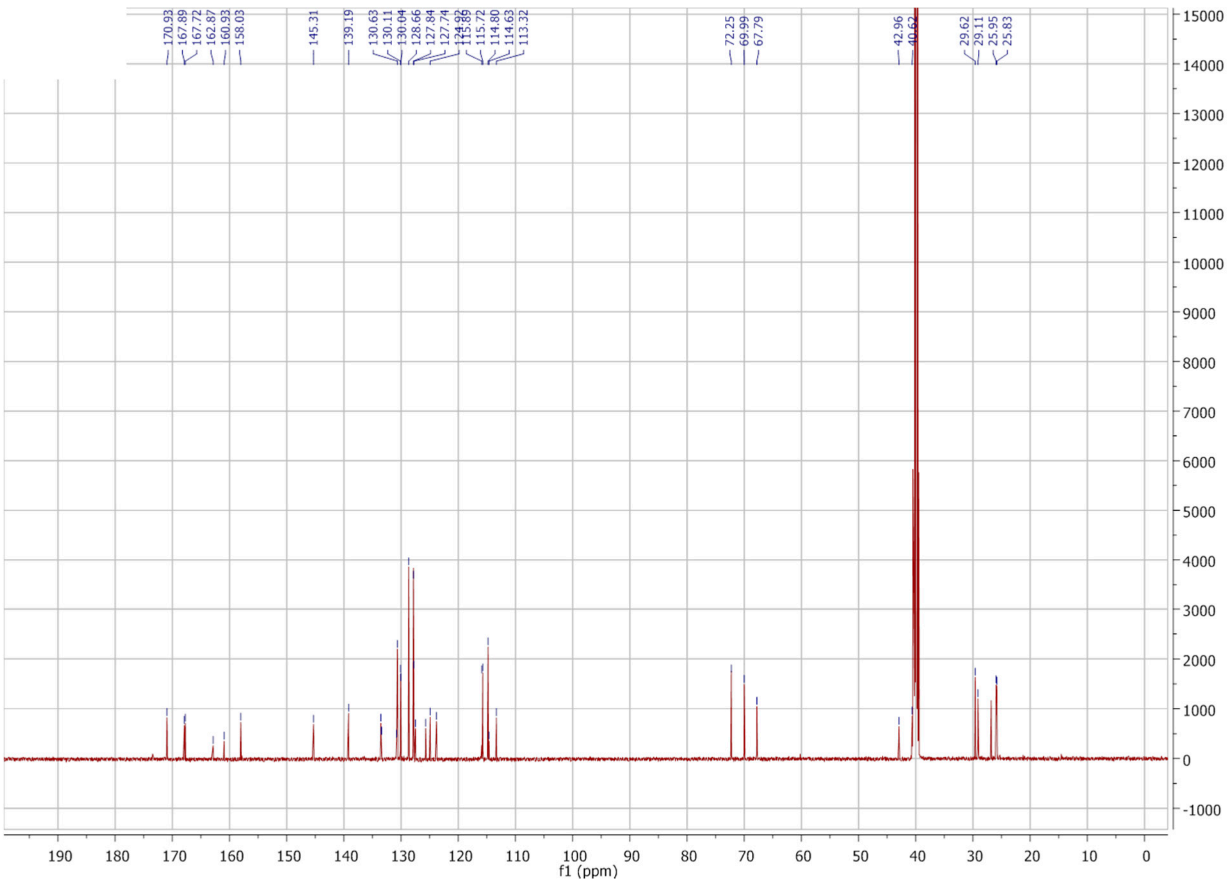

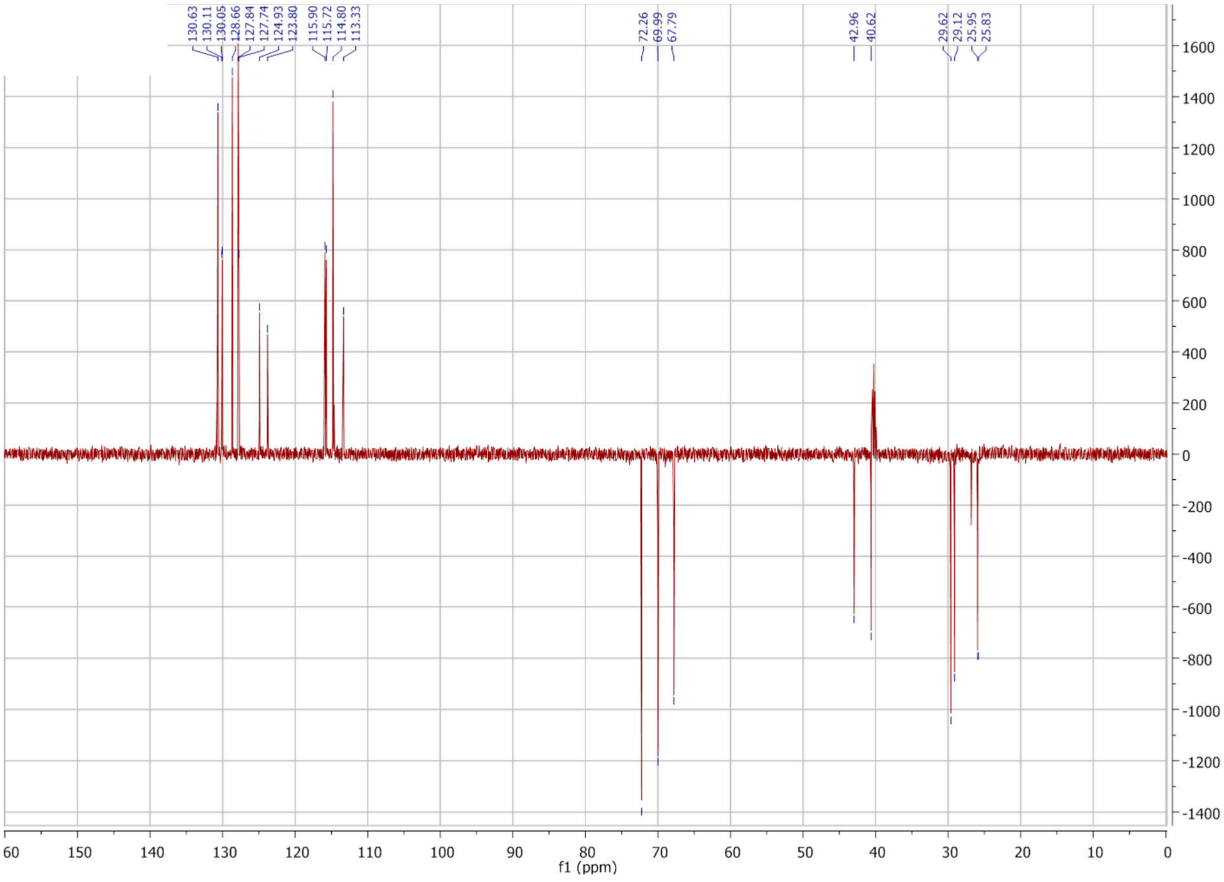

2-(4-{[7-(benzyloxy)heptyl]oxy}phenyl)-N-{2-[(4-fluorophenyl)methyl]-1,3-dioxo-2,3-dihydro-1H-isoindol-5-yl}acetamide (**12o**; ZHAWOC6635)

NMR

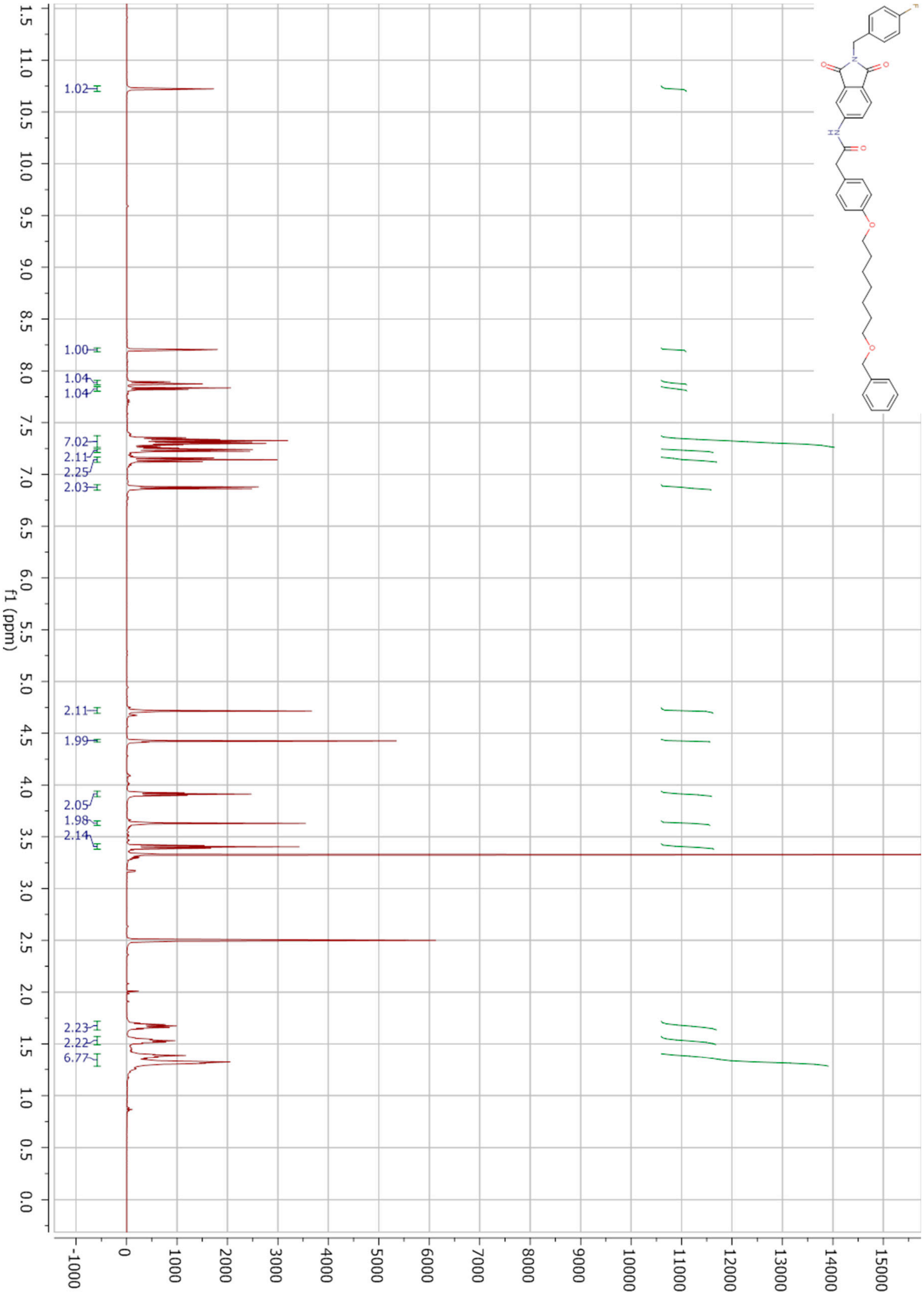

2-(4-{[7-(benzyloxy)heptyl]oxy}phenyl)-N-{2-[(4-fluorophenyl)methyl]-1,3-dioxo-2,3-dihydro-1H-isoindol-5-yl}acetamide (**12o**; ZHAWOC6635)

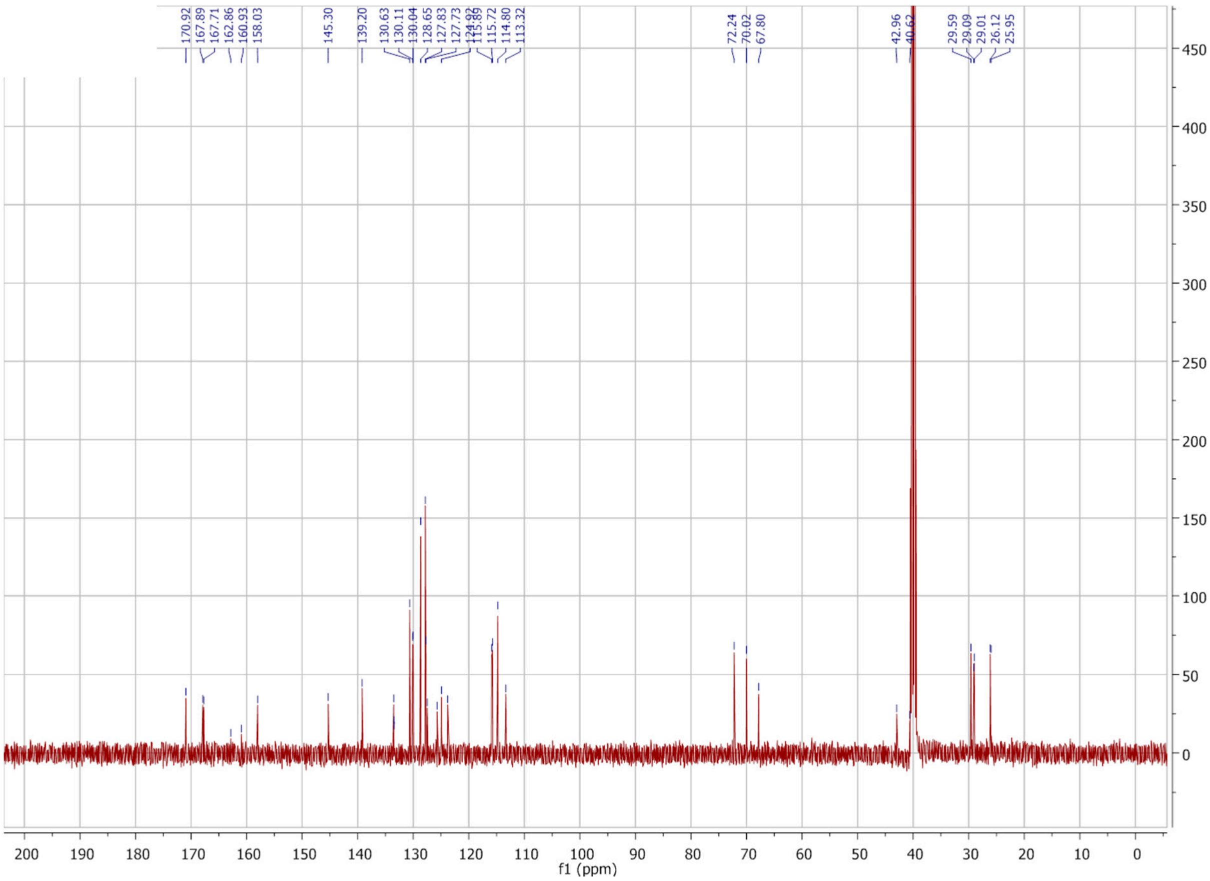

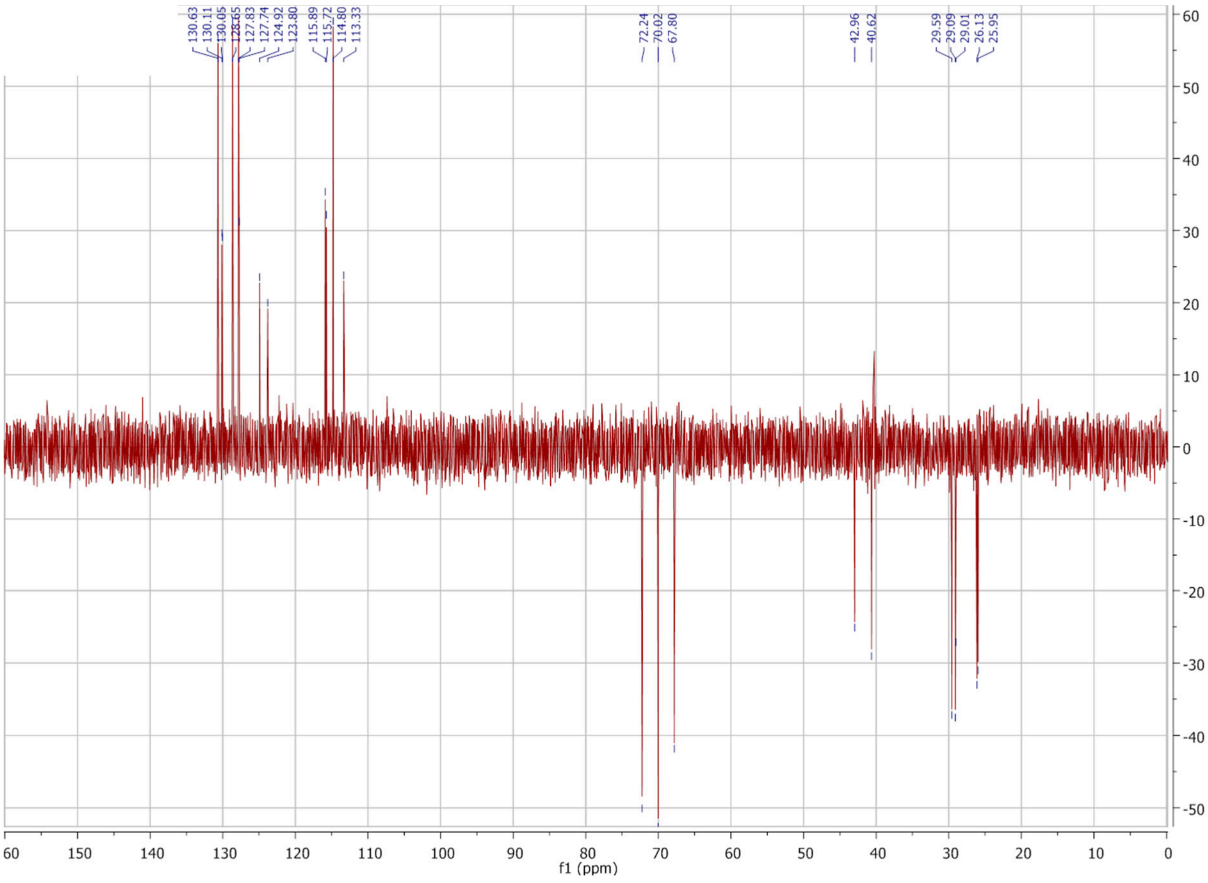

2-(4-{[8-(benzyloxy)octyl]oxy}phenyl)-N-{2-[(4-fluorophenyl)methyl]-1,3-dioxo-2,3-dihydro-1H-isoindol-5-yl}acetamide (**12p**; ZHAWOC6638)

<u>NMR</u>

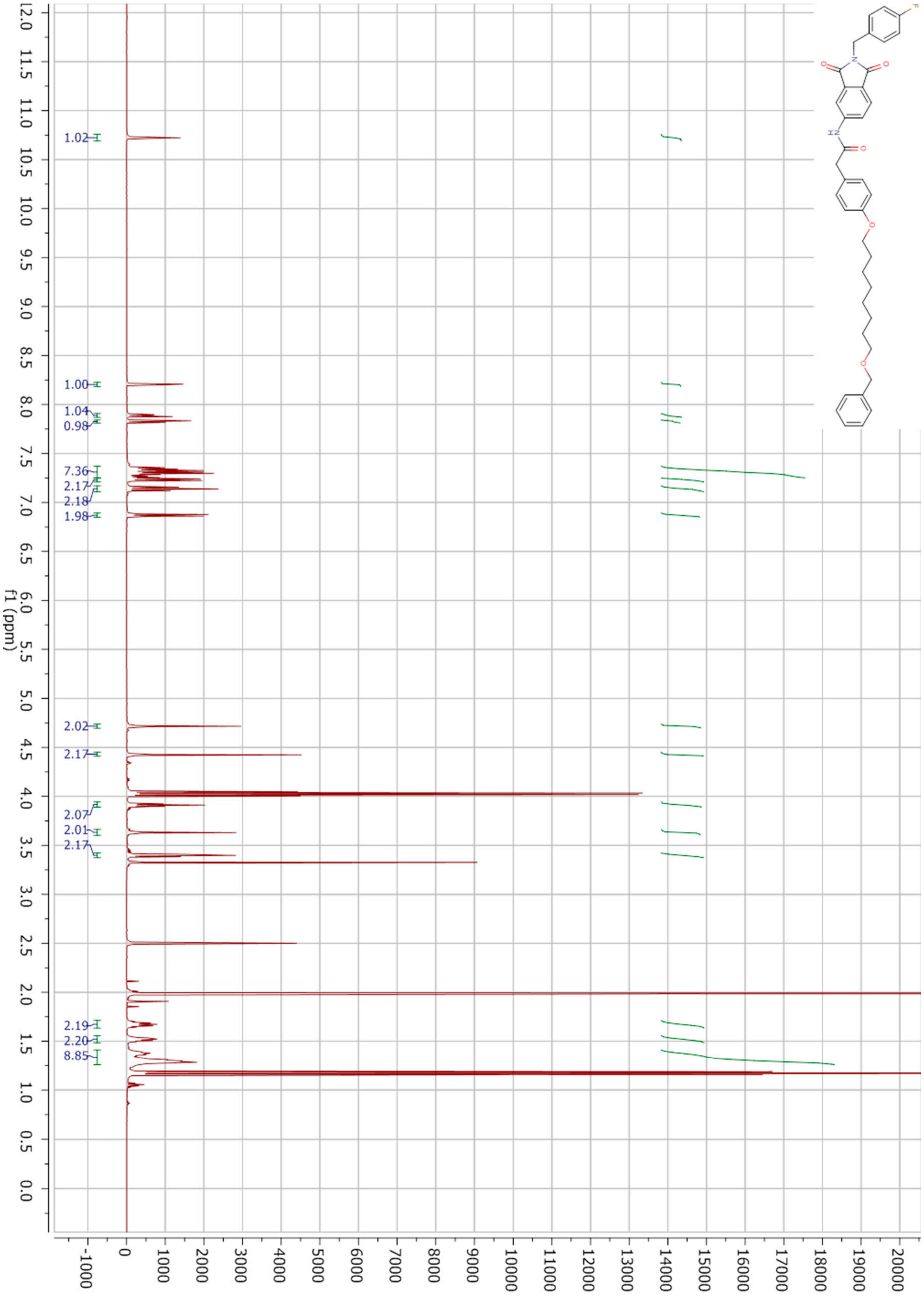

2-(4-{[8-(benzyloxy)octyl]oxy}phenyl)-N-{2-[(4-fluorophenyl)methyl]-1,3-dioxo-2,3-dihydro-1H-isoindol-5-yl}acetamide (**12p**; ZHAWOC6638)

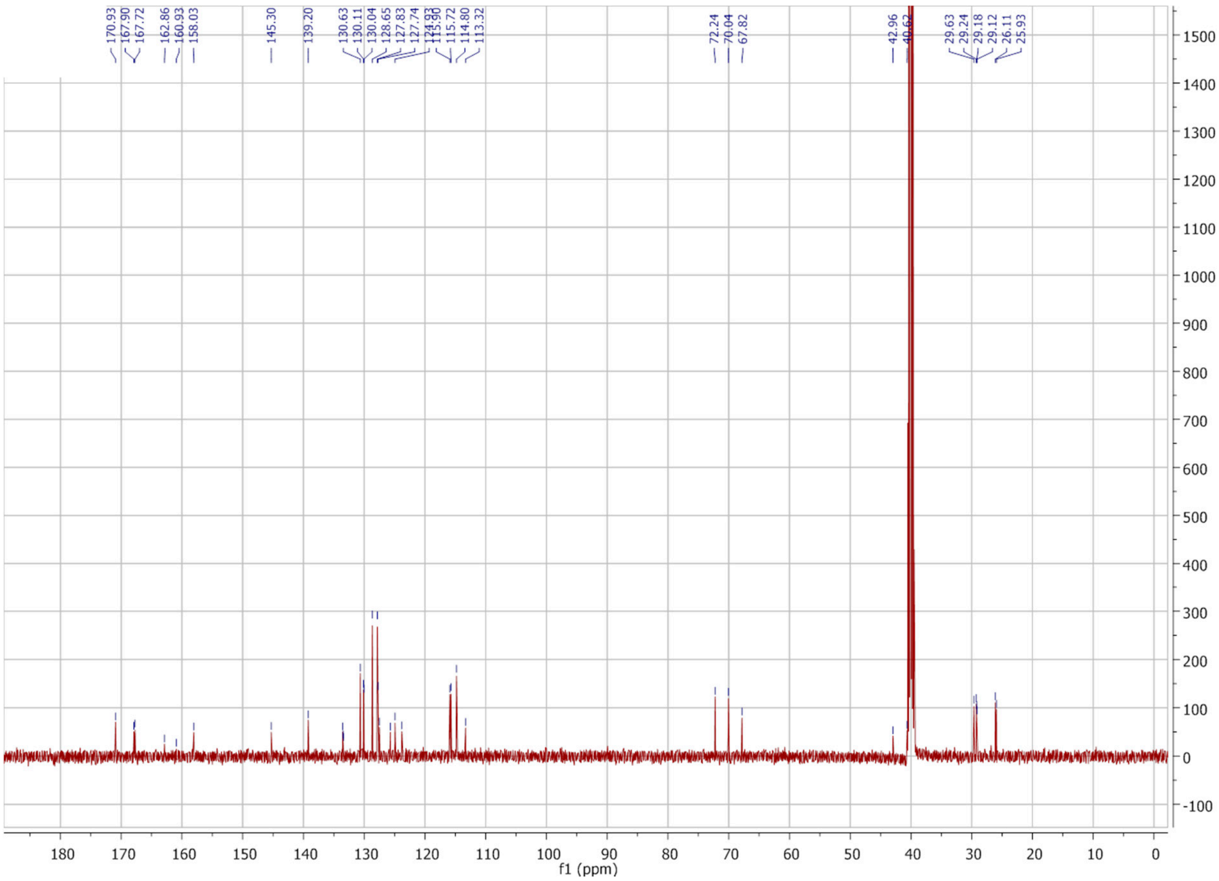

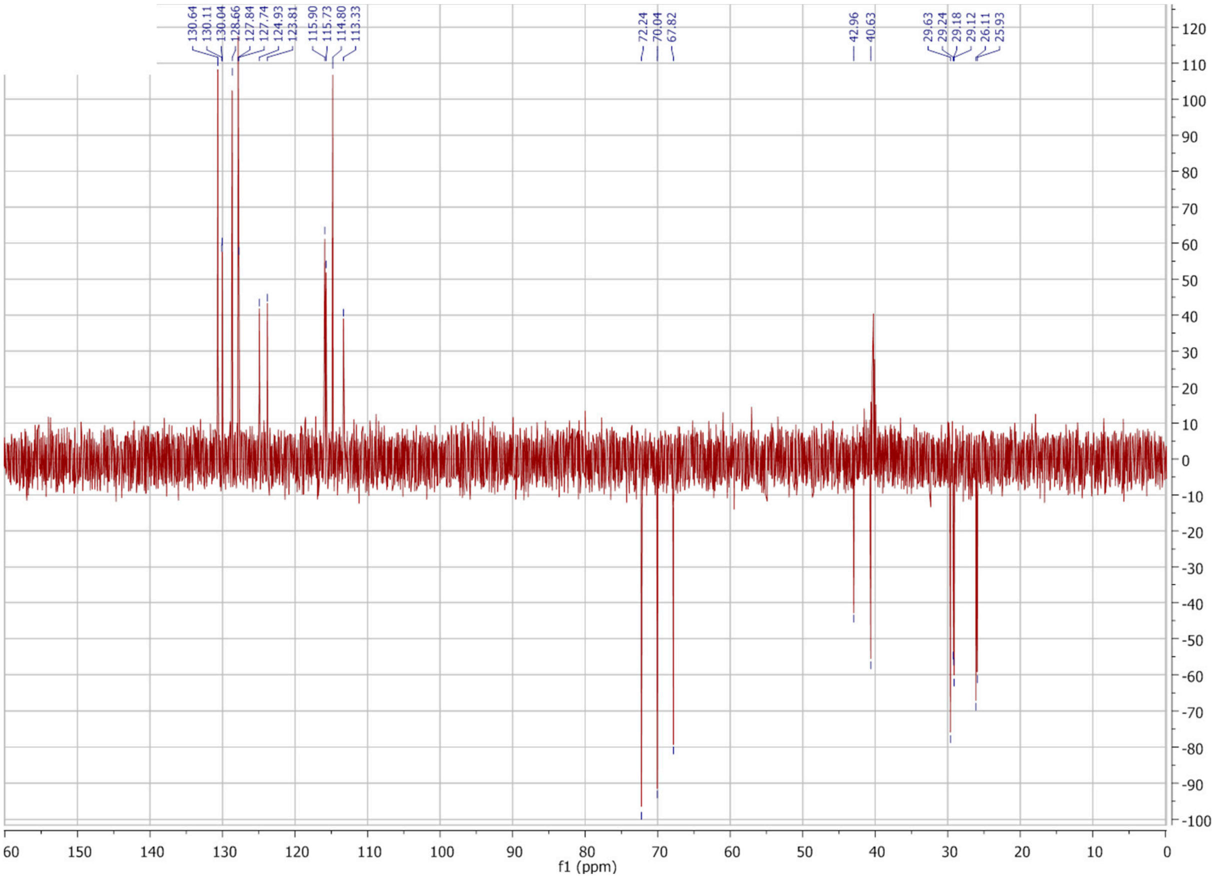

2-{4-[(5-hydroxypentyl)oxy]phenyl}-N-(2-methyl-1,3-dioxo-2,3-dihydro-1H-isoindol-5-yl)acetamide (**13a**; ZHAWOC6648)

<u>NMR</u>

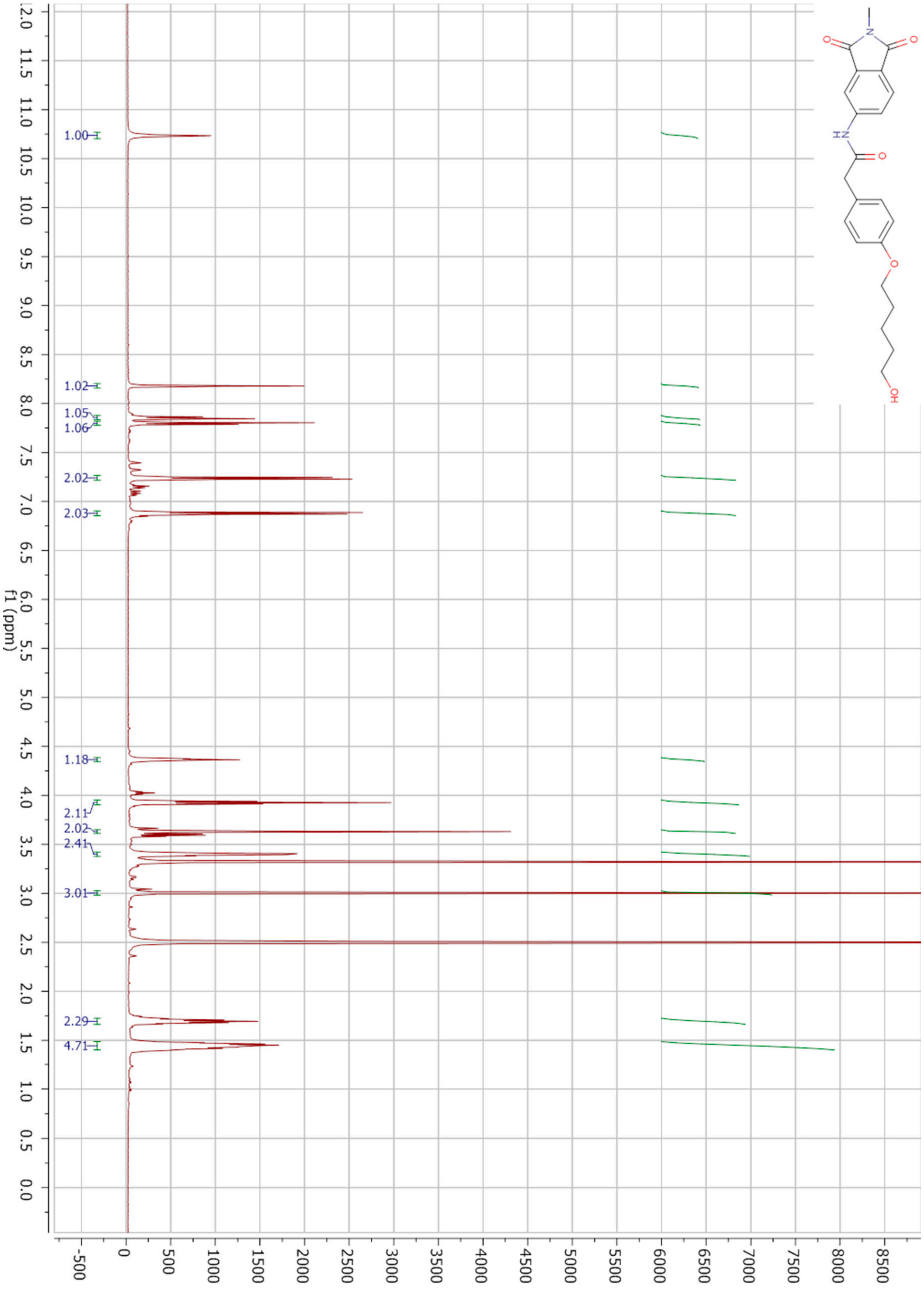

2-{4-[(5-hydroxypentyl)oxy]phenyl}-N-(2-methyl-1,3-dioxo-2,3-dihydro-1H-isoindol-5-yl)acetamide (**13a**; ZHAWOC6648)

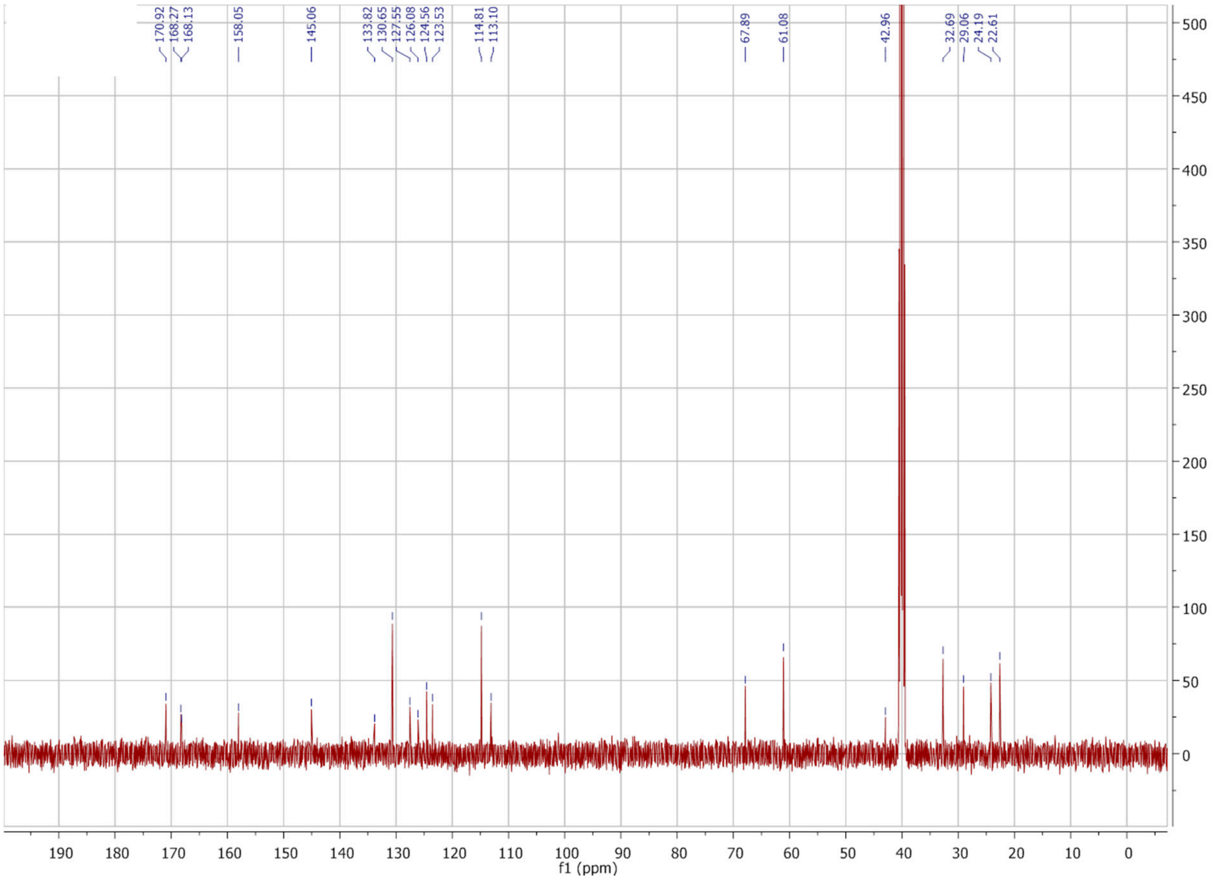

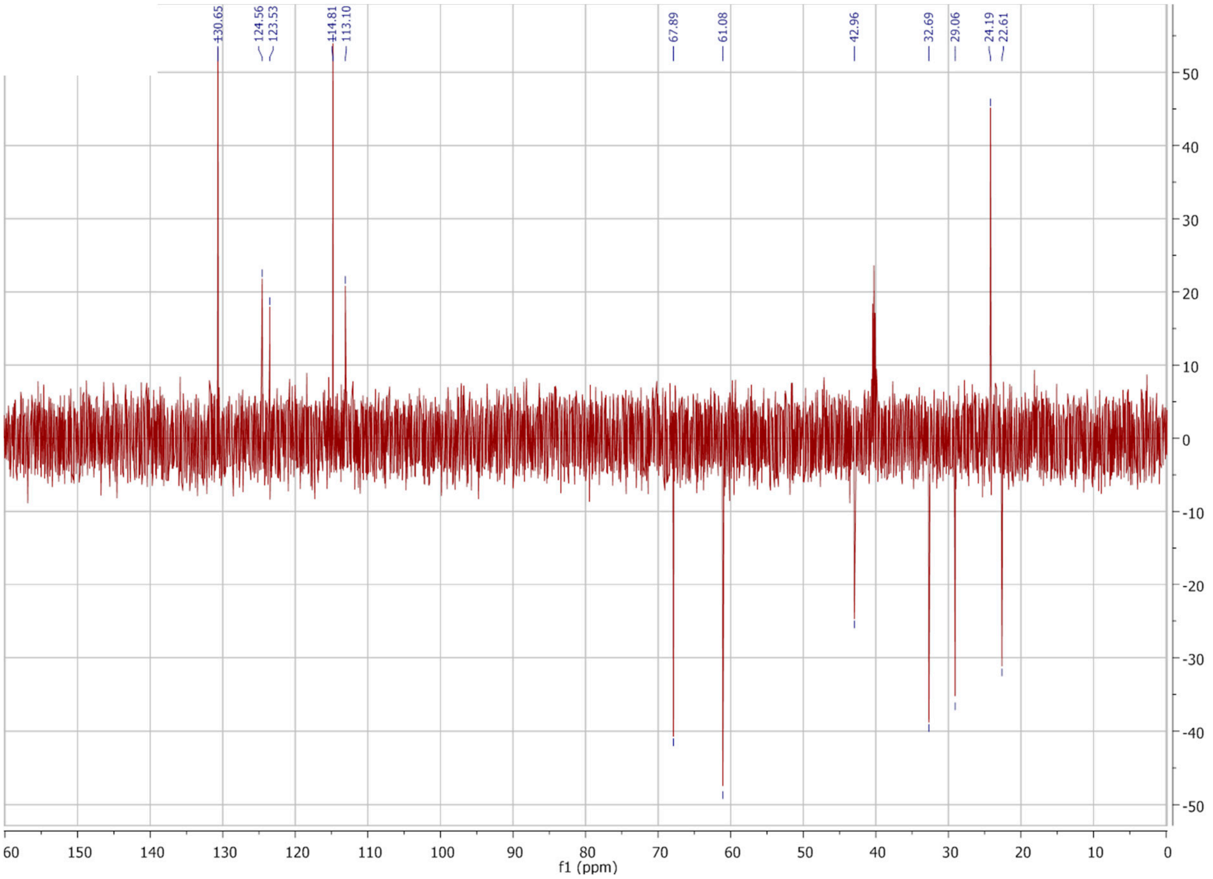

N-(2-benzyl-1,3-dioxo-2,3-dihydro-1H-isoindol-5-yl)-2-[4-(2-hydroxyethoxy)phenyl]acetamide (**13b**; ZHAWOC5473)

<u>NMR</u>

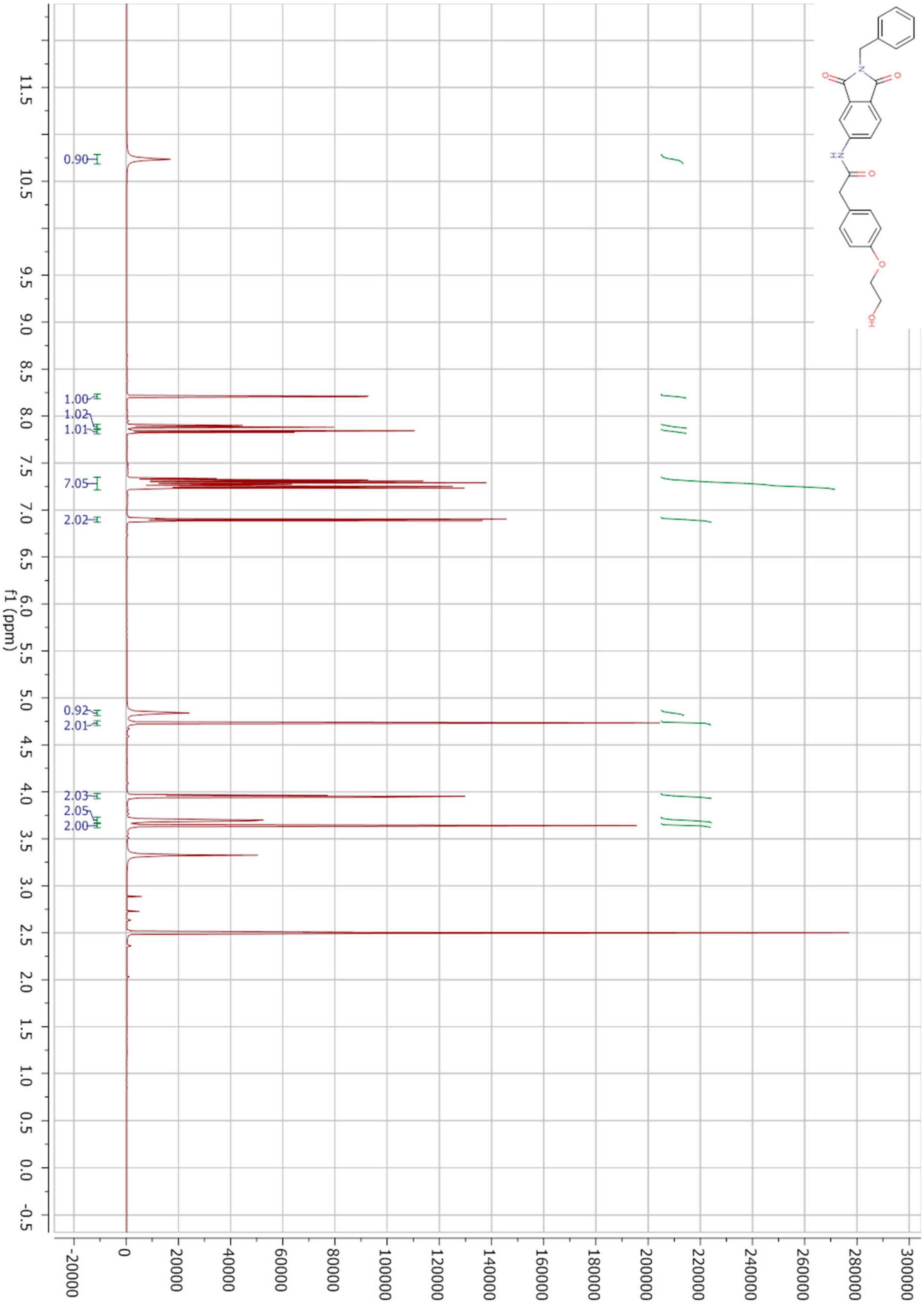

N-(2-benzyl-1,3-dioxo-2,3-dihydro-1H-isoindol-5-yl)-2-[4-(2-hydroxyethoxy)phenyl]acetamide (**13b**; ZHAWOC5473)

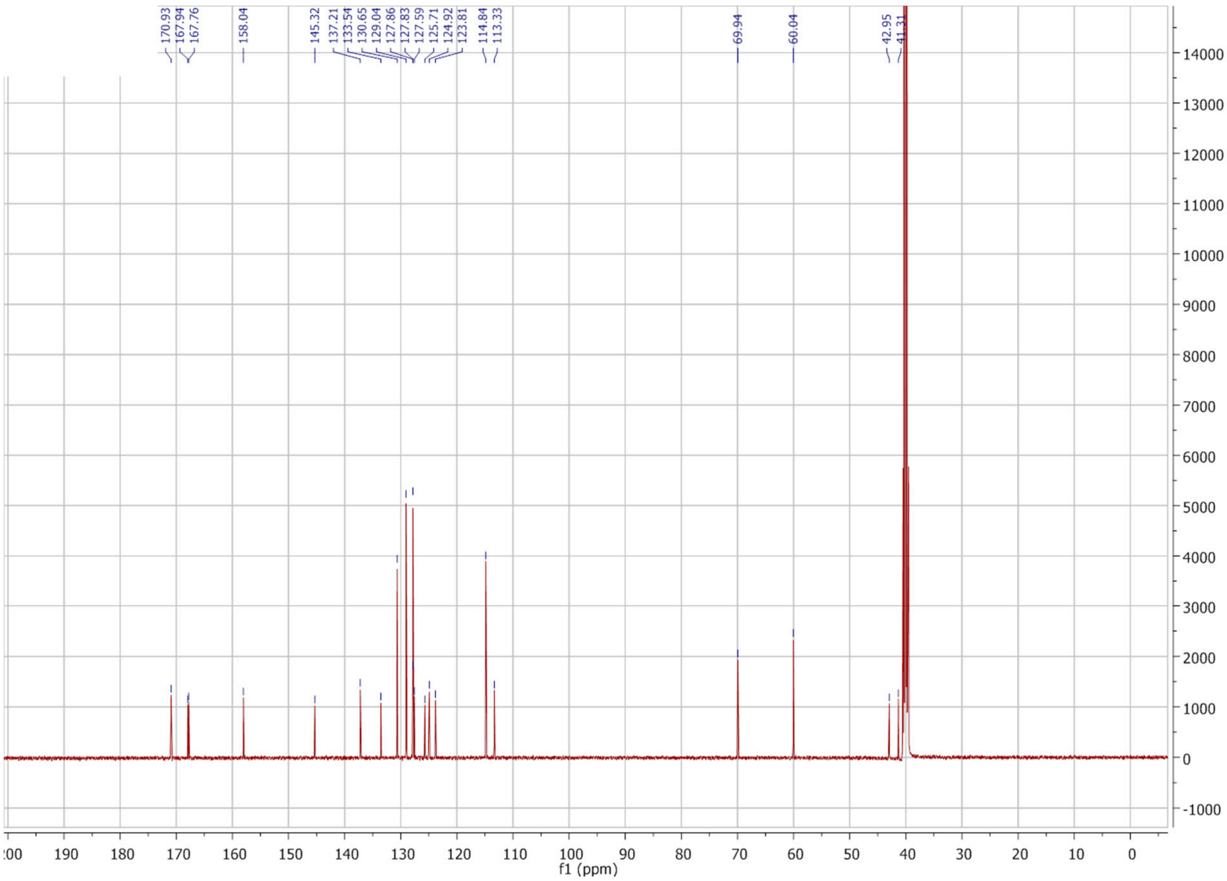

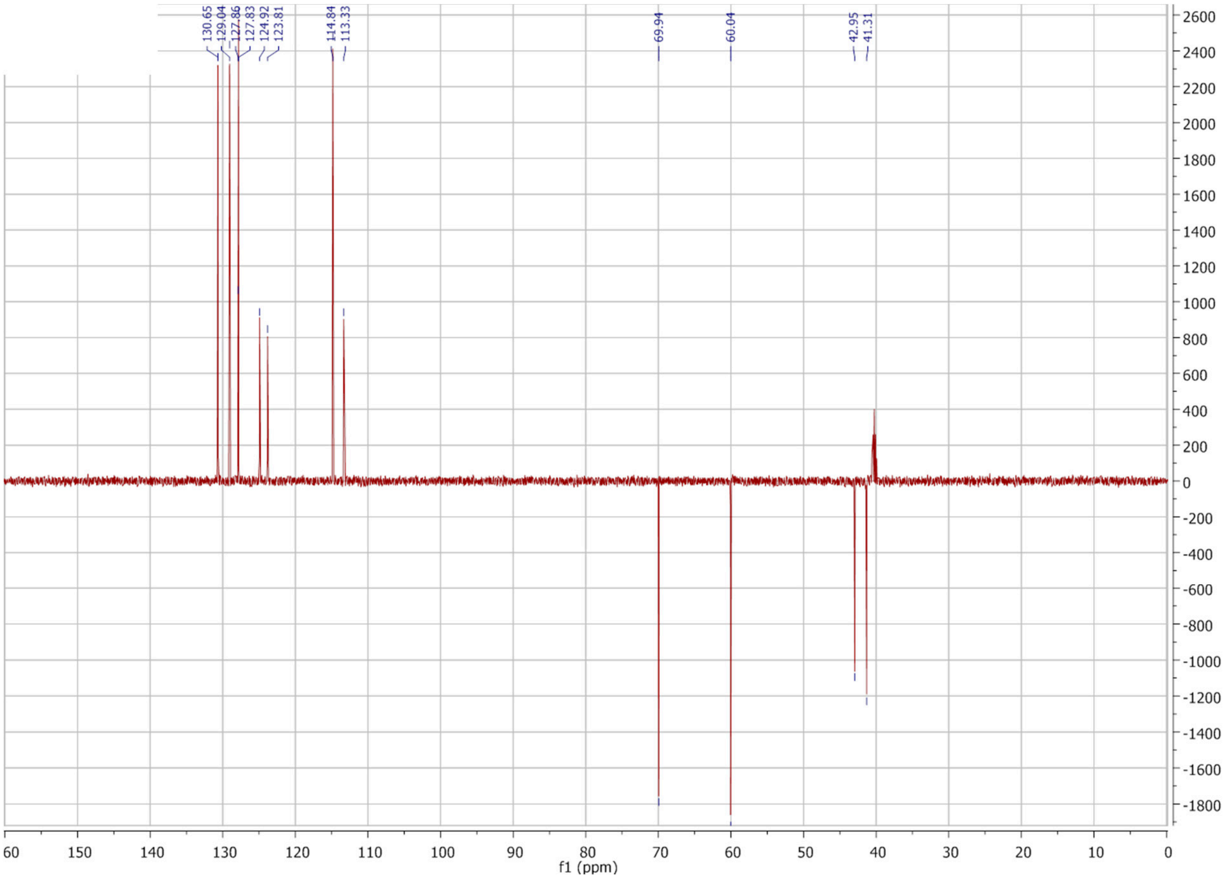

N-(2-benzyl-1,3-dioxo-2,3-dihydro-1H-isoindol-5-yl)-2-[4-(3-hydroxypropoxy)phenyl]acetamide (**13c;** ZHAWOC4512)

<u>NMR</u>

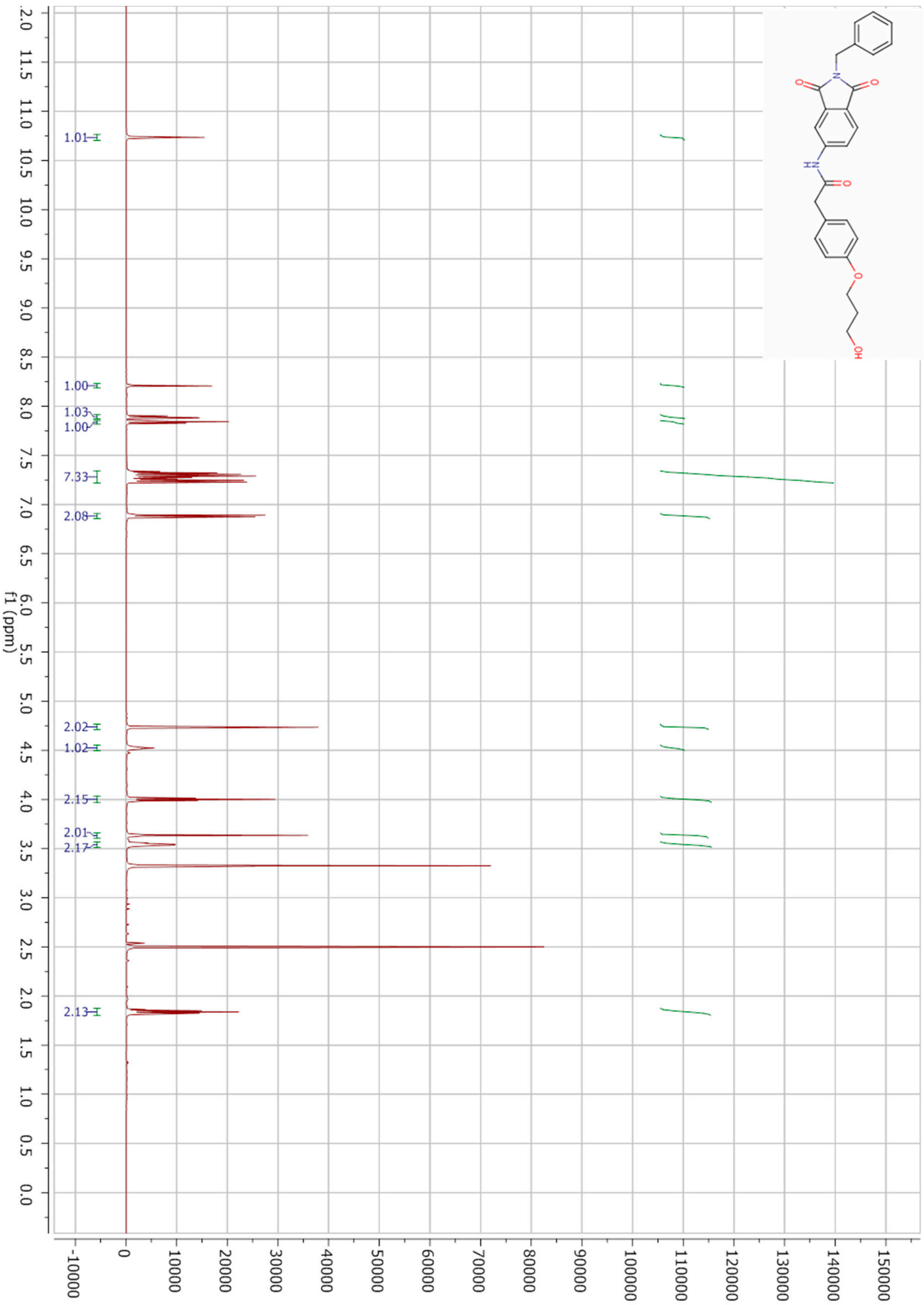

N-(2-benzyl-1,3-dioxo-2,3-dihydro-1H-isoindol-5-yl)-2-[4-(3-hydroxypropoxy)phenyl]acetamide (**13c;** ZHAWOC4512)

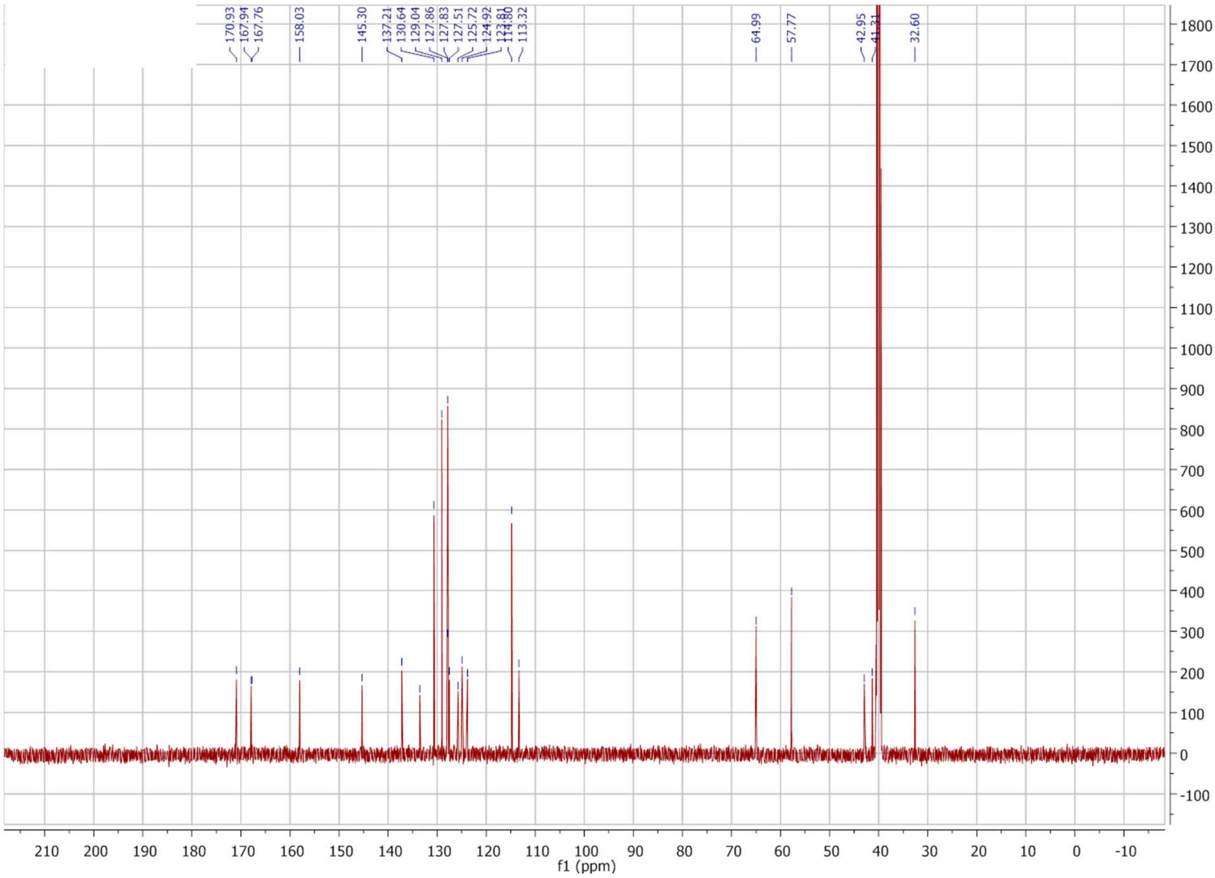

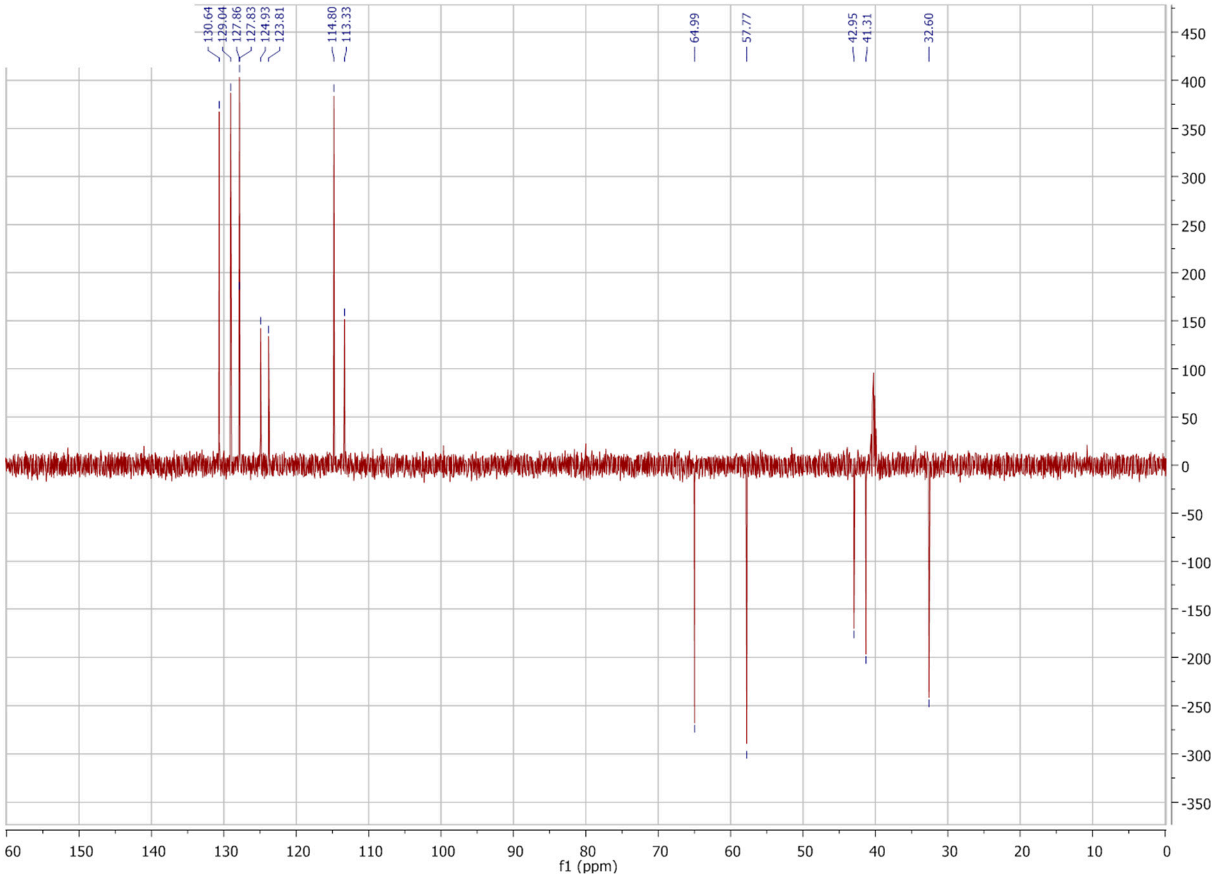

N-(2-benzyl-1,3-dioxo-2,3-dihydro-1H-isoindol-5-yl)-2-[4-(4-hydroxybutoxy)phenyl]acetamide (**13d;** ZHAWOC4753)

<u>NMR</u>

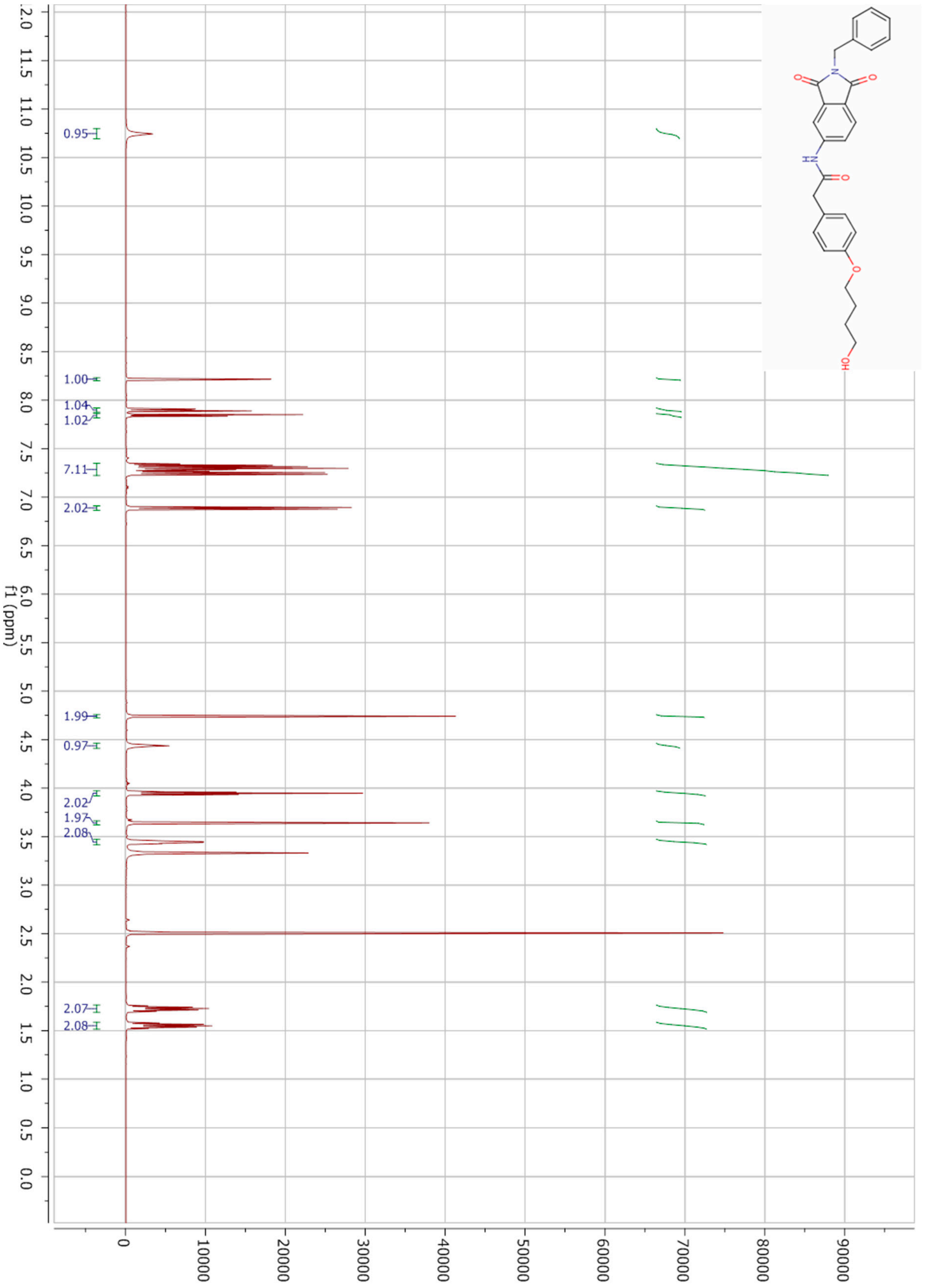

N-(2-benzyl-1,3-dioxo-2,3-dihydro-1H-isoindol-5-yl)-2-[4-(4-hydroxybutoxy)phenyl]acetamide (**13d;** ZHAWOC4753)

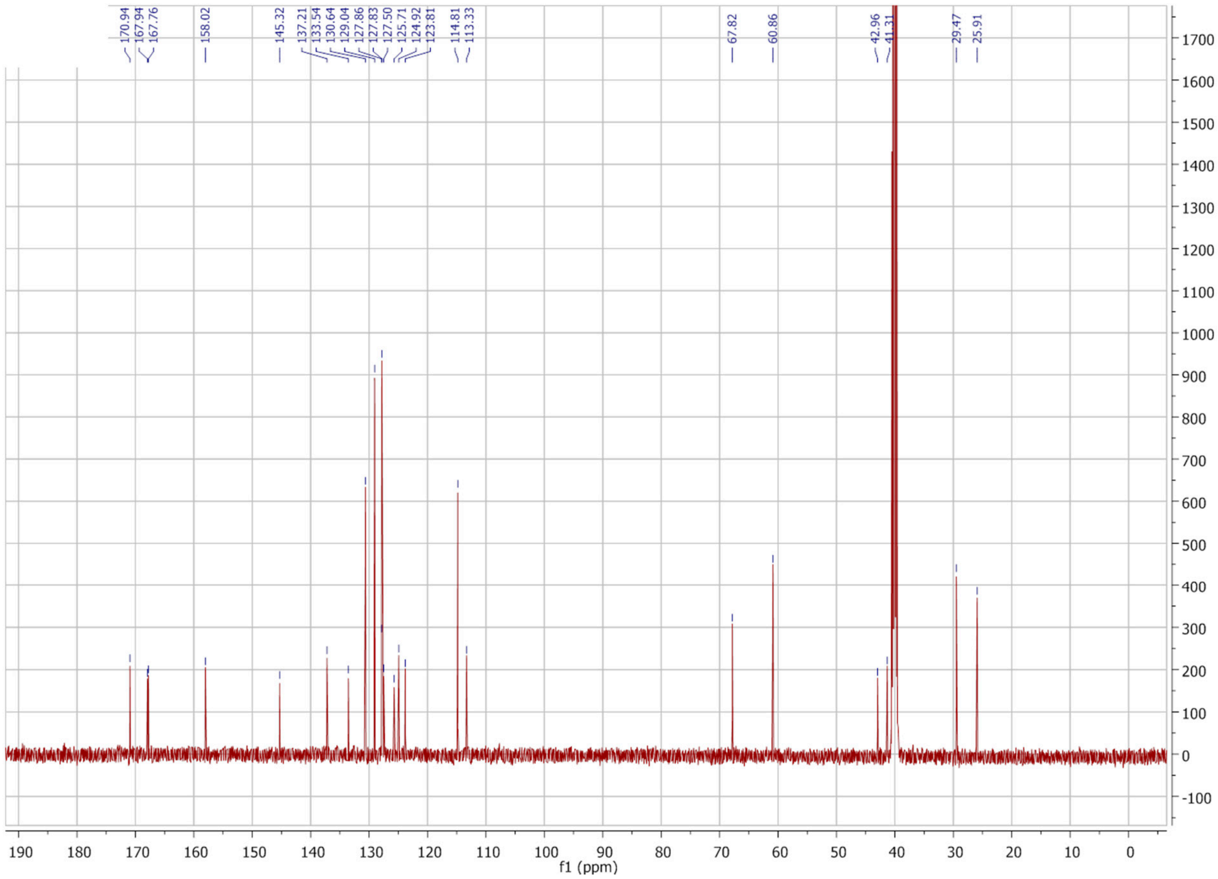

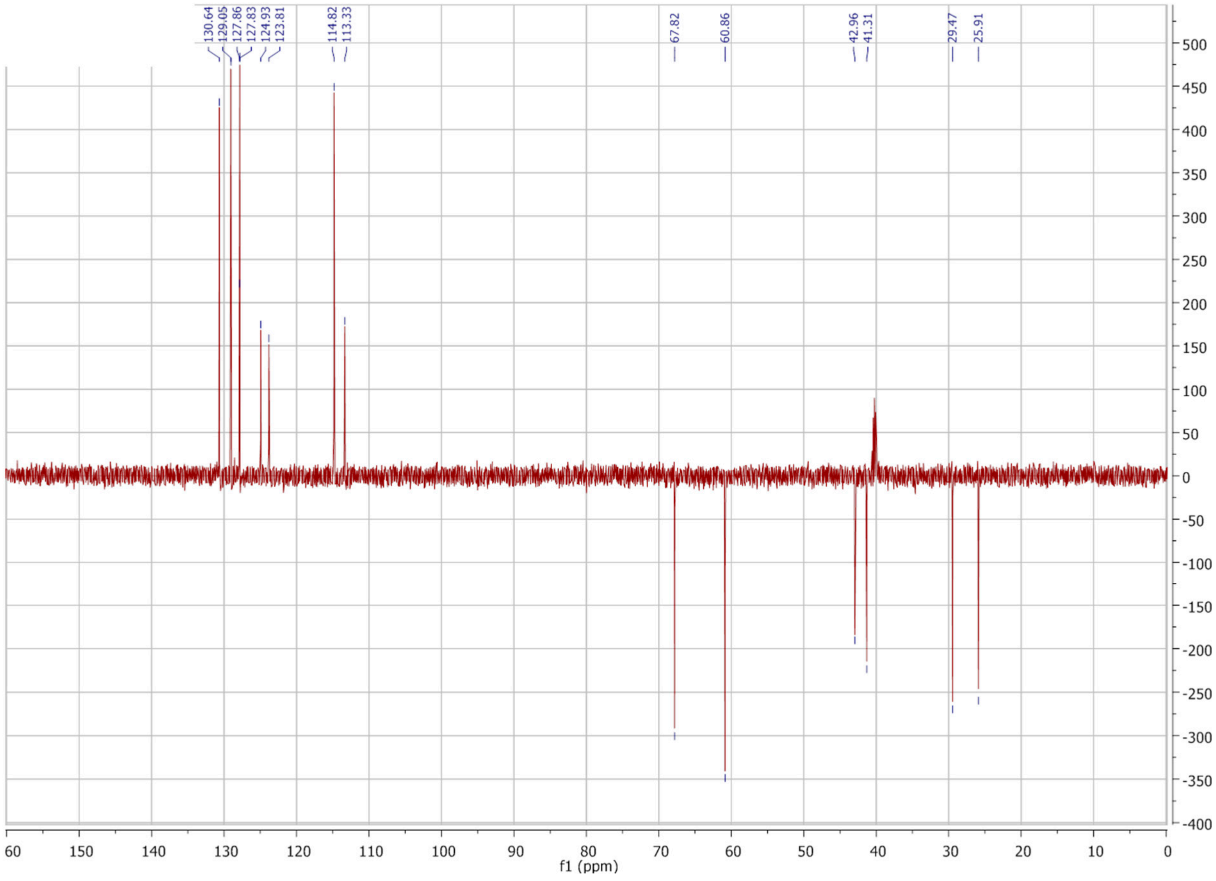

N-(2-benzyl-1,3-dioxo-2,3-dihydro-1H-isoindol-5-yl)-2-{4-[(5-hydroxypentyl)oxy]phenyl}acetamide (**13e;** ZHAWOC5130)

<u>NMR</u>

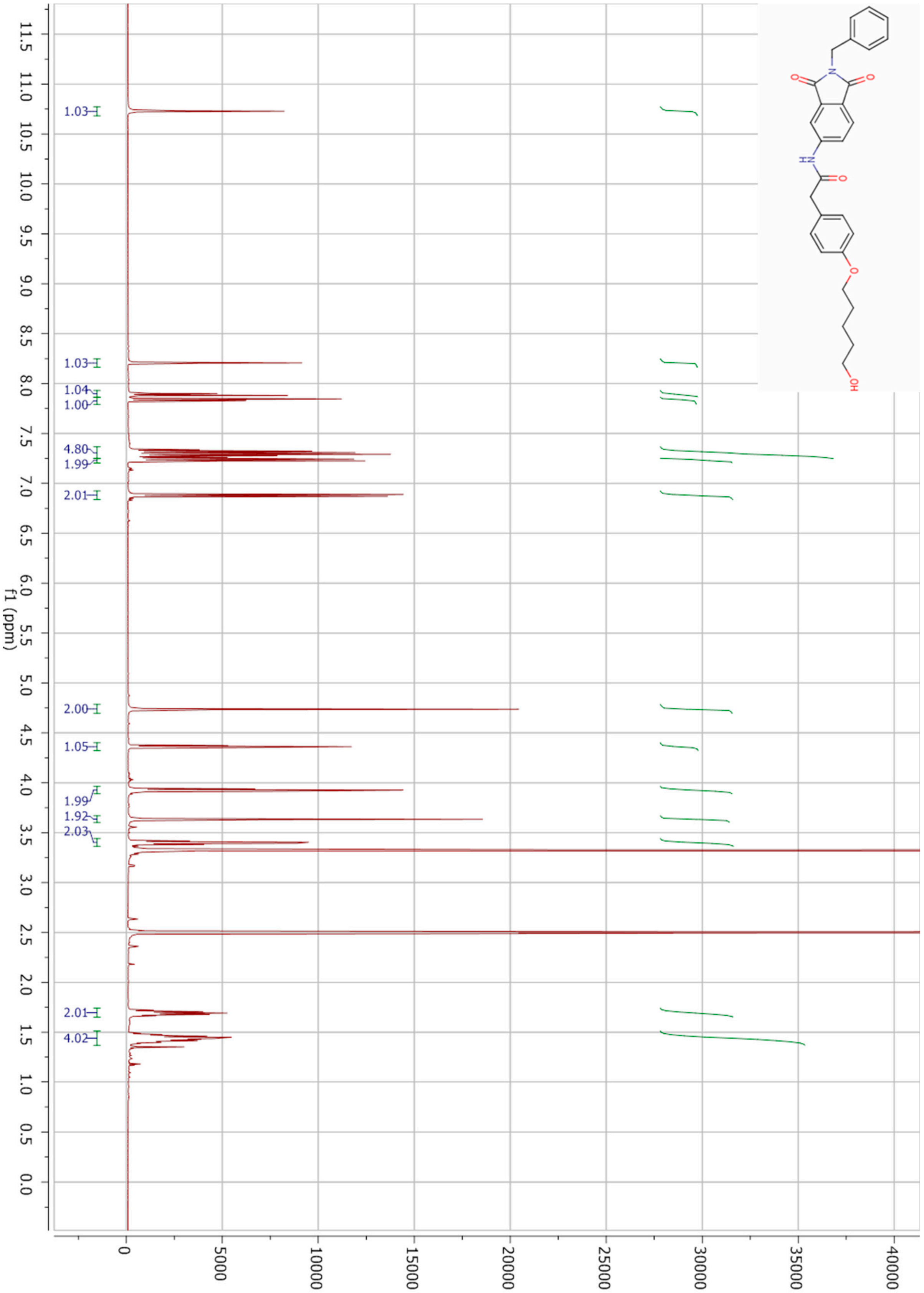

N-(2-benzyl-1,3-dioxo-2,3-dihydro-1H-isoindol-5-yl)-2-{4-[(5-hydroxypentyl)oxy]phenyl}acetamide (**13e;** ZHAWOC5130)

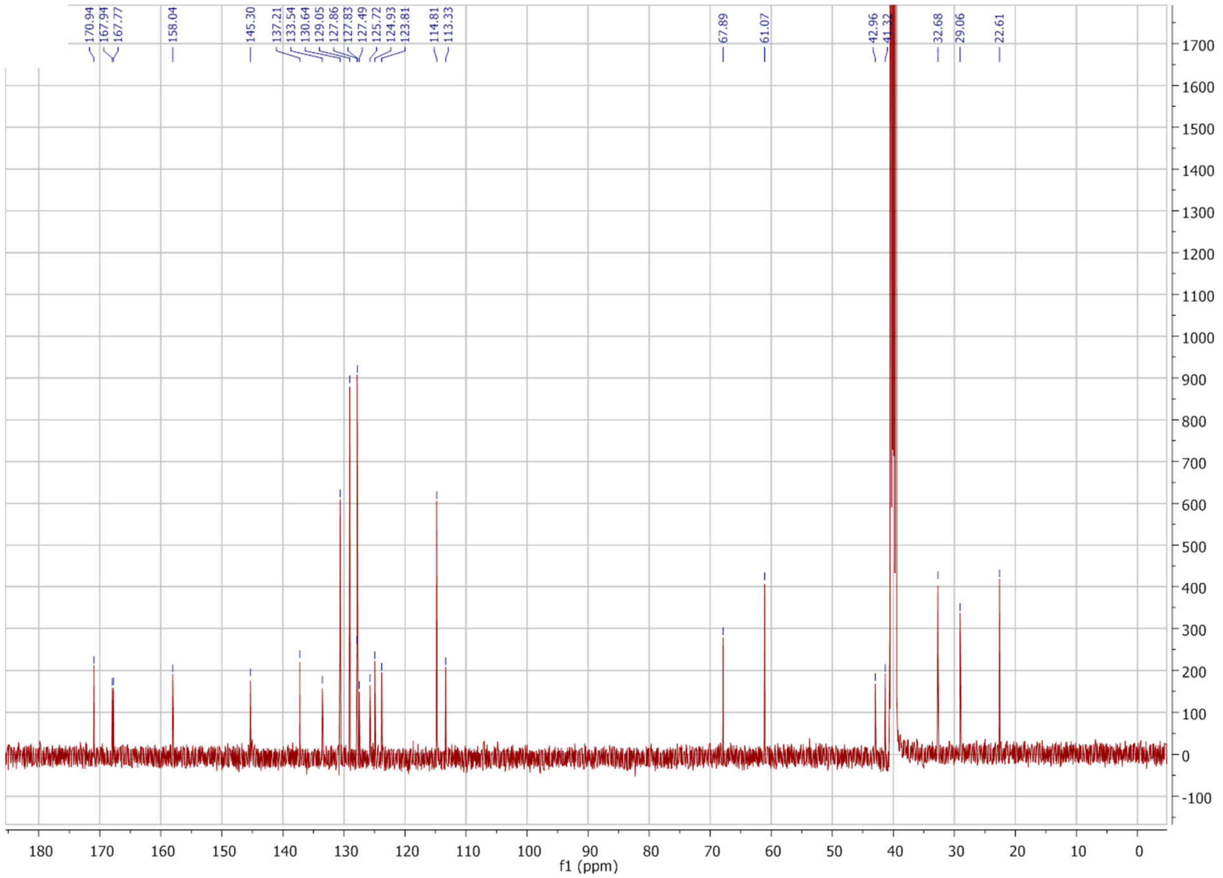

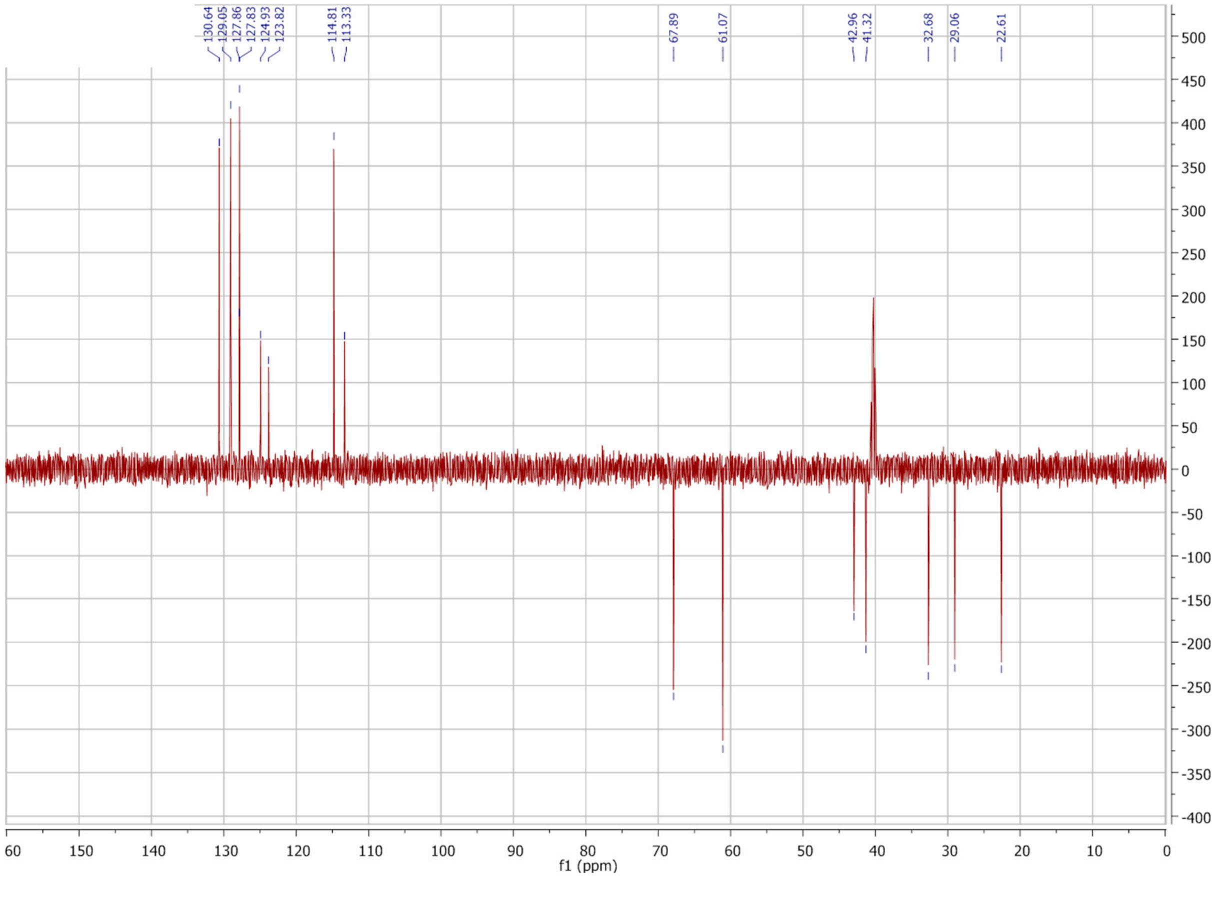

N-(2-benzyl-1,3-dioxo-2,3-dihydro-1H-isoindol-5-yl)-2-{4-[(6-hydroxyhexyl)oxy]phenyl}acetamide (**13f;** ZHAWOC5132)

<u>NMR</u>

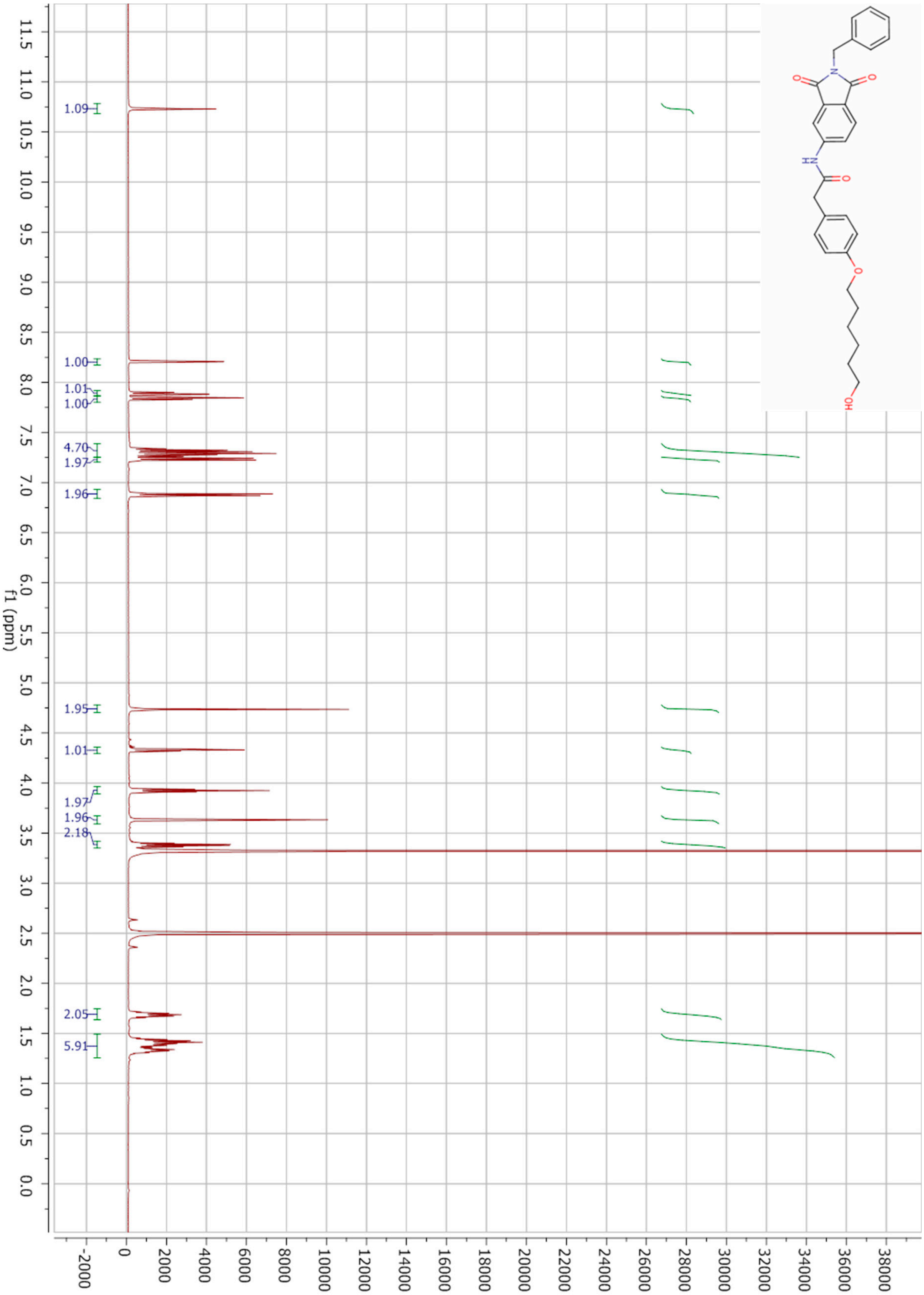

N-(2-benzyl-1,3-dioxo-2,3-dihydro-1H-isoindol-5-yl)-2-{4-[(6-hydroxyhexyl)oxy]phenyl}acetamide (**13f;** ZHAWOC5132)

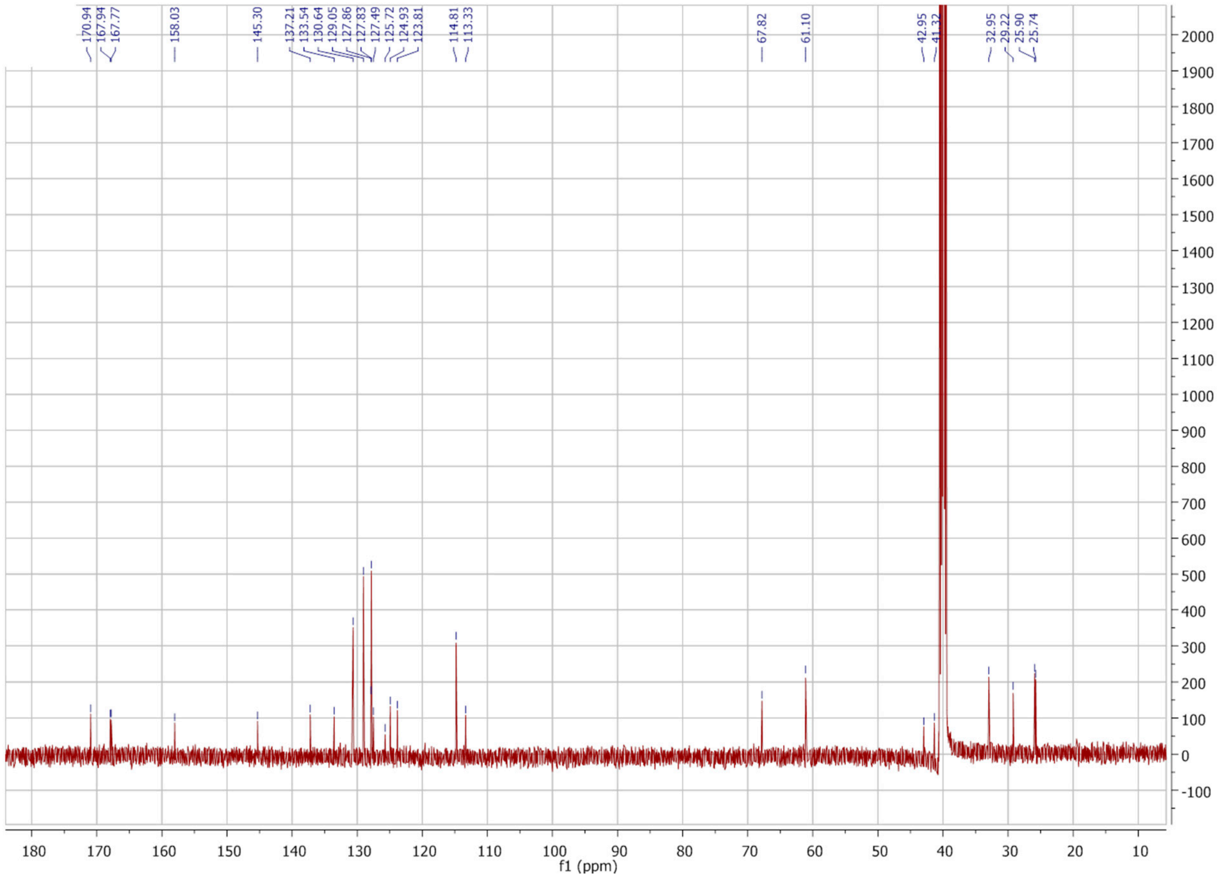

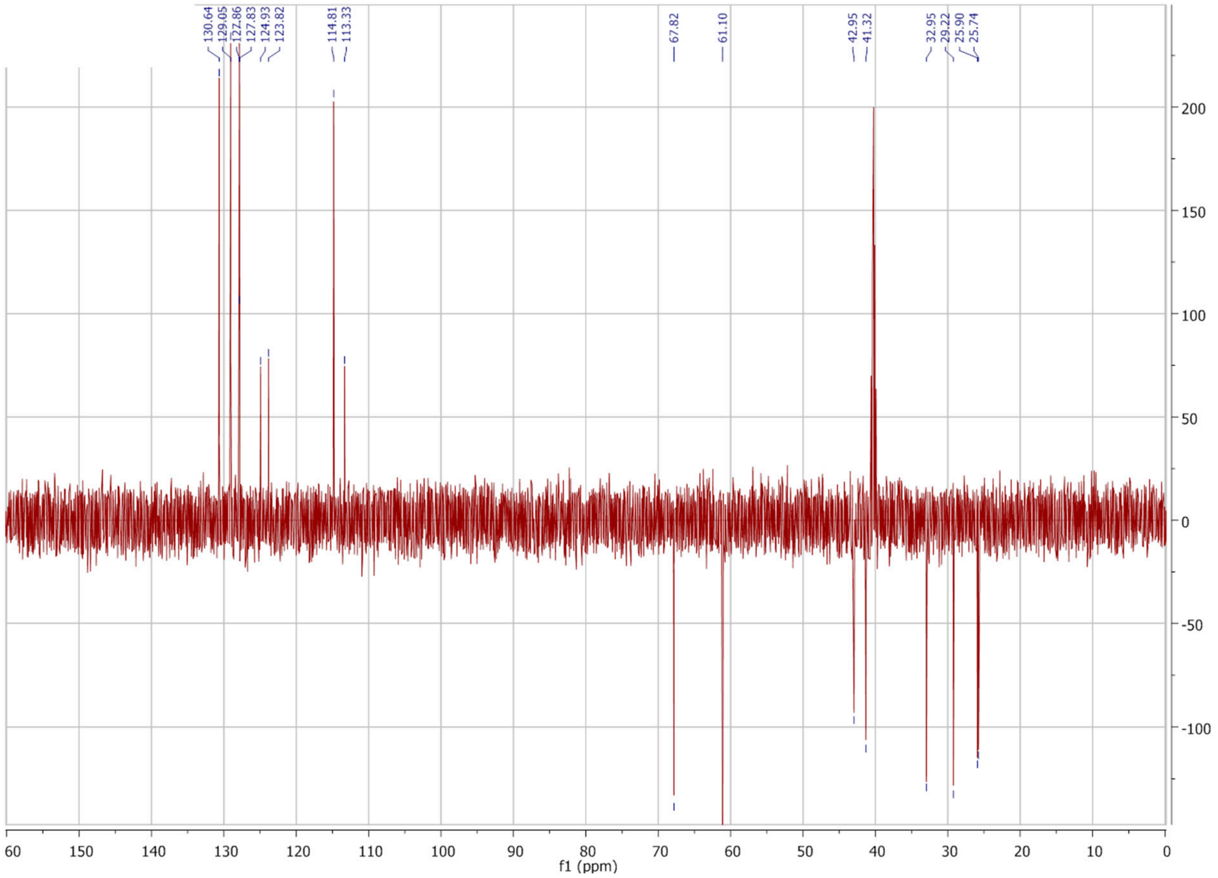

N-(2-benzyl-1,3-dioxo-2,3-dihydro-1H-isoindol-5-yl)-2-{4-[(7-hydroxyheptyl)oxy]phenyl}acetamide (**13g**; ZHAWOC7103)

<u>NMR</u>

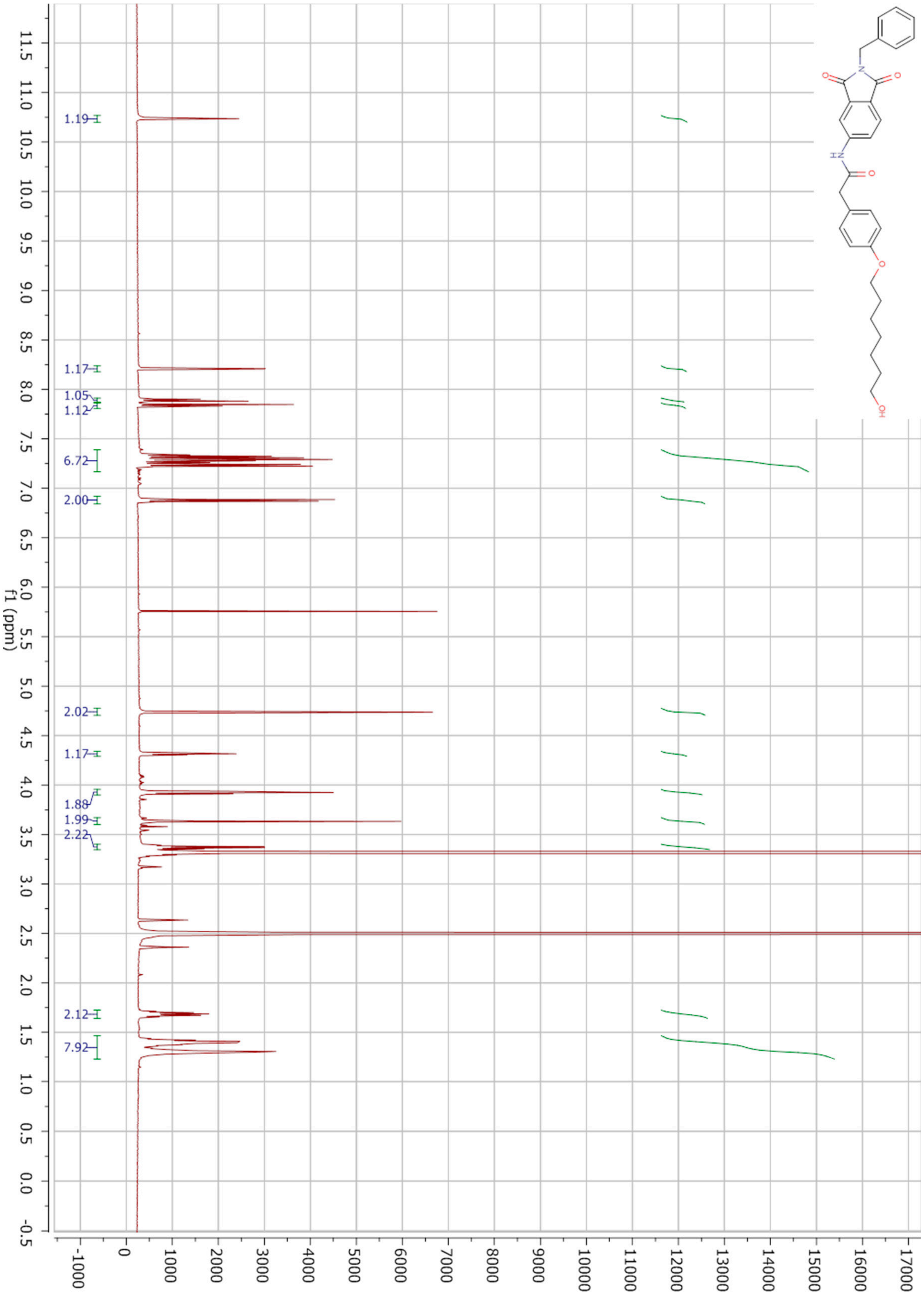

N-(2-benzyl-1,3-dioxo-2,3-dihydro-1H-isoindol-5-yl)-2-{4-[(7-hydroxyheptyl)oxy]phenyl}acetamide (**13g**; ZHAWOC7103)

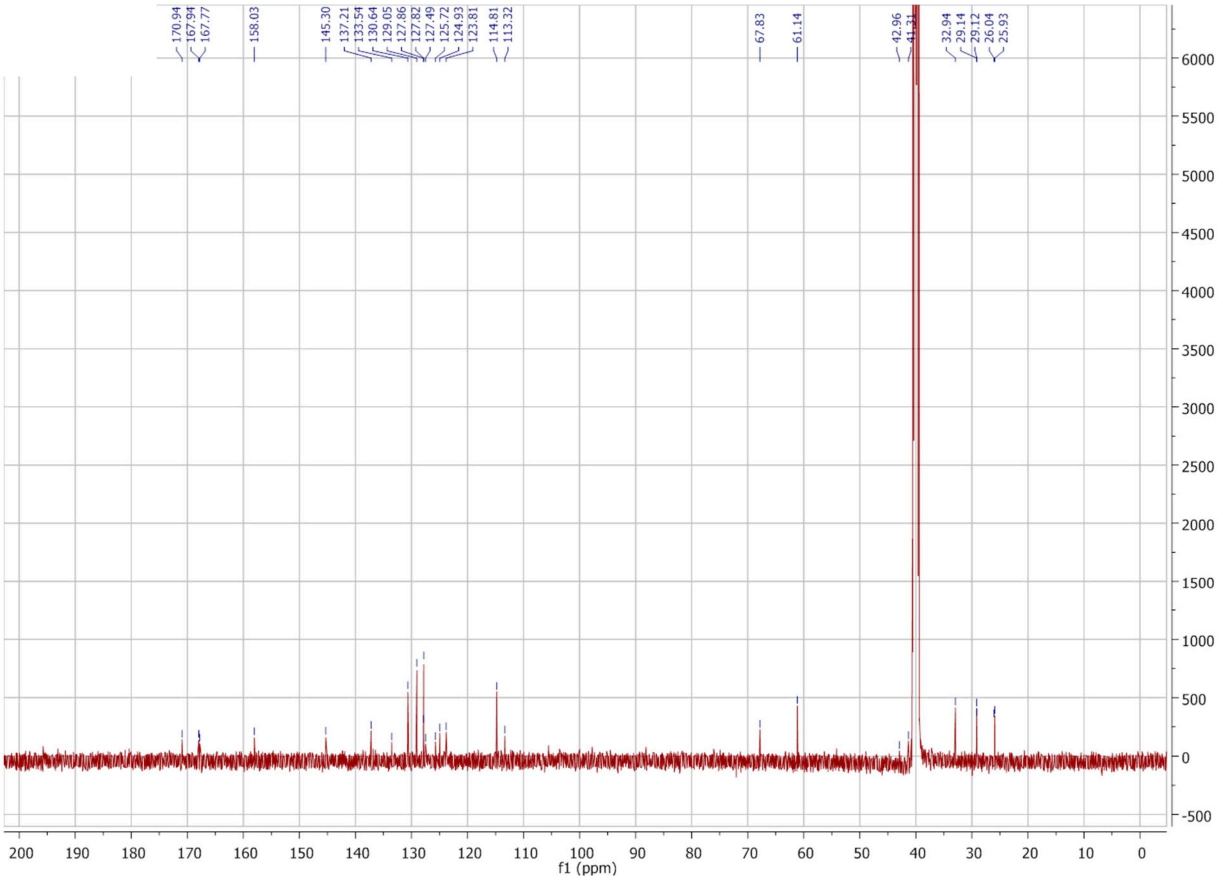

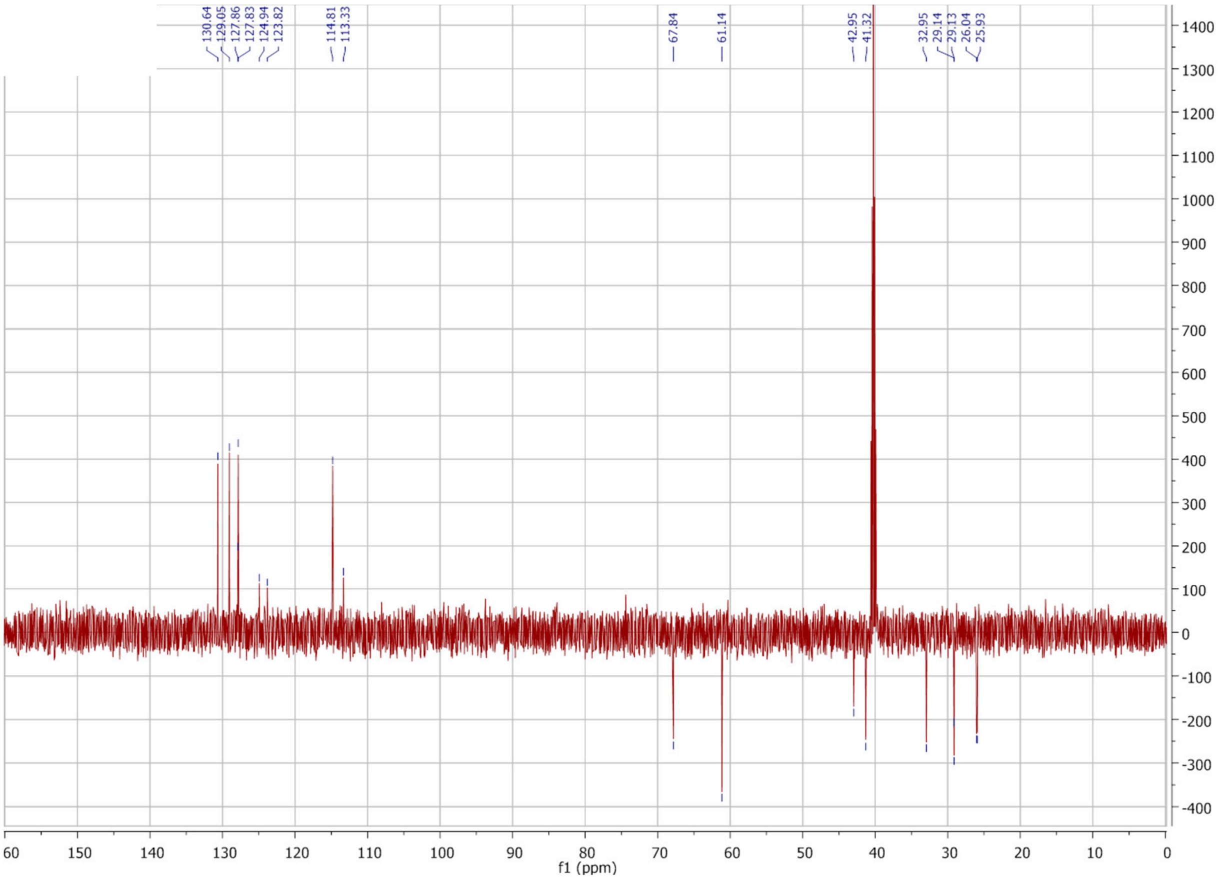

N-(2-benzyl-1,3-dioxo-2,3-dihydro-1H-isoindol-5-yl)-2-{4-[(8-hydroxyoctyl)oxy]phenyl}acetamide (**13h**: ZHAWOC7137)

<u>NMR</u>

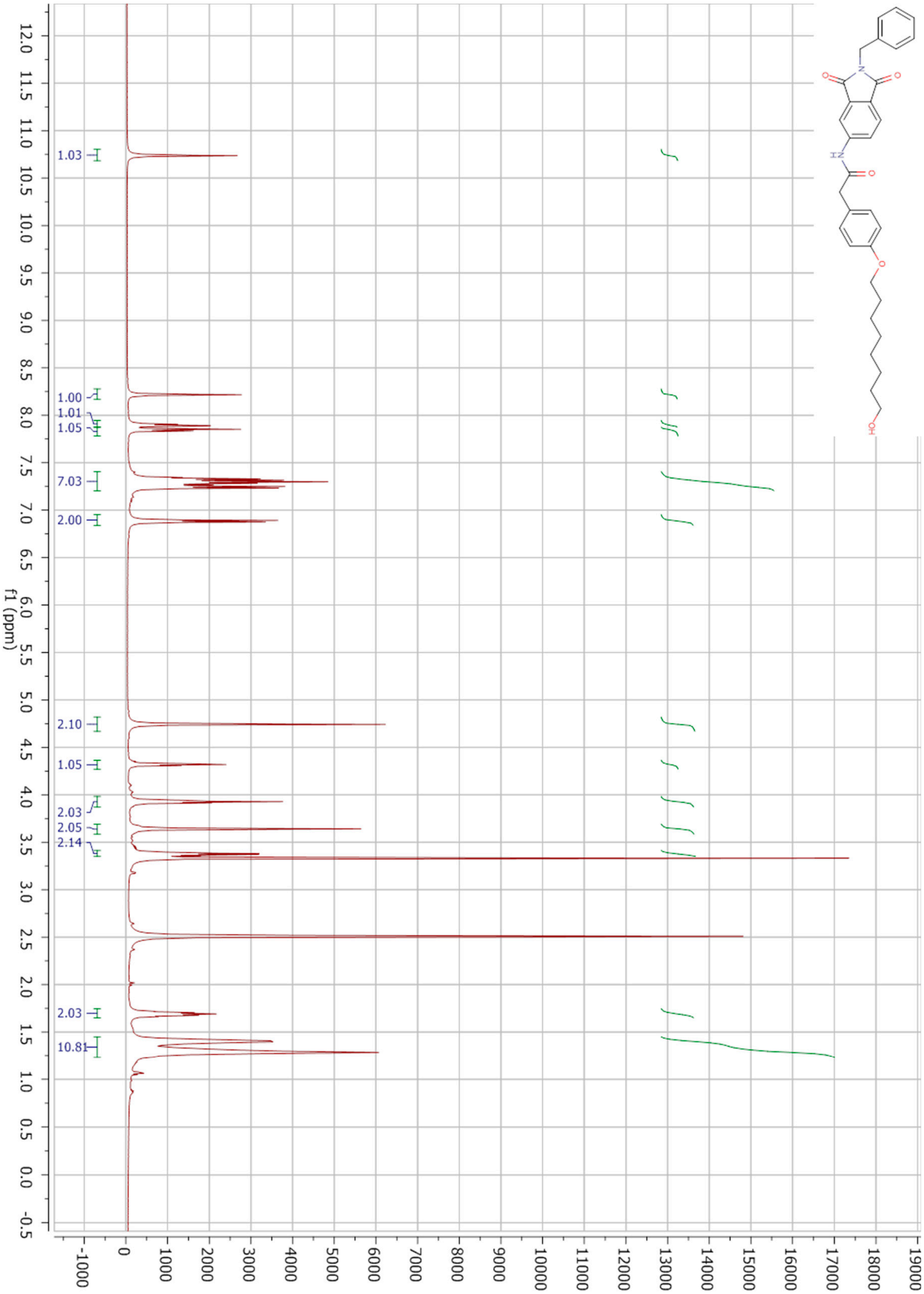

N-(2-benzyl-1,3-dioxo-2,3-dihydro-1H-isoindol-5-yl)-2-{4-[(8-hydroxyoctyl)oxy]phenyl}acetamide (**13h**: ZHAWOC7137)

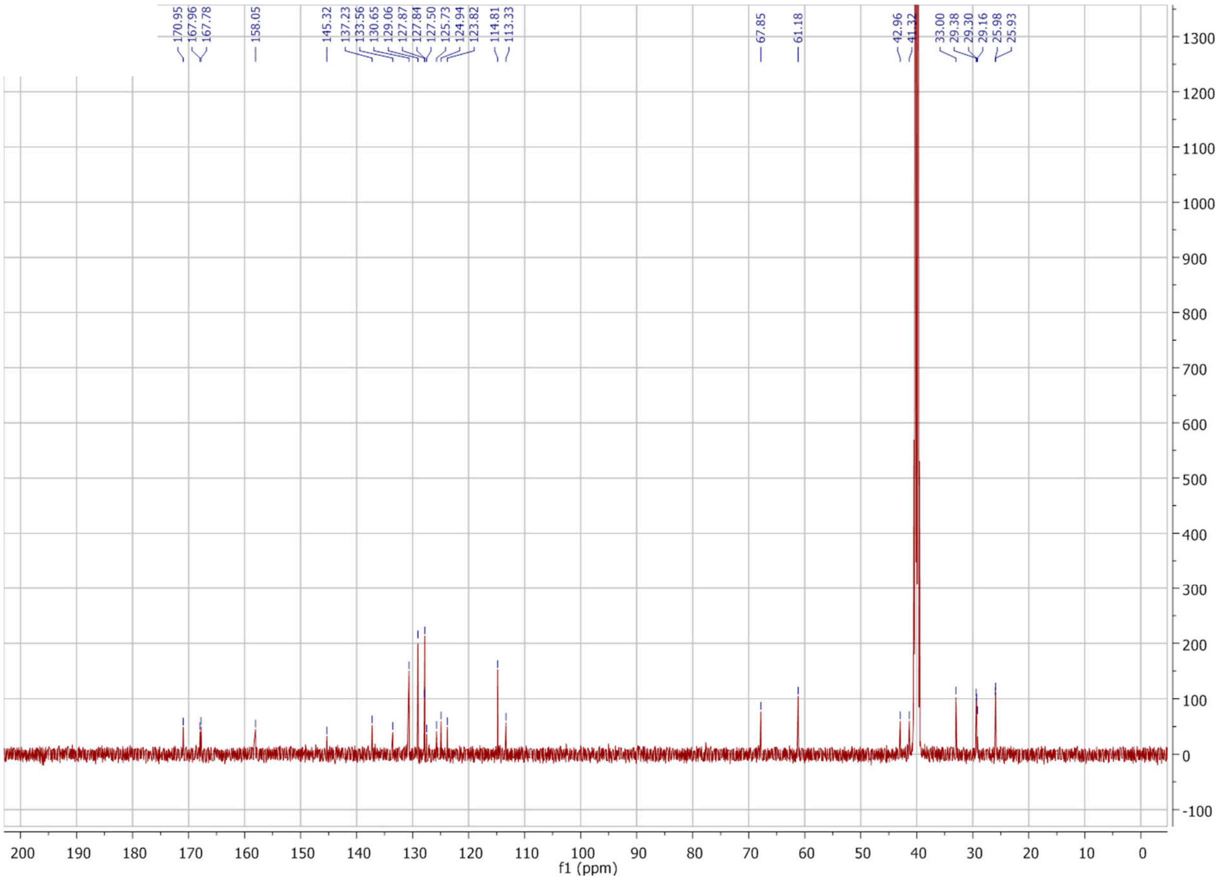

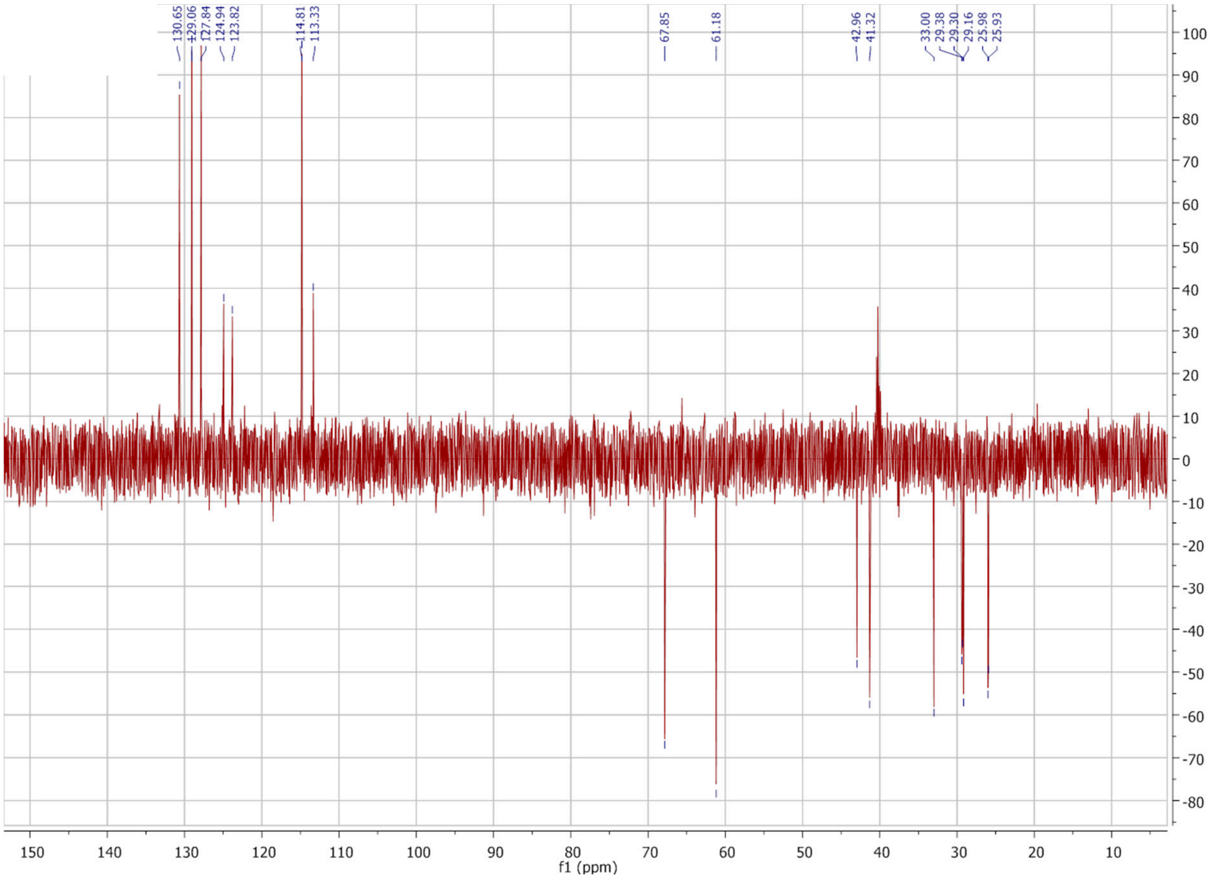

N-(2-benzyl-1,3-dioxo-2,3-dihydro-1H-isoindol-5-yl)-2-{4-[(9-hydroxynonyl)oxy]phenyl}acetamide (**13i**; ZHAWOC6936)

<u>NMR</u>

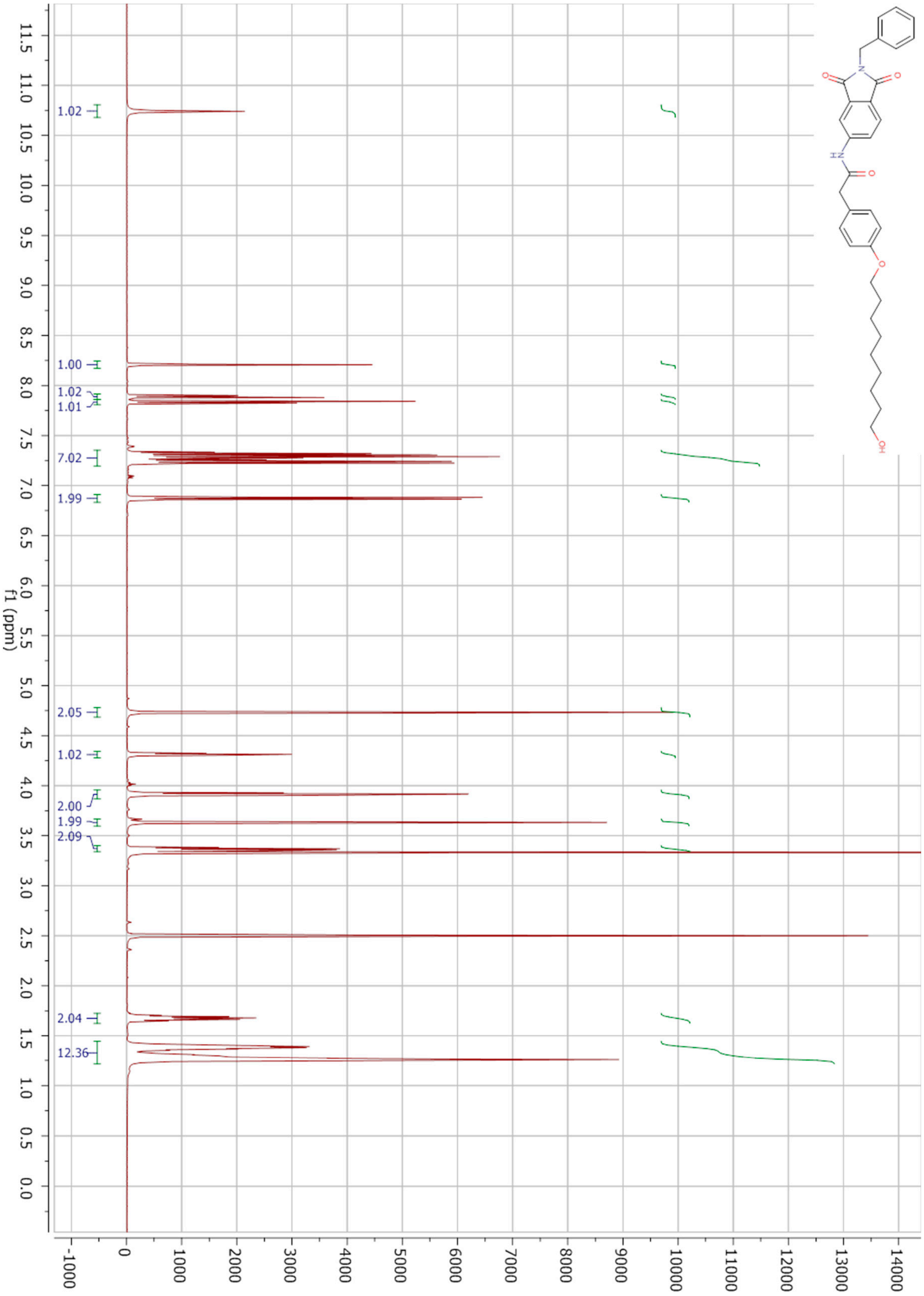

N-(2-benzyl-1,3-dioxo-2,3-dihydro-1H-isoindol-5-yl)-2-{4-[(9-hydroxynonyl)oxy]phenyl}acetamide (**13i**; ZHAWOC6936)

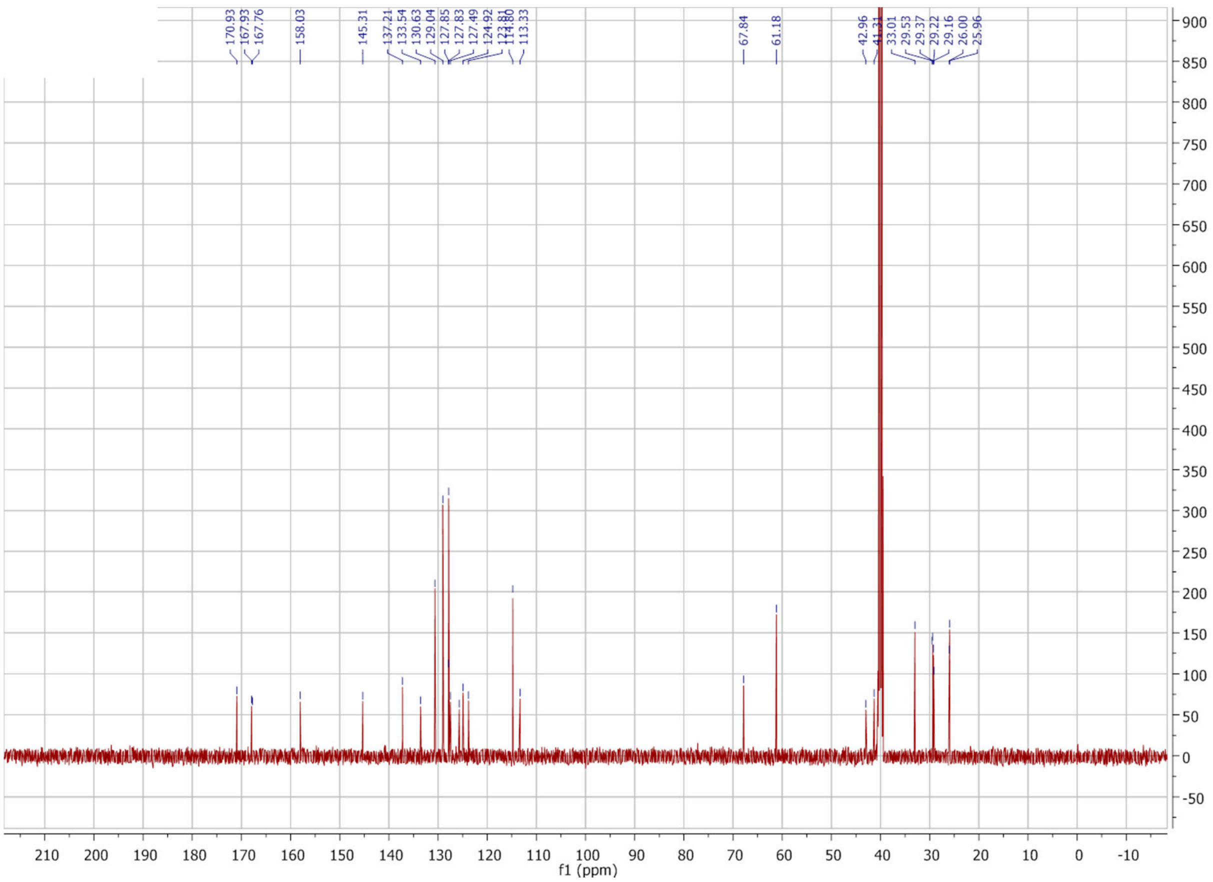

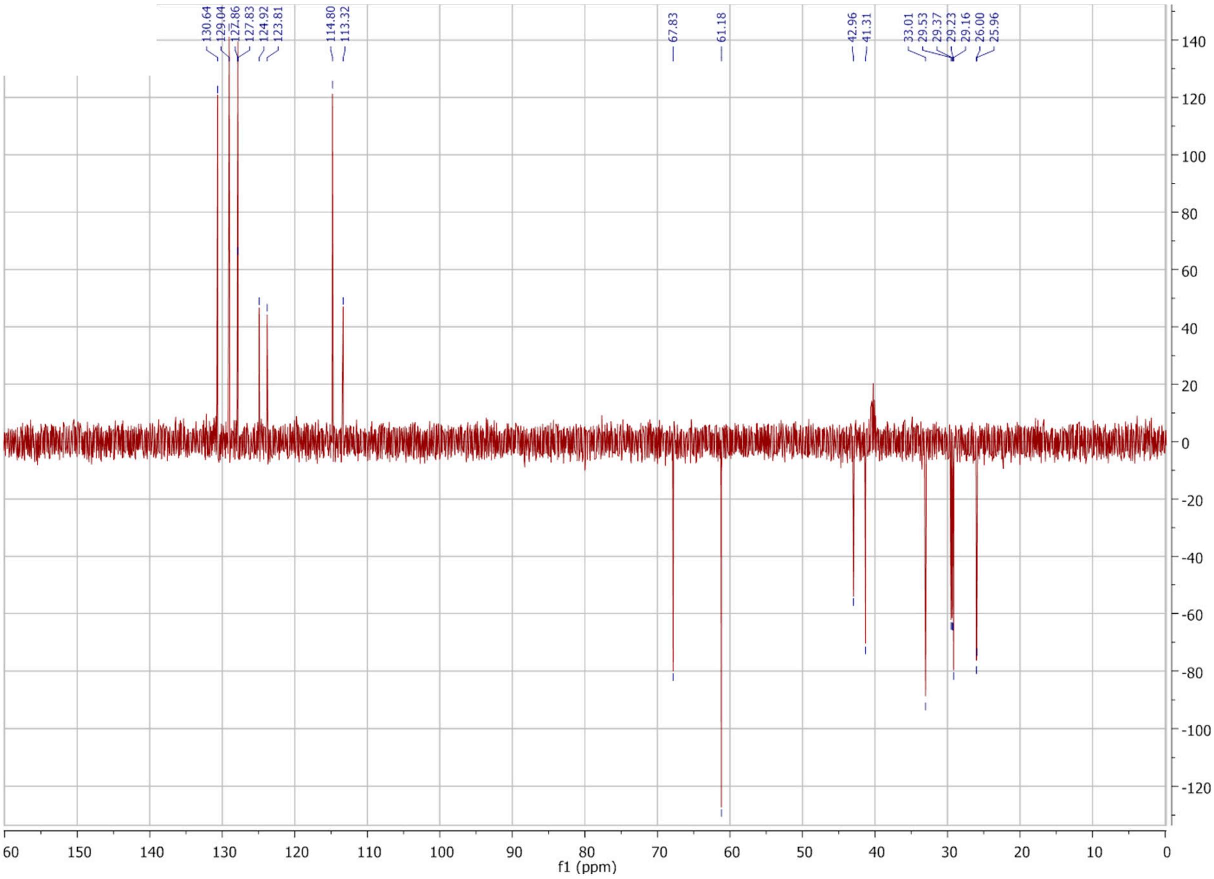

N-(2-benzyl-1,3-dioxo-2,3-dihydro-1H-isoindol-5-yl)-2-{4-[(10-hydroxydecyl)oxy]phenyl}acetamide (**13j**; ZHAWOC6937)

<u>NMR</u>

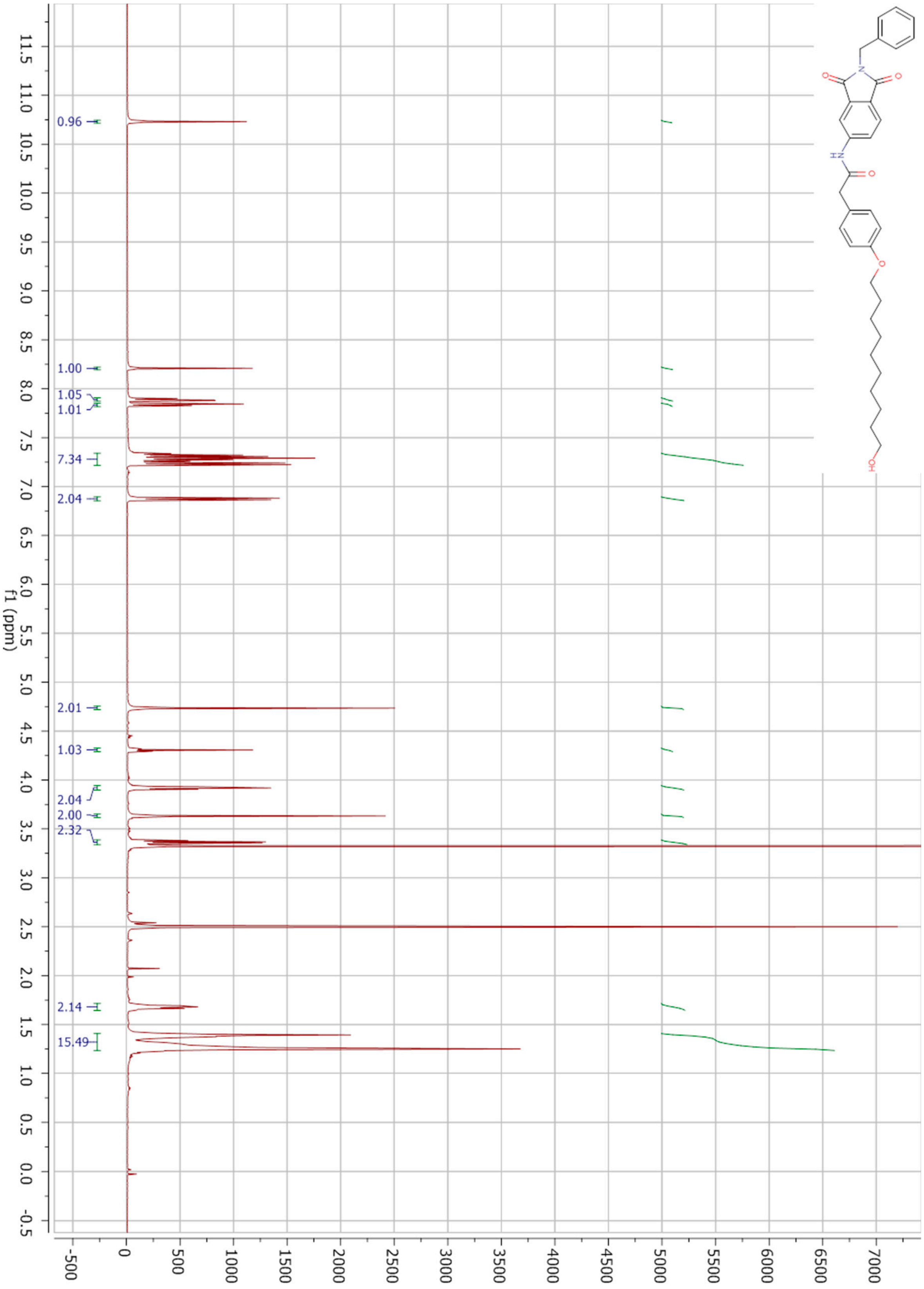

N-(2-benzyl-1,3-dioxo-2,3-dihydro-1H-isoindol-5-yl)-2-{4-[(10-hydroxydecyl)oxy]phenyl}acetamide (**13j**; ZHAWOC6937)

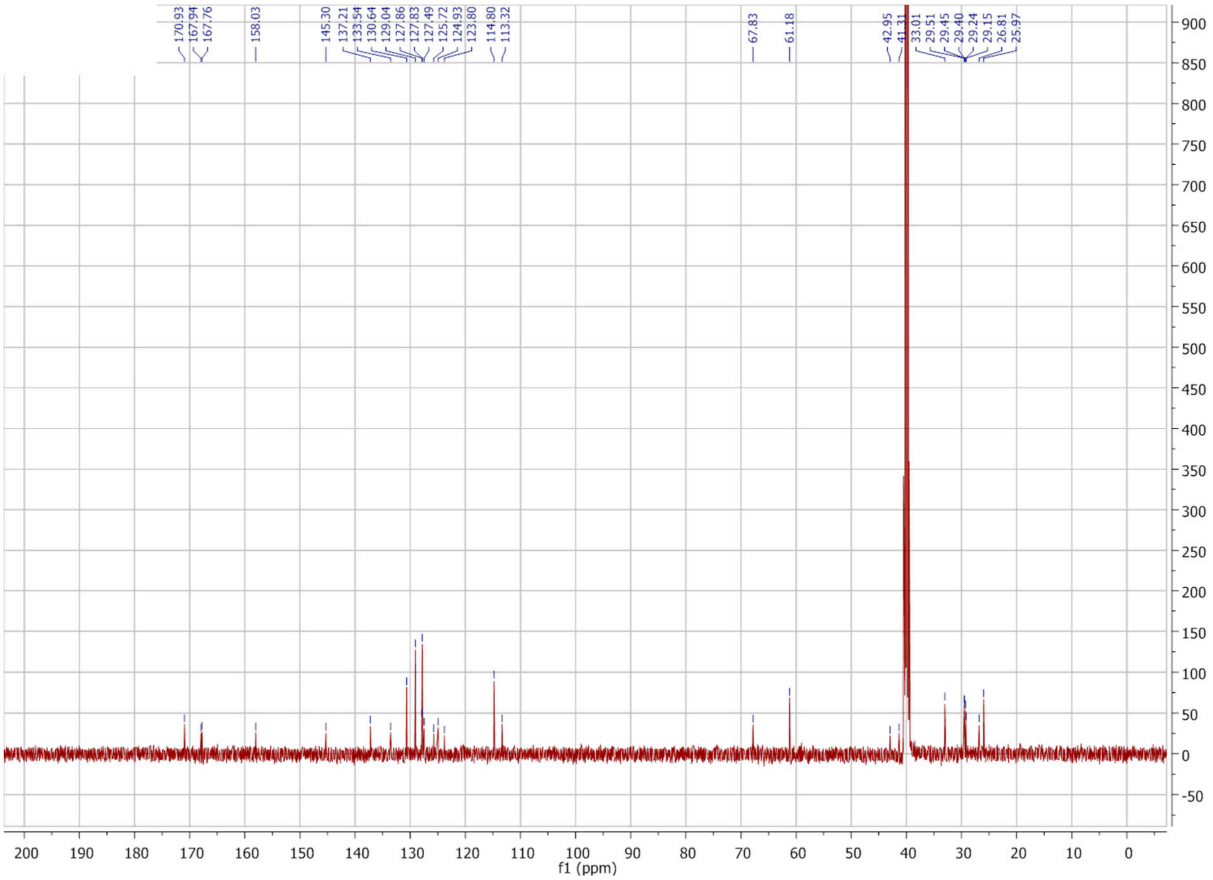

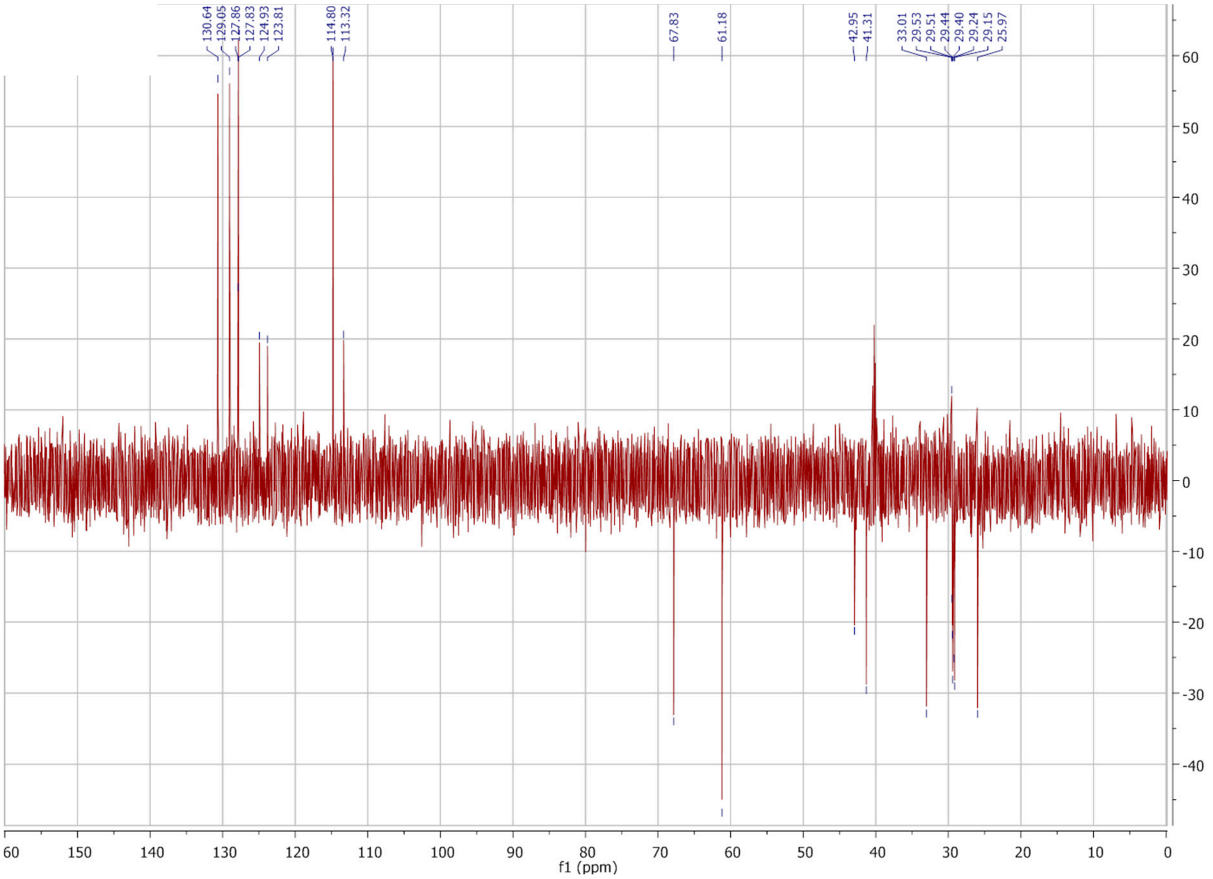

N-{2-[(4-fluorophenyl)methyl]-1,3-dioxo-2,3-dihydro-1H-isoindol-5-yl}-2-[4-(3-hydroxypropoxy)phenyl]acetamide (**13k**; ZHAWOC6642)

<u>NMR</u>

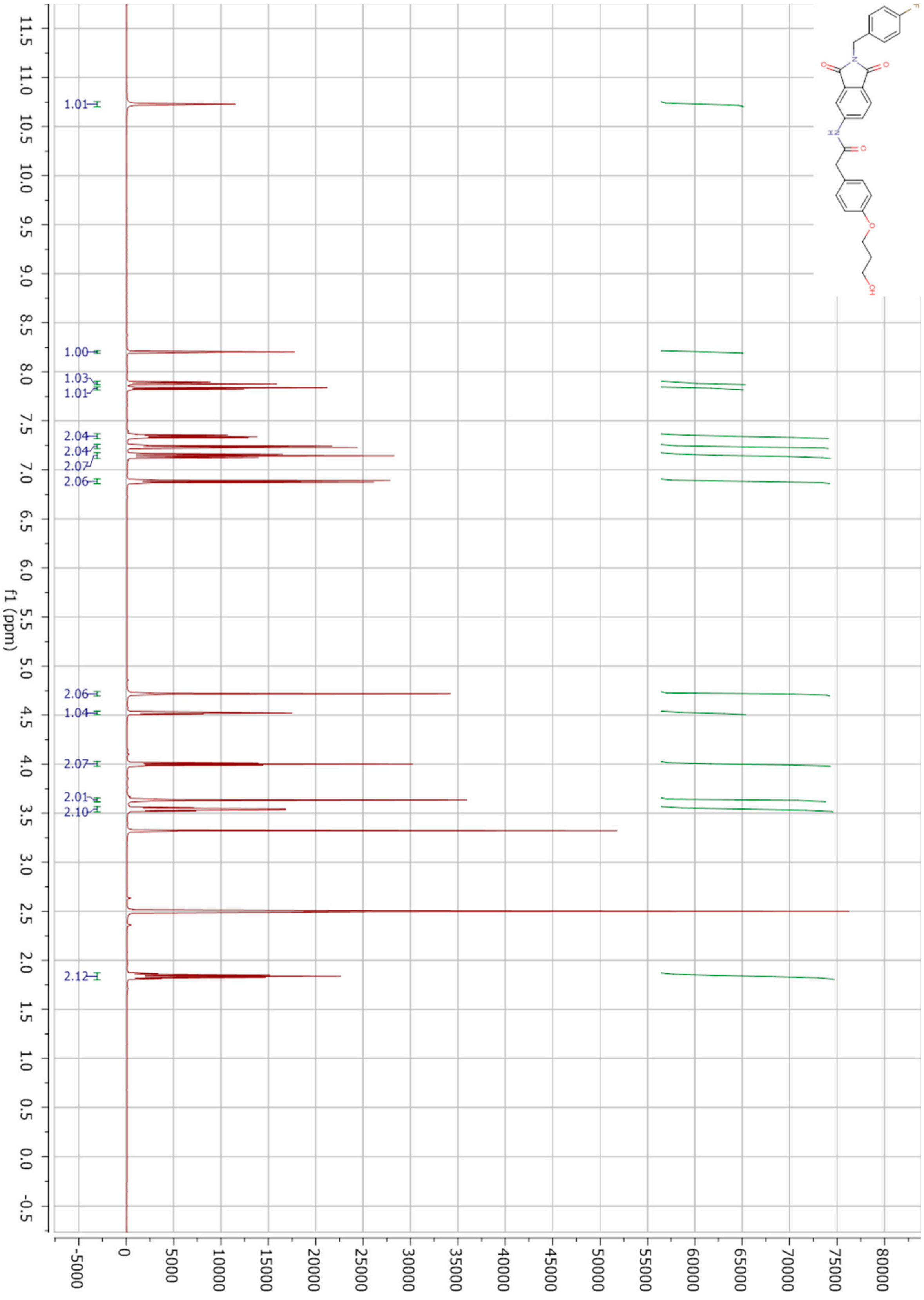

N-{2-[(4-fluorophenyl)methyl]-1,3-dioxo-2,3-dihydro-1H-isoindol-5-yl}-2-[4-(3-hydroxypropoxy)phenyl]acetamide (**13k**; ZHAWOC6642)

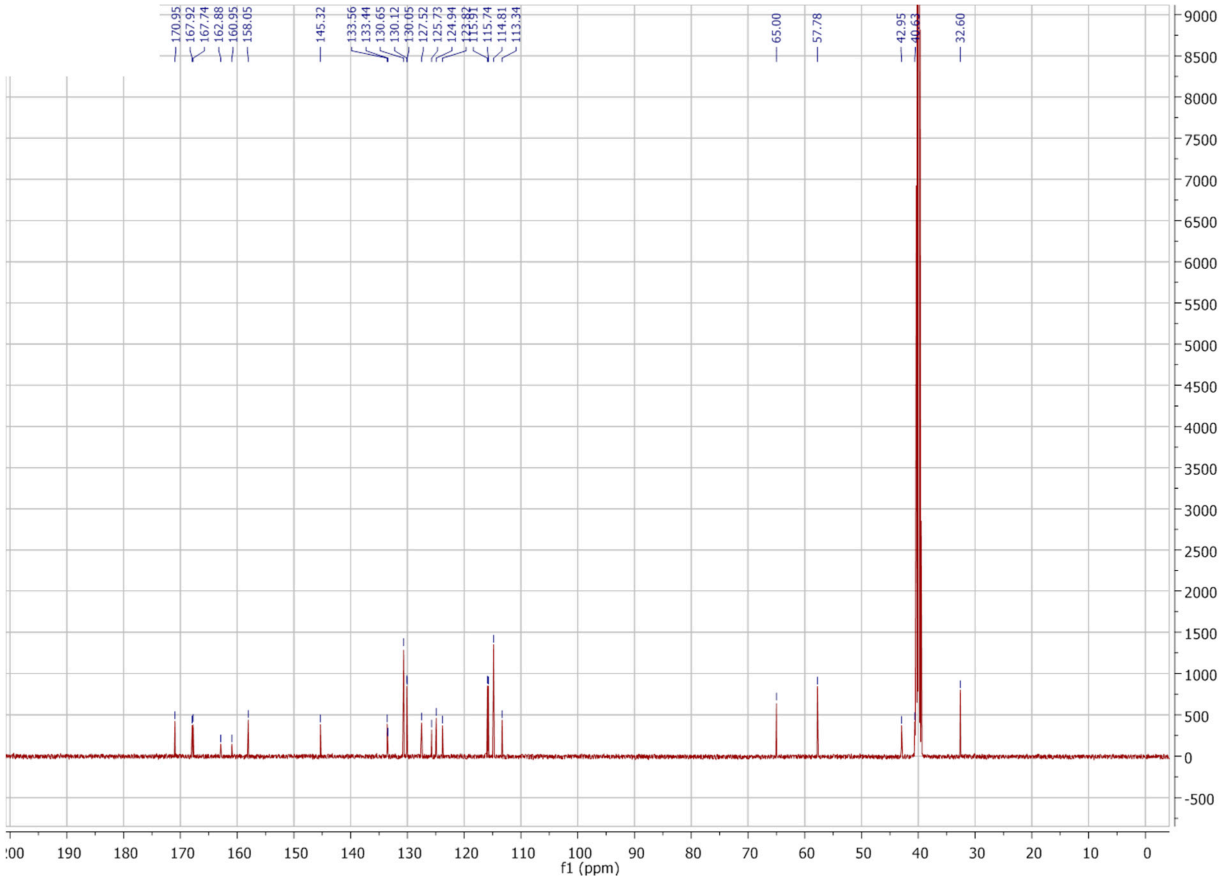

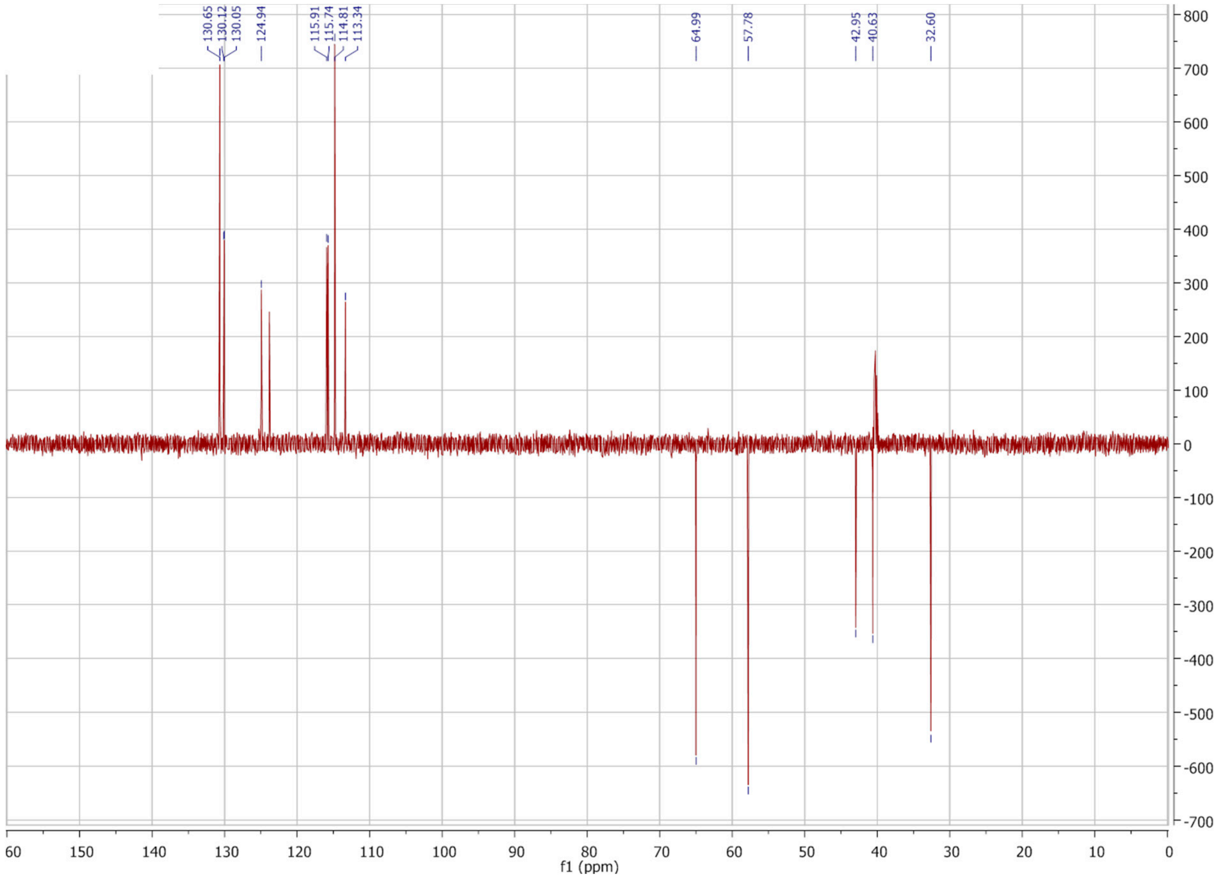

N-{2-[(4-fluorophenyl)methyl]-1,3-dioxo-2,3-dihydro-1*H*-isoindol-5-yl}-2-[4-(4-hydroxybutoxy)phenyl]acetamide (**13l**; ZHAWOC5462)

NMR

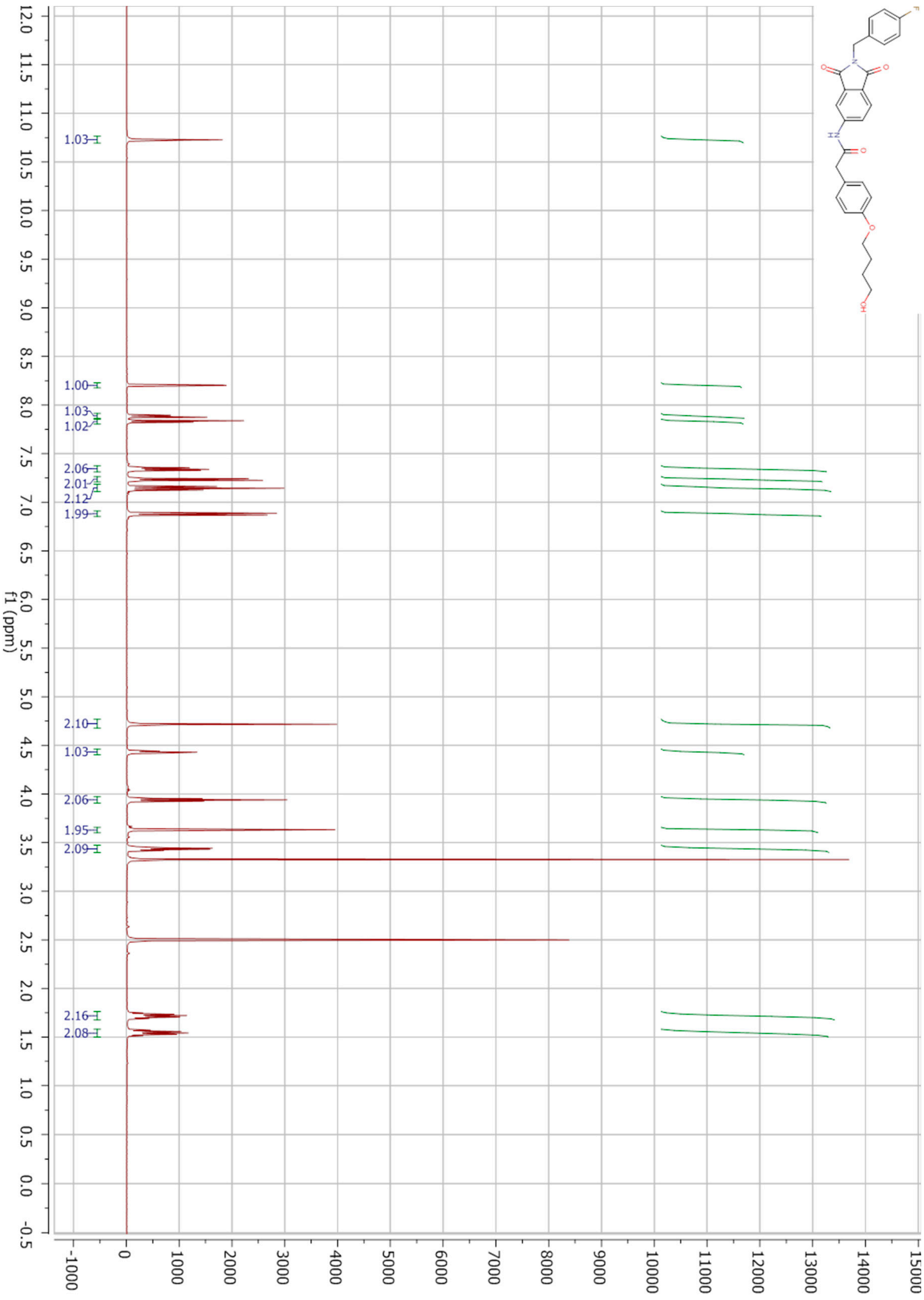

N-{2-[(4-fluorophenyl)methyl]-1,3-dioxo-2,3-dihydro-1H-isoindol-5-yl}-2-[4-(4-hydroxybutoxy)phenyl]acetamide (**13l**; ZHAWOC5462)

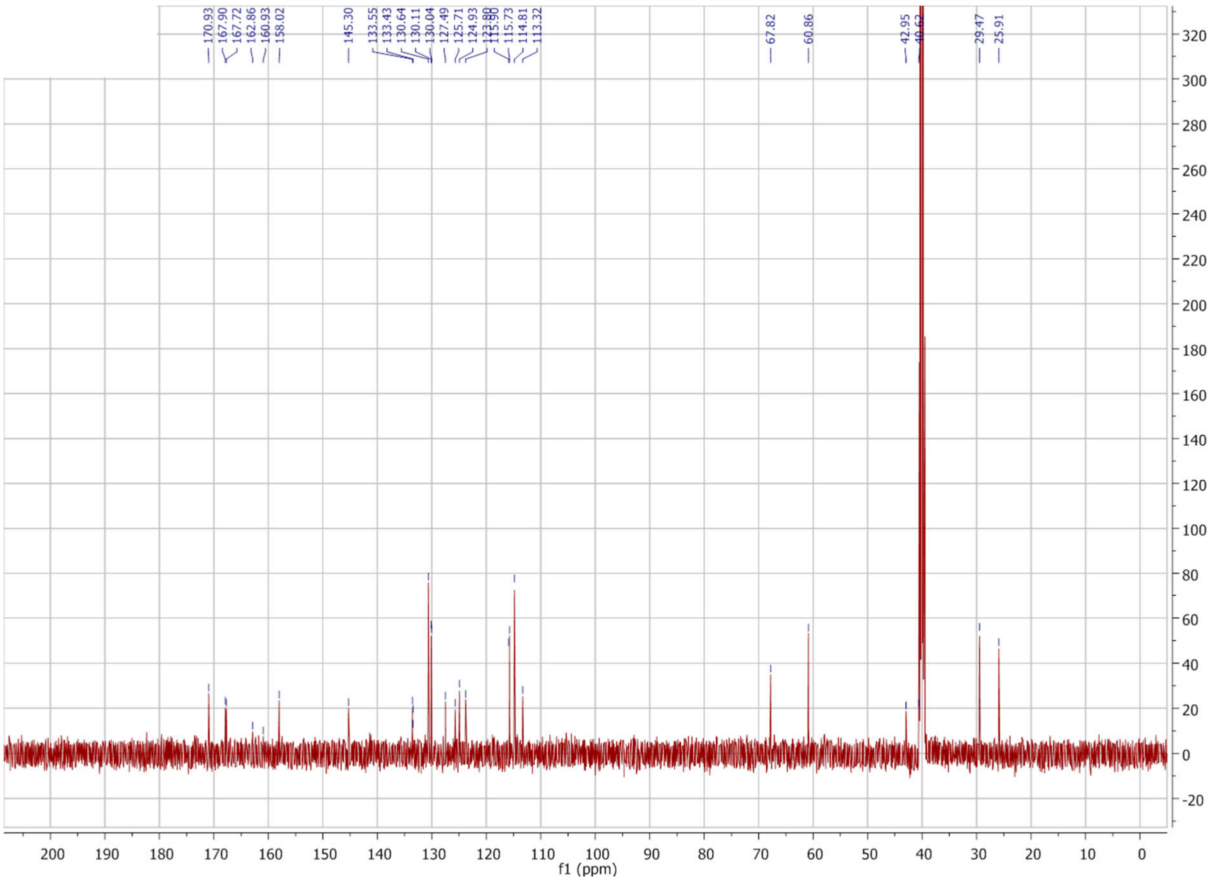

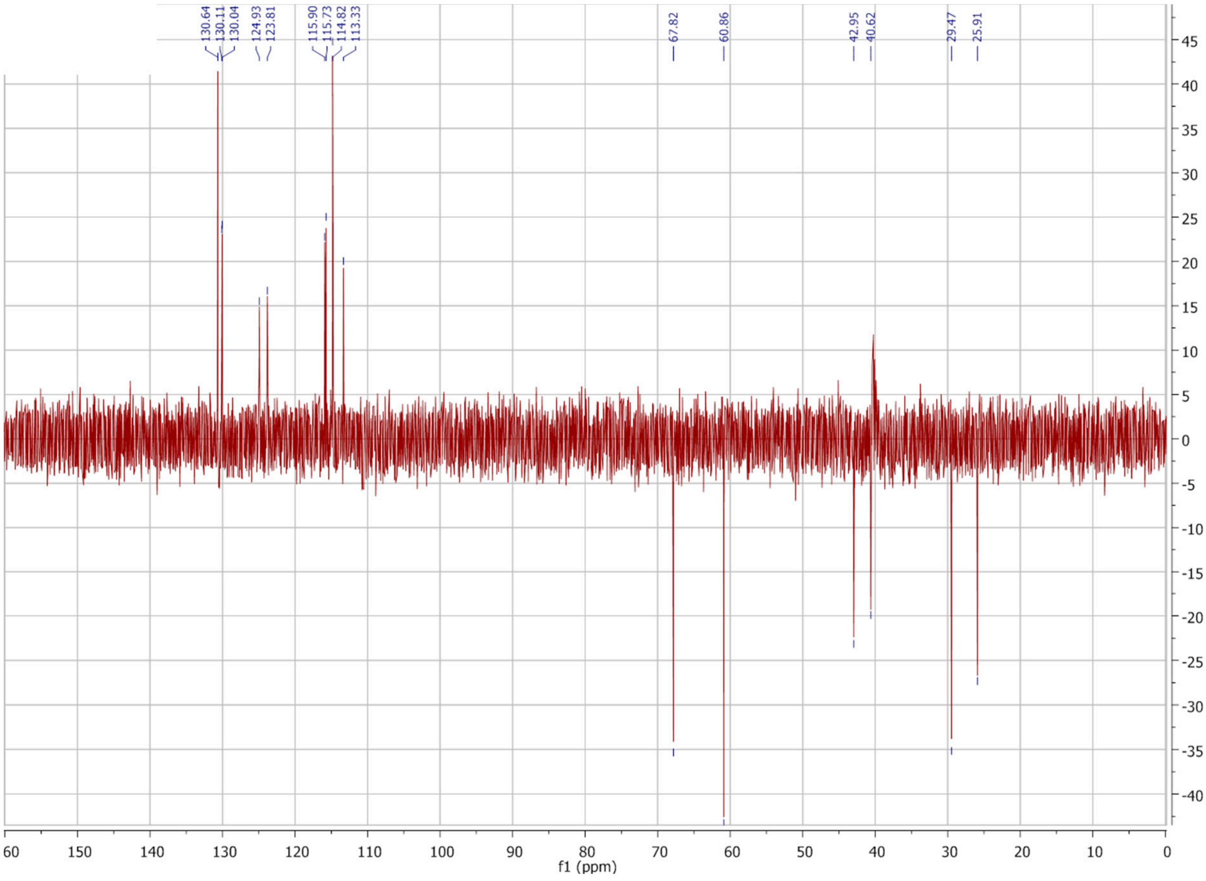

N-{2-[(4-fluorophenyl)methyl]-1,3-dioxo-2,3-dihydro-1H-isoindol-5-yl}-2-{4-[(5-hydroxypentyl)oxy]phenyl}acetamide (**13m;** ZHAWOC5683)

<u>NMR</u>

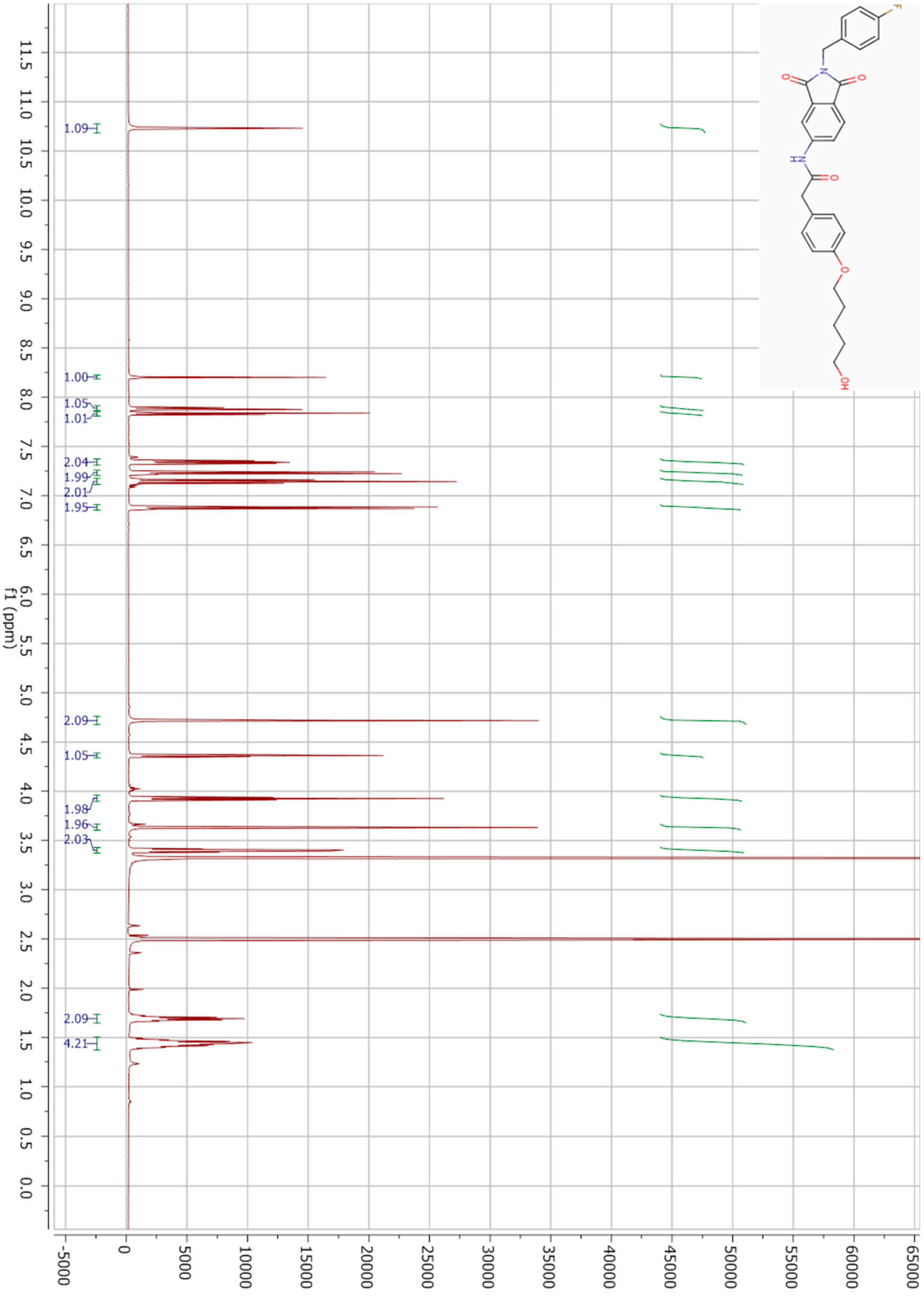

N-{2-[(4-fluorophenyl)methyl]-1,3-dioxo-2,3-dihydro-1H-isoindol-5-yl}-2-{4-[(5-hydroxypentyl)oxy]phenyl}acetamide (**13m;** ZHAWOC5683)

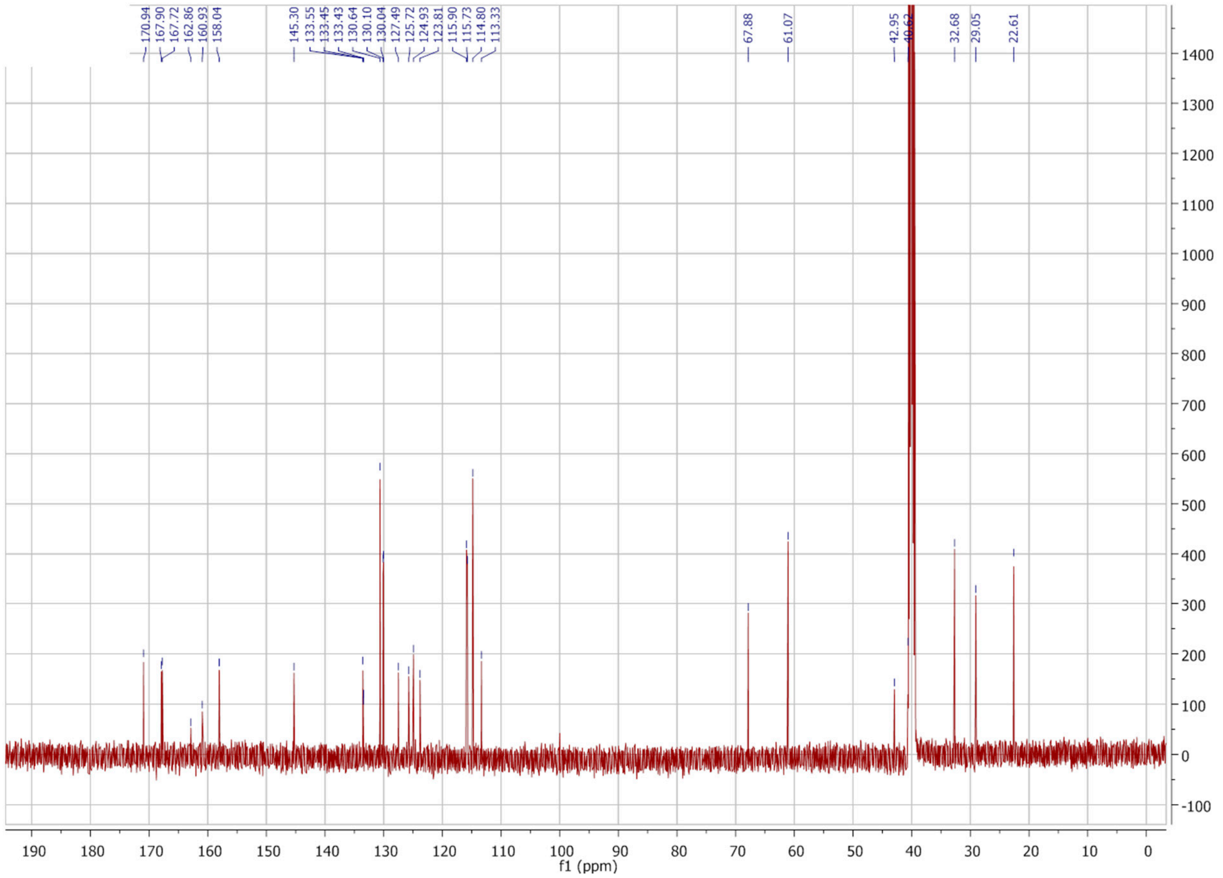

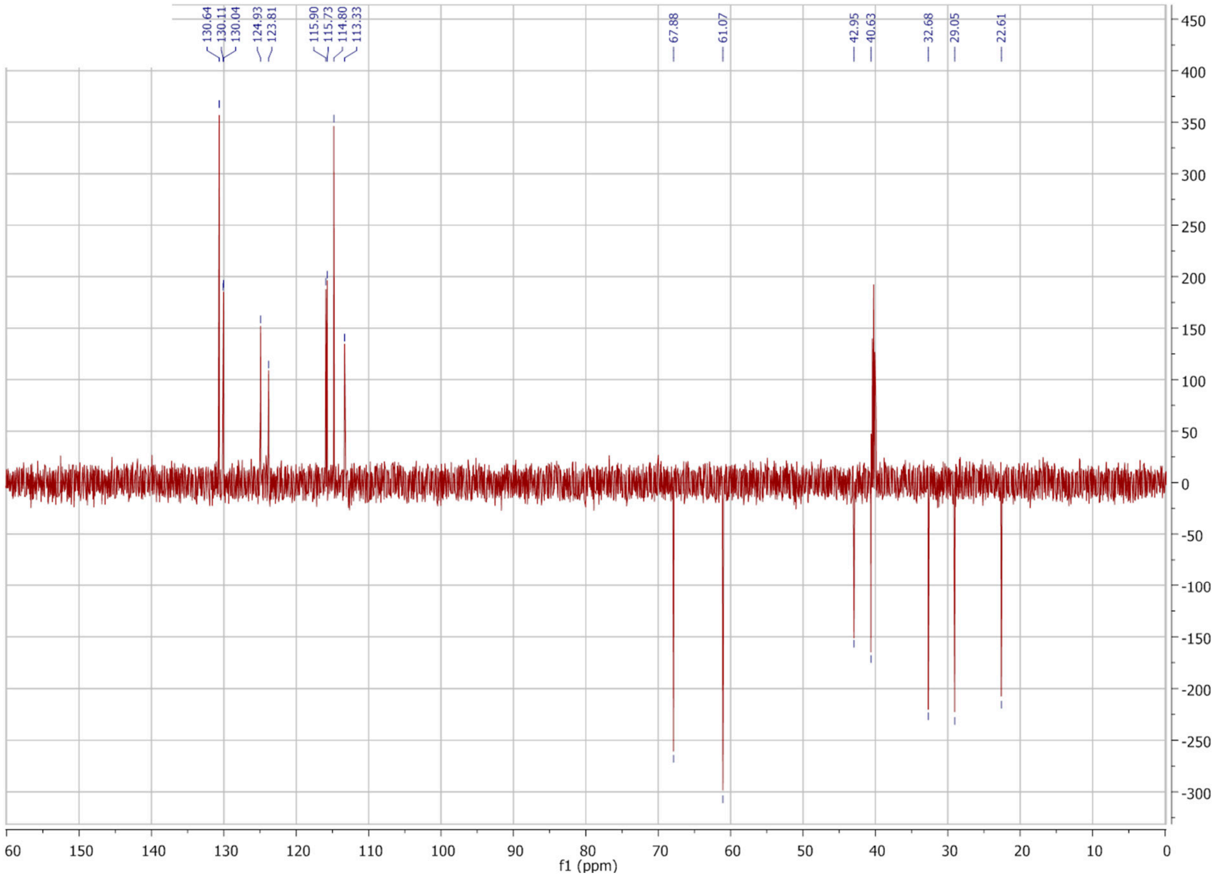

N-{2-[(4-fluorophenyl)methyl]-1,3-dioxo-2,3-dihydro-1*H*-isoindol-5-yl}-2-{4-[(6-hydroxyhexyl)oxy]phenyl}acetamide (**13n**; ZHAWOC6643)

<u>NMR</u>

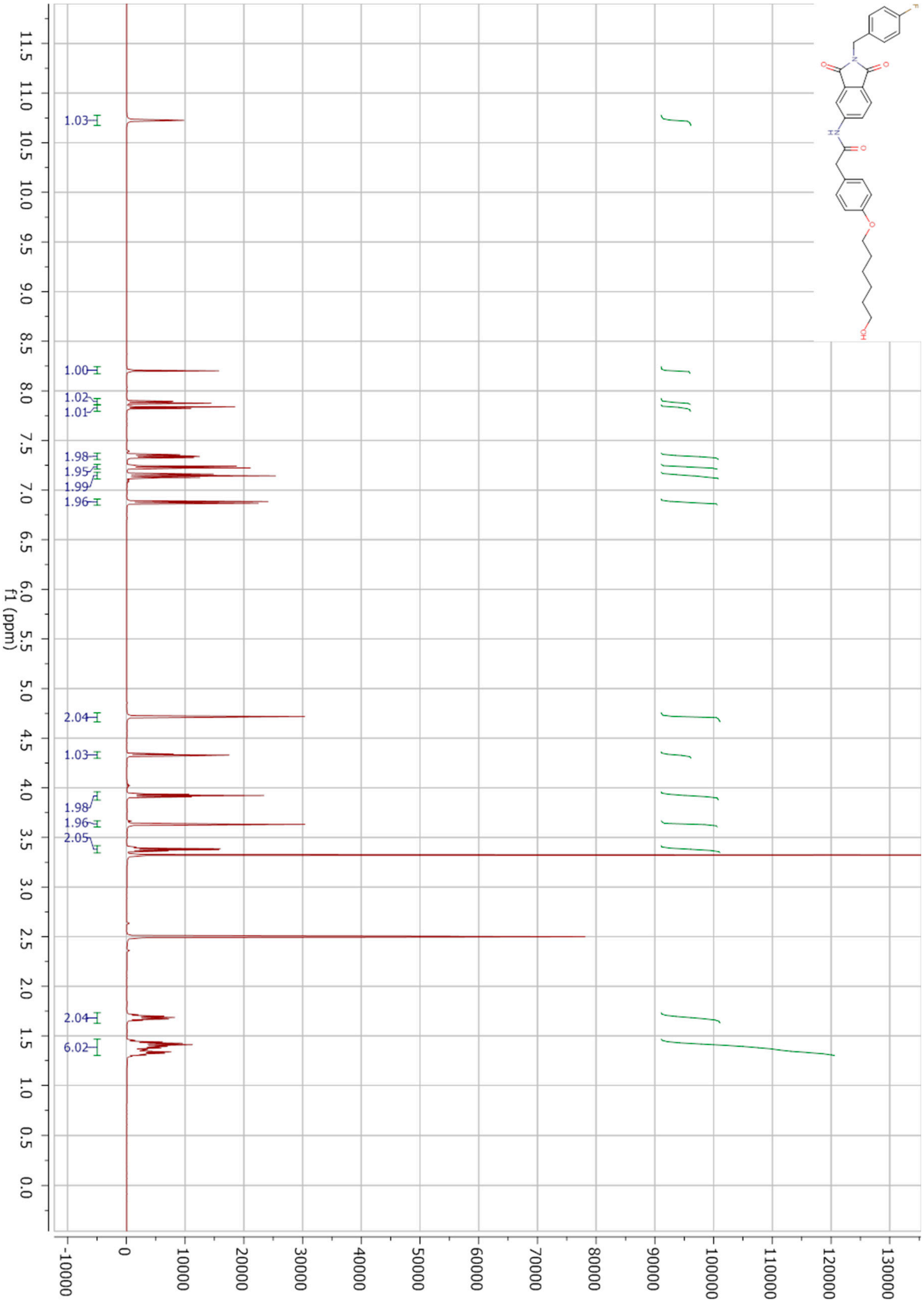

N-{2-[(4-fluorophenyl)methyl]-1,3-dioxo-2,3-dihydro-1H-isoindol-5-yl}-2-{4-[(6-hydroxyhexyl)oxy]phenyl}acetamide (**13n**; ZHAWOC6643)

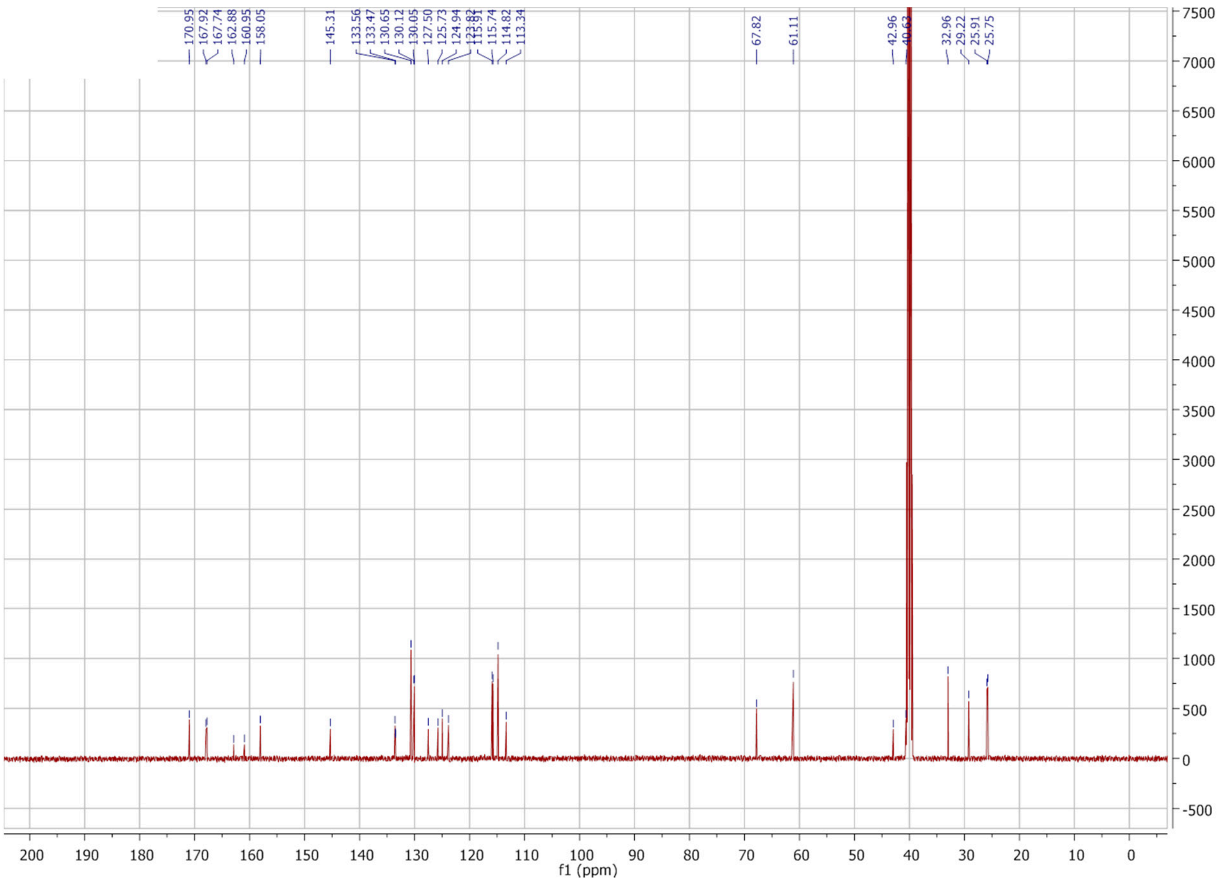

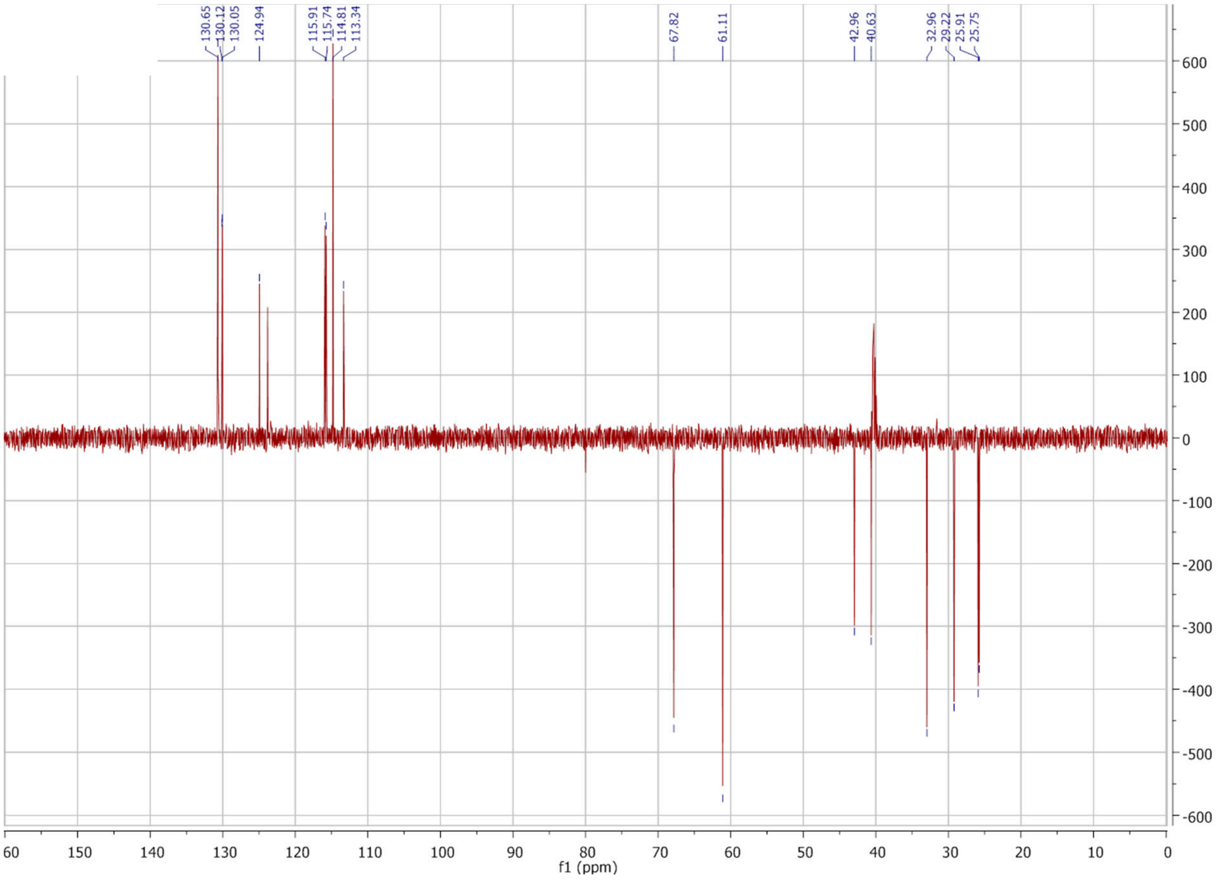

N-{2-[(4-fluorophenyl)methyl]-1,3-dioxo-2,3-dihydro-1H-isoindol-5-yl}-2-{4-[(7-hydroxyheptyl)oxy]phenyl}acetamide (**13o**; ZHAWOC6636)

<u>NMR</u>

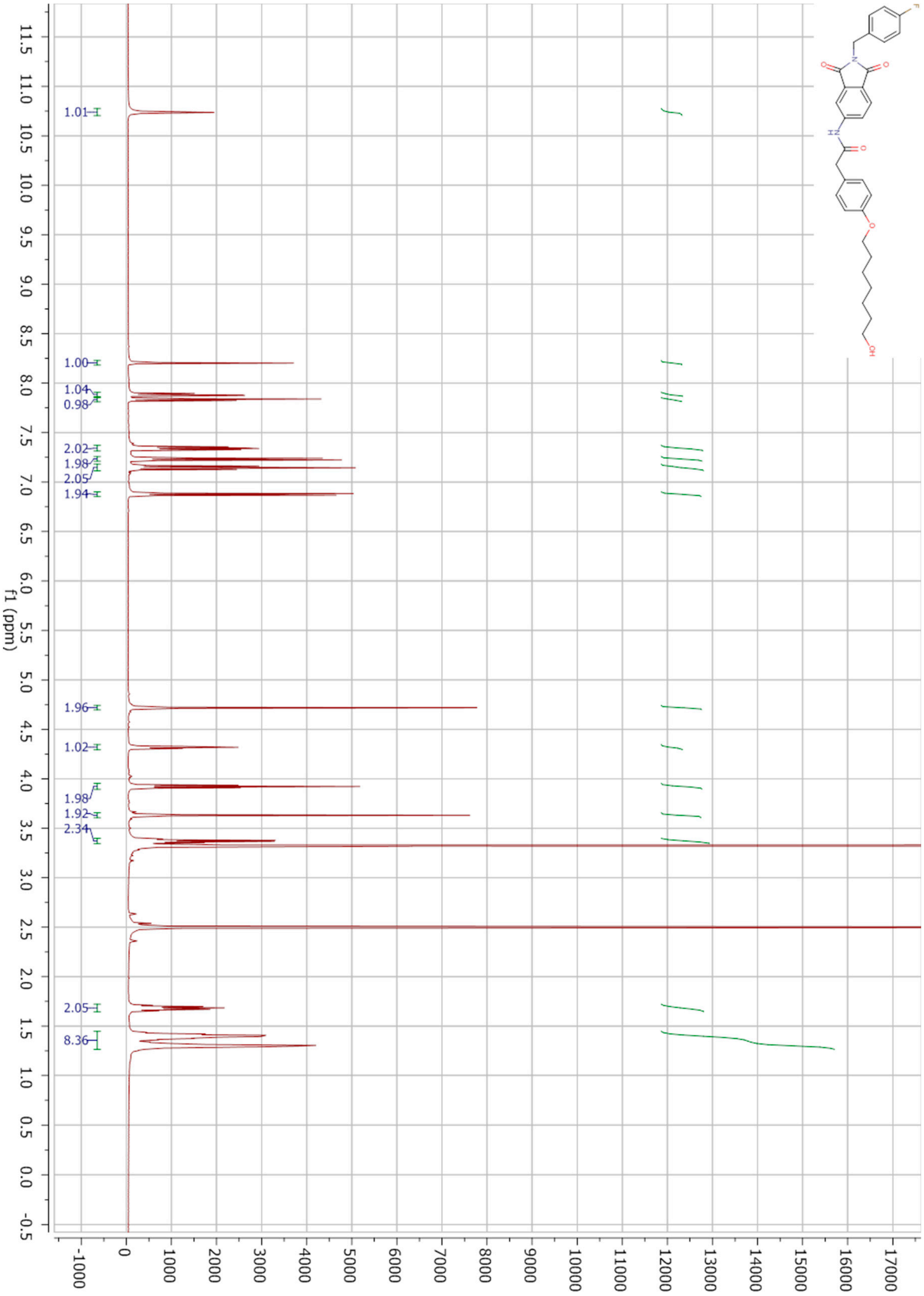

N-{2-[(4-fluorophenyl)methyl]-1,3-dioxo-2,3-dihydro-1*H*-isoindol-5-yl}-2-{4-[(7-hydroxyheptyl)oxy]phenyl}acetamide (**13o**; ZHAWOC6636)

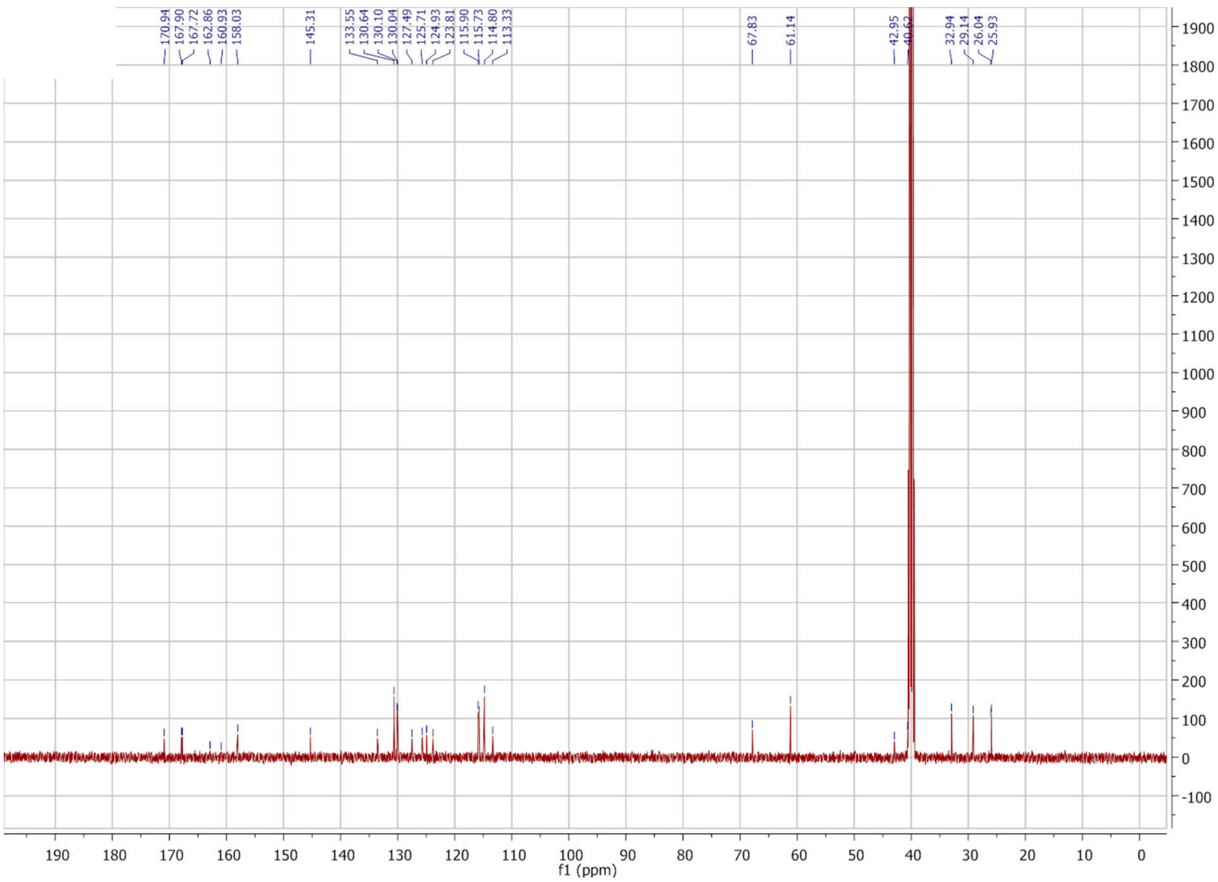

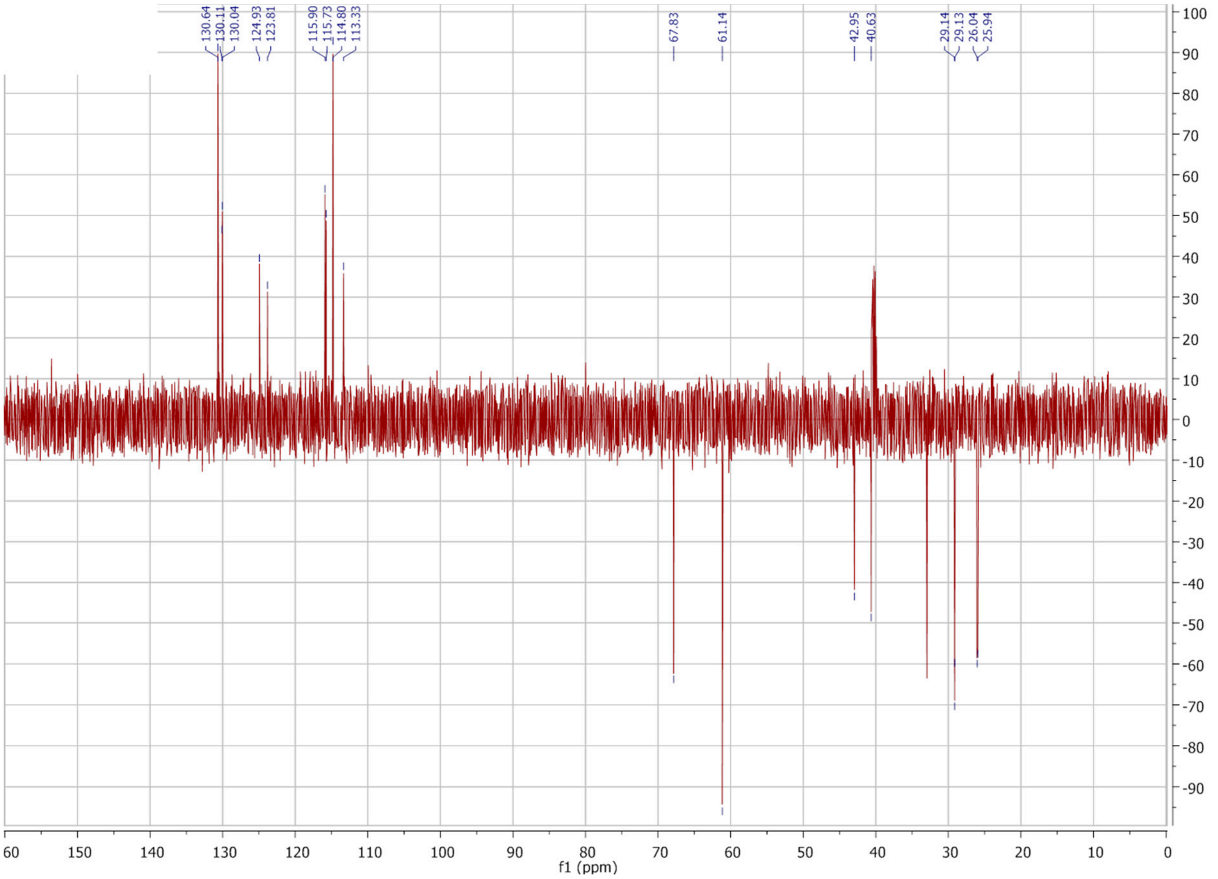

N-{2-[(4-fluorophenyl)methyl]-1,3-dioxo-2,3-dihydro-1H-isoindol-5-yl}-2-{4-[(8-hydroxyoctyl)oxy]phenyl}acetamide (**13p**; ZHAWOC6639)

<u>NMR</u>

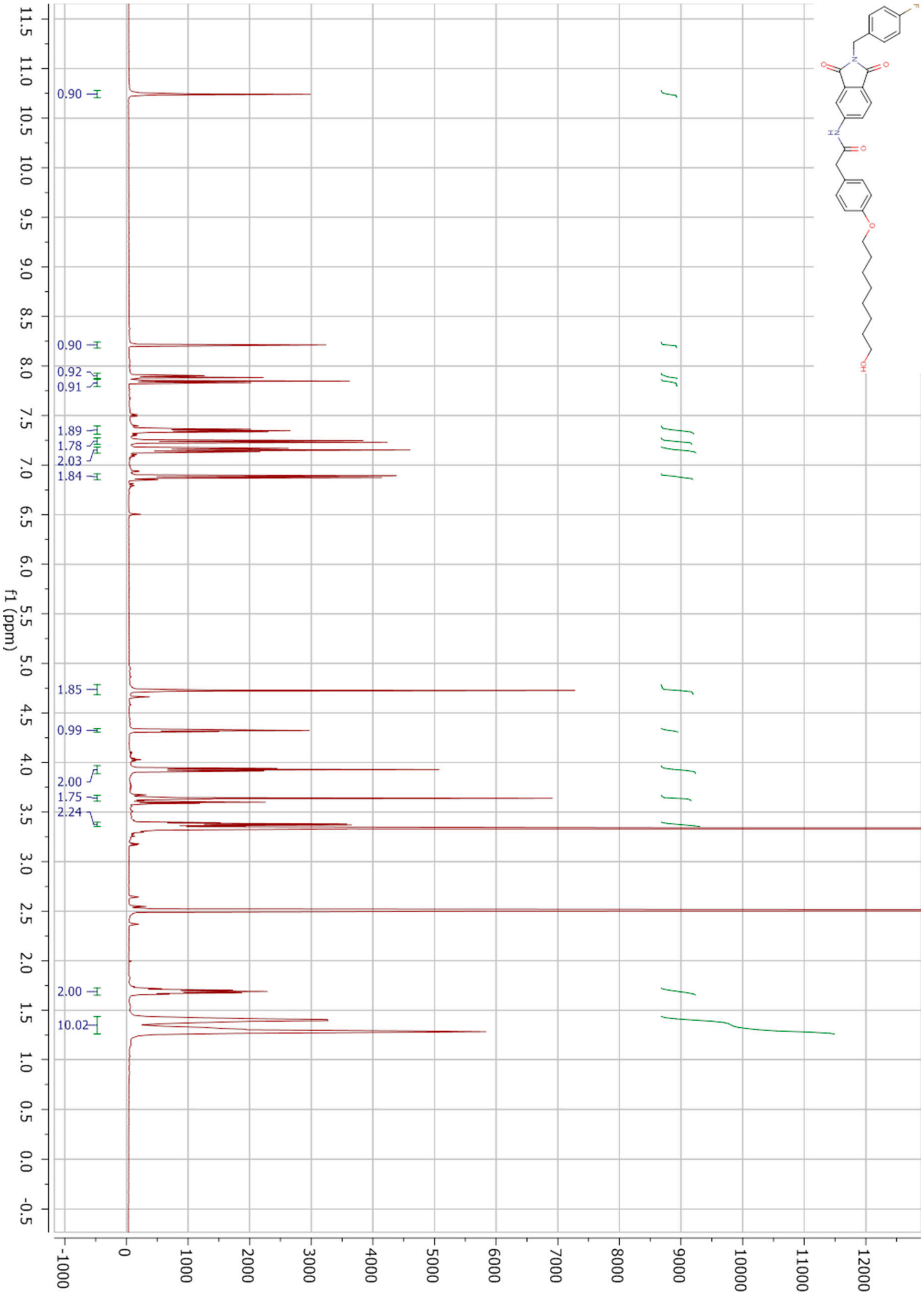

N-{2-[(4-fluorophenyl)methyl]-1,3-dioxo-2,3-dihydro-1H-isoindol-5-yl}-2-{4-[(8-hydroxyoctyl)oxy]phenyl}acetamide (**13p**; ZHAWOC6639)

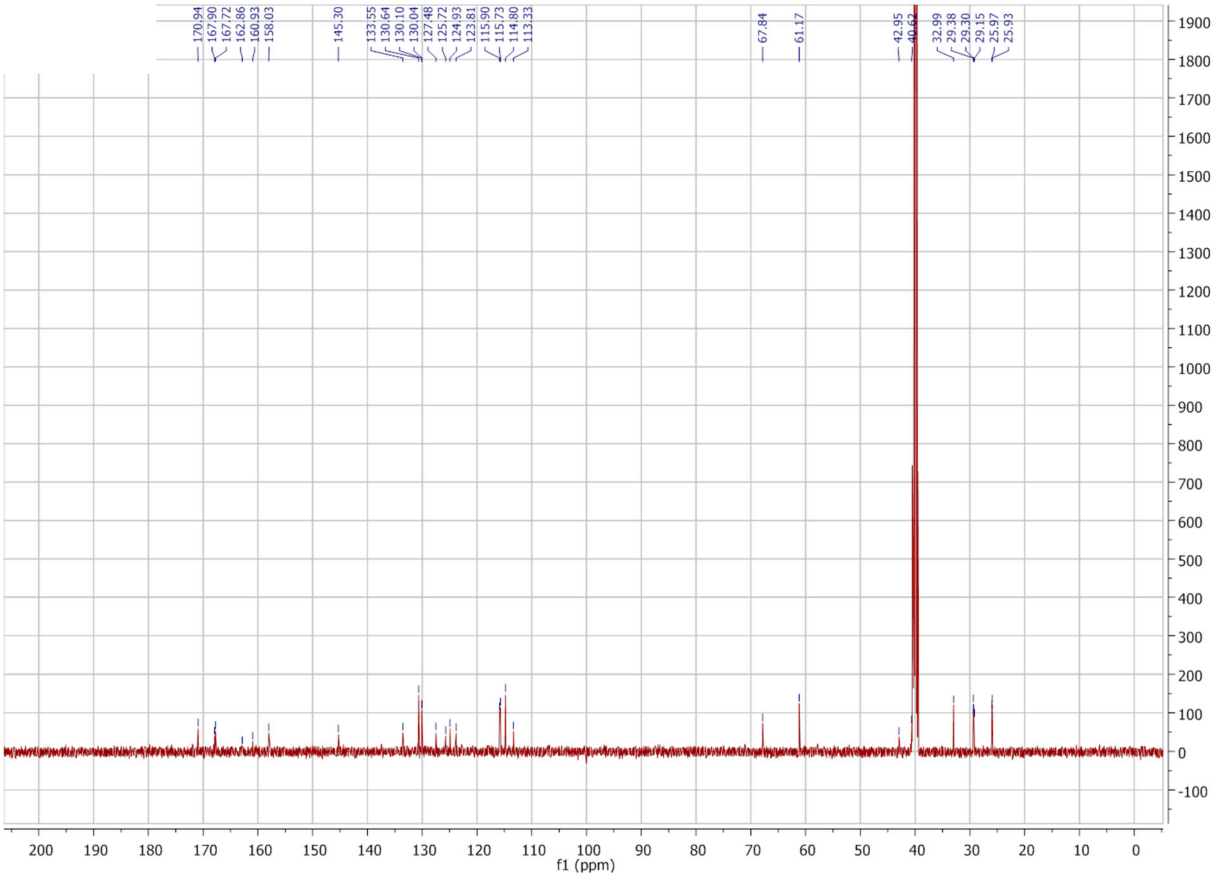

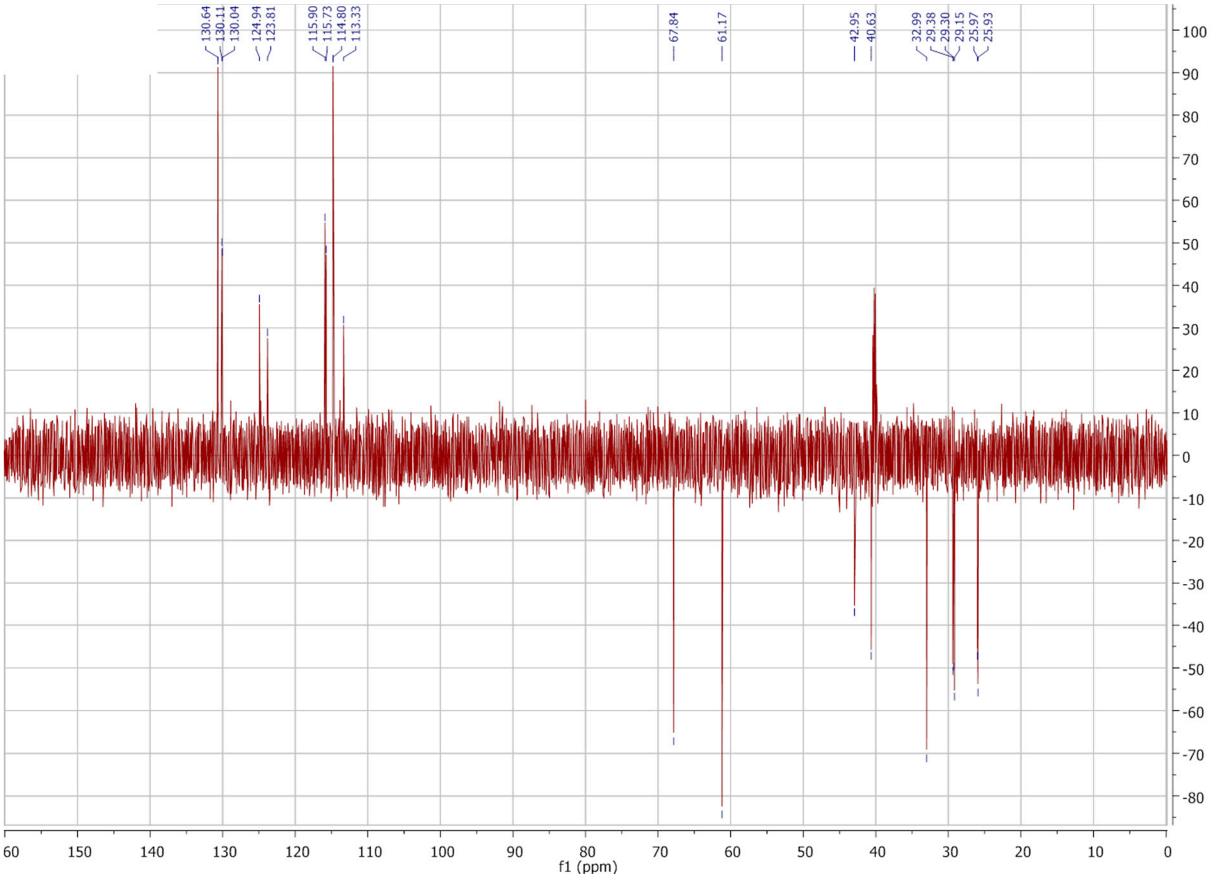

5-(4-{[(2-methyl-1,3-dioxo-2,3-dihydro-1H-isoindol-5-yl)carbamoyl]methyl}phenoxy)pentanoic acid (**14a**; ZHAWOC6649)

<u>NMR</u>

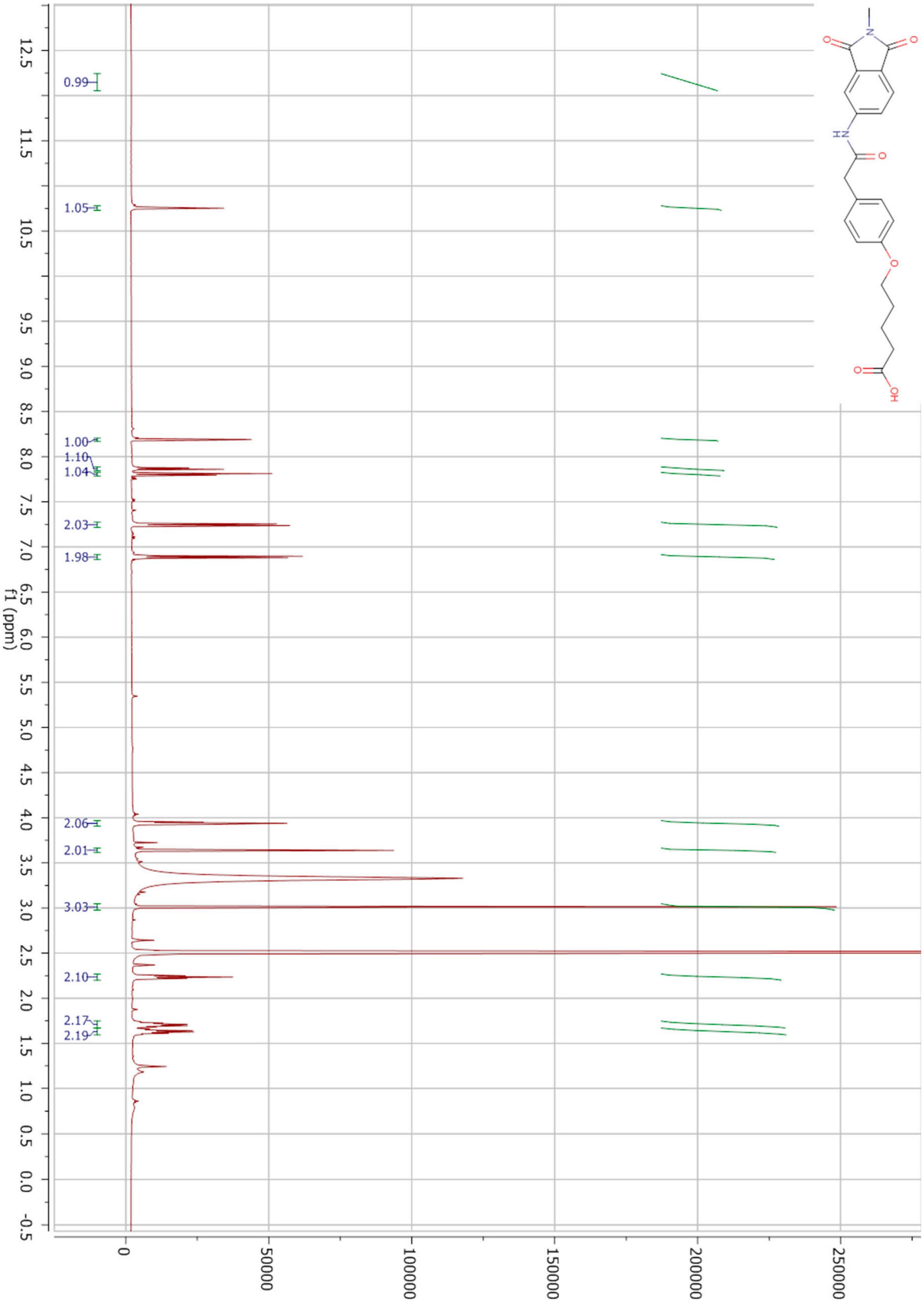

5-(4-{[(2-methyl-1,3-dioxo-2,3-dihydro-1H-isoindol-5-yl)carbamoyl]methyl}phenoxy)pentanoic acid (**14a**; ZHAWOC6649)

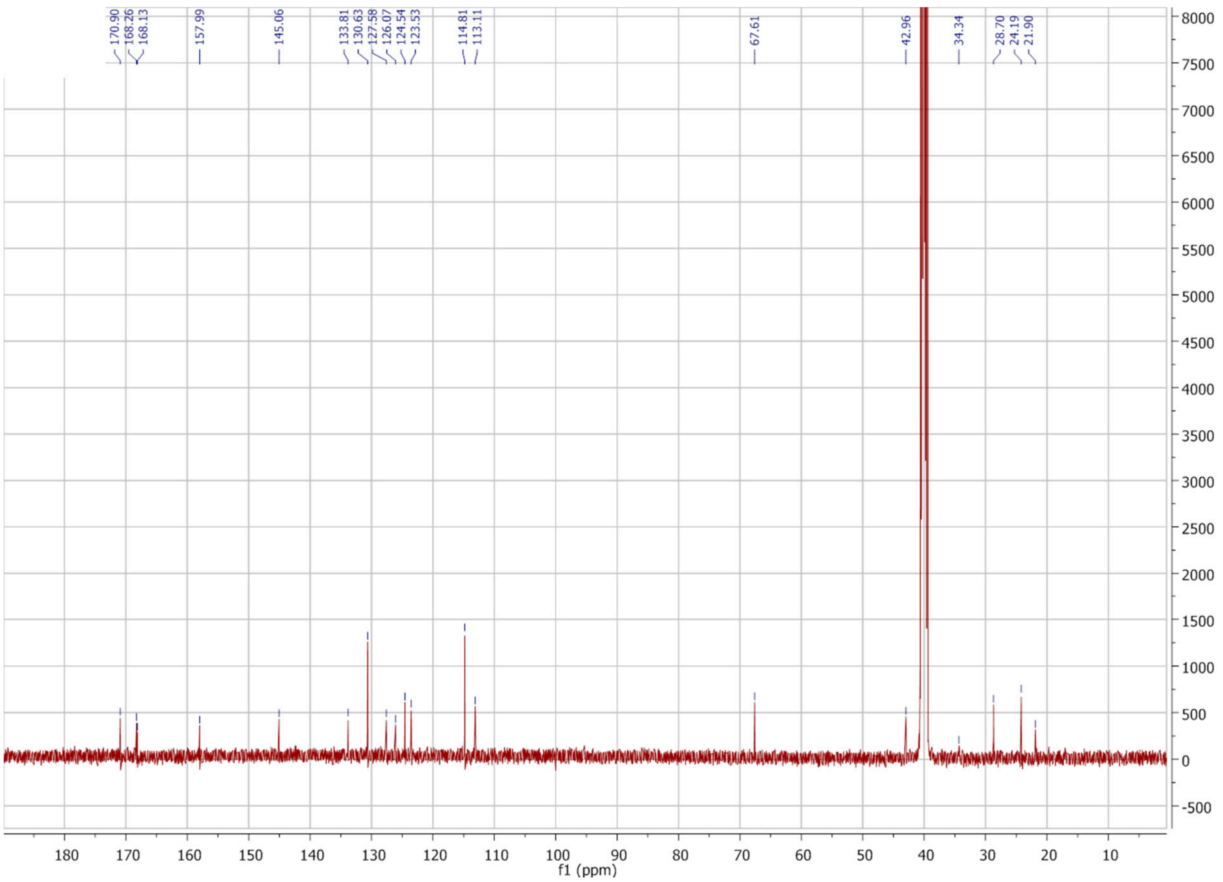

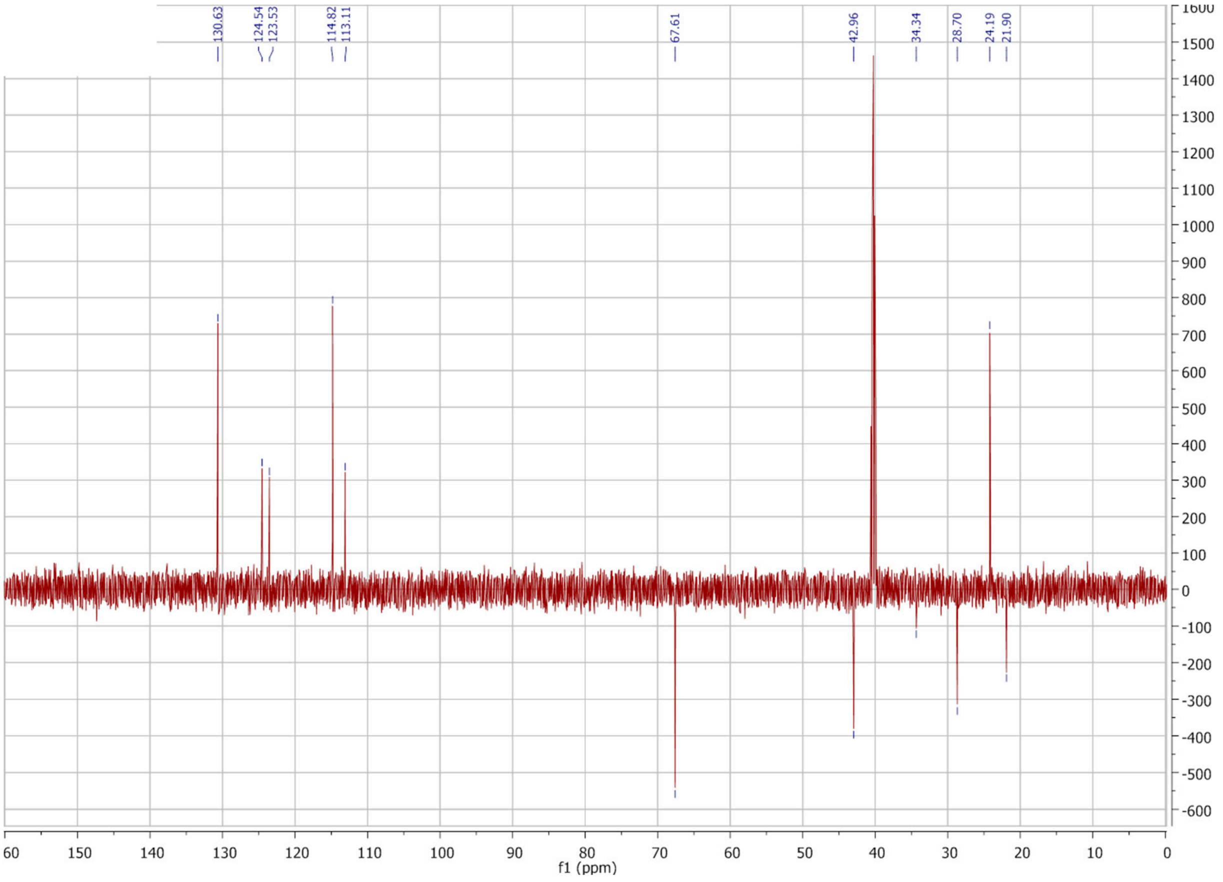

5-(4-{[(2-methyl-1,3-dioxo-2,3-dihydro-1H-isoindol-5-yl)carbamoyl]methyl}phenoxy)pentanoic acid (**14a**; ZHAWOC6649)

<u>HRMS</u>

Best	Formula	Species	m/z	Diff (ppm	Score (MFG)
WAHR	C22 H22 N	(M+H)+	411.1552	-0.18	99.88

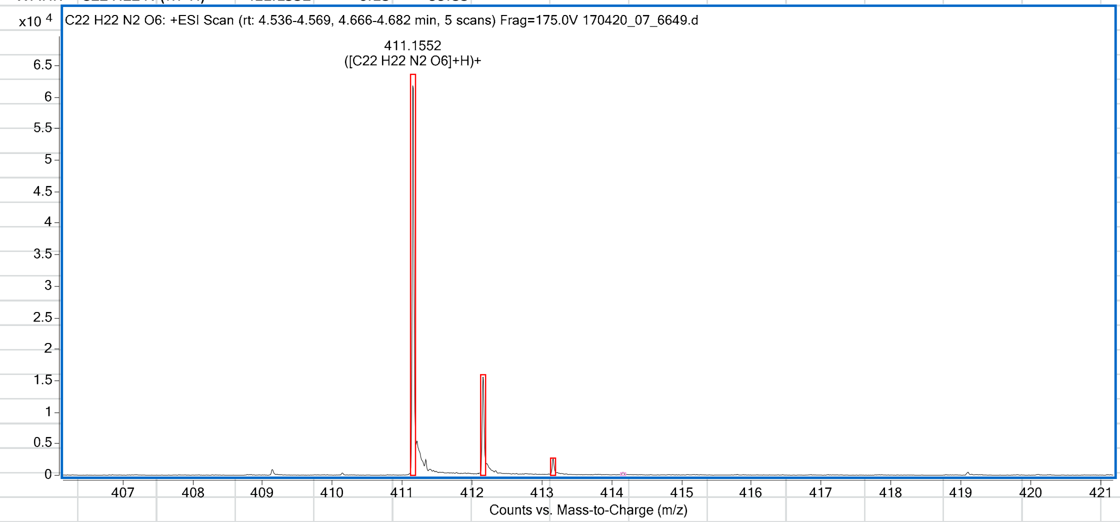

2-(4-{[(2-benzyl-1,3-dioxo-2,3-dihydro-1H-isoindol-5-yl)carbamoyl]methyl}phenoxy)acetic acid (**14b**; ZHAWOC5474)

<u>NMR</u>

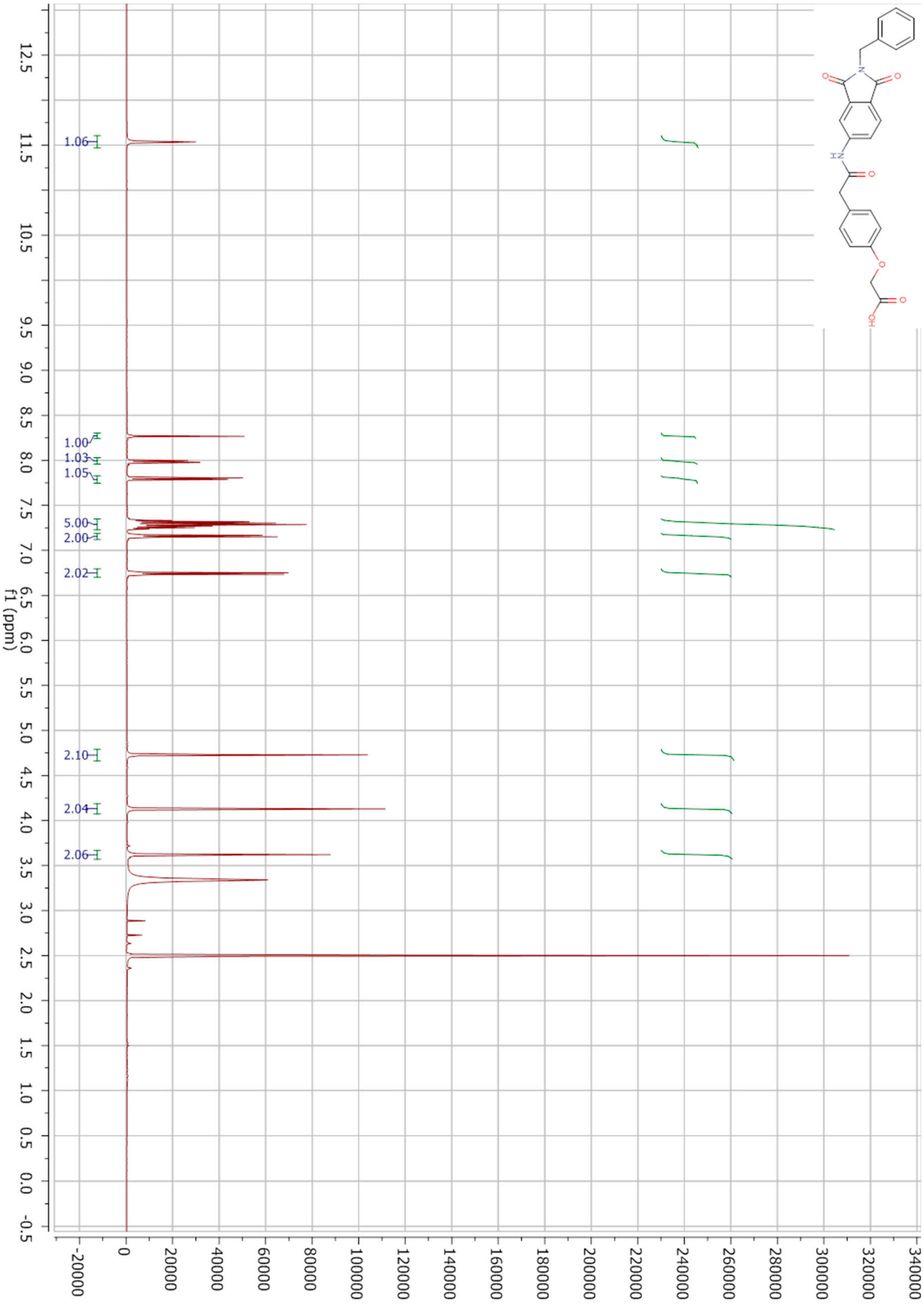

2-(4-{[(2-benzyl-1,3-dioxo-2,3-dihydro-1H-isoindol-5-yl)carbamoyl]methyl}phenoxy)acetic acid (**14b**; ZHAWOC5474)

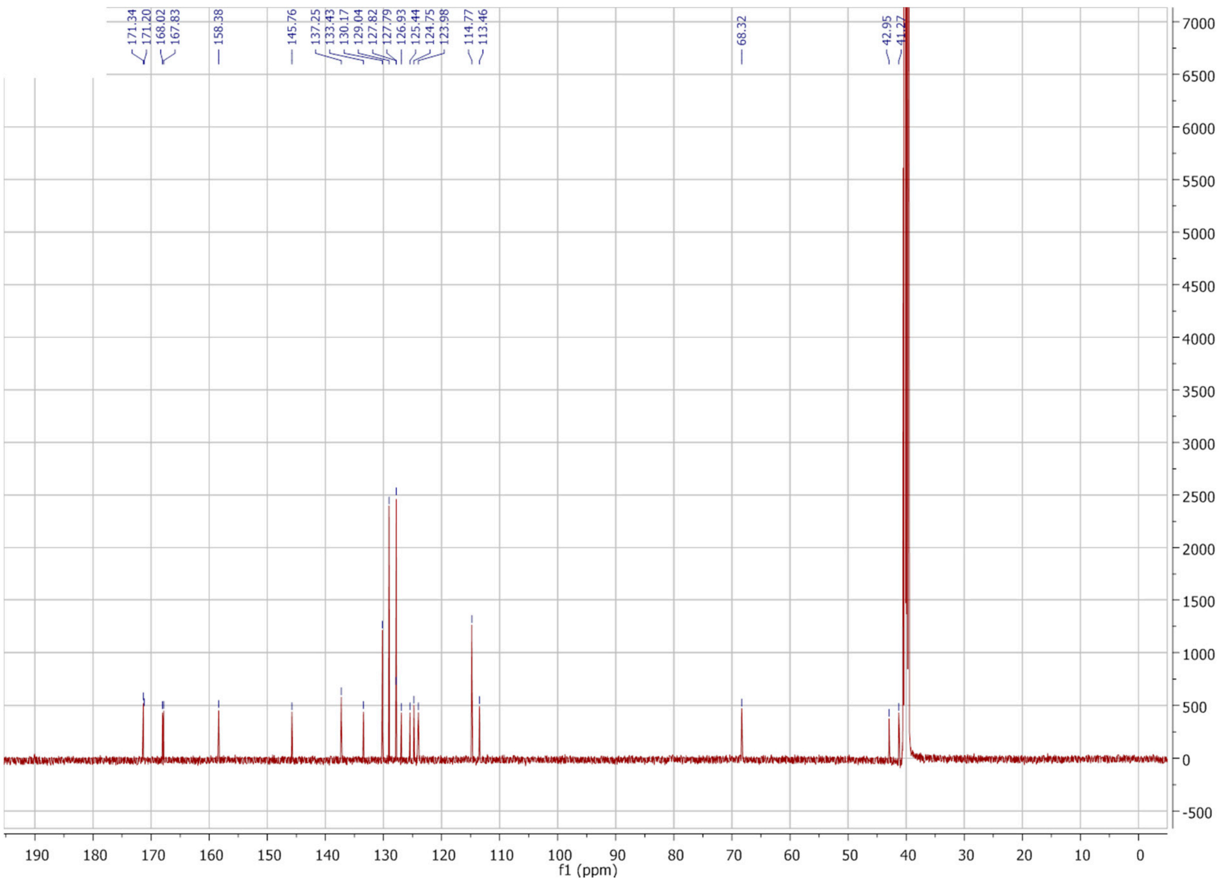

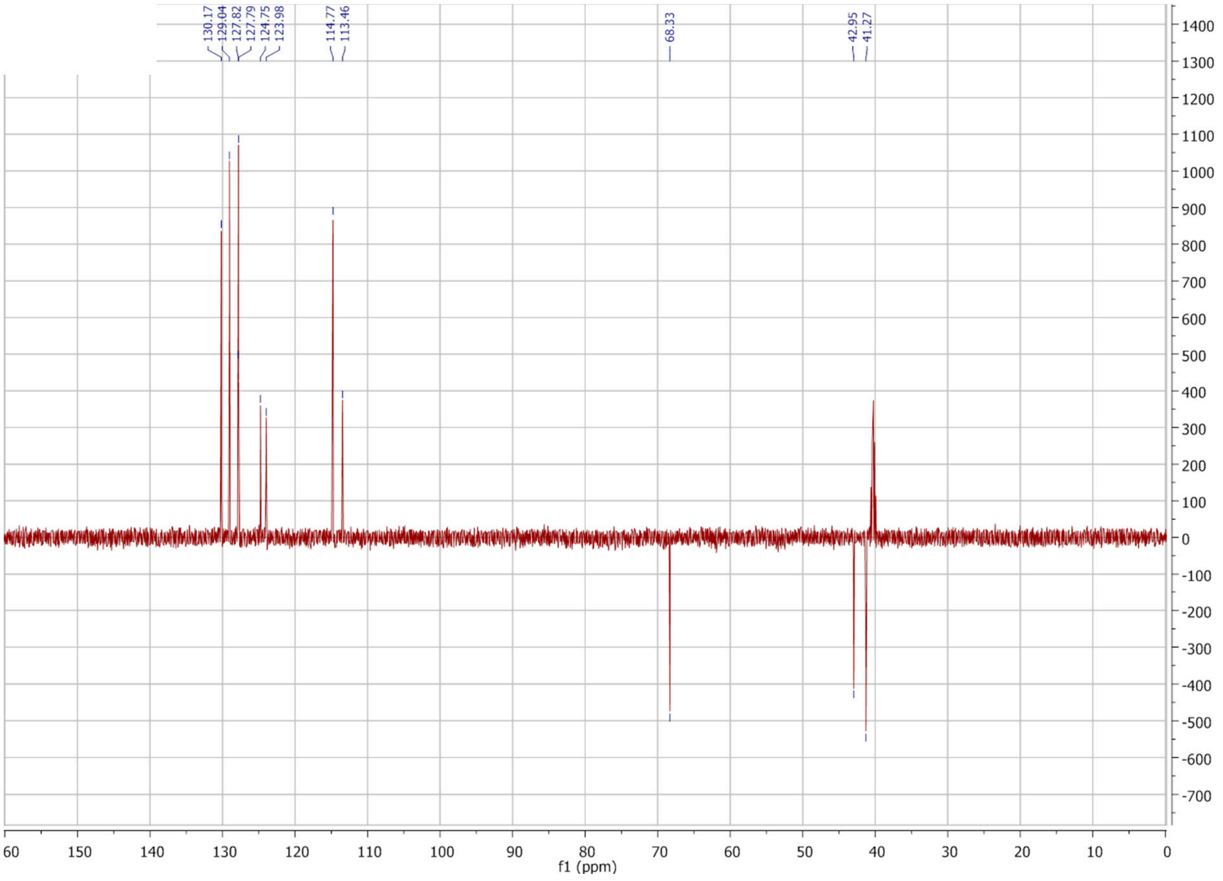

2-(4-{[(2-benzyl-1,3-dioxo-2,3-dihydro-1H-isoindol-5-yl)carbamoyl]methyl}phenoxy)acetic acid (**14b**; ZHAWOC5474)

<u>HRMS</u>

Best	Formula	Species	m/z	Diff (ppm)	Score (MFG)
WAHR	C25 H20 N	(M+H)+	445.1409	-3.73	93.41

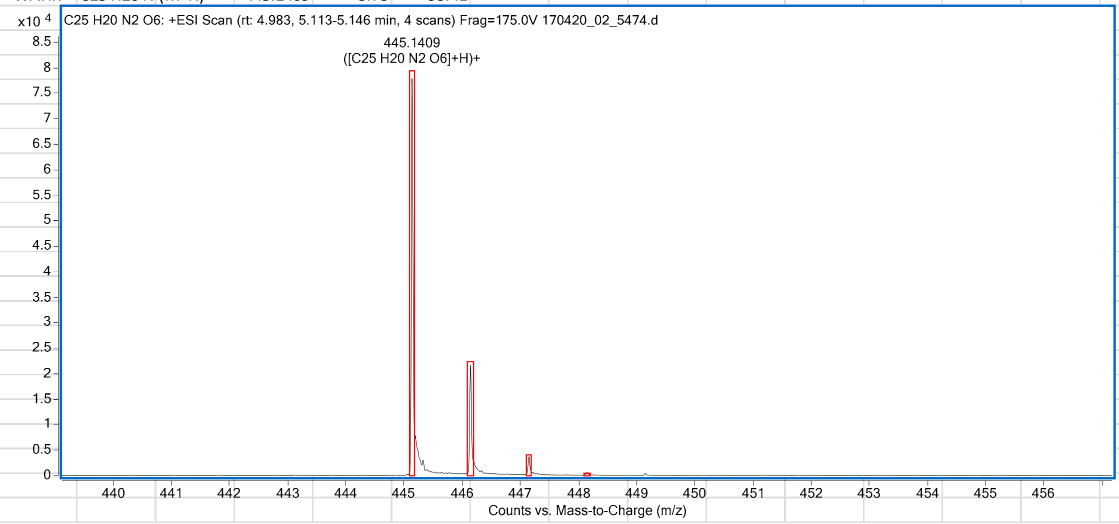

3-(4-{[(2-benzyl-1,3-dioxo-2,3-dihydro-1H-isoindol-5-yl)carbamoyl]methyl}phenoxy)propanoic acid (**14c;** ZHAWOC4765)

<u>NMR</u>

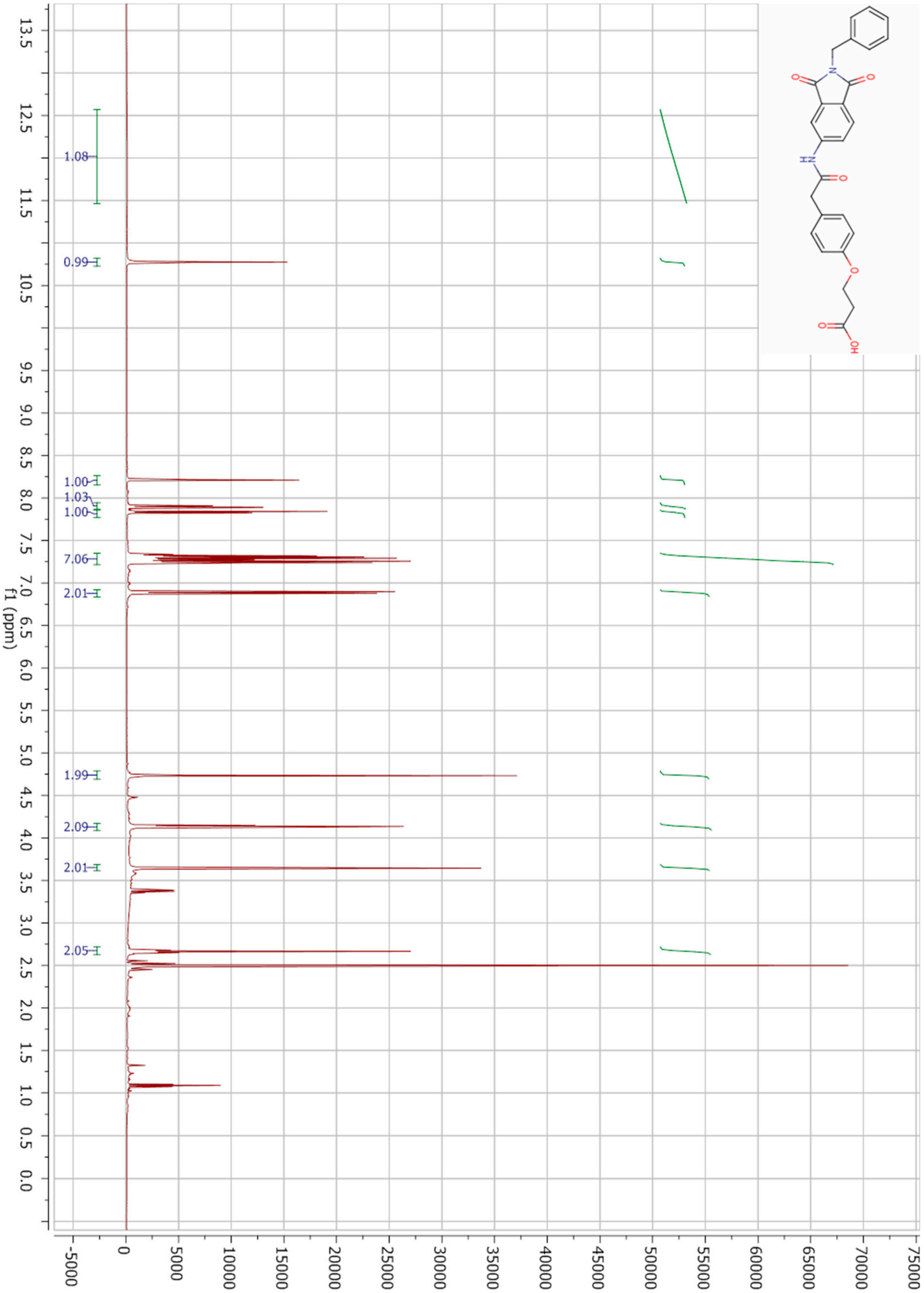

3-(4-{[(2-benzyl-1,3-dioxo-2,3-dihydro-1H-isoindol-5-yl)carbamoyl]methyl}phenoxy)propanoic acid (**14c;** ZHAWOC4765)

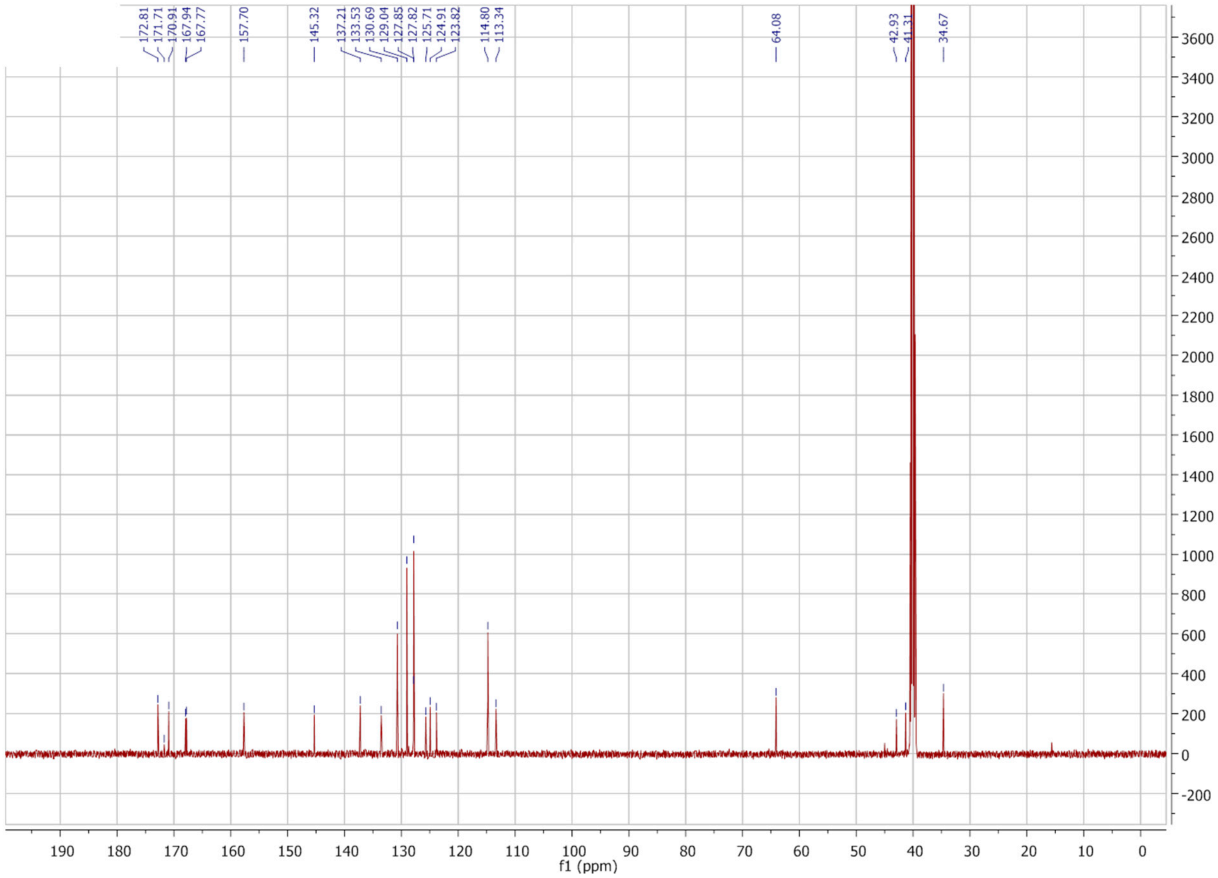

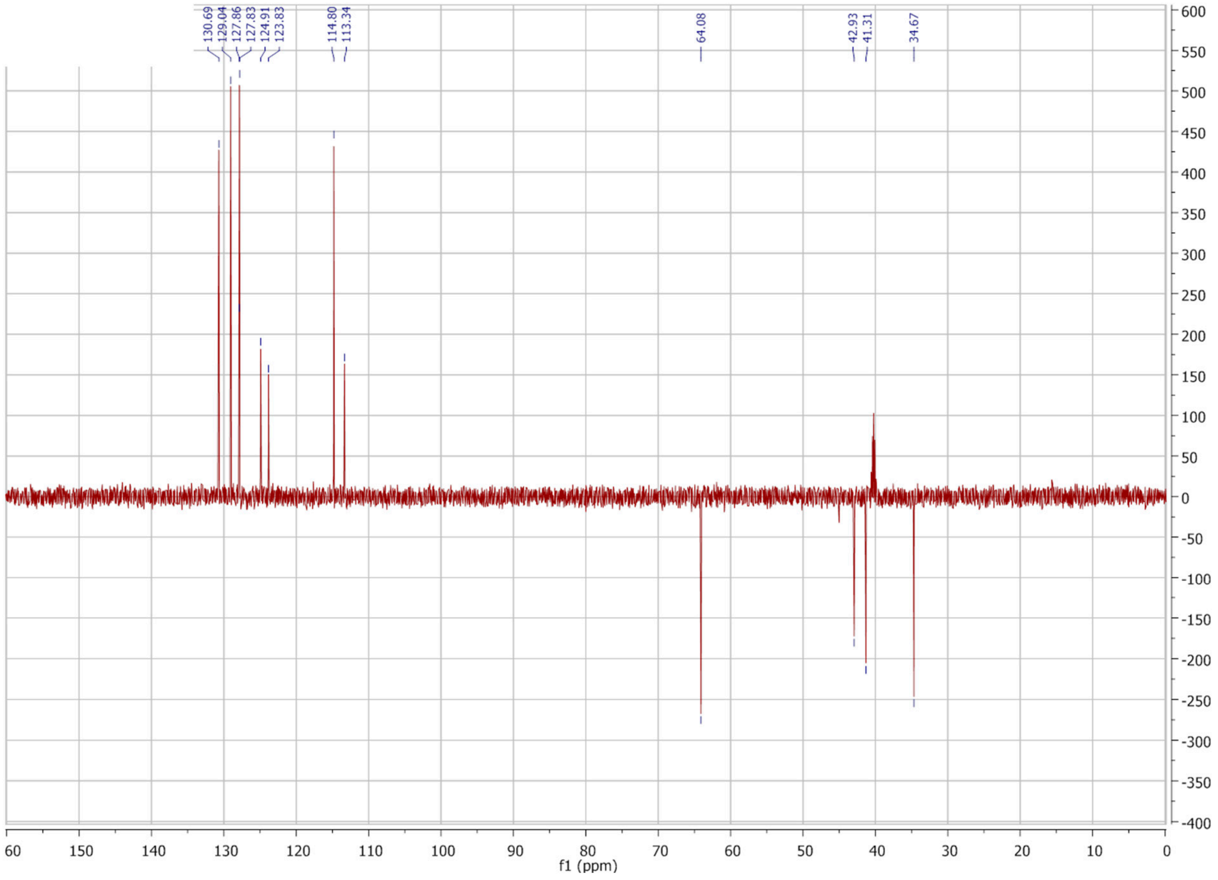

3-(4-{[(2-benzyl-1,3-dioxo-2,3-dihydro-1H-isoindol-5-yl)carbamoyl]methyl}phenoxy)propanoic acid (**14c;** ZHAWOC4765)

HRMS

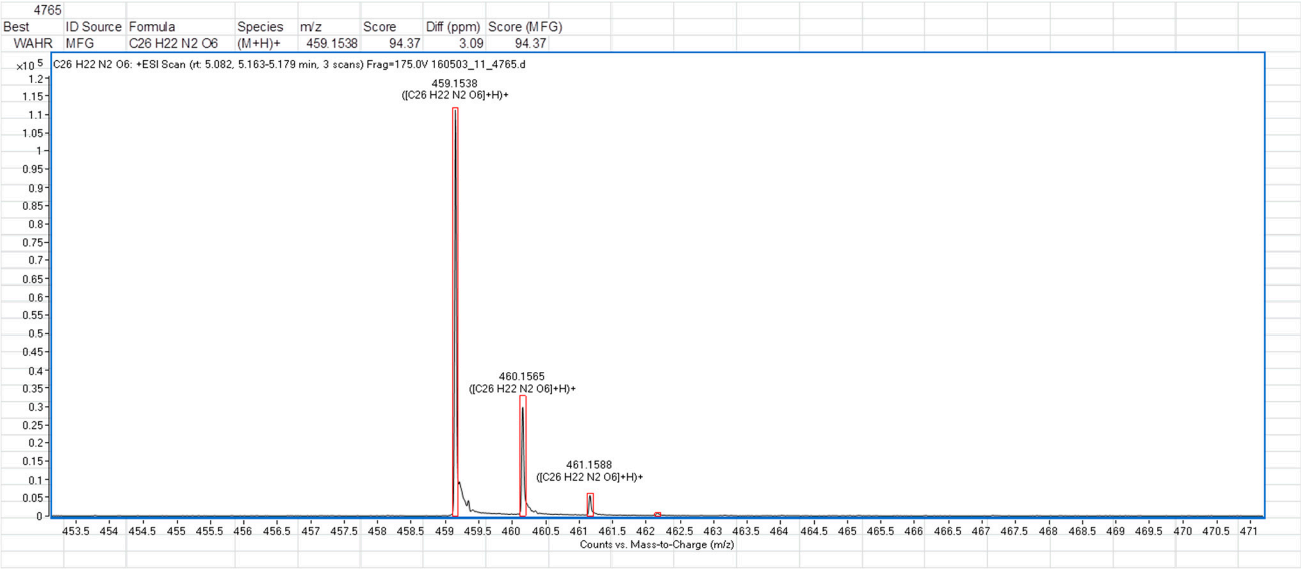

IC$_{50}$

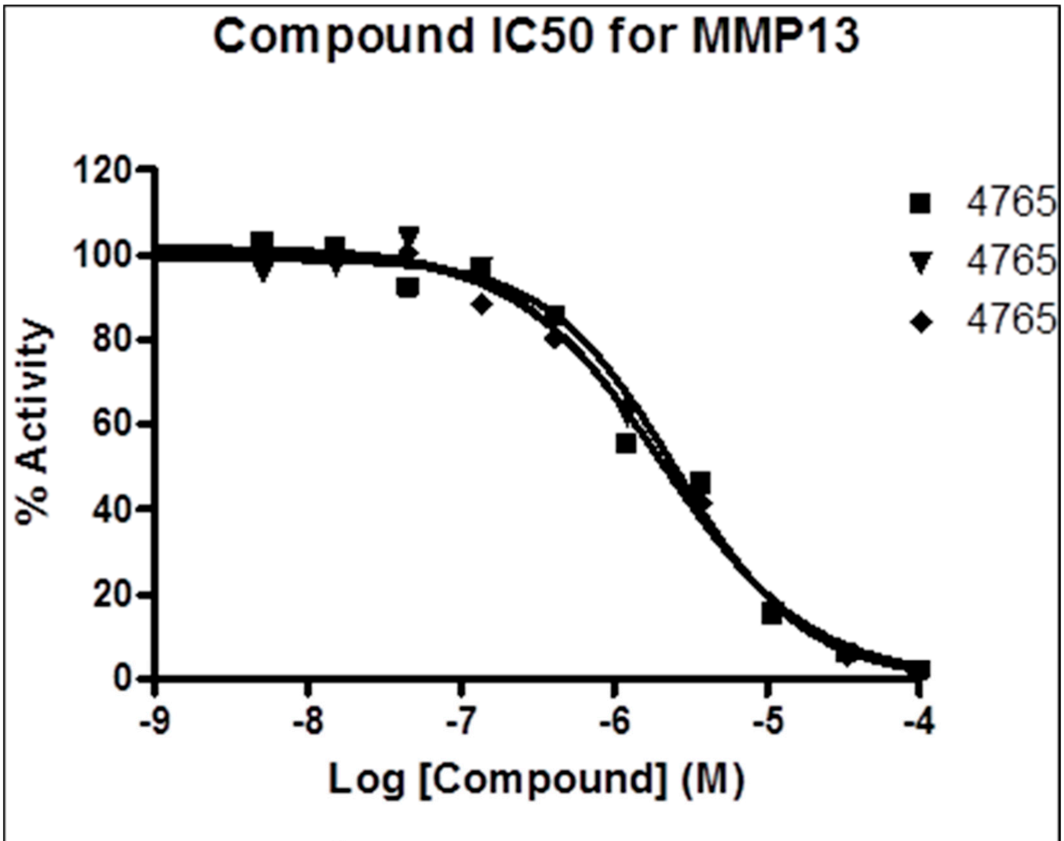

	4765	4765	4765
HILLSLOPE	-0.9016	-1.021	-0.8988
EC50	2.086e-006	2.527e-006	2.120e-006

4-(4-{[(2-benzyl-1,3-dioxo-2,3-dihydro-1H-isoindol-5-yl)carbamoyl]methyl}phenoxy)butanoic acid (**14d;** ZHAWOC4766)

NMR

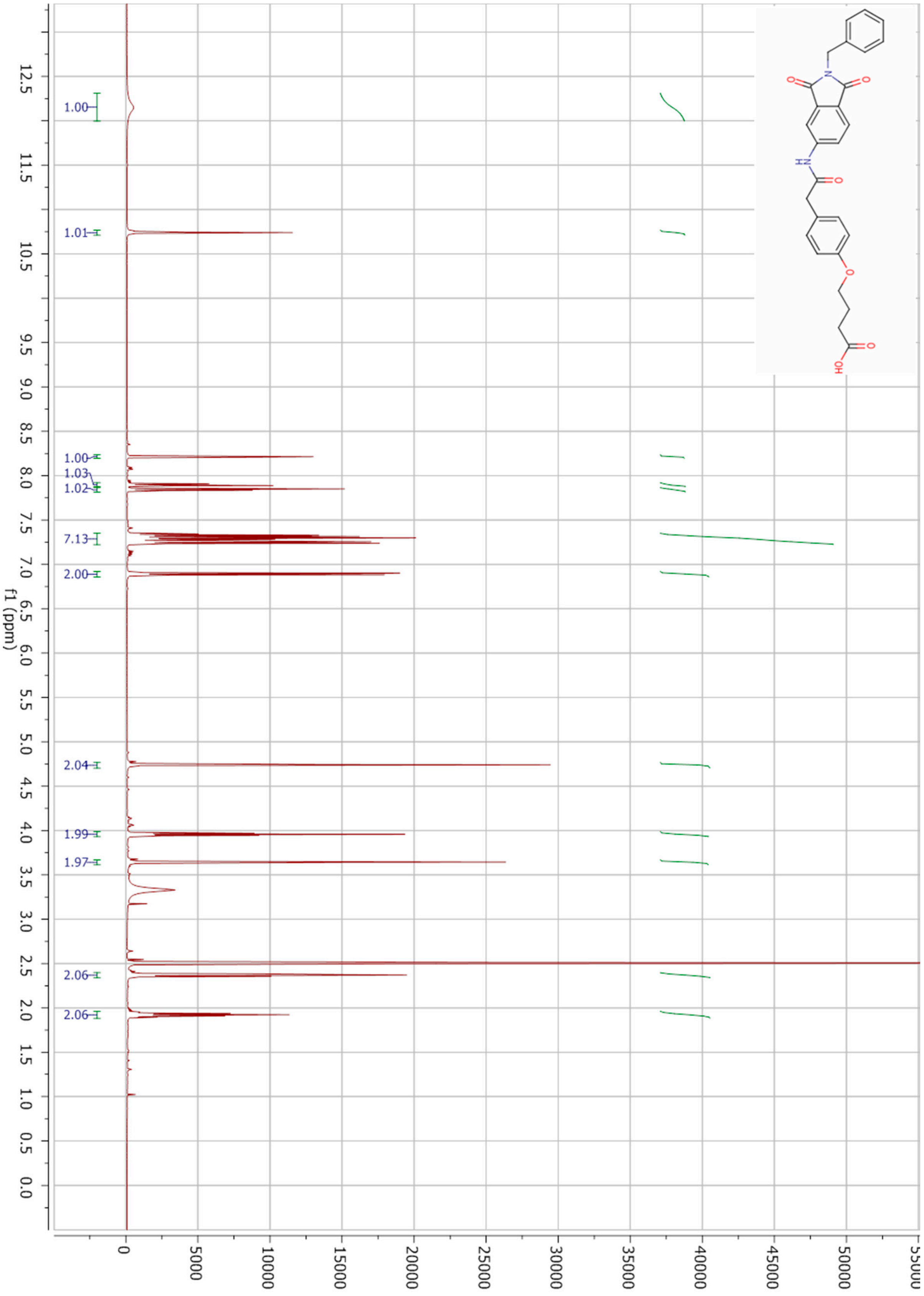

4-(4-{[(2-benzyl-1,3-dioxo-2,3-dihydro-1H-isoindol-5-yl)carbamoyl]methyl}phenoxy)butanoic acid (**14d;** ZHAWOC4766)

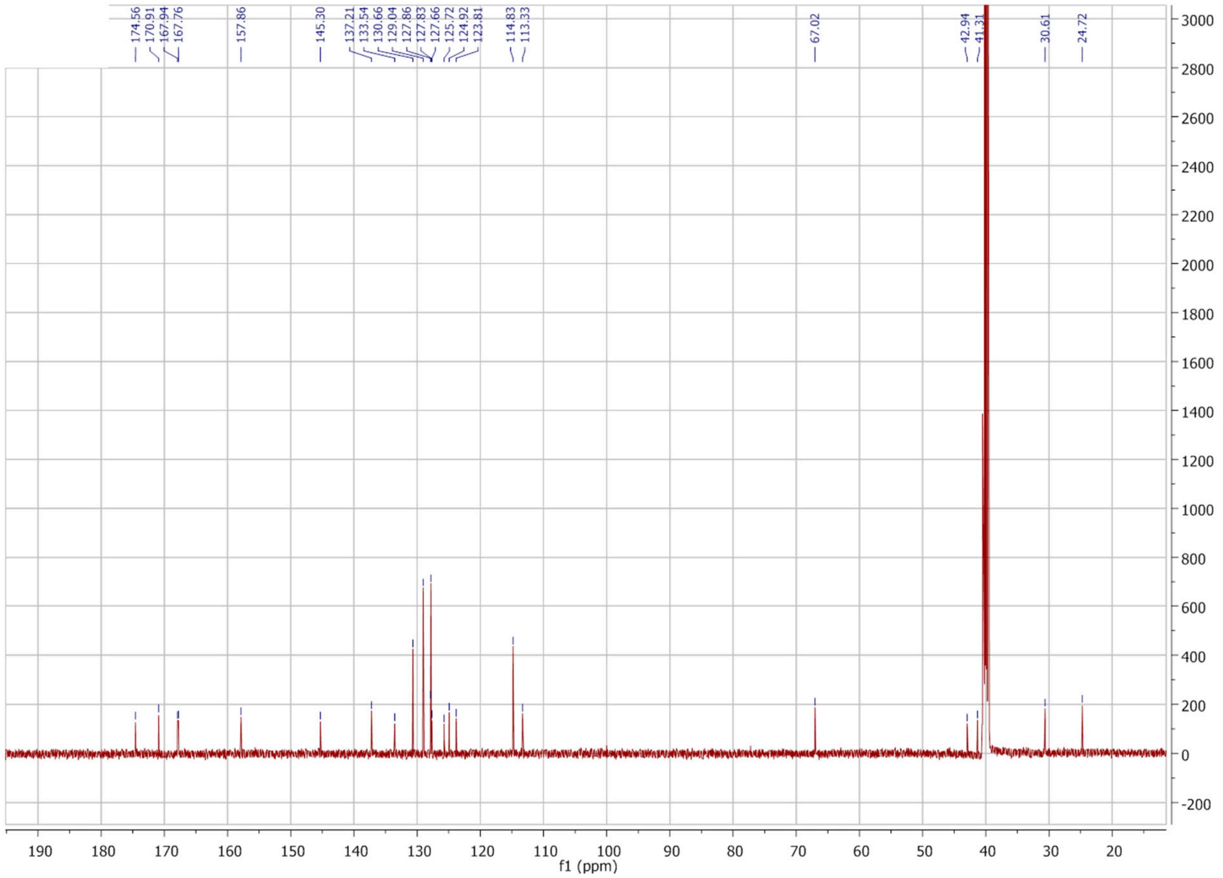

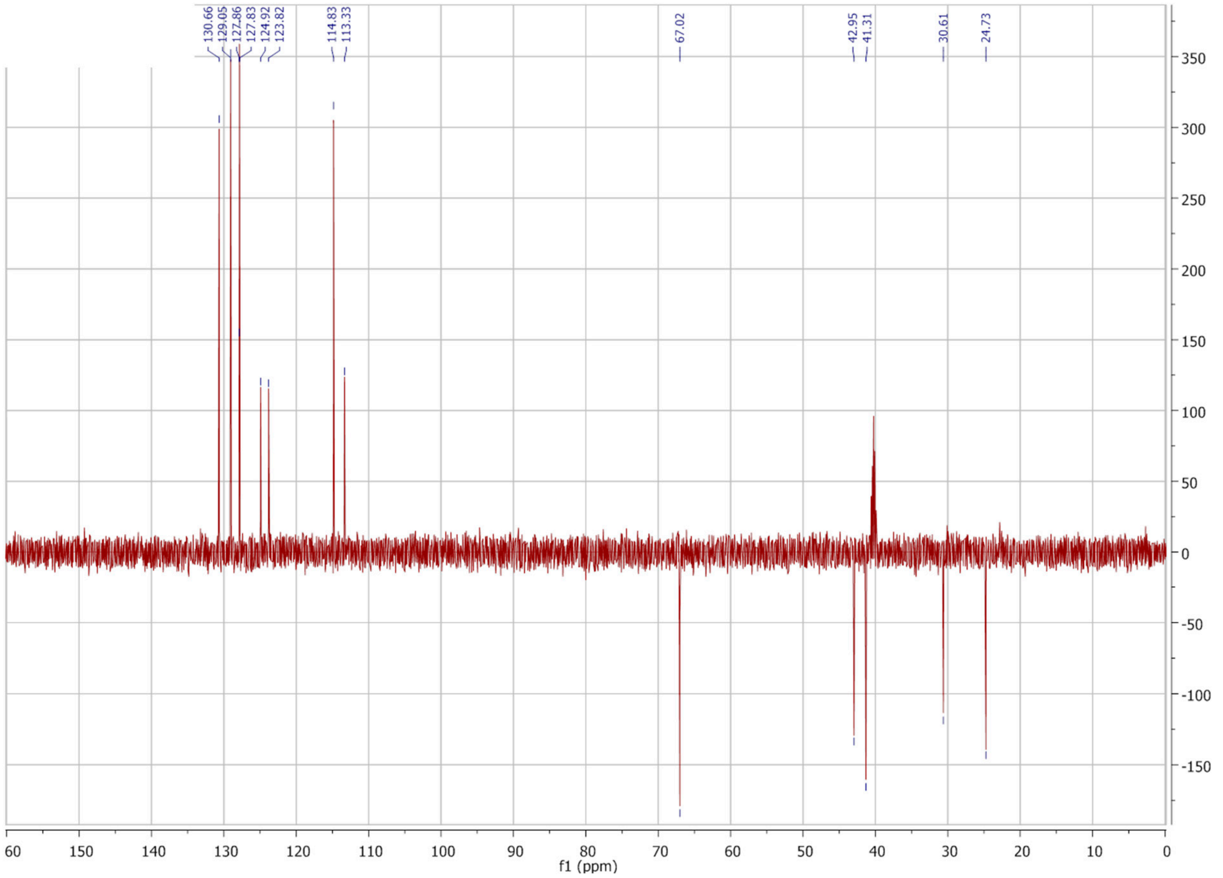

4-(4-{[(2-benzyl-1,3-dioxo-2,3-dihydro-1H-isoindol-5-yl)carbamoyl]methyl}phenoxy)butanoic acid (**14d;** ZHAWOC4766)

HRMS

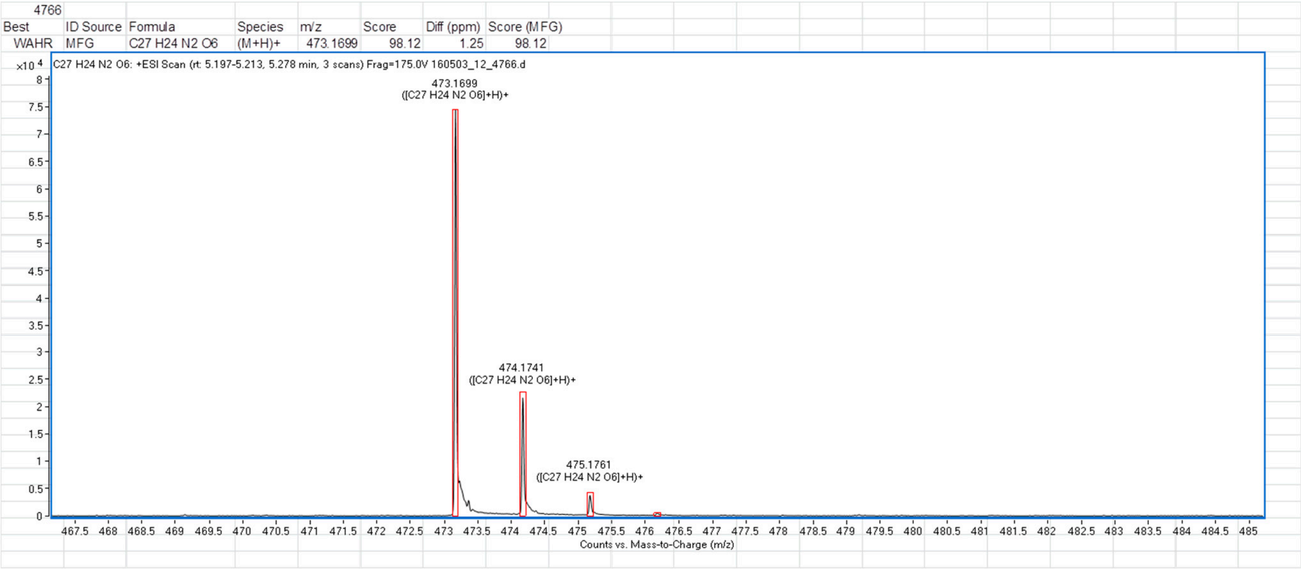

IC50

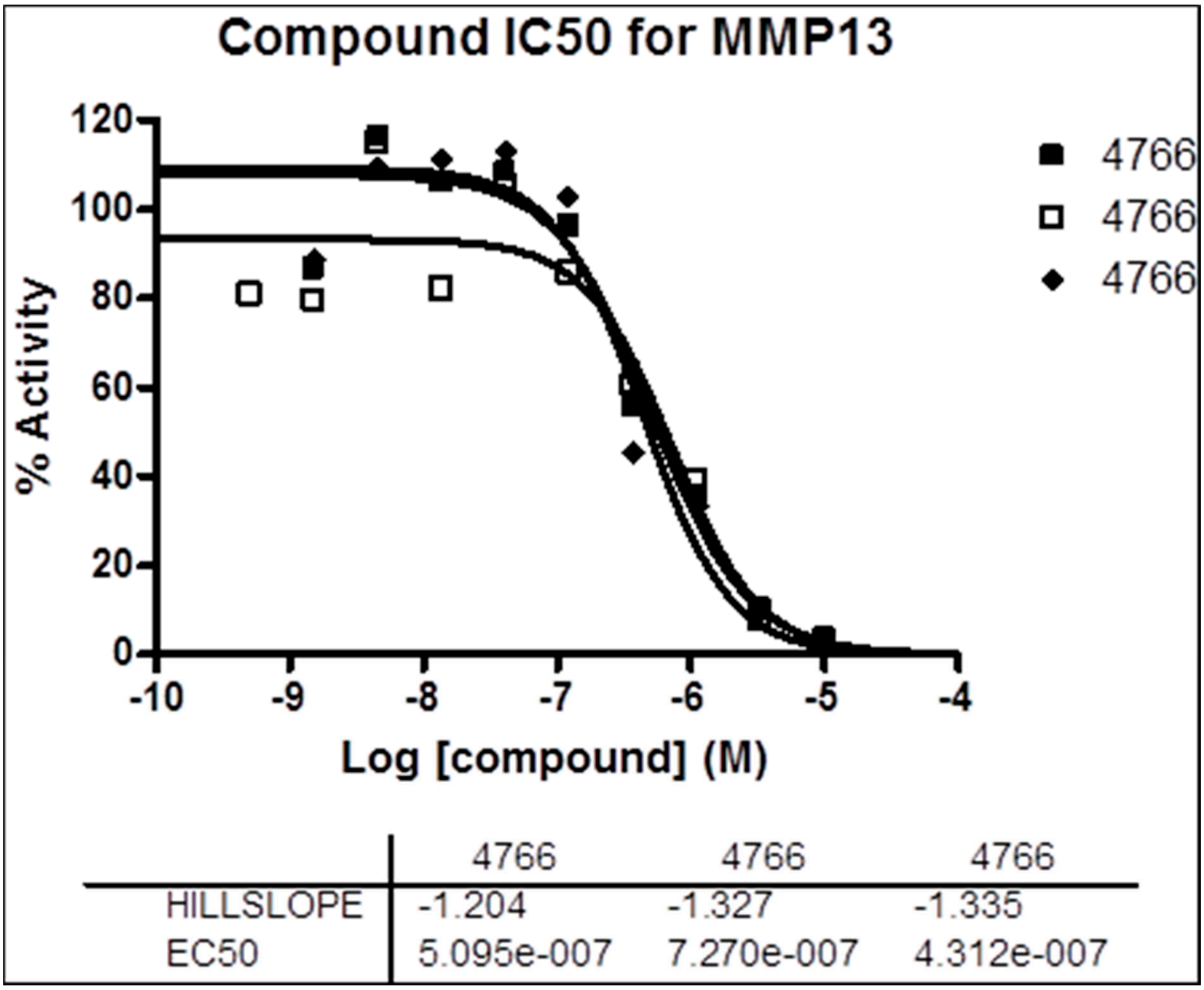

5-(4-{[(2-benzyl-1,3-dioxo-2,3-dihydro-1H-isoindol-5-yl)carbamoyl]methyl}phenoxy)pentanoic acid (**14e;** ZHAWOC5131)

<u>NMR</u>

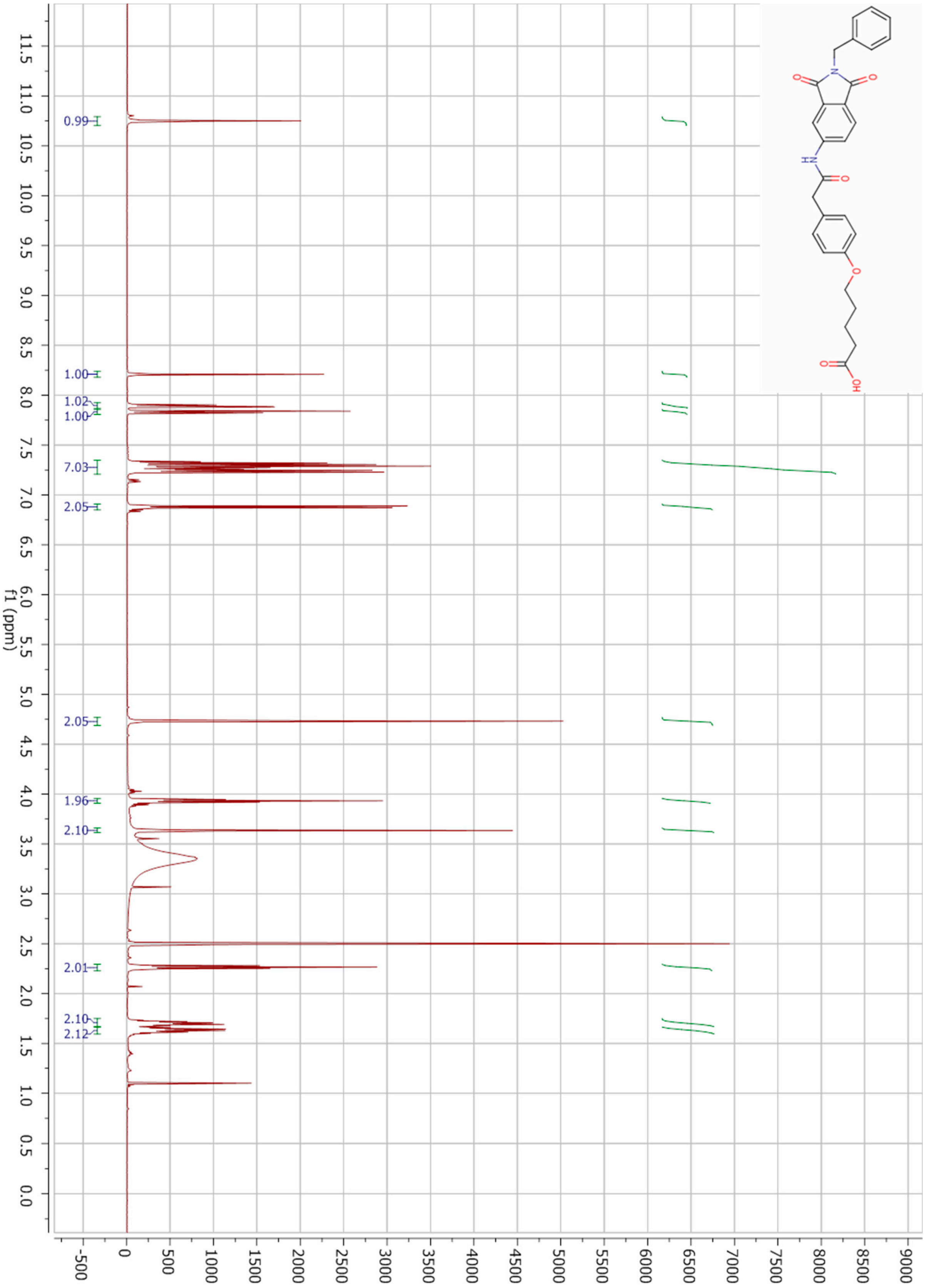

5-(4-{[(2-benzyl-1,3-dioxo-2,3-dihydro-1H-isoindol-5-yl)carbamoyl]methyl}phenoxy)pentanoic acid (**14e;** ZHAWOC5131)

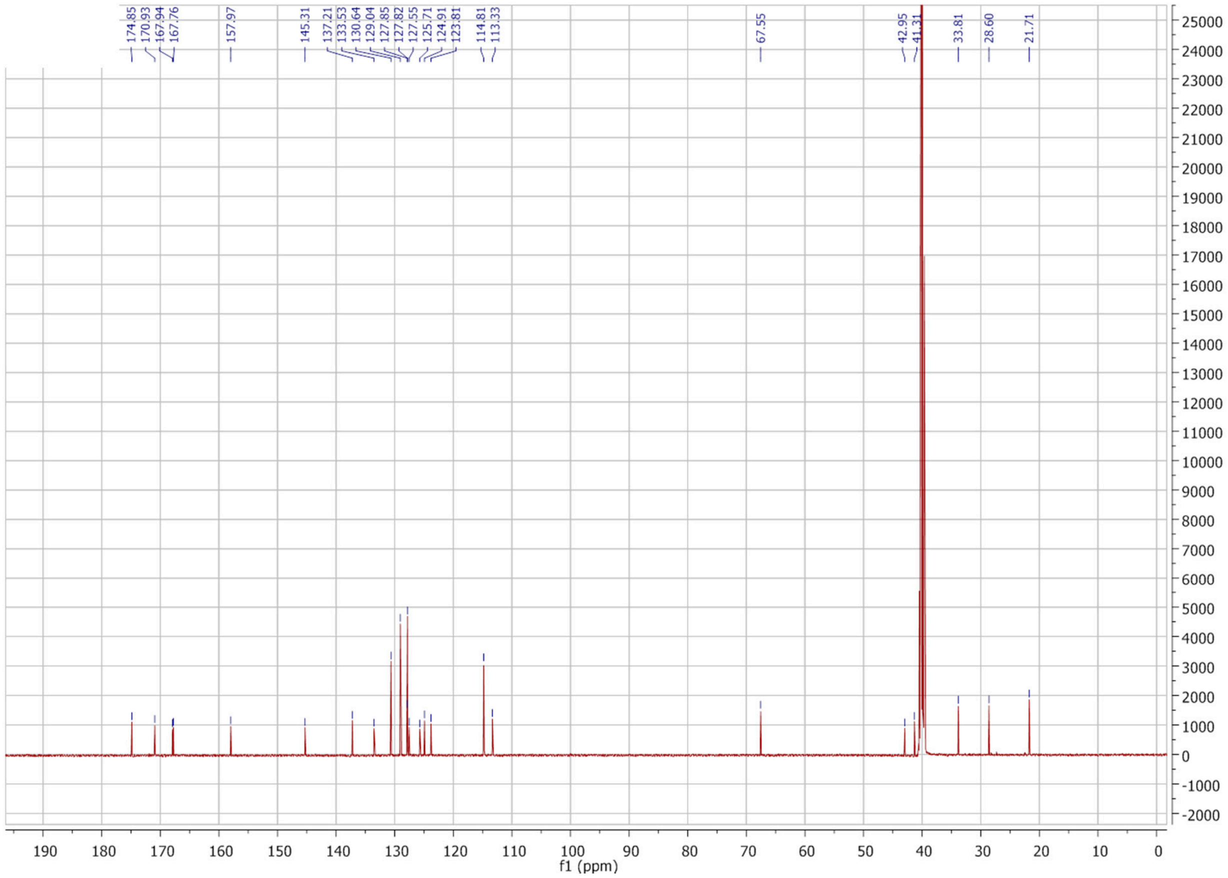

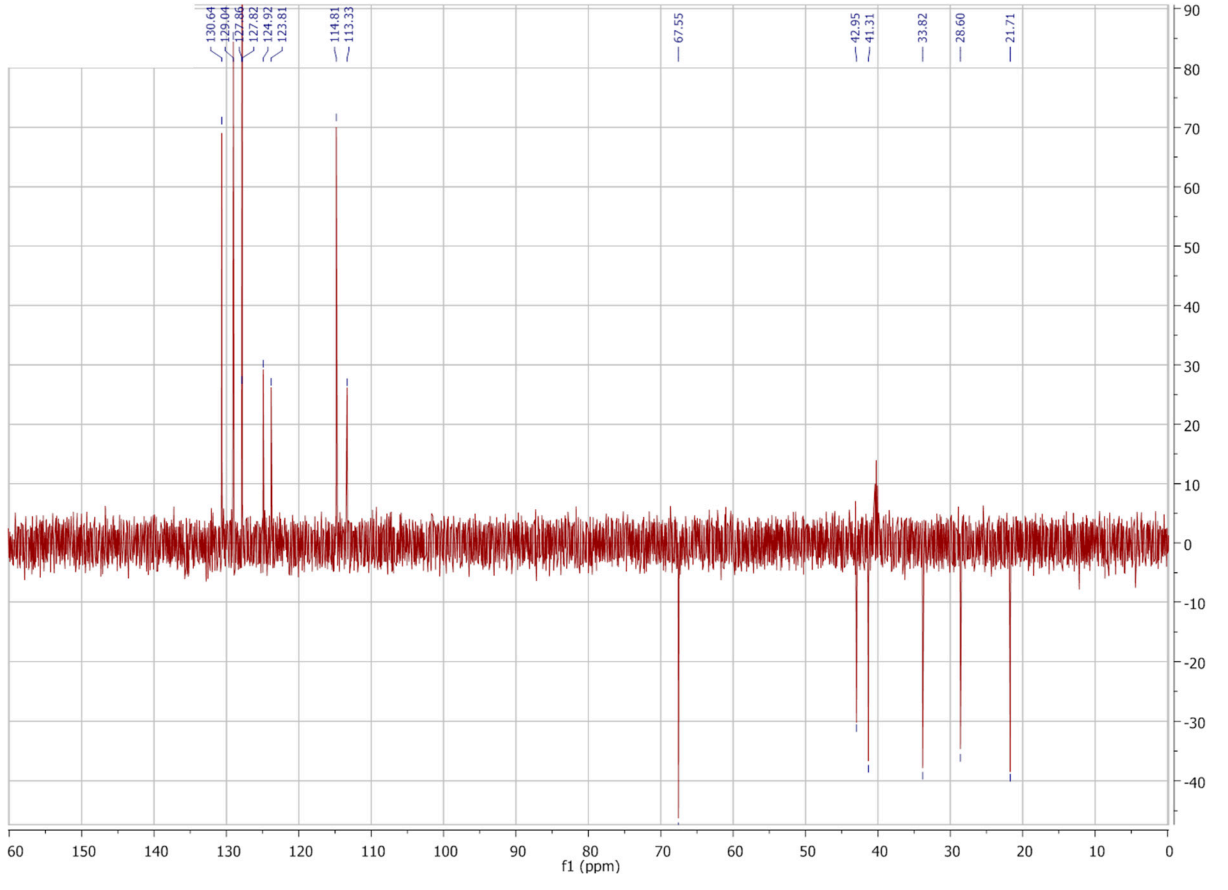

5-(4-{[(2-benzyl-1,3-dioxo-2,3-dihydro-1H-isoindol-5-yl)carbamoyl]methyl}phenoxy)pentanoic acid (**14e;** ZHAWOC5131)

HRMS

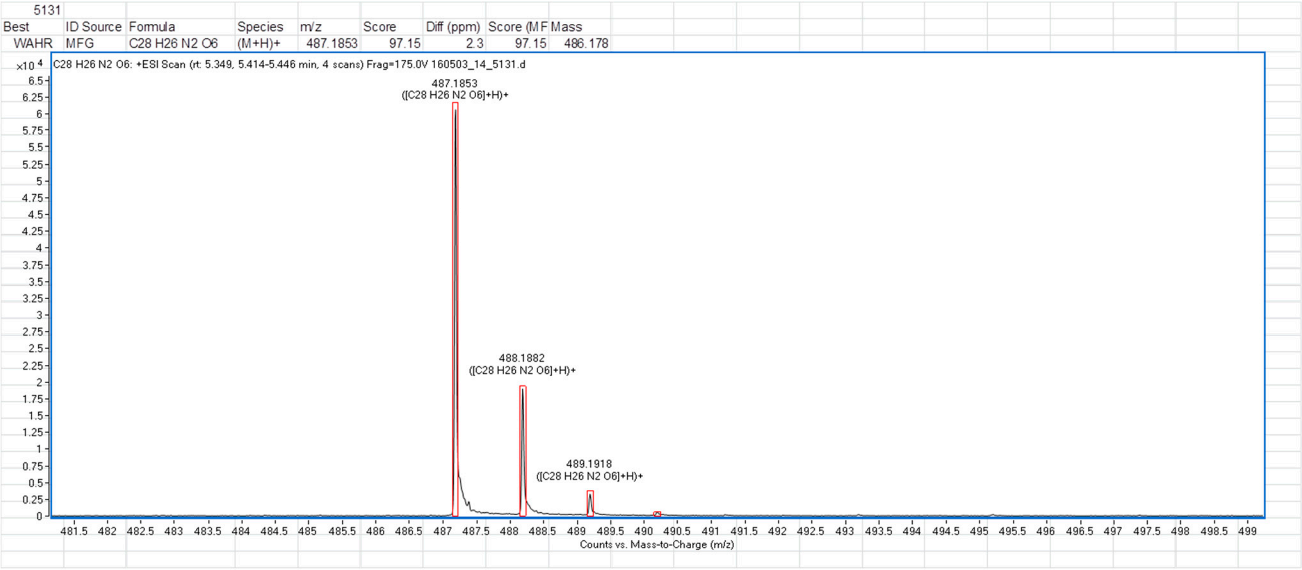

IC_{50}

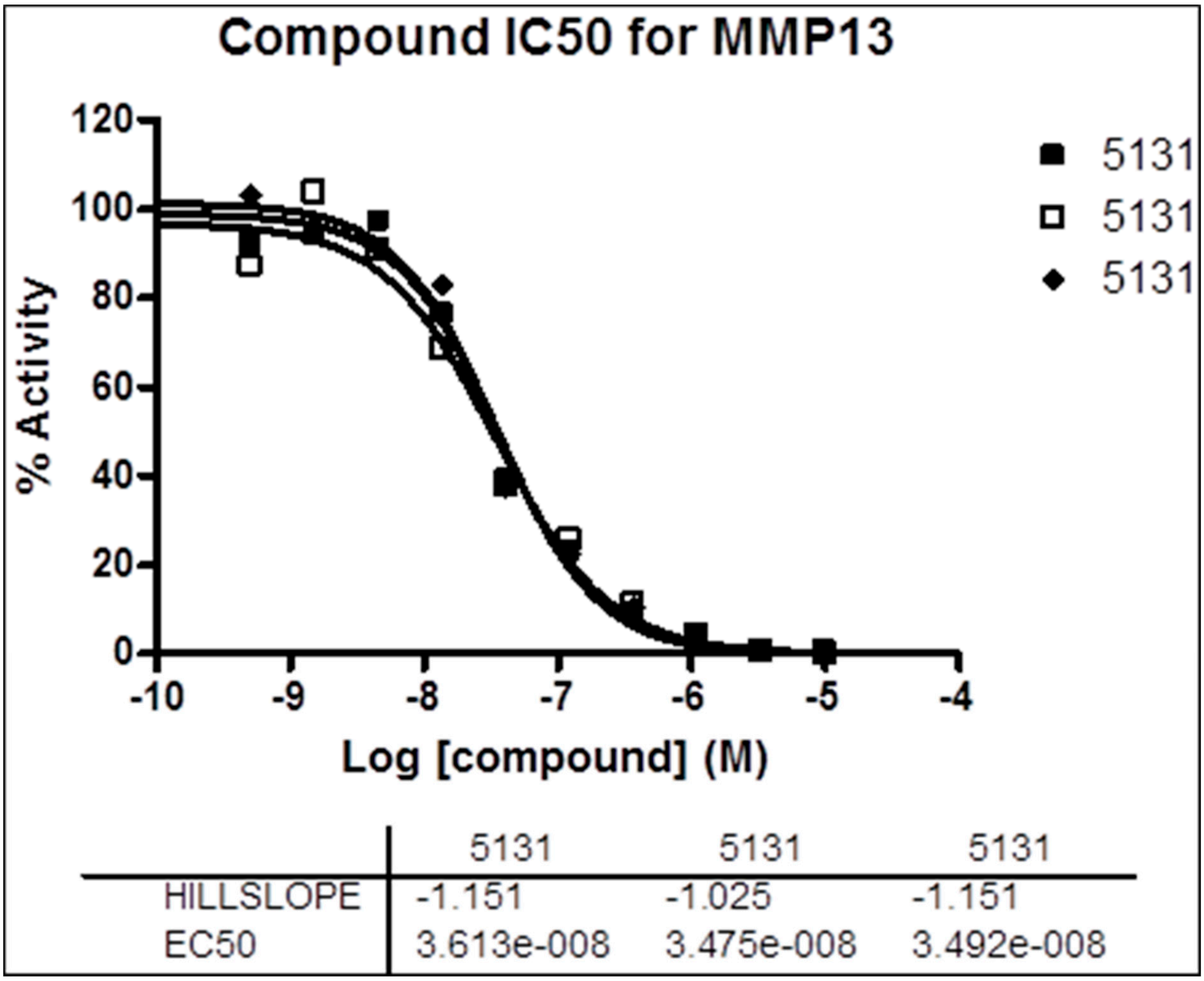

6-(4-{[(2-benzyl-1,3-dioxo-2,3-dihydro-1H-isoindol-5-yl)carbamoyl]methyl}phenoxy)hexanoic acid (**14f;** ZHAWOC5133)

<u>NMR</u>

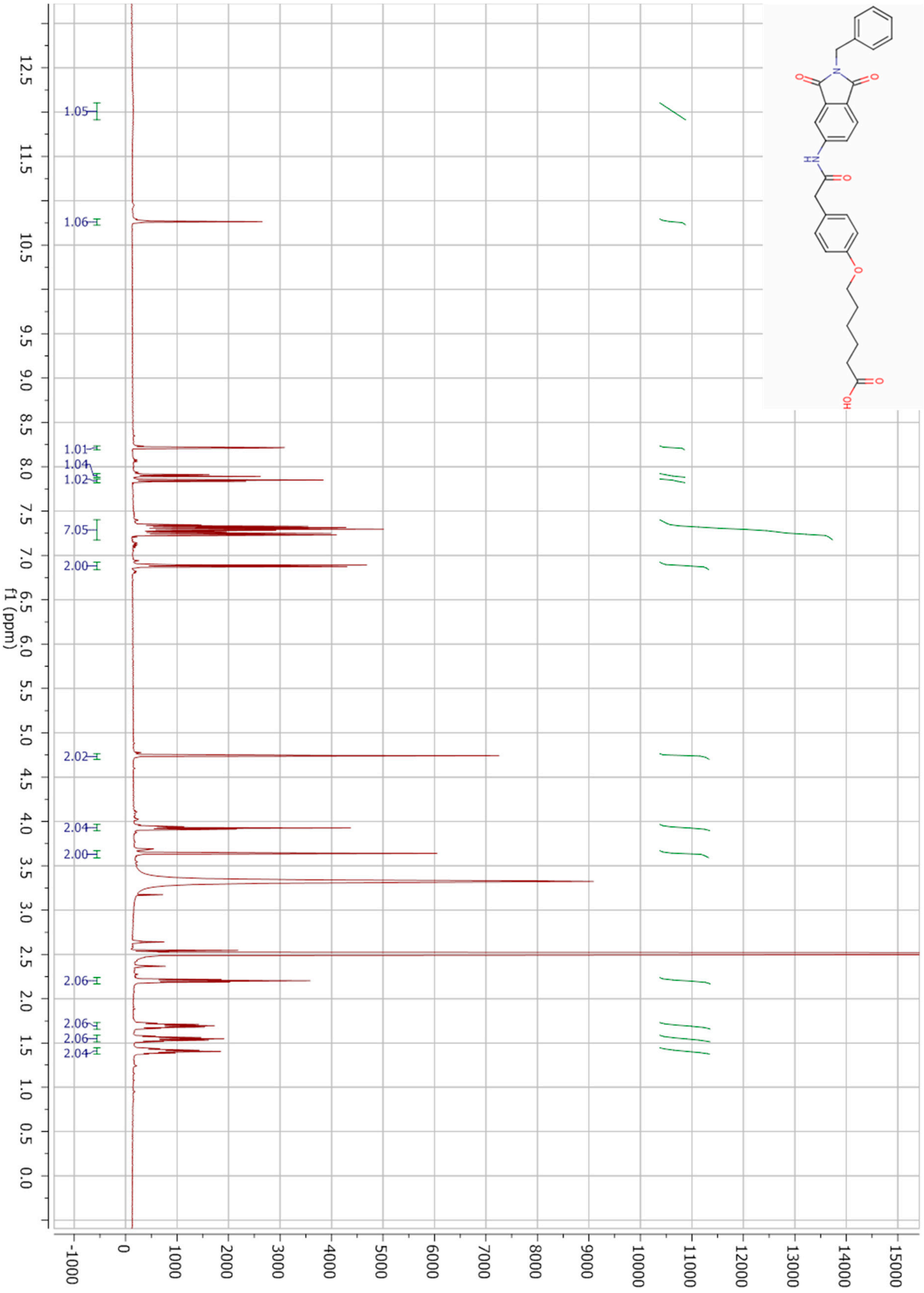

6-(4-{[(2-benzyl-1,3-dioxo-2,3-dihydro-1H-isoindol-5-yl)carbamoyl]methyl}phenoxy)hexanoic acid (**14f;** ZHAWOC5133)

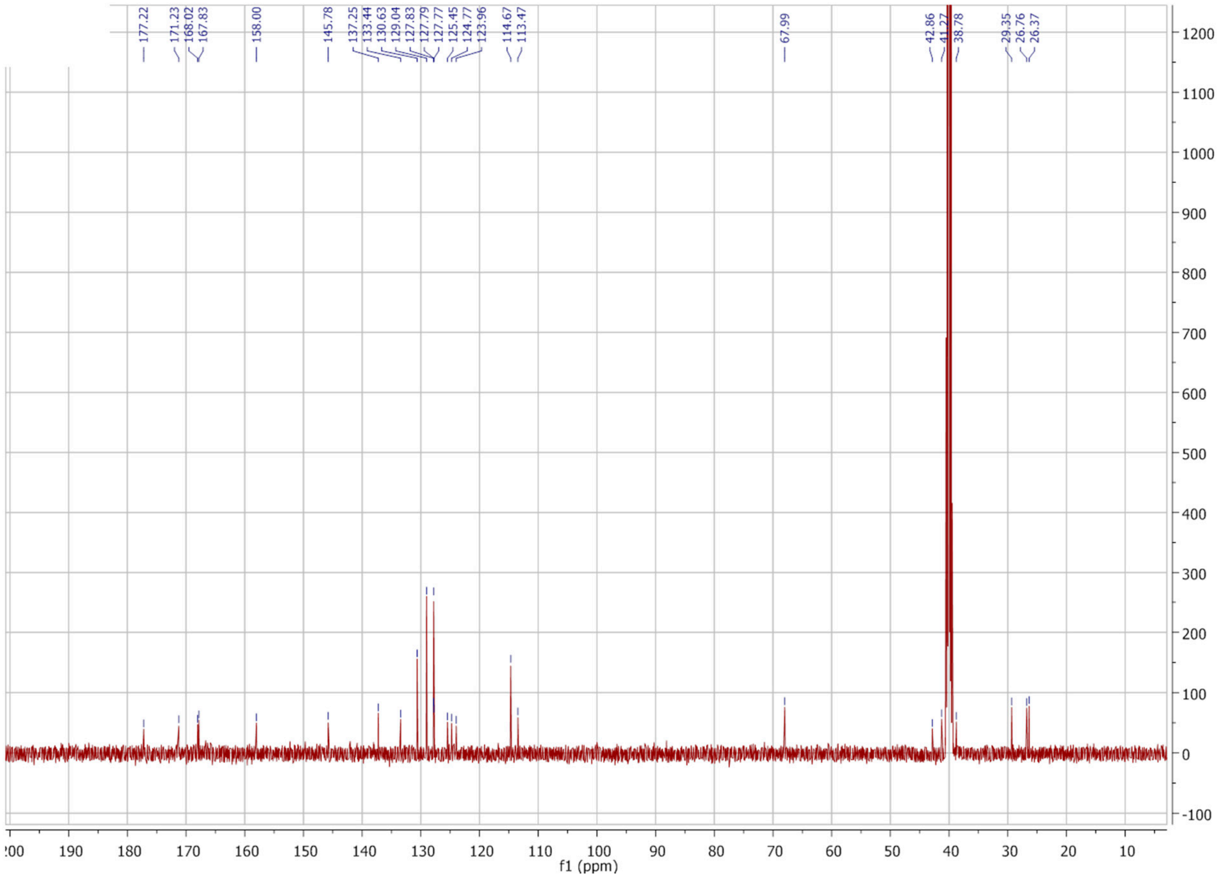

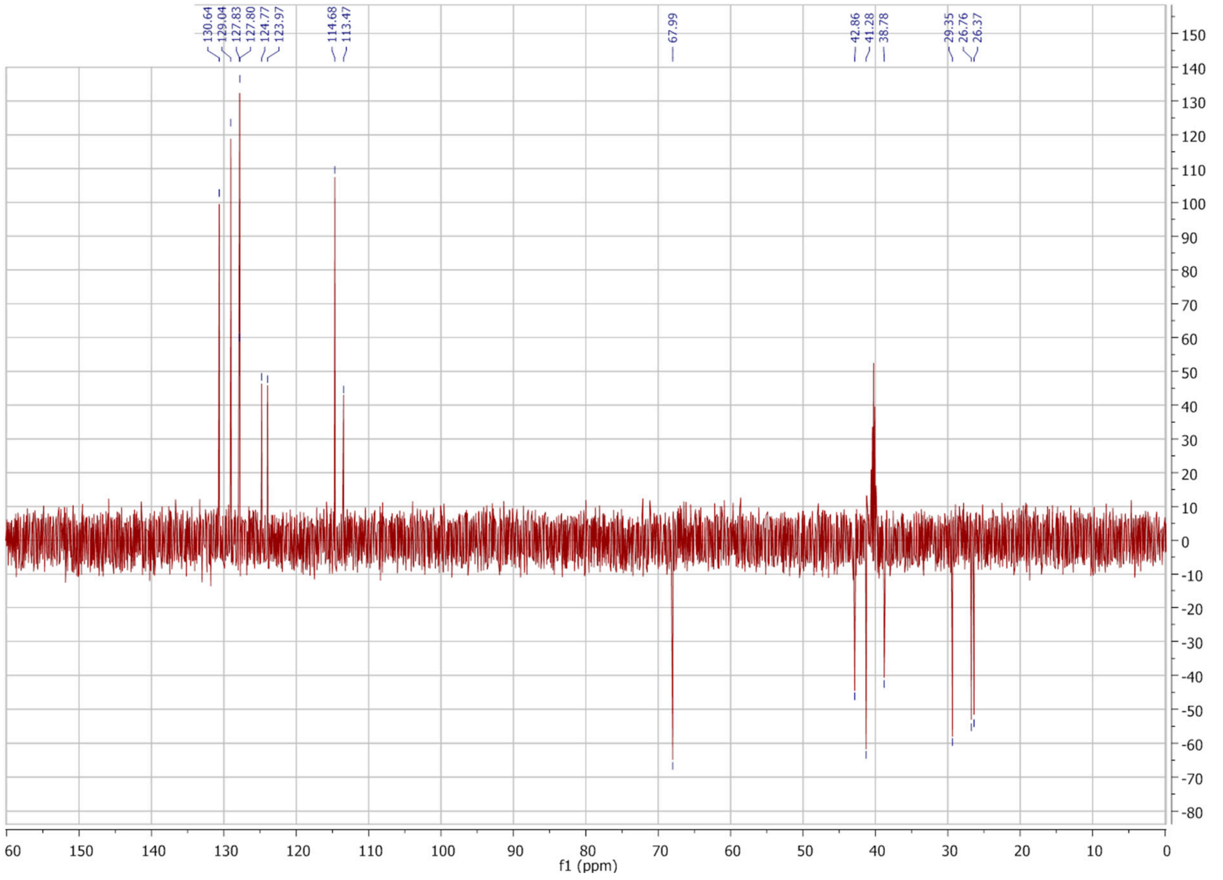

6-(4-{[(2-benzyl-1,3-dioxo-2,3-dihydro-1H-isoindol-5-yl)carbamoyl]methyl}phenoxy)hexanoic acid (**14f;** ZHAWOC5133)

HRMS

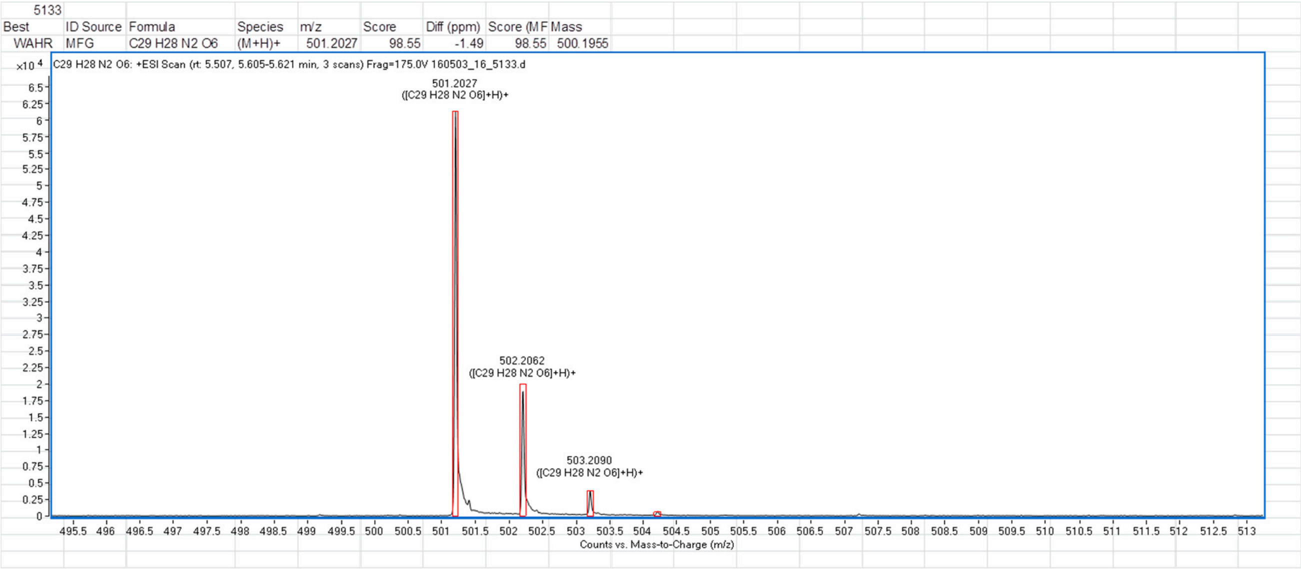

IC_{50}

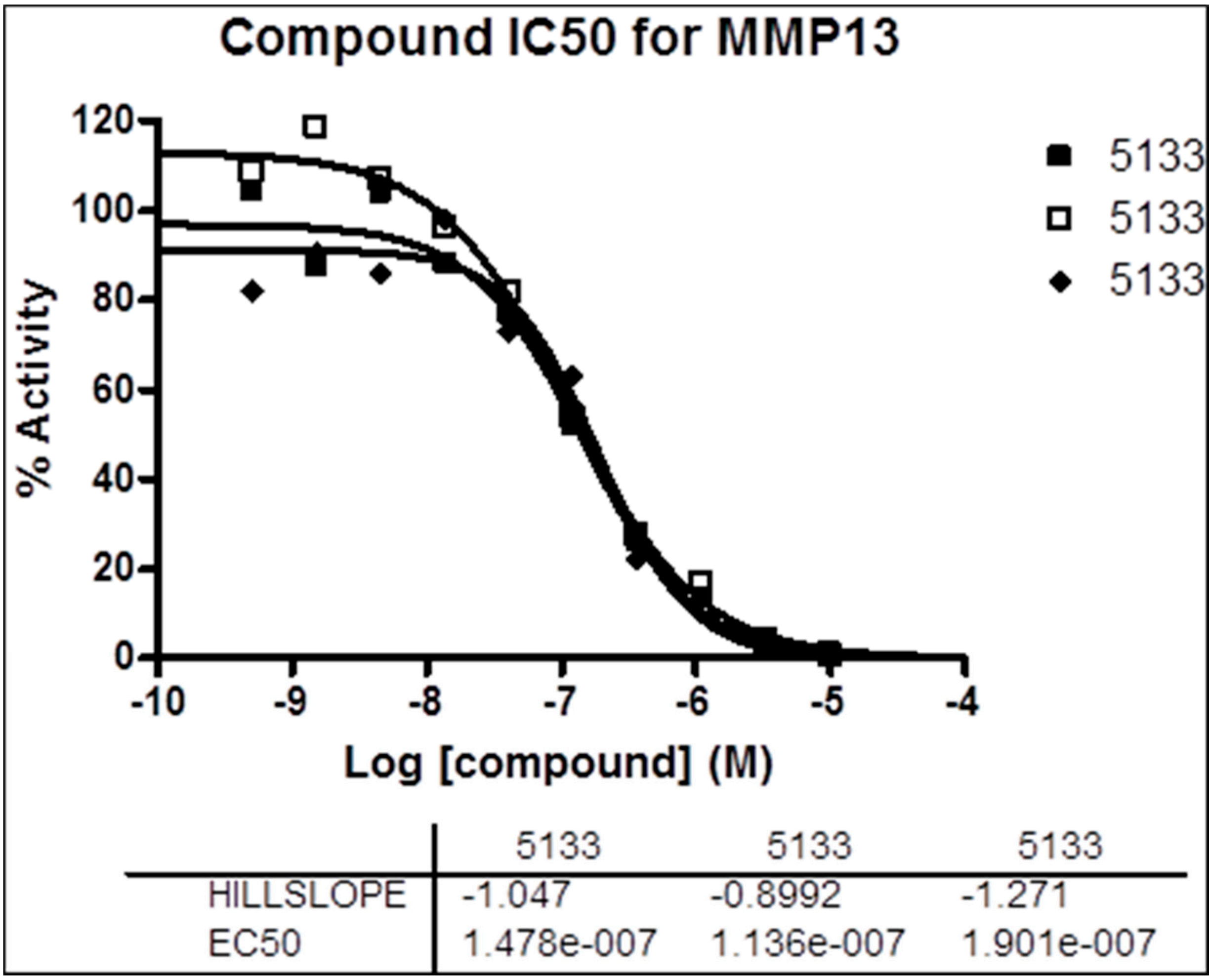

7-(4-{[(2-benzyl-1,3-dioxo-2,3-dihydro-1H-isoindol-5-yl)carbamoyl]methyl}phenoxy)heptanoic acid (**14g**; ZHAWOC6650)

<u>NMR</u>

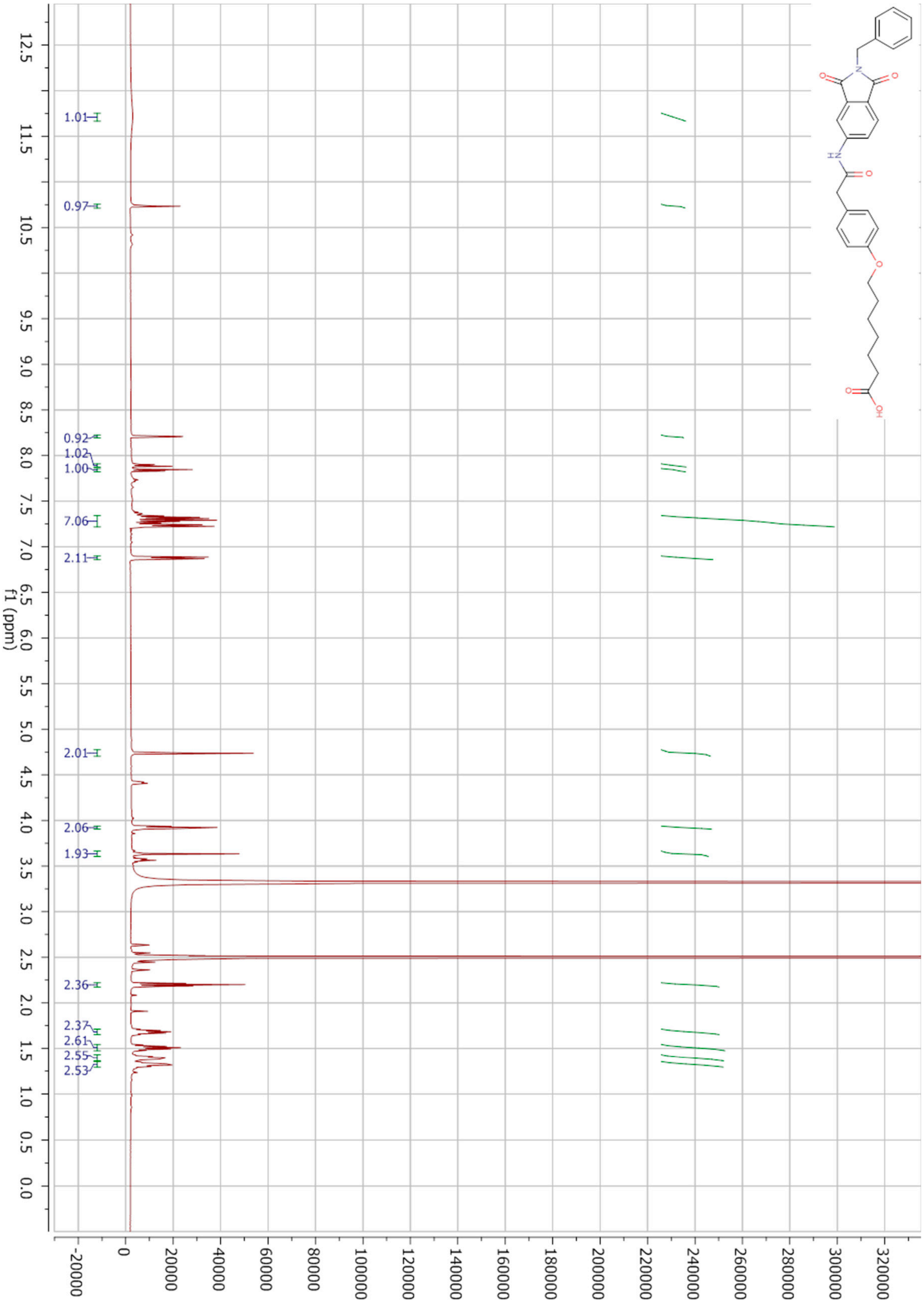

7-(4-{[(2-benzyl-1,3-dioxo-2,3-dihydro-1H-isoindol-5-yl)carbamoyl]methyl}phenoxy)heptanoic acid (**14g**; ZHAWOC6650)

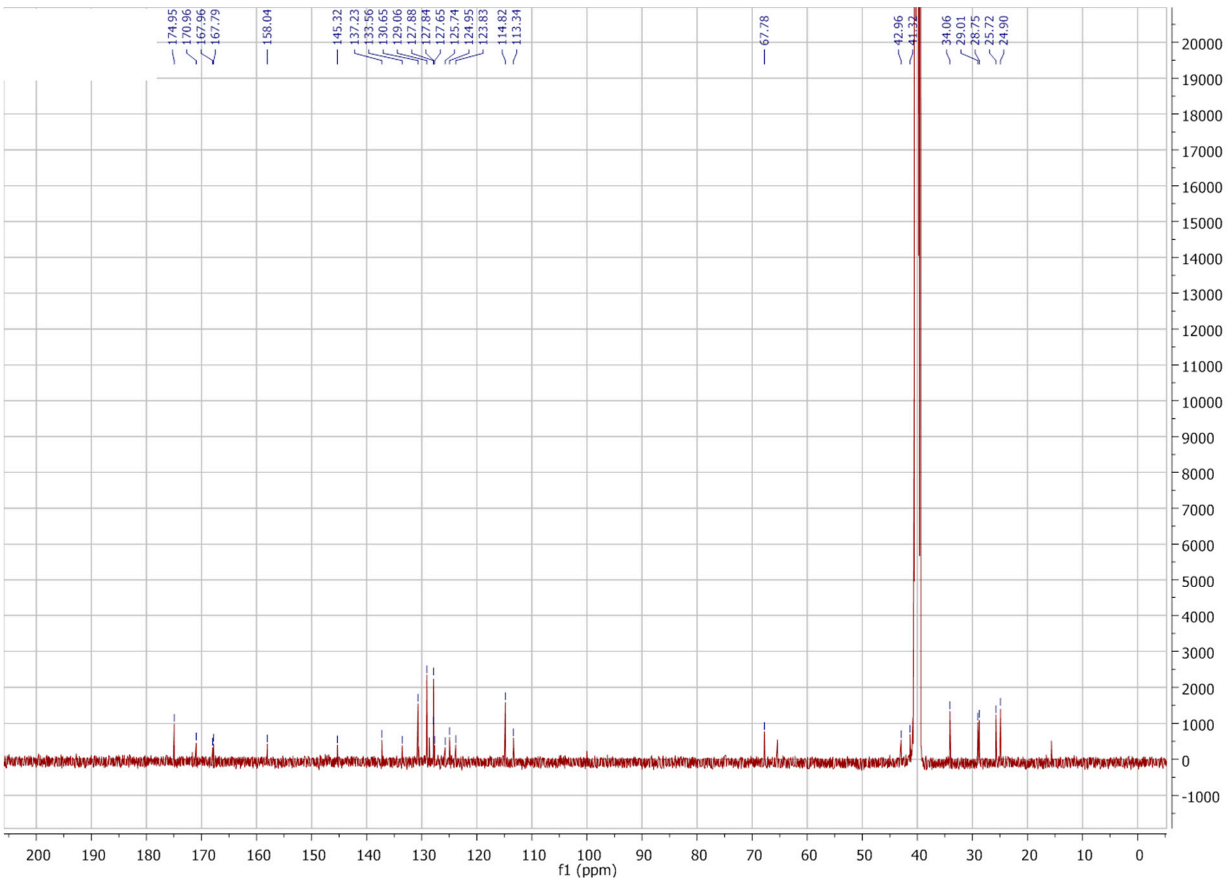

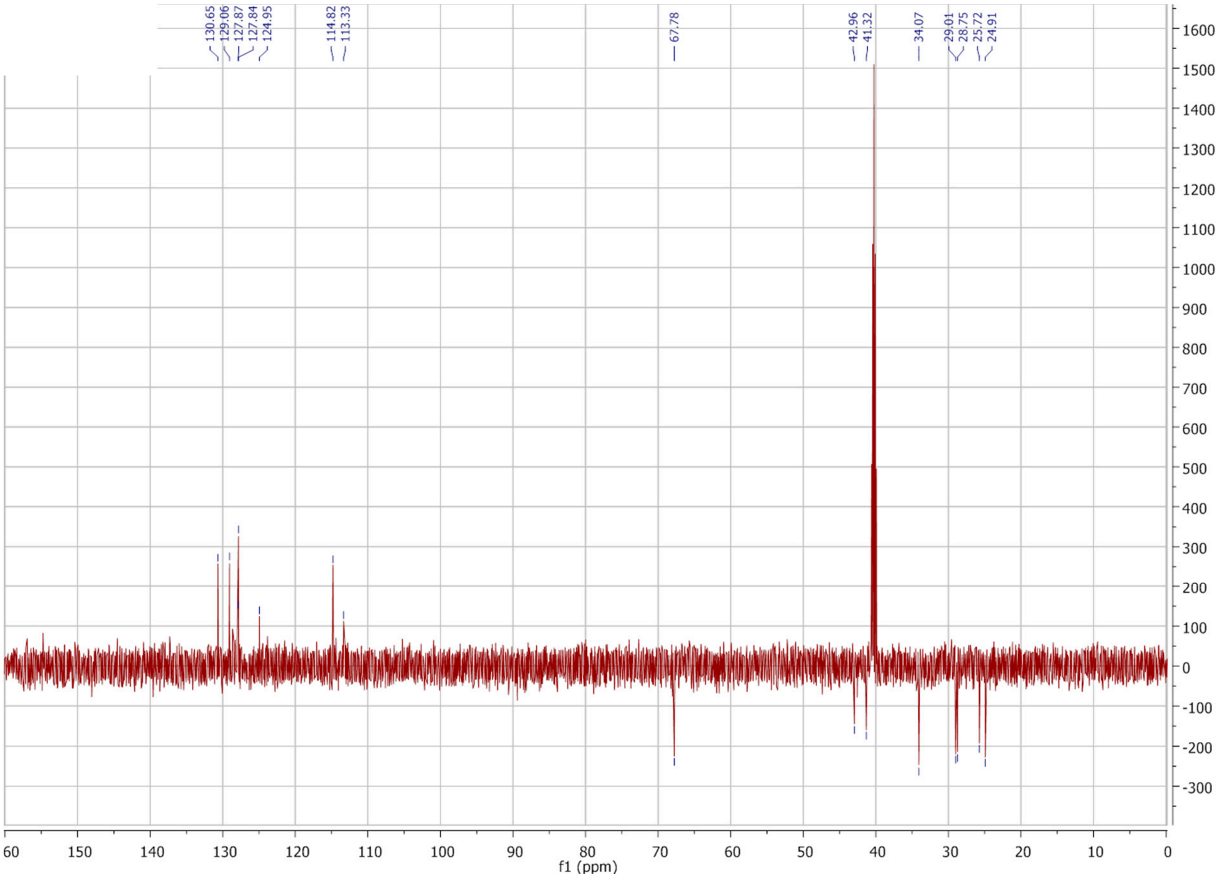

7-(4-{[(2-benzyl-1,3-dioxo-2,3-dihydro-1H-isoindol-5-yl)carbamoyl]methyl}phenoxy)heptanoic acid (**14g**; ZHAWOC6650)

<u>HRMS</u>

Best	Formula	Species	m/z	Diff (ppm)	Score (MFG)
WAHR	C30 H30 N	(M+H)+	515.2175	0.15	99.13

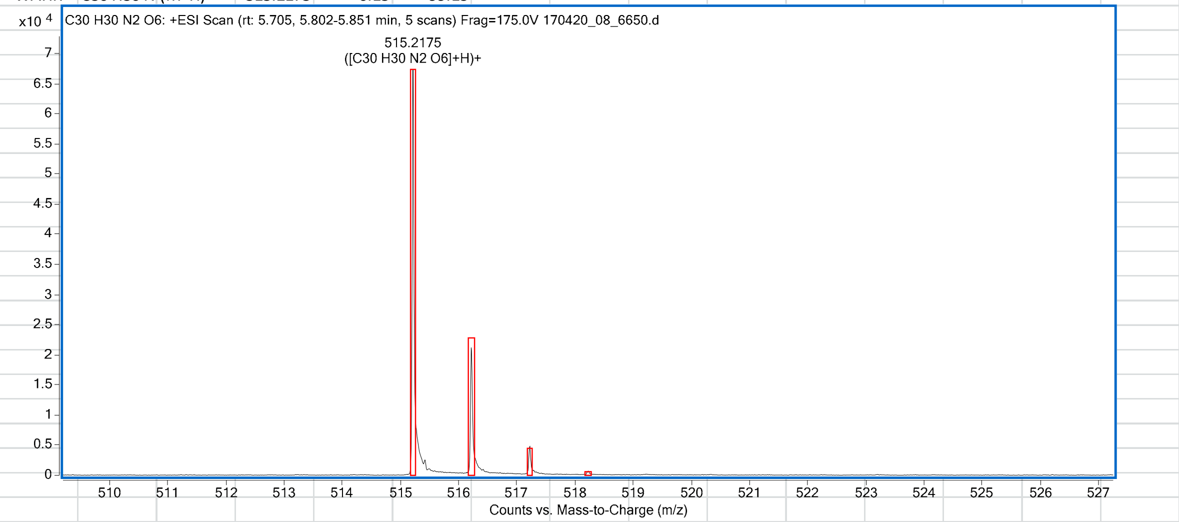

<u>IC$_{50}$</u>

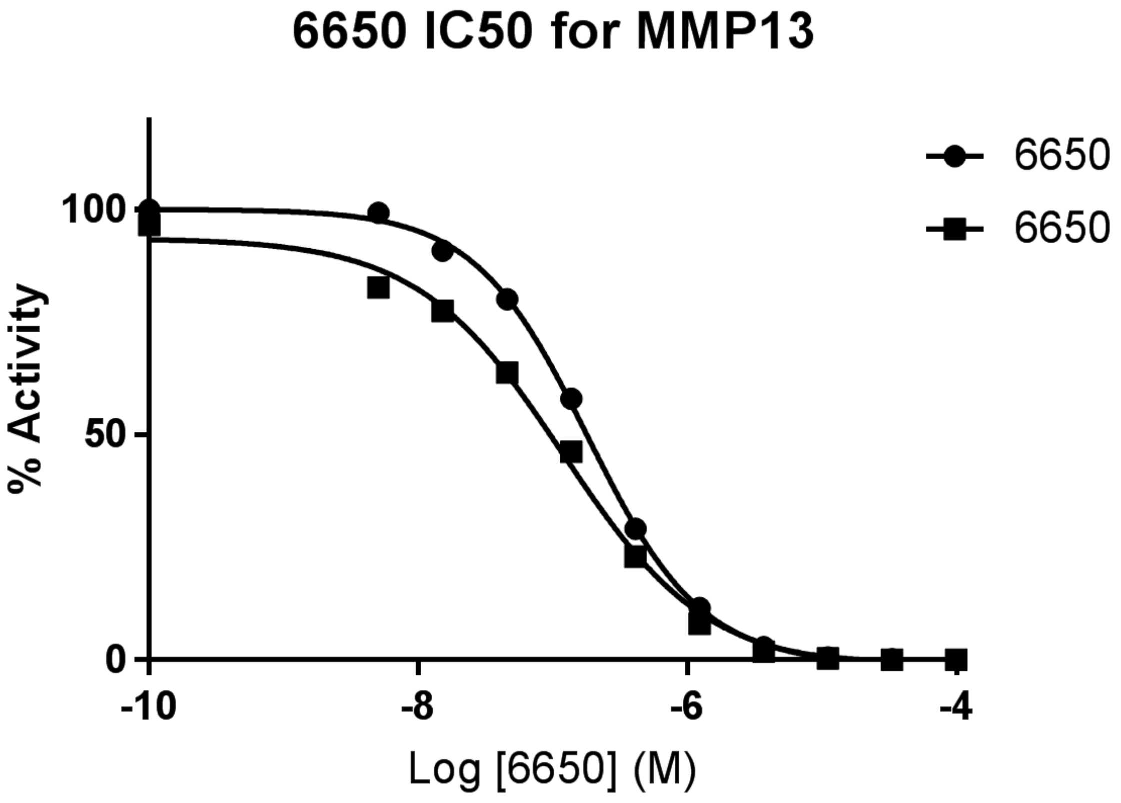

8-(4-{[(2-benzyl-1,3-dioxo-2,3-dihydro-1H-isoindol-5-yl)carbamoyl]methyl}phenoxy)octanoic acid (**14h**; ZHAWOC6651)

<u>NMR</u>

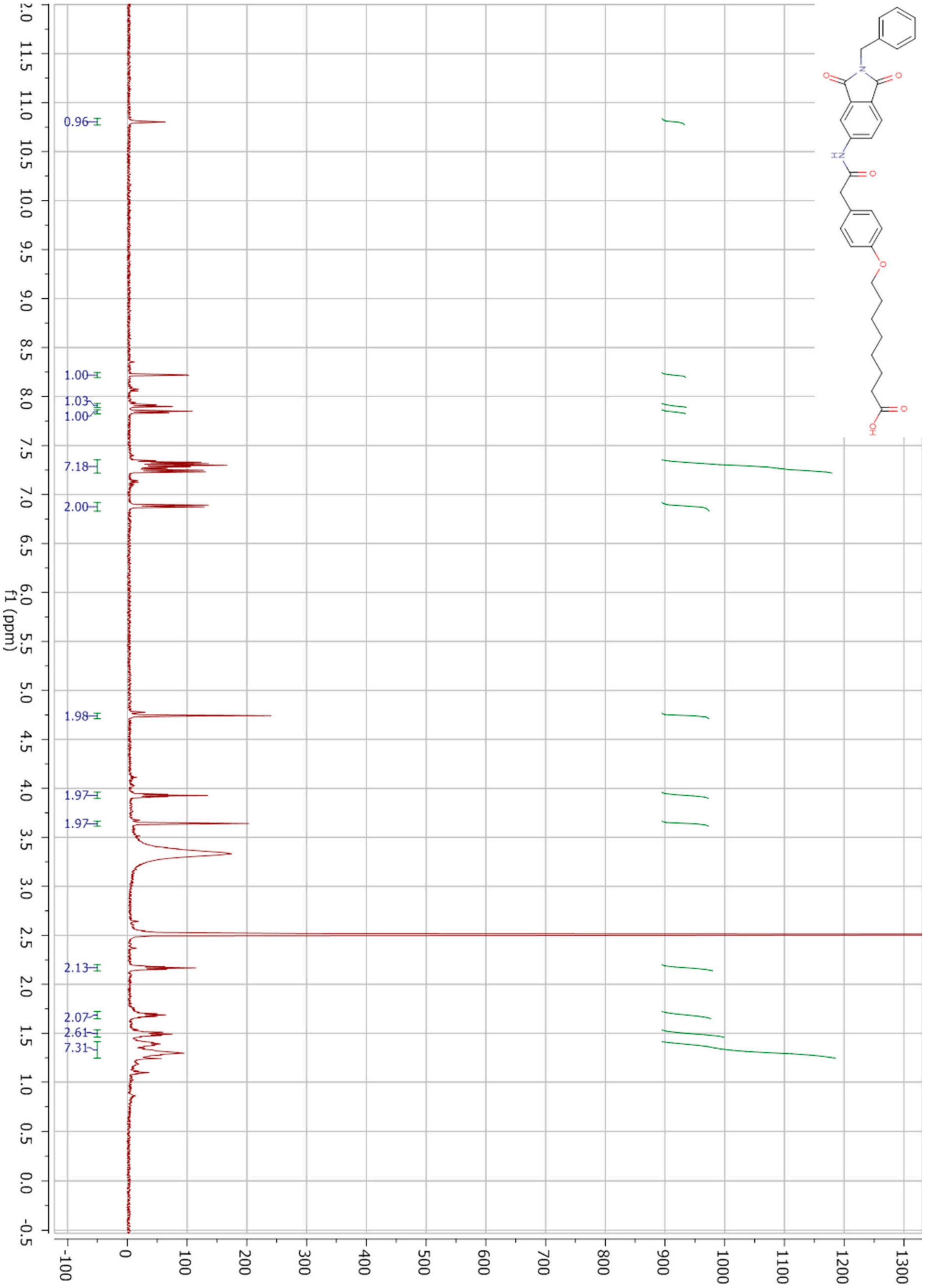

8-(4-{[(2-benzyl-1,3-dioxo-2,3-dihydro-1H-isoindol-5-yl)carbamoyl]methyl}phenoxy)octanoic acid (**14h**; ZHAWOC6651)

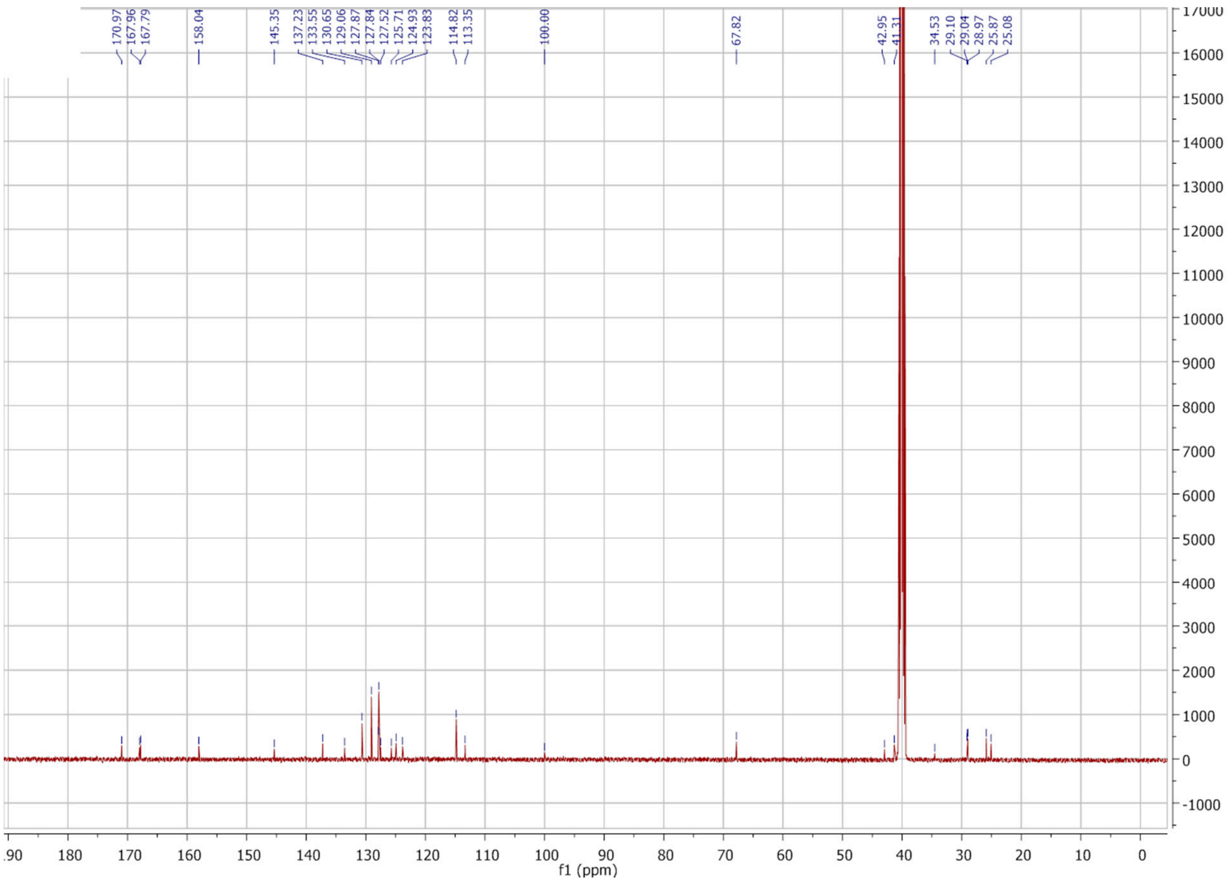

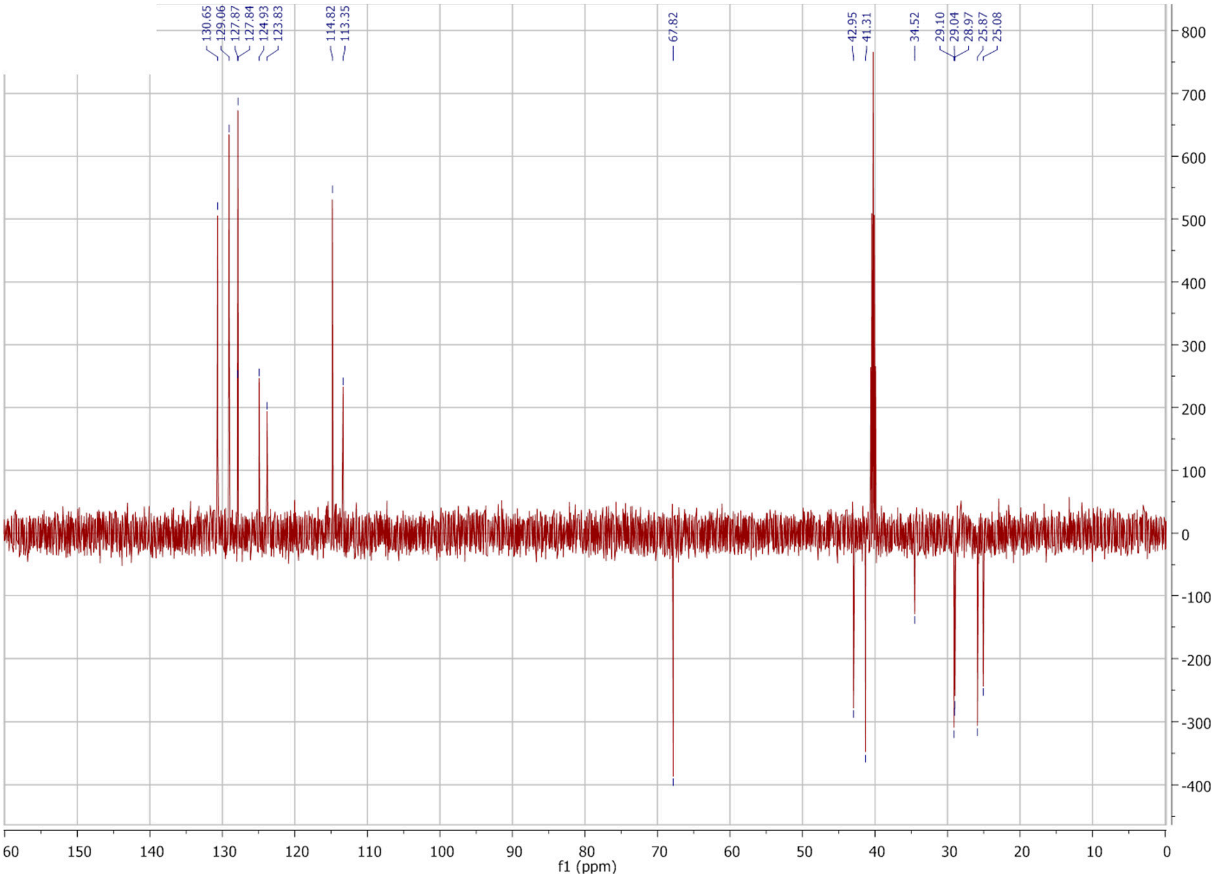

8-(4-{[(2-benzyl-1,3-dioxo-2,3-dihydro-1H-isoindol-5-yl)carbamoyl]methyl}phenoxy)octanoic acid (**14h**; ZHAWOC6651)

HRMS

Best	Formula	Species	m/z	Diff (ppm)	Score (MFG)
WAHR	C31 H32 N	(M+H)+	529.2324	1.57	97.86

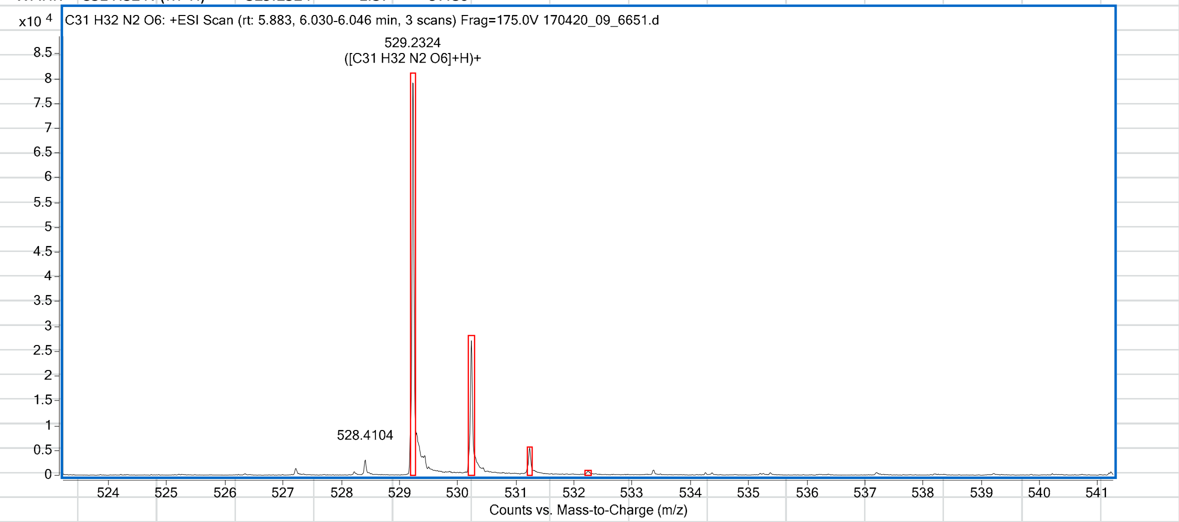

IC50

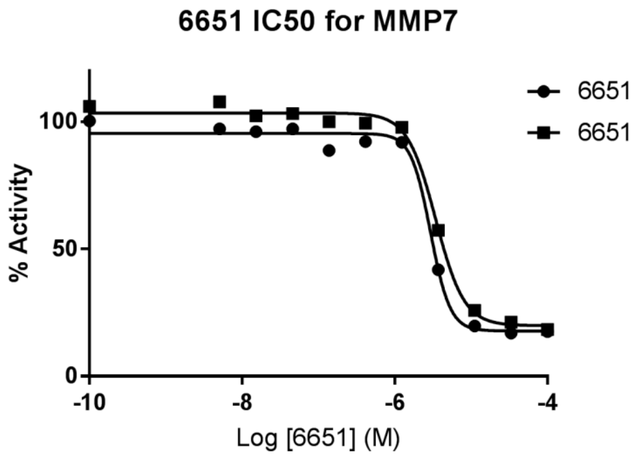

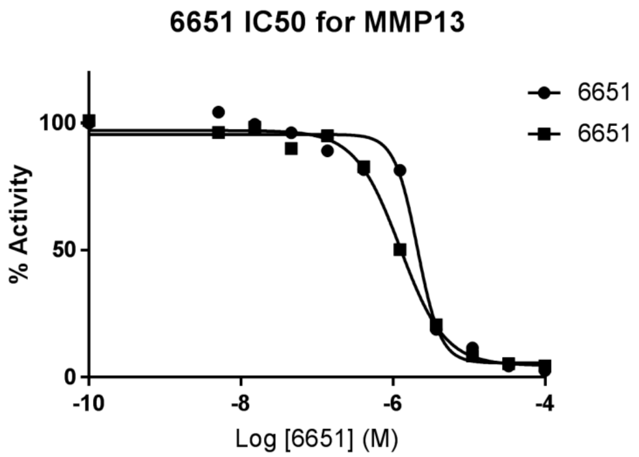

9-(4-{[(2-benzyl-1,3-dioxo-2,3-dihydro-1H-isoindol-5-yl)carbamoyl]methyl}phenoxy)nonanoic acid (**14i**; ZHAWOC6941)

<u>NMR</u>

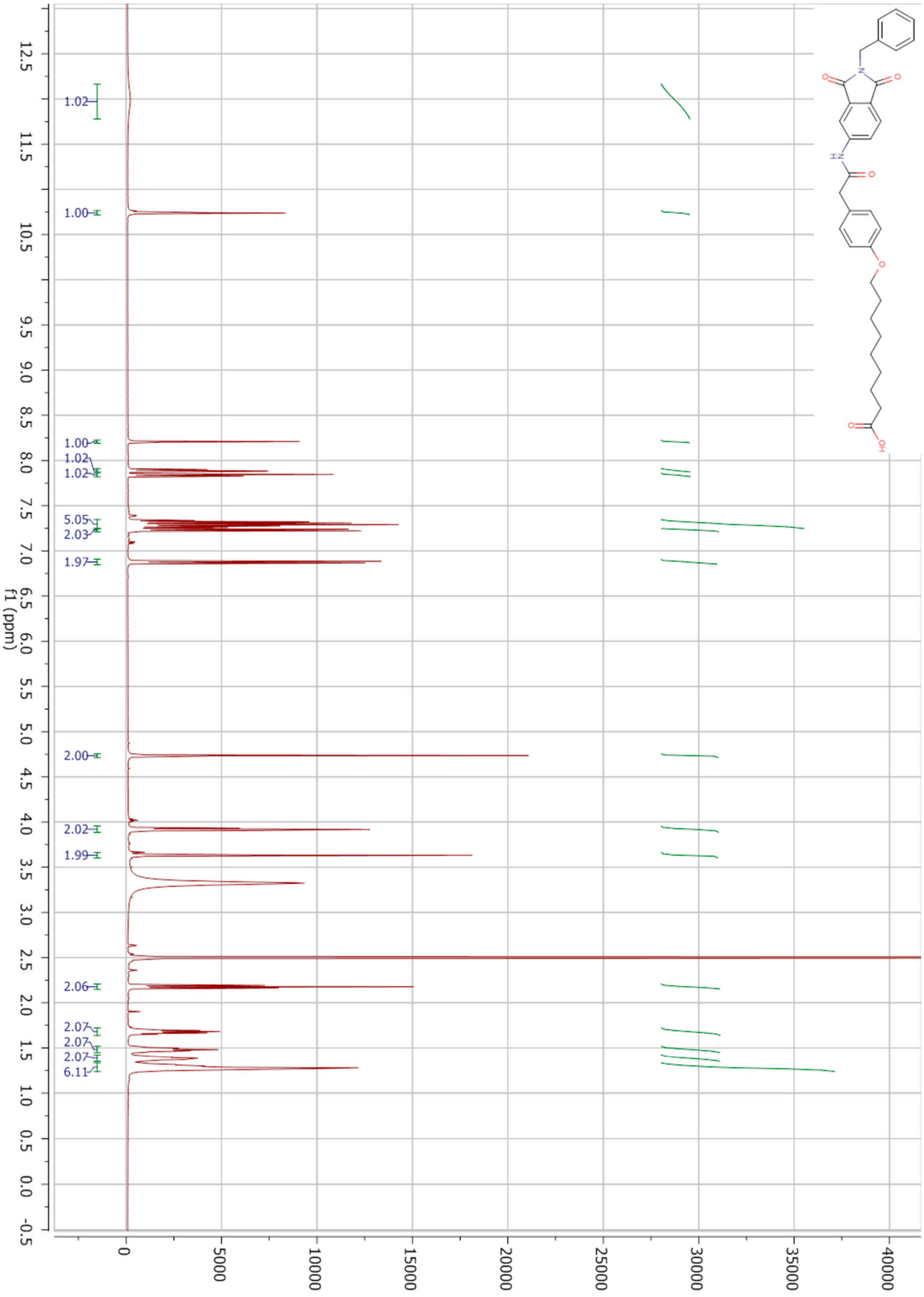

9-(4-{[(2-benzyl-1,3-dioxo-2,3-dihydro-1H-isoindol-5-yl)carbamoyl]methyl}phenoxy)nonanoic acid (**14i**; ZHAWOC6941)

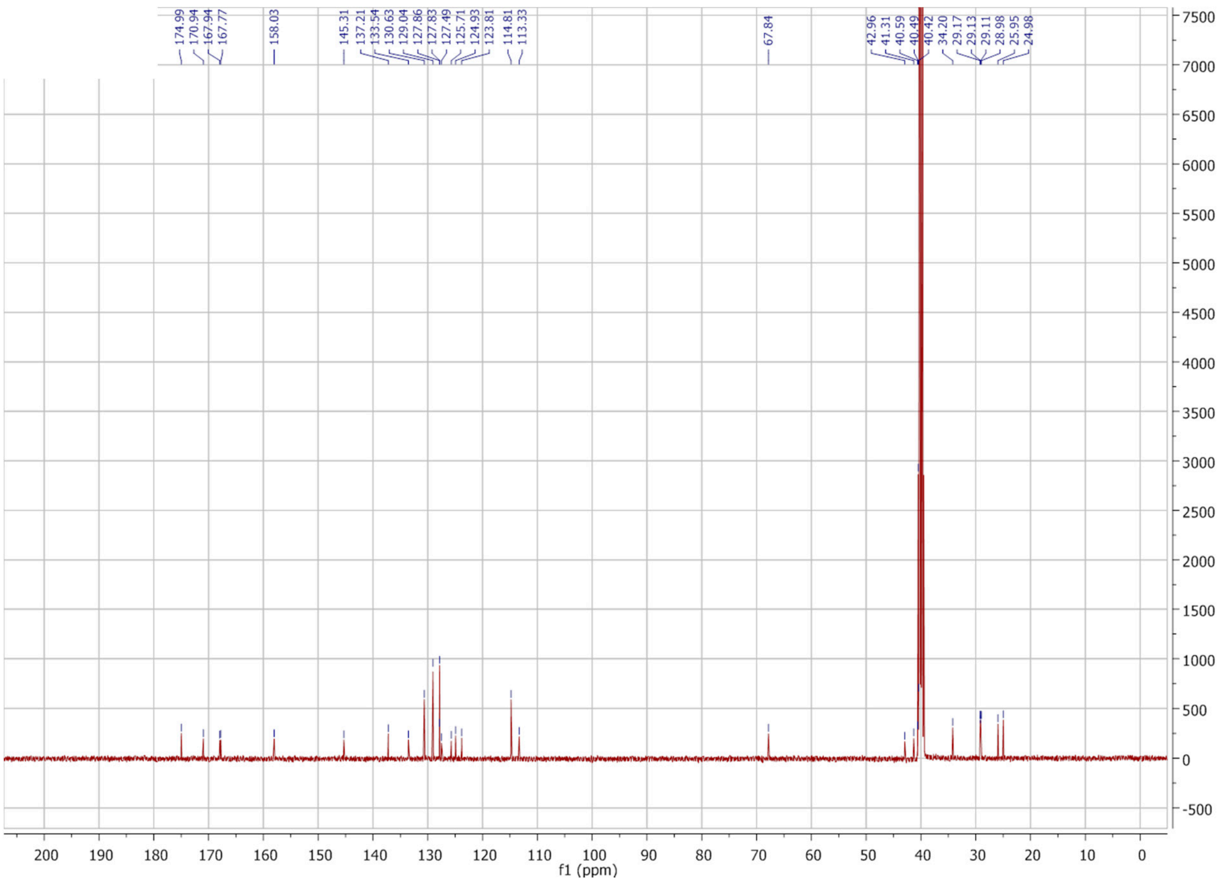

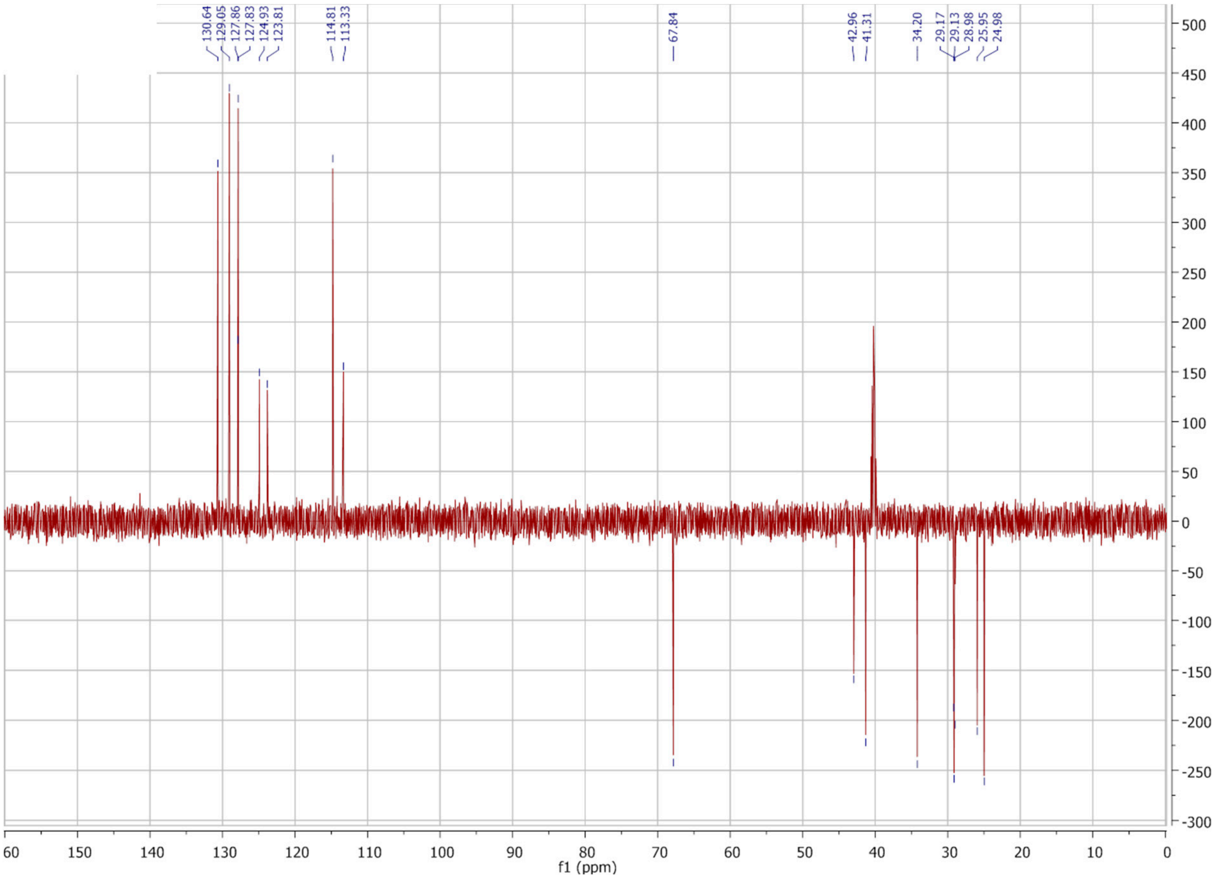

9-(4-{[(2-benzyl-1,3-dioxo-2,3-dihydro-1H-isoindol-5-yl)carbamoyl]methyl}phenoxy)nonanoic acid (**14i**; ZHAWOC6941)

HRMS

Best	Formula	Species	m/z	Diff (ppm	Score (MFG)
WAHR	C32 H34 N	(M+H)+	543.2485	0.8	99.56

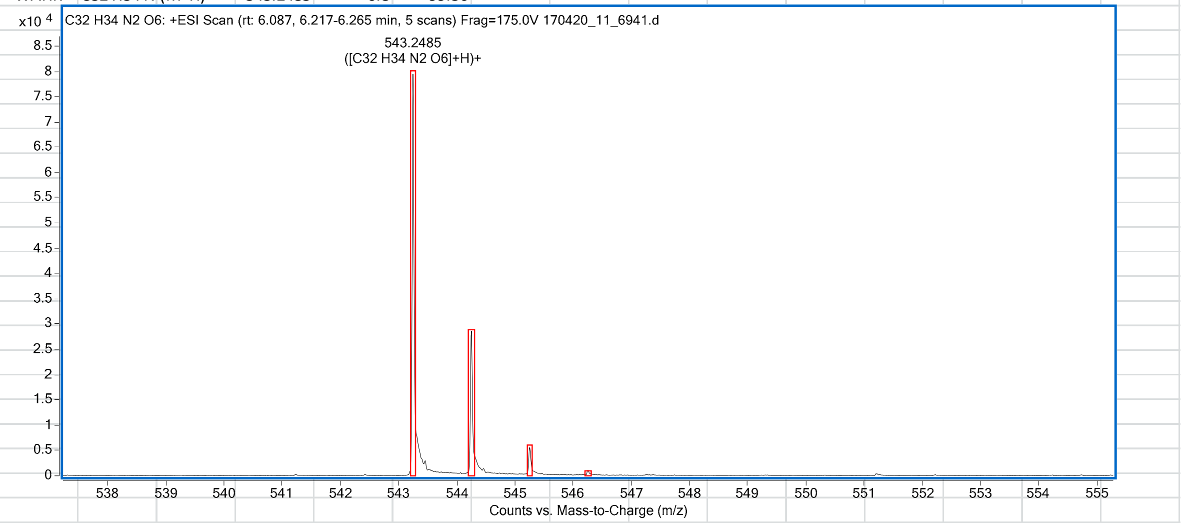

IC$_{50}$

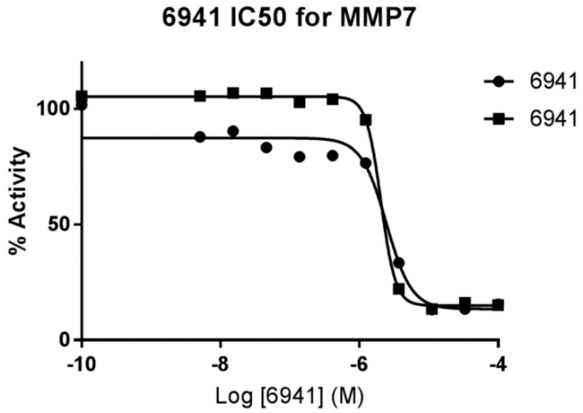

Figure S1a: Determination of the IC$_{50}$-value for compound **14i** against MMP-7.

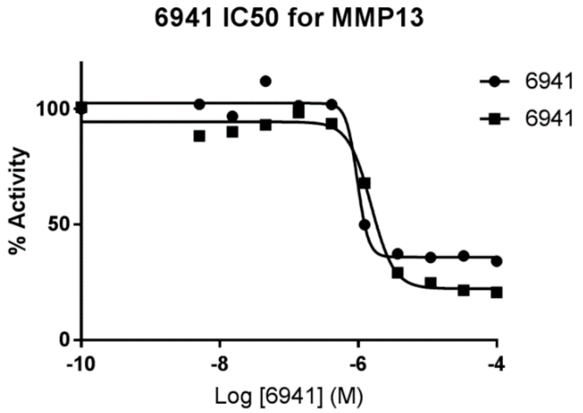

Figure S1b: Determination of the IC$_{50}$-value for compound **14i** against MMP-13.

10-(4-{[(2-benzyl-1,3-dioxo-2,3-dihydro-1H-isoindol-5-yl)carbamoyl]methyl}phenoxy)decanoic acid (**14j**; ZHAWOC6942)

<u>NMR</u>

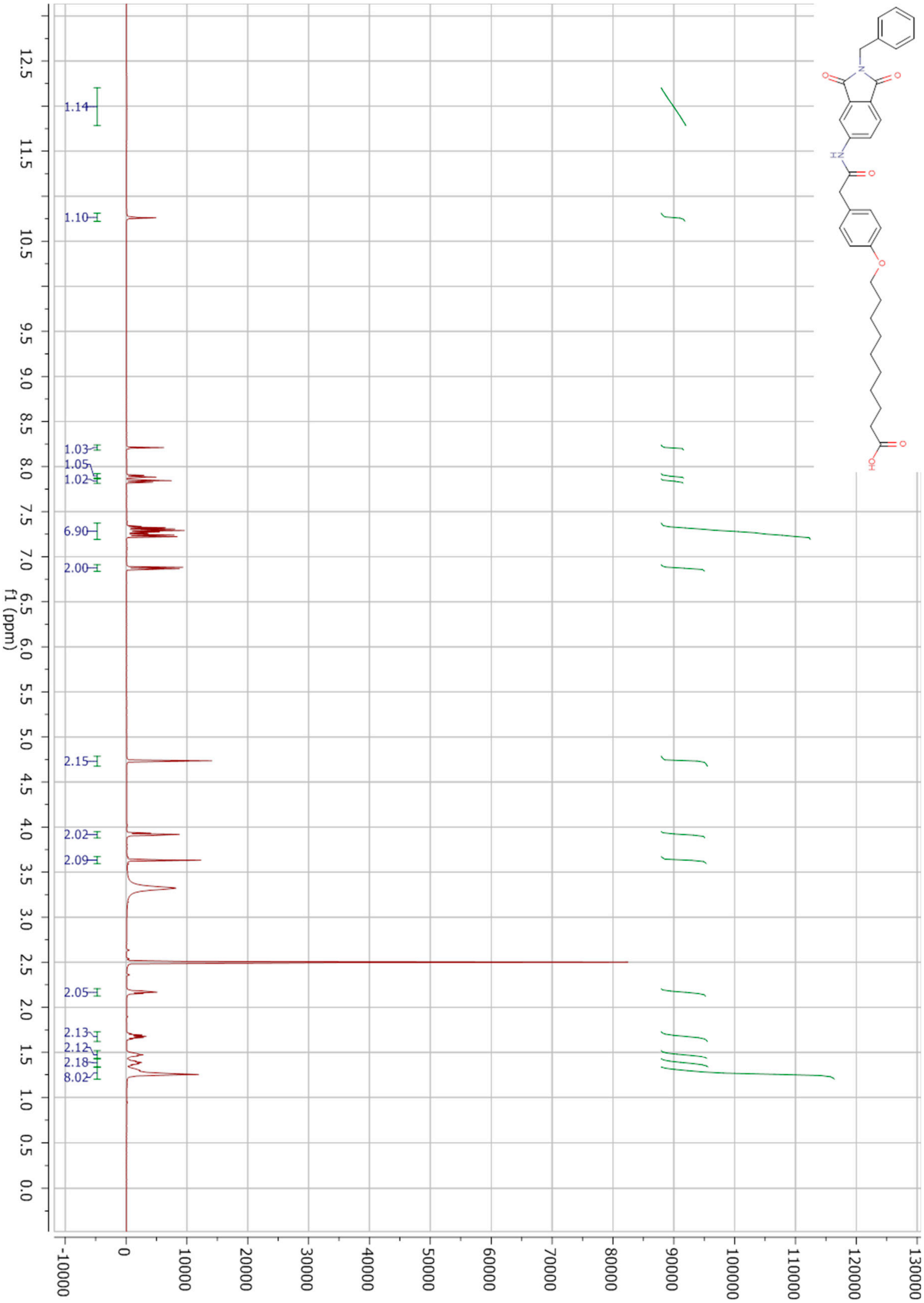

10-(4-{[(2-benzyl-1,3-dioxo-2,3-dihydro-1H-isoindol-5-yl)carbamoyl]methyl}phenoxy)decanoic acid (**14j**; ZHAWOC6942)

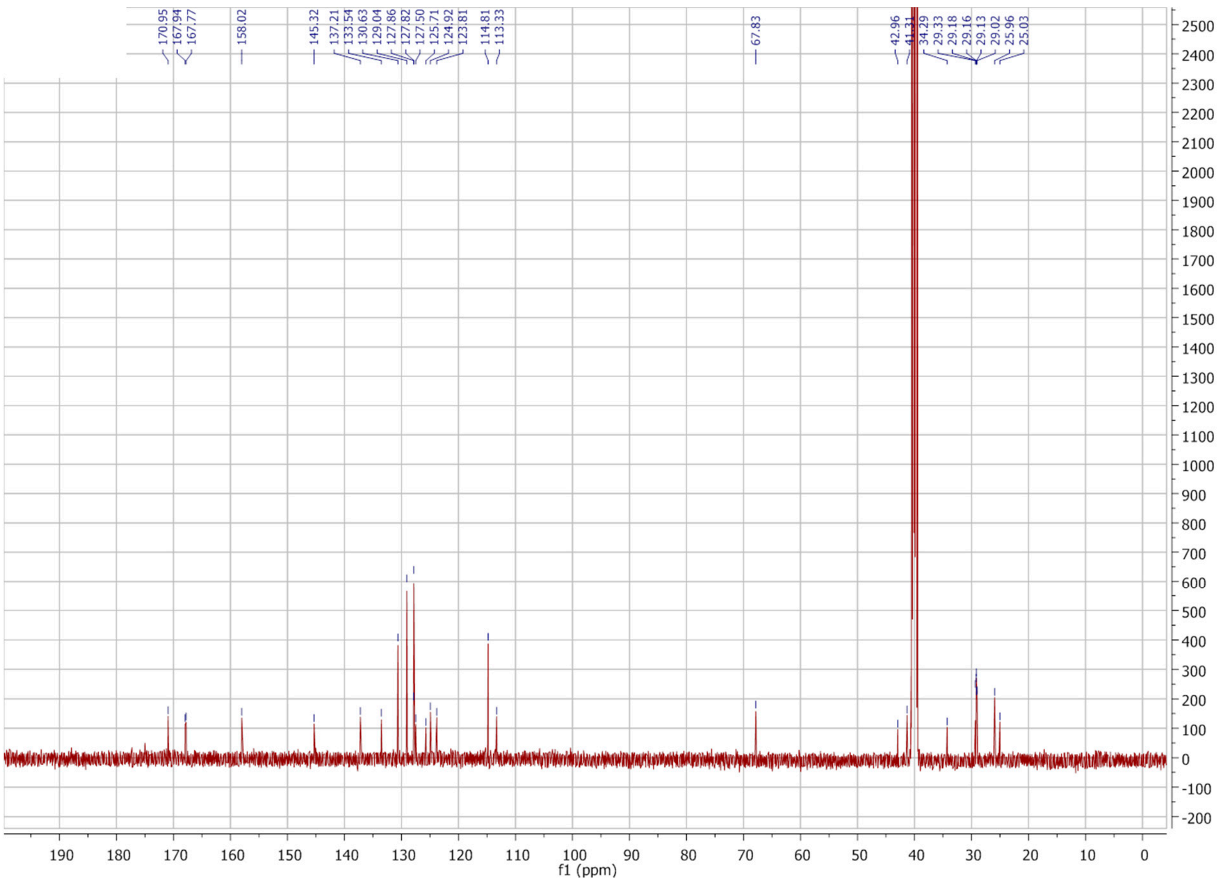

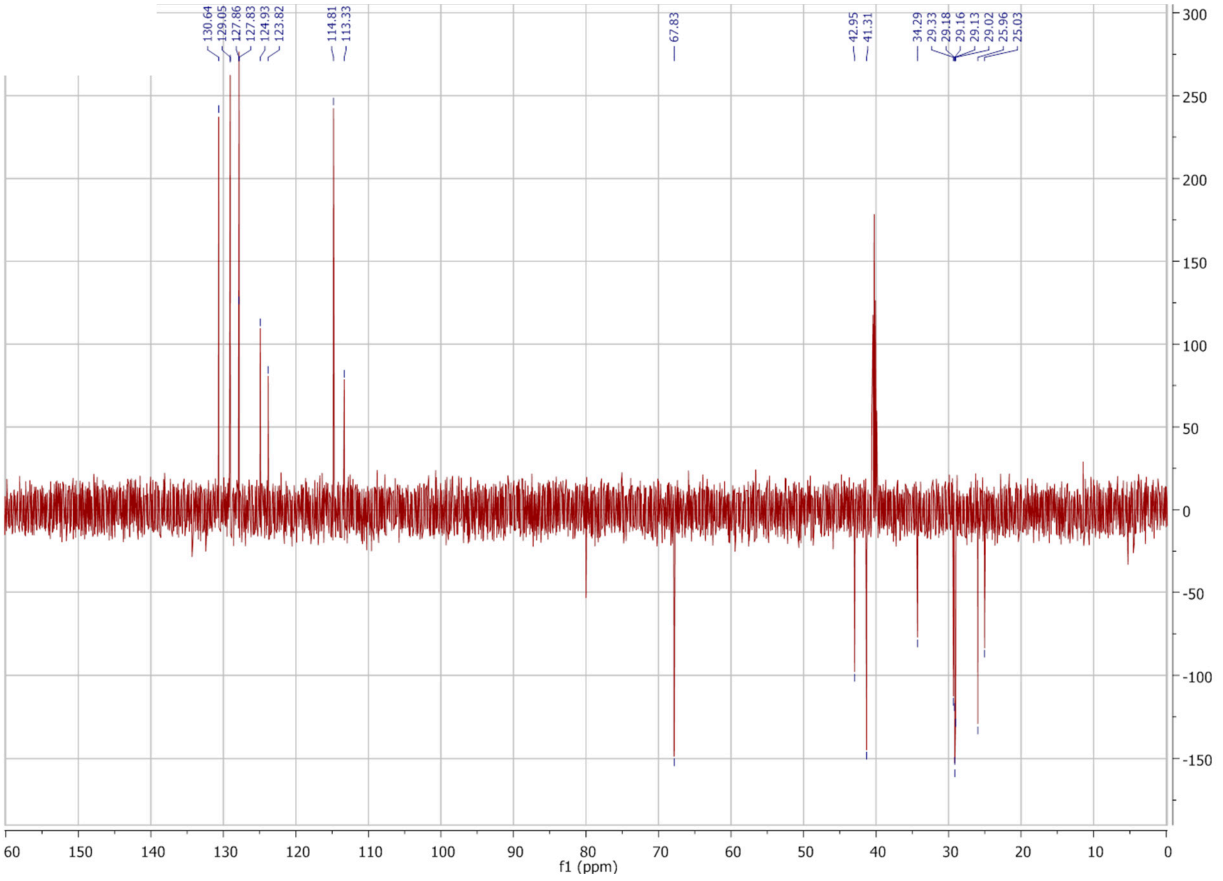

10-(4-{[(2-benzyl-1,3-dioxo-2,3-dihydro-1H-isoindol-5-yl)carbamoyl]methyl}phenoxy)decanoic acid (**14j**; ZHAWOC6942)

HRMS

Best	Formula	Species	m/z	Diff (ppm)	Score (MFG)
WAHR	C33 H36 N	(M+H)+	557.2647	-0.02	99.21

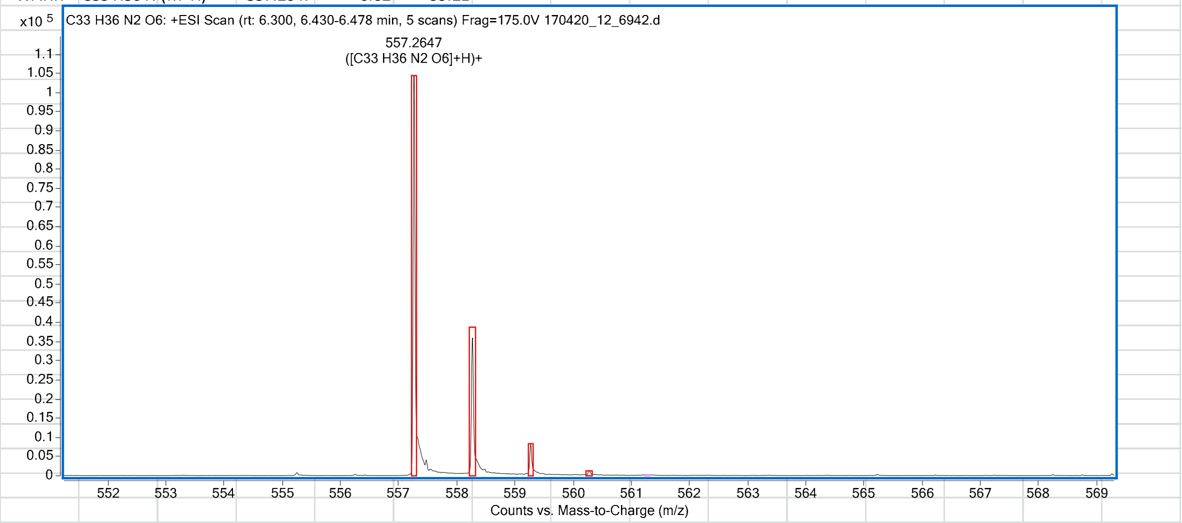

IC50

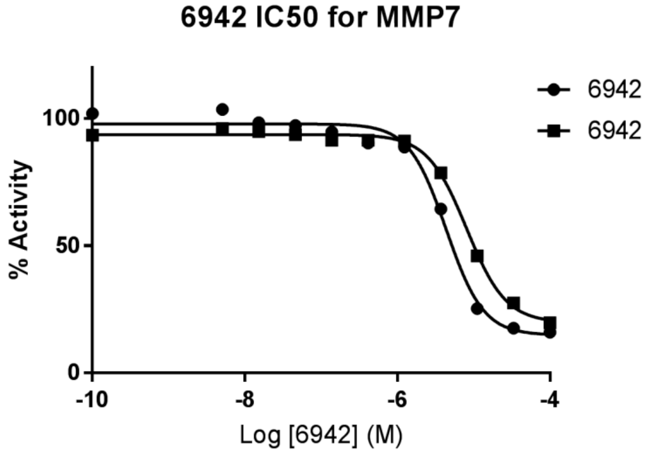

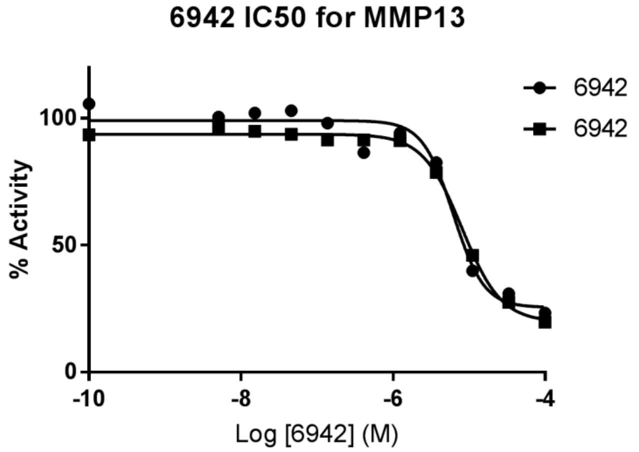

3-{4-[({2-[(4-fluorophenyl)methyl]-1,3-dioxo-2,3-dihydro-1H-isoindol-5-yl}carbamoyl)methyl]phenoxy}propanoic acid (**14k**; ZHAWOC6644)

<u>NMR</u>

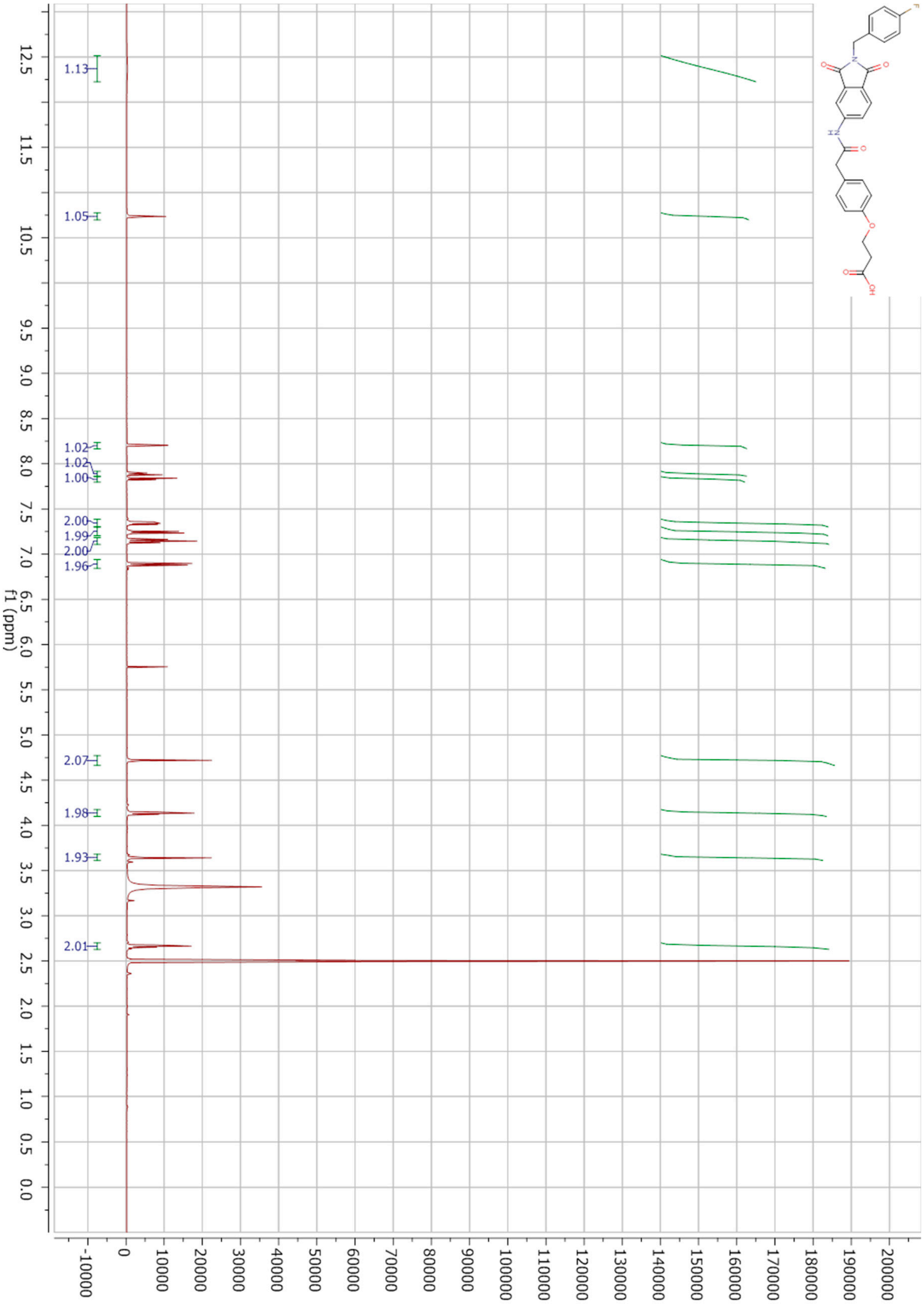

3-{4-[({2-[(4-fluorophenyl)methyl]-1,3-dioxo-2,3-dihydro-1H-isoindol-5-yl}carbamoyl)methyl]phenoxy}propanoic acid (**14k**; ZHAWOC6644)

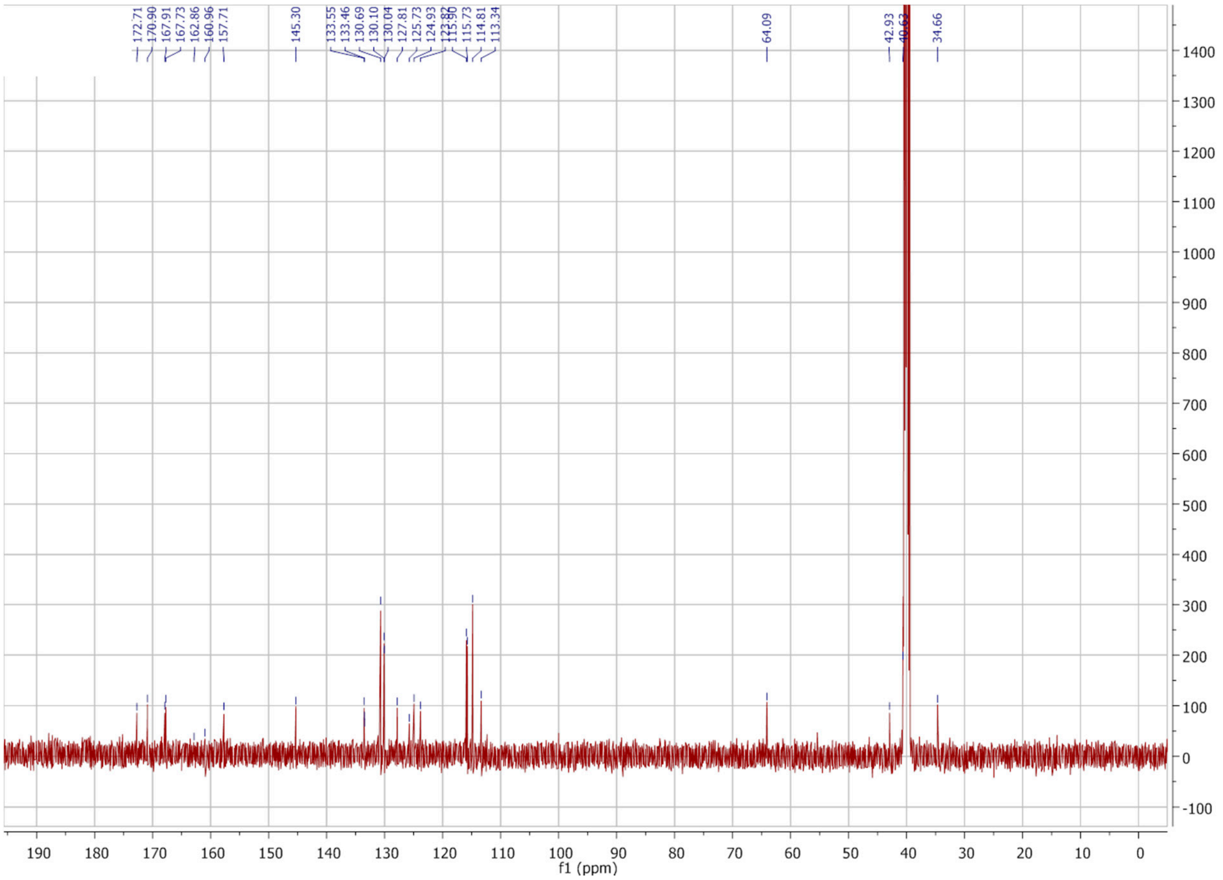

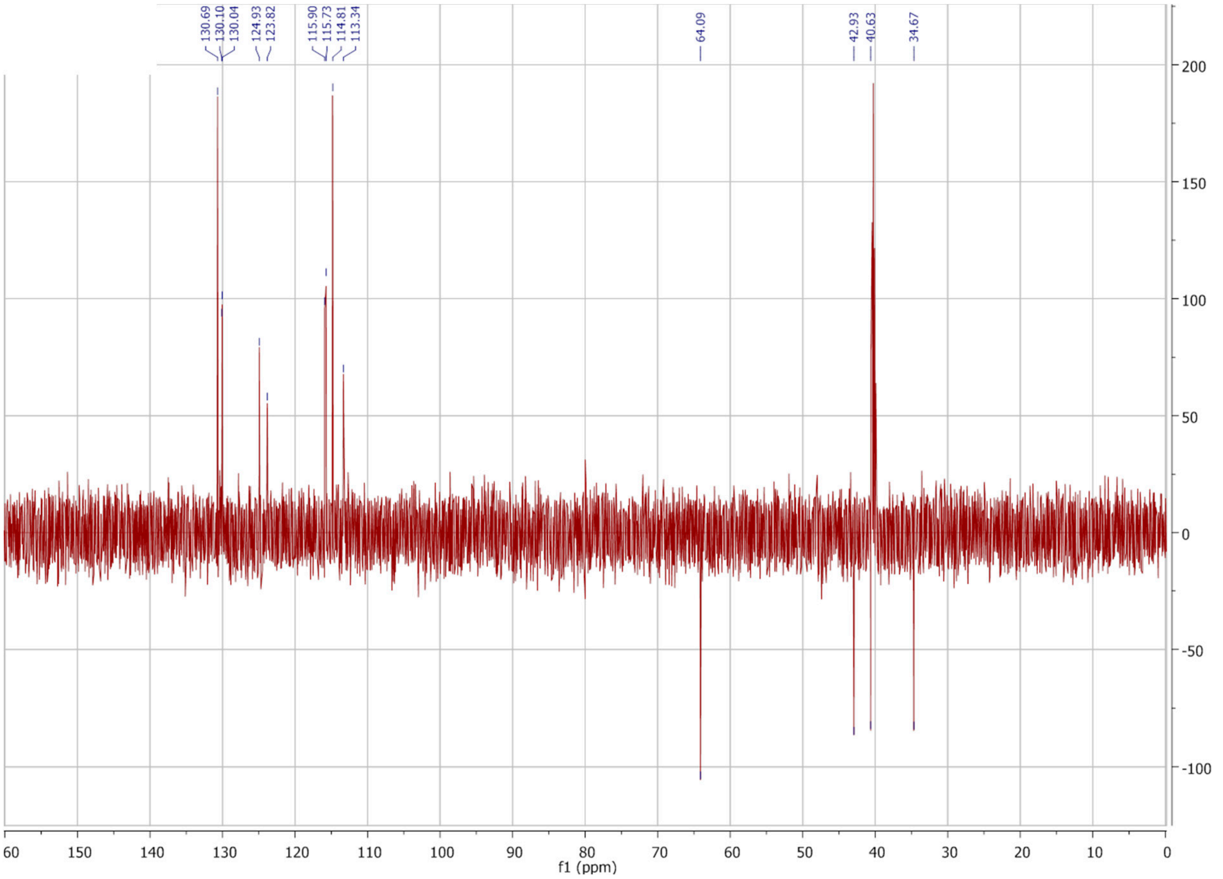

3-{4-[({2-[(4-fluorophenyl)methyl]-1,3-dioxo-2,3-dihydro-1H-isoindol-5-yl}carbamoyl)methyl]phenoxy}propanoic acid (**14k**; ZHAWOC6644)

<u>HRMS</u>

Best	Formula	Species	m/z	Diff (ppm)	Score (MFG)
WAHR	C26 H21 F	(M+H)+	477.147	-2.84	95.93

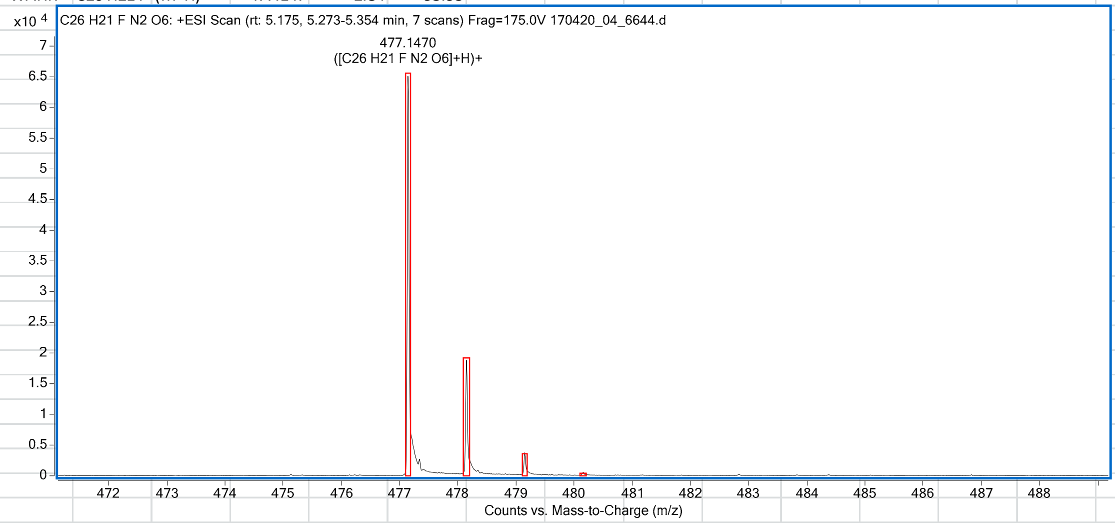

4-{4-[({2-[(4-fluorophenyl)methyl]-1,3-dioxo-2,3-dihydro-1H-isoindol-5-yl}carbamoyl)methyl]phenoxy}butanoic acid (**14l**; ZHAWOC5463)

<u>NMR</u>

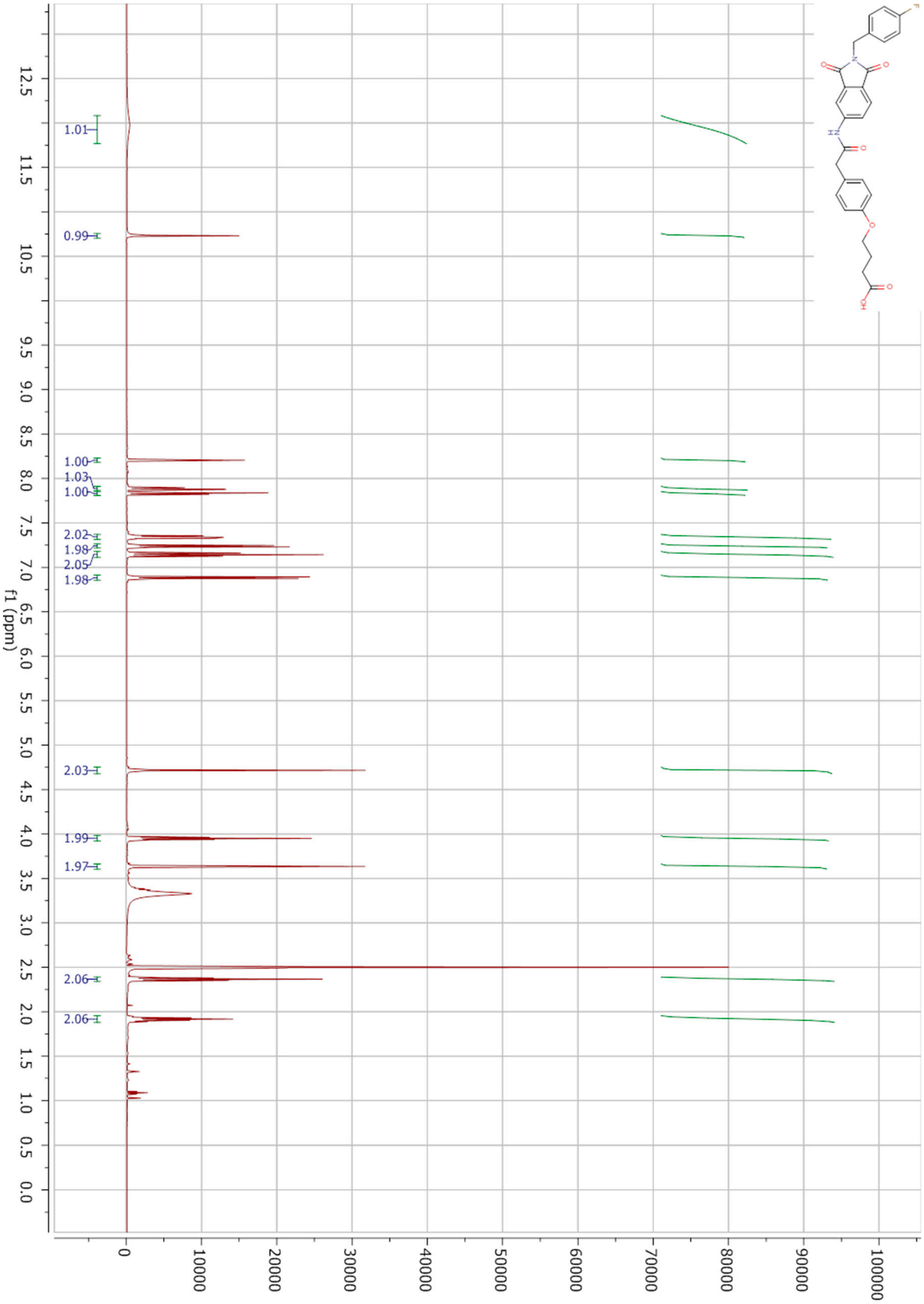

4-{4-[({2-[(4-fluorophenyl)methyl]-1,3-dioxo-2,3-dihydro-1H-isoindol-5-yl}carbamoyl)methyl]phenoxy}butanoic acid (**14l**; ZHAWOC5463)

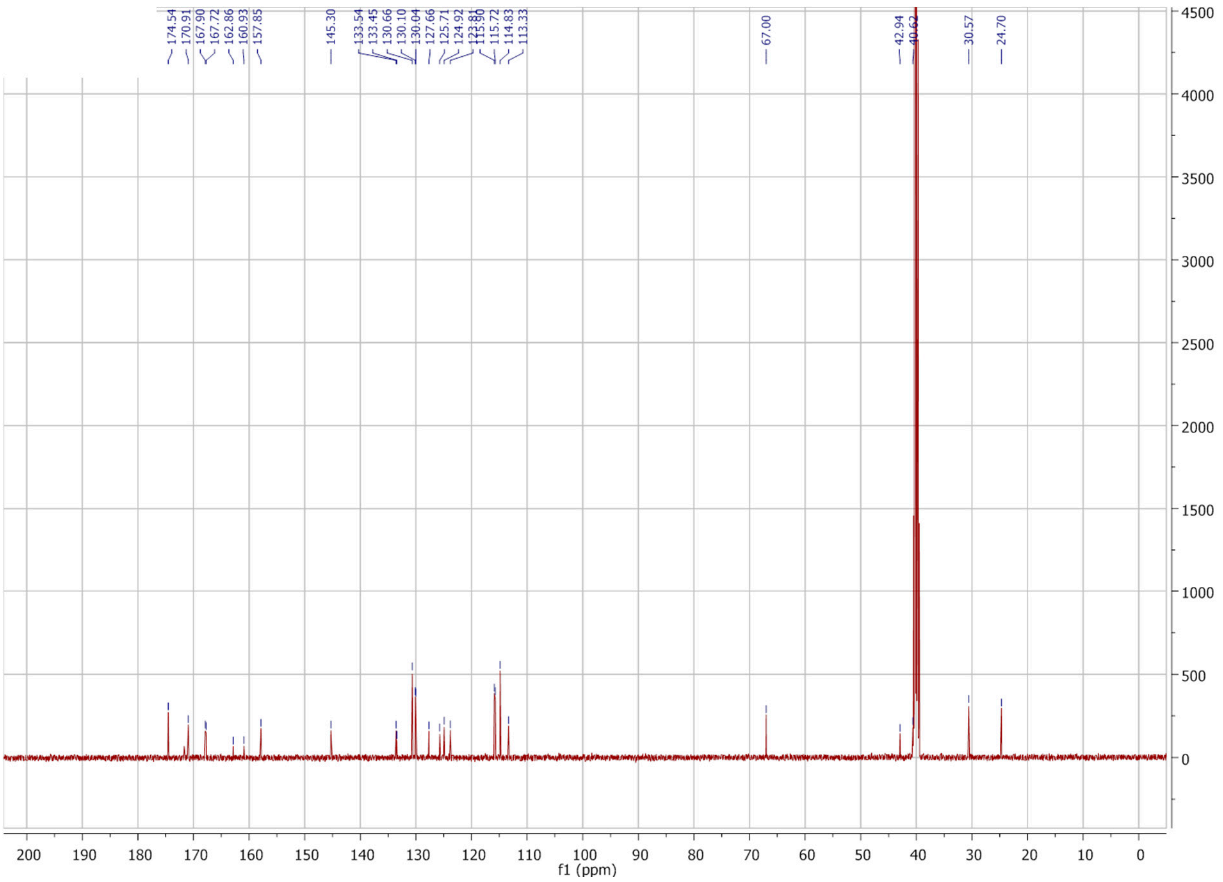

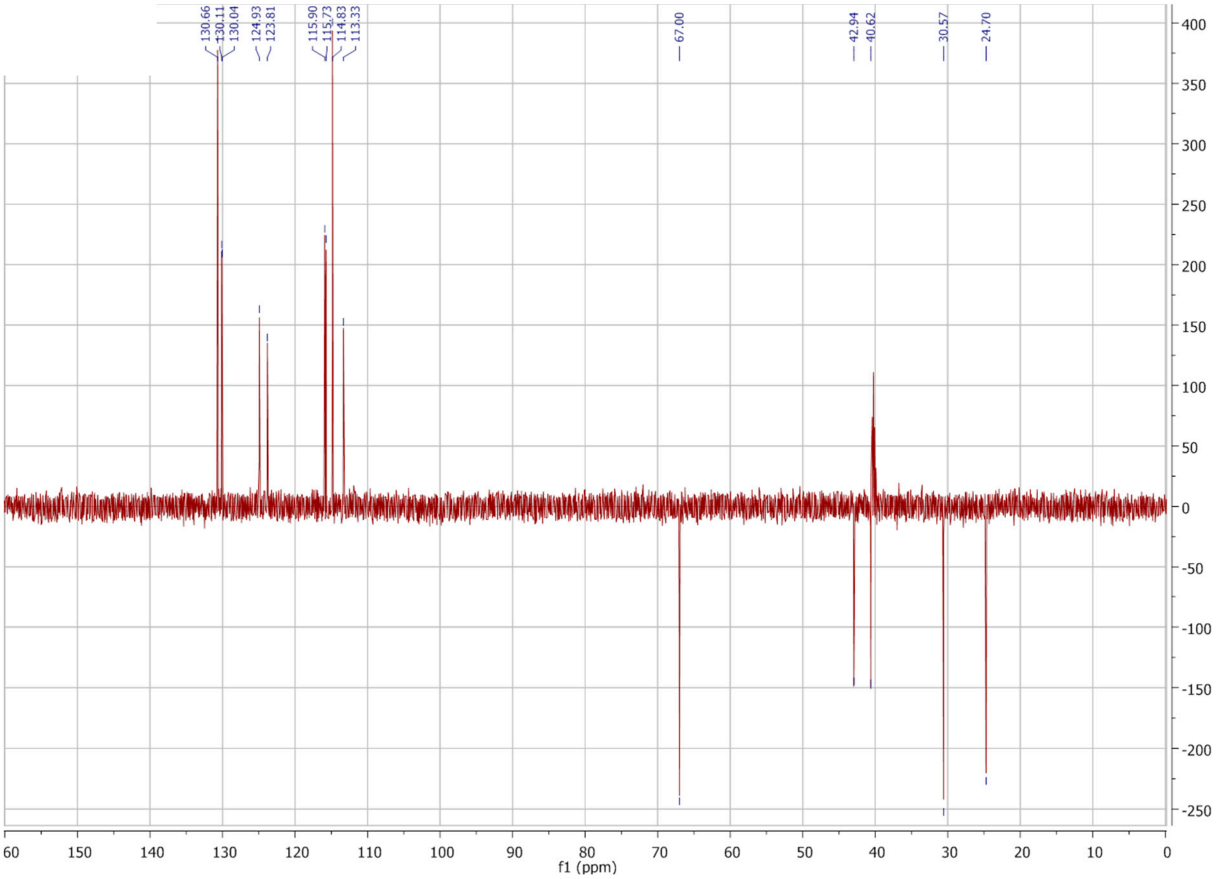

4-{4-[({2-[(4-fluorophenyl)methyl]-1,3-dioxo-2,3-dihydro-1H-isoindol-5-yl}carbamoyl)methyl]phenoxy}butanoic acid (**14l**; ZHAWOC5463)

HRMS

Best	Formula	Species	m/z	Diff (ppm)	Score (MFG)
WAHR	C27 H23 F	(M+H)+	491.1617	-0.77	99.11

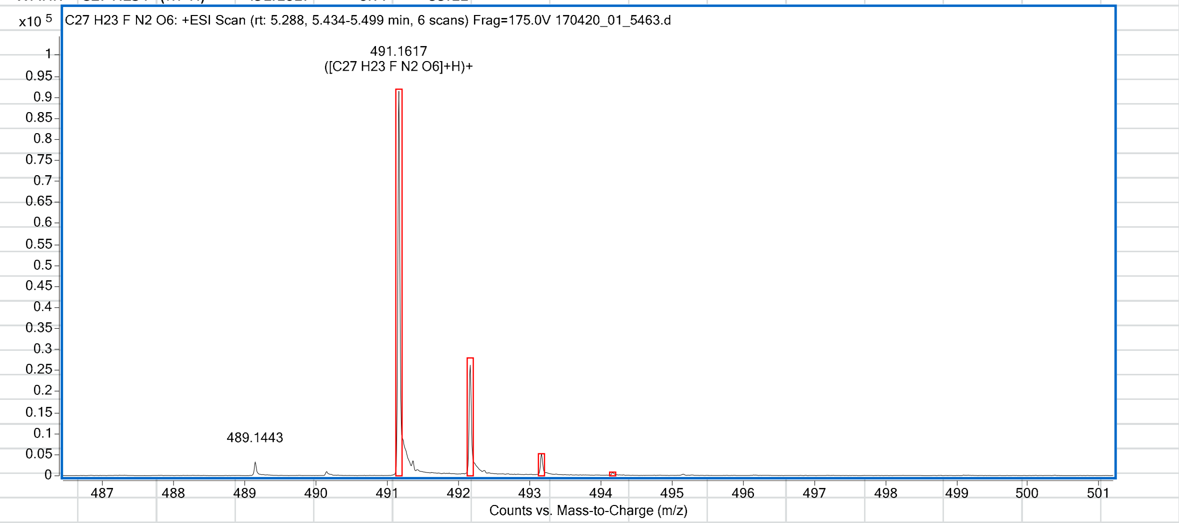

IC50

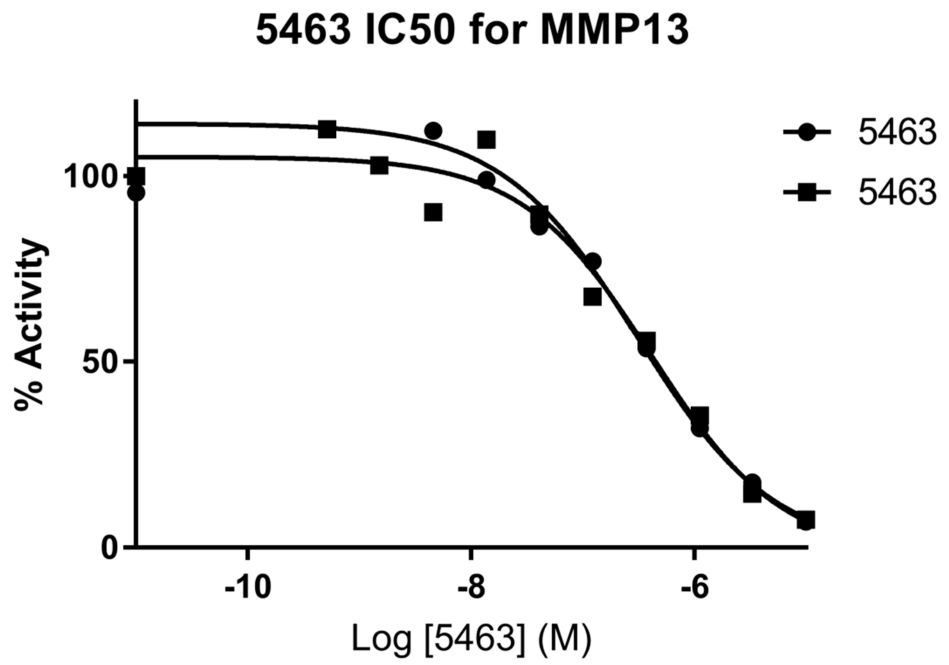

5-{4-[({2-[(4-fluorophenyl)methyl]-1,3-dioxo-2,3-dihydro-1H-isoindol-5-yl}carbamoyl)methyl]phenoxy}pentanoic acid (**5;** ZHAWOC5684)

<u>NMR</u>

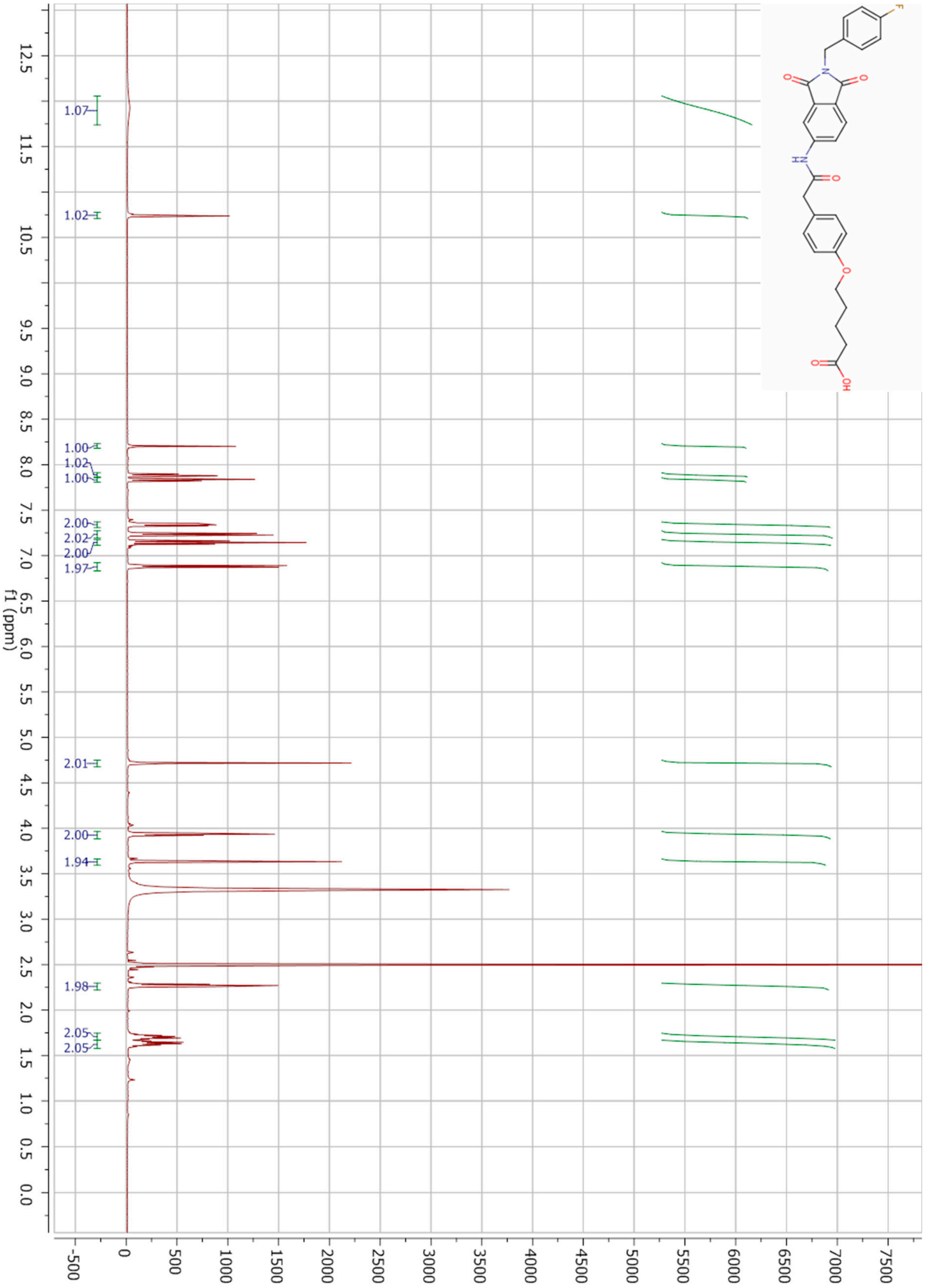

5-{4-[({2-[(4-fluorophenyl)methyl]-1,3-dioxo-2,3-dihydro-1H-isoindol-5-yl}carbamoyl)methyl]phenoxy}pentanoic acid (**5;** ZHAWOC5684)

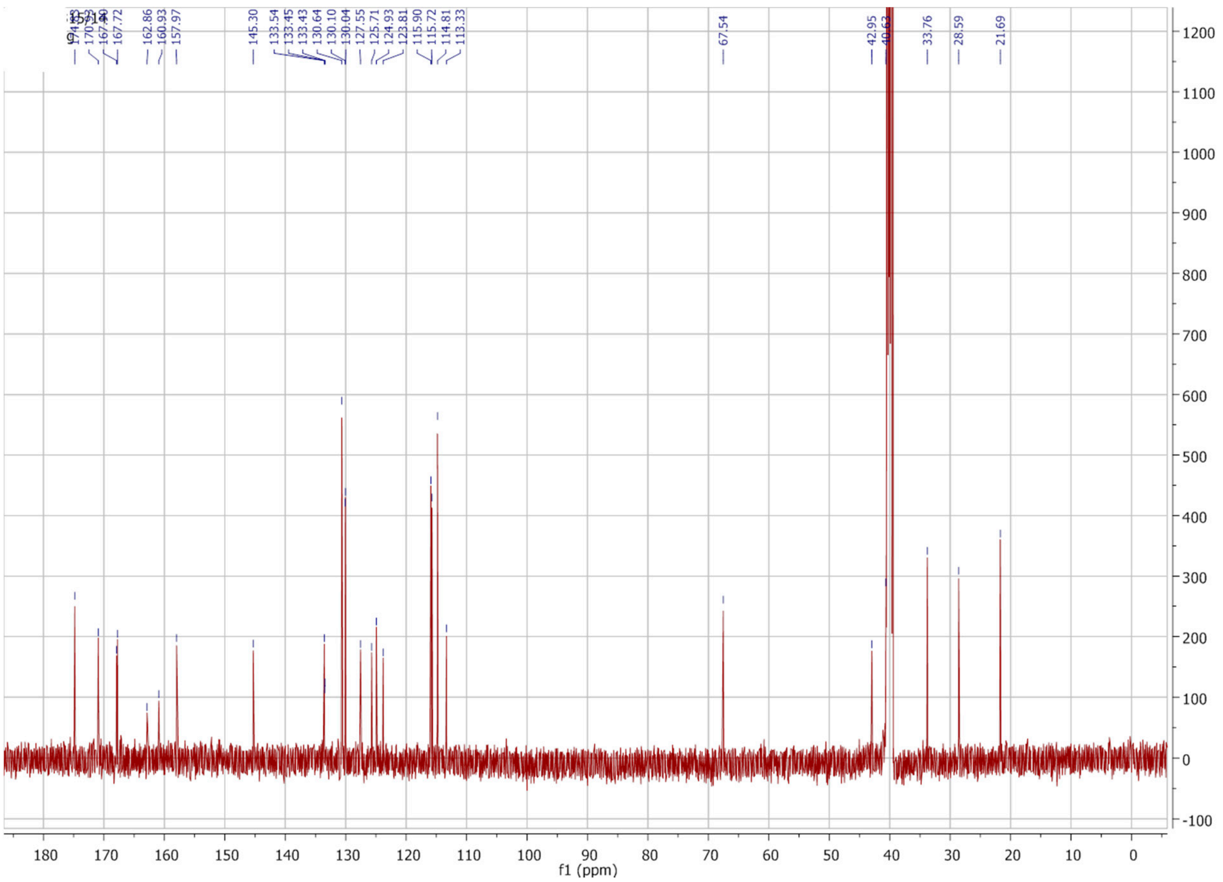

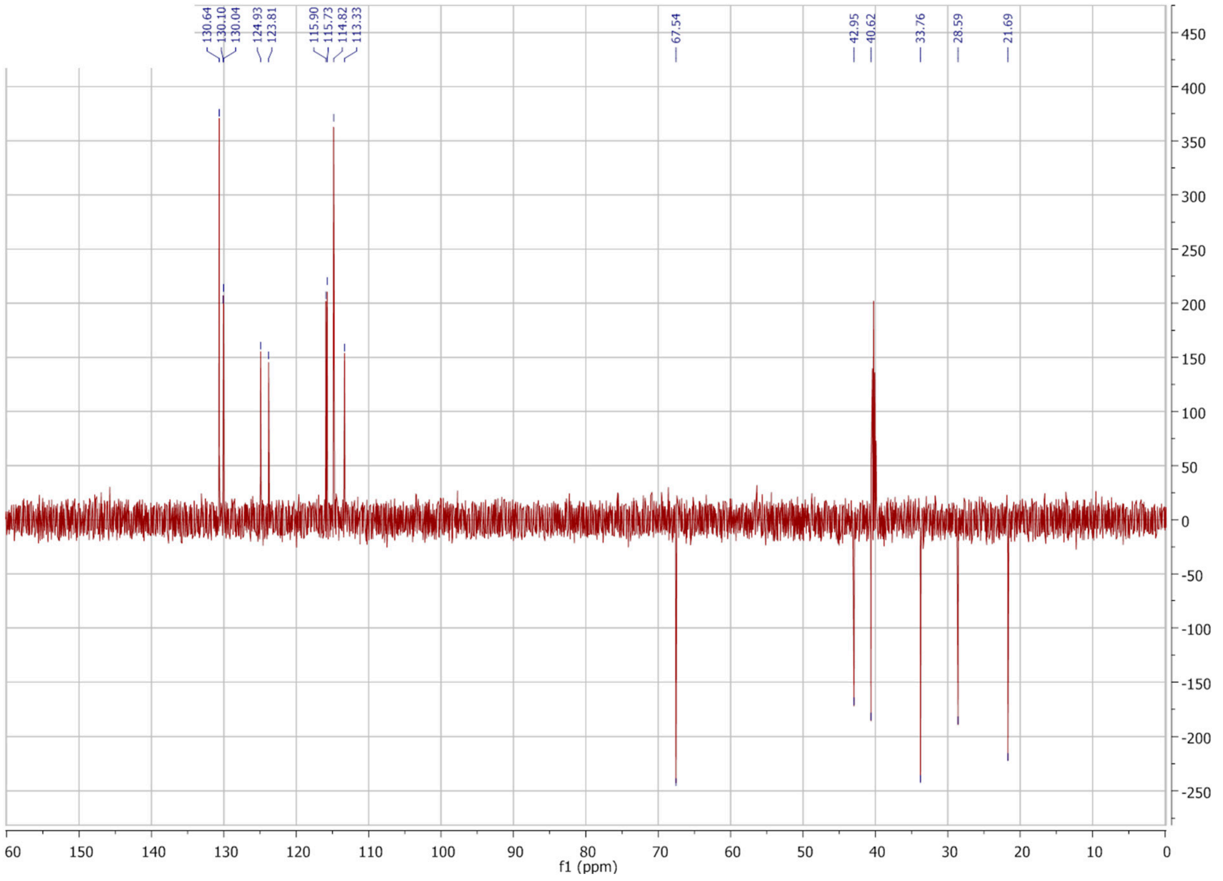

5-{4-[({2-[(4-fluorophenyl)methyl]-1,3-dioxo-2,3-dihydro-1H-isoindol-5-yl}carbamoyl)methyl]phenoxy}pentanoic acid (**5;** ZHAWOC5684)

HRMS

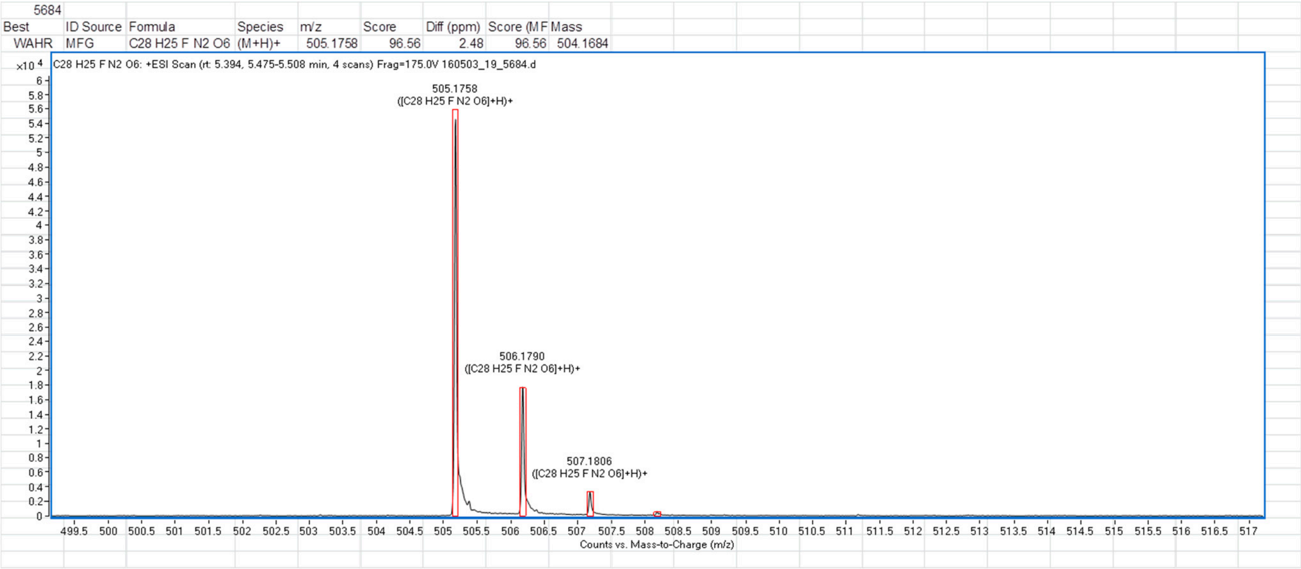

IC50

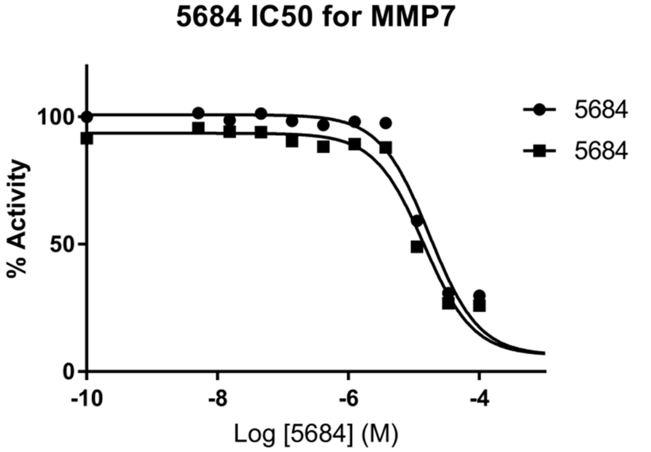

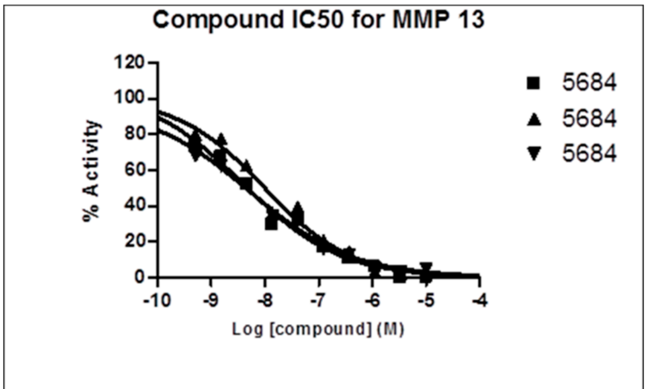

	5684	5684	5684
HILLSLOPE	-0.4789	-0.5361	-0.4540
EC50	3.835e-009	9.790e-009	5.316e-009

6-{4-[({2-[(4-fluorophenyl)methyl]-1,3-dioxo-2,3-dihydro-1H-isoindol-5-yl}carbamoyl)methyl]phenoxy}hexanoic acid (**14m**; ZHAWOC6645)

<u>NMR</u>

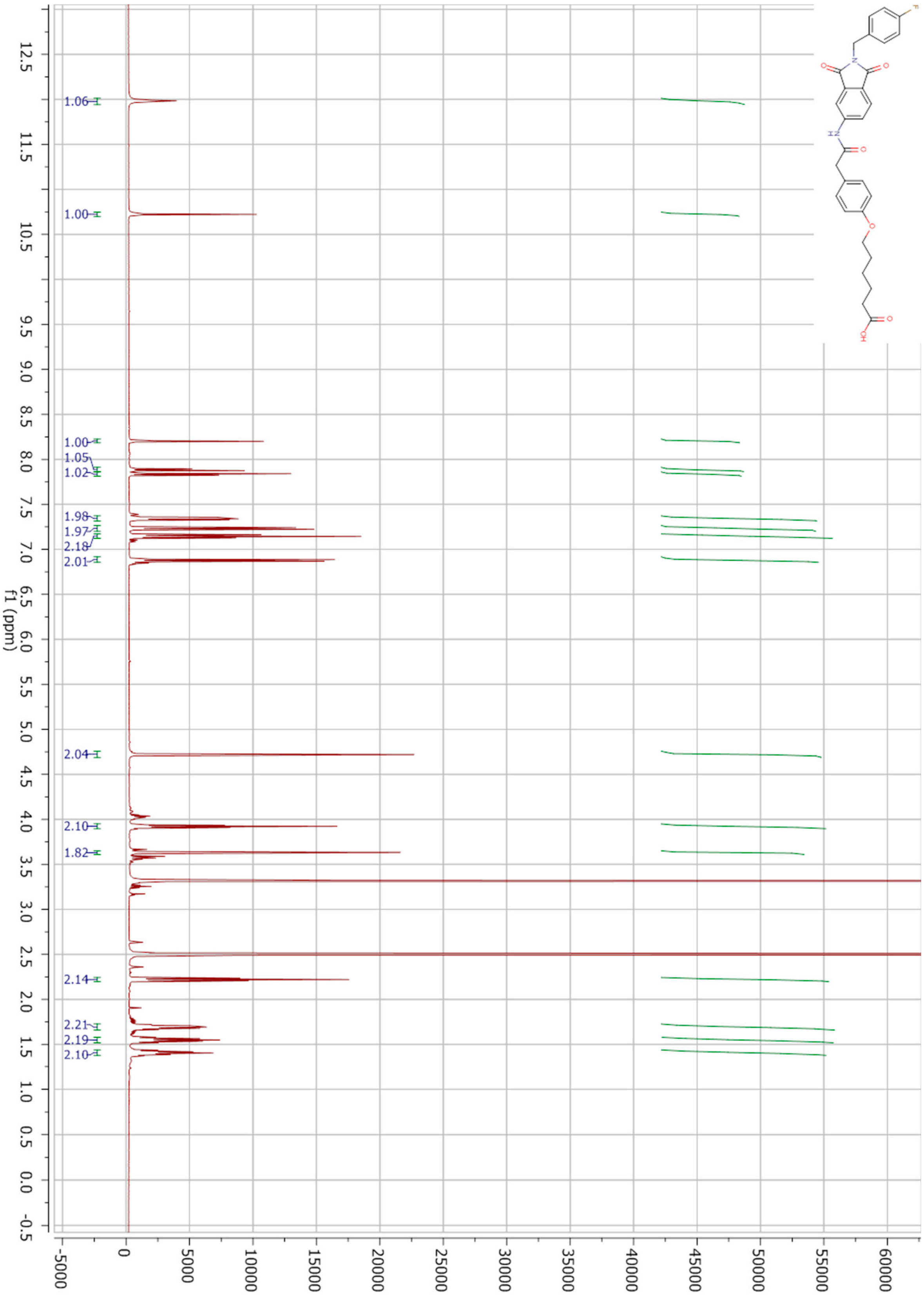

6-{4-[({2-[(4-fluorophenyl)methyl]-1,3-dioxo-2,3-dihydro-1H-isoindol-5-yl}carbamoyl)methyl]phenoxy}hexanoic acid (**14m**; ZHAWOC6645)

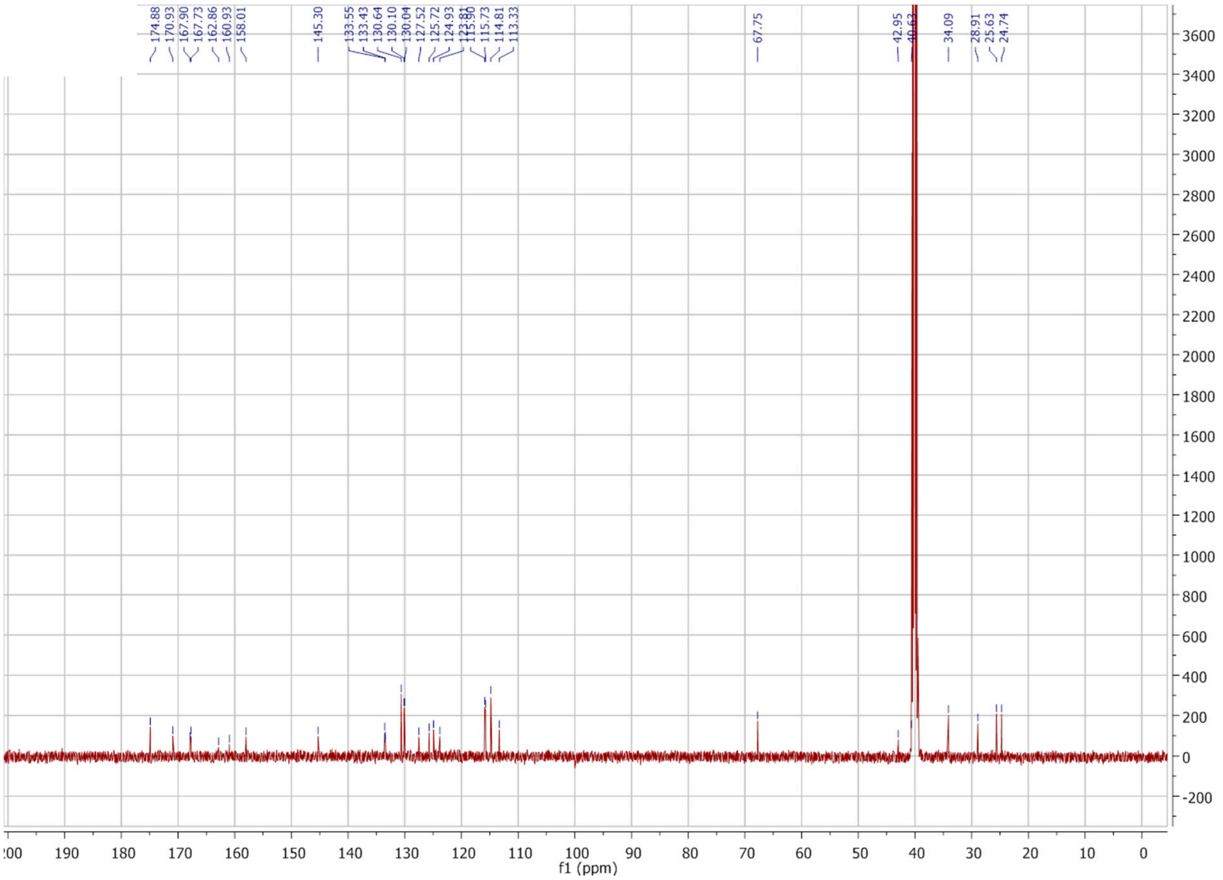

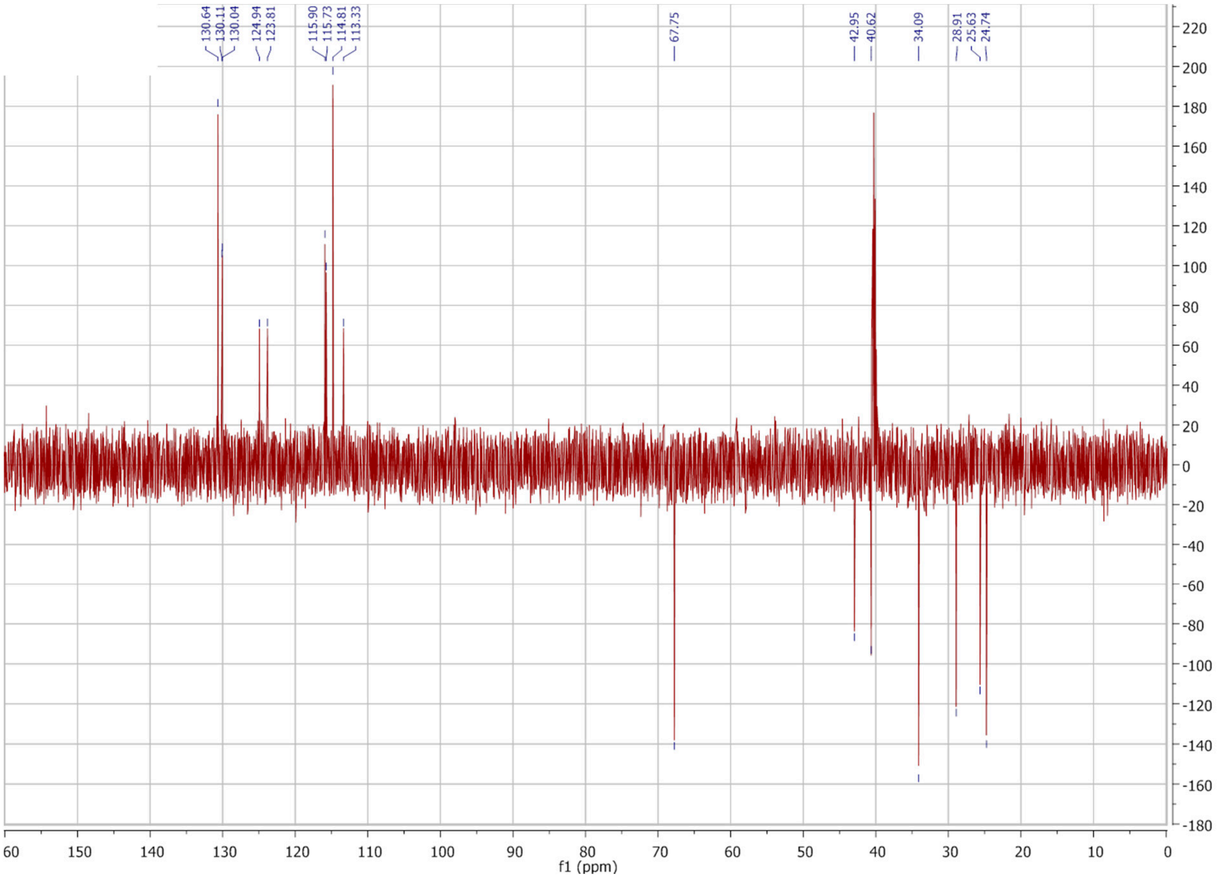

6-{4-[({2-[(4-fluorophenyl)methyl]-1,3-dioxo-2,3-dihydro-1H-isoindol-5-yl}carbamoyl)methyl]phenoxy}hexanoic acid (**14m**; ZHAWOC6645)

HRMS

Best	Formula	Species	m/z	Diff (ppm)	Score (MFG)
WAHR	C29 H27 F	(M+H)+	519.1919	1.56	98.15

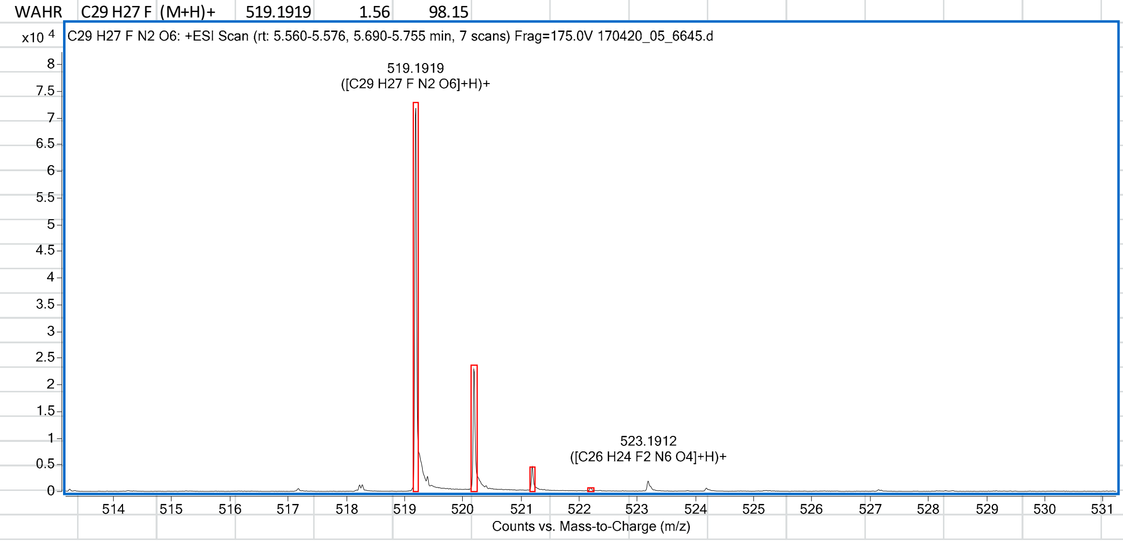

7-{4-[({2-[(4-fluorophenyl)methyl]-1,3-dioxo-2,3-dihydro-1H-isoindol-5-yl}carbamoyl)methyl]phenoxy}heptanoic acid (**14n**; ZHAWOC6637)

NMR

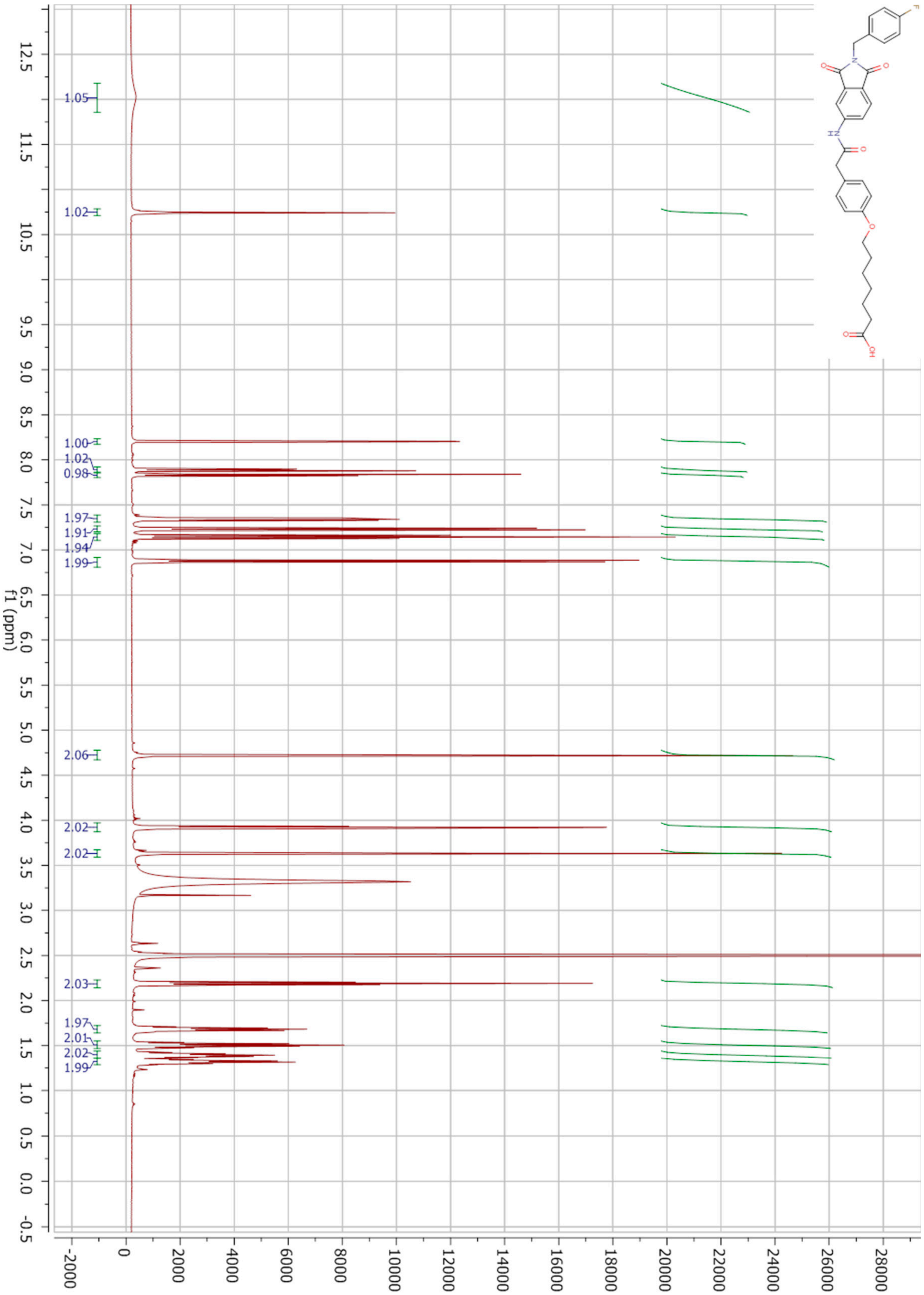

7-{4-[({2-[(4-fluorophenyl)methyl]-1,3-dioxo-2,3-dihydro-1H-isoindol-5-yl}carbamoyl)methyl]phenoxy}heptanoic acid (**14n**; ZHAWOC6637)

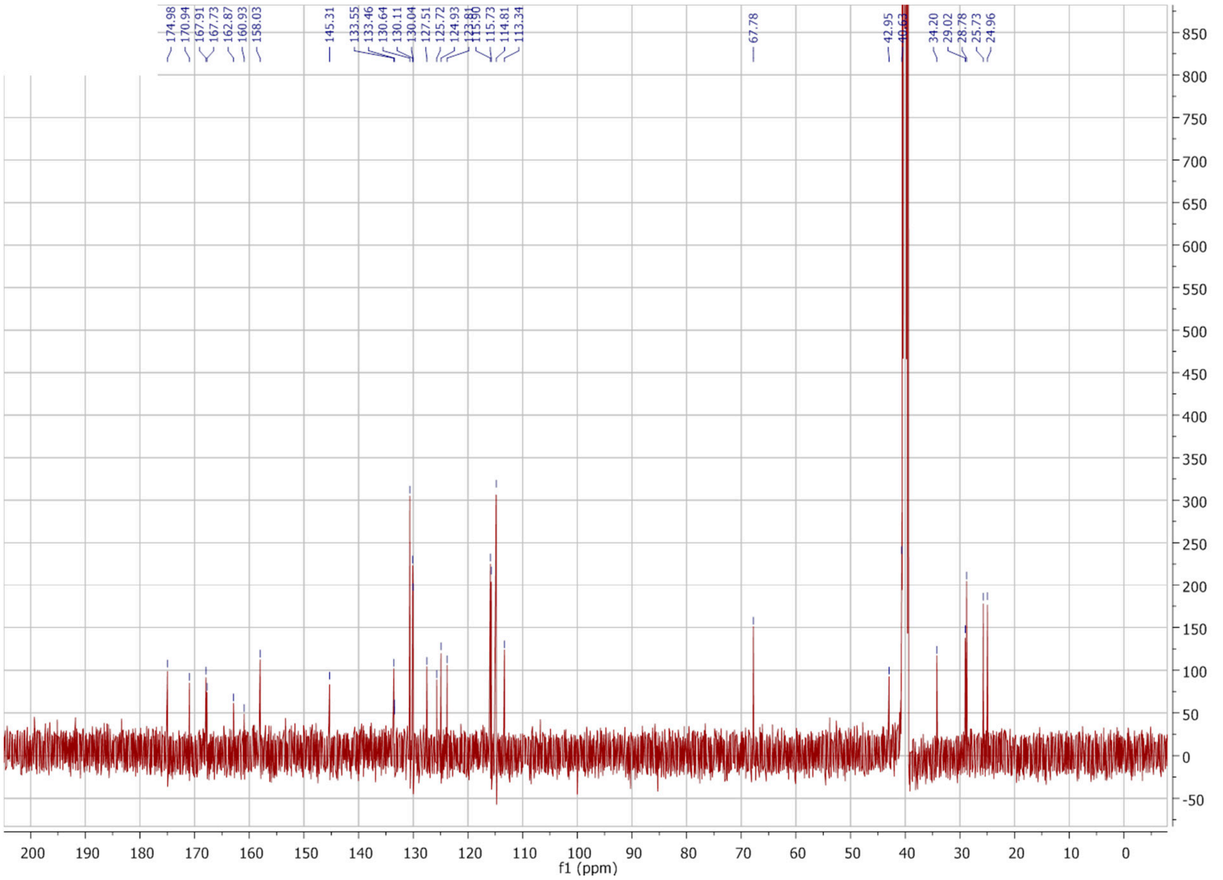

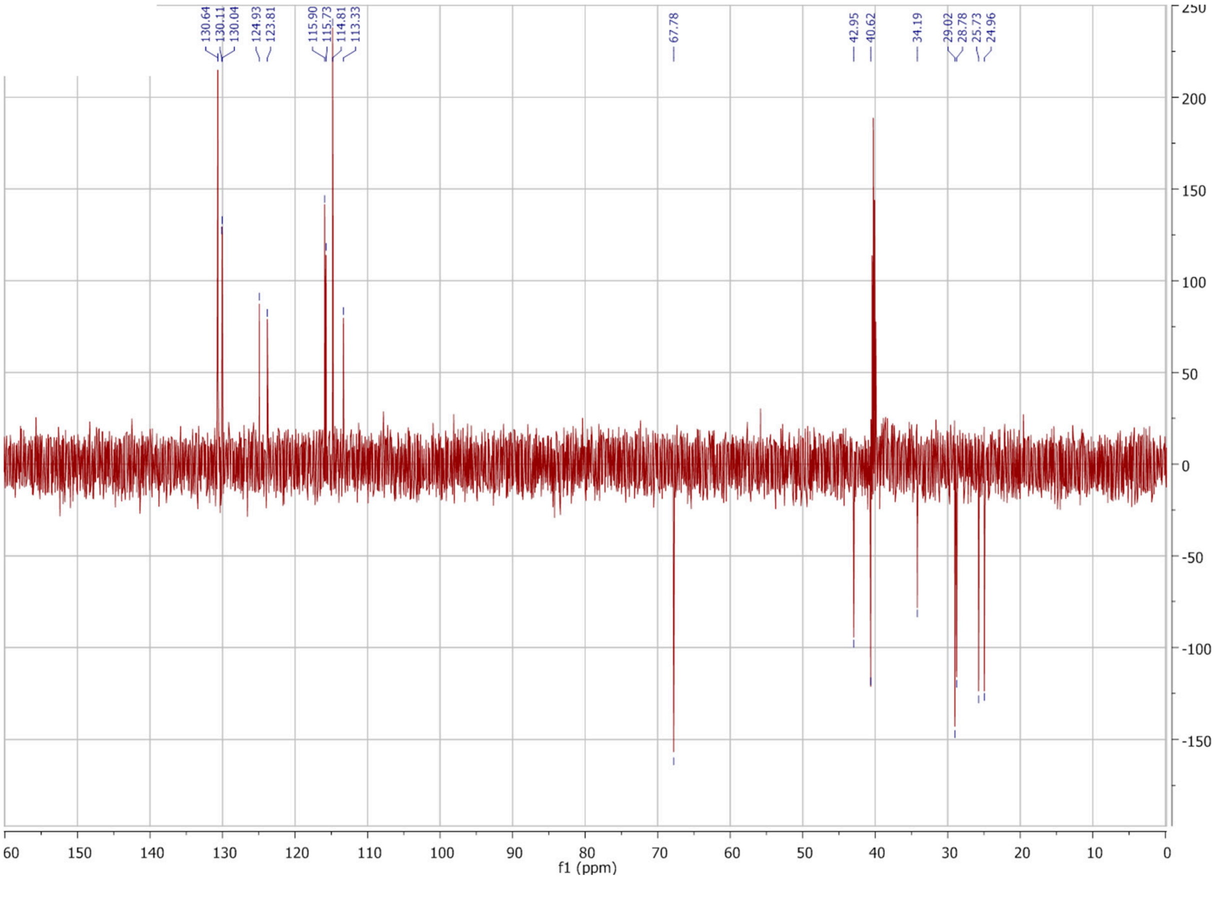

7-{4-[({2-[(4-fluorophenyl)methyl]-1,3-dioxo-2,3-dihydro-1H-isoindol-5-yl}carbamoyl)methyl]phenoxy}heptanoic acid (**14n**; ZHAWOC6637)

HRMS

Best	Formula	Species	m/z	Diff (ppm)	Score (MFG)
WAHR	C30 H29 F	(M+H)+	533.2092	-1.61	98.45

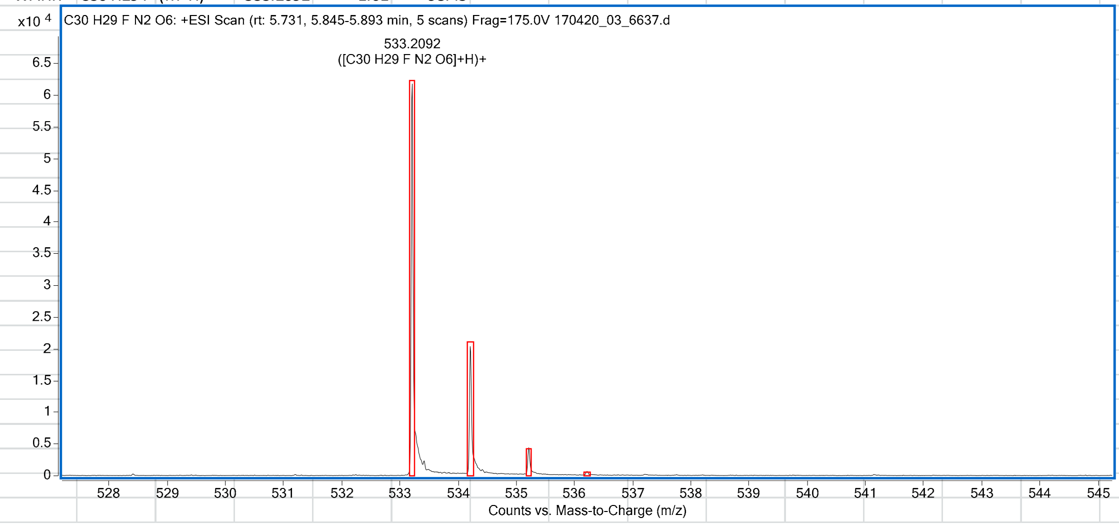

8-{4-[({2-[(4-fluorophenyl)methyl]-1,3-dioxo-2,3-dihydro-1H-isoindol-5-yl}carbamoyl)methyl]phenoxy}octanoic acid (**14o**; ZHAWOC6646)

<u>NMR</u>

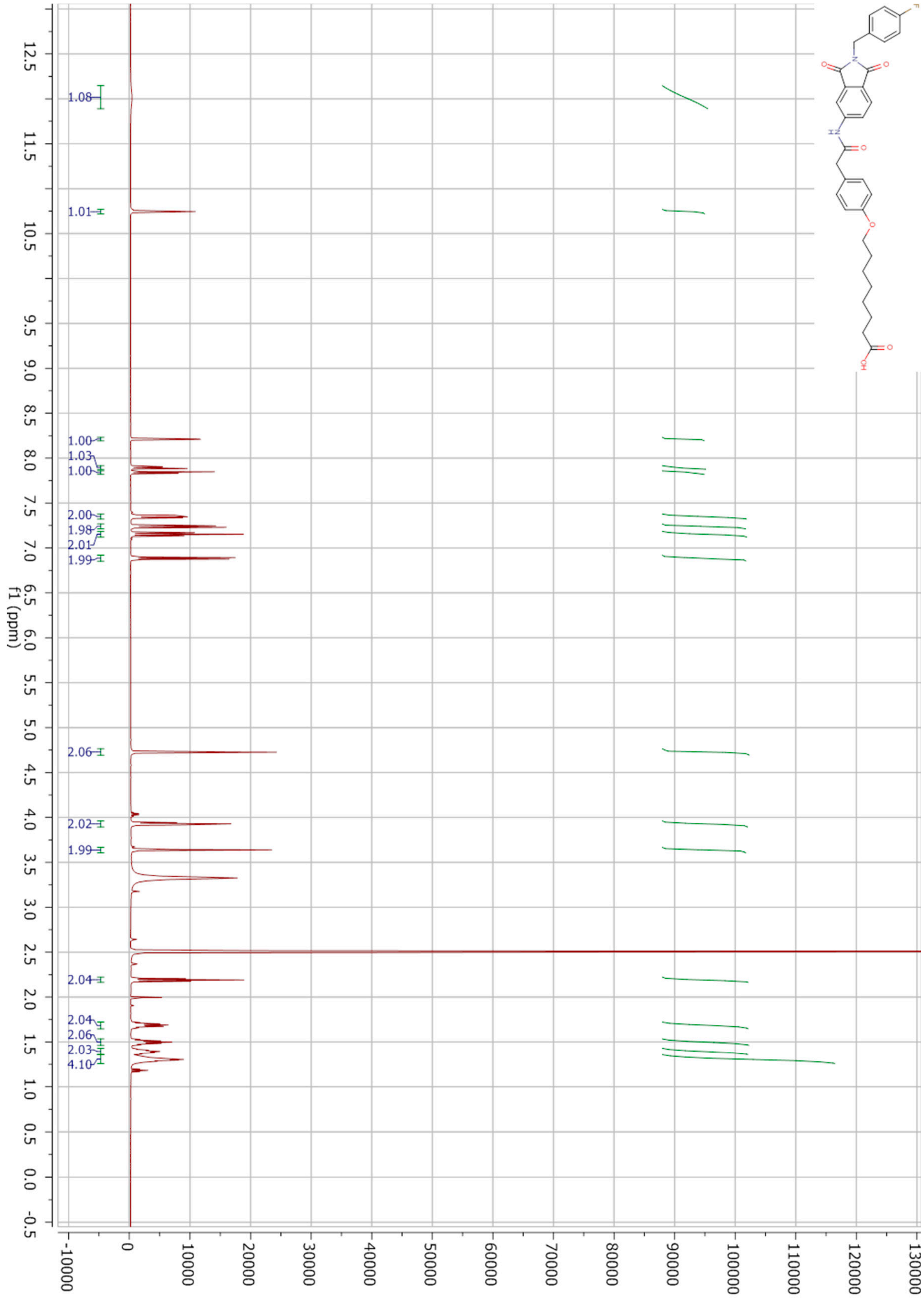

8-{4-[({2-[(4-fluorophenyl)methyl]-1,3-dioxo-2,3-dihydro-1H-isoindol-5-yl}carbamoyl)methyl]phenoxy}octanoic acid (**14o**; ZHAWOC6646)

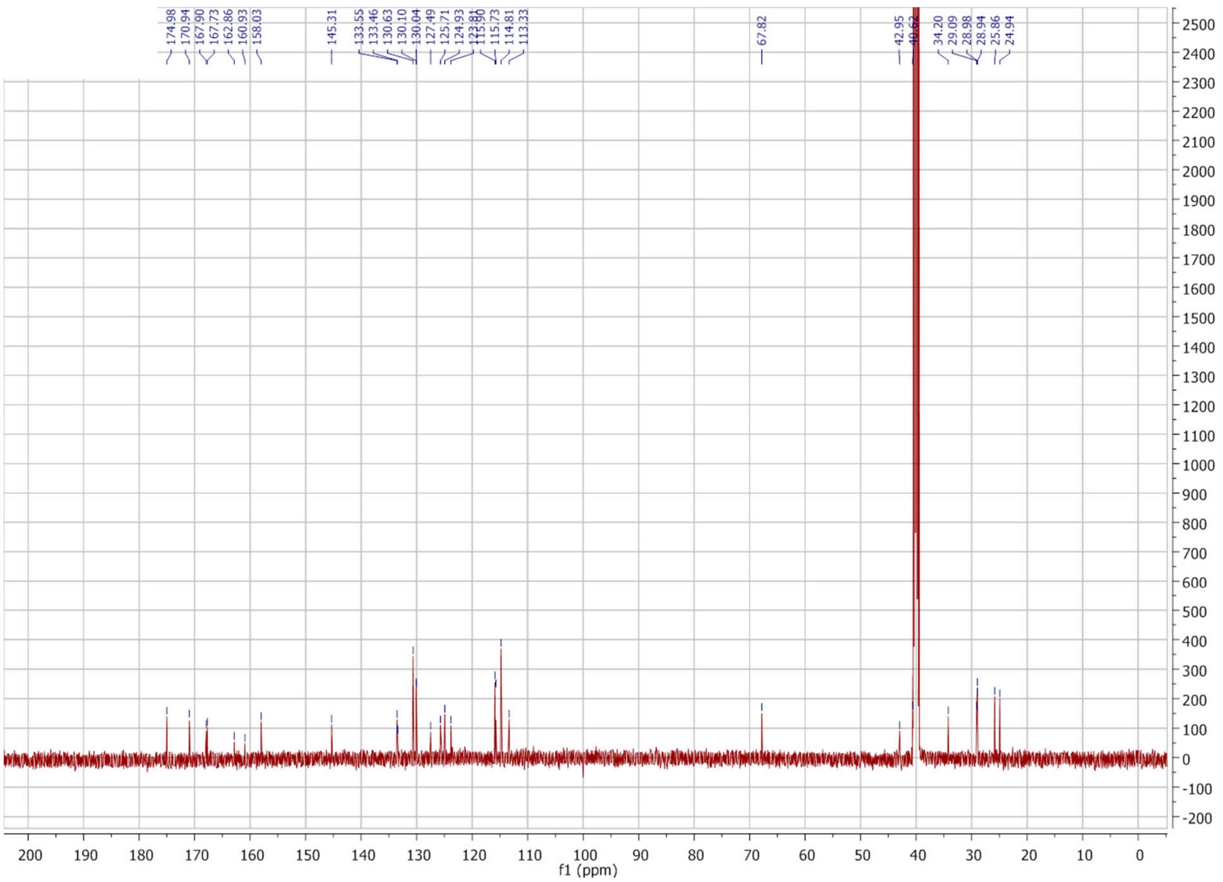

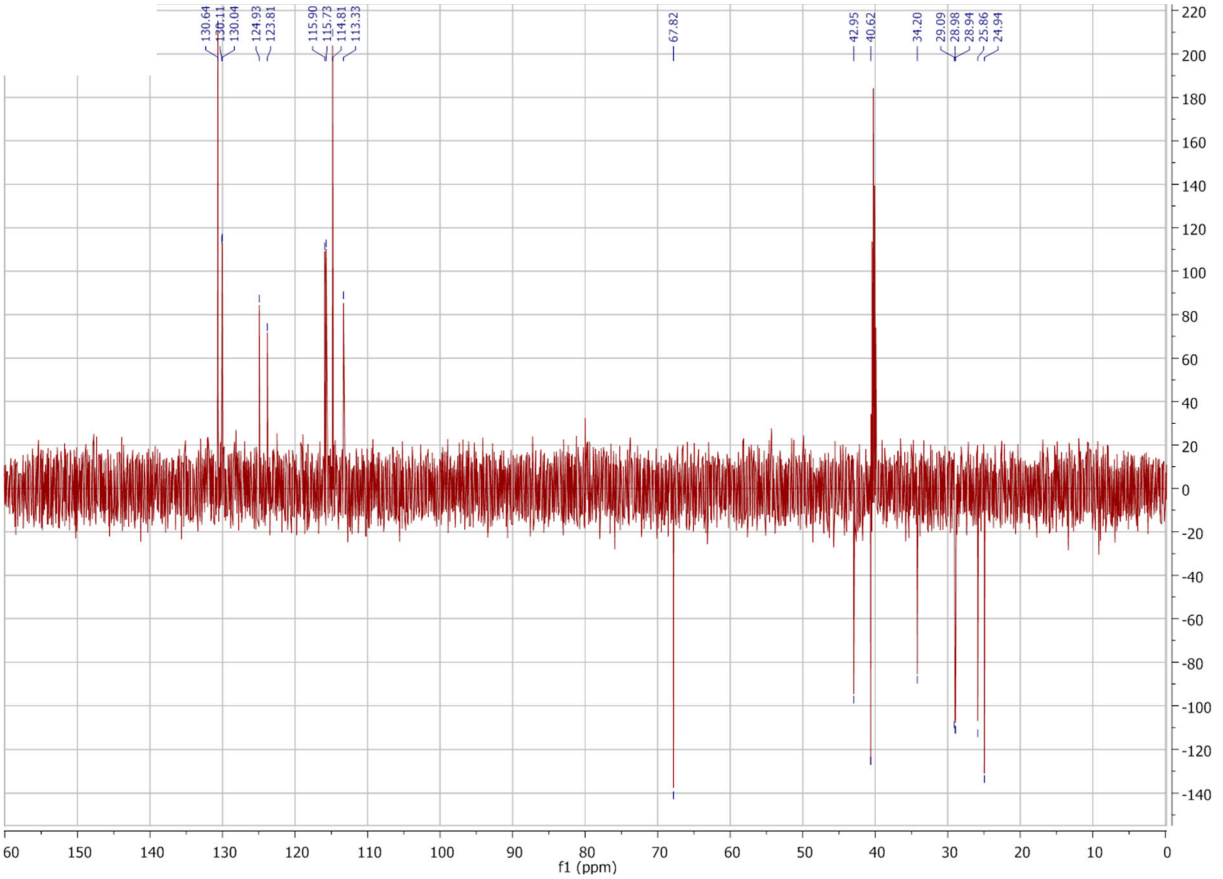

8-{4-[({2-[(4-fluorophenyl)methyl]-1,3-dioxo-2,3-dihydro-1H-isoindol-5-yl}carbamoyl)methyl]phenoxy}octanoic acid (**14o**; ZHAWOC6646)

HRMS

Best	Formula	Species	m/z	Diff (ppm	Score (MFG)
WAHR	C31 H31 F	(M+H)+	547.2248	-1.63	98.24

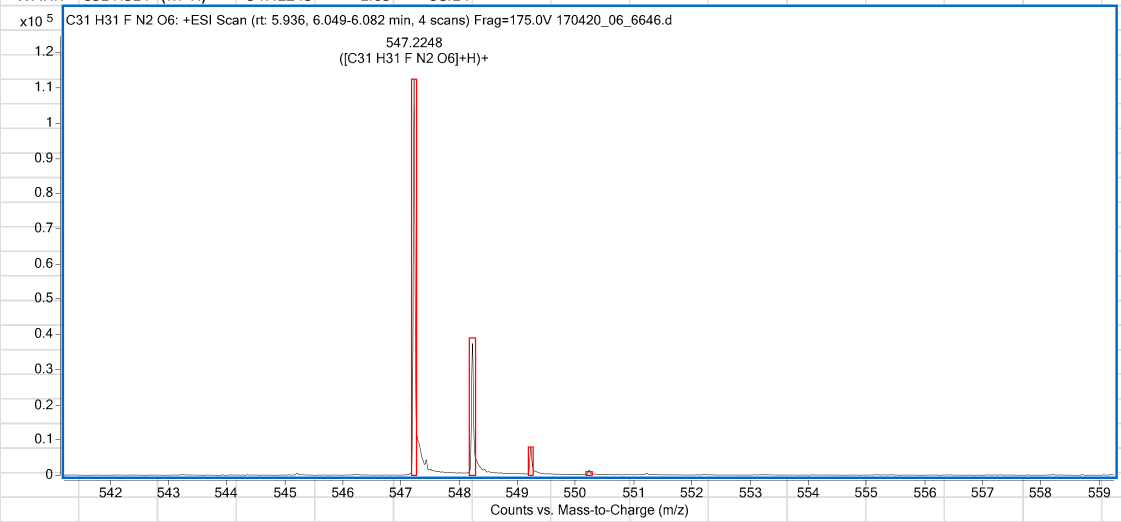

Supplement: Supplementary file 1 [file molecules-22-01548-s001.pdf]
